# Late-stage generation of ^14^C/^3^H-radiolabeled lysine residues via hydroformylation of peptides

**DOI:** 10.1038/s41467-026-74115-8

**Published:** 2026-06-12

**Authors:** Anika Schick, Marc San Jose Gracia, Hans Christian D. Hammershøj, Johan Broddefalk, Pablo Martínez-Pardo, Vitus J. Enemærke, Lena von Sydow, Anna Holub, Ranganath Gopalakrishnan, Kim S. Mühlfenzl, Troels Skrydstrup, Charles S. Elmore

**Affiliations:** 1 Early Chemical Development, Pharmaceutical Sciences, R&D, AstraZeneca, Gothenburg, Sweden; 2 Interdisciplinary Nanoscience Center (iNANO) and Department of Chemistry, Aarhus University, Aarhus, Denmark; 3 Sanofi-Aventis Deutschland GmbH, R&D, Integrated Drug Discovery, Industriepark Höchst, Frankfurt am Main, Germany; 4 Medicinal Chemistry, CVRM, Discovery Sciences, Biopharmaceuticals R&D, AstraZeneca, Gothenburg, Sweden; 5 Medicinal Chemistry, R&I, Discovery Sciences, BioPharmaceuticals R&D, AstraZeneca, Gothenburg, Sweden; 6 Early Chemical Development, Pharmaceutical Sciences, R&D, AstraZeneca, Boston, MA USA

**Keywords:** Organic chemistry, Medicinal chemistry, Catalysis

## Abstract

Peptides constitute a well-established and rapidly expanding field in the contemporary pharmaceutical drug landscape. Studies with ^14^C- or ^3^H-radiolabeled analogs are the gold standard for drug development, yet access to ^14^C-peptides is costly and limited to derivatization of the native structure with tags or lengthy multi-step syntheses. In this work, we report a platform that installs ^14^C- or ^3^H-radiolabeled lysine residues directly on solid-supported peptides. The workflow constitutes a mild, peptide-compatible hydroformylation process of allylglycine residues to generate labeled allysine, followed by reductive amination that furnishes radiolabeled lysine residues directly upon cleavage from the solid support. The hydroformylation setup can be tuned for flexible isotope introduction by using ^14^CO from solid precursors and ^3^H_2_ from standard tritium manifolds. We show that the optimized workflow tolerates diverse sequences and enables functionalization of peptides as complex as semaglutide analogs.

## Abstract (web summary)

Radiolabeled peptides are essential for drug development, but their synthesis is costly and often requires either many steps during peptide elongation or late-stage derivatization. Here, the authors report a solid-phase platform enabling direct installation of ¹⁴C- and ³H-labeled lysine residues via peptide-compatible hydroformylation and reductive amination.

## Introduction

**Fig. 1:**
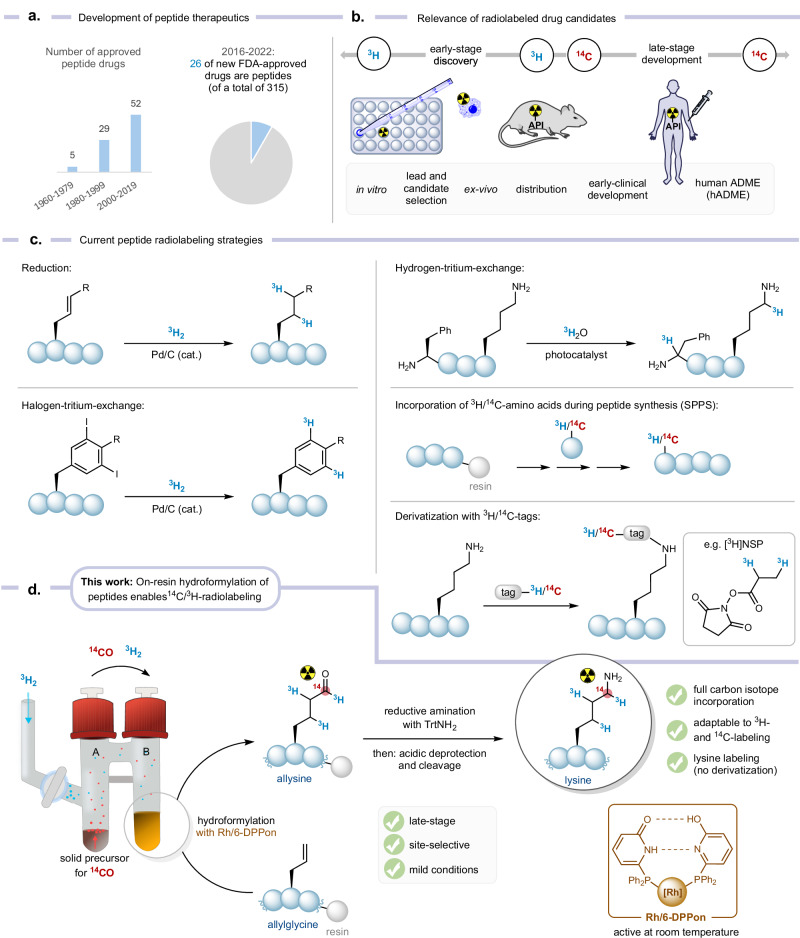
Radiolabeling of peptide therapeutics. **a** Development of peptide therapeutics^2,3^. **b** Use of radiolabeled drug analogs during drug discovery and drug development. **c** Current approaches for ^3^H/^14^C-radiolabeling of peptides. SPPS solid-phase peptide synthesis, [^3^H]NSP *N*-succinimidyl-[2,3-^3^H]propionate. **d** This work: On-resin hydroformylation of peptides under mild conditions. Chamber A—gas release; Chamber B—On-resin hydroformylation of peptides; side-arm for connection to tritium manifold allows ^3^H_2_-introduction, adaptable to ^3^H- and ^14^C-radiolabeling. 6-DPPon 6-diphenylphosphanyl-2-pyridone, TrtNH_2_ tritylamine.

Therapeutic peptides have emerged as a distinct and important class of drug molecules for the treatment of diseases. Bridging the gap between small molecules and biologics, peptides combine high target specificity, favorable safety profiles, and affordable manufacturing costs^1^. Since the first use of insulin as a therapeutic in 1922, peptide therapeutics have experienced accelerated growth in the last couple of decades, with 26 peptide drug approvals from the Food and Drug Administration during 2016–2022 underscoring this rapid development (Fig. 1a)^2,3^. This advancement is exemplified by the active pharmaceutical ingredient semaglutide, which has reshaped treatment options for type 2 diabetes and obesity^4,5^.

The success of effective and safe therapies for patients rests on rigorous drug development programs. These studies are essential yet resource-intensive and can exceed costs of three billion USD. High attrition rates, long investment horizons, and a continually shifting regulatory environment make drug discovery and development a risky investment^6,7^. Attrition is high across the different stages of development. AstraZeneca data (2001–2010) indicated that over 20% of pipeline candidates were halted in the preclinical stage mainly due to toxicity^8^, and industry-wide data (2010–2017) showed 90% failure in the clinical stage attributed predominantly to clinical efficacy and toxicity^9^. The implication is clear: earlier insights into the safety profile would weed out risky drug candidates, protect resources, and advance promising treatment options^8,9^.

Radiolabeled drug analogs contribute to understanding the drug profile, from early discovery to late-stage development (Fig. 1b). The synthesis of these radiolabeled analogs as performed by radiochemistry teams, remains a critical bottleneck for the pipeline. Tritiated analogs are routinely employed in early in vitro discovery, including binding assays and receptor occupancy studies for lead identification^10,11^. Current peptide radiolabeling strategies (Fig. 1c) are versatile for tritium-tracers^12^, including (i) metal-mediated reduction of unsaturated motifs^13,14^, (ii) halogen-tritium exchange of aryl halides^15^, or (iii) hydrogen-isotope exchange on aliphatic amides, side chains, and amino acid residues^16–18^, for example via photoredox chemistry^19^. Other strategies comprise (iv) incorporation of radiolabeled amino acids during solid-phase peptide synthesis (SPPS)^20^, or (v) conjugation of tags to lysine, arginine, or the *N*-terminus using reagents such as *N*-succinimidyl-[2,3-^3^H]propionate ([^3^H]NSP)^21–23^. For example, ^3^H-semaglutide was prepared in a two-step labeling approach. First, a fatty diacid linker was tritiated, followed by site-selective coupling to lysine residue 20. Although the strategy was effective in this case, it is not general but dependent on the linker and amino acid sequence in each peptide drug^13^.

^14^C-labeled analogs are integral to preclinical and clinical studies where precise quantification, mass-balance, and metabolite analysis are required to evaluate the absorption, distribution, metabolism, and excretion profile of the drug candidate (Fig. 1b)^10^. In contrast to ^3^H-labeling, broadly applicable late-stage ^14^C-labeling strategies of peptides remain limited and are often confined to incorporation of ^14^C-amino acids during SPPS^24^ or conjugation with ^14^C-tags (Fig. 1c)^25^. Emerging ^13^C-carbonylation strategies (including aminocarbonylation and thiocarbonylation) show promise^26,27^, but may struggle with site selectivity and derivatization-restrictions depending on the peptide sequence. Accordingly, the pharmaceutical industry experiences a pressing need to expand the ^14^C-labeling toolbox of radiochemists with late-stage, site-selective peptide labeling methodologies without relying on derivatization.

Lysine is widely prevalent in peptide sequences^28^, making it an attractive target for radiolabeling strategies that aim for broad applicability across peptide sequences. Allysine, a residue which contains a formyl functionality in place of the amine present in lysine, offers a handle for chemoselective peptide diversification. Recent oxidative and electrochemical methodologies enable its synthetic installation within peptides^29–32^, which expands the toolbox to synthesize peptide analogs in discovery^28,33–36^. We envisioned that the lysine residue could be formed by reductive amination of allysine, which in turn could be synthesized by a hydroformylation process (HFP) of an allylglycine residue directly within peptides. The forward synthesis of this workflow can be seen in Fig. 1d. Site-selective incorporation of allylglycine is straightforward using the commercial Fmoc-protected building block during SPPS^37^, and has also been achieved using synthetic biology approaches^38^. Hydroformylation has evolved as a powerful chemical transformation for the conversion of olefins into aldehydes using CO and H_2_ gas (syngas) under metal catalysis, and is employed for the industrial synthesis of oxo-products^39,40^. The HFP has been recently exploited to install stable non-radioactive carbon or hydrogen isotope labels into small molecules using ^13^CO/^2^H_2_^41,42^. However, the hydroformylation reaction has not yet been reported to install radioisotopes on peptides. Thus, we envisioned developing a method to address this gap. ^3^H_2_ is routinely available from tritium manifolds in standard radiochemistry labs^43^, and ^14^CO can be generated via solid precursors as developed by the Elmore and Skrydstrup group^44–47^ or via photocatalytic CO_2_ reduction as developed by the Audisio group^48^. The highly active catalytic system Rh/6-DPPon for hydroformylation was introduced by Breit, and is based on the self-assembling, hydrogen-bonded bidentate ligand 6-diphenylphosphanyl-2-pyridone (6-DPPon) (Fig. 1d)^49^. Rh/6-DPPon delivers excellent regioselectivity toward the desired linear aldehydes, operates at room temperature and ambient pressure, and is compatible with aqueous media^50^. Thus, Rh/6-DPPon offers mild conditions amenable for the envisioned hydroformylation reaction and application to radioisotopes and peptide substrates.

In this work, we report the installation of radiolabeled lysine residues directly within peptides (Fig. 1d). During the HFP, the allylglycine residue is converted to allysine under mild, peptide-compatible conditions. Subsequent reductive amination with tritylamine and acidic deprotection/ cleavage furnishes the ^3^H/^14^C-radiolabeled lysine residue in the native peptide sequence, thereby achieving late-stage labeling, site-selectivity, and broad applicability by installing a labeled natural amino acid. To streamline near-stoichiometric syngas use during hydroformylation, the two-chamber setup developed by Skrydstrup and co-workers is modified^42^. The platform provides a direct entry point for seamless ^3^H/^14^C-isotope incorporation from ^14^CO or ^3^H_2_, both standard in radiochemistry labs. While ^14^C-labeling of peptides was previously typically limited to the use of ^14^C-amino acids during multi-step SPPS or to late-stage derivatization with ^14^C-tags, our platform offers late-stage generation of ^14^C-labeled lysine residues. We anticipate that this generalizable workflow will lower the barrier to tracer access, reduce radiochemical waste, improve efficiency across discovery and development, and ultimately accelerate the peptide therapeutics pipeline.

## Results and discussion

### Initial hydroformylation reactions

**Fig. 2:**
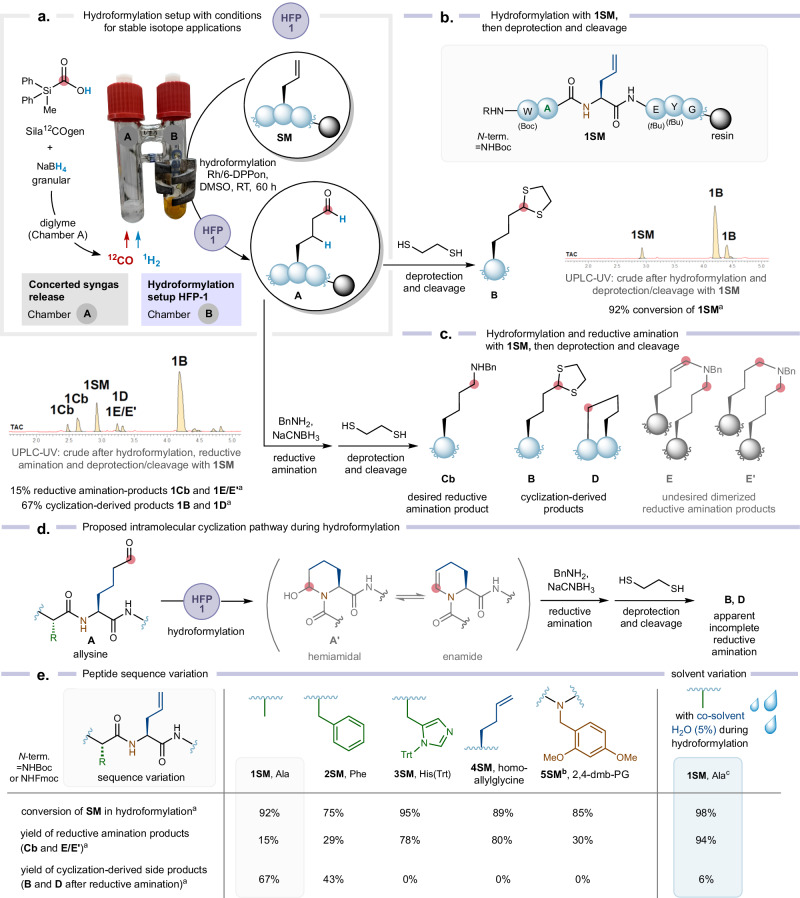
Development of a peptide-compatible hydroformylation process (HFP). **a** Hydroformylation reaction setup HFP-1 for stable isotope applications using a standard two-chamber system with concerted syngas release. **b** Hydroformylation with **1SM** to form thioacetal **B** upon cleavage. **c** Reductive amination giving minor reductive amination products while forming major cyclization-derived products **B** and **D**. **d** Proposed intramolecular cyclization leading to apparent incomplete reductive amination, due to formation of hemiamidal **A’**/enamides. The site of cyclization is suggested but was not analyzed in detail. **e** Dependence of cyclization on the peptide sequence. 2,4-dmb-PG 2,4-dimethoxybenzyl protecting group. Reaction conditions: Hydroformylation process HFP-1: substrate (ca. 2.5–6 µmol), Rh/6-DPPon (7 µmol), CO/H_2_ (0.2 mmol, ex situ generation), DMSO (1 mL), RT, 60 h. Rh/6-DPPon, from Rh(COD)_2_BF_4_/6-DPPon (1:5) pre-mixed in solvent. Reductive amination: aldehyde **A** (small aliquot of resin), benzylamine in CH_2_Cl_2_, 1% AcOH (0.12 M, 0.5 mL), RT, 2 h, NaCNBH_3_ in CH_2_Cl_2_:MeOH, 3:1 (0.15 M, 0.5 mL), RT, 1 h. Deprotection and cleavage: TFA:EDT (ethane-1,2-dithiol):H_2_O (92.5:5:2.5), 3 h, RT. See Supporting Information for detailed product isomer ratios. ^a^Conversion and yields are determined by UPLC-UV purity analysis. ^b^**5SM** was impure and had a very low loading on the resin. ^c^Using DMSO:H_2_O (95:5) (1 mL) during hydroformylation with half the amount of **1SM**.

Inspired by precedent hydroformylation of small molecules^42^, we set out to bring the hydroformylation reaction onto easily accessible, allylglycine containing peptides **SM** directly on-resin, to create allysine **A** under peptide-compatible conditions (Fig. 2a). To enable routine, safe peptide hydroformylation conditions without the need for a gas cylinder, we adopted a two-chamber setup in which syngas can be generated ex situ in chamber A and consumed in the hydroformylation reaction occurring in chamber B. For routine stable-isotope applications (^12^C, ^13^C, ^1^H), reactions were performed in a commercial COware**®** reactor, allowing concerted syngas generation from the solid precursors Sila^12^COgen (or Sila^13^COgen) and a hydrogen gas surrogate (Fig. 2a, setup HFP-1). We adapted the protocol by replacing the previously reported hydrogen gas surrogate conditions^42^ with granular NaBH_4_ in diglyme, providing a practical and accessible protocol for routine applications. To avoid isotope dilution from unlabeled ^12^CO during ^13^C- or ^14^C-labeling, the hydroformylation pre-catalyst Rh(COD)_2_BF_4_ was used in place of the previously employed Rh(CO)_2_acac^42^. The reactions were gently shaken to protect the TentaGel®/polystyrene resins, and DMSO as the reaction medium provided high conversion of **SM** to allysine **A**. On the model substrate **1SM**, test scale hydroformylations combined with direct acidic cleavage using the scavenger ethane-1,2-dithiol (EDT) revealed a clean UPLC-UV profile (Fig. 2b). A 92% conversion of **1SM** allowed the formation of thioacetals **1B** (two isomers) as the sole products, serving as a read-out for allysine **A** formation (see Supporting Information, Section 2.1 for solvent optimization). The major isomer **1B** was assigned as the linear thioacetal, while the minor UPLC-UV signal likely corresponds to branched thioacetal isomers that are formed from branched aldehydes (side product during hydroformylation, see Supporting Information for isomer details).

### Investigation and mitigation of side product formation

Subjecting the product from the hydroformylation of **1SM** to solid-supported reductive amination using benzylamine initially revealed a complex UPLC-UV profile of the crude reaction mixture (Fig. 2c). We quantified the product distribution by UPLC-UV purity comparing test cleavages after hydroformylation and reductive amination. After reductive amination, several products were identified in the product mixture. Along with the desired benzylated lysine **1Cb**, a suite of side products composed predominantly of thioacetal **1B** plus small amounts of a reduced cyclized species **1D** (combined yield of **1B** and **1D** 67%), together with cross-linked dimers **1E/E’**. This left only 15% combined yield of the reductive amination products **1Cb** plus undesired **1E/E’**. The reaction outcome suggested two causes. The initially formed secondary amine **Cb** remained sufficiently nucleophilic enabling a second reductive amination with proximal aldehydes on the resin, producing dimers **E/E’**. The major presence of **B** after reductive amination was puzzling and could be at first glance interpreted as an unproductive conversion of **A** to **Cb** during reductive amination. However, we observed that the relative amounts of **B** plus **D** vs. reductive amination products **Cb** plus **E/E’** were influenced by several factors, including the hydroformylation reaction times and temperatures, the catalytic system, and the peptide sequence (see Supporting Information, Sections 2.2 and 2.3). Previously, Szewczuk  and co-workers reported on resin-supported peptides containing ethylene acetal-protected allysine residues that led to cyclization upon acidic deprotection and cleavage from the resin^51^. We propose that during the hydroformylation the newly formed on-resin allysine **A** can engage intramolecularly with its backbone amide. We suggest that this likely forms a six-membered hemiamidal **A’**/enamide that resists reductive amination under the applied conditions, thereby halting the reaction sequence and resisting the ultimate lysine production (Fig. 2d). Hence, only small yields of the reductive amination product **Cb** (plus **E/E’**) could be obtained. Instead, major amounts of the thioacetal **B** together with smaller amounts of the reduced cyclized species **D** were formed after reductive amination and acidic cleavage with EDT. In combination with the general detection of only thioacetal **B** and the starting material **SM** in the hydroformylation crude, we hypothesize that EDT traps the cyclized species **A’** to form thioacetal **B** either by direct engagement or indirectly via an equilibrium with allysine **A** under the harsh cleavage conditions.

To demonstrate the influence of the peptide sequence on the proposed cyclization, we followed the reaction workflow using different hexapeptides (Fig. 2e). The cleavage conditions were critical for an accurate quantification. Using EDT as the sole scavenger in the cleavage cocktail (TFA:EDT:H_2_O, 92.5:5:2.5) consistently afforded clean and interpretable UPLC-UV profiles. Cyclization was assessed by comparing the yields of cyclization-derived products **B** and **D** vs. reductive amination products **Cb** and **E/E’** after reductive amination. A survey with three peptides bearing different amino acids adjacent to the reaction center revealed sequence-dependent trends in the product composition. While the conversion of substrates **1SM,** **2SM**, and **3SM** was high for each peptide (75–95%), the influence on the product balance was remarkable. The yield of the reductive amination products **Cb** plus **E/E’** increased with steric bulk of the residues adjacent to the reactive allylglycine (**1SM** (Ala), 15%; **2SM** (Phe), 29%; **3SM** (His(Trt)), 78%), while the yield of the proposed cyclization-derived products **B** plus **D** decreased (**1SM** (Ala), 67%; **2SM** (Phe), 43%; **3SM** (His(Trt), 0%). Extending the distance from the reaction site to the backbone by employing homo-allylglycine (**4SM**) instead of allylglycine similarly suppressed cyclization (Fig. 2e). This trend aligns with literature precedence from Robinson and co-workers, who observed facile six-membered enamide/hemiamidal formation upon hydroformylation of *N*-acetyl allylglycine at 80–100 °C, whereas seven-membered ring formation required harsher conditions^52^. To address peptide sequences prone to cyclization, we imposed backbone-protection using the 2,4-dimethoxybenzyl (dmb) group on the amide nitrogen adjacent to allylglycine (**5SM**), which effectively eliminated the detection of **B** and **D** after reductive amination.

The synthesis of the backbone-protected precursor **5SM** itself gave poor yields in low purity (see Supporting Information) and could have been further optimized for routine-applications^53^. Instead, we focused on identifying a more practical operational lever than backbone-protection to suppress cyclization and to increase the yield of reductive amination products. To achieve this, we examined the solvent composition. Simply switching from neat DMSO to a mixture of 5% water in DMSO maintained high conversion while minimizing cyclization (Fig. 2e). This solvent mixture was therefore adopted as the standard medium to favor formation of reductive amination products. Using tritylamine as an ammonia equivalent has been previously described by Castellino and co-workers^54^. To further improve the reaction workflow, we substituted benzylamine with the bulkier tritylamine (Fig. 1d) for two reasons. First, this provides the free lysine residue directly upon acidic cleavage. Secondly, it inhibits undesired dimerization, likely because the bulky trityl group prevents further reaction with other aldehydes on resin (see Supporting Information, Sections 2.3 and 2.4). For stable-isotope applications such as diversification, where less hindered amines may be required, lowering the resin loading should minimize intermolecular encounters. We did not pursue this in our work and focused instead on radiolabeling applications of the non-derivatized natural amino acid lysine.

### Optimization on larger reaction scale

**Fig. 3:**
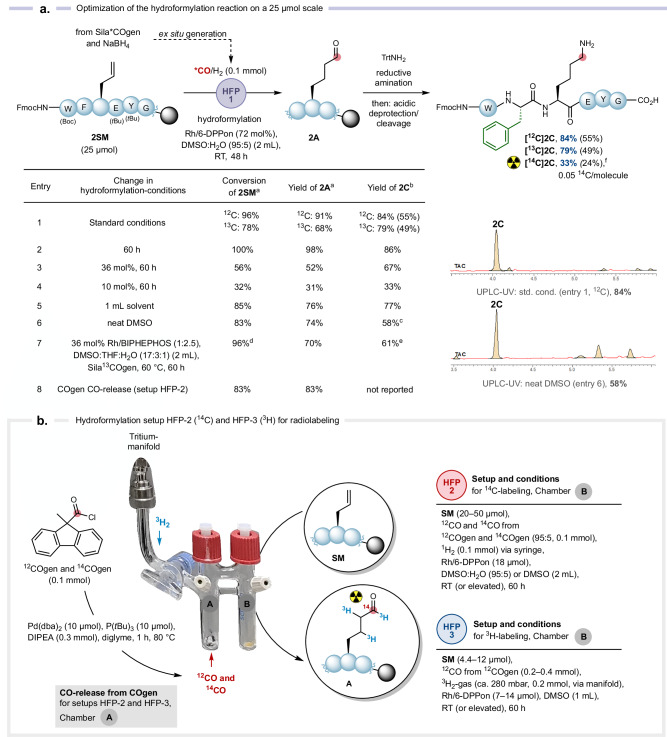
Optimization of the hydroformylation reaction on a 25 µmol reaction scale and radiolabeling setup. **a** Optimization on 25 µmol scale with **2SM** using hydroformylation process HFP-1. Yield determined by UPLC-UV purity analysis. Isolated yields are given in parentheses. Please see Supporting Information for experimental details and product isomer ratios. Reaction conditions: Hydroformylation process HFP-1: Chamber A—Sila^12^COgen or Sila^13^COgen (0.1 mmol), granular NaBH_4_ (0.1 mmol), diglyme (1 mL). Chamber B—**SM** (ca. 25 µmol), Rh/6-DPPon (18 µmol), DMSO:H_2_O (95:5, 2 mL), RT, 48 h. Reductive amination: tritylamine in DMSO, 10% AcOH (0.12 M), RT, 2 h, then NaCNBH_3_ in CH_2_Cl_2_:MeOH, 3:1 (0.15 M), RT, 1 h. Deprotection and Cleavage: TFA:H_2_O:TiPS (triisopropylsilane):DODT (3,6-dioxa-1,8-octanedithiol) (92.5:2.5:2.5:2.5), RT, 3 h. **b**. Hydroformylation reaction setup and conditions for ^14^C- (hydroformylation process HFP-2) and ^3^H-labeling (hydroformylation process HFP-3) using a custom-made two-chamber system that can be attached to the tritium manifold. Rh/6-DPPon, from Rh(COD)_2_BF_4_/6-DPPon (1:5) pre-mixed in solvent. ^a^Determined by cleaving a test-portion of the resin after hydroformylation with TFA:EDT:H_2_O (92.5:5:2.5), RT, 3 h, and analyzing thioacetal **B** and remaining **SM** by UPLC-UV purity. ^b^Yield of **2C** determined by UPLC-UV purity analysis after cleavage with TFA:H_2_O:TiPS:DODT (92.5:2.5:2.5:2.5). ^c^The remaining signals in the UV-chromatogram were **2SM** and side products derived from unreacted/cyclized aldehyde(-analogs). ^d^96% conversion of **2SM**, but formation of 19% peptide with hydrogenated allylglycine residue. ^e^Test cleavage with TFA:EDT:H_2_O (92.5:5:2.5), RT, 3 h, shows presence of 10% remaining **[**^**13**^**C]2B**. ^f^HFP-2 setup, 50 µmol scale, ^12^CO:^14^CO (95:5, 0.1 mmol), ^1^H_2_ (0.1 mmol), Rh/6-DPPon (18 µmol), 60 h; 13% RCY.

**Fig. 4:**
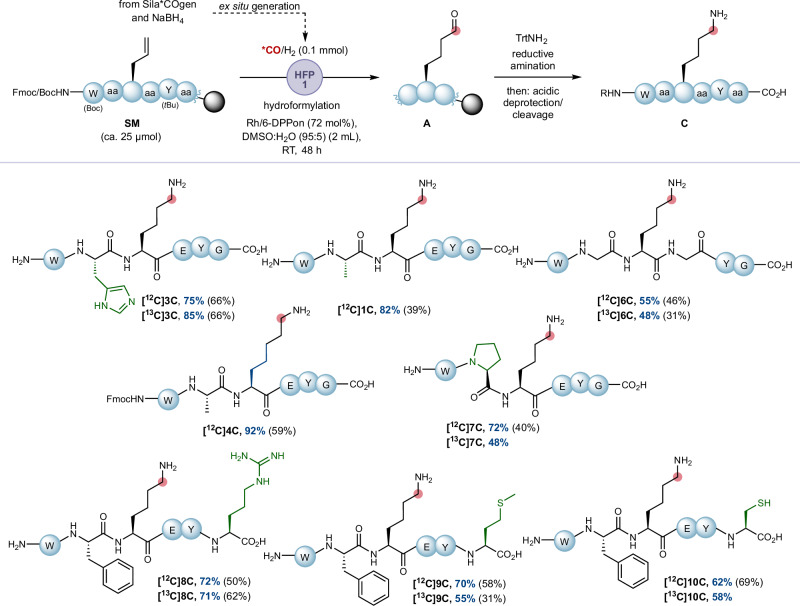
Hexapeptide product scope. Yields determined by UPLC-UV purity analysis. Isolated yields are given in parentheses. See Supporting Information for experimental details and product isomer ratios. Aa amino acid. Reaction conditions: Hydroformylation process HFP-1: Chamber A—Sila^12^COgen or Sila^13^COgen (0.1 mmol), granular NaBH_4_ (0.1 mmol), diglyme (1 mL). Chamber B—**SM** (ca. 25 µmol), Rh/6-DPPon (18 µmol), DMSO:H_2_O (95:5, 2 mL), RT, 48 h. Reductive amination: tritylamine in DMSO, 10% AcOH (0.12 M), RT, 2 h, then NaCNBH_3_ in CH_2_Cl_2_:MeOH, 3:1 (0.15 M), RT, 1 h. Deprotection and cleavage: TFA:H_2_O:TiPS:DODT (92.5:2.5:2.5:2.5), RT, 3 h.

With critical parameters for the hydroformylation reaction in place, we finalized the reaction conditions on preparative reaction scale (ca. 25 µmol), allowing the use of 4 equivalents of syngas to reduce radiochemical waste and to enable efficient downstream purification (Fig. 3a). Under the shown conditions using hydroformylation process HFP-1, the model substrate **2SM** furnished the corresponding allysine **2A** (read-out via thioacetal **2B**) in 91% yield, with 96% conversion of **2SM**. Following reductive amination, general deprotection and cleavage from the solid support, **[**^**12**^**C]2C** was delivered in an 84% yield (55% isolated) and **[**^**13**^**C]2C** in a 79% yield (49% isolated), showcasing the transferability of this method to ^13^C-labeled peptides (Fig. 4a, entry 1) (UPLC-UV profile of **[**^**12**^**C]2C** reaction shown in the upper chromatogram). Extending the reaction time only led to a minimal increase in yield (entry 2), while reducing the catalyst loading decreased conversion (entry 3 and 4), and increasing the concentration of the reaction mixture reduced the yield marginally (entry 5). A control experiment in neat DMSO confirmed the established critical role of water, which again in its absence led to formation of side products derived from cyclized aldehyde analogs reacting with the scavengers present in the cleavage mixture. Conversion of **2SM** remained high (83%), but the **2C** yield dropped to 58% (entry 6, UPLC-UV profile shown in the bottom chromatogram). An alternative hydroformylation ligand BIPHEPHOS was tested at 60 °C, 36 mol% catalyst loading and 60 h reaction time. High starting material conversion (96%) provided the product **[**^**13**^**C]2C** in high yield (61%), but alongside ca. 19% hydrogenated starting material (entry 7).

We were further interested in whether the COD-ligand of the pre-catalyst can act as a competitive substrate and thereby consume valuable syngas. When analyzing the resin-wash solutions after ^13^C-labeling hydroformylation experiments, no ^13^C-signals in the aldehyde region could be detected by ^13^C-NMR. However, in reactions without substrates other than the 1,5-COD of the pre-catalyst present, aldehyde signals were detected, alongside the isomerization product 1,3-COD (see Supporting Information, Section 2.8 for details). Since increased Rh-catalyst loadings (and thus increased COD ligands) overall improved peptide conversion substantially (Fig. 3a, entry 2–4), no considerable influence of COD on the peptide yield is expected. A completely olefin-free Rh-precatalyst would be optimal, but was not further envisioned for this project.

### Radiolabeling setup

Transferring the chemistry to ^14^C- and ^3^H-radiolabeling required a specialized apparatus (Fig. 3b). A custom two-chamber system with a side arm enabled connection to the tritium manifold to facilitate safe and controlled introduction of ^3^H_2_. For ^14^C-labeling applications, we employed ^14^COgen as a solid ^14^CO source with a palladium catalyst in chamber A, following Skrydstrup’s established methodology^45,55,56^. The custom two-chamber setup with COgen (using hydroformylation process setup HFP-2) on peptide **2SM** gave a comparable yield of the allysine **2A** (83%, entry 8, Fig. 3a), and increasing the reaction scale to 50 µmol for ^14^C-labeling allowed the use of only 2 equivalents of syngas instead of 4 equivalents, affording **[**^**14**^**C]2C** in a 33% yield (24% isolated, 13% RCY). Reaction conditions compared to ^14^C-labeling (Fig. 3b; hydroformylation process HFP-2) differed for ^3^H-labeling (Fig. 3b; hydroformylation process HFP-3) mainly in a smaller reaction scale (4.4–12 µmol) and the requirement for water-free solvent conditions to avoid isotope dilution. The low levels of 0.05 ^14^C/molecule are pre-determined by using a dilution of 95:5, ^12^CO:^14^CO in these experiments, due to economic and environmental reasons. However, for real biological applications, more ^14^CO could be used in relation to ^12^CO (or only using ^14^CO) to achieve higher specific activity of the product, which enables easier detection in biological studies.

### (Radio-)Isotope labeling of peptides

The optimal parameters for the hexapeptides on a 25 µmol reaction scale using stable isotopes were defined as 4 equivalents syngas and 72 mol% catalyst loading in DMSO:H_2_O (95:5, 2 mL) as reaction medium (Fig. 4). Following the three-step hydroformylation-reductive amination-deprotection/ cleavage sequence, we evaluated functional group tolerance and steric effects across diverse amino acids. The method demonstrated excellent compatibility with sterically demanding residues. **[**^**12**^**C** or ^**13**^**C]3C**, with histidine adjacent to the reaction centre, was obtained in high yields of 75–85%, despite the bulky trityl protecting group present during the main transformations. Notably, the optimized solvent system effectively addressed the undesired cyclization pathway. While we before showed that Ala as the neighboring amino acid promoted cyclization using neat DMSO as solvent at smaller reaction scale (see Fig. 2b, c), the use of DMSO:H_2_O (95:5) restored productivity to deliver **[**^**12**^**C]1C** in an 82% yield. The less bulky **[**^**12**^**C** or ^**13**^**C]6C** gave moderate yields of 48–55%, likely reflecting reduced steric shielding. The substrate with homo-allylglycine (**4SM**) delivered **[**^**12**^**C]4C** in a high yield of 92%. Furthermore, the reaction showed tolerance toward diverse functional groups. The conformationally constrained peptide containing proline (**7C**) and peptides with more complex amino acids such as arginine, methionine, and cysteine (**8C,** **9C**, and **10C**) were all compatible, affording yields between 48 and 72%. This comprehensive scope demonstrates broad functional group compatibility and indicates that several common protected amino acids do not compromise the hydroformylation performance.

**Fig. 5:**
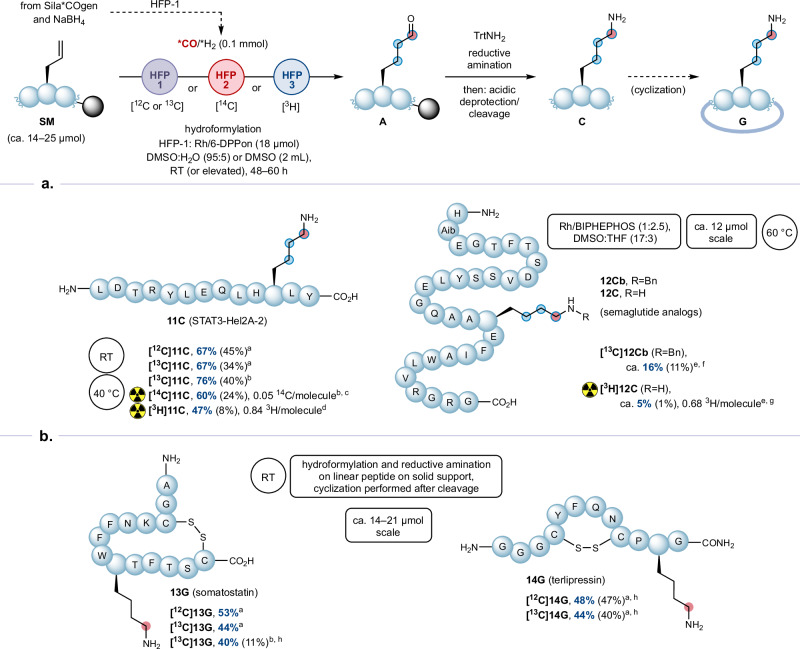
Application of the hydroformylation-reductive amination (-cyclization) workflow on pharmaceutically relevant peptides for ^13^C-, ^14^C-, and ^3^H-labeling. **a** Synthesis of isotope-labeled linear peptides STAT3-Hel2A-2 **11C** and semaglutide analogs **12Cb** and **12C**. **b** Application to the synthesis of the isotope-labeled cyclized peptides somatostatin **13G** and terlipressin **14G**. Reaction setup and conditions are dependent on the employed isotope (detailed in Figs. 2a and 3b, see Supporting Information for changes and detailed product isomer ratios): Hydroformylation process HFP-1 for stable isotopes [^12^C], [^13^C]; HFP-2 for [^14^C]; HFP-3 for [^3^H]; Reductive amination: tritylamine in DMSO, 1 or 10% AcOH (0.12 M), RT, 2 h, then NaCNBH_3_ in CH_2_Cl_2_:MeOH, 3:1 (0.15 M), RT, 1 h; Deprotection and cleavage: TFA:H_2_O:TiPS:DODT (92.5:2.5:2.5:2.5), RT, 3 h; for **13G** and **14G**: Cyclization: AcOH/H_2_O, (NH_4_)_2_CO_3_ (pH6), 10% DMSO, stirred open to atmosphere for 3–5 days. Yields determined by UPLC-UV purity analysis. Isolated yields are given in parentheses. ^a^21–25 µmol scale, 48 h, DMSO:H_2_O (95:5) as solvent in hydroformylation. ^b^20 µmol scale, 60 h, neat DMSO as solvent in hydroformylation. ^c^Using ^12^COgen:^14^COgen, 95:5. ^d^4.4 µmol scale, ^12^CO/^3^H_2_ (0.2 mmol), Rh/6-DPPon (7 µmol), 60 h, DMSO as solvent in hydroformylation. ^e^Ca. 12 µmol scale, ^12^CO or ^13^CO and ^1^H_2_ or ^3^H_2_ (0.2 mmol), 60 h, Rh/BIPHEPHOS 1:2.5 (14 µmol), DMSO:THF (17:3) as solvent in hydroformylation. ^f^Benzylamine in DMF used for reductive amination. ^g^Reductive amination was repeated three times, leading to partial decomposition. ^h^14–16 µmol scale.

We then investigated the platform using more complex and pharmaceutically relevant peptide sequences (see Supporting Information for detailed experimental procedures). First, the reaction sequence was applied to the synthesis of STAT3-Hel2A-2 **11C**, a peptide previously identified for potential breast cancer treatment (Fig. 5a)^57^. Depending on the isotope, different reaction setups were employed (HFP-1, HFP-2, or HFP-3) on a 4.4–25 µmol scale, at reaction times of 48–60 h, at RT or 40 °C. For ^13^C- and ^14^C-labeling on a 20–25 µmol scale, nearly stoichiometric Rh-catalyst and 4–5 equivalents of syngas were employed. Notably, the peptide was not prone to cyclization in neat DMSO and underwent complete reductive amination under these conditions, likely due to the neighboring steric environment with His(Trt). Using DMSO:H_2_O (95:5) in the hydroformylation step furnished **[**^**12**^**C** or ^**13**^**C]11C** in 67% yield, while neat DMSO at 40 °C rendered **[**^**13**^**C]11C** in a comparable yield. ^14^C-labeling afforded **[**^**14**^**C]11C** in 60% yield (24% isolated, 5% RCY), while a small-scale reaction (4.4 µmol) with tritium gas provided **[**^**3**^**H]11C** in 47% yield (8% isolated) with a tritium incorporation of 0.84 ^3^H/molecule. Variations in yield were attributed to the influence of different reaction setups, reaction scales, or challenging purifications. The hydroformylation platform offers the clear convenience in which a single precursor (**SM**) can be used for both ^14^C- and/or ^3^H-labeling because the workflow is flexible and can be tuned to the desired isotope. Synthesizing the precursor and optimizing the workflow once should reduce human resources, while late-stage labeling lowers the radiolabeling costs (current market price for 3.3 g Ba^14^CO_3_, 37 GBq, 2.15–2.20 GBq/mmol, ca. 50,000 euro).

Semaglutide analogs represent important targets for radiolabeling studies^4,13^. We synthesized **12 C** (Lys20-NH_2_) and **12Cb** (Lys20-benzyl), which lack the fatty di-acid chain at Lys20 present in native semaglutide (Fig. 5a). Hydroformylation of the solid-supported 31-mer required tailored conditions, including a reaction temperature of 60 °C for higher conversion, changing the ligand of the catalyst to BIPHEPHOS to improve the product isomer selectivity, and using DMSO:THF (17:3) to enhance the solubility of the new ligand (see Supporting Information, Section 2.5). Aqueous medium was not needed because the reductive amination for the semaglutide analogs was complete (no detectable cyclization occurring). Also, reductive amination with benzylamine did not yield any undesired dimerization on the 31-mer. The sequence provided ca. 16% of **[**^**13**^**C]12Cb** (Lys20-benzyl) when using benzylamine for reductive amination, and ca. 5% of **[**^**3**^**H]12C** (Lys20-NH_2_) (1% isolated) when using tritylamine for reductive amination with 0.68 ^3^H/molecule isotope incorporation. The lower yield when using tritylamine can be explained by tritylamine proving less efficient at reductive amination compared to benzylamine on the semaglutide analogs. Thus, three reductive amination cycles were required, leading to partial peptide decomposition and lower yield. This highlights sequence-specific limitations and a future opportunity to either optimize tritylamine conditions tailored to semaglutide analogs, add an additional benzyl deprotection step to the workflow when using benzylamine^58^ or to explore alternative protecting group strategies beyond the trityl group.

When extending the product scope to cyclic peptides containing disulfide bridges (Fig. 5b), we combined the standard workflow on linear resin-supported substrates with oxidative cyclization post-cleavage to access somatostatin **13G** and terlipressin **14G**. Somatostatin, an endogenous peptide hormone regulating secretion and proliferation^59^, contains two lysine residues that can be targeted by the hydroformylation approach. Our platform enables site-selective labeling by replacing a single lysine residue with allylglycine during SPPS. **[**^**12**^**C** or ^**13**^**C]13G** was obtained in 44–53% yield using DMSO:H_2_O (95:5), and in 40% yield when using neat DMSO in the hydroformylation reaction. Furthermore, terlipressin **[**^**12**^**C** or ^**13**^**C]14G**, a vasopressin analog effective in treating the hepatorenal syndrome and acute-on-chronic liver failure^60^, was also successfully synthesized in 44–48% yield. Collectively, these applications establish the method as a versatile platform for generating radiolabeled peptide tracers across diverse indication areas.

We have developed an on-resin peptide-compatible hydroformylation platform that addresses both critical bottlenecks in ^14^C- and ^3^H-radiolabeling of peptides. The method transforms allylglycine residues of complex peptides into allysine on solid support while incorporating isotopes from readily available ^13^CO or ^14^CO and ^3^H_2_, eventually affording ^13^C, ^14^C- or ^3^H-labeled lysine residues at a late stage without the need for derivatization. By systematically mitigating sequence-dependent allysine-backbone cyclization, the method delivers high yields with broad functional group tolerance. We implemented a simplified reaction setup using commercially available glassware for stable isotope applications and an adapted reactor design for radiolabeling. The workflow was validated by successful labeling of pharmaceutically relevant peptides, including STAT3-Hel2A-2, semaglutide analogs, somatostatin, and terlipressin, establishing utility across diverse therapeutic areas. The platform addresses a longstanding gap in ^14^C-labeling of peptides by providing robust and late-stage access to ^14^C-radiolabeled lysine residues. This preserves the native sequence, which is critical for preclinical and clinical studies. The approach has demonstrated isotope flexibility and pharmaceutical relevance, and we anticipate that the method will contribute to accelerating peptide drug discovery and drug development across the pharmaceutical industry.

## Methods

Detailed reaction optimization, experimental procedures, and changes in the procedure and reaction scale for individual peptides are provided in the Supporting Information.

### General procedure: test cleavage after hydroformylation and reductive amination

After hydroformylation and before proceeding with reductive amination a small aliquot of the resin was subjected to a cleavage reaction to estimate the conversion of the allylglycine-containing substrate **SM**. The aliquot after hydroformylation was added into a 1–2 mL peptide reactor. TFA:EDT:H_2_O (92.5:5:2.5, 330 µL) was added, the reaction mixture was shaken for 3 h and then filtered into a 1.5 mL Eppendorf vial. Diethyl ether (1 mL) was added for precipitation, the mixture was centrifuged, the supernatant discarded, and the peptide pellet washed with diethyl ether (3–5×) by centrifugation. UPLC-UV analysis of the test cleavage after hydroformylation typically provided a clean chromatogram composed of unreacted starting material **SM** and thioacetal product **B** (formed by reaction of EDT with the aldehyde or proposed cyclized analogs).

The test cleavage procedure was optionally performed after reductive amination (before the final cleavage) to assess whether the reductive amination reaction was successful. The presence of thioacetal **B** after the reductive amination test cleavage can indicate incomplete reductive amination, necessitating a second reductive amination cycle before final cleavage. The presence of **B** may also indicate the proposed formation of cyclized aldehyde analogs that are unreactive towards reductive amination. Depending on the peptide, this can be accompanied by the detection of reduced cyclization product **D** in the UPLC-UV chromatogram.

### General Procedure: hydroformylation for stable isotope-applications (^12^C or ^13^C and ^1^H)

The reaction was conducted in a commercially available two-chamber system (COware**®**) with H-caps. The hydroformylation chamber B was prepared with the resin-bound substrate **SM** (ca. 25 µmol, 1 equiv.). To the syngas-producing chamber A was added Sila^12^COgen or Sila^13^COgen (0.1 mmol, 4 equiv.) and granular NaBH_4_ (0.1 mmol, 4 equiv.). The two-chamber system was attached to the Schlenk-line via chamber A and the atmosphere was exchanged with nitrogen gas. A freshly prepared solution containing Rh(COD)_2_BF_4_ (18 µmol)/ 6-DPPon (90 µmol) in deoxygenated DMSO:H_2_O (95:5, 2 mL) was added to the substrate in the hydroformylation chamber B under a flow of nitrogen gas. Then, chamber B was closed with an H-cap and a red screw cap. The adapter connecting chamber A with the Schlenk-line was opened, and diglyme (1 mL) was added under a stream of nitrogen gas to the syngas-producing chamber A, which was then quickly closed with an H-cap and a red screw cap. The two-chamber reactor was shaken at room temperature for 48 h. Then, the reaction mixture was filtered over a peptide reactor and washed with 3× CH_2_Cl_2_ and 3× MeOH. A small aliquot of the resin was subjected to a test cleavage (procedure described above) and the remaining resin underwent the reductive amination reaction.

### General procedure: changes for ^14^C-hydroformylation reactions

The reaction was conducted in a custom-made two-chamber system (ca. 20 mL) with a side arm. The hydroformylation chamber B was prepared with the resin-bound substrate **SM** (ca. 20–50 µmol). To the CO-producing chamber A was added Pd(dba)_2_ (10 µmol) and a stirring bar. The two-chamber system was evacuated through the side arm. Then, a degassed solution of tri-*tert*-butylphosphine (10 µmol, 1 M in toluene) and DIPEA (0.3 mmol) in diglyme (0.5 mL) was added to chamber A, followed by a solution of ^12^COgen:^14^COgen (95:5, 0.1 mmol, 10.95 MBq) in diglyme (0.5 mL). Chamber A was stirred at 80 °C for 1 h. After allowing the reactor to cool down to room temperature, hydrogen gas (2.5 mL, 0.1 mmol) was added using a syringe. The system was allowed to equilibrate for 2–3 min. Then, a solution of Rh(COD)_2_BF_4_ (19 µmol)/ 6-DPPon (97 µmol) in DMSO or DMSO:H_2_O, 95:5 (2 mL) was added to chamber B. The two-chamber system was shaken at room temperature (or elevated temperature) for 60 h. After this time, the two-chamber was first carefully evacuated via the side-arm to remove radioactive gases, and then the reactor was opened. The reaction mixture was filtered over a peptide reactor and washed with 3× CH_2_Cl_2_ and 3× MeOH. A small aliquot of the resin was subjected to a test cleavage (procedure described above) and the remaining resin underwent the reductive amination reaction.

### General procedure: changes for ^3^H-hydroformylation reactions, exemplified for the synthesis of [^3^H]11 C

The reaction was conducted in a custom-made two-chamber system (ca. 20 mL) with a side arm. The hydroformylation chamber B was prepared with the resin-bound substrate **11SM** (ca. 4.4 µmol). To the CO-producing chamber A was added COgen (0.2 mmol), Pd(dba)_2_ (10 µmol), and a stirring bar. The two-chamber system was attached to the tritium manifold via the side arm, evacuated, and loaded with tritium gas (ca. 0.2 mmol). The valve connecting the reactor with the manifold was closed and the reactor was detached from the manifold. Then, a degassed mixture of DIPEA (0.3 mmol) and tri-*tert*-butylphosphine (10 µmol, 1 M in toluene) in diglyme (1 mL) was added to chamber A. Chamber A was stirred at 80 °C for 1 h to allow complete CO formation. After allowing the reactor to cool down to room temperature, a solution containing Rh(COD)_2_BF_4_ (7 µmol)/ 6-DPPon (36 µmol) in DMSO (1 mL) was added to the substrate in chamber B through the septum. The septa were parafilmed and the reactor was shaken at 40 °C for 60 h. After this time, MeOH (ca. 1 mL) was added to both chambers via the septa to allow hydrogen exchange of exchangeable tritium atoms. MeOH/MeOT was then carefully removed under a stream of nitrogen. This process was performed in total three times. Then, the reaction mixture was filtered over a peptide reactor and washed with 3× CH_2_Cl_2_ and 3× MeOH. A small aliquot of the resin was subjected to a test cleavage (procedure described above) and the remaining resin underwent the reductive amination reaction.

### General procedure for reductive amination and final deprotection and cleavage

A solution of tritylamine in DMSO, 10% acetic acid (5 mL, 0.12 M) was added to the resin, and the reaction mixture was shaken for 2 h. Then, a solution of NaCNBH_3_ in CH_2_Cl_2_:MeOH, 3:1 (5 mL, 0.15 M) was added to the reaction mixture and shaken for an additional 1 h. Thereafter, the reaction mixture was filtered over a peptide reactor and the resin was washed with 3× CH_2_Cl_2_ and 3× MeOH. Optionally, a small aliquot of the resin was subjected to a test cleavage (described above). For final cleavage and deprotection, the peptide on-resin was shaken in a peptide reactor with a solution of TFA:DODT:TiPS:H_2_O, 92.5:2.5:2.5:2.5 (2 mL) for 3 h, the solution was filtered and the resin washed with TFA. Then, diethyl ether was added to the filtrate for precipitation, followed by centrifugation. The supernatant was discarded, and the peptide pellet was washed with diethyl ether (4–5×) by centrifugation.

## Supplementary information

Supplementary Information

Transparent Peer Review file

## Data Availability

The UPLC-UV(-MS) profiles and NMR data generated in this study are provided in the Supporting Information. All data is available from the corresponding authors Chad Elmore and Anika Schick upon request.
