# Supplementary material for: Late-stage generation of 14C/3H-radiolabeled lysine residues via hydroformylation of peptides
Source: Nat Commun. 2026 Jun 12;17:5307. doi: 10.1038/s41467-026-74115-8 (PMC13272800; doi:10.1038/s41467-026-74115-8)
Supplement: Supplementary file 1 — Supplementary Information [file 41467_2026_74115_MOESM1_ESM.pdf]

Supplementary Information

Late-Stage Generation of $^{14}C/^{3}H$-Radiolabeled Lysine Residues via Hydroformylation of Peptides

Anika Schick[a,b]*, Marc San Jose Gracia[c], Hans Christian D. Hammershøj[b], Johan Broddefalk[d], Pablo Martínez-Pardo[a], Vitus J. Enemærke[b], Lena von Sydow[e], Anna Holub[c], Ranganath Gopalakrishnan[d], Kim S. Mühlfenzl[a], Troels Skrydstrup[b], Charles S. Elmore[f]*

[a]Early Chemical Development, Pharmaceutical Sciences, R&D, AstraZeneca, Gothenburg, Sweden; [b]Interdisciplinary Nanoscience Center (iNANO) and Department of Chemistry, Aarhus University, Denmark; [c]Sanofi-Aventis Deutschland GmbH, R&D, Integrated Drug Discovery, Industriepark Höchst, 65926 Frankfurt am Main, Germany; [d]Medicinal Chemistry, CVRM, Discovery Sciences, Biopharmaceuticals R&D, AstraZeneca, Gothenburg, Sweden; [e]Medicinal Chemistry, R&I, Discovery Sciences, BioPharmaceuticals R&D, AstraZeneca, Gothenburg, Sweden; [f]Early Chemical Development, Pharmaceutical Sciences, R&D, AstraZeneca, Boston, MA, USA.

**E-mail:* anika.schick@astrazeneca.com, chad.elmore@astrazeneca.com

Table of contents

Table of contents 2
1. General notes 5
1.1 General notes on reporting yield and isotope incorporation 5
1.2 Analytical instrumentation and purification instrumentation 7
1.3 General instrumentation for radiolabeling 8
1.4 General instrumentation for peptide chemistry 8
2. Optimization and analytical details 11
2.1 Optimization of the hydroformylation reaction on solid support 11
2.2 Analytical LC-MS workflow of peptides prone to cyclization 13
2.3 Challenge 1: Cyclization 15
2.4 Challenge 2: Dimerization 24
2.5 Optimization of hydroformylation process for semaglutide analogs 26
2.6 Analysis of cyclized products 28
2.7 Influence of aqueous conditions on isotope incorporation – of small molecule substrate 31
2.8 Investigation of COD-hydroformylation products 32
3. General Procedure for peptide syntheses 40
3.1 General Procedure GP1A: Manual peptide synthesis 40
3.2 General Procedure GP1B: Automated peptide synthesis 40
4. General Procedure for peptide cleavage and deprotection 41
4.1 GP2A: Hydroformylation-test cleavage of protected peptides from resin with a Wang-/Rink amide linker 41
4.2 GP2B: Cleavage of protected peptides from resin with a Wang-/Rink amide linker 41
4.3 GP2C: Cleavage of peptides from a resin with a 2-chlorotrityl-linker 41
5. General Procedure of hydroformylation reactions for stable isotope applications (^{12}C or ^{13}C and ^{1}H) 42
5.1 General Procedure GP3A: [^{12}C or ^{13}C, ^{1}H]Hydroformylation of resin-bound peptides 42
5.2 General Procedure GP3B: [^{12}C or ^{13}C, ^{1}H]Hydroformylation of resin-bound peptides 43
6. General Procedure of hydroformylation reactions for ^{14}C-, ^{2}H or ^{3}H-labeling 44
6.1 General Procedure GP4: [^{14}C]Hydroformylation with ^{14}COgen 44
6.2 General Procedure GP5: [^{2}H or ^{3}H]Hydroformylation with deuterium- or tritium-Gas 45
7. General Procedure GP6 for reductive aminations 47
7.1 GP6A: With trityl amine, 1% AcOH 47
7.2 GP6B: With trityl amine, 10% AcOH 47
7.3 GP6C: With benzylamine, 1% AcOH 47
8. Peptide (Allylglycine)-precursor syntheses 48
8.1 General Procedure GP7 for loading determination of the allylglycine-containing peptide on-resin 48
8.1.1 SI-1, SI-2, SI-3 48
8.1.2 SI-4 and SI-4-cleav 49

8.1.3 SI-5 and SI-6 *L*-allylglycine (Fmoc, dmb) ... 50
8.1.4 1SMa ... 51
8.1.5 1SMb ... 52
8.1.6 2SM ... 53
8.1.7 3SM ... 54
8.1.8 4SM ... 55
8.1.9 5SM ... 56
8.1.10 6SM ... 57
8.1.11 7SM ... 58
8.1.12 8SM ... 59
8.1.13 9SM ... 60
8.1.14 10SM ... 61
8.1.15 11SM ... 62
8.1.16 12SM ... 63
8.1.17 13SM ... 63
8.1.18 14SM ... 64
8.1.19 15SM ... 65
9. Hydroformylation syntheses and late-stage modifications ... 66
9.1 Hydroformylation of peptides ... 66
9.1.1 Tripeptides ... 66
9.1.2 Hexapeptide 1C ... 67
9.1.3 Hexapeptide 2C ... 68
9.1.4 Hexapeptide 3C ... 72
9.1.5 Hexapeptide 4C ... 74
9.1.6 Hexapeptide 6C ... 75
9.1.7 Hexapeptide 7C ... 77
9.1.8 Hexapeptide 8C ... 79
9.1.9 Hexapeptide 9C ... 81
9.1.10 Hexapeptide 10C ... 83
9.1.11 11C (STAT3-Hel2A-2) ... 85
9.1.12 12C and 12Cb (semaglutide analogs) ... 90
9.1.13 13G (somatostatin) ... 92
9.1.14 14G (terlipressin) ... 95
9.1.15 15B (BIO-11006-analog thioacetal) and 15A'a, 15A'b, and 15D (cyclized BIO-11006 analogs) 97
10. NMR spectra, UPLC-UV, and isotope patterns ... 100
10.1 NMR/LC-MS spectra of precursor peptides ... 101
10.1.1 SI-3 ... 101
10.1.2 SI-4 ... 103
10.1.3 SI-5 and SI-6 ... 104

10.1.4 1SMa and 1SMb 109
10.1.5 2SM 110
10.1.6 3SM 111
10.1.7 4SM 112
10.1.8 5SM (Precursor for 1C during optimization) 113
10.1.9 6SM 114
10.1.10 7SM 115
10.1.11 8SM 117
10.1.12 9SM 118
10.1.13 10SM 120
10.1.14 11SM 121
10.1.15 12SM 122
10.1.16 13SM 123
10.1.17 14SM 124
10.1.18 15SM 126
10.2 NMR/LC-MS spectra of final peptides 127
10.2.1 Tripeptides 127
10.2.2 Hexapeptide 1C 129
10.2.3 Hexapeptide 2C 131
10.2.4 Hexapeptide 3C 143
10.2.5 Hexapeptide 4C 147
10.2.6 Hexapeptide 6C 149
10.2.7 Hexapeptide 7C 154
10.2.8 Hexapeptide 8C 157
10.2.9 Hexapeptide 9C 162
10.2.10 Hexapeptide 10C 167
10.2.11 11C (STAT3-Hel2A-2) 170
10.2.12 12Ca and 12Cb (semaglutide analogs) 189
10.2.13 13G (somatostatin) 202
10.2.14 14D (terlipressin) 209
10.2.15 15B (BIO-11006 analog thioacetal) and 15A'a, 15A'b, 15D (cyclized BIO-11006 analogs) 215
11. Supplementary references 248

1. General notes

Nomenclature used for this project to name hydroformylation peptide intermediates, side products and products:
SM: Starting material (peptide with allylglycine)
A: Aldehyde
A': Hemiamidals (aldehyde analogs)
B: Thioacetal formed with EDT
C: Amine product (lysine) **Cb:** Benzylated lysine
D: Reduced cyclization product
E: Dimer enamine
E': Dimer reduced enamine
F: NMe_2 adduct
G: Disulfide bridged oxidative cyclization product

1.1 General notes on reporting yield and isotope incorporation

- <u>qNMR yield:</u> Analyzed using MestReNova. Quantification assumes identical dissolution of compounds and internal standards as well as full relaxation of the same NMR active nuclei in different chemical environments. Minor errors may arise if solvation differs (even when solution appears clear), if the relaxation differs (even though a longer relaxation time was allowed for qNMR experiments), or if signals overlap with each other.
- <u>UPLC-UV yields:</u> Typically analyzed using the ACD-Spectrus software. Starting material conversion, product and side product yields were estimated from UV-purity of the analyte in the crude mixture, a common approach in peptide chemistry. In the main manuscript, the isomers (likely linear and branched) were combined when reporting the UPLC-UV yields of **C**.
  Possible reasons for errors may include: UV-absorbance may differ between analyte and impurities; co-eluting impurities may be not identified in the analysis (in case of mass suppression effects); very polar or very apolar impurities may not elute under the chosen gradient and are thus not accounted for. Isomerized starting material (internal olefin) or hydrogenated starting material may co-elute with the starting material containing the allylglycine residue, which could not be accounted for when reporting starting material conversion/ remaining starting material in crude UPLC-UV profile using low-resolution mass spectrometry. Specific carryover was observed for the semaglutide precursor **12SM**, which retained on the LC-injector/column/etc. skewing the starting material/product ratios.
- <u>Isolated yields</u>: Reported for TFA salts of non-desalted products obtained typically after evaporation of TFA and MeCN/H_2O after purification by HPLC. The reported isolated yields are not corrected for UV-purity. We assumed for all peptides in the scope that only arginine, lysine, histidine and the free NH_2-*N*-terminus would be protonated by TFA with the respective counterion. The possibility for variable counterion content could not be accounted for in the yield. In some purified peptides, the PEG linker could not be fully removed by HPLC, which may lead to slightly increased mass and reported isolated yield. The isolated yields are based on the scale of the allylglycine containing peptide substrate, the loading of which on solid phase was calculated by qNMR integration of the allyl signals (unless otherwise stated); eventual overlapping signals of

impurities also containing the allyl signal, varying behaviour in the cleavage step, or different solubilization in the diethyl ether washing step cannot be accounted for, and might therefore lead to minor errors. The ”chemical” yield of radiolabeled compounds was calculated using the isotope incorporation (as determined by HRMS) in combination with the total radioactivity (as determined by scintillation counting) and the radiochemical purity, and not by mass.

- Linear:branched isomer ratios: During optimization, the products were not isolated and characterized; the linear isomer was assigned as the major signal detected in LC-MS (and for tripeptides in ^{1}H-NMR), whereas the minor signals likely correspond to isomeric branched products. Throughout a reaction sequence, there were slight differences in the isomer ratios when comparing intermediates and final products. This was not further investigated but could be a sign for co-eluting branched isomers or LC-MS artifacts and lead to minor inaccuracies in the reported isomer ratios (only reported in the Supporting Information).
- Resin-loading determination: The resin-loading was typically determined by qNMR analysis using the allylglycine olefin signals in ^{1}H-NMR of the peptide cleaved from an aliquot of the solid support, unless otherwise stated. In some cases, the loading by weight difference of the solid support before and after loading with the peptide was given in addition.
- Isotope incorporation: Determined using the excel sheet ”IsoPat[2]” with an algorithm developed by Kroutil and co-workers.[1] MassLynx V4.2 was used for analyzing the HRMS data. We attached the experimental isotope pattern of the (labeled) products and as a comparison the predicted isotope pattern of the non-labeled compound. For the ^{3}H-labeling experiments we expect an incorporation of less than the maximum of ca. two ^{3}H/molecule, due to isotope dilution. However, for $^{13/14}$C-labeling experiments full isotope incorporation is expected, i.e. 0.99 ^{13}C/molecule when starting with Sila^{13}COgen/^{13}COgen labeled with 0.99 ^{13}C/molecule; or an isotope incorporation of 0.05 ^{14}C/molecule when starting with a ^{12}COgen/^{14}COgen dilution of 95:5. In some instances, a slightly lower or higher incorporation was calculated, when comparing the isotope pattern of predicted non-labeled products vs. labeled experimental products. Also, occasionally minor negative values were calculated e.g. for a mass of +2 Da/ +3 Da (i.e. 2 ^{13}C-labels/3 ^{13}C-labels), where the isotope incorporation value should be zero. This may be explained by the uncertainty of the method, the prediction, or overlapping signals of side-products/ co-eluting impurities. Full carbon isotope incorporation is therefore still assumed for all carbon-labeling experiments using the hydroformylation approach, especially when using no dilution in CO-precursor for ^{13}C-labeling. In general, when working with compounds having a low specific activity, it is reported that the accuracy of HRMS-determined specific activity decreases.[2] Low specific activity was the case for this project in particular when working with a ^{12}COgen:^{14}COgen dilution of 95:5 to reduce radiochemical waste and costs. For practical and biological studies, no dilution would be used to increase the specific activity, but this was not appropriate for this study. Further, with larger biomolecules such as peptides, the error in calculated isotope incorporation is assumed to be high due to the more complex isotope pattern (as observed for example with the synthesized semaglutide analogs **12C** and **12Cb**).
- NMR: The NMR spectra are attached but not assigned and reported in detail due to the complexity of the large peptides. The signal for the ^{13}C-labels are given. Based on HPLC

and HMBC analyses we propose NMR-assignments for the ^{1}H-signals (or ^{2}H- and ^{3}H-labels) of the lysine residue's methylene group that bears the amine group (position **a**). The ^{1}H-signals for the other two positions (**b** and **c**) next to the amine-bearing methylene are given together (no individual proposed assignment).

1.2 Analytical instrumentation and purification instrumentation

All chemicals were purchased from commercial suppliers and were used without further purification. Some custom-made peptides were ordered by external suppliers on-resin, whose synthesis is not described. The solvents were degassed prior to hydroformylation reactions. The oxygen-sensitive hydroformylation reactions were carried out under nitrogen atmosphere (Schlenk technique), whereas the other reactions were carried out under ambient atmosphere. ^{14}COgen was prepared according to the procedure of Skrydstrup and co-workers.[3, 4]
^{1}H and ^{13}C **Nuclear Magnetic Resonance** (NMR) spectra were acquired on Bruker Avance (III) or Avance Neo spectrometers at a proton frequency of 500 or 600 MHz and referenced to DMSO-d$_6$ (2.50 ppm for ^{1}H and 39.52 ppm for ^{13}C). The NMR description is reported with coupling constants in Hertz, chemical shifts in ppm, integration, and multiplicities (as following: s = singlet, bs = broad singlet, d = doublet, t = triplet, q = quartet, m = multiplet, dd = doublet of doublets, etc.). In some cases, 2D NMR experiments (HSQC, HMBC, COSY, TOCSY, NHBMC, NHSQC, ROESY) were performed.

Analytical LC-MS data were typically acquired on two different Ultra-Performance Liquid Chromatography (UPLC) systems (Waters), coupled to a single quadrupole SQD2 System (Waters) equipped with an Acquity UPLC HSS T3 column (C18, 50 mm x 2.1 mm, 1.7 µm particles) (Waters), or an Acquity UPLC CSH C18 column (50 mm x 2.1 mm, 1.7 µm particles) (Waters), column temperature 60 °C, flow rate 0.5 mL/min. The gradients used varied depending on the polarity of the analyte in question, e.g. holding 3% B for 0.5 min, followed by a gradient of 3–43% B for 9.5 min, a steep rise in 0.1 min to 93% B, holding at 93% B for 0.8 min (different alternative gradients were e.g. 10–50%/20–60%/3–93%). Both mobile phases A (Milli-Q) and B (acetonitrile 95%), were buffered to pH3 with 10 mM formic acid and 1 mM ammonium formate. Ionization was achieved by pos-neg switching electrospray (ESI+/ESI-), scanning from 160–2000 m/z. The instruments give unit mass resolution. The TAC chromatograms reported are given over a wavelength range of 210–350 nm.

High resolution mass spectrometry (HRMS) was performed on an Ultra-Performance Liquid Chromatography (UPLC) system (Waters), coupled to a Synapt G2 Si Quadrupole Time-of-Flight (Q-ToF) system (Waters). The chromatographic separation was achieved using different 10-minute reversed phase focused gradients depending on the lipophilicity of the peptides, e.g. 10–60% B, in 10 minutes, 0.5 mL/min flow rate, using a Waters Acquity UPLC CSH column (C18, 100 mm x 2.1 mm, 1.7 µm particles), column temperature 50 °C, flow rate 0.5 mL/min. Both mobile phases A (Milli-Q) and B (acetonitrile 95%), were buffered to pH3 with 10 mM formic acid and 1 mM ammonium formate, plus an addition of 0.03% TFA. Ionization was achieved in positive electrospray mode (ESI+), scanning from 50–2000 m/z. The instrument was operated at a mass resolution of 20000 (FWHM), and mass accuracies were within 2 mDa. Peptide sequence information was acquired using alternating low- and high-energy fragmentation (MSE) in the trap cell of the Q-TOF.

Preparative reversed-phase high-performance liquid chromatography (HPLC) was conducted using a Waters 2545 Quaternary Gradient Module equipped with a Waters 2489 UV/Vis detector with an XBridge Prep C-18 (30 x 250 mm column, 10 µm OBD); mobile phases: A=0.1% TFA in H_2O; B=acetonitrile.

GC-MS analyses were performed on the GC-MS instrument Agilent Intuvo 9000 GC 5977C MS with split injection full scan method: split injection; 1 µL injection, split ratio 50:1; liner 250 °C; carrier gas He at 1.2 mL/min; column HP-5MS, 30 m x 250 µm x 0.25 µm; oven temp program: 40 °C (2.25 min); 40 °C/min to 250 (2.5 min); total run time 10 min; transfer line temperature: 250 °C; scan range: 50–550 m/z.

1.3 General instrumentation for radiolabeling

Liquid scintillation counting was performed using a Hidex 300 SL with Ultima Gold cocktail.

Radio-HPLC to determine radiochemical purity was performed on a Waters Acquity UPLC equipped with a Waters Xbridge C18 (4.6 x 100 mm, 3.5 µm) column, with mobile phases H_2O, 0.15% TFA and acetonitrile, with in-line radioactivity detection via a lab logic Beta Ram 5, and detection at 254 nm.

1.4 General instrumentation for peptide chemistry

Peptide synthesizer: For 0.3 mmol scale automated peptide syntheses, a Biotage® Initiator/Alstra^{TM} automated microwave peptide synthesizer was used.
For small scale manual peptide syntheses, filtrations, and washes of resins "reactors for peptide synthesis" with "luer stop caps for reactors" purchased from Carl Roth were used. They were used on an orbital shaker.

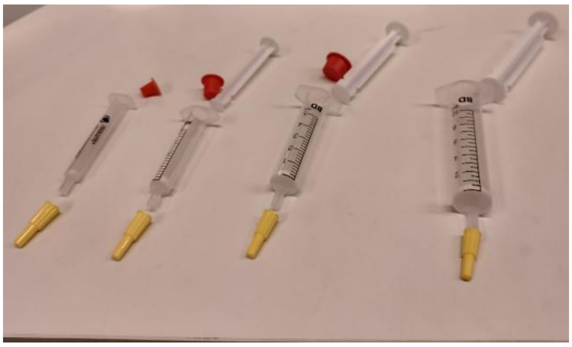

Figure S1. Peptide reactors for small scale manual peptide syntheses.

For large scale manual peptide syntheses (>0.3 mmol) a glass reactor with a frit was used that could be attached to the Schlenk line, in order to agitate the resin reaction mixture with a stream of nitrogen and to wash the resin with solvent under connected vacuum.

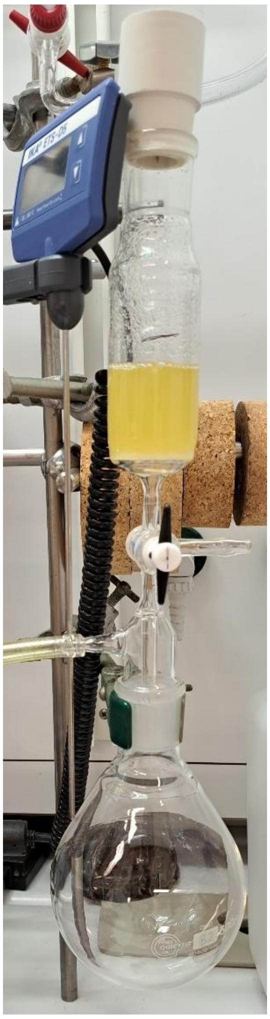

Figure S2. Peptide reactor for large scale manual synthesis.

Specialized equipment: For reactions operating at room temperature, an IKA/MTS 2/4 digital shaker was used. For reactions with peptide resins that had to be shaken and heated, an Eppendorf® ThermoMixer® C was used with an Eppendorf™ ThermoMixer™ C Accessory adapter and a Smartblock™ Thermoblock to allow the connection of the metallic heating block.

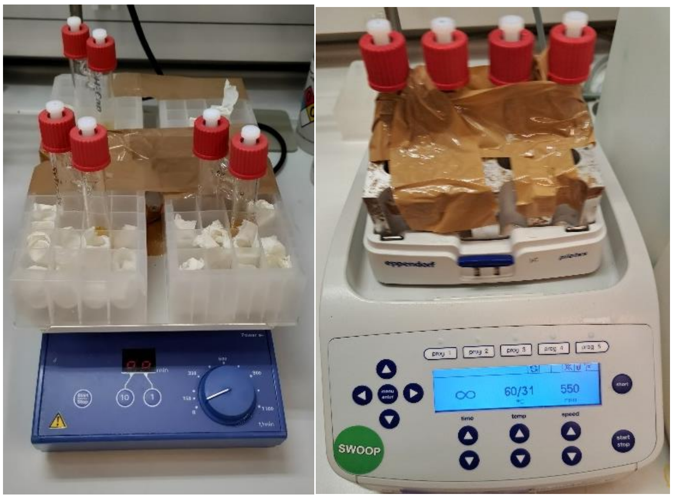

Figure S3. Shakers. ***Left:*** IKA/MTS 2/4 digital shaker operating at room temperature.
Right: Eppendorf® ThermoMixer® C operating at elevated temperatures.

For conventional reactions, the commercially available 20 mL COware® reactor with H-caps and red screw caps purchased from Merck or SyTracks was used (Figure S4, left).
<u>Warning for glassware under pressure</u>: The reactions are performed under pressure and must be conducted in a fume hood behind a protective shield. COware® may not be operated above 5 bar.

For radiolabeling reactions, specialized glassware was purchased from RC Tritec (Figure S4, right). The reactor could be attached to the deuterium/tritium manifold *via* the side arm and detached by closing the valve to allow shaking. The two chambers were closed by H-caps and red screw caps. The two small openings were closed by a tight septum and a beige screw cap, which allowed addition of reagents or solvents.

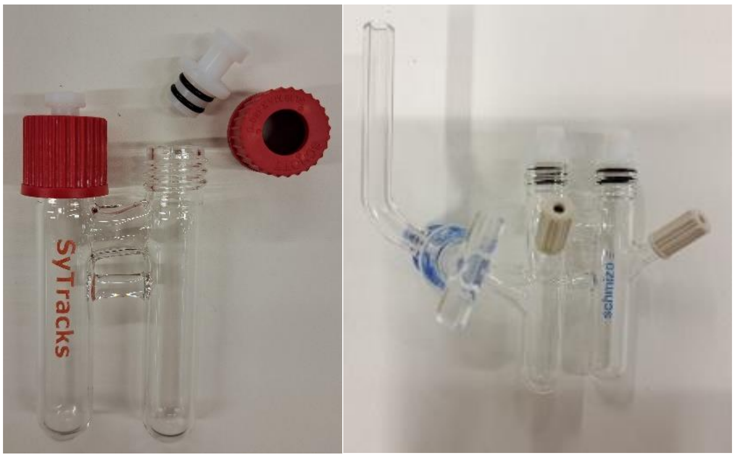

Figure S4. ***Left:*** Commercial two-chamber system with H-caps and red screw caps.
Right: Custom-made two-chamber system with side-arm, valve, as well as small openings with septa for addition of reagents/solvents, H-caps (and red screw caps, not shown in the picture).

2. Optimization and analytical details

2.1 Optimization of the hydroformylation reaction on solid support

Resin optimization

For peptide chemistry, it is often advantageous to conduct reactions with the peptide substrate anchored via its *C*-terminus to a resin such as polystyrene or polystyrene-PEG (TentaGel®). Solid-phase synthesis simplifies work-up/ purification and handling, and it facilitates access to fully protected peptides. For peptide products containing a carboxylic acid *C*-terminus, the peptide can be attached to the resin using for example a Wang-linker or chlorotrityl-linker. The Wang-linker is highly stable and typically requires harsh acidic conditions (about 95% TFA) for cleavage. In contrast, only mild conditions are needed for cleaving peptides with the chlorotrityl-linker (e.g. TFE:CH_2Cl_2, 2:8) which was ideal for preserving reactive aldehyde functionalities during cleavage. We first evaluated how the solid support/ *C*-terminal functionality influences the reaction performance, and the results are shown in Table S1. Although conversion on resin was lower than in solution, the results demonstrate that hydroformylation is feasible on solid support. Notably, the sum of product yield and remaining starting material did not reach 100% in qNMR quantification using an internal standard. This discrepancy is also confirmed by comparison of the sum of product (aldehyde signal in ^{1}H-NMR) and starting material (allyl signal in ^{1}H-NMR) to the higher peak integration of Valin signals in the ^{1}H-NMR spectrum, which can serve as an internal standard within the peptide. The observed discrepancies by qNMR suggest formation of side products.

Table S1. Comparison of hydroformylation using three different tripeptides; **SI-3** bearing a CO_2*t*Bu terminus, **SI-4cleav** bearing a free CO_2H terminus, and **SI-4** being linked to a polystyrene resin via a chlorotrityl linker.

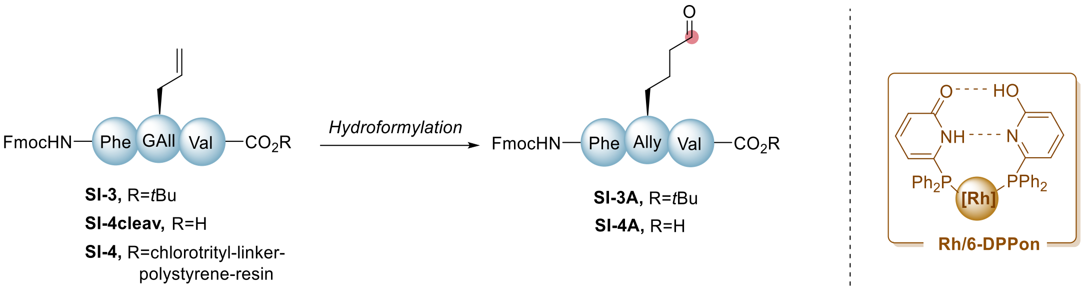

SI-3, R=*t*Bu
SI-4cleav, R=H
SI-4, R=chlorotrityl-linker-polystyrene-resin

SI-3A, R=*t*Bu
SI-4A, R=H

Entry	Tripeptide	Conversion [%][a]	Yield (l:b) [%][a]
1	**SI-3**	90	86 (70:16)[c]
2	**SI-4cleav**	96	49 (40:9)
3[b]	**SI-4**	29	10 (9:1)

Hydroformylation: Chamber A – SilaCOgen (11 equiv.), granular $NaBH_4$ (11 equiv.), THF (1 mL). Chamber B – substrate (ca. 15 µmol), $Rh(COD)_2BF_4$ (3.5 mol%), 6-DPPon (17.3 mol%), THF (156 µL), 30 °C, 16 h. [a]Analysis by qNMR using 1,3,5-trimethoxybenzene as internal standard, branched and likely diastereomeric aldehydes were summarized to one value, and determined by integration of the minor aldehyde signals. [b]Chamber B – THF (0.5 mL), RT, shaking. *Cleavage:* TFE:CH_2Cl_2 (2:8). [c]Corrected qNMR yield using *t*Bu-signal as internal standard within the peptide.

Solvent optimization

Optimization of the hydroformylation of tripeptide **SI-4** is summarized in Table S2. Among the preliminary solvents evaluated, DMF afforded highest performance. During solvent optimization, we noted partial transfer of THF from the syngas-generating chamber (chamber A) into the hydroformylation chamber (chamber B), attributed to THF's low boiling point and volatility. To suppress cross-chamber contamination, THF was replaced with diglyme in subsequent experiments, a solvent with high boiling point but also equally compatible for syngas generation.

Table S2. Optimization of the hydroformylation of tripeptide **SI-4** on-resin.

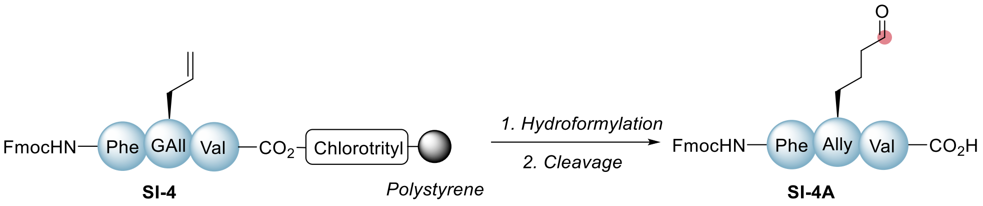

Entry	Solvent [mL]	$Rh(COD)_2BF_4$/ 6-DPPon (1:5) [mol%]	Conversion [%][a]	Yield (l:b) [%][a]
1	THF (0.5)	3.5	29	10 (9:1)
2	THF (0.5)	10	40	36 (29:7)
3	THF (0.5)	30	38	27 (23:4)
4	THF (1.0)	10	21	13 (11:2)
5	DMF (1.0)	10	43[b]	26 (22:4)
6	NMP (1.0)	10	27	18 (15:3)
7	Dioxane (1.0)	10	18	8 (7:1)
8	DMA (1.0)	10	12	4 (3:1)
9	Anisole (1.0)	10	12	3
10	CH_2Cl_2 (1.0)	10	11	5 (4:1)
11	Toluene (1.0)	10	1	1

Hydroformylation: Chamber A – SilaCOgen (11 equiv.), granular $NaBH_4$ (11 equiv.), THF (1 mL). Chamber B – **SI-4** (ca. 15 µmol), $Rh(COD)_2BF_4$ (3.5-30 mol%), 6-DPPon (17.3-150 mol%), solvent (0.5 or 1 mL), RT, 16 h, shaking. *Cleavage:* TFE:CH_2Cl_2 (2:8). [a]Analysis by qNMR using 1,3,5-trimethoxybenzene as internal standard, branched diastereomeric aldehydes were summarized to one value. [b]By LC-UV-MS, the ratio of (**SI-4A**):(**SI-4**) was 32:68. Under the **SI-4A** signal, a co-eluting compound with the mass of the product minus water was observed, a potential indication for side-product formation; similar observations for other entries.

We then examined the optimized hydroformylation conditions using DMF on hexapeptide **1SMa** tethered to a TentaGel® resin via a Wang-linker (Table S3). In this series, the yield of aldehyde **1A** was estimated by the LC-UV-MS purity of thioacetal **1B**, formed upon reaction of **1A** with the scavenger 1,2-ethanedithiol (EDT) under the strongly acidic cleavage conditions. The observed formation of **1F** by MS is plausibly due to DMF decomposition to dimethylamine under the reaction conditions. With dimethylamine formed, the aldehyde **1A** is thought to undergo catalyst-mediated reductive amination, yielding the observed dimethyl amine adduct **1F** (other mechanisms might also be possible). DMSO allows comparable conversion but without formation of **1F**, identifying DMSO as the preferred solvent for hydroformylation of peptides.

Table S3. DMF vs. DMSO and comparison of the nature of the resin.

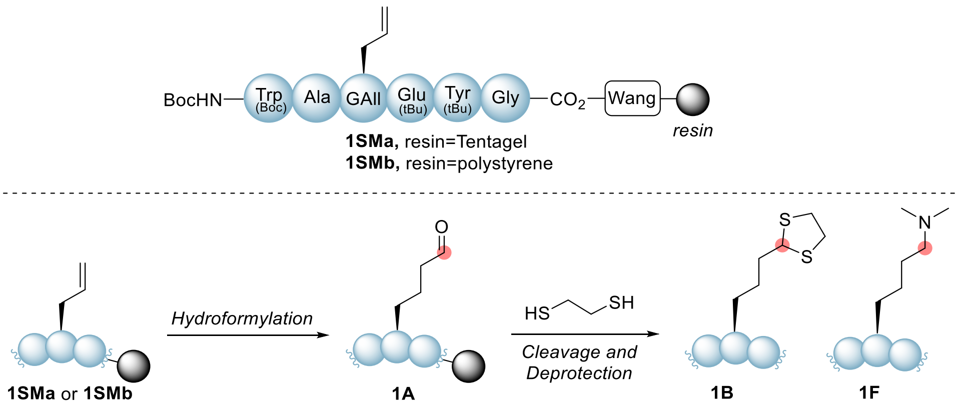

Entry	SM	Solvent	Temp.	Conversion [%][a]	Yield of 1B (l:b) [%][a]	Yield of 1F (l:b) [%][a]
1[b]	**1SMa**	DMF	RT	22	14 (11:3)	9 (7:2)
2	**1SMa**	DMF	RT	100	67 (52:15)	27 (19:8)
3	**1SMa**	DMF	60 °C	100	25 (20:5)	76 (50:26)
4	**1SMa**	DMSO	RT	92	92 (76:16)	0
5[c]	**1SMb**	DMSO	RT	85	83 (68:15)	0

Hydroformylation: Chamber A – SilaCOgen (40 equiv.), granular $NaBH_4$ (40 equiv.), diglyme (1 mL). Chamber B – **1SMa** (ca. 5-6 µmol) (or **1SMb**), $Rh(COD)_2BF_4$ (1.4 equiv.), 6-DPPon (7 equiv.), solvent (1 mL), 60 h, shaking. *Cleavage:* TFA:EDT:H_2O (92.5:5:2.5), 3 h, RT. [a]Analysis by LC-UV-MS via comparing the peak integration. [b]Chamber A – SilaCOgen (11 equiv.), granular $NaBH_4$ (11 equiv.), diglyme (1 mL). Chamber B – **1SMa** (ca. 15-17 µmol), $Rh(COD)_2BF_4$ (10 mol%), 6-DPPon (50 mol%), DMF (1 mL), RT, 16 h, shaking, conditions comparable as for tripeptide **SI-4** (Table S2, entry 5). [c]ca. 1.5x higher resin loading, i.e. higher scale and fewer equivalents of reagents were used.

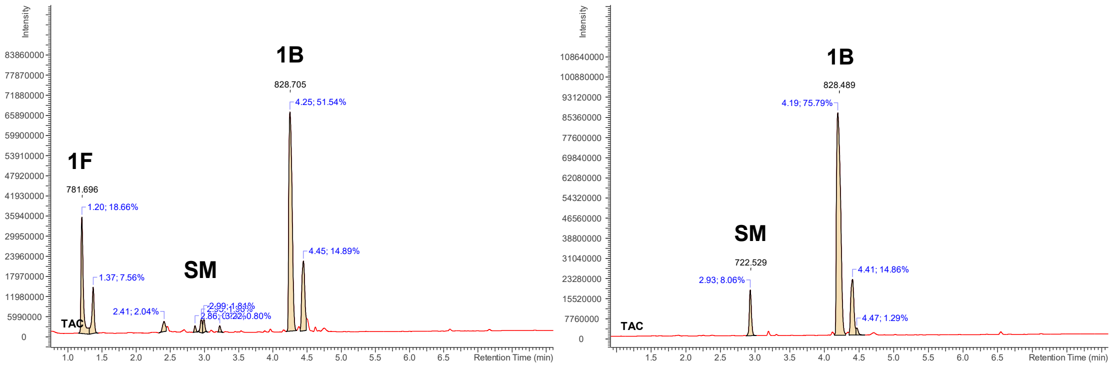

Figure S5. LC-MS spectra (low resolution) of crude reaction mixtures after hydroformylation. ***Left:*** Table S3, entry 2, using DMF. ***Right:*** Table S3, entry 4, using DMSO.

2.2 Analytical LC-MS workflow of peptides prone to cyclization

We investigated hydroformylation of a subset of peptides that proved unexpectedly challenging. Despite modest size and functional complexity, these substrates exhibited competing pathways including intramolecular cyclization and dimerization. Here, we examine these challenges and outline strategies to understand and mitigate those in related peptides.

Optimization was centered on two model substrates: the 10-mer **15SM** (a precursor to the pharmaceutically interesting peptide BIO-11006[5]) and hexapeptide **1SMa**, both identified as prone to cyclization (Figure S6).

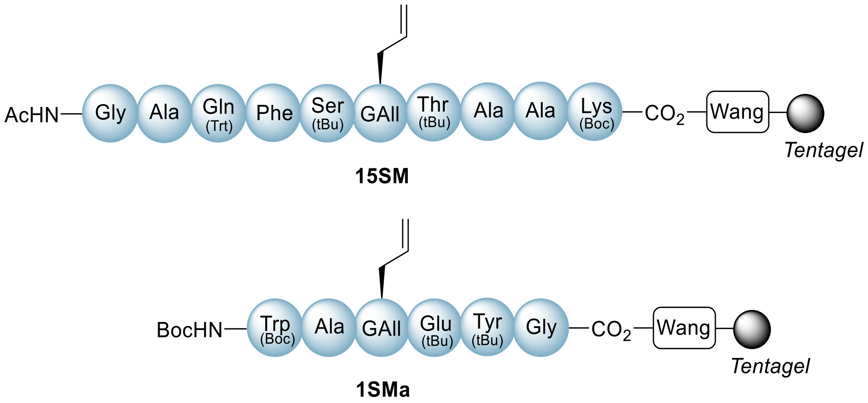

Figure S6. 10-mer **15SM** and hexapeptide **1SMa** being prone to intramolecular cyclization and serving as model substrates during optimization.

The analytical workflow employed to monitor reaction progress and (side-) product profiles isoutlined in Scheme S1. The sequence comprises on-resin hydroformylation of **SM** to generate aldehyde **A** (and analogs), followed by on-resin reductive amination using benzylamine to form amine **Cb**, and final acidic general deprotection and cleavage of the products (and side products).

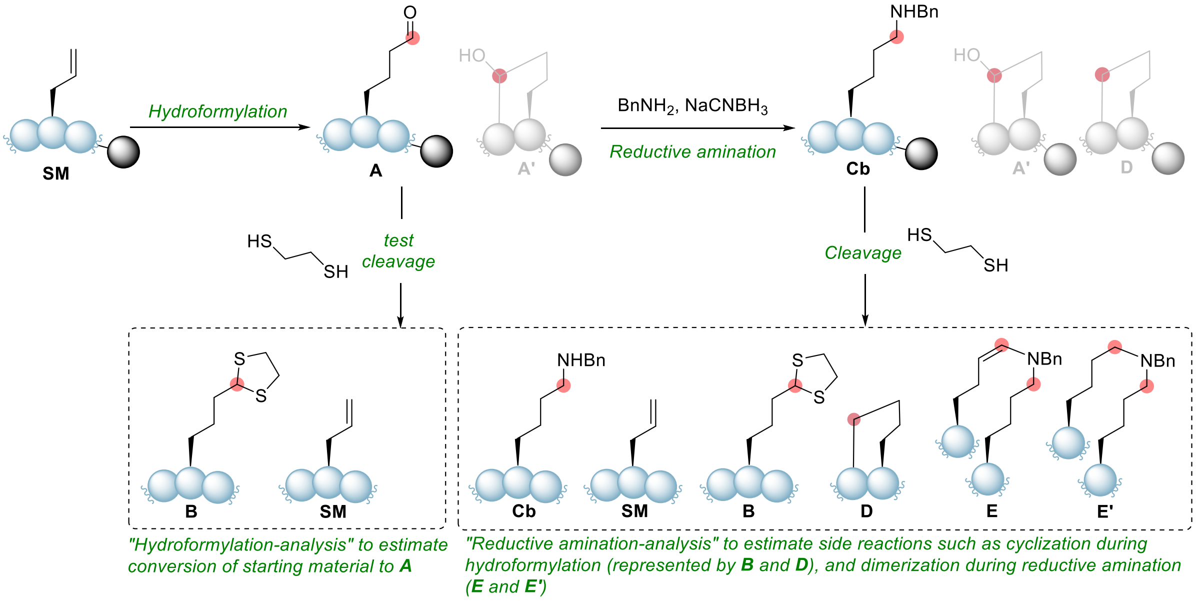

Scheme S1. General (analytical) workflow to determine side reactions, including test cleavages to perform “hydroformylation analysis” and “reductive amination analysis”. Typical (side-)products for these peptides are shown. *Hydroformylation:* Chamber A – SilaCOgen, granular $NaBH_4$, diglyme. Chamber B – substrate **SM**, $Rh(COD)_2BF_4$:6-DPPon (1:5), DMSO, RT, 60 h, shaking. *Reductive amination:* Benzylamine in CH_2Cl_2, 1% AcOH (0.12 M), RT, 2 h, $NaCNBH_3$ in CH_2Cl_2:MeOH, 3:1 (0.15 M), RT, 1 h, shaking. *Cleavage/Deprotection:* TFA:EDT:H_2O (92.5:5:2.5), 3 h, RT.

Hydroformylation analysis: The general starting material conversion after hydroformylation was assessed by cleaving a small test aliquot of resin **A** using TFA:EDT:H_2O (92.5:5:2.5), in which EDT serves as the thiol scavenger. EDT reacts with aldehyde **A** (and related species such as cyclized hemiamidal **A’**) to afford thioacetal **B**. LC-MS of the crude after hydroformylation generally displayed only two peptide signals: starting material **SM** and product thioacetal **B,** which allows estimation of starting material conversion. Importantly, this cleavage mixture proved to be superior to the typically employed mixture TFA:DODT:TiPS:H_2O (92.5:2.5:2.5:2.5) (DODT=3,6-Dioxa-1,8-octanedithiol, TiPS=triisopropylsilane) for analysis of conversion and cyclization. TFA:EDT:H_2O (92.5:5:2.5) typically yielded the single product

B. In contrast, TFA:DODT:TiPS:H_2O led to a mixture of different cyclized peptides (signals corresponding to the mass of enamides detected in LC-MS), reduced cyclized peptides due to the presence of a silane, and different thioacetals formed with DODT (either cyclized or double-addition of DODT to the aldehyde), which are not shown; using this typically employed cleavage mixture would have therefore complicated the analysis.

Reductive amination analysis: (An aliquot of) peptide **A** on-resin was subjected to reductive amination with benzylamine to form the benzyl-substituted lysine product **Cb**. Peptide **Cb** was then cleaved from the resin using TFA:EDT:H_2O (92.5:5:2.5) and analyzed by LC-MS. In the ideal case without side reactions, only product **Cb** and residual **SM** would be observed. However, additional peptide signals appeared for the "challenging" peptides prone to side reactions, that we hypothesize to be formed the following way:

> *Thioacetal* **B**: Results either from unreacted aldehyde **A** in case of incomplete reductive amination, or can indicate presence of cyclic hemiamidal **A'** formed under hydroformylation conditions. **A'** is thought to not react under reductive amination conditions but instead be able to react with EDT during cleavage, revealing **B**.
>
> *Cyclization product* **D**: Results from formation of hemiamidal **A',** which is reduced during reductive amination to yield the reduced cyclization product.
>
> *Dimer* **E**: Results from intermolecular coupling (hemiaminal formation) between the secondary amine peptide **Cb** with aldehyde **A** on-resin. The structure of **E** is proposed based on MS data but was not isolated and characterized.
>
> *Dimer* **E'**: Results from reduction of **E** during reductive amination. The structure of **E'** is proposed based on mass data but was not isolated and characterized.

2.3 Challenge 1: Cyclization

With the preliminary optimized conditions, substrates **15SM** and **1SMa** resulted in high conversion during hydroformylation, reaching 70% and 92%, respectively (Table S4, entries 1 and 2). However, the following reductive amination was inefficient: only modest yields of the desired amine product **Cb** were obtained, namely 21% of **15Cb** (entry 1) and 12% of **1Cb** (entry 2). For substrate **1SMa**, trace dimerization products **1E/1E'** (3%) were detected (entry 2).

Instead of reductive amination, a substantial fraction of the peptide appeared as thioacetal **B** after cleavage with TFA:EDT:H_2O, possibly indicating insufficient reductive amination of the aldehyde on the solid support prior to cleavage. By LC-MS, 40% of **15B** (entry 1) and 64% of **1B** (entry 2) were present in the crude profiles. For both peptides, minor amounts of reduced cyclized peptide **D** were observed. These findings indicate that a significant portion of the aldehyde was rendered unreactive toward reductive amination, indicating intramolecular cyclization to hemiamidal **A'** that resists imine formation yet can undergo (to a minor extent) reduction under reductive amination conditions to form **D**. This could explain the observation of minor amounts of **D** with the residual cyclized peptide being captured by EDT during cleavage to give mainly **B**.

An initial working hypothesis was that reductive amination parameters required adjustments to fully convert the aldehydes (and analogs). Different variations (not shown), failed to improve conversion to the desired product **Cb**.

Table S4. Challenging substrates **15SM** and **1SMa** that lead to major presence of **B** after reductive amination.

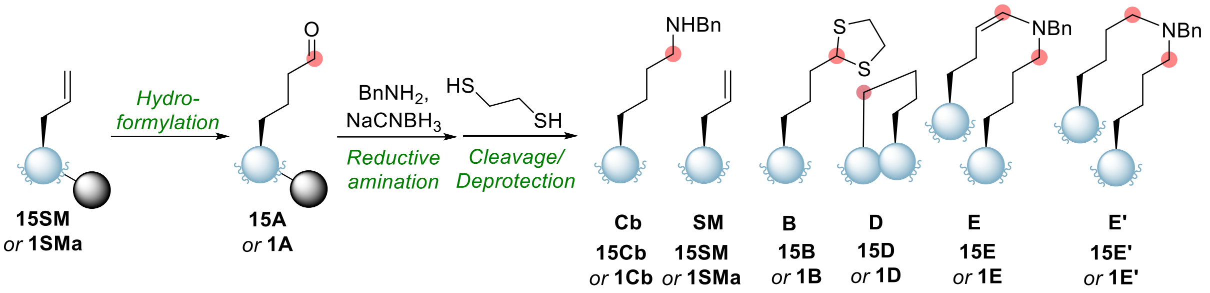

Entry	SM	Conversion to A, l:b [%][a,b]	Conversion of SM [%][a,b]	Yield of Cb, (l:b) [%][a]	Yield of B (l:b) [%][a]	Yield of D (l:b) [%][a]
1	**15SM**	70 (65:5)	71	21 (19:2)	40 (39:1)	11
2[c]	**1SMa**	92 (76:16)	92	12 (9:3)[c]	64 (61:3)	3

Hydroformylation: Chamber A – SilaCOgen (0.2 mmol), granular $NaBH_4$ 0.2 mmol), diglyme (1 mL). Chamber B – **15SM/ 1SMa** (ca. 5-6 μmol, 34 mg), Rh/6-DPPon (1:5, 6.9 μmol), DMSO (1 mL), RT, 60 h, shaking. *Reductive amination:* aldehyde **A** (small test amount of resin), benzylamine in CH_2Cl_2, 1% AcOH (0.12 M, 0.5 mL), RT, 2 h, $NaCNBH_3$ in CH_2Cl_2:MeOH, 3:1 (0.15 M, 0.5 mL), RT, 1 h, shaking. *Cleavage/Deprotection:* TFA:EDT:H_2O (92.5:5:2.5), 3 h, RT. [a]Yield estimated by LC-UV-MS after cleavage with TFA:EDT:H_2O (92.5:5:2.5) via comparing the peak integration. [b]Conversion to aldehyde **A** (and cyclized analogs) and conversion of **SM** estimated by cleaving a test portion of the resin after hydroformylation with TFA:EDT:H_2O (92.5:5:2.5) and analyzing the purity of formed thioacetal **B** and remaining **SM**. [c]3% of dimers **1E**/ **1E'** detected after reductive amination.

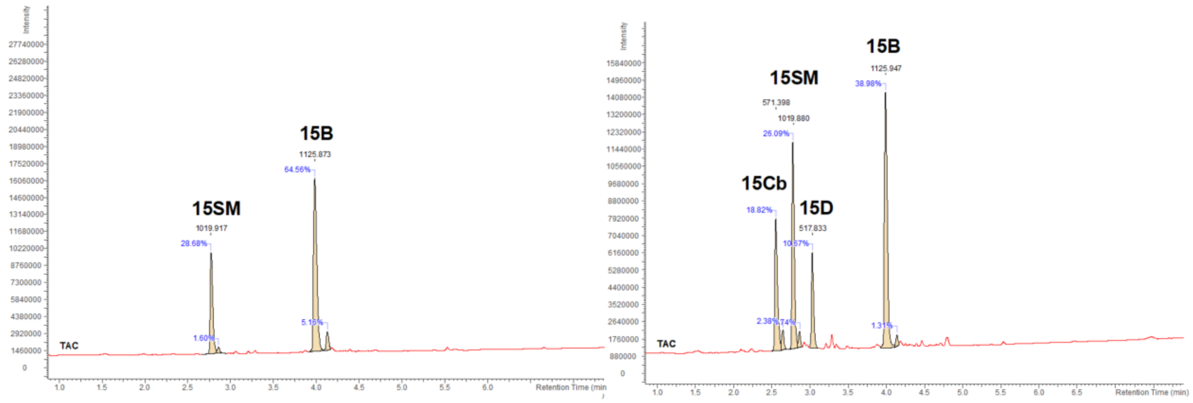

Figure S7. LC-MS spectra (low resolution) of crude reaction mixtures with **15SM**. ***Left:*** Table S4, entry 1, after hydroformylation. ***Right:*** Table S4, entry 1, after reductive amination.

Guided by the observation of intramolecular cyclization occurring (presence of **D**), we refocused optimization on conditions that disfavor cyclization during hydroformylation. The variables encompassed are: (i) the structural features proximal to the reaction center, (ii) reaction time and temperature, (iii) catalyst/ ligand selection, and (iv) solvent effects. Across all studies, a critical readout to determine the degree of cyclization was persistence of thioacetal **B** and/ or cyclization product **D** after reductive amination.

(i) Dependence on structural features

We first examined how structural features proximal to the reaction center allylglycine influence cyclization (Table S5).

Table S5. Comparison of structural features that influence cyclization.

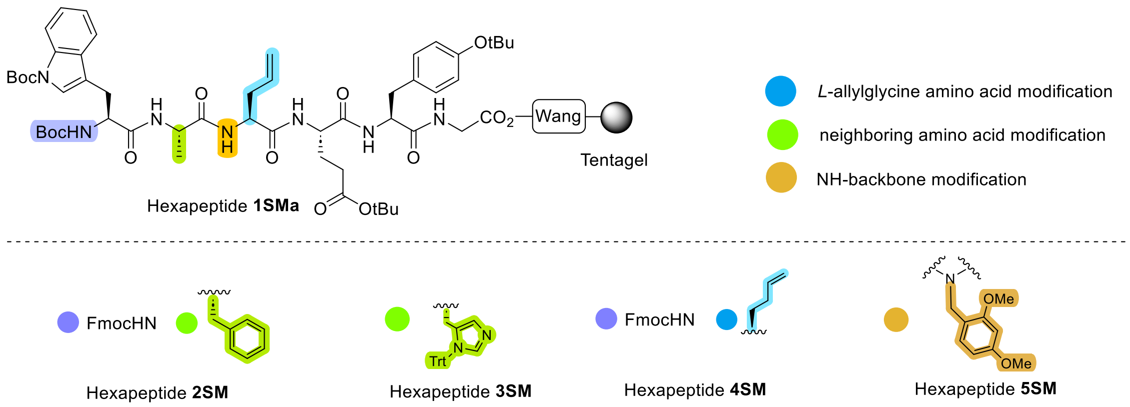

Entry	SM	Conversion to A (l:b) [%][a,b]	Conversion of SM [%][a,b]	Yield of Cb (l:b) [%][a]	Yield of E and E' [%][a,c]	Yield of B (l:b) [%][a]	Yield of D (l:b) [%][a]
1	**1SMa**	92 (76:16)	92	12 (9:3)	3	64 (61:3)	3
2	**2SM**	75 (70:5)	75	13 (8:5)	16	43	0
3	**3SM**	87 (62:25)	95	41 (20:21)[d]	37	0	0
4	**4SM**	89	89	14 (13:1)	66	0	0
5[e]	**5SM**	60[e]	85[e]	30[e]	0	0	0

Hydroformylation: Chamber A – SilaCOgen (0.2 mmol), granular $NaBH_4$ (0.2 mmol), diglyme (1 mL). Chamber B – substrate **SM** (ca. 4-6 µmol, 34 mg, 0.11-0.17 mmol/g), Rh/6-DPPon (1:5, 6.9 µmol), DMSO (1 mL), RT, 60 h, shaking. Note: ca. 5 µmol substrate used but (slight) differences in loading lead to varying differences in reaction scale and reagent equivalents. *Reductive amination:* aldehyde **A** (small test amount of resin), benzylamine in CH_2Cl_2, 1% AcOH (0.12 M, 0.5 mL), RT, 2 h, $NaCNBH_3$ in CH_2Cl_2:MeOH, 3:1 (0.15 M, 0.5 mL), RT, 1 h, shaking. *Cleavage/Deprotection:* TFA:EDT:H_2O (92.5:5:2.5), 3 h, RT. [a]Yield estimated by LC-UV-MS after cleavage with TFA:EDT:H_2O (92.5:5:2.5) via comparing the peak integration. [b]Conversion to aldehyde **A** (and cyclized analogs) and conversion of **SM** estimated by cleaving a test portion of the resin after hydroformylation with TFA:EDT:H_2O (92.5:5:2.5) and analyzing the purity of formed thioacetal **B** and remaining **SM**. [c]Signals of **E** and **E'** were combined for an overall yield. [d]The ratio of isomers of **A** and isomers of **Cb** was not further investigated, nor were the isomers isolated and identified for either of the screening reactions. [e]Impure starting material on-resin (conversion cannot be assessed reliably), lower resin loading.

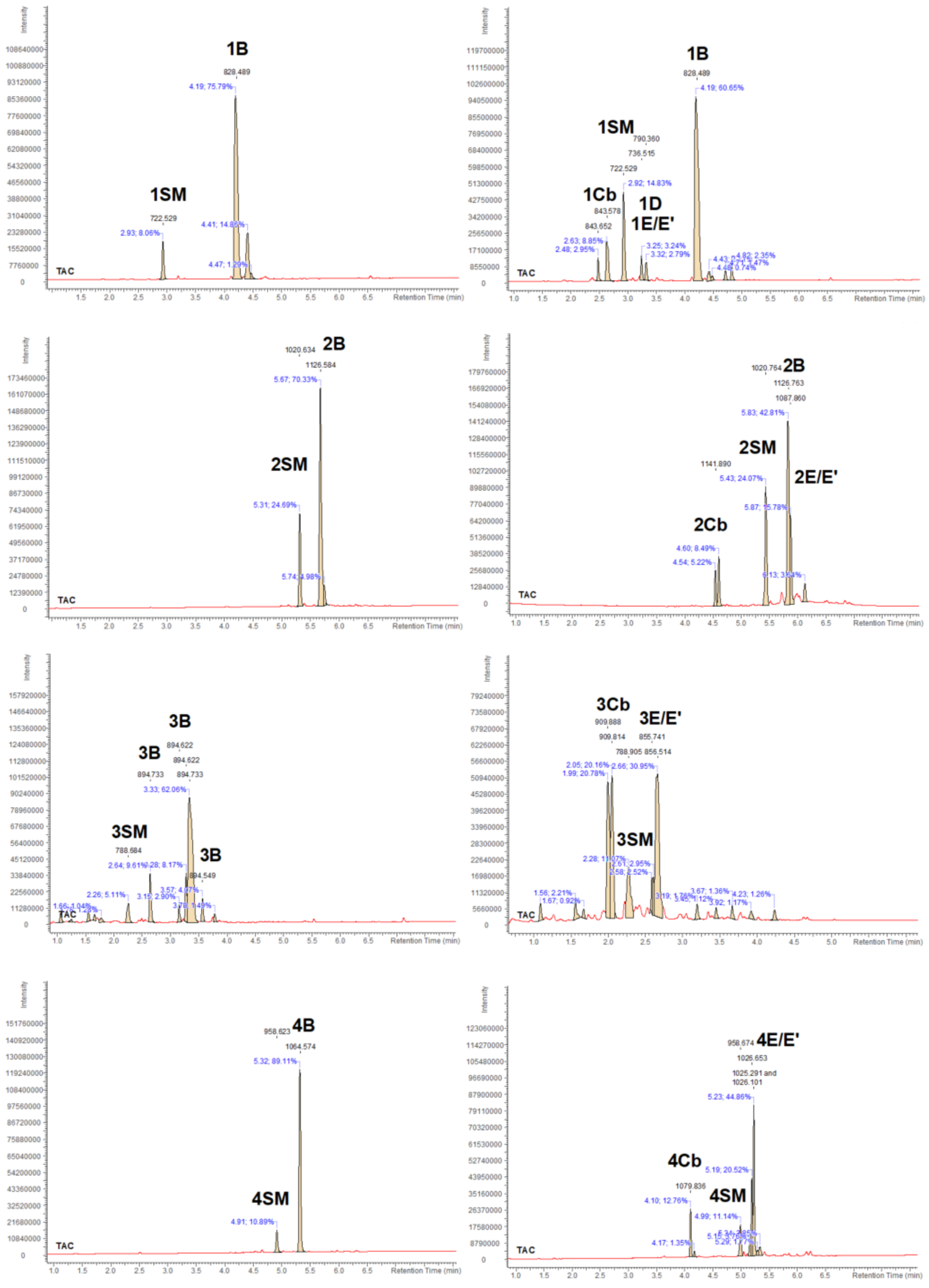
1SM
1B
1Cb
1D
1E/E'
2B
2SM
2Cb
2E/E'
3B
3SM
3Cb
3E/E'
4B
4SM
4Cb
4E/E'
TAC
Intensity
Retention Time (min)

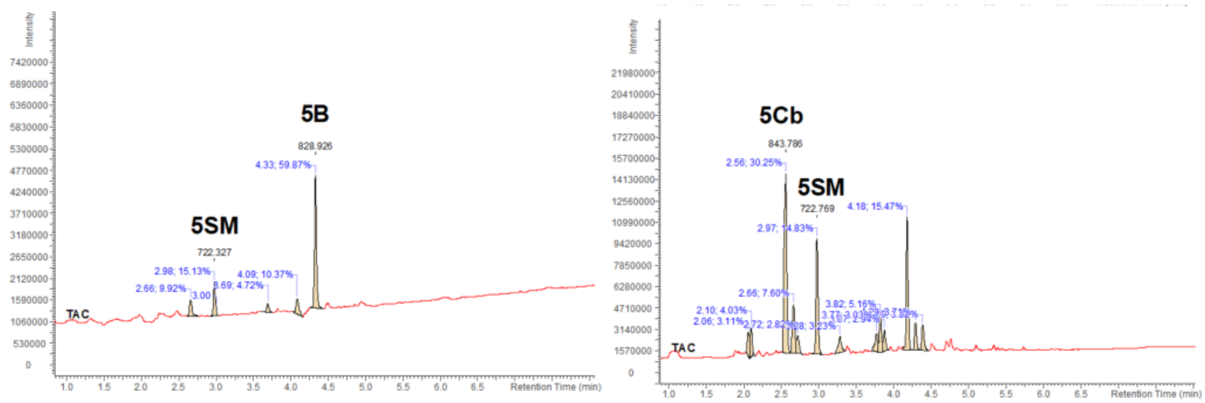

Figure S8. LC-MS spectra (low resolution) of crude reaction mixtures, ***left:*** crude after hydroformylation; ***right:*** crude after reductive amination. ***Row 1:*** Table S5, entry 1 (using **1SM**). ***Row 2:*** Table S5, entry 2 (using **2SM**). ***Row 3:*** Table S5, entry 3 (using **3SM**); after hydroformylation several signals of **3B** found, and two isomers of **3C** after reductive amination in bad selectivity (this could not be explained, but may also be an LC-MS artifact as the upscaled version did have good linear:branched selectivity (see experimental details and attached UPLC profile for **3C**). ***Row 4:*** Table S5, entry 4 (using **4SM**). ***Row 5:*** Table S5, entry 5 (using **5SM**), the analysis with **5SM** gives a cleaner crude reaction mixture after hydroformylation than what the starting material purity of **5SM** would have allowed (could not be explained, likely artifacts); for the reductive amination, the signal at 4.2 min does not correspond to the mass of **5B** but appears only after reductive amination, which was not further investigated. Note: **1SM/B/C/D/E/E'=5SM/B/C/D/E/E'**; with the only difference that they originate from a differently protected starting material (with or without the 2,4-dmb backbone protection).

Notably, the specialized amino acid *L*-allylglycine (Fmoc, dmb) (**SI-6**) required for SPPS to synthesize **5SM**, had to be prepared in two steps as it was not commercially available. SPPS using this double-protected amino acid yielded a low-loading, impure product **5SM** on-resin. As the use of **5SM** serves more as a proof-of-concept in our context (i.e. no detection of **B** and **D** after reductive amination), rather than being the simplest solution to avoid cyclization, no further efforts to optimize the synthesis of **5SM** were made for this study.

(ii) Dependence on reaction time and temperature

We next evaluated how reaction time/ temperature influence cyclization, using **15SM** (Table S6). Collectively, extended reaction times/ elevated reaction temperatures improve overall conversion at the expense of enhanced amounts of cyclization products.

Table S6. Comparison of varying reaction time/ temperature that influence cyclization, using peptide **15SM**.

Entry	Time [hours]	Temp.	Conversion to A (l:b) [%][a,b]	Conversion of SM [%][a,b]	Yield of Cb (l:b) [%][a]	Yield of B (l:b) [%][a]	Yield of D (l:b) [%][a]
1	16	RT	40 (37:3)	40	28 (26:2)	9 (8:1)	3
2	40	RT	60 (55:5)	60	29 (27:2)	26 (25:1)	8
3	60	RT	70 (65:5)	71	21 (19:2)	40 (39:1)	11
4	16	40 °C	83 (74:9)	90	11 (9:2)	58 (56:2)	16
5[c]	16	60 °C	89 (82:7)	95	7 (6:1)	51 (50:1)	26

Hydroformylation: Chamber A – SilaCOgen (0.2 mmol), granular $NaBH_4$ (0.2 mmol), diglyme (1 mL). Chamber B – **15SM** (ca. 5-6 µmol, 34 mg), Rh/6-DPPon (1:5, 6.9 µmol), DMSO (1 mL), RT, 60 h, shaking. *Reductive amination:* aldehyde **A** (small test amount of resin), benzylamine in CH_2Cl_2, 1% AcOH (0.12 M, 0.5 mL), RT, 2 h, $NaCNBH_3$ in CH_2Cl_2:MeOH, 3:1 (0.15 M, 0.5 mL), RT, 1 h, shaking. *Cleavage/Deprotection:* TFA:EDT:H_2O (92.5:5:2.5), 3 h, RT. [a]Yield estimated by LC-UV-MS after cleavage with TFA:EDT:H_2O (92.5:5:2.5) via comparing the peak integration. [b]Conversion to aldehyde **A** (and cyclized analogs) and conversion of **SM** estimated by cleaving a test portion of the resin after hydroformylation with TFA:EDT:H_2O (92.5:5:2.5) and analyzing the purity of formed thioacetal **B** and remaining **SM**. [c]Chamber B – DMSO:THF (17:3).

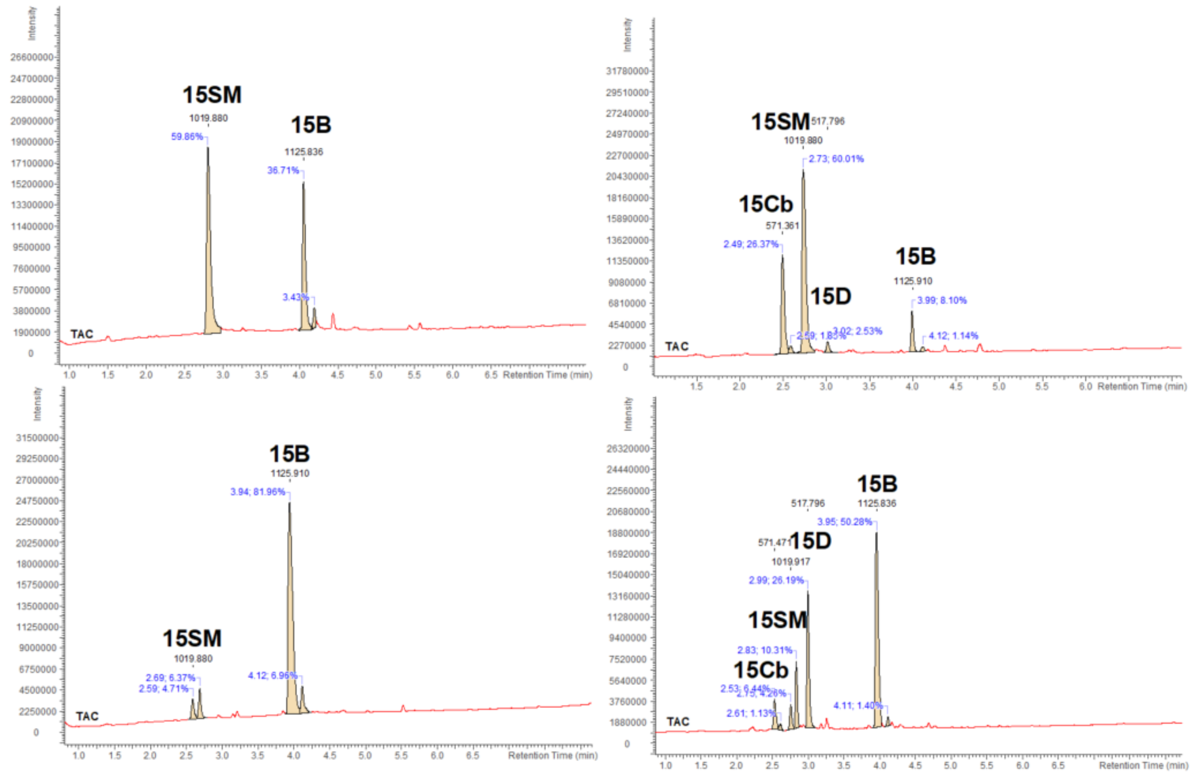

Figure S9. LC-MS spectra (low resolution) of crude reaction mixtures, ***left:*** crude after hydroformylation; ***right:*** crude after reductive amination. ***Row 1:*** Table S6, entry 1 (hydroformylation for 16 h at RT). ***Row 2:*** Table S6, entry 5 (hydroformylation for 16 h at 60 °C).

(iii) Dependence on the ligand of the catalytic system

We next examined whether the catalytic system affects cyclization, using peptide **15SM** (Table S7). The standard ligand used throughout this project was 6-DPPon. To assess ligand effects, we examined alternative ligands such as the monodentate triphenylphosphine (PPh_3) and bis-4-fluoro-6-DPPon. Since using PPh_3 requires elevated temperatures, the reactions were conducted at 60 °C. Using PPh_3 maintained high conversion (91%) and, consistent with precedent literature, delivered the aldehyde (and cyclized analog) **15A** with a lower linear:branched ratio (66:19) (entry 2).[6] Notably, the amount of cyclization was suppressed compared to using 6-DPPon. The amount of cyclization products **15B** and **15D** dropped to 27% and 10% respectively, while the desired amine **15Cb** was yielded in increased amounts of 47% (entry 2). We then compared two ligands active at room temperature, namely 6-DPPon and the more electron-withdrawing bis-4-fluoro-6-DPPon. Both exhibited similar tendencies toward cyclization, with the more electron-withdrawing ligand performing slightly worse in formation of amine vs. cyclization products.

Table S7. Comparison of varying ligands that influence cyclization, using peptide **15SM.**

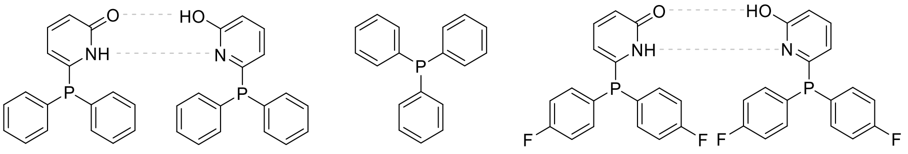

6-DPPon **PPh_3** **bis-4-fluoro-6-DPPon**

Entry	Ligand	Conversion to A (l:b) [%][a,b]	Conversion of SM [%][a,b]	Yield of Cb (l:b) [%][a]	Yield of B (l:b) [%][a]	Yield of D (l:b) [%][a]
1	6-DPPon	89 (82:7)	95	7 (6:1)	51 (50:1)	26
2	PPh_3	88 (66:19)	91	**47 (41:6)**	27 (21:6)	10
3[c]	6-DPPon	70 (65:5)	71	21 (19:2)	40 (39:1)	11
4[c]	bis-4-fluoro-6-DPPon	76 (68:8)	79	14 (11:3)	49 (48:1)	13

Hydroformylation: Chamber A – SilaCOgen (0.2 mmol), granular $NaBH_4$ (0.2 mmol), diglyme (1 mL). Chamber B – **15SM** (ca. 5-6 µmol, 34 mg), Rh/ligand (1:5, 6.9 µmol), DMSO:THF, 17:3 (1 mL), 60 °C, 16 h, shaking. *Reductive amination:* aldehyde **A** (small test amount of resin), benzylamine in CH_2Cl_2, 1% AcOH (0.12 M, 0.5 mL), RT, 2 h, $NaCNBH_3$ in CH_2Cl_2:MeOH, 3:1 (0.15 M, 0.5 mL), RT, 1 h, shaking. *Cleavage/Deprotection:* TFA:EDT:H_2O (92.5:5:2.5), 3 h, RT. [a]Yield estimated by LC-UV-MS after cleavage with TFA:EDT:H_2O (92.5:5:2.5) via comparing the peak integration. [b]Conversion to aldehyde **A** (and cyclized analogs) and conversion of **SM** estimated by cleaving a test portion of the resin after hydroformylation with TFA:EDT:H_2O (92.5:5:2.5) and analyzing the purity of formed thioacetal **B** and remaining **SM**. [c]Chamber B – DMSO (1 mL), 60 h, RT.

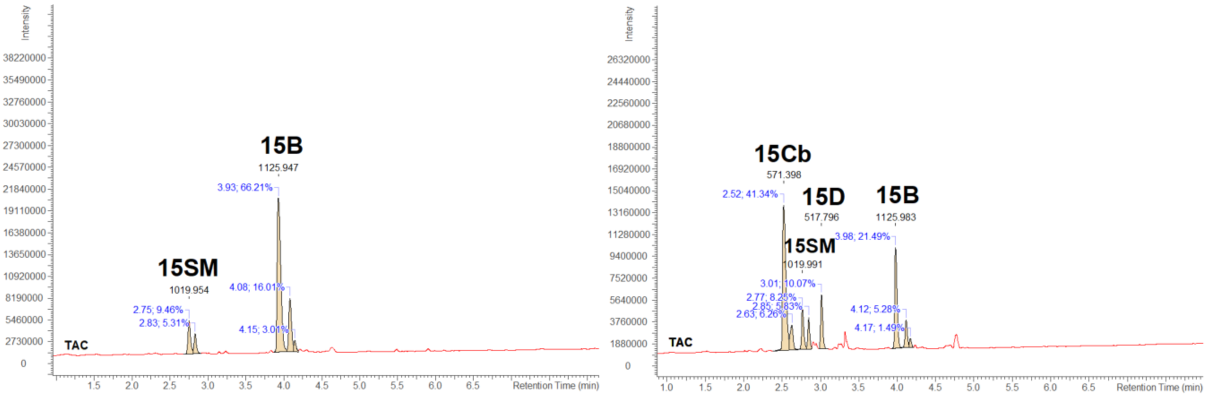

Figure S10. LC-MS spectra (low resolution) of crude reaction mixtures of Table S7, entry 2 (hydroformylation for 16 h at 60 °C using PPh_3 as ligand), ***left:*** crude after hydroformylation; ***right:*** crude after reductive amination.

Building on the observed ligand effect, we conducted a targeted screening with substrate **1SMa** to examine how ligand electronics impact cyclization versus desired amine formation. The metal-hydride bond polarity of the active hydroformylation catalyst is known to influence catalytic activity, with increased acidity facilitating olefin addition and hydrogenolysis of the metal acyl complex during the catalytic hydroformylation cycle.[7, 8] Accordingly, for the more targeted ligand screen we selected phosphine ligands with both electron-withdrawing and electron-donating characteristics: P(*p*-CF_3-C_6H_4)$_3$ (electron-withdrawing groups, EWG), PPh_3 (non-decorated), and P(*p*-OMe-C_6H_4)$_3$ (electron-donating groups, EDG) (Table S8).

Table S8. Dependence of cyclization using different phosphine ligands with peptide **1SMa.**

Entry	Ligand	Conversion to A (l:b) [%][a,b]	Conversion of SM [%][a,b]	Yield of Cb (l:b) [%][a]	Yield of E and E' [%][a,c]	Yield of B (l:b) [%][a]	Yield of D (l:b) [%][a]
1	P(*p*-CF_3-C_6H_4)$_3$	84 (71:13)	83	17 (15:2)	2	44 (42:2)	19 (17:2)
2	PPh_3	94 (63:31)	94	28	18	30 (26:4)	16 (13:3)
3	P(*p*-OMe-C_6H_4)$_3$	22 (14:8)	22	15 (10:5)	8	<1	0

Hydroformylation: Chamber A – SilaCOgen (0.2 mmol), granular $NaBH_4$ (0.2 mmol), diglyme (1 mL). Chamber B – **1SMa** (ca. 2-3 µmol, 17 mg), Rh/ligand (1:5, 6.9 µmol), DMSO:THF, 17:3 (1 mL), 60 °C, 16 h, shaking. *Reductive amination:* aldehyde **A** (small test amount of resin), benzylamine in CH_2Cl_2, 1% AcOH (0.12 M, 0.5 mL), RT, 2 h, $NaCNBH_3$ in CH_2Cl_2:MeOH, 3:1 (0.15 M, 0.5 mL), RT, 1 h, shaking. *Cleavage/Deprotection:* TFA:EDT:H_2O (92.5:5:2.5), 3 h, RT. [a]Yield estimated by LC-UV-MS after cleavage with TFA:EDT:H_2O (92.5:5:2.5) via comparing the peak integration. [b]Conversion to aldehyde **A** (and cyclized analogs) and conversion of **SM** estimated by cleaving a test portion of the resin after hydroformylation with TFA:EDT:H_2O (92.5:5:2.5) and analyzing the purity of formed thioacetal **B** and remaining **SM**. [c]Signals of **E** and **E'** were combined for an overall yield.

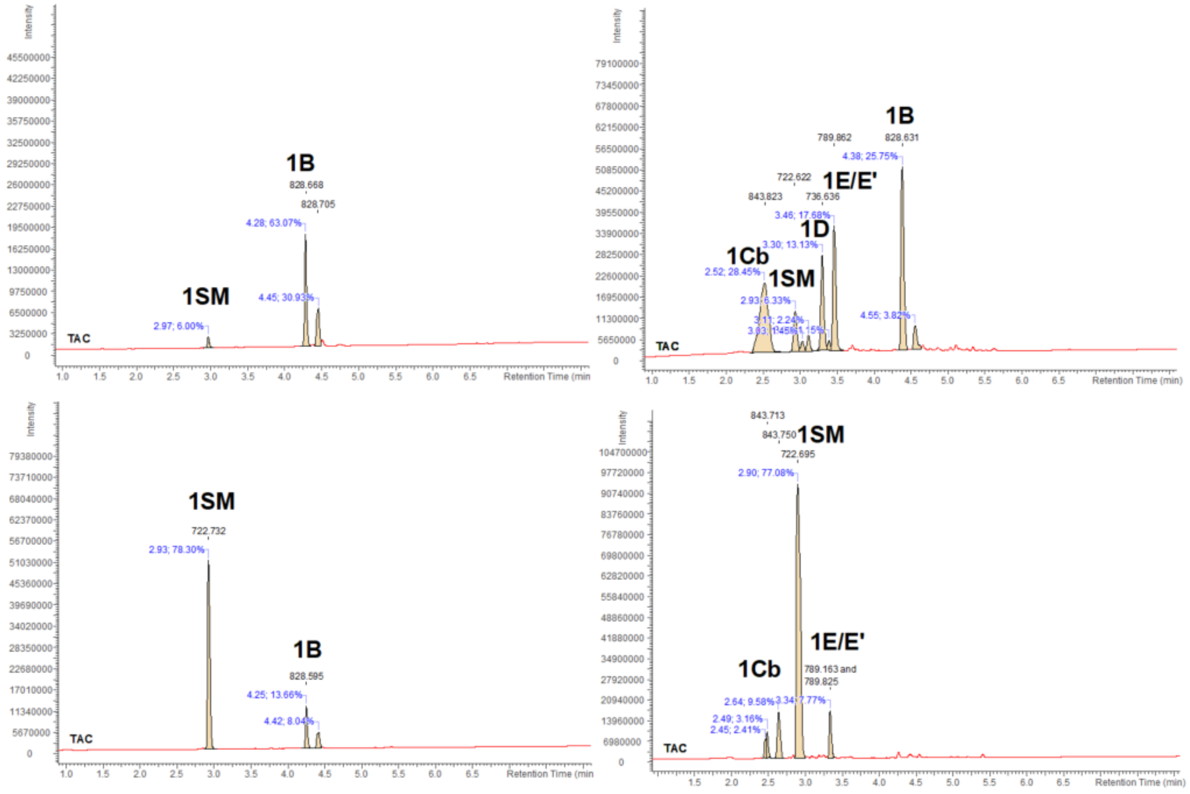

Figure S 11. LC-MS spectra (low resolution) of crude reaction mixtures, ***left:*** crude after hydroformylation; ***right:*** crude after reductive amination. ***Row 1:*** Table S8, entry 2 (hydroformylation for 16 h at 60 °C using PPh_3 as ligand). ***Row 2:*** Table S8, entry 3 (hydroformylation for 16 h at 60 °C using P(*p*-OMe-C_6H_4)$_3$ as ligand).

The reason for varying Rh-catalysts influencing allysine cyclization to a different extent was not further investigated.

(iv) Dependence on solvents

We assessed solvent effects on cyclization, using **1SMa** (Table S9**)**. Introducing water as a co-solvent significantly altered selectivity. Using DMSO:H_2O (7:3), cyclization was completely suppressed: **1B** and **1D** were not detected. Upon 30% water addition, the overall conversion

however decreased from 92% to 52%, likely due to reduced solubility of the catalyst in DMSO:H_2O (7:3), as observed by a turbid reaction medium. A favorable balance was achieved using DMSO:H_2O (95:5) to maximize conversion (98%, catalyst was clearly solubilized) while minimizing cyclization (6% **1B**).

Table S9. Dependence of cyclization on solvents using peptide **1SMa**.

Entry	Solvent	Conversion to A (l:b) [%][a,b]	Conversion of SM [%][a,b]	Yield of Cb (l:b) [%][a]	Yield of E and E' [%][a,c]	Yield of B, l:b [%][a]	Yield of D, l:b [%][a]
1[d]	DMSO	92 (76:16)	92	12 (9:3)	3	64 (61:3)	3
2	DMSO:H_2O (7:3)	50 (48:2)	52	11 (8:3)	32	0	0
3	DMSO:H_2O (7:3), 1% Triton X-100	58 (56:2)	60	12 (8:4)	41	0	0
4	DMSO:H_2O (95:5)	96 (90:6)	98	14 (8:6)	81	6	0

Hydroformylation: Chamber A – SilaCOgen (0.2 mmol), granular $NaBH_4$ (0.2 mmol), diglyme (1 mL). Chamber B – **1SMa** (ca. 2-3 µmol, 17 mg), Rh/6-DPPon (1:5, 6.9 µmol), solvent (1 mL), RT, 60 h, shaking. *Reductive amination:* aldehyde **A** (small test amount of resin), benzylamine in CH_2Cl_2, 1% AcOH (0.12 M, 0.5 mL), RT, 2 h, $NaCNBH_3$ in CH_2Cl_2:MeOH, 3:1 (0.15 M, 0.5 mL), RT, 1 h, shaking. *Cleavage/Deprotection:* TFA:EDT:H_2O (92.5:5:2.5), 3 h, RT. [a]Yield estimated by LC-UV-MS after cleavage with TFA:EDT:H_2O (92.5:5:2.5) via comparing the peak integration. [b]Conversion to aldehyde **A** (and cyclized analogs) and conversion of **SM** estimated by cleaving a test portion of the resin after hydroformylation with TFA:EDT:H_2O (92.5:5:2.5) and analyzing the purity of formed thioacetal **B** and remaining **SM**. [c]Signals of **E** and **E'** were combined for an overall yield. [d]Chamber A – SilaCOgen (40 equiv.), granular $NaBH_4$ (40 equiv.), diglyme (1 mL). Chamber B – **1SMa** (ca. 5-6 µmol, 34 mg), $Rh(COD)_2BF_4$ (1.4 equiv.), 6-DPPon (7 equiv.), DMSO (1 mL).

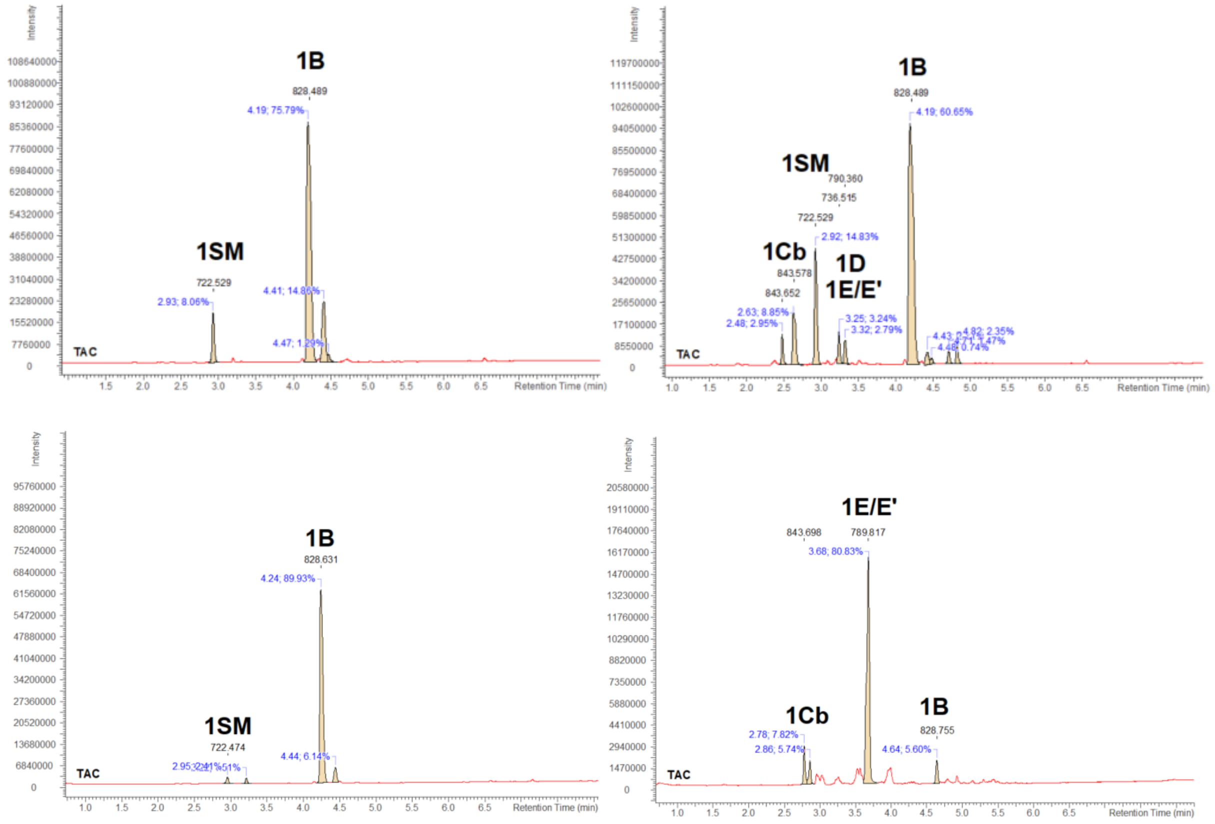

Figure S12. LC-MS spectra (low resolution) of crude reaction mixtures, ***left:*** crude after hydroformylation; ***right:*** crude after reductive amination. ***Row 1:*** Table S9, entry 1 (using DMSO). ***Row 2:*** Table S5, entry 4 (using DMSO:H_2O, 95:5).

Suppressing cyclization revealed a competing side reaction: dimerization during reductive amination. In DMSO:H_2O (95:5), the amount of dimerized products **1E/ 1E'** rose to 81%, whereas under neat DMSO (where cyclization dominated) dimerization was barely observed as a side reaction (3%) (Table S9, entries 1 and 4). Consequently, although cyclization was effectively controlled, the yield of the desired amine product **1Cb** remained low (14%) under the DMSO:H_2O (95:5) solvent conditions, motivating the dimerization-focused studies in the following chapter.

2.4 Challenge 2: Dimerization

The undesired dimerization pathway is proposed in Figure S13. LC-HRMS revealed a signal consistent with **E** and **E'**. Compound **E** can likely be assigned to an enamine that appears stable under LC-MS conditions, allowing its detection. However, the dimer structures were not further characterized.

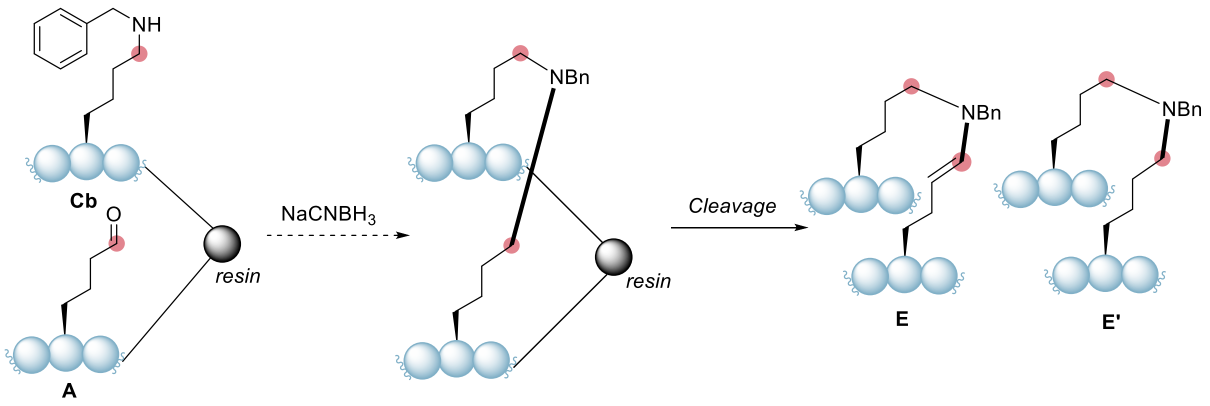

Figure S13. Proposed reaction sequence leading to undesired dimerization on-resin during reductive amination.

To investigate and suppress dimer formation of **2SM**, we modified the post-hydroformylation reaction sequence (Table S10). Substituting benzylamine with tritylamine in the reductive amination eliminated dimerization. The steric bulk of the tritylated lysine likely prevents subsequent reaction with another aldehyde peptide on-resin, hence preventing formation of dimerization products. Due to the steric hindrance of trityl amine compared to benzylamine, the reductive amination was not complete, furnishing 22% of unreacted residual aldehyde, detected in the form of its thioacetal **2B**. These data confirm that bulky/ protected amines can effectively prevent dimerization, although incomplete conversion using bulky amines may necessitate iterative reductive amination. We therefore advise a test cleavage after first reductive amination (using TFA:EDT:H_2O, 92.5:5:2.5) to quantify residual aldehyde and determine the necessity for a second reductive amination cycle. In separate screenings, we also found that using 10% AcOH instead of 1% AcOH can lead to higher conversion of the aldehyde during the first reductive amination cycle when using the bulky tritylamine (data not shown) and can hence be a strategy for more complete conversion during reductive amination (especially if the peptide and its protecting groups are stable at 10% AcOH). As an alternative post-hydroformylation reaction, treatment with the reducing agent $NaCNBH_3$ alone reduced the aldehyde to form the corresponding alcohol **2Ca**.

Table S10. Comparison of different post-hydroformylation reactions and their influence on dimerization.

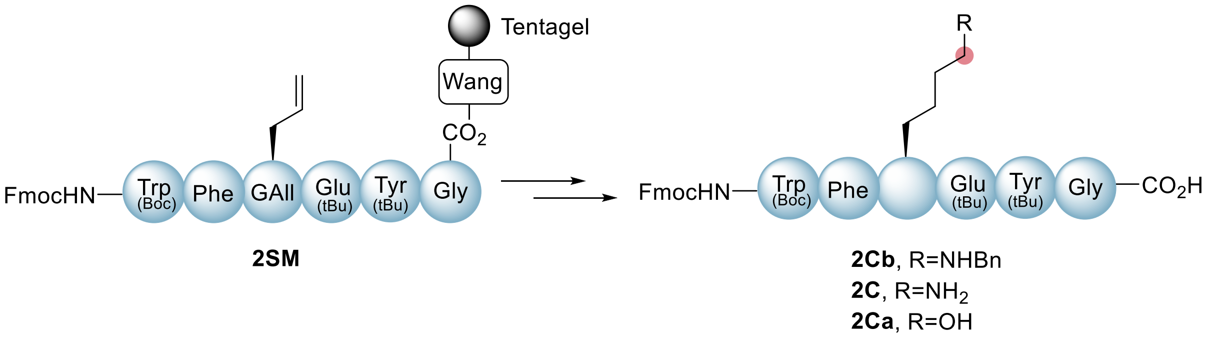

Entry	Amine for red. Amin.	Conversion to A (l:b) [%][a,b]	Conversion of SM [%][a,b]	Yield of C/Cb/Ca (l:b) [%][a]	Yield of Eand E' [%][a,d]	Yield of B, (l:b) [%][a]
**1**[c]	$BnNH_2$	75 (70:5)	75	13 (8:5) (**Cb**)	16	43
**2**	Trt-NH_2	97 (87:10)	97	49 (**2C**)	0	22 (17:5)
**3**[e]	2x Trt-NH_2			52 (**2C**)	0	13 (7:6)
**4**	No amine, only $NaCNBH_3$			69 (54:15) (**2Ca**)	0	9

Hydroformylation: Chamber A – SilaCOgen (0.2 mmol), granular $NaBH_4$ (0.2 mmol), diglyme (1 mL). Chamber B – **2SM** (ca. 5-6 µmol, 34 mg), Rh/6-DPPon (1:5, 6.9 µmol), DMSO:H_2O, 95:5 (1 mL), RT, 60 h, shaking. *Reductive amination:* aldehyde **A** (for entry 2-4 small test amount of resin **A** of entry 2 was used), benzylamine in CH_2Cl_2, 1% AcOH (0.12 M, 0.5 mL) or tritylamine in DMSO, 1% AcOH (0.12 M, 0.5 mL), RT, 2 h, $NaCNBH_3$ in CH_2Cl_2:MeOH, 3:1 (0.15 M, 0.5 mL), RT, 1 h, shaking. *Cleavage/Deprotection:* TFA:EDT:H_2O (92.5:5:2.5), 3 h, RT. [a]Yield estimated by LC-UV-MS after cleavage with TFA:EDT:H_2O (92.5:5:2.5) via comparing the peak integration. [b]Conversion to aldehyde **A** (and cyclized analogs) and conversion of SM estimated by cleaving a test portion of the resin after hydroformylation with TFA:EDT:H_2O (92.5:5:2.5) and analyzing the purity of formed thioacetal and remaining SM. [c]Conditions using only DMSO in hydroformylation. [d]Signals of **E** and **E'** were combined for an overall yield. [e]Reductive amination procedure performed two times.

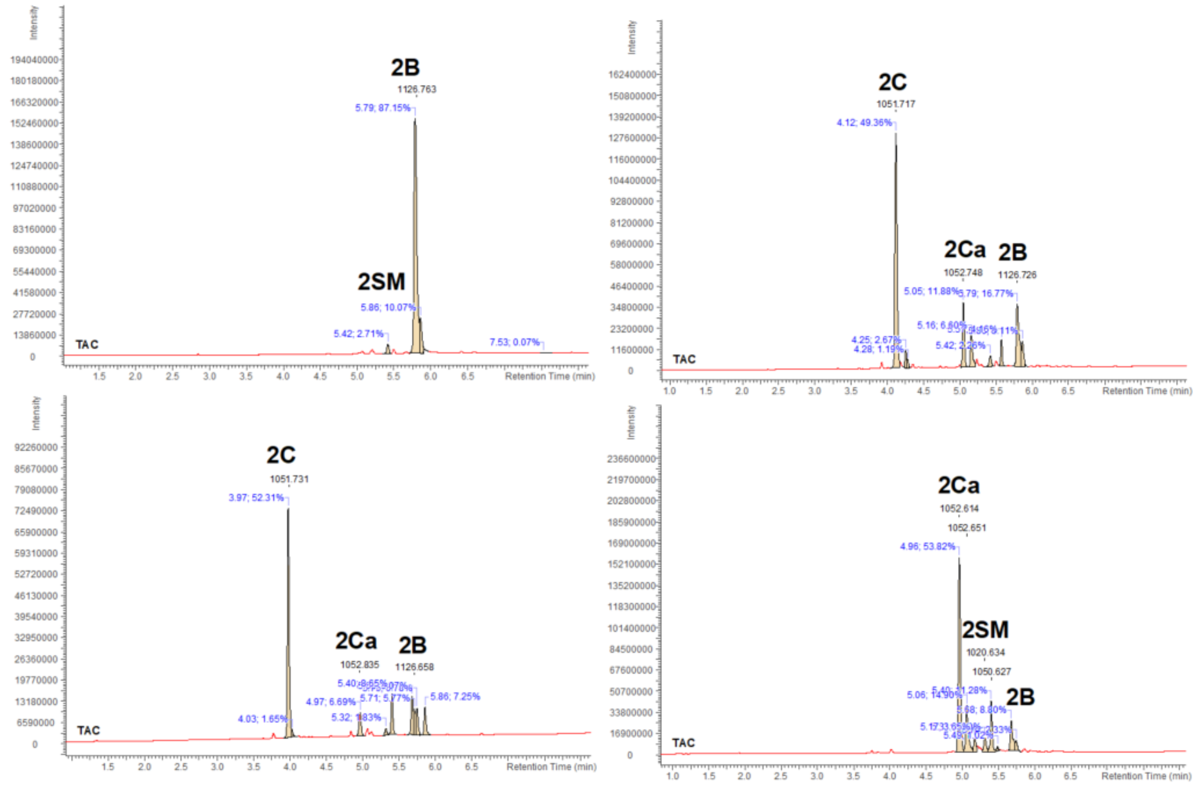

Figure S14. LC-MS spectra (low resolution) of crude reaction mixtures. ***Top-left:*** Table S10**,** entry 2/3/4 crude after hydroformylation; ***top-right:*** Table S10**,** entry 2, crude after reductive amination (using Trt-NH_2), ***bottom-left:*** Table S10**,** entry 3, crude after 2x reductive amination (using 2x Trt-NH_2); ***bottom-right:*** Table S10**,** entry 4, crude after reduction (no amine, only $NaCNBH_3$), the signal at 5.4 min (by low-resolution mass analysis m/z=1050.627) could correspond to a (rarely) observed residual aldehyde **2A** typically not stable under the cleavage conditions (not further analyzed with HRMS).

2.5 Optimization of hydroformylation process for semaglutide analogs

When applying the standard hydroformylation conditions (using ligand 6-DPPon at RT) to the 31-mer precursor of the semaglutide analog **12SM**, neat DMSO could be used as solvent because the 31-mer was not prone to cyclization. However, the hydroformylation conditions had to be optimized (Table S11, Figure S15) due to the reduced isomer selectivity with 6-DPPon (shown by the extracted-ion chromatograms (XIC)) and lower conversion at room temperature. On the basis of yield and selectivity, BIPHEPHOS at 60 °C was adopted as the preferred condition set for semaglutide analogs. The solvent combination DMSO:THF (17:3) was used to solubilize the new ligand.

Analytical note: During LC-MS analysis of the semaglutide analog crude reaction mixtures, we observed occasional carryover, primarily of starting material **12SM**, and to a less extent **12Cb**, into subsequent blank injections, indicating different adsorption/retention of the starting material vs. product to the injection needle, module, or similar. Such carryover can artificially reduce the apparent abundance of **12SM** in the corresponding chromatogram and reported LC-

MS conversions could be skewed due to the **12SM**:**12Cb** ratios. The reported LC-MS yields/conversions should therefore be interpreted with caution; and LC-MS wash protocols are necessary (by performing blank injections after each run for this peptide). Additionally, isomerized starting material (internal olefin) or hydrogenated starting material may co-elute with the starting material containing the allylglycine residue, which could not be accounted for when reporting starting material conversion/ remaining starting material in crude UPLC-UV profile using low-resolution mass spectrometry, especially for triple-charged semaglutide analogs.

Table S11. Optimization of hydroformylation of the semaglutide analog **12SM**.

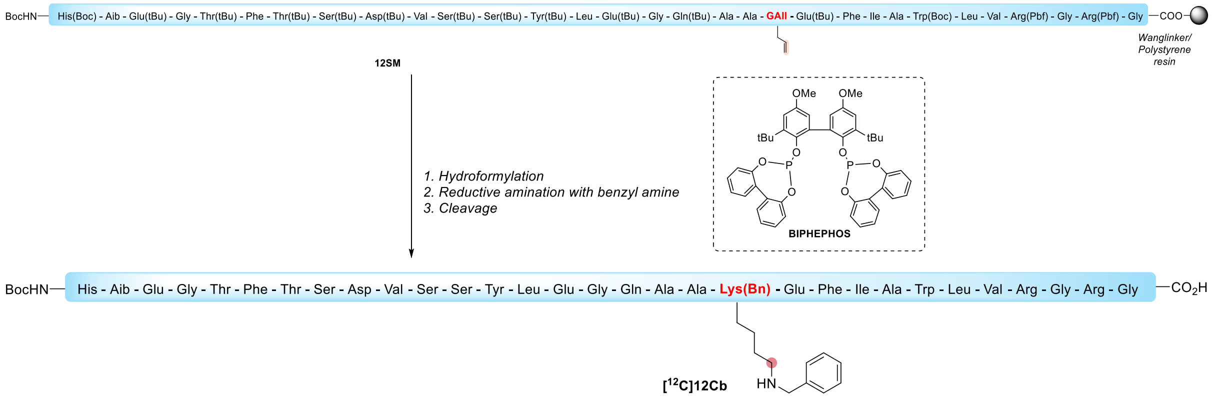

Entry	Ligand	Temp.	Conversion to A, l:b [%][a,b]	Purity of remaining SM [%][a,b]	Yield of Cb, l:b [%][a]
1	6-DPPon	RT	33 (24:9)	58	30 (21:9)
2	BIPHEPHOS	RT	8	78	8
3	6-DPPon	60 °C	50 (40:10)	39	47 (39:8)
4	BIPHEPHOS	60 °C	50	29	43

Hydroformylation: Chamber A – SilaCOgen (74 equiv.), granular $NaBH_4$ (74 equiv.), diglyme (1 mL). Chamber B – **12SM** (ca. 2.7 μmol, 34 mg), Rh/6-DPPon (1:5, 6.9 μmol) or Rh/BIPHEPHOS (1:2.5, 6.9 μmol), DMSO:THF, 17:3 (1 mL), 60 °C or RT, 60 h, shaking. *Reductive amination:* aldehyde (small test amount of resin), benzylamine in CH_2Cl_2,1% AcOH (0.12 M, 0.5 mL), RT, 2 h, $NaCNBH_3$ in CH_2Cl_2:MeOH, 3:1 (0.15 M, 0.5 mL), RT, 1 h, shaking. *Cleavage/Deprotection:* TFA:EDT:H_2O (92.5:5:2.5), 3 h, RT. [a]Yield estimated by LC-UV-MS after cleavage with TFA:EDT:H_2O (92.5:5:2.5) via comparing the peak integration. [b]Conversion to aldehyde **A** and conversion of **SM** estimated by cleaving a test-portion of the resin after hydroformylation with TFA:EDT:H_2O (92.5:5:2.5) and analyzing the purity of formed thioacetal **B** and remaining **SM**.

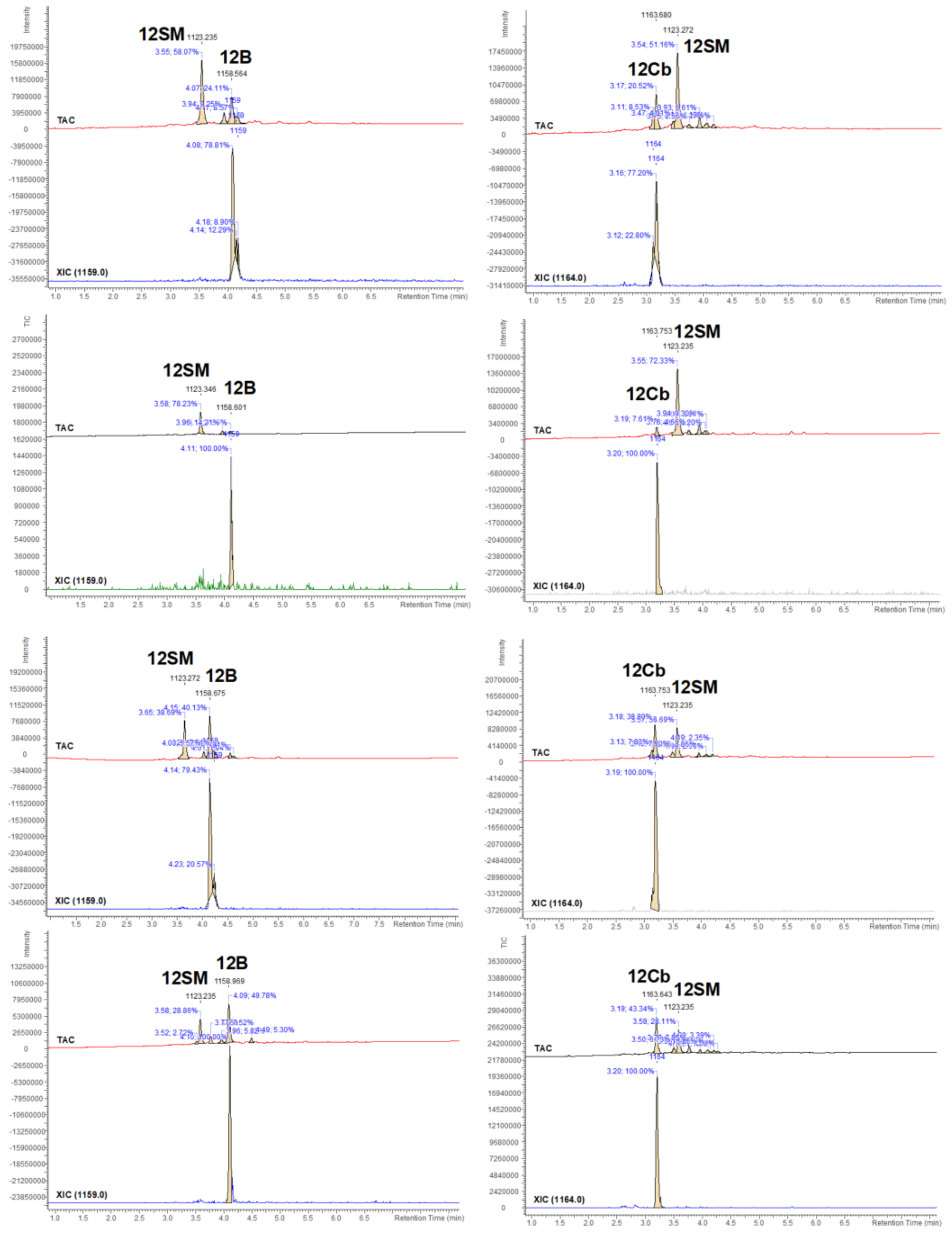

Figure S15. LC-MS spectra (low resolution) of crude reaction mixtures (TAC), and extracted ion chromatograms (XIC) for **12B** (left,) and for **12Cb** (right); ***left:*** crude after hydroformylation; ***right:*** crude after reductive amination. ***Row 1:*** Table S11, entry 1 (using 6-DPPon at RT). ***Row 2:*** Table S11, entry 2 (using BIPHEPHOS at RT). ***Row 3:*** Table S11, entry 3 (using 6-DPPon at 60 °C). ***Row 4:*** Table S11, entry 4 (using BIPHEPHOS at 60 °C).

2.6 Analysis of cyclized products

To characterize cyclization products in more detail, hydroformylation of peptide **15SM** was conducted on a larger scale (21 µmol) using neat DMSO (Scheme S2), a solvent condition that

we identified to promote cyclization for this peptide at lower scale (Table S4, entry 1). After hydroformylation of **15SM**, the resin (containing aldehyde, suspected cyclized analogs, and residual starting material) was divided into three portions and subjected to different cleavage protocols.

Cleavage (i), TFA:EDT:H_2O (92.5:5:2.5): EDT scavenging of the aldehyde (and analogs) led to formation of solely thioacetal **15B** in 61% yield by UPLC-UV (47% isolated, as highlighted in the main manuscript for late-stage functionalization). The crude LC-MS profile is shown in Figure S16. Under these strongly acidic conditions, we expect that EDT either reacts directly with cyclized aldehyde analogs or that cyclized forms equilibrate back to the aldehyde, which is then trapped as the thioacetal, allowing full conversion of all aldehyde analogs.

Cleavage (ii), TFA:H_2O (95:5): To favor observation of cyclized species, cleavage was performed without thiol or silane scavenger. LC-MS analysis revealed, in addition to residual **15SM**, cyclized peptide masses. Only the enamide masses were detected by MS (likely due to hard ionization); however, after cleavage, purification and NMR analysis we assume the analogs in the DMSO-d6 solvent for NMR analysis being the hemiamidal analogs supported by ^{13}C-NMR with the ^{13}C-label having a shift of 82–86 ppm. The structure of the proposed products are six-membered **15A'a** (<54% yield by UPLC-UV, <32% isolated, co-elutes with **15SM**) and seven-membered **15A'b** (30% yield by UPLC-UV, 12% isolated). Based on MSe fragmentation, we propose **15A'a** as a six-membered ring that is formed via the neighboring backbone nitrogen toward the *N*-terminus, and **15A'b** as a seven-membered ring that is formed via the neighboring backbone nitrogen toward the *C*-terminus; however, structures were not fully elucidated (2D-NMR and MS data are attached; not fully characterized). Notably, the isomers exhibited distinct LC-MS retention times (2.75 min and 3.25 min), despite both likely being hemiamidals and therefore are expected to have similar retention characteristics. Preliminary reactivity tests (not shown) suggest different reactivity of these isomers with EDT. Combined with the full conversion observed in Cleavage (i)-conditions, this may indicate that only one cyclized isomer – likely **15A'a** – predominates on resin, while the other may form mainly during the acidic cleavage. This hypothesis was not further investigated and proven. While the selectivity for different isomer formation under different conditions, as well as their distinct reactivities are, in principle, worthwhile topics for future work, they were outside the scope for this study.

Cleavage (iii), TFA:TiPS:H_2O (92.5:5:2.5): Noteworthy, the reaction was conducted three times in total (3x 3 h), to allow increased formation of **15D**. Using TiPS as scavenger produced three additional signals to the starting material **15SM**; for two of them we could propose a structure. According to MS-data, one corresponds to a reduced cyclic product, assigned as the reduced six-membered ring **15D** (with a homoproline residue) (20% yield by UPLC-UV, 17% isolated). MSe analysis indicates **15D** as the reduced form of **15A'a**, whereas **15A'b** seemed to not yield an identifiable reduced analog under these conditions. However, as with **15A'a** and **15A'b**, structures were not fully confirmed; 2D-NMR spectra are provided but not assigned. The isotopically enriched signal in the ^{13}C NMR spectrum for **15D** was found at 43 ppm.

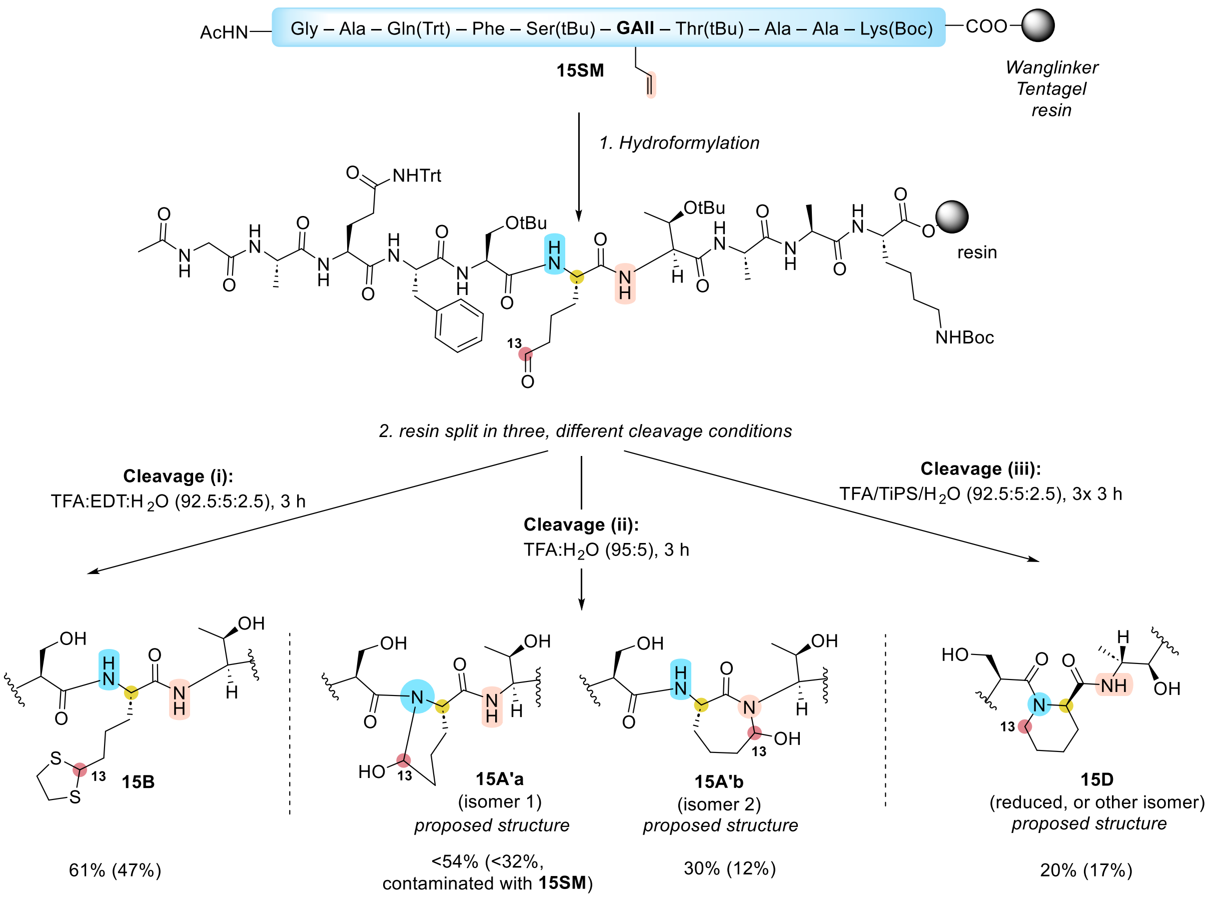

Scheme S2. *Hydroformylation:* Chamber A – Sila^{13}COgen (5 equiv.), $NaBH_4$ (5 equiv.), diglyme (1 mL). Chamber B – **15SM** on Wang linker-tentagel resin (21 µmol, 1 equiv.), Rh/6-DPPon (1:5, 18 µmol), DMSO (2 mL), RT, 60 h, shaking. *Cleavage/Deprotection*: the resin was split in three with each different cleavage procedure. Yields determined by UPLC-UV purity analysis. Isolated yields are given in parentheses. ***Cleavage (i):*** TFA:EDT:H_2O (92.5:5:2.5), RT, 3 h; to promote thioacetal formation. ***Cleavage (ii):*** TFA:H_2O (95:5), RT, 3 h; to either not scavenge the cycles **A'** that formed during hydroformylation (which was realized with EDT), and to further promote cyclization under the acidic cleavage conditions. ***Cleavage (iii):*** TFA:TiPS:H_2O (92.5:5:2.5), RT, 3x 3 h; to promote reduction of the cyclized enamide/hemiamidal **A'**.

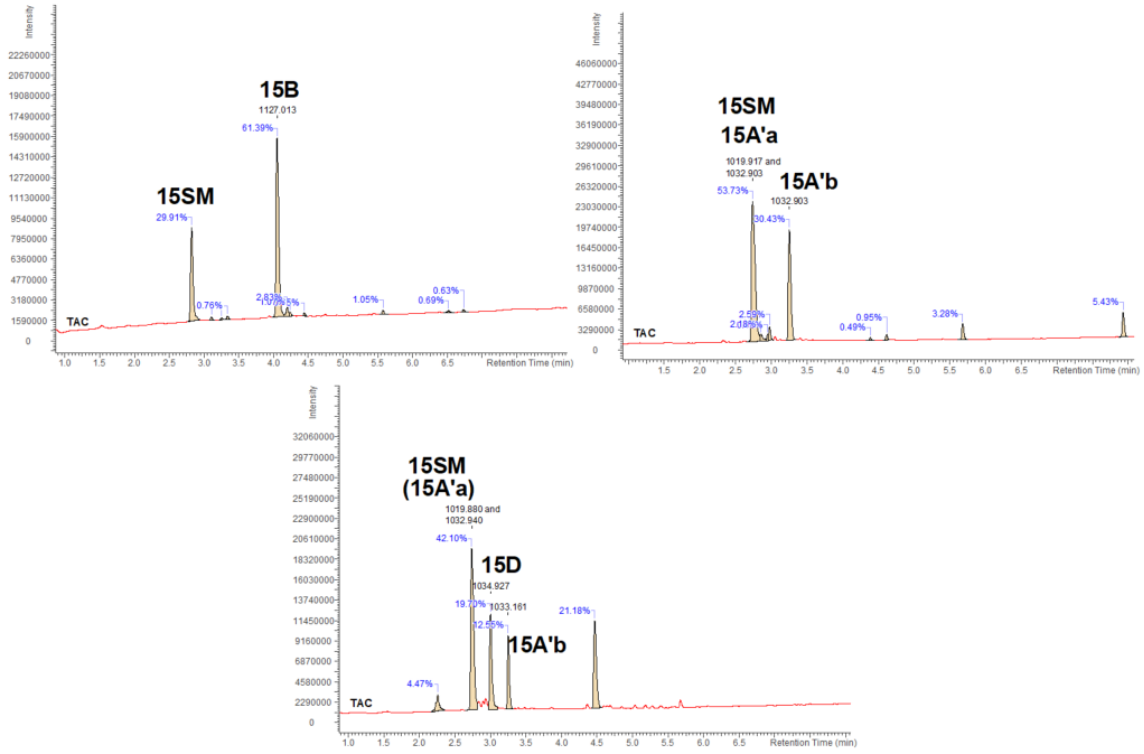

Figure S16. LC-MS spectra (low resolution) of crude reaction mixtures. ***Top-left:*** crude after cleavage with TFA:EDT:H_2O (92.5:5:2.5) (Scheme S2, cleavage (i)); ***Top-right:*** crude after cleavage with TFA:H_2O (95:5) (Scheme S2, cleavage (ii)); ***Top-left:*** crude after cleavage with TFA:TiPS:H_2O (92.5:2.5:2.5) (Scheme S2, cleavage (iii)).

The different cleavage protocols enabled isolation and tentative assignment of possible cyclization product structures from **15SM**, such as the two hemiamidal isomers **15A'a** and **15A'b** without scavenger, and a reduced six-membered cyclized species **15D** with TiPS. Analytical characteristics such as different LC-MS retention times, characteristic ^{13}C-NMR signals (82–86 ppm for hemiamidals; 43 ppm for the reduced ring), preliminary observed selective reactivity toward EDT, as well as the study on dependence on structural features around the reaction centre suggest that a six-membered cyclic hemiamidal (**15A'a**) (under certain conditions in equilibrium with its enamide) could be the predominant on-resin cyclized form; with additional isomers likely arising during acidic cleavage. Further structural confirmation (MS/MS, 2D-NMR analysis) could provide additional insights but was in the purpose of this study not further pursued.

2.7 *Influence of aqueous conditions on isotope incorporation – of small molecule substrate*

This section shows how protic media affect isotope incorporation during deuterium-labeling, with implications for tritium-labeling, using the non-peptidic model-substrate eugenol (**SI-8SM**). Eugenol served both to benchmark setup parameters, and as a convenient, commercially available test substrate before transitioning to more complex peptide substrates (Table S12). The results underscore the need for aprotic media: As D_2O drives ^{2}H-incorporation when operating with H_2, the inverse experiments (H_2O present during D_2 or T_2 runs) will also dilute (radio)isotope incorporation. In summary, achieving high and reproducible ^{2}H/ ^{3}H-labeling requires strict exclusion of proton sources. Variability in labeling levels or different labeling levels of different substrates may be due to water-contamination of solvent/ glassware, or from intrinsic protic functionalities of the substrate.

Table S12. Deuterium incorporation and influence of water.

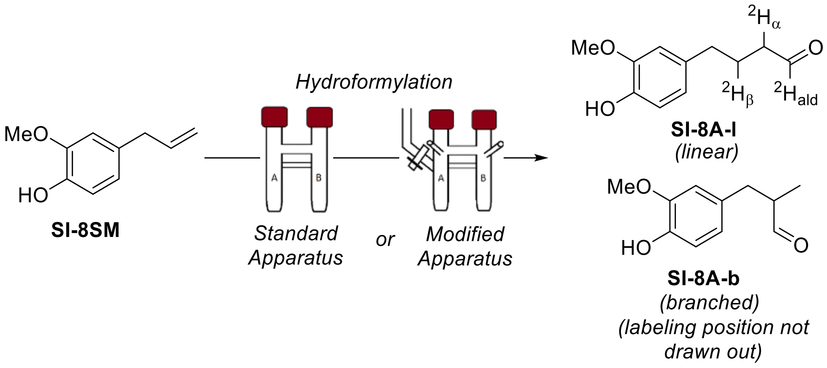

Entry	Two-chamber system	Addition of D_2O [equiv.]	Conversion[b] [%]	Yield of SI-8A (l:b)[b] [%]	Deuteration[b,c] [^2H/molecule]		
					H_{ald}	H_α	H_β
1	Standard	0	>96	98 (99:1)	-	-	-
2[a]	Modified	0	99	86 (84:2)	0.92	0.09	0.68
3	Standard	10	95	90 (85:5)	0.08	0.03	0.08
4	Standard	553	100	60 (59:1)	0.75	0.31	1.11

Hydroformylation with standard apparatus: Chamber A – SilaCOgen (2 equiv.), granular $NaBH_4$ (2 equiv.), THF (1 mL). Chamber B – **SI-8SM** (0.1 mmol), Rh/6-DPPon (1:5, 3.5 mol%), THF (containing 0, 10 or 553 equiv. D_2O) (2 mL), 16 h, 30 °C, stirring. [a]*Hydroformylation with modified apparatus:* $^2H_2$-gas (2 equiv., 280 mbar), Chamber A – SilaCOgen-*d* (2 equiv.), KF (3 equiv.), DMF (1 mL). Chamber B – **SI-8SM** (0.1 mmol), Rh/6-DPPon (1:5, 3.5 mol%), THF (2 mL), 16 h, 30 °C. *Synthesis of SilaCOgen-d:* SilaCOgen (250 mg, 0.5 mmol) was stirred in MeOD (8 mL) for 16 h, and then the solvent was removed under reduced pressure to yield SilaCOgen-*d* as a white solid. [b]Analysis by qNMR using 1,3,5-trimethoxybenzene as internal standard. The ^{1}H-NMR spectrum of **SI-8A-l** was compared with literature details.[9] [c]Deuteration only determined for linear aldehyde **SI-8A-l.**

2.8 Investigation of COD-hydroformylation products

The optimized conditions for the hydroformylation reactions (Manuscript, Figure 4) use a large amount of rhodium-"catalyst", i.e. 0.72 equiv. of the pre-catalyst $Rh(COD)_2BF_4$, which would correlate to effective 1.44 equiv. of cyclooctadiene or 2.88 equiv. of COD-derived double-bond functional groups relative to the terminal alkene of the allylglycine residue. Increasing Rh-catalyst loading (and thus COD ligand) improved peptide conversion (manuscript, Figure 3a) and argues against considerable COD competition under the conditions employed. Since 1,5-COD could however, in principle, consume labeled syngas, we investigated this with the following two experiments:

- Experiment 1: uses the standard hydroformylation conditions (manuscript, Figure 3a) on hexapeptide **2SM**, but using ^{13}CO and deuterated solvents DMSO-d_6:D_2O (95:5). ^{13}C-NMR of the wash solution showed no ^{13}C-aldehyde signals near 200 ppm and therefore no evidence for COD-derived hydroformylation products. Of the initial 36 µmol 1,5-COD only ca. 14 µmol were recovered by qNMR, likely due to the loss of COD into the gas phase. The additional presence of non-identified COD-derived products can also not be excluded.

- Experiment 2: uses more forcing hydroformylation conditions at 60 °C and 0.2 mmol $^{12}CO/H_2$, without any substrate other than the 1,5-COD already present in the Rh-

precatalyst (ca. 36 µmol 1,5-COD). In two parallel experiments, the ligands 6-DPPon and BIPHEPHOS were compared. The reaction using 6-DPPon revealed formation of ca. 2 and 4 µmol aldehyde species, 4–5 µmol 1,5-COD recovery, and 4-5 µmol 1,3-COD formation. When using the ligand BIPHEPHOS, major formation of isomerization product 1,3-COD was found (14 µmol). These experiments reveal that both ligands lead to major conversion of 1,5-COD, and respectively to different COD-hydroformylation product compositions.

Loss of COD into the gas phase and the poor quality of the obtained NMR spectra (presence of solvents) are both sources of consistent errors in the qNMR measurements. The obtained qNMR yields in this chapter should therefore not be regarded as absolute yields, but the relative composition of COD-derived products is more relevant.

Details for Experiment 1:
In the first experiment, the standard conditions (as shown in the manuscript, Figure 3a) were repeated on the hexapeptide-model substrate **2SM** but using ^{13}CO to facilitate detection of formylation products by ^{13}C-NMR. In addition, deuterated solvents (DMSO-d$_6$:D_2O, 95:5) were used during the hydroformylation step. The reaction is shown in Scheme S3, together with the potential side products that may form with 1,5-COD.

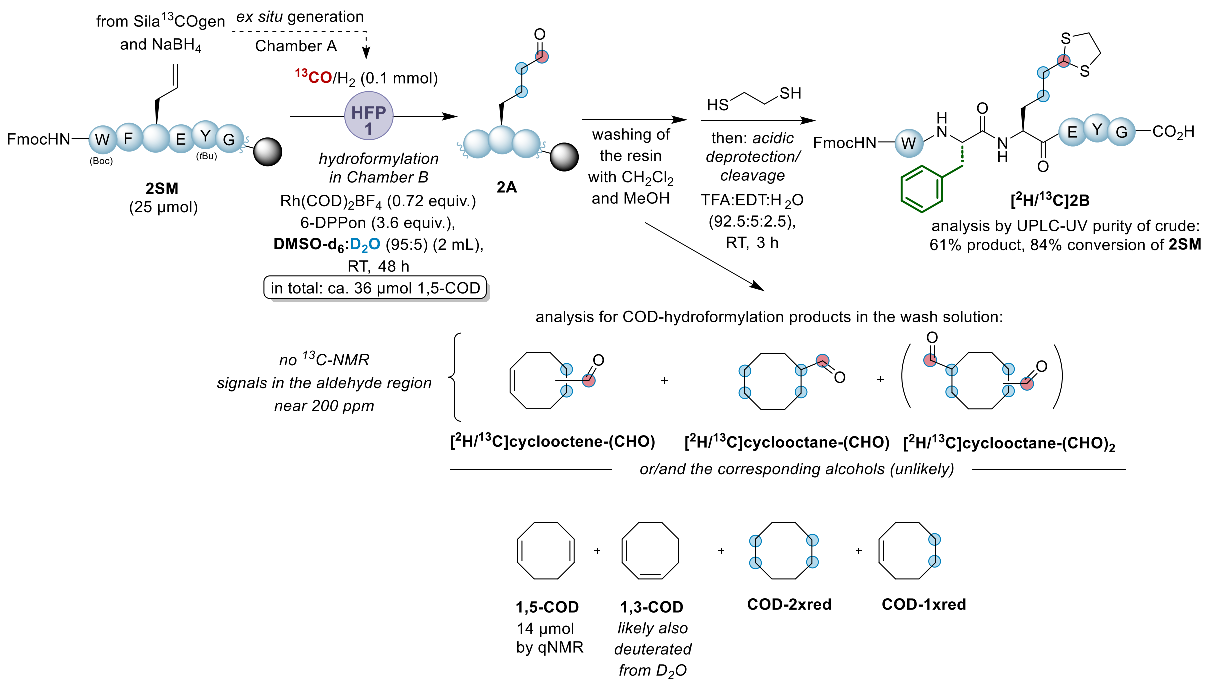

Scheme S3. Hydroformylation reaction with **2SM** using $^{13}CO/H_2$ in DMSO-d$_6$:D_2O, 95:5; and analysis for 1,5-COD hydroformylation products in the resin-wash solution by ^{1}H-NMR, ^{13}C-NMR and GC-MS.

The reaction gave a slightly lower conversion of the starting material **2SM** compared to the results shown in the manuscript (reason not investigated further) and yielded 61% of **[^{13}C, ^{2}H]2B**. ^{13}C-NMR analysis revealed the characteristic ^{13}C-shift of the [^{13}C]thioacetal **2B** at 52.9 ppm (Figure S17).

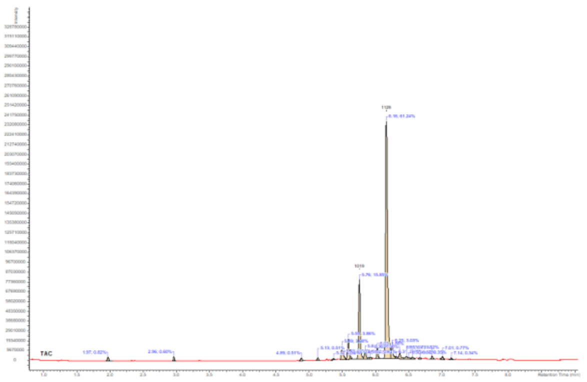

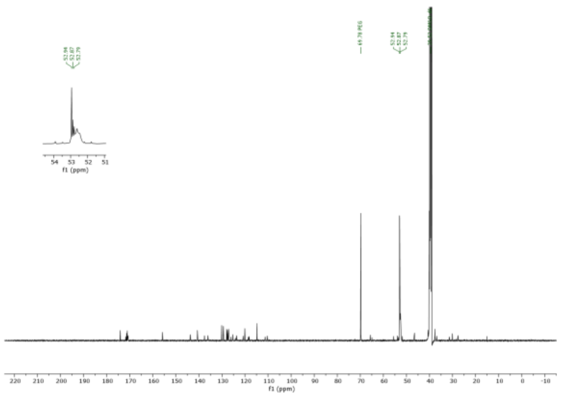

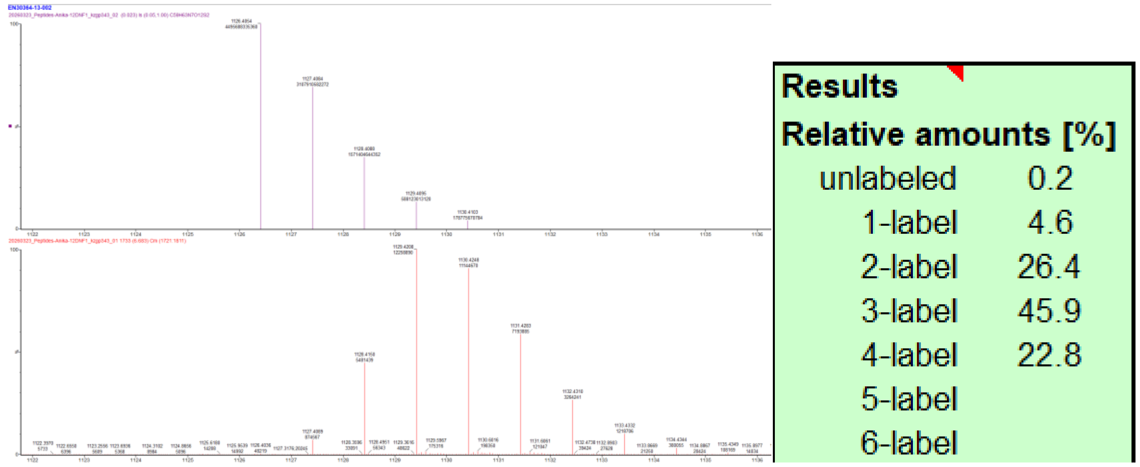

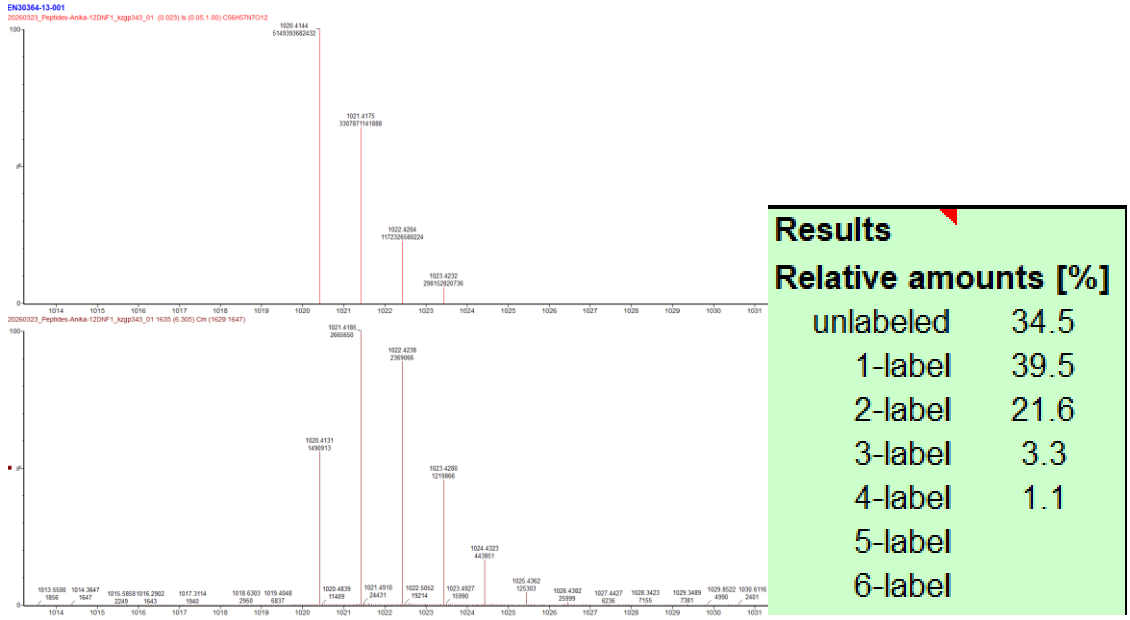

Figure S17. **Top-Left:** UPLC-UV chromatogram of the crude peptide, revealing 61% yield by UPLC-UV purity. **Top-Right:** ^{13}C-NMR of crude peptide **[^{13}C, ^{2}H]2B** and a carbon isotope label at 52.9 ppm of the ^{13}C-labeled thioacetal (the signal at 69.9 ppm stems from the PEG linker). **Middle:** HR-MS analysis of analyte **[^{13}C, ^{2}H]2B** (bottom) vs. predicted unlabeled molecule (top), shows an incorporation of ca. 2.86 stable isotopes/molecule, i.e. ca. 0.99 ^{13}C/molecule, and 1.87 ^{2}H/molecule. **Bottom:** as a side note, the HRMS of the assigned starting material signal in the crude is added, which has a deuterium incorporation of 0.97 ^{2}H/molecule, HR-MS analysis of analyte **[^{2}H]2SM** (bottom) vs. predicted unlabeled molecule (top).

The resin wash solution after hydroformylation containing DMSO-d$_6$, D_2O, MeOH, CH_2Cl_2, and 0.1 mmol internal standard 1,3,5-trimethoxybenzene (TMB) was then analyzed for ^{13}C-

labeled COD hydroformylation products (Figure S18). Quantitative ^{1}H-NMR indicated recovery of ca. 14 µmol 1,5-COD of the initial 36 µmol used. Since D_2O was used in the solvent, any potential COD-derived aldehydes formed would be masked by incorporated deuterium in the ^{1}H-NMR spectrum. However, because no ^{13}C-aldehyde signals in ^{13}C-NMR appeared in the near 200 ppm region, we concluded that no detectable ^{13}C-aldehyde was incorporated, or rather no formation of COD-hydroformylation products could be detected. Reductive hydroformylation of 1,5-COD, i.e. reduction to alcohols, may be more difficult to detect due to the ^{13}C-resonances in the 60 ppm region. However, reductive hydroformylation is rather unlikely with the mild conditions used and since the peptide itself was not undergoing reductive hydroformylation either. Therefore, we did not quantify for formation of alcohols. Another side reaction that can occur with 1,5-COD is isomerization of the double bond or reduction (which would also lead to ^{2}H-incorporation when using D_2O, and masking of the signals). The presence of the washing solvent dichloromethane in ^{1}H-NMR analysis overlaps with potentially isomerized COD that may have been formed. The formation of cyclooctane was not further investigated, but no major signal was observed in the ^{1}H-NMR in the 1.5 ppm region. In other experiments (not detailed here) part of 1,5-COD (despite its high boiling point of 150 °C) could be also detected in the syngas releasing chamber. This explains (in part) why only 14 µmol of 1,5-COD were recovered in the hydroformylation chamber.

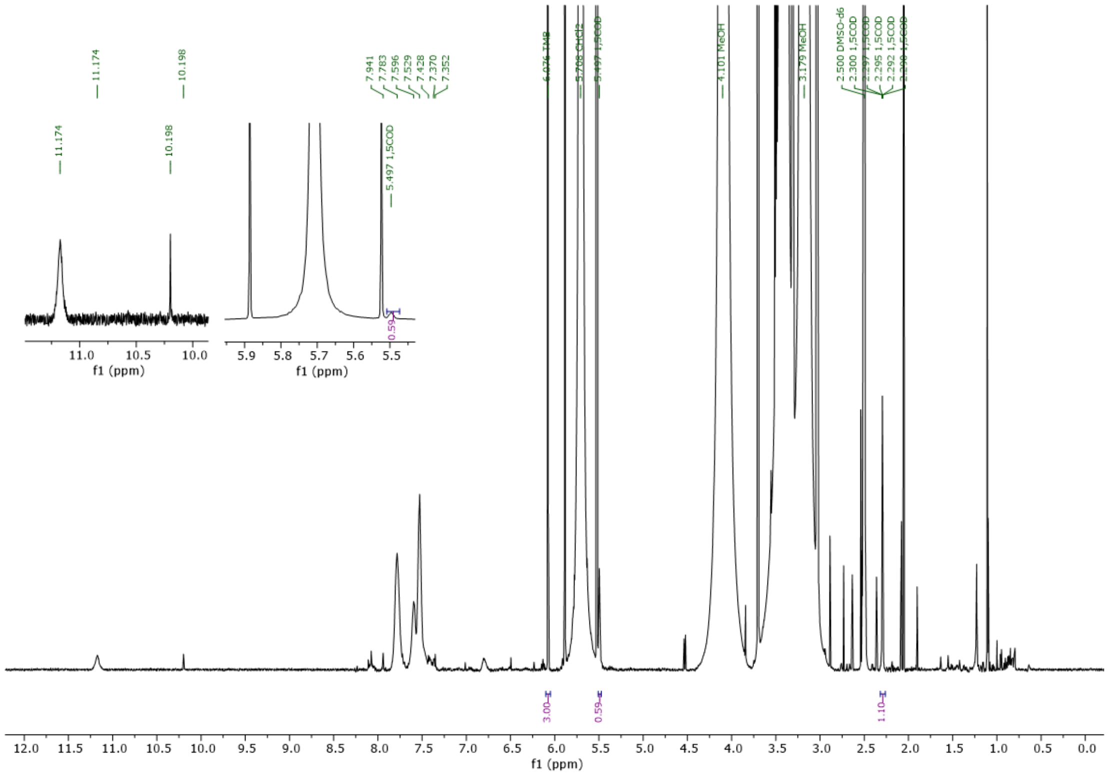

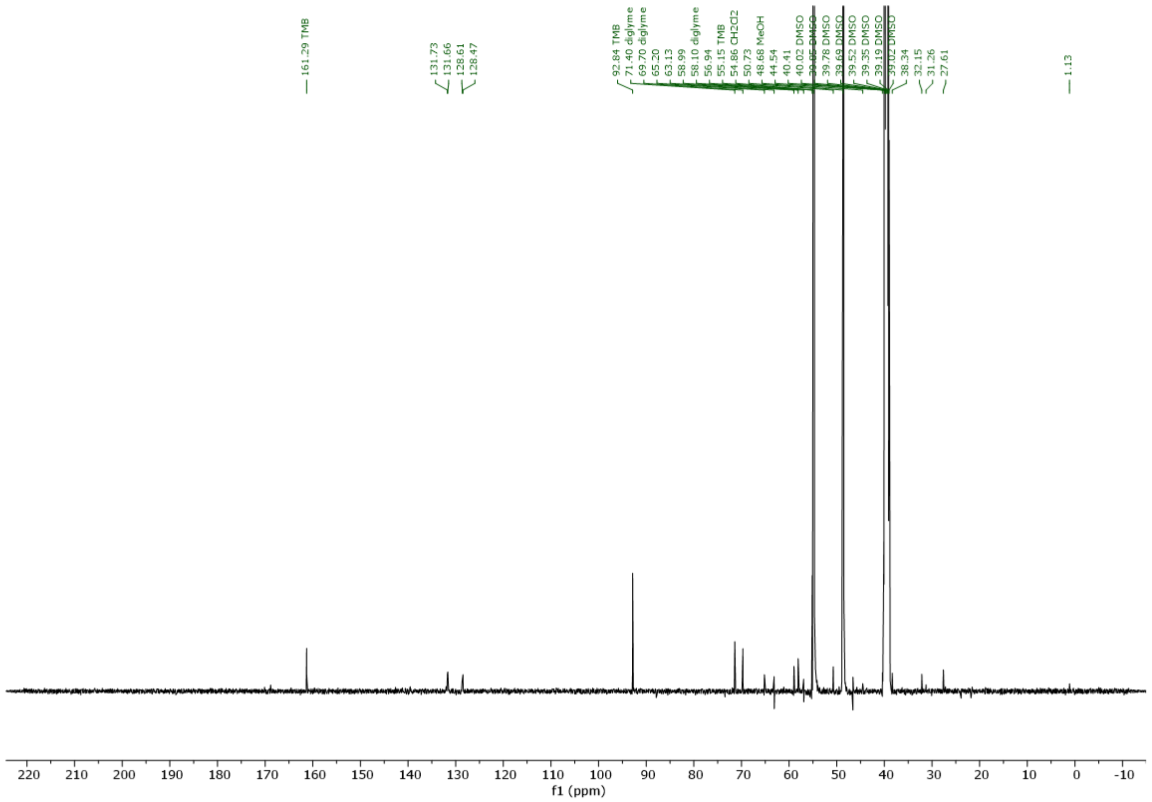

Figure S18. ^{1}H- and ^{13}C-NMR analysis of the resin wash solution after hydroformylation, containing DMSO-d_6, D_2O, MeOH, CH_2Cl_2 and 0.1 mmol internal standard 1,3,5-trimethoxybenzene (TMB), to analyze for ^{13}C-labeled COD-derived hydroformylation products. Quantitative ^{1}H-NMR showed presence of only 14 µmol of 1,5-COD (of the initial 36 µmol used), but no ^{13}C-aldehyde signals (the 161 ppm resonance in ^{13}C-NMR stems from 1,3,5-TMB).

GC-MS analysis of the waste fraction shows the major 1,3,5-TMB internal standard at 6.936 min, alongside 1,5-COD at 4.882 min, and a minor signal at 4.718 and 4.658 min, likely corresponding to deuterated isomerized COD (Figure S19, Row 1 and 2). The signal at 4.969 min is unknown (M=58.1, 59.1).

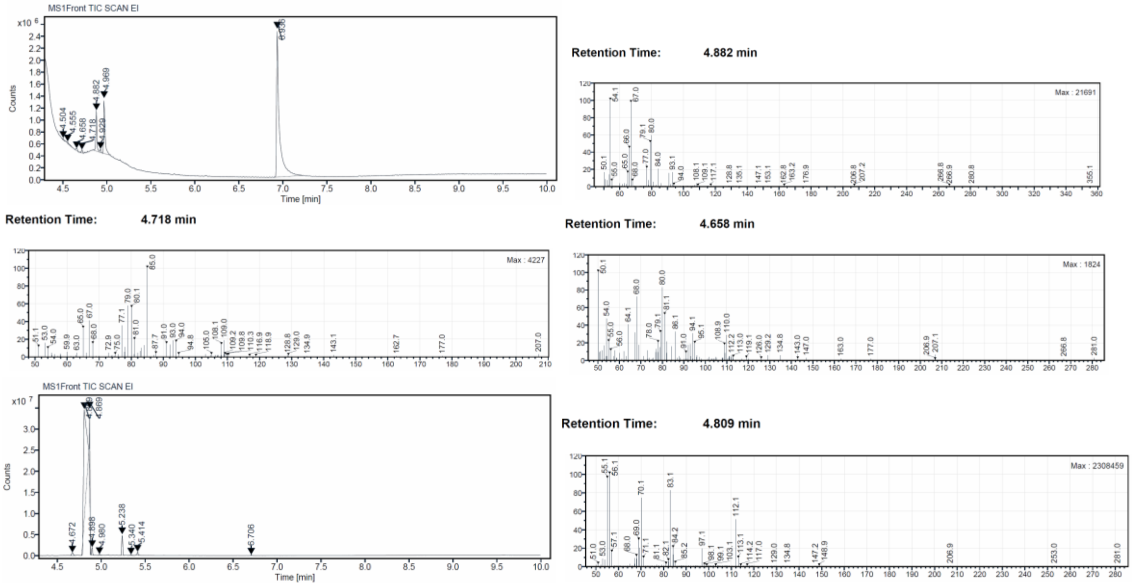

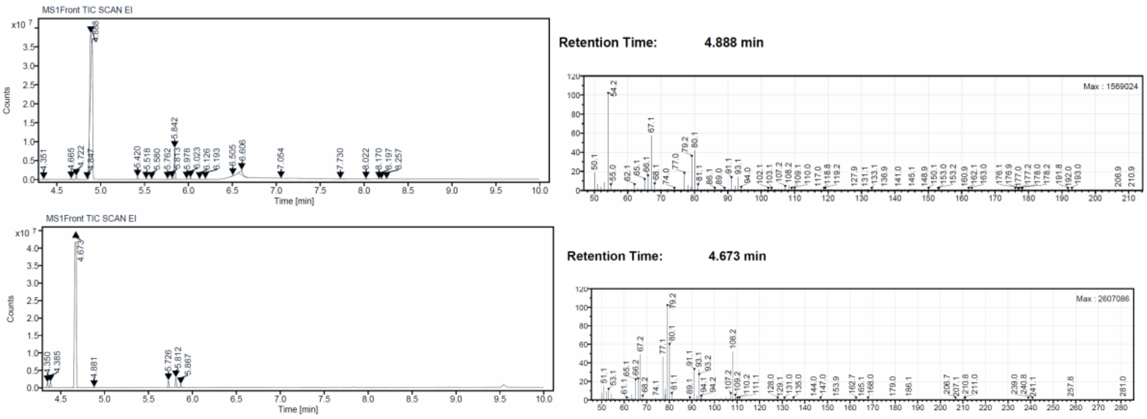

Figure S19. **Row1 and 2:** GC-MS analysis of the wash-solution; signal at 6.931 min is derived from 1,3,5-trimethoxybenzene (internal standard), and signal at 4.969 min is unknown (M=58.1, 59.1). **Row3:** cyclooctane as reference, M=112.1, 56.1. **Row4:** 1,5-cyclooctadiene as reference, fragment M=54.2. **Row5:** 1,3-cyclooctadiene as reference, M=108.2.

Details for Experiment 2:

In a second experiment, we employed more forcing hydroformylation conditions at 60 °C, in the solvent DMSO:THF (17:3), and using 0.2 mmol $^{12}CO/H_2$. Further, no substrate other than the 1,5-COD already present in the Rh-precatalyst was used (ca. 36 µmol 1,5-COD) with a reaction time of only 16 hours. We compared the ligands 6-DPPon and BIPHEPHOS and analyzed the crude reaction in the hydroformylation chamber by qNMR for COD-derived hydroformylation products. The results are shown in Table S13, the qNMR analyses are shown in Figure S20, and GC-MS analyses in Figure S21.

The analyses reveal that when using ligand 6-DPPon two [^{12}C]aldehyde species are formed, i.e. 2 and 4 µmol with respective resonances at 9.59 ppm and 9.54 ppm. Additionally, 4–5 µmol 1,5-COD were recovered and 4–5 µmol 1,3-COD were formed. Instead, when using the ligand BIPHEPHOS no aldehyde species were detected in the 9.5 ppm region, but a signal at 9.38 ppm appeared (ca. 1 µmol). In total, less than 1 µmol 1,5-COD was recovered, while major formation of 14 µmol isomerization product 1,3-COD were found. Whether the signal in the aldehyde region stemmed from the ligand or is a COD-derived aldehyde was not further investigated. The GC-MS analyses were puzzling. In the reaction using 6-DPPon, a peak at 6.117 min with M=120.1 appeared, corresponding to the mass of [^{12}C]cyclooctene-carbaldehyde minus 18 (H_2O). Instead, for the reaction using BIPHEPHOS, this signal did not appear, but instead a trace peak with the mass of 138.1 was found at 6.313 min. This signal would fit the parent mass of the [^{12}C]cyclooctene-carbaldehyde but no aldehyde resonance in ^{1}H-NMR other than the small trace at 9.38 ppm was found.

In summary, we could identify aldehyde resonances in the ^{1}H-NMR that are likely derived from COD, the GC-MS analysis was however inconclusive for the different ligands (different masses/retention times with different ligands, that are both related to the same structure).

Table S13. Analysis of the hydroformylation reaction using the ligands 6-DPPon or BIPHEPHOS at 60 °C without a substrate other than the 1,5-COD present in the pre-catalyst.

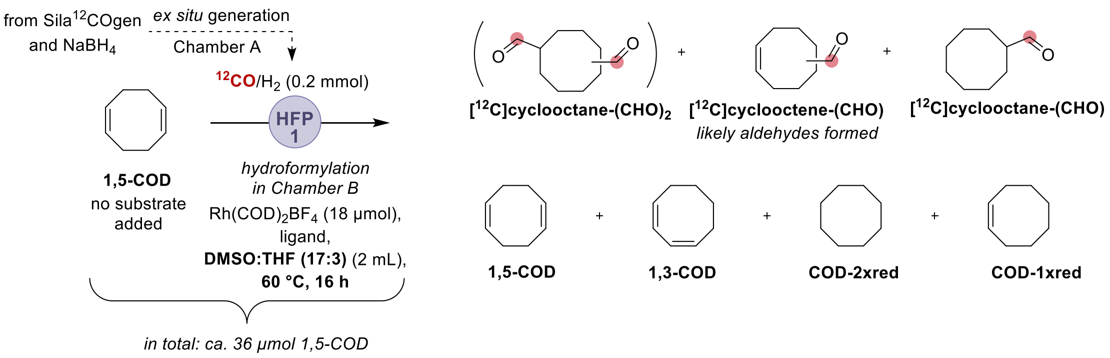

Entry	Ligand	Analysis of crude solution in Chamber B for COD products by qNMR[a]				
		Potential aldehyde species 1 at 9.59 ppm	Potential aldehyde species 2 at 9.54 ppm	Possible aldehyde species at 9.38 ppm	Recovery of 1,5-COD	1,3-COD
1	6-DPPon (90 μmol)	2 μmol	4 μmol	-	4–6 μmol	4–5 μmol
2	BIPHEPHOS (45 μmol)	-	-	1 μmol	<1 μmol	14 μmol

Hydroformylation: Chamber A – SilaCOgen (0.2 mmol), granular $NaBH_4$ (0.2 mmol), diglyme (1 mL). Chamber B – **no substrate** (but ca. 36 μmol 1,5-COD of pre-catalyst present), $Rh(COD)_2BF_4$ (18 μmol)/ 6-DPPon (90 μmol) or BIPHEPHOS (45 μmol), DMSO:THF, 17:3 (2 mL), 60 °C, 16 h, stirring. [a]Using 1,3,5-trimethoxybenzene (0.1 mmol) as internal standard.

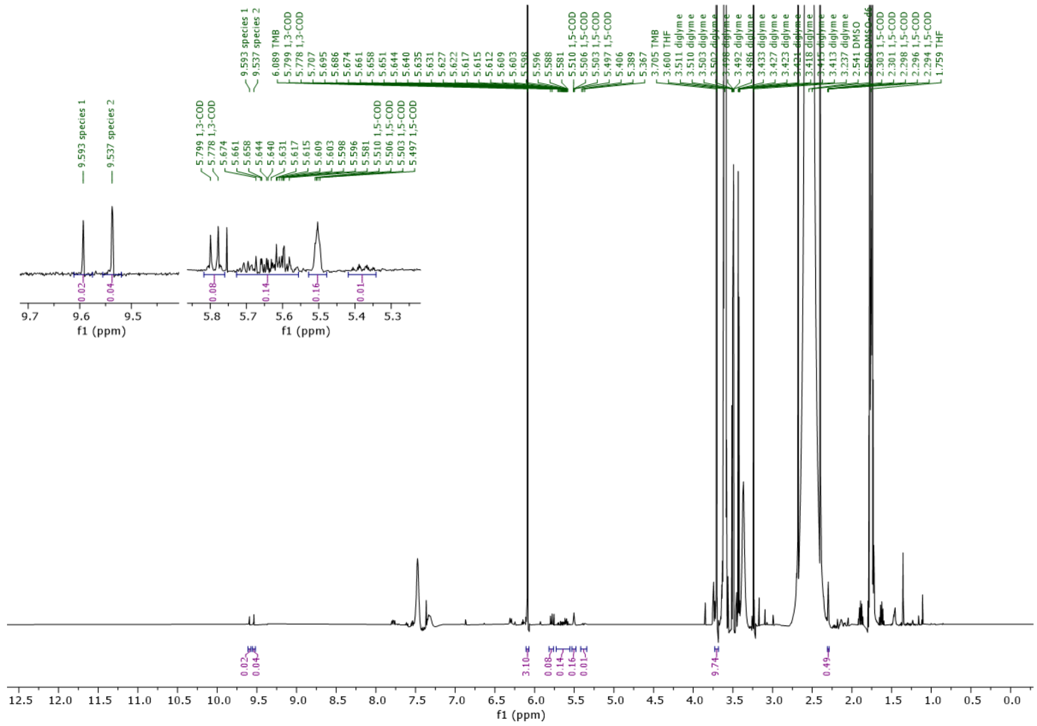

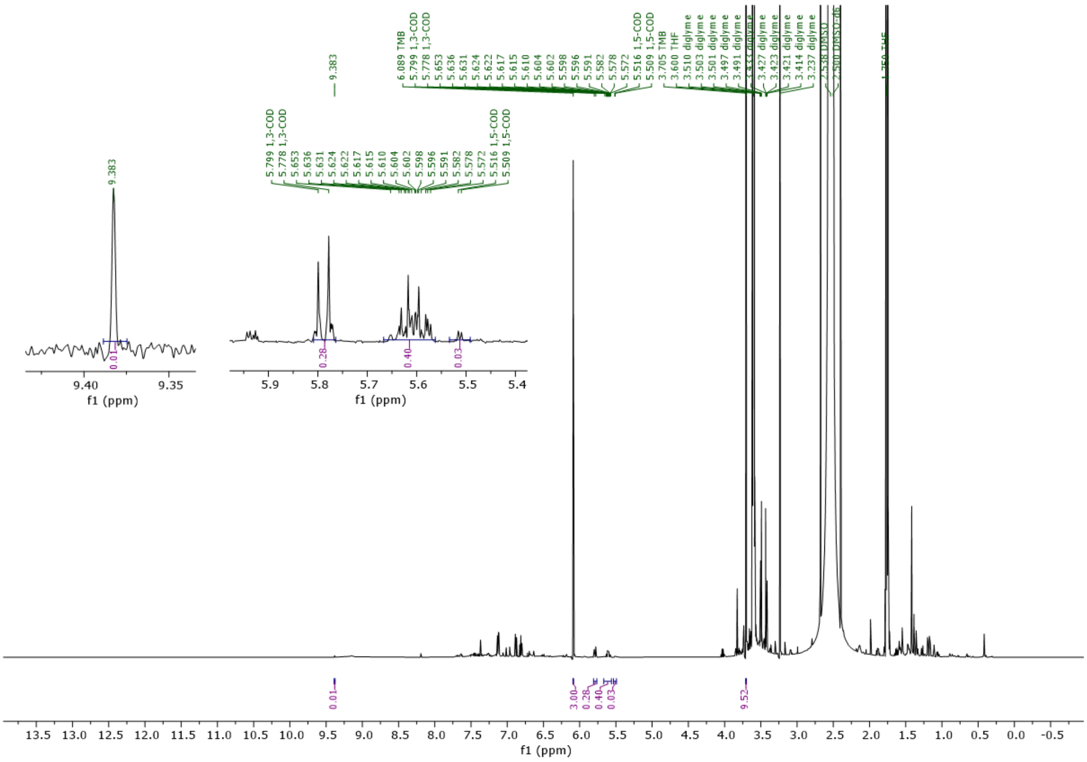

Figure S20. ^{1}H-NMR (qNMR) in DMSO-d_6 of the substrate-free reactions, using internal standard 1,3,5-trimethoxbenzene (TMB) (0.1 mmol). **Top:** using the ligand 6-DPPon. **Bottom:** using the ligand BIPHEPHOS.

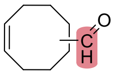

[^{12}C]cyclooctene-(CHO)

likely aldehydes formed

Exact Mass: 138.1045

[M-18]$^{+}$=120.1

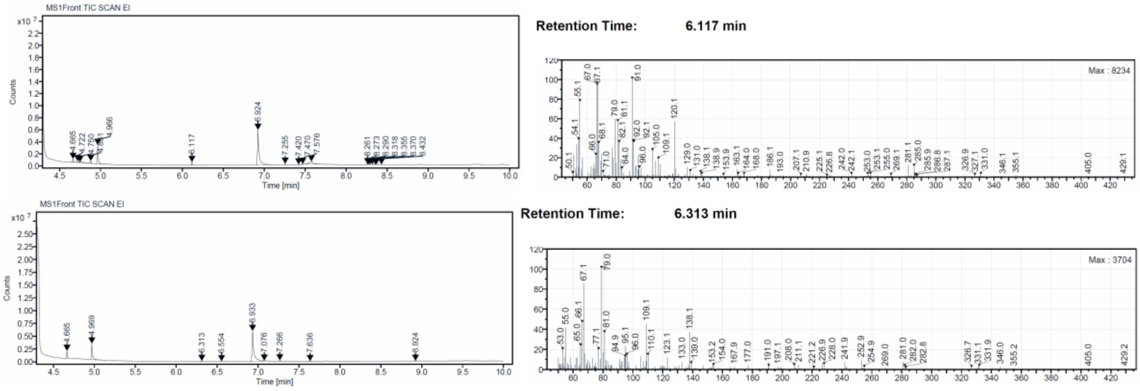

Figure S21. GC-MS analyses. **Top:** using the ligand 6-DPPon, mass 120.1 at 6.117 min found that likely corresponds to [^{12}C]cyclooctene-carbaldehyde (M-18). **Bottom:** using the ligand BIPHEPHOS, mass 138.1 found in trace signal at 6.313 min that could correspond to [^{12}C]cyclooctene-carbaldehyde; inconclusive but may be an artifact.

3. General Procedure for peptide syntheses

3.1 General Procedure GP1A: Manual peptide synthesis

Notes: ca. 10 mL solvent was used per 1 g resin. For resins with a Wang-linker, the first amino acid was pre-loaded. For resins with a 2-chlorotrityl-linker, the first amino acid had to be loaded onto the resin. This first coupling was performed by agitating the resin, the first Fmoc-protected amino acid (1 equiv.) and DIPEA (3.2 equiv.) in CH_2Cl_2 for two hours. Thereafter, the solution is removed by filtration under vacuum, and the resin agitated with the capping mixture CH_2Cl_2/MeOH/DIPEA (17:2:1) (3x 15 min), followed by a wash with 2x CH_2Cl_2/MeOH/DIPEA (17:2:1), 2x CH_2Cl_2, 2x DMF, 2x CH_2Cl_2, 2x Et_2O.

1. The resin was added to the manual peptide synthesizer and swollen for 10 min (under agitation with a nitrogen stream). After swelling, CH_2Cl_2 was removed by filtration under vacuum.
2. Coupling of amino acids: 1–2 equiv. of the amino acid relative to the resin loading was used for the coupling. A mixture of Fmoc-protected amino acid (1 equiv.), DIPEA (3.2 equiv.), and HATU (0.99 equiv.) in DMF was added to the resin and agitated for 3–60 h.
3. Optional capping after couplings or final *N*-terminal acetylation: The resin was agitated with acetic anhydride (5%) and lutidine (6%) in NMP.
4. Deprotection: The resin was agitated with 20% piperidine in DMF for ca. 1 h.
5. Wash after each coupling and each deprotection: with 7x DMF. Wash after last amino acid coupling/final wash: 4x DMF, 2x CH_2Cl_2, 2x Et_2O.

3.2 General Procedure GP1B: Automated peptide synthesis

Notes: The syntheses on the automated peptide synthesizer were in general carried out on a 0.3 mmol scale in a 30 mL fritted plastic vial, using 4 equiv. amino acid relative to the loading of the resin and 0.2 M reaction concentration.

1. The resin was swollen in EtOAc:DMSO (9:1) for 10 min at RT.
2. Deprotection with 20% piperidine in DMF, 2x for 3 and 10 min respectively, at RT. Exception: in cases where the peptide was longer than 17 amino acids, the deprotection procedure was extended, using 20% piperidine in DMF, 3x for 3, 10, and additional 10 min respectively, at RT.
3. Coupling steps when using resin with a Wang-linker: amino acid (4 equiv.), ethyl 2-cyano-2-(hydroxyimino)acetate (=Oxyma) (4 equiv.), *N,N'*-diisopropylcarbodiimide (DIC) (4 equiv.), in DMF, for 10 min at 75 °C.
   Exceptions:
   Coupling with arginine was carried out for 20 min at RT, followed by 10 min at 75 °C.
   Coupling with histidine was carried out 3x, each 20 min at 40 °C.
   Coupling with Aib was carried out 2x, each 10 min at 75 °C.
4. Washes: with EtOAc:DMSO (9:1), 4x 1 min.
   Final wash: with 4x MeOH, 4x CH_2Cl_2.

4. General Procedure for peptide cleavage and deprotection

4.1 GP2A: Hydroformylation-test cleavage of protected peptides from resin with a Wang-/Rink amide linker

A test cleavage of the peptide on-resin after hydroformylation was performed in order to assure successful hydroformylation. The best cleavage cocktail found was TFA:EDT:H_2O (92.5:5:2.5), which was able to convert the aldehydes (or expected cyclized analogs thereof) to the thioacetal analog to provide a clean LC-MS profile typically only composed of the starting material and thioacetal product.

Procedure: A small test-amount of the resin (ca. 3–10 mg) after hydroformylation was added into a 1–2 mL peptide reactor. TFA:EDT:H_2O (92.5:5:2.5, 330 μL) was added, shaken for 3 h, and filtered into a 1.5 mL Eppendorf vial. The solvent was, if necessary, partially removed by carefully blowing air, and 1 mL of cold ether (Et_2O) was added for precipitation. The mixture was centrifuged, the supernatant was discarded, and the peptide pellet was washed with Et_2O or cold Et_2O (3–5x 1 mL) by centrifugation.

4.2 GP2B: Cleavage of protected peptides from resin with a Wang-/Rink amide linker

Peptides that contained protected amino acids were cleaved by shaking the resin with a solution of TFA:DODT:TiPS:H_2O, 92.5:2.5:2.5:2.5 (2 mL) for 3 h, the solution was filtered and the resin washed with TFA (ca. 2x 0.75 mL). To maximize precipitation, the filtrate was, if necessary, partially concentrated under a gentle stream of air. Then, cold diethyl ether (Et_2O, 13 mL) was added. For some peptides (that were more difficult to precipitate), the suspension was cooled in a freezer for ca. five minutes to ensure complete precipitation, followed by centrifugation (either at RT or 4 °C). The supernatant was discarded, and the peptide pellet was washed with Et_2O or cold Et_2O (4–5x 13 mL) by centrifugation.

4.3 GP2C: Cleavage of peptides from a resin with a 2-chlorotrityl-linker

(only applicable for peptides described in the Supporting Information in Section 2.1)
The peptide was cleaved by agitating the resin with CH_2Cl_2:TFE (8:2) for 3x 1 h. After each 1 h, the resin was washed with 5x CH_2Cl_2 and 5x MeOH. The combined solutions were then evaporated *in vacuo*.
Alternatively, the 2-chlorotrityl resin was cleaved with 1% TFA in CH_2Cl_2 for 3x 15 min, filtered, after which the solution was added to 20% pyridine in MeOH. The solvent was removed *in vacuo* until ca. 10 vol% of the solution remained. This solution was crashed out with cold water and the salt was washed by several washes with cold water.

5. General Procedure of hydroformylation reactions for stable isotope applications (^{12}C or ^{13}C and ^{1}H)

The hydroformylation reactions were all performed with dry, deoxygenated solvents (unless water was used as a solvent). The reactions were performed under oxygen-free atmosphere. The practical workflow is depicted in Figure S22.

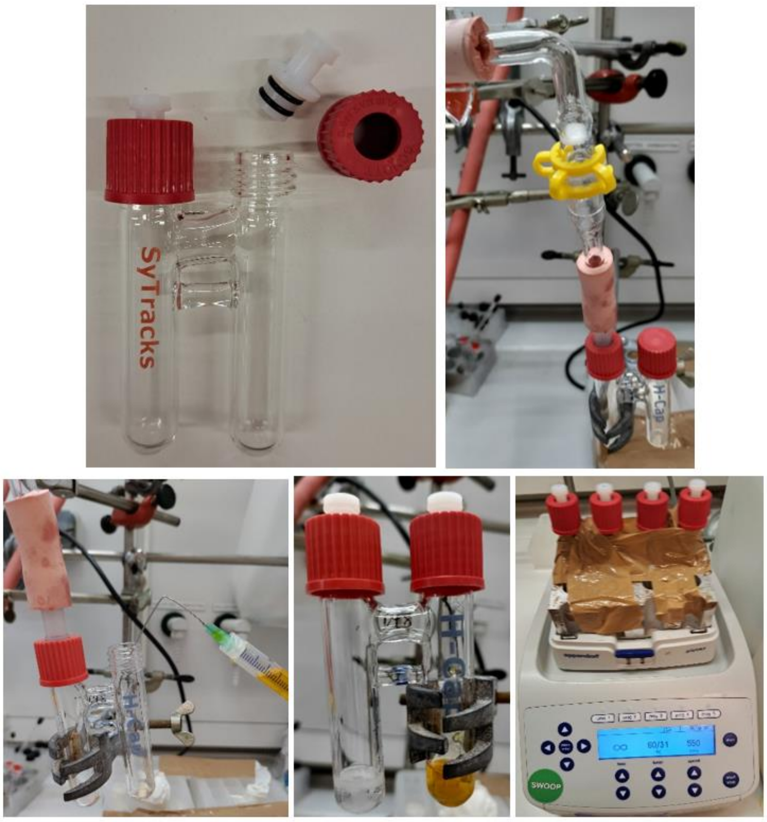

Figure S22. **GP3A and GP3B:** Setup of hydroformylation reaction outside glovebox using Sila^{12}COgen or Sila^{13}COgen in a two-chamber system with H-caps containing O-rings and red screw caps *(**Top, Left**)*. ***Top-Right***, step1**:** Evacuation and nitrogen-refill (3x) of two-chamber system through adapter to Schlenk-line. ***Bottom-left***, step 2**:** Addition of Rh/ligand-catalyst solution to Chamber B (Hydroformylation chamber) under a flow of nitrogen gas, followed by closing Chamber B with an H-cap and screw cap. Step 3 (not shown): quickly removing the nitrogen-gas adapter, quick addition of solvent to Chamber A (syngas-evolution chamber) while keeping the nitrogen flow on top of the opening. Quick closing of Chamber A with H-cap and screw cap. ***Bottom-middle:*** Observation of syngas evolution (slowed down due to use of *granular* $NaBH_4$, allowing enough time to close two-chamber without losing syngas). ***Bottom-right:*** Shaking/Heating by the use of Eppendorf® ThermoMixer® C.

5.1 General Procedure GP3A: [^{12}C or ^{13}C, ^{1}H]Hydroformylation of resin-bound peptides

Due to the work on-resin, the reactions for resin-bound peptides had to be agitated by being shaken rather than stirred with a magnet. The experimental setup is similar to that presented in Figure S22. The exact reaction scale and possible changes in the procedure (highlighted in blue) are detailed in the experimental procedure of the respective compounds.

1. A solution containing $Rh(COD)_2BF_4$ (7.5 mg, 18.5 µmol, ca. 0.92 equiv.) and 6-DPPon (27 mg, 97 µmol, ca. 4.8 equiv.) (or BIPHEPHOS) in freshly deoxygenated solvent DMSO or DMSO:H_2O, 95:5 (2 mL) (or DMSO:THF, 17:3 for BIPHEPHOS) was prepared.

2. The hydroformylation chamber (chamber B) of a standard two-chamber system was prepared with the resin-bound substrate **SM** (ca. 20 µmol, see exact amount in respective individual procedure).
3. To the syngas-producing chamber (chamber A) was added Sila^{12}COgen or Sila^{13}COgen (24 mg, 0.1 mmol, 5 equiv.) and granular $NaBH_4$ (4 mg, 0.1 mmol, 5 equiv.).
4. The catalyst-ligand solution (2 mL) containing $Rh(COD)_2BF_4$ (7.5 mg) and 6-DPPon (27 mg) was added to the substrate in the hydroformylation chamber B under a positive pressure of nitrogen, after which the chamber B was closed with an H-cap and a red screw cap.
5. Diglyme (1 mL) was quickly added under a stream of nitrogen to the syngas-producing chamber (chamber A) which was quickly closed with an H-cap and a red screw cap.
6. The two-chamber reactor was shaken at RT (or 40 °C – 60 °C) for 60 h.
7. Then, the reaction mixture was filtered over a peptide reactor and washed with 3x CH_2Cl_2 and 3x MeOH. The peptide was then subjected to the reductive amination procedure, typically GP6A.

5.2 General Procedure GP3B: [^{12}C or ^{13}C, ^{1}H]Hydroformylation of resin-bound peptides

Due to the work on-resin, the reactions for resin-bound peptides had to be agitated by being shaken rather than stirred with a magnet. The experimental setup is similar to that presented in Figure S22. The main difference compared to GP3A is that the reactions were shaken for 48 h, and the reaction scale was typically 25 µmol. The exact reaction scale and changes in the procedure are highlighted in the experimental procedure of the respective compounds. Independent of different scales, the same molar amounts of catalyst and syngas were used.

1. A solution containing $Rh(COD)_2BF_4$ (7.2 mg, 17.7 µmol, 0.72 equiv.) and 6-DPPon (25 mg, 90 µmol, 3.8 equiv.) in freshly deoxygenated solvent DMSO:H_2O (95:5, 2 mL) was prepared.
2. The hydroformylation chamber (chamber B) of a standard two-chamber system was prepared with the resin-bound substrate **SM** (ca. 25 µmol, see exact amount in respective individual procedure).
3. To the syngas-producing chamber (chamber A) was added Sila^{12}COgen or Sila^{13}COgen (24 mg, 0.1 mmol, 4 equiv.) and granular $NaBH_4$ (4 mg, 0.1 mmol, 4 equiv.).
4. The catalyst-ligand solution (2 mL) containing $Rh(COD)_2BF_4$ (7.2 mg) and 6-DPPon (25 mg) was added to the substrate in the hydroformylation chamber B under a positive pressure of nitrogen, after which the chamber B was closed with an H-cap and a red screw cap.
5. Diglyme (1 mL) was quickly added under a stream of nitrogen to the syngas-producing chamber (chamber A) which was quickly closed with an H-cap and a red screw cap.
6. The two-chamber reactor was shaken at RT for 48 h.
7. Then, the reaction mixture was filtered over a peptide reactor and washed with 3x CH_2Cl_2 and 3x MeOH. The peptide was then subjected to the reductive amination procedure, typically GP6B.

(*Note:* in some cases, a COware® reactor without H-caps but instead stabilizing PTFE discs with PTFE/silicone septa and red screw caps were used)

6. General Procedure of hydroformylation reactions for ^{14}C-, ^{2}H or ^{3}H-labeling

A custom-made modified two-chamber system (Figure S23) was manufactured, in order to apply the hydroformylation reaction to deuterium/tritium labeling (using D_2/T_2 gas) and to ^{14}C-labeling (using ^{14}COgen). The modified two-chamber system with a side arm enables the connection to a deuterium/tritium manifold. This allows the safe introduction of deuterium gas and radioactive tritium gas, and further enables evacuation of the reactor. In Chamber A, ^{12}CO and ^{14}CO could be released from the solid precursor ^{12}COgen and ^{14}COgen. The valve ensured a tight reaction vessel when closed, and enabled the reactor to be detached from the manifold to allow shaking of the reaction mixture. H-caps with O-rings further ensured a tight system, and the small septa with the beige screw caps allowed addition of reagents. These septa were developed by RC Tritec for reactions involving tritium gas to allow the addition of solvents with a syringe while still guaranteeing a tight set-up without leakage of tritium gas, as might be possible when using standard glassware with septa.

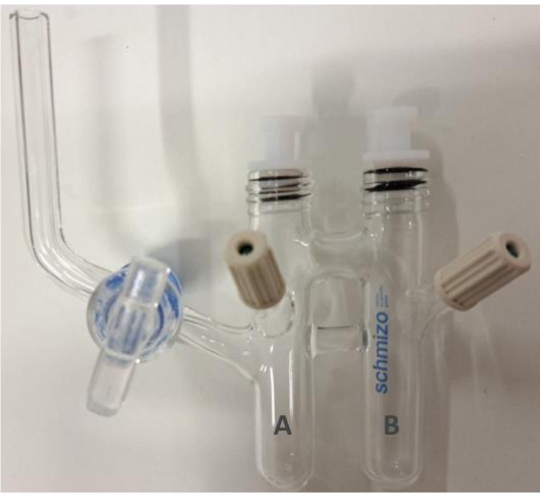

Figure S23. Custom-made two-chamber system with side-arm, valve, as well as small septa for addition of reagents/solvents, and H-caps (as well as screw caps, not shown); developed for ^{14}C-, ^{2}H, or ^{3}H-labeling. First, the chamber is evacuated and refilled with nitrogen (3x) through the side arm, with a final evacuation. **Procedure for ^{2}H or ^{3}H-labeling:** a) Addition of D_2-gas/T_2-gas from deuterium/tritium manifold via side arm, then closing of the valve, and detachment of the manifold. b) Addition of reaction mixture to Chamber A, stirring of Chamber A at 80 °C for 1 h to allow CO evolution from ^{12}COgen. c) Addition of Rh/ligand-catalyst solution to Chamber B. d) Shaking of reaction mixture. **Procedure for ^{14}C-labeling:** a) Addition of reaction mixtures to Chamber A, 80 °C, 1 h. b) Addition of hydrogen gas with syringe via small septa. c) Addition of Rh/ligand-catalyst solution to Chamber B.
d) Shaking of reaction mixture.

6.1 General Procedure GP4: [^{14}C]Hydroformylation with ^{14}COgen

When performing hydroformylation reactions for ^{14}C-labeling, ^{14}COgen was used because synthesis of Sila^{14}COgen has not yet been achieved. The addition of reagents was slightly different compared to ^{3}H-labeling.

<u>Synthesis of ^{12}COgen and ^{14}COgen mixture (95:5):</u>

^{14}COgen was prepared according to the procedure of Skrydstrup and coworkers.[3, 4]

9-methyl-9H-fluorene-9-carboxylic-^{14}C-acid (calculated mass 1.13 mg, 0.005 mmol, 110 µL of a 99.4 MBq/mL, 2.19 TBq/mol solution in toluene, 0.05 equiv., 10.95 MBq) and 9-methyl-9H-fluorene-9-carboxylic-^{12}C acid (21 mg, 0.095 mmol, 0.95 equiv.) was loaded in a 10 mL round-bottom flask. The solvent was removed under reduced pressure and redissolved in CH_2Cl_2

(1.5 mL) under a nitrogen atmosphere. To this solution was added oxalyl dichloride (26 µL, 0.3 mmol, 3 equiv.), followed by DMF (12 drops of a 1 drop/mL solution in CH_2Cl_2). The reaction started to bubble, and was stirred for three hours at RT. The solvent was removed *in vacuo*, and the product was redissolved in diglyme (0.5 mL).

<u>Hydroformylation:</u>
The exact reaction scale and changes in the procedure are detailed in the experimental procedure of the respective compounds. The handling of the two-chamber for the ^{14}C-labeling experimental setup contained the following steps after evacuating the system:
For **^{14}C-labeling**: In theory, the side arm of the modified two-chamber system would not be necessary for ^{14}C-labeling because hydrogen gas was not added *via* the side arm but was instead injected with a syringe. Therefore, a standard two-chamber system could be used. However, the modified two-chamber system has the advantage that the reactor can be easily evacuated through the side-arm, allowing a reaction under sub atmospheric pressure. This could be advantageous for CO solubilization in the solvent as well as to reduce the risk of a leaky system when working under pressure. Further, the small septa allow the addition of solvents with a syringe which is not possible with the standard two-chamber system with H-caps that does not contain these septa. As a side note (and in contrast to deuterium/tritium labeling for some peptides), a 1:1 mixture of (^{12}CO:^{14}CO, 95:5):H_2 was used with the expensive ^{14}COgen. Also, the hydrogen gas was added with a syringe *after* the carbon monoxide release to ensure no (potential) hydrogen-gas mediated inhibition of the Pd-catalyzed CO-release.

1. A solution containing $Rh(COD)_2BF_4$ (7.5 mg, 18.5 µmol, 0.92 equiv.) and 6-DPPon (27 mg, 97 µmol, 4.8 equiv.) in DMSO (or DMSO:H_2O, 95:5) (2 mL) was prepared.
2. The hydroformylation chamber (chamber B) of a custom-made two-chamber system was prepared with the resin-bound substrate **SM** (ca. 20 µmol, see exact amount in respective individual procedure).
3. To the CO-producing chamber (chamber A) was added $Pd(dba)_2$ (5.8 mg, 10 µmol), and a stirring bar.
4. The two-chamber system (20 mL) was evacuated through the side arm. Then, a degassed solution of tri-*tert*-butylphosphine (10 µL, 10 µmol, 1M in toluene) and DIPEA (52 µL, 0.3 mmol) in diglyme (0.5 mL) was added to Chamber A, followed by a solution of ^{12}COgen:^{14}COgen (95:5, 24 mg, 0.1 mmol, 5 equiv.) in diglyme (0.5 mL). Chamber A was stirred at 80 °C for 1 hour.
5. After allowing the reactor to cool down to RT, hydrogen gas (2.5 mL, 0.1 mmol, 5 equiv.) was added *via* a syringe. The system was allowed to equilibrate for 2–3 minutes.
6. Then, a solution of $Rh(COD)_2BF_4$ (7.5 mg) and 6-DPPon (27 mg) in DMSO (or DMSO/H_2O, 95:5) (2 mL) was added to Chamber B. The septa were parafilmed, and the two-chamber system was shaken at RT (or 40 °C) for 60 h.
7. After this time, the two-chamber was first carefully evacuated *via* the side-arm to remove radioactive gases, and then the reactor was opened. The reaction mixture was filtered over a peptide reactor and washed with 3x CH_2Cl_2 and 3x MeOH. The peptide was then subjected to the reductive amination procedure, typically GP6A.

6.2 General Procedure GP5: [^{2}H or ^{3}H]Hydroformylation with deuterium- or tritium-Gas

When performing hydroformylation reactions for ^{2}H or ^{3}H-labeling, ^{12}COgen was used instead of the more easy-to-handle SilaCOgen. This was mainly because during the KF-mediated

release of CO from SilaCOgen[10] gaseous hydrogen fluoride is formed which may lead to labeling dilution of the deuterium/tritium isotope.
In tritium chemistry, there is a main reservoir of tritium gas stored on uranium that has nearly pure ^3H. There is a second uranium bed reservoir that recovers excess tritium gas after reactions to prevent its release into the atmosphere. This reservoir is often contaminated with protons from previous reactions and the ratio of tritium gas to hydrogen gas is lower. To make our chemistry greener, the second (waste) bed of tritium gas was used for this proof-of-concept study of the methodology, allowing to conserve our main tritium supply and to use what is generally just a waste product. The obtained lower tritium incorporations in the reactions (<2 ^3H/molecule) may be in part explained by using this waste reservoir and by the presence of water traces during the reaction, or potential hydrogen isotope dilution from 1,5-COD isomerization of the pre-catalyst. For tritium-labeling, the tritium gas was not recovered after the hydroformylation reaction, which we usually do in our laboratory for standard tritium chemistry. This was mainly due to concerns of contaminating the tritium uranium bed with carbon monoxide. Also, the reaction scale was reduced for tritium chemistry.
For both deuterium and tritium labeling, typically a 1:1 or 2:1 CO:($^2H_2$ or $^3H_2$)-mixture was used, the latter because of concerns of slower CO formation in presence of $^2H_2$ or $^3H_2$ gas. Importantly, in case the carbon monoxide was not formed completely, reduction or isomerization of the double bond rather than productive hydroformylation was observed. Therefore, complete formation of carbon monoxide is important, and we advise the use of a 2:1 mixture of CO:H_2 with the inexpensive ^{12}COgen for ^2H or ^3H-labeling-applications. The exact reaction scale and changes in the procedure are highlighted in the experimental procedure of the respective compounds.

1. A solution containing $Rh(COD)_2BF_4$ (2.8 mg, 6.9 µmol, 1.6 equiv.) and 6-DPPon (10 mg, 36 µmol, 8.1 equiv.) (or BIPHEPHOS) in solvent DMSO (1 mL) (or DMSO:THF, 17:3 for BIPHEPHOS) was prepared.
2. The hydroformylation chamber of a custom-made two-chamber system (chamber B) was prepared with the resin-bound substrate **SM** (ca. 4.4 µmol, see exact amount in respective individual procedure).
3. To the CO-producing chamber (chamber A) was added COgen (97 mg, 0.4 mmol, if not elsewise stated), $Pd(dba)_2$ (11.4 mg, 20 µmol), and a stirring bar.
4. The two-chamber system (20 mL) was attached to the deuterium/tritium manifold, evacuated, and loaded with deuterium gas/tritium gas (ca. 280 mbar, ca. 0.2 mmol). The valve was closed, and the two-chamber system was detached from the manifold.
5. Then, a degassed mixture of DIPEA (0.105 mL, 0.6 mmol) and tri-*tert*-butylphosphine (20 µL, 20 µmol, 1M in toluene) in diglyme (2 mL) was added to chamber A *via* the small septum. Chamber A was then stirred at 80 °C for 1 h to allow complete carbon monoxide formation.
8. After allowing the reactor to cool down to RT, $Rh(COD)_2BF_4$ (2.8 mg) and 6-DPPon (10 mg) (or BIPHEPHOS) dissolved in DMSO (1 mL) was added to the substrate in chamber B *via* the septa.
8. The septa were parafilmed and the reactor was then shaken at 40 °C–60 °C (depending on the substrate) for 60 h.
9. After this time, MeOH (ca. 1 mL) was added chambers via the small septa to allow hydrogen exchange of exchangeable to both tritium atoms. MeOH/MeOT was then carefully removed under a stream of nitrogen. This process was performed in total three times. Then, the reaction mixture was filtered over a peptide reactor and washed with

3x CH_2Cl_2 and 3x MeOH. The peptide was then subjected to the reductive amination procedure, typically GP6A.

7. General Procedure GP6 for reductive aminations

After hydroformylation of peptides, usually a reductive amination on resin-bound peptides was performed. The amines used were either tritylamine or benzylamine. For benzylamine, CH_2Cl_2 could be used as the solvent for iminium ion formation. For the iminium ion formation with tritylamine, DMSO had to be used as solvent due to poor solubility of tritylamine in CH_2Cl_2. Reductive amination was performed either according to GP6A, GP6B, or GP6C. The difference between GP6A and GP6B is only the amount of AcOH; it was found that increasing the amount of AcOH to 10% led to higher conversion of the aldehyde when using tritylamine, and we did not, for the employed sequences, observe side reactions from too high acid amount at these concentrations.
After reductive amination, the reaction mixture was filtered over a peptide reactor and washed with 3x CH_2Cl_2 and 3x MeOH. The peptide was then cleaved typically according to GP2B.

7.1 GP6A: With trityl amine, 1% AcOH

A solution of tritylamine in DMSO, 1% acetic acid (2–5 mL, 31 mg/mL, 0.12 mmol/mL) was added to the resin, and the reaction mixture was shaken for 2 hours. Then, a solution of $NaCNBH_3$ in CH_2Cl_2/MeOH, 3:1 (2–5 mL, 10 mg/mL, 0.15 mmol/mL) was added to the reaction mixture and shaken for an additional 1 hour.
Please see the procedure of the respective compounds for exact amounts of reagents.

7.2 GP6B: With trityl amine, 10% AcOH

A solution of tritylamine in DMSO, 10% acetic acid (5 mL, 31 mg/mL, 0.12 mmol/mL) was added to the resin, and the reaction mixture was shaken for 2 hours. Then, a solution of $NaCNBH_3$ in CH_2Cl_2/MeOH, 3:1 (5 mL, 10 mg/mL, 0.15 mmol/mL) was added to the reaction mixture and shaken for an additional 1 hour.

7.3 GP6C: With benzylamine, 1% AcOH

(mainly performed for the optimization series and not for final molecules)
A solution of benzylamine in CH_2Cl_2, 1% acetic acid (0.5–6 mL, 13 µL/mL, 0.12 mmol/mL) was added to the resin, and the reaction mixture was shaken for 2 hours. Then, a solution of $NaCNBH_3$ in CH_2Cl_2/MeOH, 3:1 (0.5–6 mL, 10 mg/mL, 0.15 mmol/mL) was added to the reaction mixture and shaken for an additional 1 hour.

Note: For the synthesis of **12Cb**, we used benzylamine in DMF for the imine formation; however typically (as performed during the optimization) we employed CH_2Cl_2. If not degassed, DMF can contain small amounts of dimethylamine which can also undergo reductive amination, leading to minor side product formation.

8. Peptide (Allylglycine)-precursor syntheses

8.1 General Procedure GP7 for loading determination of the allylglycine-containing peptide on-resin

The resin-bound peptide (ca. 70 mg) was added to a 2 mL peptide reactor and cleaved, typically according to GP2B. The peptide was analyzed by LC-MS and qNMR analysis was conducted using 1,3,5-trimethoxybenzene (1,3,5-TMB) as the internal standard.

8.1.1 SI-1, SI-2, SI-3

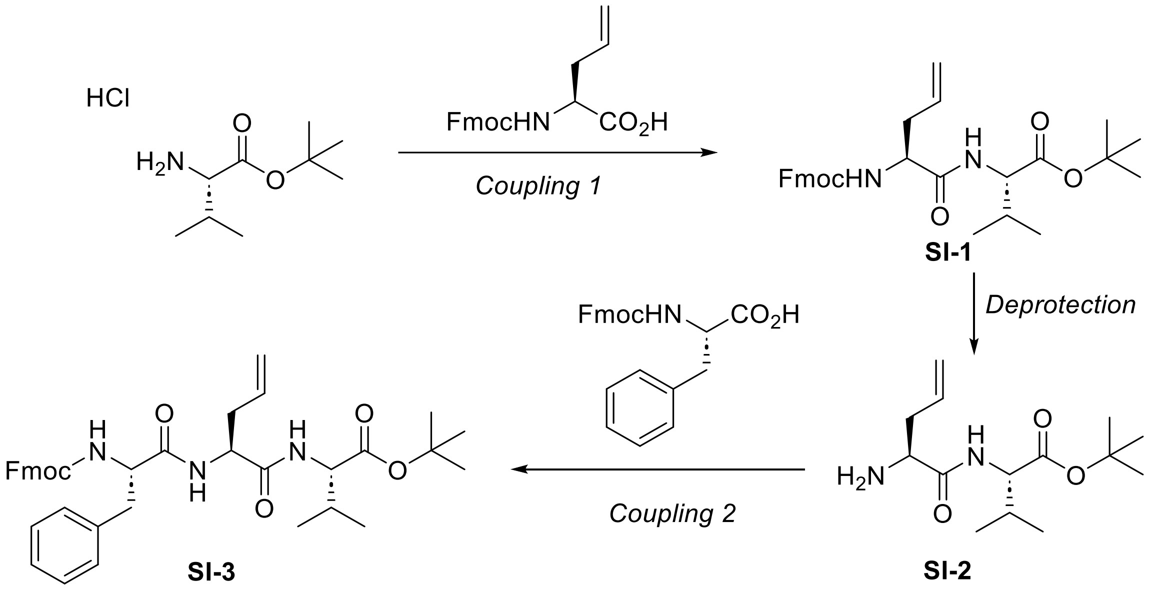

Chemical Formula: $C_{38}H_{45}N_3O_6$
Exact Mass: 639.3308

Sequence: FmocHN – Phe –GAll – Val – CO2*t*Bu

Coupling 1:

A solution of (*S*)-2-((((9H-fluoren-9-yl)methoxy)carbonyl)amino)pent-4-enoic acid (531 mg, 1.57 mmol, 1.1 equiv.), DIPEA (0.249 mL, 1.43 mmol, 1 equiv.) and PyBOP (819 mg, 1.57 mmol, 1.1 equiv.) was prepared in CH_2Cl_2 (3 mL). To this solution was added *tert*-butyl-*L*-valinate hydrochloride (300 mg, 1.43 mmol, 1 equiv.) dissolved in CH_2Cl_2 (3 mL) at 0 °C. The solution was stirred at RT for 17 h. Then, sat. aq. NH_4Cl (20 mL) and water (20 mL) was added. The aqueous layer was extracted with CH_2Cl_2 (3x 30 mL). The combined organic phases were washed with water (100 mL) and brine (100 mL) and concentrated *in vacuo*. The crude was purified by silica column chromatography (EtOAc in heptane, 5-50 %) to obtain product **SI-1** as a white solid (530 mg, 1.076 mmol, 75%).

Deprotection:

A round-bottomed flask was charged with compound **SI-1** (500 mg, 1.01 mmol, 1 equiv.) and prepared at 0 °C. Then, 20% piperidine in CH_2Cl_2 (15 mL) was added and the solution was stirred for 1.5 hours at RT. The solvent was removed *in vacuo* to obtain the crude without further purification (530 mg crude, product **SI-2** (ca. 273 mg, 1.01 mmol), and by-product 1-((9H-fluoren-9-yl)methyl)piperidine (ca. 266 mg, 1.01 mmol)).

Coupling 2:

The crude of the deprotection step containing **SI-2** (0.273 g, 1.01 mmol) was prepared together with DIPEA (0.387 mL, 2.22 mmol, 2.2 equiv.) in CH_2Cl_2 (3 mL). A solution of (((9H-fluoren-

9-yl)methoxy)carbonyl)-*L*-phenylalanine (0.509 g, 1.31 mmol, 1.3 equiv.), DIPEA (0.229 mL, 1.31 mmol, 1.3 equiv.) and PyBOP (0.720 g, 1.38 mmol, 1.4 equiv.) in CH_2Cl_2 (3 mL) was added to this solution dropwise at 0 °C. This solution was stirred at RT for 1 h, after which an additional amount of (((9H-fluoren-9-yl)methoxy)carbonyl)-*L*-phenylalanine (0.257 g, 0.65 mmol, 0.65 equiv.), DIPEA (0.115 mL, 0.65 mmol, 0.65 equiv.) and PyBOP (0.360 g, 0.69 mmol, 0.7 equiv.) in CH_2Cl_2 (1.5 mL) was added (because the activated ester seemed to react with left-over piperidine from the crude). Then, the solution was stirred for 17 hours at RT, followed by addition of sat. aq. NH_4Cl (70 mL) and water (70 mL). The aqueous layer was extracted with CH_2Cl_2 (3x 30 mL). The combined organic phases were washed with water (2x 100 mL) and brine (100 mL) and concentrated *in vacuo*. The crude was purified by silica column chromatography (EtOAc in heptane, 10–20%) to obtain product **SI-3** as a white solid (408 mg, 0.64 mmol, 63%).

^{1}H NMR (500 MHz, DMSO, 25°C) δ 8.07 (t, *J* = 7.5 Hz, 2H), 7.87 (d, *J* = 7.6 Hz, 2H), 7.64 (s, 1H), 7.58–7.62 (m, 2H), 7.37–7.44 (m, 2H), 7.27–7.33 (m, 4H), 7.24 (t, *J* = 7.6 Hz, 2H), 7.17 (t, *J* = 7.3 Hz, 1H), 5.74 (dt, *J* = 16.9, 8.4 Hz, 1H), 5.08 (d, *J* = 17.1 Hz, 1H), 5.00 (d, *J* = 10.3 Hz, 1H), 4.49 (q, *J* = 7.4 Hz, 1H), 4.23–4.32 (m, 1H), 4.08–4.21 (m, 3H), 4.04 (t, *J* = 7.2 Hz, 1H), 3.00 (dd, *J* = 14.0, 3.7 Hz, 1H), 2.72–2.8 (m, 1H), 2.45 (q, *J* = 6.7 Hz, 1H), 2.33 (dt, *J* = 14.7, 7.6 Hz, 1H), 2.02 (h, *J* = 6.8 Hz, 1H), 1.40 (s, 9H), 0.88 (t, *J* = 6.3 Hz, 6H).

^{13}C NMR (126 MHz, DMSO) δ 171.1, 170.8, 170.2, 155.6, 143.6, 140.5, 138.0, 133.8, 129.0, 127.8, 127.4, 126.9, 126.0, 125.2, 125.1, 119.9, 117.3, 80.4, 65.5, 57.8, 55.9, 51.6, 46.3, 37.2, 36.4, 29.8, 27.4, 18.7, 17.9.

HRMS (ESI+) (m/z): $[M+H]^+$ calculated for $C_{38}H_{46}N_3O_6^+$ 640.3381; found 640.3389.

8.1.2 SI-4 and SI-4-cleav

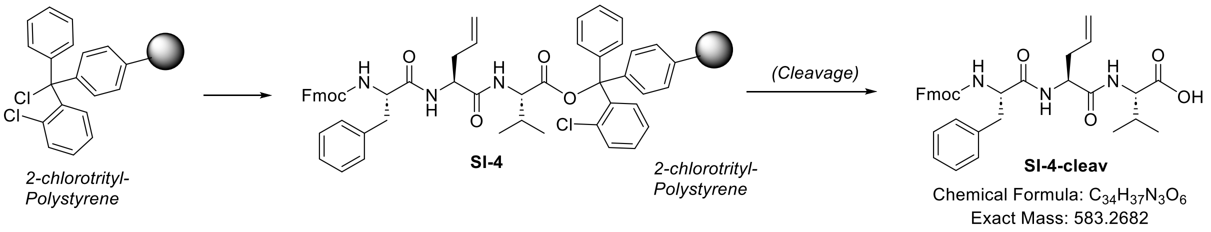

Sequence SI-4: FmocHN – Phe – Gall – Val – chlorotrityl-linker-polystyrene resin
Sequence SI-4-cleav: FmocHN – Phe – Gall – Val – CO_2H

The title compound was prepared according to General Procedure GP1A on a 3 mmol scale using 2-chlorotrityl-linker-polystyrene-resin (3.990 g, 5.7 mmol, 1.9 equiv., ca. 1.43 mmol/g loading). Coupling 1 was run with (((9H-fluoren-9-yl)methoxy)carbonyl)-L-valine (1.018 g, 3 mmol, 1 equiv.), followed by capping. Coupling 2 was carried out with (S)-2-((((9H-fluoren-9-yl)methoxy)carbonyl)amino)pent-4-enoic acid (1.012 g, 3 mmol, 1 equiv.), followed by capping. Coupling 3 was performed with (((9H-fluoren-9-yl)methoxy)carbonyl)-L-phenylalanine (2.324 g, 6 mmol, 2 equiv.). Cleavage of the peptide according to GP2C yielded the title compound **SI-4-cleav** as a white solid (1.55 g, 2.66 mmol, 89%).
The synthesis was repeated (with minor modifications) to obtain **SI-4** on-resin (not cleaved).

^{1}H NMR (500 MHz, DMSO) δ = 12.6 (s, 1H), 8.1 (d, *J*=8.0, 1H), 8.0 (d, *J*=8.5, 1H), 7.9 (d, *J*=7.6, 2H), 7.6 – 7.7 (m, 3H), 7.4 (td, *J*=7.4, 3.3, 2H), 7.2 – 7.4 (m, 6H), 7.2 (t, *J*=7.3, 1H), 5.7 (ddt, *J*=17.1, 10.3, 6.9, 1H), 5.1 (dd, *J*=17.2, 2.0, 1H), 5.0 (dd, *J*=10.2, 2.1, 1H), 4.5 (td, *J*=8.1,

5.2, 1H), 4.3 (ddd, J=12.1, 8.8, 3.7, 1H), 4.1 (tdd, J=13.2, 11.1, 8.5, 4H), 3.0 (dd, J=13.8, 3.7, 1H), 2.8 (dd, J=13.8, 10.9, 1H), 2.5 (dt, J=12.9, 5.8, 1H), 2.3 (dt, J=14.5, 7.7, 1H), 2.1 (dq, J=13.4, 6.7, 1H), 0.9 (dd, J=6.9, 2.3, 6H).

^{13}C NMR (126 MHz, DMSO) δ = 172.8, 171.4 , 171.0 , 155.7, 143.7 (d, J=3.1), 140.6 (d, J=2.3), 138.2, 134.0, 129.2, 128.0, 127.6, 127.0, 126.2, 125.3 (d, J=11.0), 120.1, 117.5, 65.6, 57.1, 56.0, 51.9, 46.5, 37.4, 36.4, 29.9, 19.1, 17.9.

HRMS (ESI+) (m/z): $[M+H]^+$ calculated for $C_{34}H_{38}N_3O_6^+$ 584.2755; found 584.2772.

8.1.3 SI-5 and SI-6 *L*-allylglycine (Fmoc, dmb)

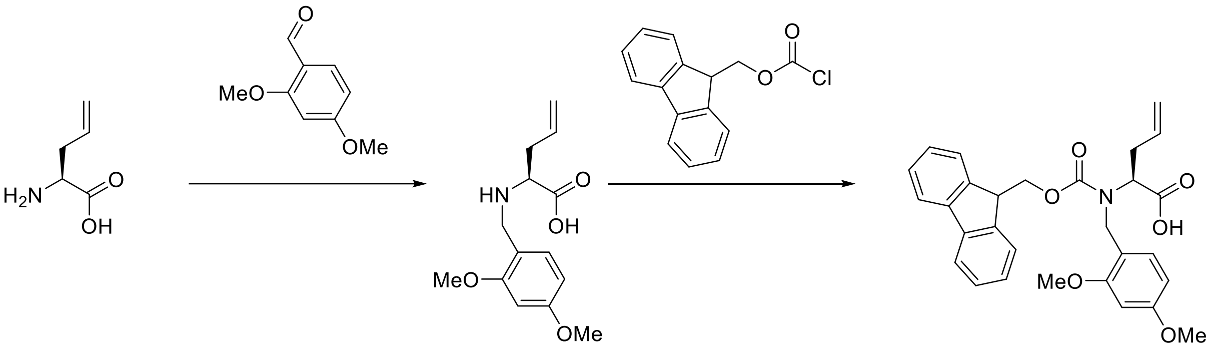

SI-5

SI-6

Chemical Formula: $C_{29}H_{29}NO_6$
Exact Mass: 487.1995

The synthesis of the special amino acid *L*-allylglycine(Fmoc, dmb) **SI-6** was inspired by the synthesis developed by Albericio and co-workers for the synthesis of hmb-protected amino acids.[11]

A solution of (*S*)-2-aminopent-4-enoic acid (576 mg, 5 mmol, 1 equiv.) and 2,4-dimethoxybenzaldehyde (831 mg, 5 mmol, 1 equiv.) in H_2O:MeOH (2:1, 54 mL) was stirred at RT for 2 h. Then, sodium cyanoborohydride (628 mg, 10 mmol, 2 equiv.) was added and the reaction mixture was stirred at RT. After 24 h, AcOH (100 µL) was added, and the solution was stirred for further 16 h. Then, the solvent was evaporated to remove MeOH. The aqueous solution was passed through a C18 (EC) column. The aqueous solution was lyophilized to obtain a solid crude. The solid was re-dissolved in MeOH and CH_2Cl_2 and passed through an Isolute® filter. The solvent was removed *in vacuo* to obtain *L*-allyl-glycine(dmb) **SI-5**. The crude product was used without further purification in the next step.

SI-5 was dissolved in CH_2Cl_2 (50 mL) under nitrogen. Then, DIPEA (5.23 mL, 30 mmol, 6 equiv.) and trimethylsilyl chloride (3.81 mL, 30 mmol, 6 equiv.) was added. The suspension was stirred at RT for 10 min. The solution was cooled to -78 °C, followed by a dropwise addition of (9H-fluoren-9-yl)methyl carbonochloridate (1.294 g, 5 mmol, 1 equiv.) in CH_2Cl_2 (20 mL) over 10 min. The solution was stirred at -78 °C for 20 min, and at RT for additional 90 min. The reaction mixture was concentrated *in vacuo*. H_2O (100 mL) was added to the compound dissolved in CH_2Cl_2 (ca. 5 mL, leading to a pH of ca. 3). The compound was extracted with CH_2Cl_2 (2 x 50 mL). The combined organic phases were passed through an Isolute® filter. The crude was purified by preparative HPLC (gradient: 60% B for 3 min, 60–85% B for 10 min) to afford the desired compound **SI-6** (971 mg, 2 mmol, 40% over two steps, ca. 83% purity by qNMR) as a white solid.

SI-5: crude, baseline correction not complete, therefore too many protons integrated, ^{1}H-NMR spectrum attached but not reported.

SI-6: Presence of rotamers

^{1}H NMR (600 MHz, DMSO) δ = 12.62 (bs, 1H), 7.86 (t, *J*=8.7, 2H), 7.66 (t, *J*=7.5, 0.7H), 7.45 – 7.54 (m, 1.3H), 7.36 – 7.44 (m, 2H), 7.32 (t, *J*=7.5, 0.7H), 7.2 – 7.29 (m, 1.3H), 7.02 (d, *J*=8.4, 0.3H), 6.87 (d, *J*=8.4, 0.6H), 6.48 – 6.53 (m, 1H), 6.42 (d, *J*=8.4, 0.3H), 6.35 (dd, *J*=8.3, 2.4, 0.6H), 5.51 – 5.65 (m, 0.7H), 5.39 (dq, *J*=16.8, 7.1, 0.3H), 4.89 (d, *J*=11.8, 1.3H), 4.83 (d, *J*=10.2, 0.3H), 4.73 (d, *J*=17.2, 0.3H), 4.3 – 4.51 (m, 2H), 4.1 – 4.3 (m, 4H), 3.74 (d, *J*=2.8, 6H), 2.57 (dt, *J*=12.9, 6.0, 0.7H), 2.36 – 2.48 (m, 1H), 2.09 – 2.18 (m, 0.3H).

^{13}C NMR (151 MHz, DMSO) δ = 171.9, 159.7 (d, *J*=11.6), 157.5 (d, *J*=18.1), 155.4, 143.8 (d, *J*=6.3), 140.8, 135.2, 129.6 (d, *J*=17.3), 127.6 (d, *J*=3.9), 127.0 (d, *J*=3.6), 125.0 (d, *J*=5.3), 120.1 (d, *J*=4.1), 117.8, 117.0, 104.3, 97.9, 66.6 (d, *J*=9.8), 60.1, 55.3, 55.2, 46.7, 45.7, 33.4.

→23 ^{13}C-signals reported, however there are some additional signals in the spectrum that were not reported (complex due to presence of rotamers).

HRMS (ESI+) (m/z): $[M+Na]^{+}$ calculated for $C_{29}H_{29}NNaO_6^{+}$ 510.1893; found 510.1902.

8.1.4 1SMa

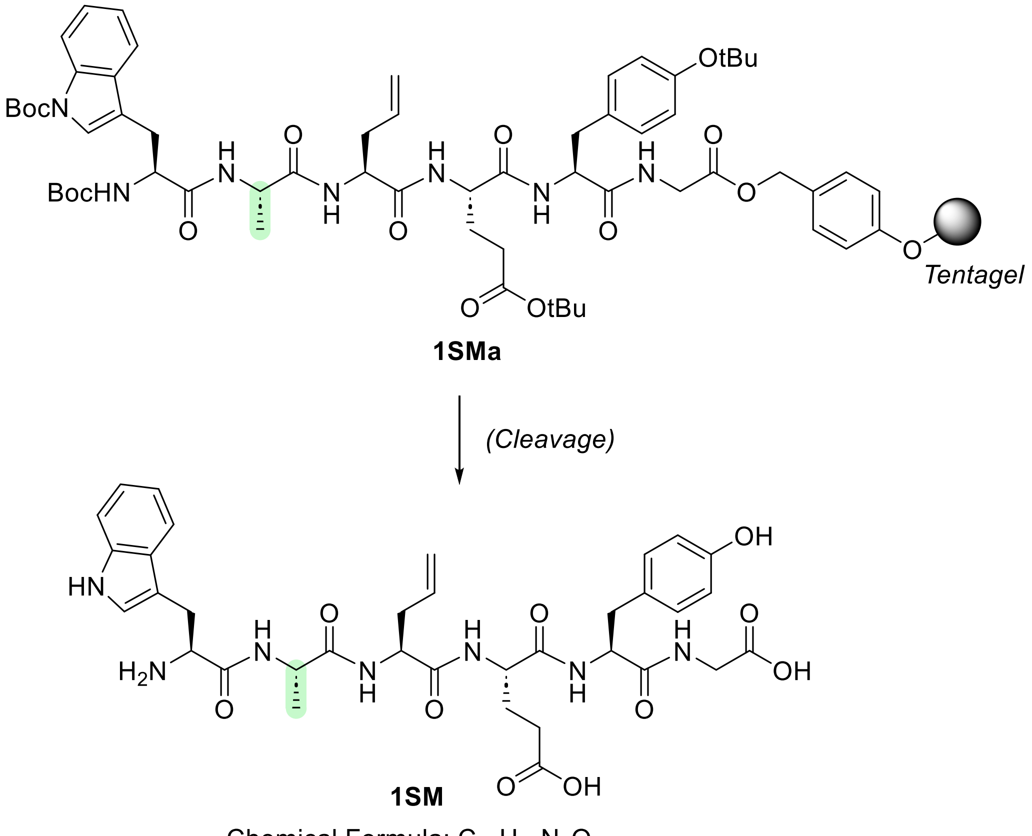

1SMa

1SM

Chemical Formula: $C_{35}H_{43}N_7O_{10}$

Exact Mass: 721.3071

Sequence: BocHN – Trp(Boc) – Ala – GAll – Glu(tBu) – Tyr(tBu) – Gly – Wanglinker – Tentagel resin

The title compound was prepared according to General Procedure GP1B on a 0.3 mmol scale using FmocHN-Gly-Wanglinker-Tentagel-resin (1.304 g, 0.3 mmol, 0.23 mmol/g loading).

Test cleavage of the peptide on-resin according to GP7/GP2B yielded the title compound as a white solid and allowed loading determination by qNMR (1.452 g resin, 0.25 mmol, 0.15 mmol/g loading determined by weight difference, 0.17 mmol/g loading determined by qNMR, 82%, ca. 98% UV-purity).

NMR details: spectra attached, not in detail reported.
HRMS (ESI+) (m/z): $[M+H]^+$ calculated for $C_{35}H_{44}N_7O_{10}^+$ 722.3144; found 722.3170.

8.1.5 1SMb

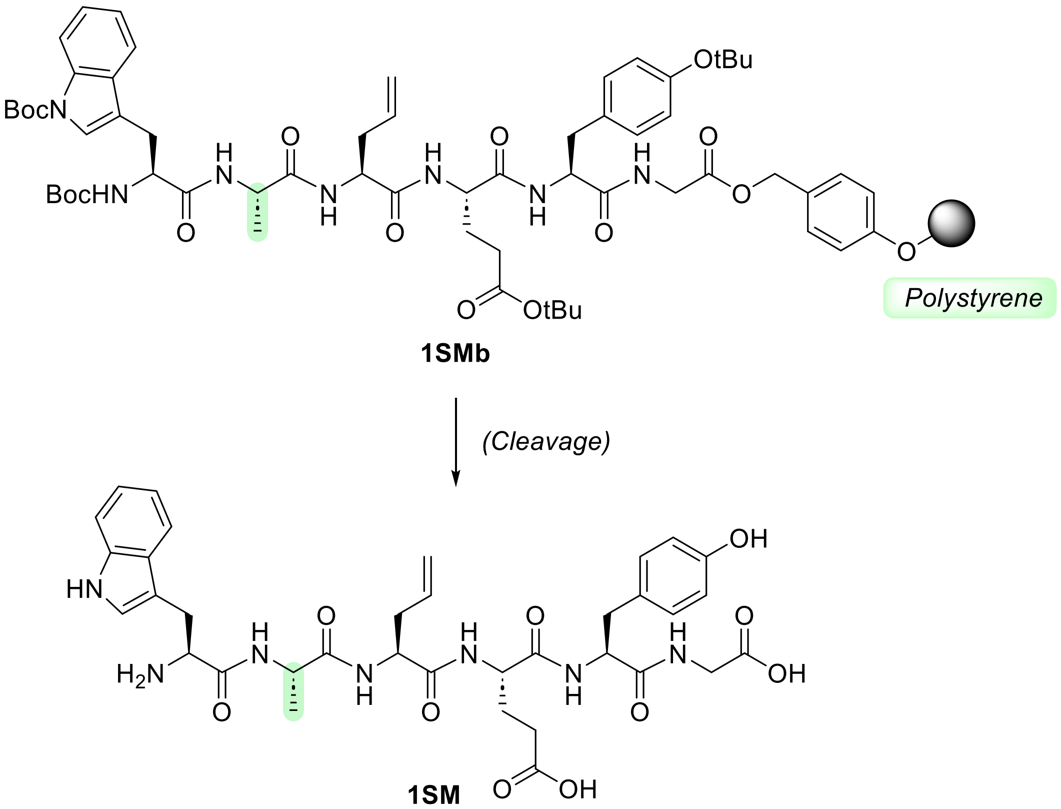

1SM

Chemical Formula: $C_{35}H_{43}N_7O_{10}$
Exact Mass: 721.3071

Sequence: BocHN – Trp(Boc) – Ala – GAll – Glu(tBu) – Tyr(tBu) – Gly – Wanglinker – Polystyrene-resin

The title compound was prepared according to General Procedure GP1B on a 0.3 mmol scale using FmocHN-Gly-Wanglinker-polystyrene-resin (0.769 g, 0.3 mmol, 0.39 mmol/g loading). Test cleavage of the peptide on-resin according to GP7/GP2B yielded the title compound as a white solid and allowed loading determination by qNMR (1.086 g resin, 0.28 mmol, 0.26 mmol/g loading determined by qNMR, 94%).

NMR/LC-MS details: see **1SMa**

8.1.6 2SM

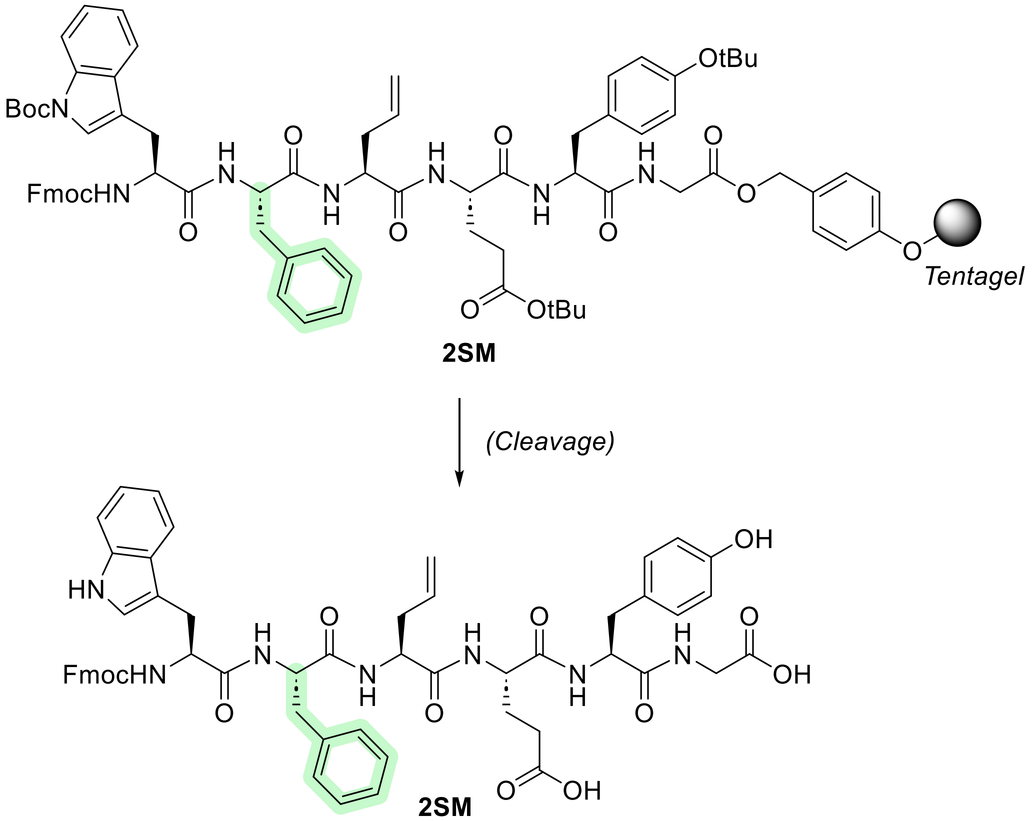

2SM

Chemical Formula: $C_{56}H_{57}N_{7}O_{12}$

Exact Mass: 1019.4065

Sequence: FmocHN – Trp(Boc) – Phe – GAll – Glu(tBu) – Tyr(tBu) – Gly – Wanglinker – Tentagel-resin

The title compound was prepared according to General Procedure GP1B on a 0.276 mmol scale using FmocHN-Gly-Wanglinker-Tentagel-resin (1.2 g, 0.276 mmol, 0.23 mmol/g loading). Test cleavage of the peptide on-resin according to GP7/GP2B yielded the title compound as a white solid and allowed loading determination by qNMR (1.408 g resin, 0.21 mmol, 0.168 mmol/g loading determined by weight difference, 0.146 mmol/g loading determined by qNMR, 74%, ca. 99% UV-purity).

The peptide was prepared in several batches for optimization, leading to slight changes in loading.

NMR details: spectra attached, not in detail reported.
HRMS (ESI+) (m/z): $[M+H]^+$ calculated for $C_{56}H_{58}N_{7}O_{12}^+$ 1020.4138; found 1020.4136.

8.1.7 3SM

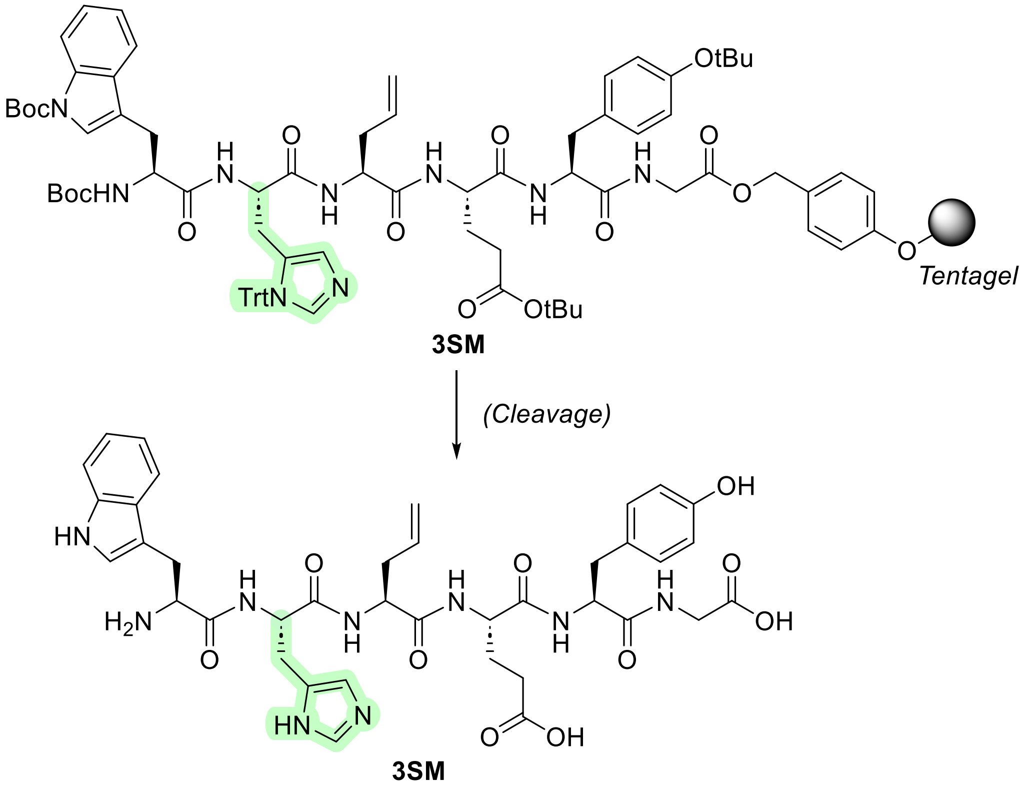

Chemical Formula: $C_{38}H_{45}N_9O_{10}$
Exact Mass: 787.3289

Sequence: BocHN – Trp(Boc) – His(Trt) – GAll – Glu(tBu) – Tyr(tBu) – Gly – Wanglinker – Tentagel-resin

The title compound was prepared according to General Procedure GP1B on a 0.3 mmol scale using FmocHN-Gly-Wanglinker-Tentagel-resin (1.304 g, 0.3 mmol, 0.23 mmol/g loading). Test cleavage of the peptide on-resin according to GP7/GP2B yielded the title compound as a white solid and allowed loading determination by qNMR (1.522 g resin, 0.22 mmol, 0.145 mmol/g loading determined by weight difference, 0.129 mmol/g loading determined by qNMR, 74%, ca. 81% UV-purity).

NMR details: spectra attached, not in detail reported.
HRMS (ESI+) (m/z): $[M+H]^+$ calculated for $C_{38}H_{46}N_9O_{10}^+$ 788.3362, found 788.3370.

8.1.8 4SM

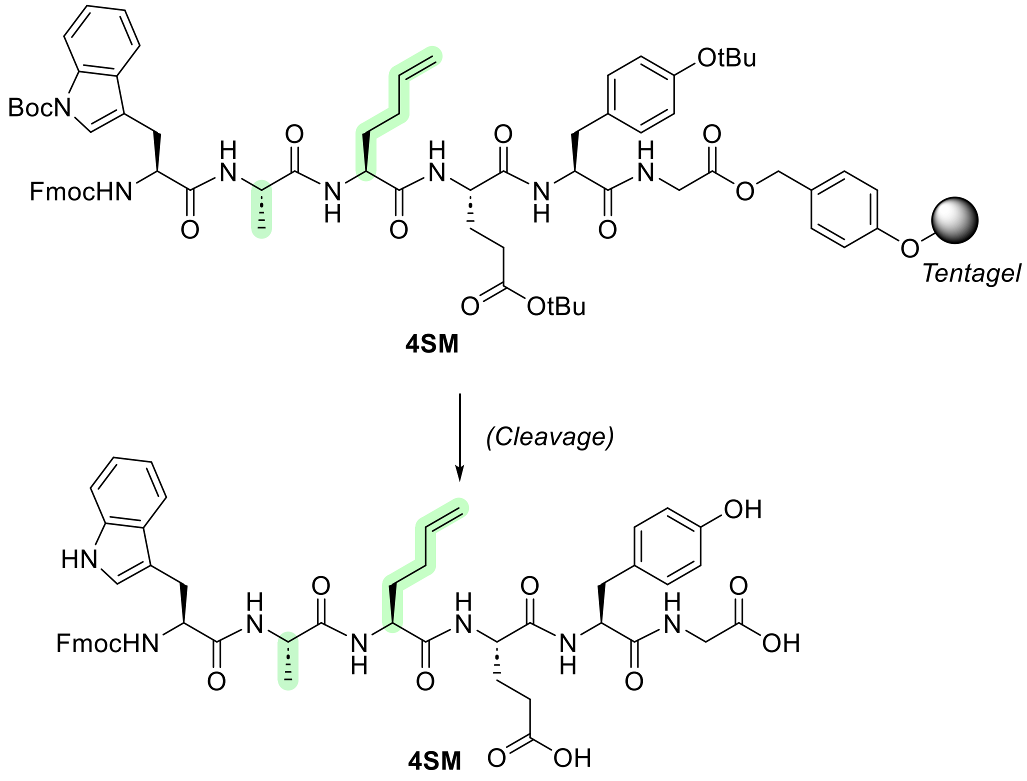

Chemical Formula: $C_{51}H_{55}N_7O_{12}$
Exact Mass: 957.3909

Sequence: FmocHN – Trp(Boc) – Ala – hGAll – Glu(tBu) – Tyr(tBu) – Gly – Wanglinker – Tentagel-resin
The title compound was prepared according to General Procedure GP1B on a 0.276 mmol scale using FmocHN-Gly-Wanglinker-Tentagel-resin (1.2 g, 0.276 mmol, 0.23 mmol/g loading). Test cleavage of the peptide on-resin according to GP7/GP2B yielded the title compound as a white solid and allowed loading determination by qNMR (1.373 g resin, 0.2 mmol, 0.15 mmol/g loading determined by weight difference, 0.114 mmol/g determined by qNMR, 75%, ca. 95% UV-purity).

NMR details: spectra attached, not in detail reported.
HRMS (ESI+) (m/z): $[M+H]^+$ calculated for $C_{51}H_{56}N_7O_{12}^+$ 958.3981; found 958.3997.

8.1.9 5SM

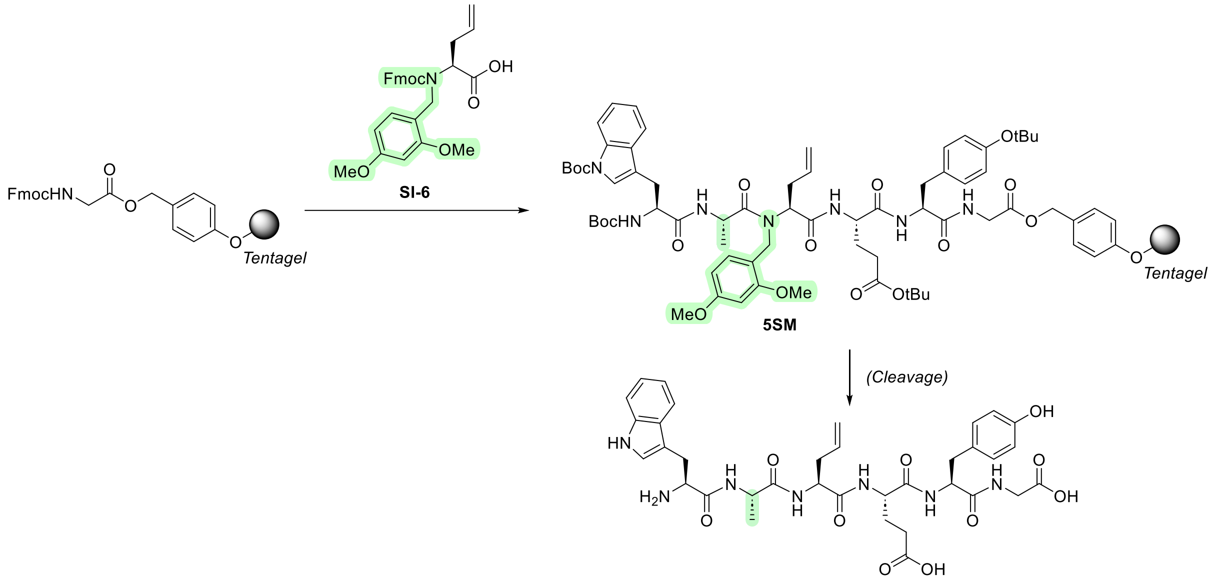

5SM (less pure than **1SM**)
Chemical Formula: $C_{35}H_{43}N_{7}O_{10}$
Exact Mass: 721.3071

Sequence: BocHN – Trp(Boc) – Ala – GAll(dmb) – Glu(tBu) – Tyr(tBu) – Gly – Wanglinker – Tentagel-resin

The title compound was prepared according to General Procedure GP1B on a 0.1 mmol scale using FmocHN-Gly-Wanglinker-Tentagel-resin (0.435 g, 0.1 mmol, 0.23 mmol/g loading). The SPPS was carried out in a 10 mL fritted plastic vial. After coupling of Glu(tBu), acetylation (capping) was performed after each further couling step. The coupling of the special amino acid *L*-allylglycine(Fmoc,dmb) **SI-6** was performed at RT for 20 min, followed by a rise to 75 °C for 5 min. The coupling of the amino acid Ala was repeated 4x before Fmoc-deprotection.

Test cleavage of the peptide on-resin according to GP7/GP2B yielded the title compound as a white solid and allowed loading determination by qNMR (0.448 g resin resin, 0.009 mmol, 0.031 mmol/g loading determined by weight difference, 0.021 mmol/g loading determined by qNMR, 9%, ca. 46% UV-purity).

NMR details: compare to **1SM** (spectra of **5SM** attached, but impure)
HRMS (ESI+): compare to **1SM**

8.1.10 6SM

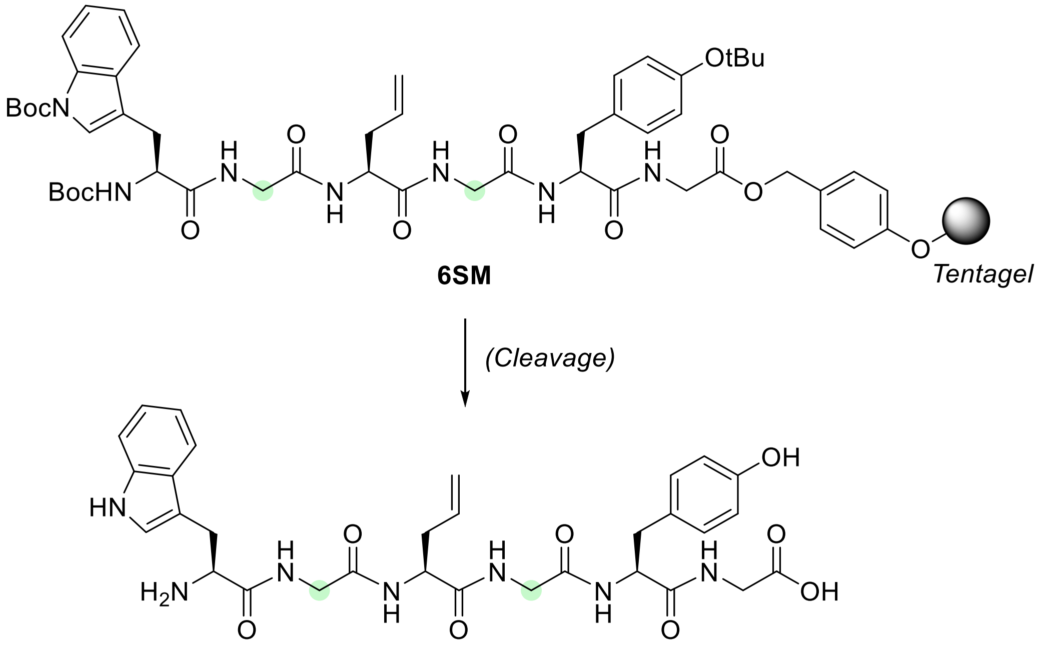

6SM

Chemical Formula: $C_{31}H_{37}N_7O_8$

Exact Mass: 635.2704

Sequence: BocHN – Trp(Boc) – Gly – GAll – Gly – Tyr(tBu) – Gly – Wanglinker – Tentagel-resin

The title compound was ordered on-resin. Test cleavage of the peptide on-resin according to GP7/GP2B yielded the title compound as a white solid and allowed loading determination by qNMR (0.214 mmol/g loading determined by qNMR, ca. 90% UV-purity).

NMR details: spectra attached, not in detail reported.

HRMS (ESI+) (m/z): $[M+H]^+$ calculated for $C_{31}H_{38}N_7O_8^+$ 636.2776; found 636.2791.

8.1.11 7SM

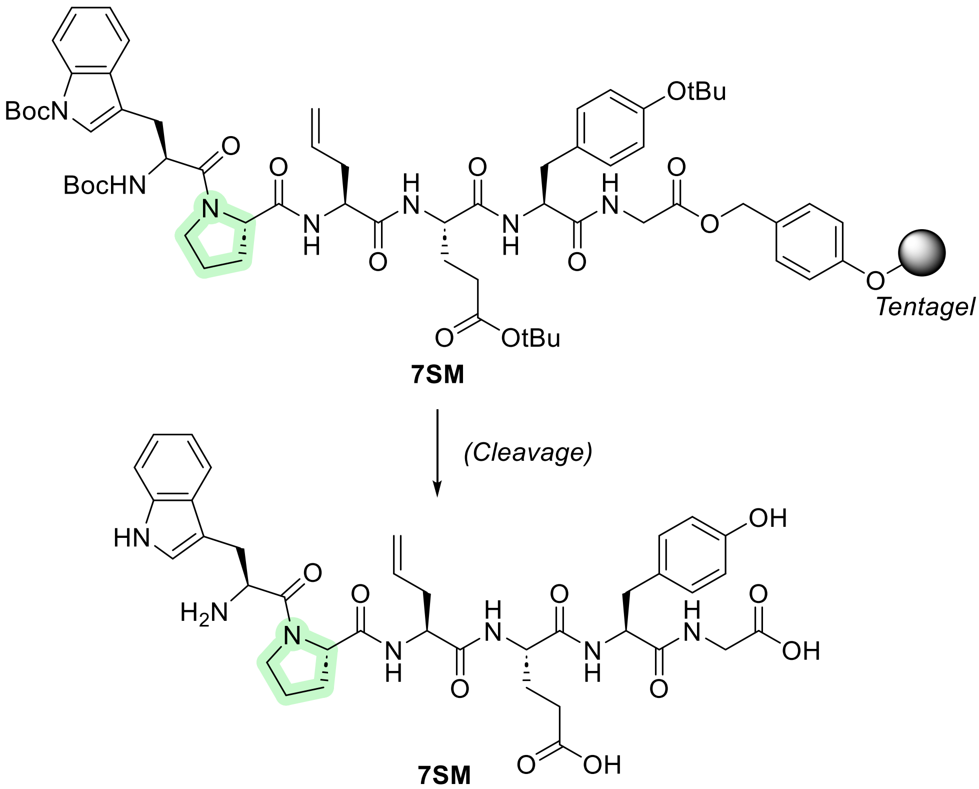

7SM

Chemical Formula: $C_{37}H_{45}N_7O_{10}$

Exact Mass: 747.3228

Sequence: BocHN – Trp(Boc) – Pro – GAll – Glu(tBu) – Tyr(tBu) – Gly – Wanglinker – Tentagel-resin

The title compound was ordered on-resin. Test cleavage of the peptide on-resin according to GP7/GP2B yielded the title compound as a white solid and allowed loading determination by qNMR (0.119 mmol/g loading determined by qNMR, ca. 95% UV-purity).

NMR details: spectra attached, not in detail reported.

HRMS (ESI+) (m/z): $[M+H]^+$ calculated for $C_{37}H_{46}N_7O_{10}^+$ 748.3301; found 748.3309.

8.1.12 8SM

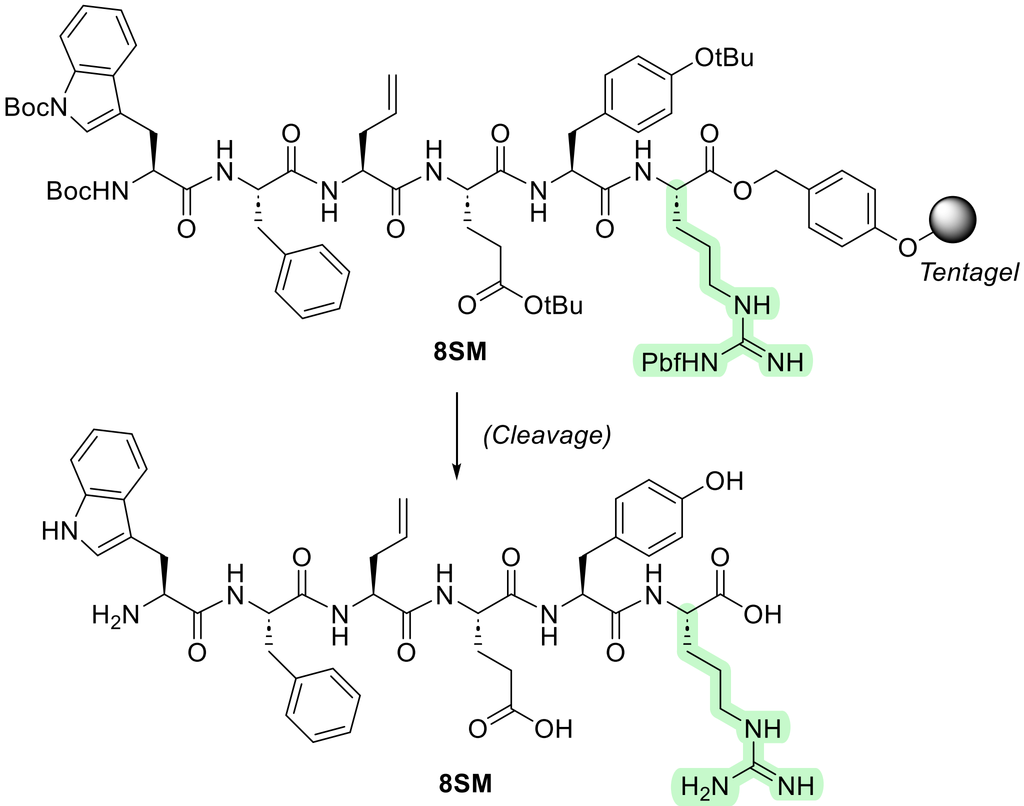

Chemical Formula: $C_{45}H_{56}N_{10}O_{10}$
Exact Mass: 896.4181

Sequence: BocHN – Trp(Boc) – Phe – GAll – Glu(tBu) – Tyr(tBu) – Arg(Pbf) – Wanglinker – Tentagel-resin

The title compound was ordered on-resin. Test cleavage of the peptide on-resin according to GP7/GP2B yielded the title compound as a white solid and allowed loading determination by qNMR (0.123 mmol/g loading determined by qNMR, ca. 97% UV-purity).

NMR details: spectra attached, not in detail reported.
HRMS (ESI+) (m/z): $[M+H]^+$ calculated for $C_{45}H_{57}N_{10}O_{10}^+$ 897.4254; found 897.4280.

8.1.13 9SM

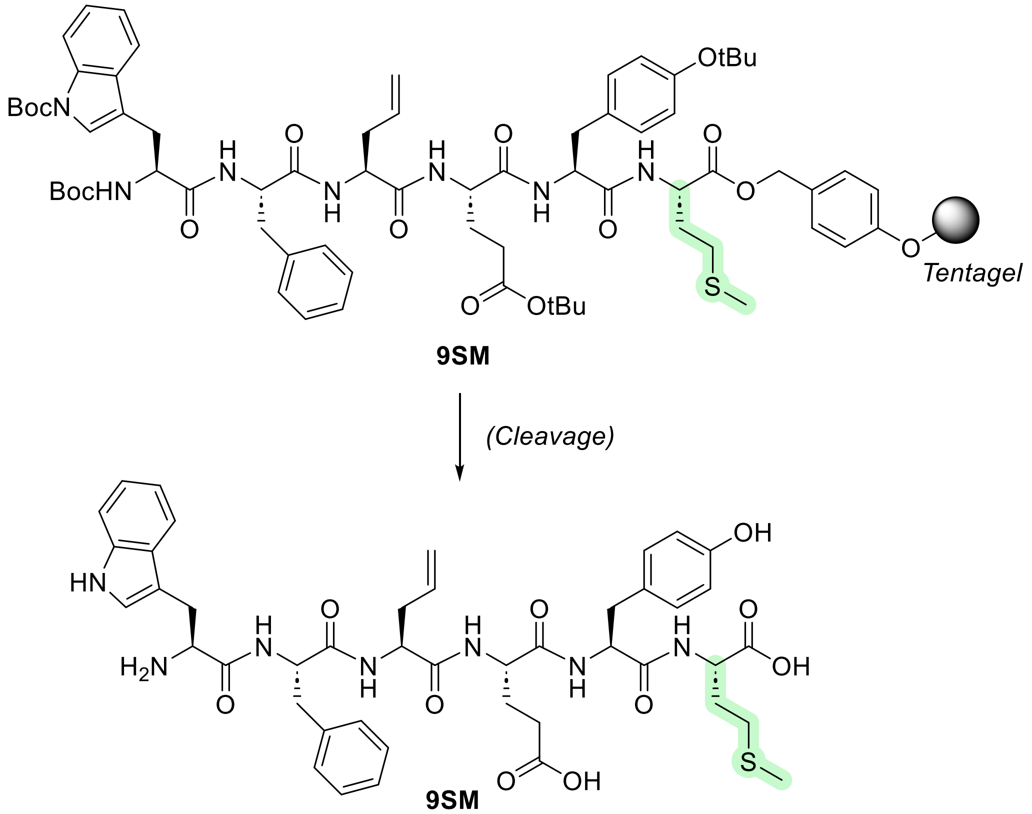

9SM

Chemical Formula: $C_{44}H_{53}N_7O_{10}S$

Exact Mass: 871.3575

Sequence: BocHN – Trp(Boc) – Phe – GAll – Glu(tBu) – Tyr(tBu) – Met – Wanglinker – Tentagel-resin

The title compound was ordered on-resin. Test cleavage of the peptide on-resin according to GP7/GP2B yielded the title compound as a white solid and allowed loading determination by qNMR (0.114 mmol/g loading determined by qNMR, ca. 67% UV-purity).

NMR details: spectra attached, not in detail reported.

HRMS (ESI+) (m/z): $[M+H]^+$ calculated for $C_{44}H_{54}N_7O_{10}S^+$ 872.3647; found 872.3660.

8.1.14 10SM

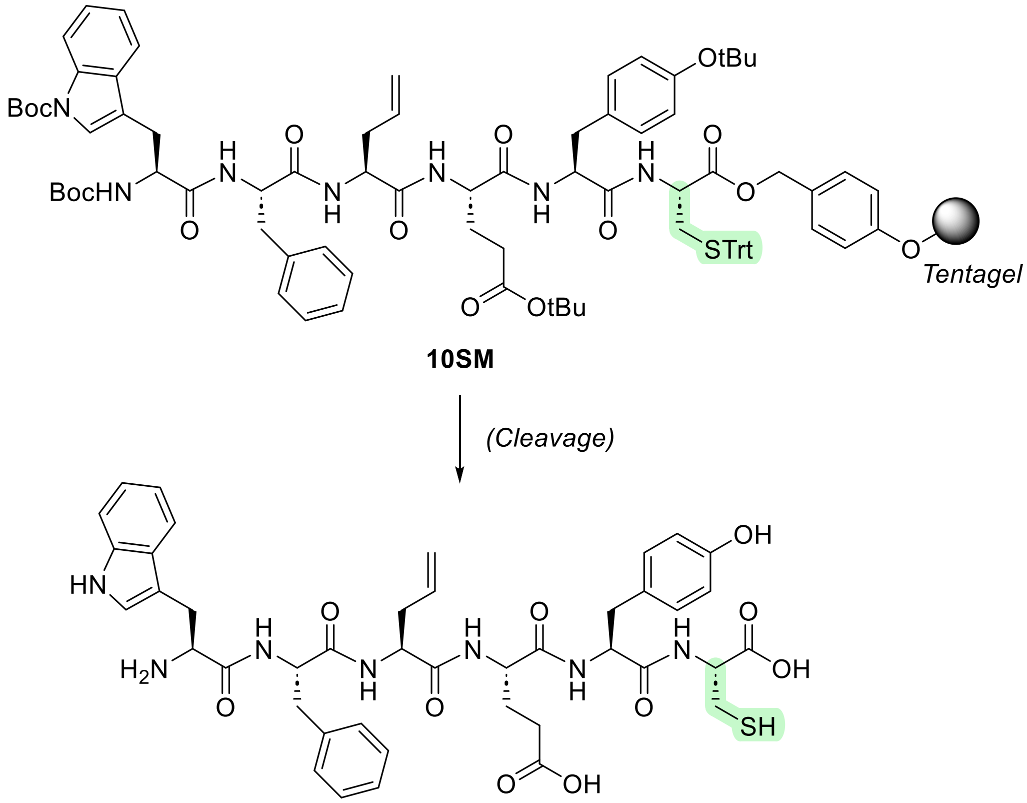

10SM
Chemical Formula: $C_{42}H_{49}N_7O_{10}S$
Exact Mass: 843.3262

Sequence: BocHN – Trp(Boc) – Phe – GAll – Glu(tBu) – Tyr(tBu) – Cys(Trt) – Wanglinker – Tentagel-resin

The title compound was ordered on-resin. Test cleavage of the peptide on-resin according to GP7/GP2B yielded the title compound as a white solid and allowed loading determination by qNMR (0.143 mmol/g loading determined by qNMR, ca. 75% UV-purity)

NMR details: spectra attached, not in detail reported.
HRMS: most decomposed until measurement, but product signal visible in traces
HRMS (ESI+) (m/z): $[M+H]^+$ calculated for $C_{42}H_{50}N_7O_{10}S^+$ 844.3334; found 844.3340.

8.1.15 11SM

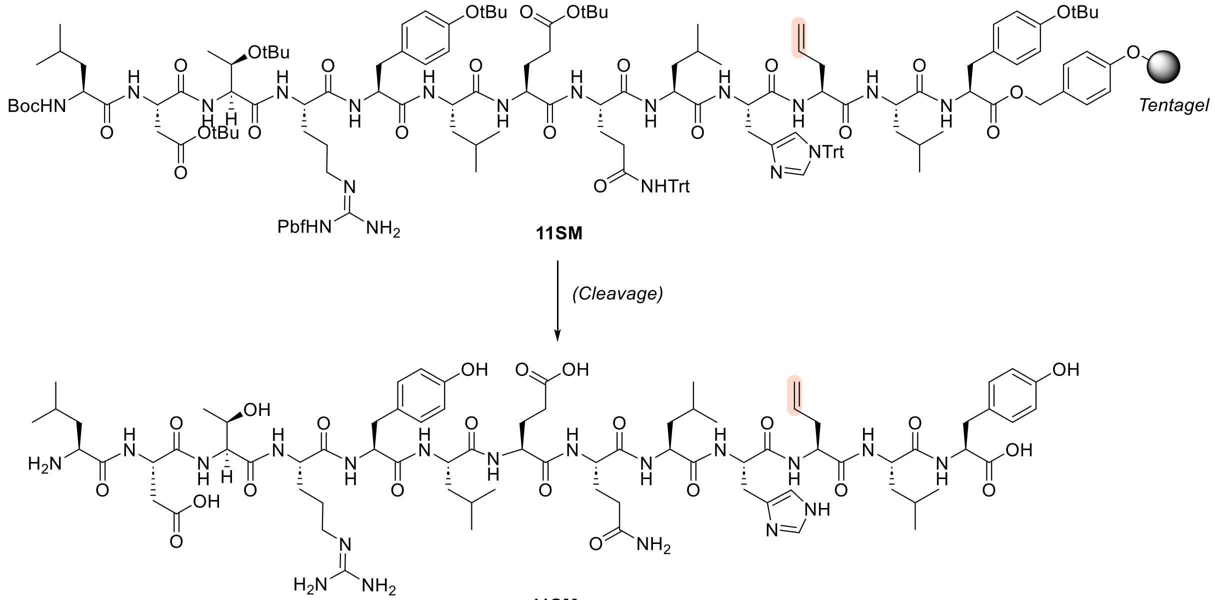

11SM
Chemical Formula: $C_{77}H_{117}N_{19}O_{22}$
Exact Mass: 1659.8621

Sequence: BocHN – Leu – Asp(tBu) – Thr(tBu) – Arg(Pbf) – Tyr(tBu) – Leu – Glu(tBu) – Gln(Trt) – Leu – His(Trt) – GAll – Leu – Tyr(tBu) – Wanglinker-Tentagel-resin

The title compound was prepared according to General Procedure GP1B on a 0.3 mmol scale using FmocHN-Tyr(tBu)-Wanglinker-Tentagel-resin (1.250 g, 0.3 mmol, 0.24 mmol/g loading). Test cleavage of the peptide on-resin according to GP7/GP2B yielded the title compound as a white solid and allowed loading determination by qNMR (1.751 g resin, 0.23 mmol, 0.13 mmol/g loading determined by weight difference, 0.13 mmol/g loading determined by qNMR, 76%, ca. 88% UV-purity).

NMR details: spectra attached, not in detail reported.
HRMS (ESI+) (m/z): $[M+H]^+$ calculated for $C_{77}H_{118}N_{19}O_{22}^+$ 1660.8693; found 1660.8722.

8.1.16 12SM

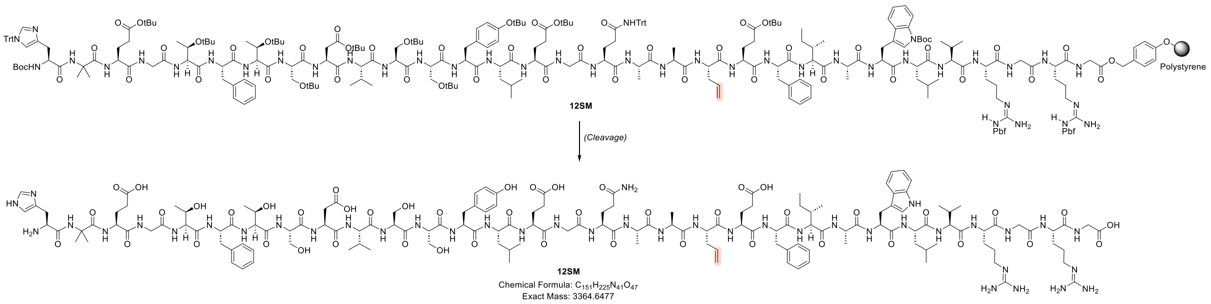

Sequence: BocHN – His(Trt) – Aib – Glu(tBu) – Gly – Thr(tBu) – Phe – Thr(tBu) – Ser(tBu) – Asp(tBu) – Val – Ser(tBu) – Ser(tBu) – Tyr(tBu) – Val – Glu(tBu) – Gly – Gln(Trt) – Ala – Ala – GAll – Glu(tBu) – Phe – Ile – Ala – Trp(Boc) – Leu – Val – Asp(Pbf) – Gly – Asp(Pbf) – Gly - Wanglinker – Polystyrene-resin

The title compound was prepared according to General Procedure GP1B on a 0.3 mmol scale using FmocHN-Gly-Wanglinker-Polystyrene-resin (0.769 g, 0.3 mmol, 0.39 mmol/g loading). Test cleavage of the peptide on-resin according to GP7/GP2B yielded the title compound as a white solid and allowed loading determination by qNMR (2.6 g resin, 0.20 mmol, 0.15 mmol/g loading determined by weight difference, 0.079 mmol/g loading estimated with all the impurities (rather inexact), 68%, <64% UV-purity).

NMR: spectra attached, not in detail reported.
HRMS (ESI+) (m/z): $[M+3H]^{3+}$ calculated for $C_{151}H_{228}N_{41}O_{47}{}^{3+}$ 1122.5565; found 1122.5559.

8.1.17 13SM

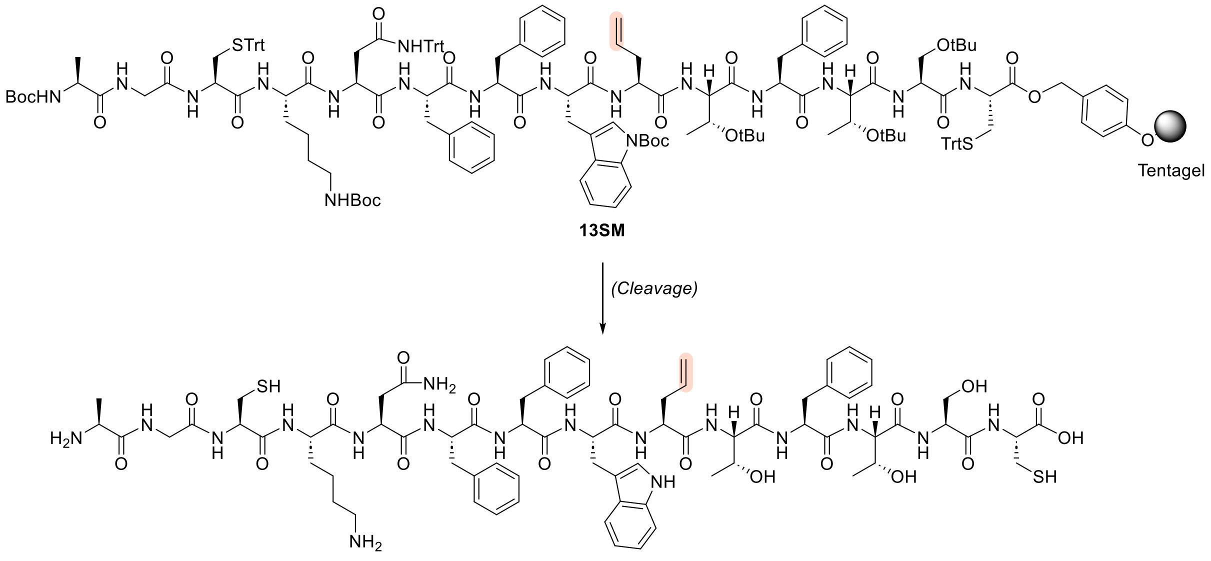

13SM
Chemical Formula: $C_{75}H_{101}N_{17}O_{19}S_2$
Exact Mass: 1607.6901

Sequence: BocHN – Ala – Gly – Cys(Trt) – Lys – Asn(Trt) – Phe – Phe – Trp(Boc) – GAll – Thr(tBu) – Phe – Thr(tBu) – Ser(tBu) – Cys(Trt) – Wanglinker – Tentagel-resin

The title compound was prepared according to General Procedure GP1B on a 0.3 mmol scale using FmocHN-Cys(Trt)-Wanglinker-Tentagel-resin (1.364 g, 0.3 mmol, 0.22 mmol/g

loading). Test cleavage of the peptide on-resin according to GP7/GP2B yielded the title compound as a white solid and allowed loading determination by qNMR (1.855 g resin, 0.17 mmol, 0.12 mmol/g loading determined by weight difference, 0.09 mmol/g loading determined by qNMR, 56%, ca. 87% UV-purity).

NMR details: spectra attached, not in detail reported.
HRMS (ESI+) (m/z): $[M+2H]^{2+}$ calculated for $C_{75}H_{103}N_{17}O_{19}S_2^{2+}$ 804.8524; found 804.8569.

8.1.18 14SM

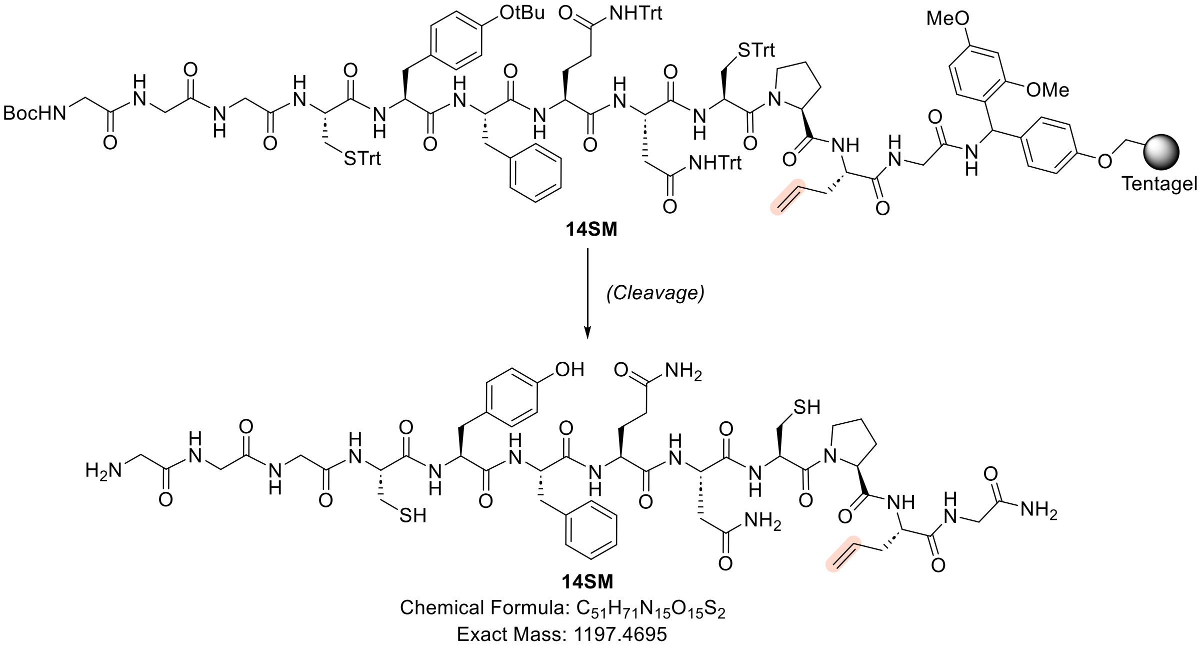

Sequence: BocHN – Gly – Gly – Gly – Cys(Trt) – Tyr(tBu) – Phe – Gln(Trt) – Asn(Trt) – Cys(Trt) – Pro – GAll – Gly – Rink amide linker – Tentagel-resin

The title compound was ordered on-resin. Test cleavage of the peptide on-resin according to GP7/GP2B yielded the title compound as a white solid and allowed loading determination by qNMR (0.063 mmol/g loading determined by qNMR, ca. 89% UV-purity).

NMR details: spectra attached, not in detail reported.
HRMS: sample very impure until measurement; accurate mass not found
HRMS (ESI+) (m/z): $[M+H]^{+}$ calculated for $C_{51}H_{72}N_{15}O_{15}S_2^{+}$ 1198.4769; found 1198.4316.

8.1.19 15SM

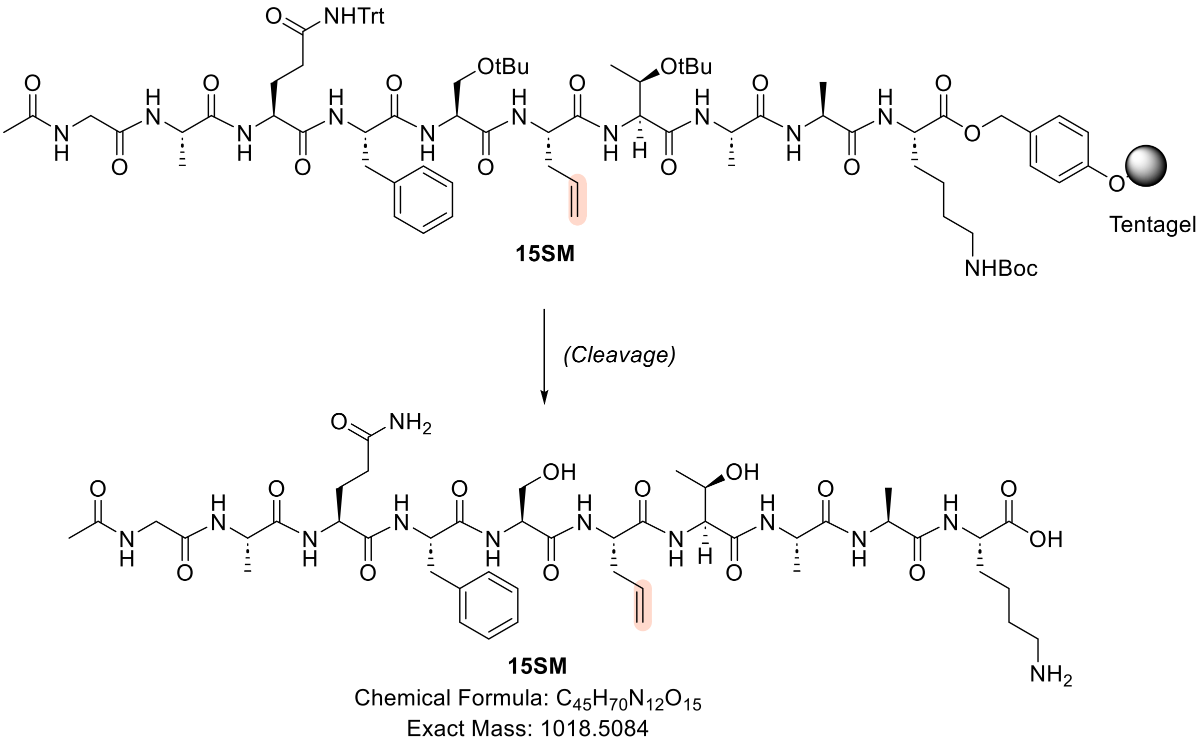

Sequence: AcHN – Gly – Ala – Gln(Trt) – Phe – Ser(tBu) – GAll – Thr(tBu) – Ala – Ala – Lys(Boc) – Wanglinker-Tentagel-resin

The title compound was prepared according to General Procedure GP1B on a 0.3 mmol scale using FmocHN-Lys(Boc)-Wanglinker-Tentagel-resin (1.304 g, 0.3 mmol, 0.23 mmol/g loading). Test cleavage of the peptide on-resin according to GP7/GP2B yielded the title compound as a white solid and allowed loading determination by qNMR (1.514 g resin, 0.23 mmol, 0.14 mmol/g loading determined by weight difference, 0.15 mmol/g loading determined by qNMR, 76%, ca. 98% UV-purity).

NMR details: spectra attached, not in detail reported.
HRMS (ESI+) (m/z): $[M+2H]^{2+}$ calculated for $C_{45}H_{72}N_{12}O_{15}^{2+}$ 510.2615; found 510.2635.

9. Hydroformylation syntheses and late-stage modifications

9.1 Hydroformylation of peptides

9.1.1 Tripeptides

SI-3A

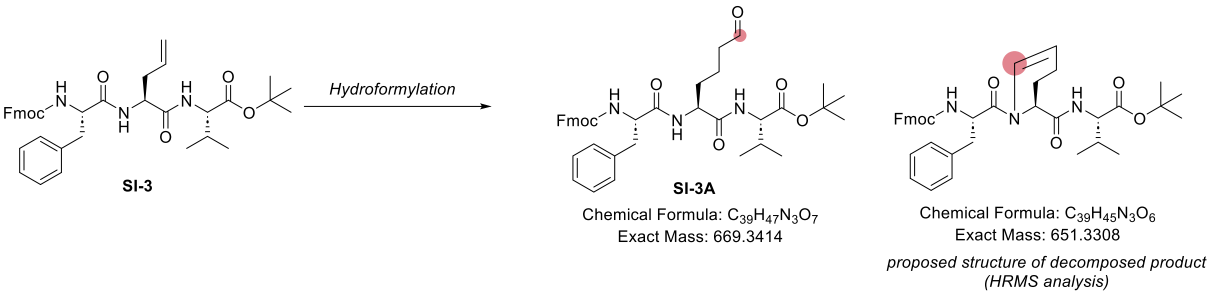

The title compound was prepared following general procedure GP3A (but not resin-bound, stirred instead, prepared in glovebox, using stabilizing PTFE discs with PTFE/silicone septa for two-chamber (no H-caps)), employing **SI-3A** (9.6 mg, 15 µmol, 1 equiv.), $Rh(COD)_2BF_4$ (0.2 mg, 0.5 µmol, 3.5 mol%)/6-DPPon (0.7 mg, 2.5 µmol, 17 mol%) in THF (156 µL) in chamber B, and [^{12}C]-SilaCOgen (39 mg, 0.16 mmol, 11 equiv.) and $NaBH_4$ (6 mg, 0.16 mmol, 11 equiv.) in THF (1 mL) in chamber A. The two-chamber reactor was stirred at 30 °C for 16 hours. The solvent was removed with a flow of nitrogen, and the crude product **SI-3A** was analyzed by qNMR using 1,3,5-trimethoxy-benzene as internal standard.

Decomposition until HRMS analysis:
Minor Mass: **HRMS** (ESI+) (m/z): $[M+H]^+$ calculated for $C_{39}H_{48}N_3O_7^+$ 670.3487; found 670.3492.
Minor Mass: **HRMS** (ESI+) (m/z): $[M+H]^+$ calculated for $C_{39}H_{46}N_3O_6^+$ 652.3381; found 652.3386.

SI-4A

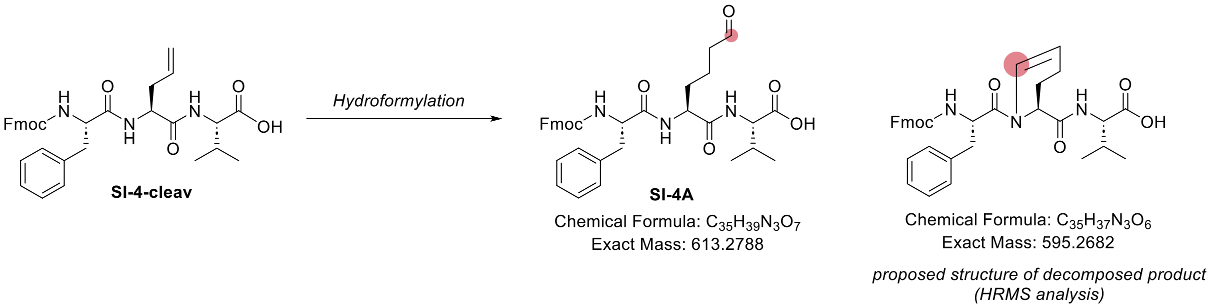

The title compound was prepared following general procedure GP3A (but not resin-bound, stirred instead, prepared in glovebox, using stabilizing PTFE discs with PTFE/silicone septa for two-chamber (no H-caps)), employing **SI-4-cleav** (8.8 mg, 15 µmol, 1 equiv.) (cleaved **SI-4**), $Rh(COD)_2BF_4$ (0.2 mg, 0.5 µmol, 3.5 mol%)/6-DPPon (0.7 mg, 2.5 µmol, 17 mol%) in THF (156 µL) in chamber B, and [^{12}C]-SilaCOgen (39 mg, 0.16 mmol, 11 equiv.) and $NaBH_4$ (6 mg, 0.16 mmol, 11 equiv.) in THF (1 mL) in chamber A. The two-chamber reactor was stirred at 30 °C for 16 hours. The solvent was removed with a flow of nitrogen, and the crude product **SI-4A** was analyzed by qNMR using 1,3,5-trimethoxy-benzene as internal standard.

SI-4A (synthesis on resin)

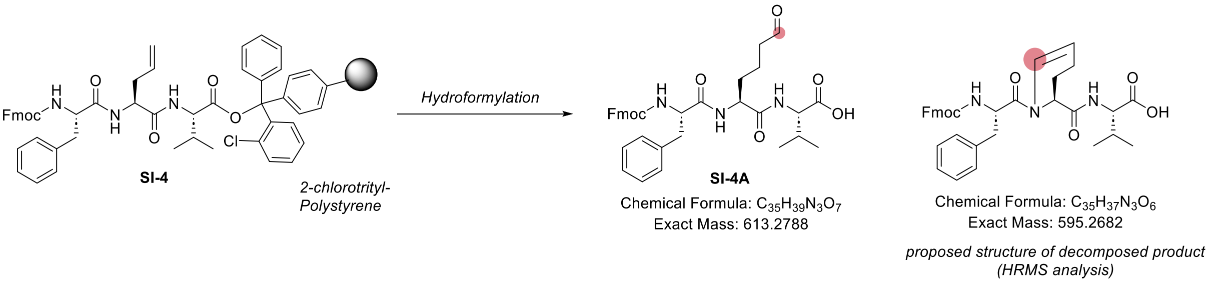

The title compound was prepared following general procedure GP3A, employing **SI-4** on-resin (34 mg, ca. 15 µmol, 0.44 mmol/g, 1 equiv.), $Rh(COD)_2BF_4$ (0.2 mg, 0.5 µmol, 3.5 mol%)/6-DPPon (0.7 mg, 2.5 µmol, 17.3 mol%) in THF (0.5 mL) in chamber B, and $[^{12}C]$-SilaCOgen (39 mg, 0.16 mmol, 11 equiv.) and $NaBH_4$ (6 mg, 0.16 mmol, 11 equiv.) in THF (1 mL) in chamber A. The two-chamber reactor was shaken at RT for 16 hours. After cleavage according to GP2C, the crude product was analyzed by qNMR using 1,3,5-trimethoxy-benzene as internal standard.

9.1.2 Hexapeptide 1C

$[^{12}C]$1C

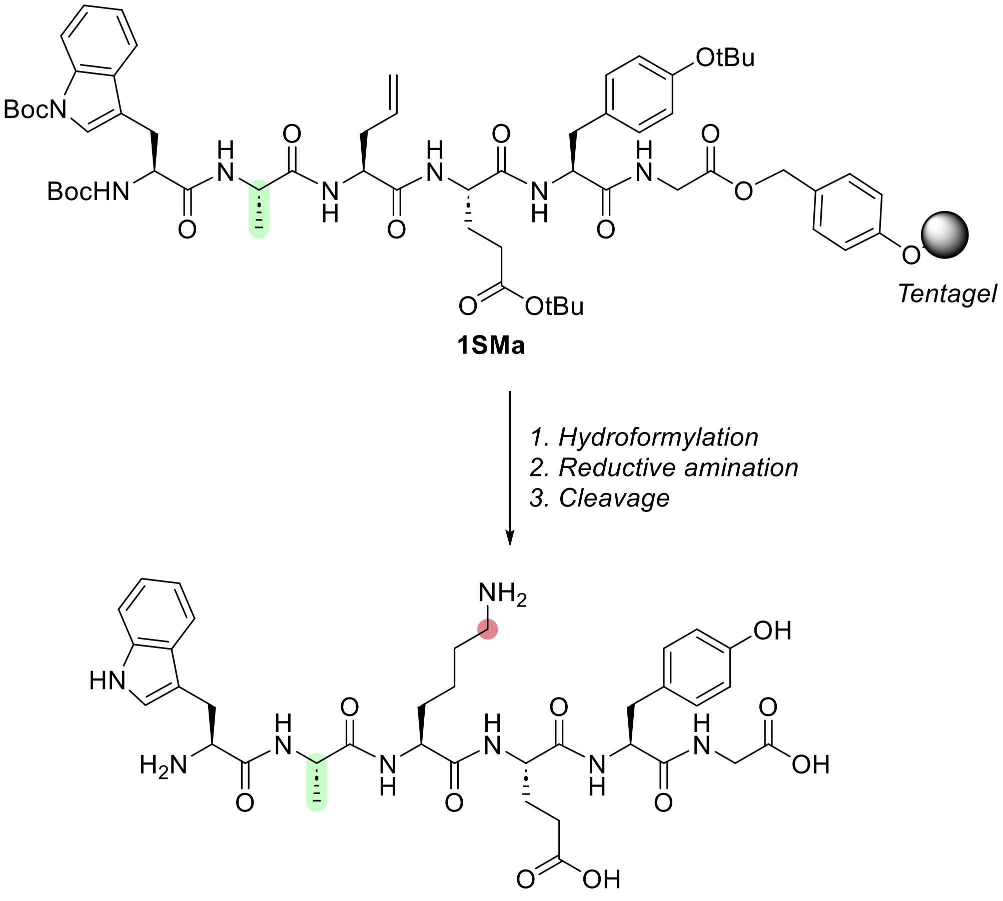

$[^{12}C]$1C
Chemical Formula: $C_{36}H_{48}N_8O_{10}$
Exact Mass: 752.3493

The title compound was prepared following general procedure GP3B, employing resin-bound **1SMa** (147 mg, ca. 25 µmol, 1 equiv., 0.170 mmol/g loading).

Reductive amination was performed according to general procedure GP6B, and was in total performed 1 time, followed by cleavage according to GP2B.

The crude product was purified by preparative HPLC (5% B for 1 min, 5–35% B for 11 min, 35–95% B for 3 min, 95–5% B for 1 min, 5% B for 1 min) to isolate **[12C]1C** as a white solid (9.5 mg, with 2x TFA salt: 9.7 µmol, yield 39%, >99% UV-purity by LC-MS).

NMR details: spectra attached, not in detail reported.

HRMS (ESI+) (m/z): $[M+H]^+$ calculated for $C_{36}H_{49}N_8O_{10}^+$ 753.3566; found 753.3665.

9.1.3 Hexapeptide 2C

[12C]2C

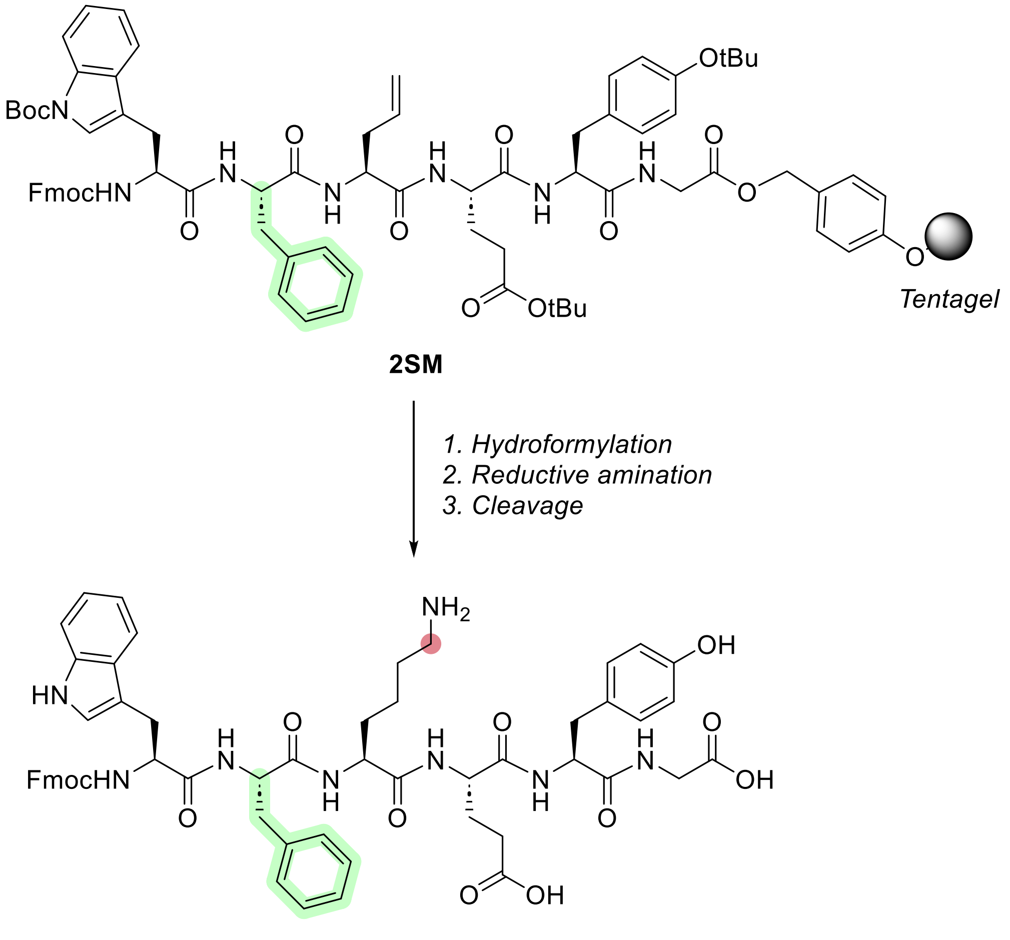

[12C]2C
Chemical Formula: $C_{57}H_{62}N_8O_{12}$
Exact Mass: 1050.4487

The title compound was prepared following general procedure GP3B, employing resin-bound **2SM** (210 mg, ca. 22.5 µmol, 1 equiv., 0.107 mmol/g loading, note: different loading than described in the reported **2SM**, because it was prepared in several batches).

Reductive amination was performed according to general procedure GP6B, and was in total performed 1 time, followed by cleavage according to GP2B.

The crude product was purified by preparative HPLC (20% B for 1 min, 20–95% B for 17 min, 95% B for 1 min, 95–20% B for 1 min, 20% B for 1 min) to isolate **[12C]2C** as a white solid (14.5 mg, with 1x TFA salt: 12.5 µmol, yield 55%, 96% UV-purity by LC-MS).

NMR details: spectra attached, not in detail reported.
HRMS (ESI+) (m/z): $[M+H]^+$ calculated for $C_{57}H_{63}N_8O_{12}^+$ 1051.4560; found 1051.4572.

[13C]2C

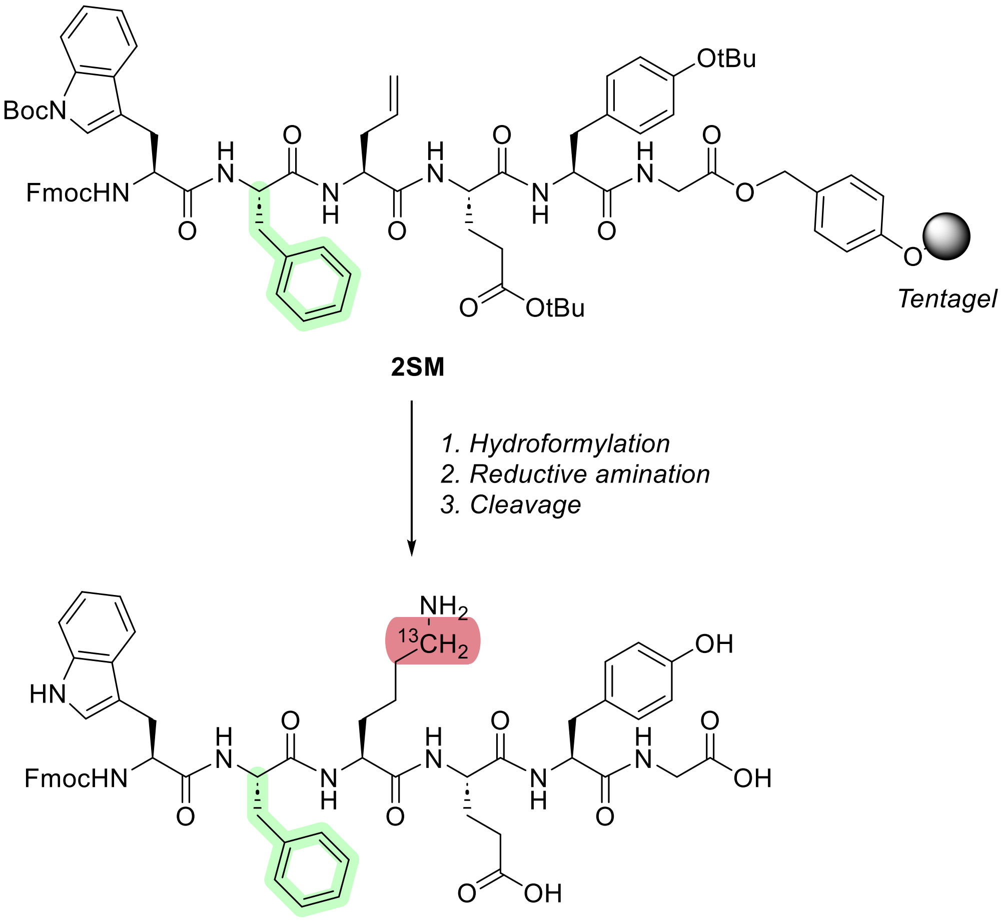

[13C]2C
Chemical Formula: $C_{56}{}^{13}CH_{62}N_8O_{12}$
Exact Mass: 1051.4521

The title compound was prepared following general procedure GP3B, employing resin-bound **2SM** (210 mg, ca. 22.5 µmol, 1 equiv., 0.107 mmol/g loading, note: different loading than described in the reported **2SM**, because it was prepared in several batches).

Reductive amination was performed according to general procedure GP6B, and was in total performed 1 time, followed by cleavage according to GP2B.

The crude product was purified by preparative HPLC (20% B for 1 min, 20–95% B for 17 min, 95% B for 1 min, 95–20% B for 1 min, 20% B for 1 min) to isolate **[12C]2C** as a white solid (12.7 mg, with 1x TFA salt: 10.9 µmol, yield 49%, isotope incorporation +0.99 ^{13}C/molecule, >99% UV-purity by LC-MS).

NMR details: spectra attached, not in detail reported.

HRMS (ESI+) (m/z): $[M+H]^+$ calculated for $C_{56}{}^{13}CH_{63}N_8O_{12}{}^+$ 1052.4594; found 1052.4623.

[^{13}C]2C (0.36 equiv. cat., not described in main manuscript, but includes 2D-NMR data)

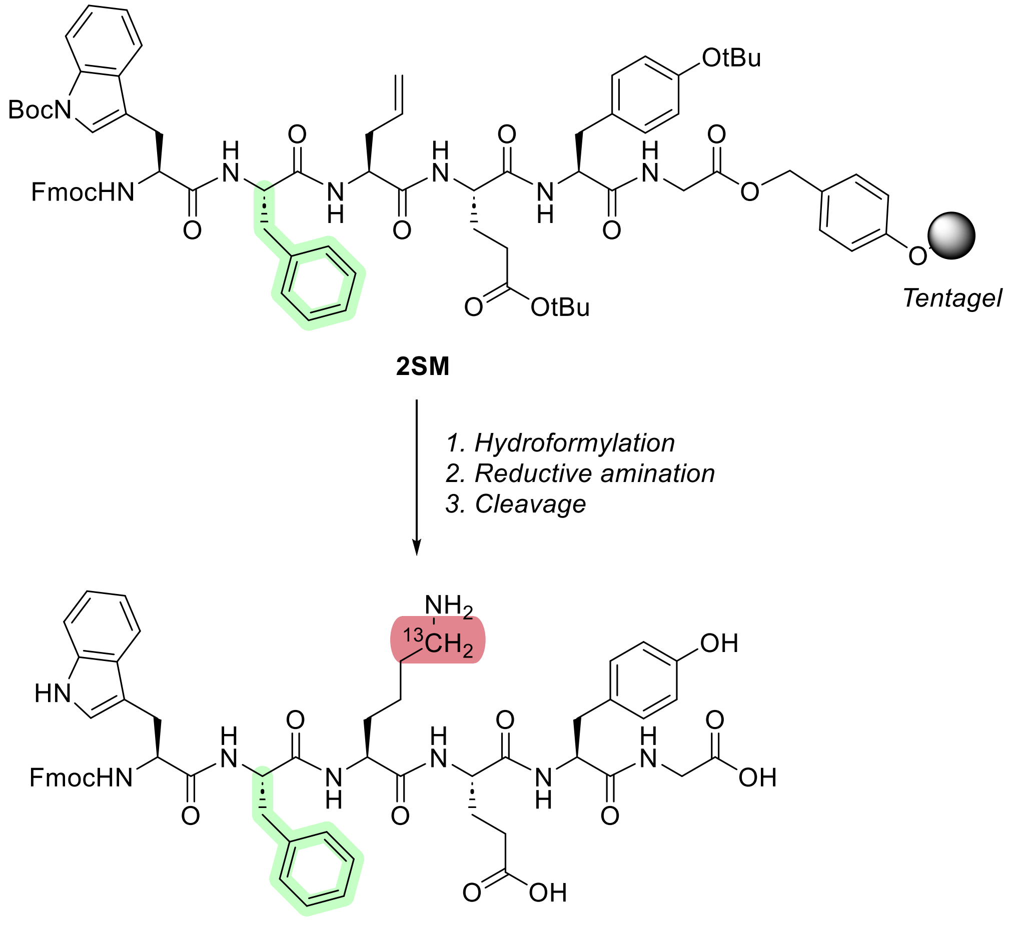

[^{13}C]2C
Chemical Formula: $C_{56}{}^{13}CH_{62}N_8O_{12}$
Exact Mass: 1051.4521

The title compound was prepared following general procedure GP3A, employing **2SM** (146 mg, ca. 25 µmol, 1 equiv., 0.17 mmol/g loading), $Rh(COD)_2BF_4$ (3.7 mg, 9 µmol, 0.36 equiv.)/6-DPPon (13.5 mg, 48 µmol, 1.9 equiv.) in DMSO:H_2O, 95:5 (1 mL) in chamber B, and [^{13}C]-SilaCOgen (24 mg, 0.1 mmol, 4 equiv.) and $NaBH_4$ (4 mg, 0.1 mmol, 4 equiv.) in diglyme (1 mL) in chamber A. The two-chamber reactor was shaken at RT for 60 hours.

Reductive amination was performed according to general procedure GP6A, employing a solution of tritylamine in DMSO, 1% acetic acid (5 mL, 31 mg/mL, 0.12 mmol/mL) and a solution of $NaCNBH_3$ in CH_2Cl_2:MeOH, 3:1 (5 mL, 10 mg/mL, 0.15 mmol/mL). The reductive amination procedure was performed two times, followed by cleavage according to GP2B.
Only half of the crude product was purified by preparative HPLC (gradient: 5% B for 3 min, 5–35% B for 2 min, 35–45% B for 15 min, 45–60% B for 4 min) to isolate **[^{13}C]2C** as a white solid (with 1x TFA salt: 5.5 mg, 4.7 µmol, 38%, isotope incorporation ca. +0.99 ^{13}C/molecule, 98% UV-purity by LC-MS).

It should be noted that the product was instable at room temperature in DMSO, leading to Fmoc-deprotection.
NMR details: spectra attached, not in detail reported.
HRMS (ESI+) (m/z): $[M+H]^+$ calculated for $C_{56}{}^{13}CH_{63}N_8O_{12}{}^+$ 1052.4594; found 1052.4594.

[^{14}C]2C

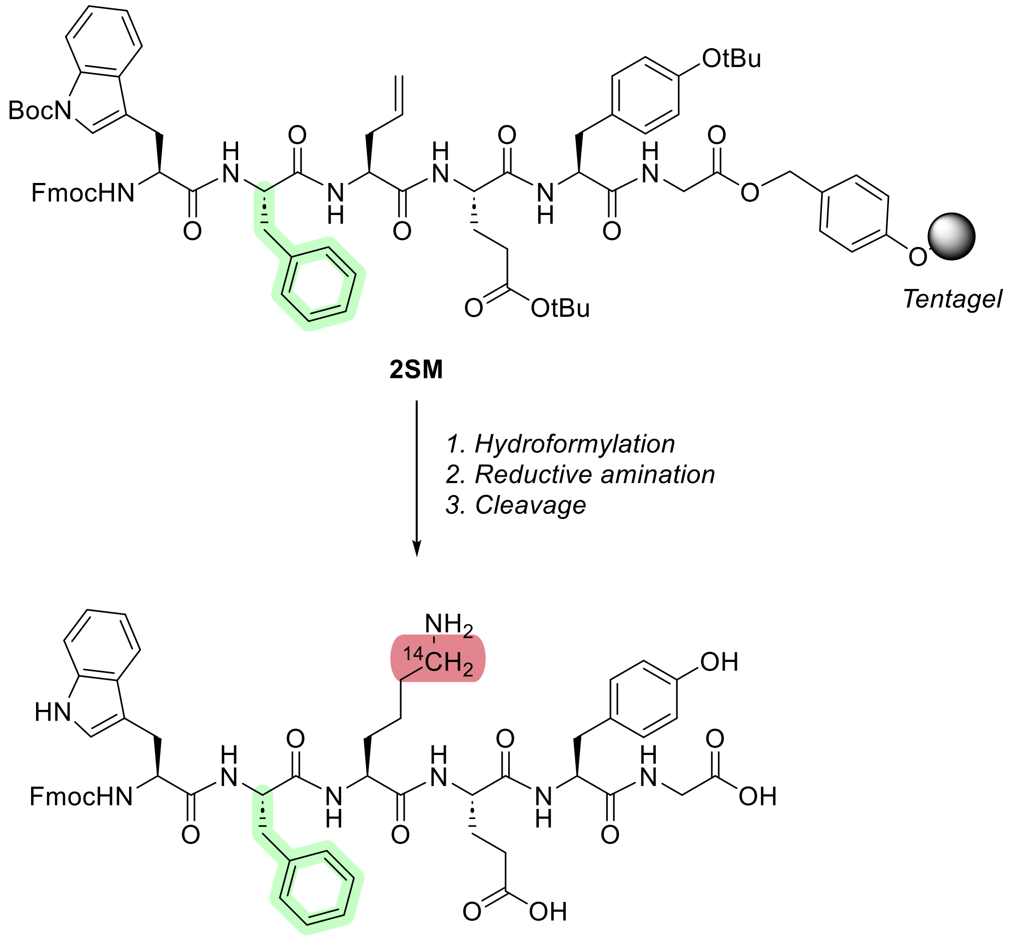

[^{14}C]2C
Chemical Formula: $C_{56}{}^{14}CH_{62}N_8O_{12}$
Exact Mass: 1052.4520

The title compound was prepared following general procedure GP4, employing resin-bound **2SM** (294 mg, ca. 50 µmol, 1 equiv., 0.17 mmol/g loading), $Rh(COD)_2BF_4$ (7.5 mg, 18.5 µmol, 0.37 equiv.)/6-DPPon (27 mg, 97 µmol, 1.9 equiv.) in DMSO:H_2O, 95:5 (2 mL) in chamber B, $^1H_2$-gas (2.5 mL, 2 equiv., via syringe), and ^{12}COgen:^{14}COgen (95:5, 24 mg, 0.1 mmol, 2 equiv., 10.95 MBq), $Pd(dba)_2$ (5.8 mg, 10 µmol), DIPEA (52 µL, 0.3 mmol), and tri-tert-butylphosphine (10 µL, 10 µmol, 1M in toluene) in diglyme (1 mL) in chamber A.

Reductive amination was performed according to general procedure GP6A, employing a solution of tritylamine in DMSO, 1% acetic acid (5 mL, 31 mg/mL, 0.12 mmol/mL) and a solution of $NaCNBH_3$ in CH_2Cl_2:MeOH, 3:1 (5 mL, 10 mg/mL, 0.15 mmol/mL). The reductive amination step was performed two times, followed by cleavage according to GP2B.

The crude product was purified by preparative HPLC (gradient: 5% B for 3 min, 5–35% B for 2 min, 35–45% B for 18 min, 45–60% B for 2 min) to isolate **[^{14}C]2C** (1.51 MBq, isotope incorporation ca. 0.05 ^{14}C/molecule, specific activity 116 MBq/mmol, 3.1 mCi/mmol,

13% RCY, 24% (12.2 µmol) yield calculated *via* radioactivity, 94% radiochemical purity, 96% UV-purity by LC-MS).
It should be noted that the product was instable at room temperature in DMSO, leading to Fmoc-deprotection.

NMR details: spectra attached, not in detail reported.
HRMS (ESI+) (m/z): $[M+H]^+$ calculated for $C_{56}{}^{12}CH_{63}N_8O_{12}{}^+$ 1051.4560; found 1051.4562.

9.1.4 Hexapeptide 3C

[12C]3C

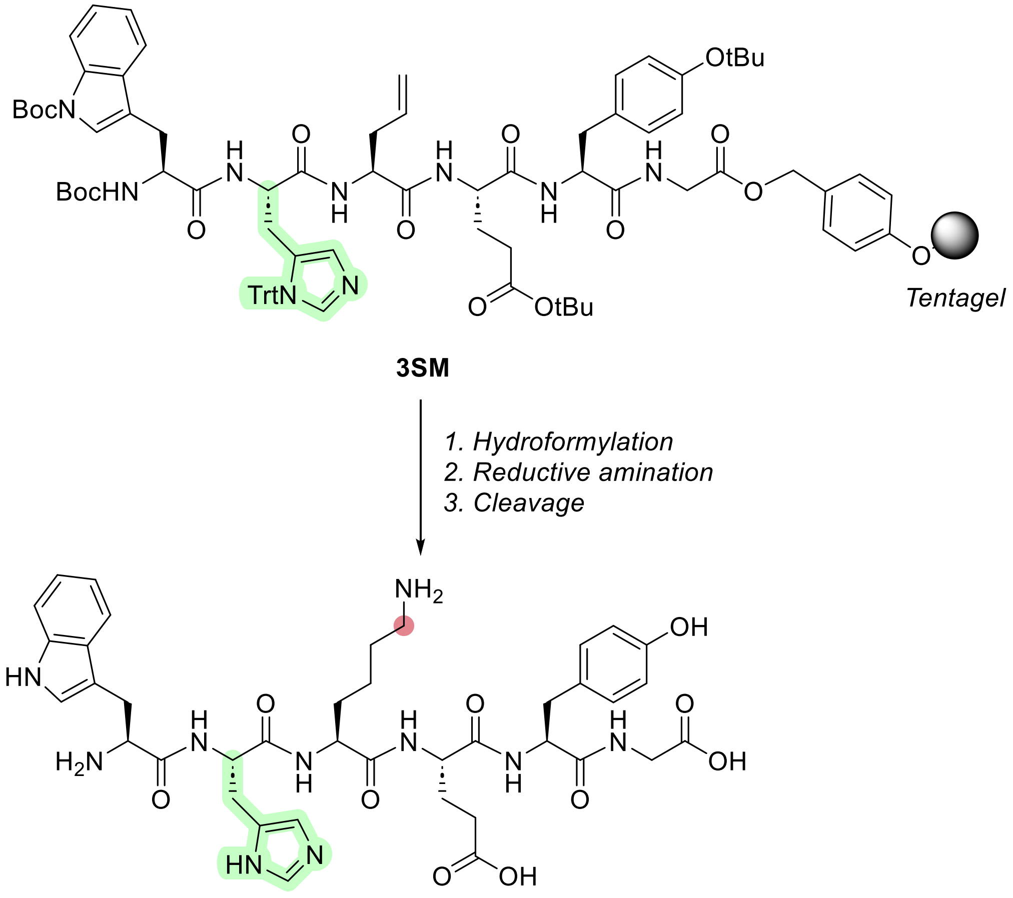

[12C]3C
Chemical Formula: $C_{39}H_{50}N_{10}O_{10}$
Exact Mass: 818.3711

The title compound was prepared following general procedure GP3B, employing resin-bound **3SM** (169 mg, ca. 21.8 µmol, 1 equiv., 0.129 mmol/g loading).

Reductive amination was performed according to general procedure GP6B, and was in total performed 1 time, followed by cleavage according to GP2B.

The crude product was purified by preparative HPLC (5% B for 1 min, 5–60% B for 17 min, 60–95% B for 1 min, 95–5% B for 1 min, 5% B for 1 min) to isolate **[12C]3C** as a white solid (16.8 mg, with 3 x TFA salt: 14.5 µmol, yield 66%, 93% UV-purity by LC-MS).

NMR details: spectra attached, not in detail reported.
HRMS (ESI+) (m/z): $[M+H]^+$ calculated for $C_{39}H_{51}N_{10}O_{10}{}^+$ 819.3784; found 819.3791.

[^{13}C]3C

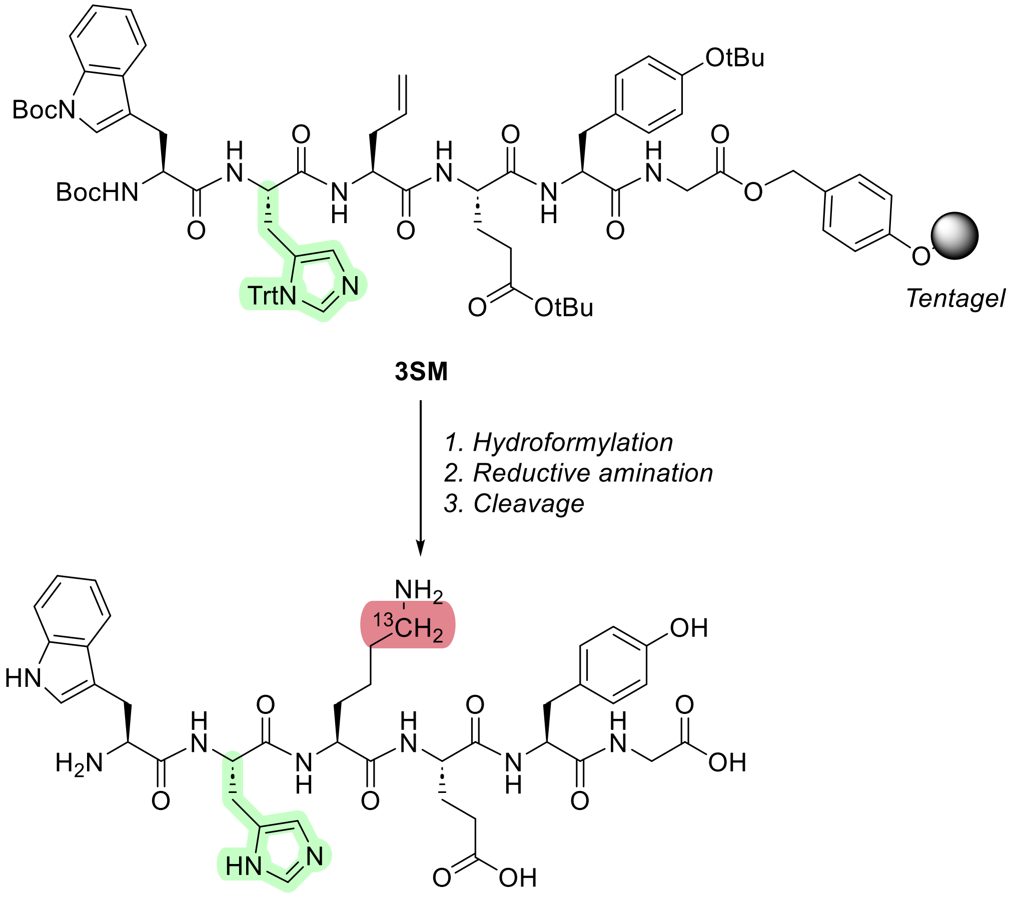

[^{13}C]3C

Chemical Formula: $C_{38}{}^{13}CH_{50}N_{10}O_{10}$
Exact Mass: 819.3745

The title compound was prepared following general procedure GP3B, employing resin-bound **3SM** (172 mg, ca. 22.2 µmol, 1 equiv., 0.129 mmol/g loading).

Reductive amination was performed according to general procedure GP6B, and was in total performed 1 time, followed by cleavage according to GP2B.

The crude product was purified by preparative HPLC (5% B for 1 min, 5–60% B for 17 min, 60–95% B for 1 min, 95–5% B for 1 min, 5% B for 1 min) to isolate **[^{13}C]3C** as a white solid (17 mg, with 3 x TFA salt: 14.7 µmol, yield 66%, isotope incorporation +0.99 ^{13}C/molecule, >99% UV-purity by LC-MS).

NMR details: spectra attached, not in detail reported.

HRMS (ESI+) (m/z): $[M+H]^+$ calculated for $C_{38}{}^{13}CH_{51}N_{10}O_{10}{}^+$ 820.3818; found 820.3864.

9.1.5 Hexapeptide 4C

[12C]4C

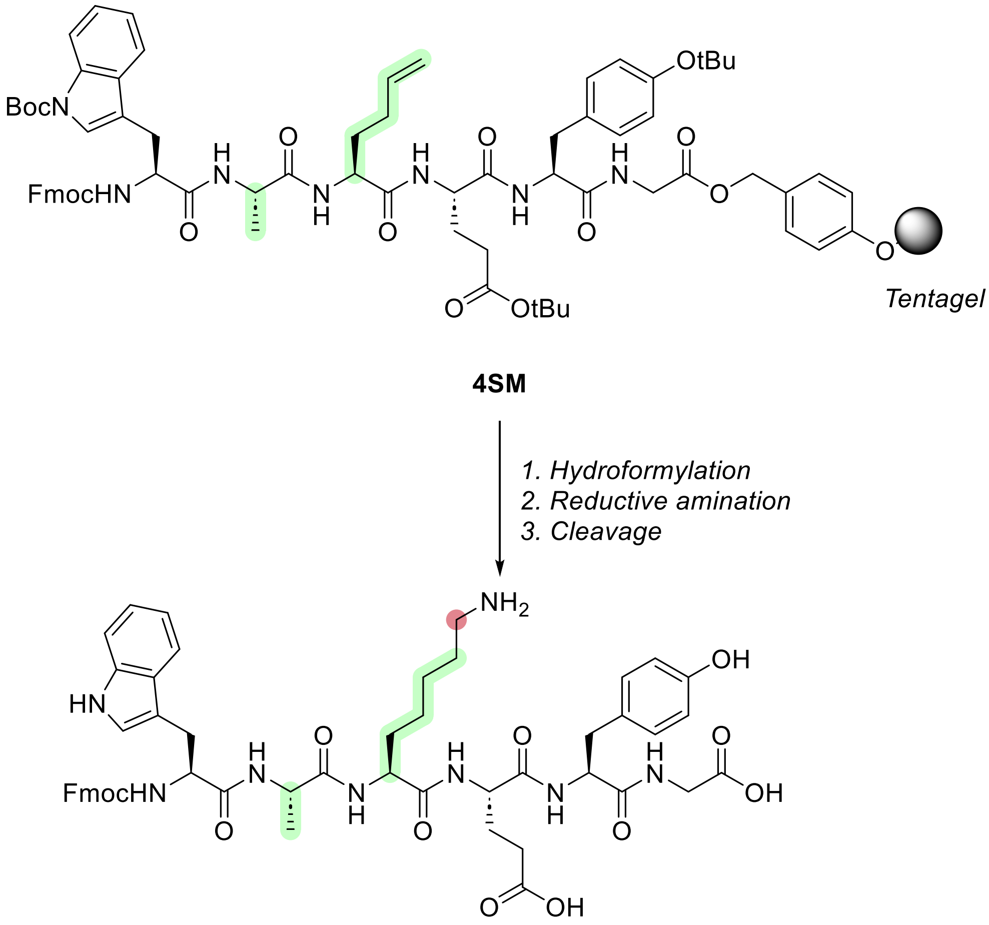

[12C]4C

Chemical Formula: $C_{52}H_{60}N_8O_{12}$

Exact Mass: 988.4331

The title compound was prepared following general procedure GP3B, employing resin-bound **4SM** (170 mg, ca. 19.4 µmol, 1 equiv., 0.114 mmol/g loading).

Reductive amination was performed according to general procedure GP6B, and was in total performed 1 time, followed by cleavage according to GP2B.

The crude product was purified by preparative HPLC (20% B for 1 min, 20–95% B for 17 min, 95% B for 1 min, 95–20% B for 1 min, 20% B for 1 min) to isolate **[^{12}C]4C** as a white solid (12.6 mg, with 1x TFA salt: 11.4 µmol, yield 59%, 90% UV-purity by LC-MS).

NMR details: spectra attached, not in detail reported.

HRMS (ESI+) (m/z): $[M+H]^+$ calculated for $C_{52}H_{61}N_8O_{12}^+$ 989.4403; found 989.4435.

9.1.6 Hexapeptide 6C

[^{12}C]6C

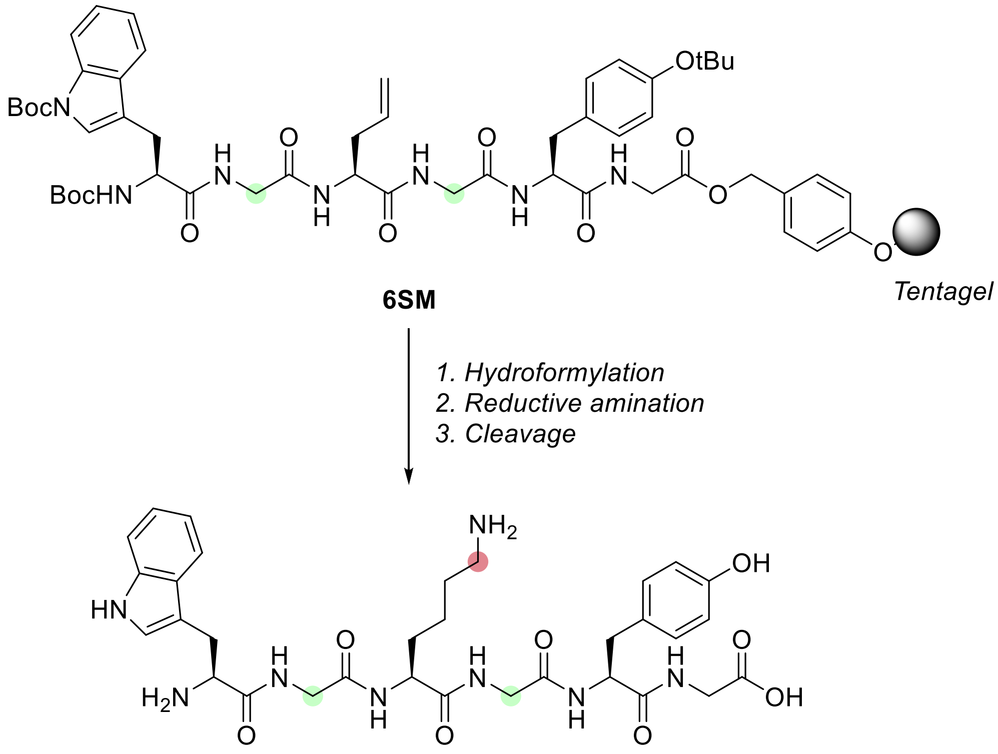

[^{12}C]6C

Chemical Formula: $C_{32}H_{42}N_8O_8$

Exact Mass: 666.3126

The title compound was prepared following general procedure GP3B, employing resin-bound **6SM** (119 mg, ca. 25.5 µmol, 1 equiv., 0.214 mmol/g loading).

Reductive amination was performed according to general procedure GP6B, and was in total performed 1 time, followed by cleavage according to GP2B.

The crude product was purified by preparative HPLC (5% B for 1 min, 5–60% B for 17 min, 60–95% B for 1 min, 95–5% B for 1 min, 5% B for 1 min) to isolate **[^{12}C]6C** as a white solid (10.5 mg, with 2x TFA salt: 11.7 µmol, yield 46%, 92% UV-purity by LC-MS (2 product isomer signals by UV, respectively 83% and 9%)).

NMR details: spectra attached, not in detail reported.

HRMS (ESI+) (m/z): $[M+H]^+$ calculated for $C_{32}H_{43}N_8O_8^+$ 667.3198; found 667.3204.

[^{13}C]6C

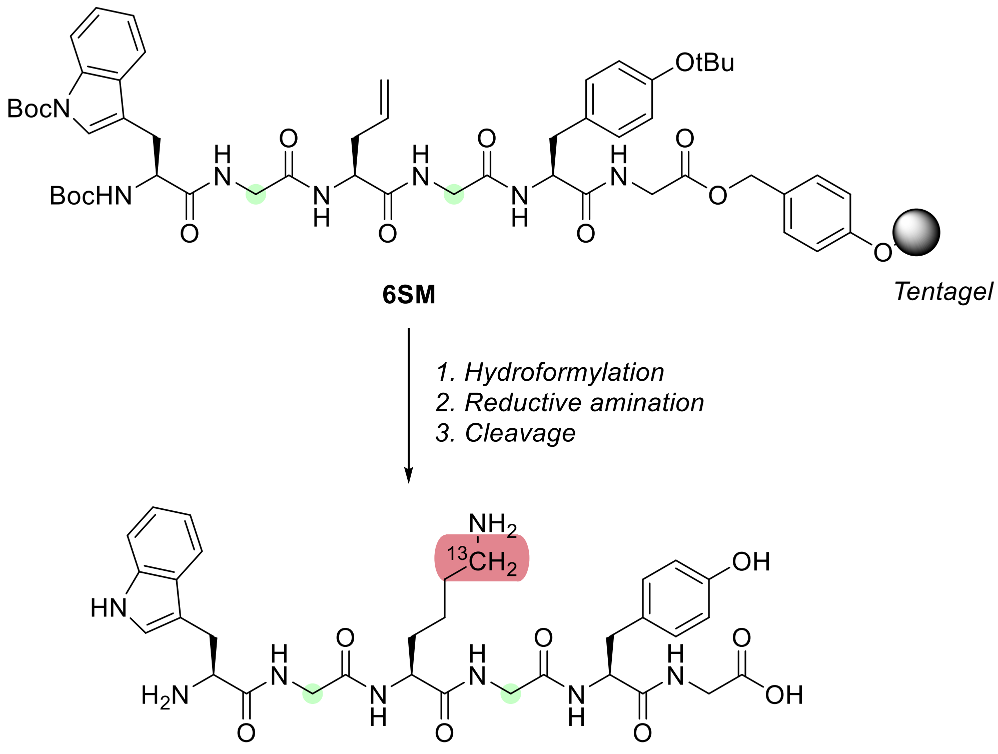

[^{13}C]6C
Chemical Formula: $C_{31}{}^{13}CH_{42}N_8O_8$
Exact Mass: 667.3159

The title compound was prepared following general procedure GP3B, employing resin-bound **6SM** (119 mg, ca. 25.5 µmol, 1 equiv., 0.214 mmol/g loading).

Reductive amination was performed according to general procedure GP6B, and was in total performed 1 time, followed by cleavage according to GP2B.

The crude product was purified by preparative HPLC (5% B for 1 min, 5–60% B for 17 min, 60–95% B for 1 min, 95–5% B for 1 min, 5% B for 1 min) to isolate **[^{13}C]6C** as a white solid (7.2 mg, with 2x TFA salt: 8 µmol, yield 31%, isotope incorporation +0.99 ^{13}C/molecule, 85% UV-purity by LC-MS (2 product isomer signals by UV, respectively 76% and 9%)).

NMR details: spectra attached, not in detail reported.
HRMS (ESI+) (m/z): $[M+H]^+$ calculated for $C_{31}{}^{13}CH_{43}N_8O_8{}^+$ 668.3232; found 668.3287.

9.1.7 Hexapeptide 7C

[^{12}C]7C

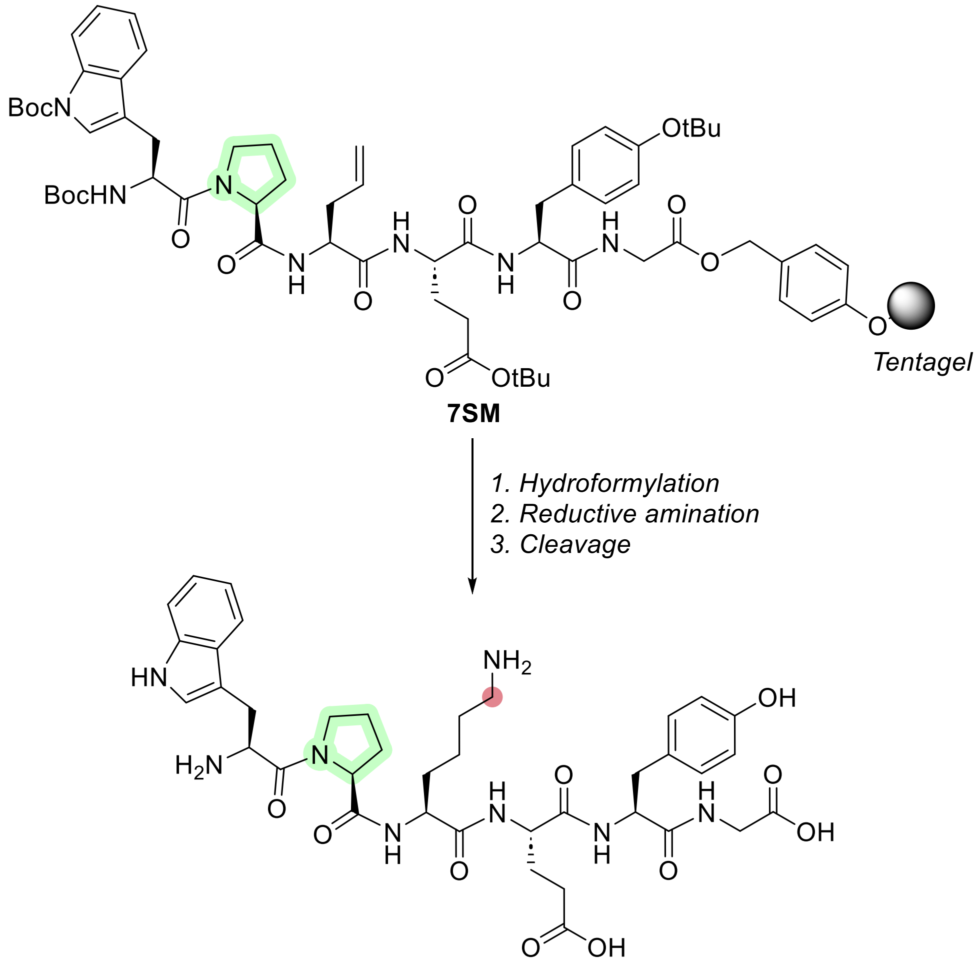

[^{12}C]7C

Chemical Formula: $C_{38}H_{50}N_8O_{10}$

Exact Mass: 778.3650

The title compound was prepared following general procedure GP3B, employing resin-bound **7SM** (205.5 mg, ca. 24.5 µmol, 1 equiv., 0.119 mmol/g loading).

Reductive amination was performed according to general procedure GP6B, and was in total performed 1 time, followed by cleavage according to GP2B.

The crude product was purified by preparative HPLC (5% B for 1 min, 5–50% B for 17 min, 50–95% B for 1 min, 95–5% B for 1 min, 5% B for 1 min) to isolate **[^{12}C]7C** as a white solid (9.8 mg, with 2x TFA salt: 9.7 µmol, yield 40%, 95% UV-purity by LC-MS (2–3x product isomer signals by UV, respectively 88%, 2% and 5%)).

NMR details: spectra attached, not in detail reported.

HRMS (ESI+) (m/z): $[M+H]^+$ calculated for $C_{38}H_{51}N_8O_{10}^+$ 779.3723; found 779.3749.

[^{13}C]7C

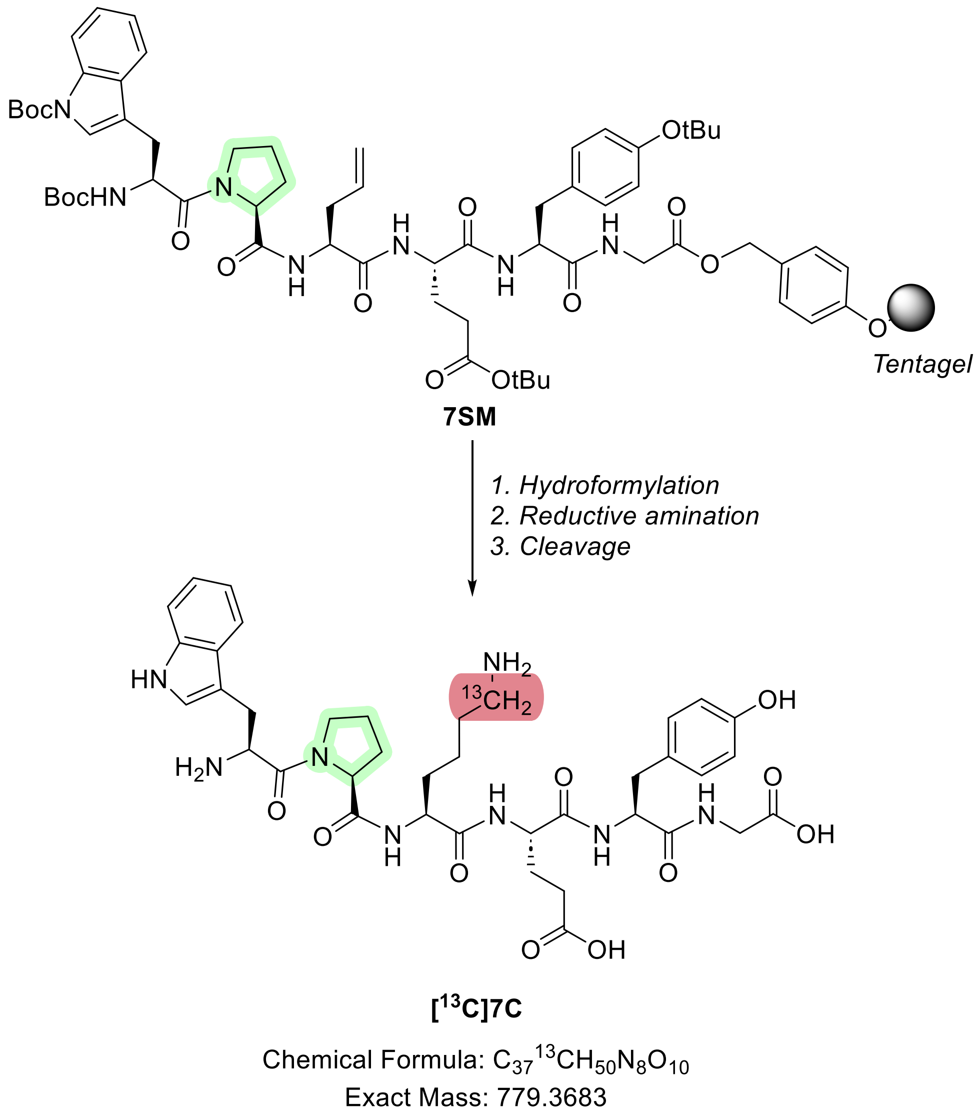

The title compound was prepared following general procedure GP3B, employing resin-bound **7SM** (205.5 mg, ca. 24.5 µmol, 1 equiv., 0.119 mmol/g loading).

Reductive amination was performed according to general procedure GP6B, and was in total performed 1 time, followed by cleavage according to GP2B.

The crude product was not purified but analyzed by LC-MS (isotope incorporation +0.99 ^{13}C/molecule, ca. 48% UV-purity (2x product peak, respectively 45% and 3%)).

NMR details: spectra attached, not in detail reported.

HRMS (ESI+) (m/z): $[M+H]^+$ calculated for $C_{37}{}^{13}CH_{51}N_8O_{10}^+$ 780.3756; found 780.3784.

9.1.8 Hexapeptide 8C

[^{12}C]8C

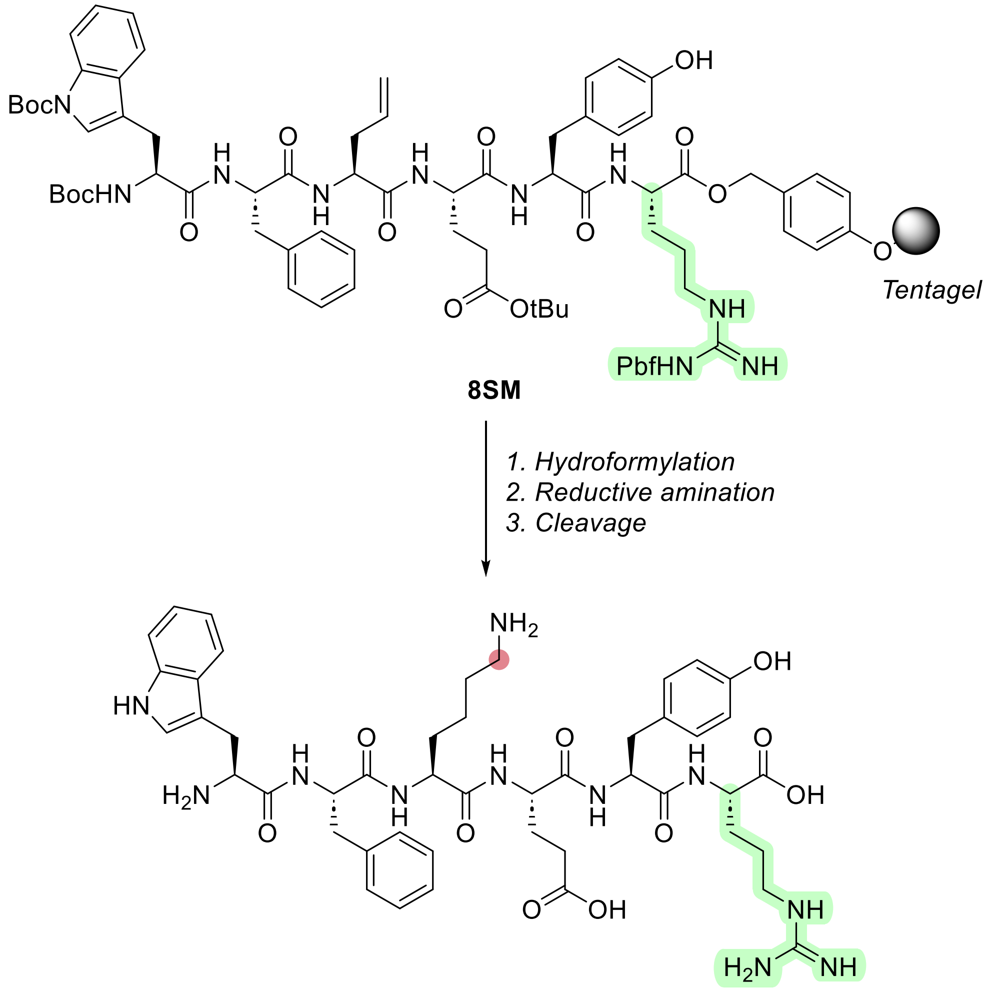

[^{12}C]8C

Chemical Formula: $C_{46}H_{61}N_{11}O_{10}$

Exact Mass: 927.4603

The title compound was prepared following general procedure GP3B, employing resin-bound **8SM** (186 mg, ca. 22.9 µmol, 1 equiv., 0.123 mmol/g loading).

Reductive amination was performed according to general procedure GP6B, and was in total performed 1 time, followed by cleavage according to GP2B.

The crude product was purified by preparative HPLC (5% B for 1 min, 5–50% B for 17 min, 50–95% B for 1 min, 95–5% B for 1 min, 5% B for 1 min) to isolate **[^{12}C]8C** as a white solid (14.5 mg, with 3x TFA salt: 11.4 µmol, yield 50%, 93% UV-purity by LC-MS).

NMR details: spectra attached, not in detail reported.

HRMS (ESI+) (m/z): $[M+H]^+$ calculated for $C_{46}H_{62}N_{11}O_{10}^+$ 928.4676; found 928.4689.

[^{13}C]8C

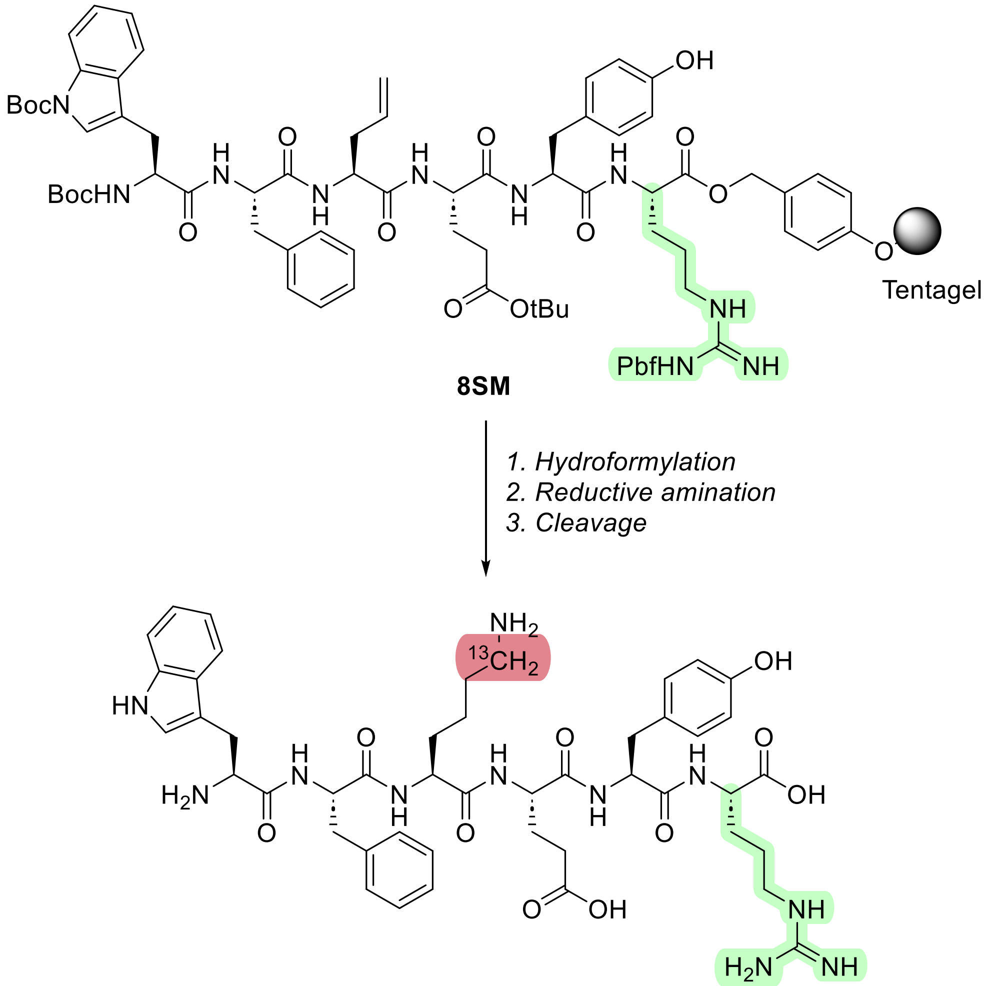

[^{13}C]8C

Chemical Formula: $C_{45}{}^{13}CH_{61}N_{11}O_{10}$
Exact Mass: 928.4636

The title compound was prepared following general procedure GP3B, employing resin-bound **8SM** (186 mg, ca. 22.9 µmol, 1 equiv., 0.123 mmol/g loading).

Reductive amination was performed according to general procedure GP6B, and was in total performed 1 time, followed by cleavage according to GP2B.

The crude product was purified by preparative HPLC (5% B for 1 min, 5–50% B for 17 min, 50–95% B for 1 min, 95–5% B for 1 min, 5% B for 1 min) to isolate **[^{13}C]8C** as a white solid (18 mg, with 3x TFA salt: 14.3 µmol, yield 62%, isotope incorporation +0.99 ^{13}C/molecule, 86% UV-purity by LC-MS (2 product isomer signals by UV, respectively 80% and 6%)).

NMR details: spectra attached, not in detail reported.

HRMS (ESI+) (m/z): $[M+H]^+$ calculated for $C_{45}{}^{13}CH_{62}N_{11}O_{10}{}^+$ 929.4709; found 929.4718.

9.1.9 Hexapeptide 9C

[^{12}C]9C

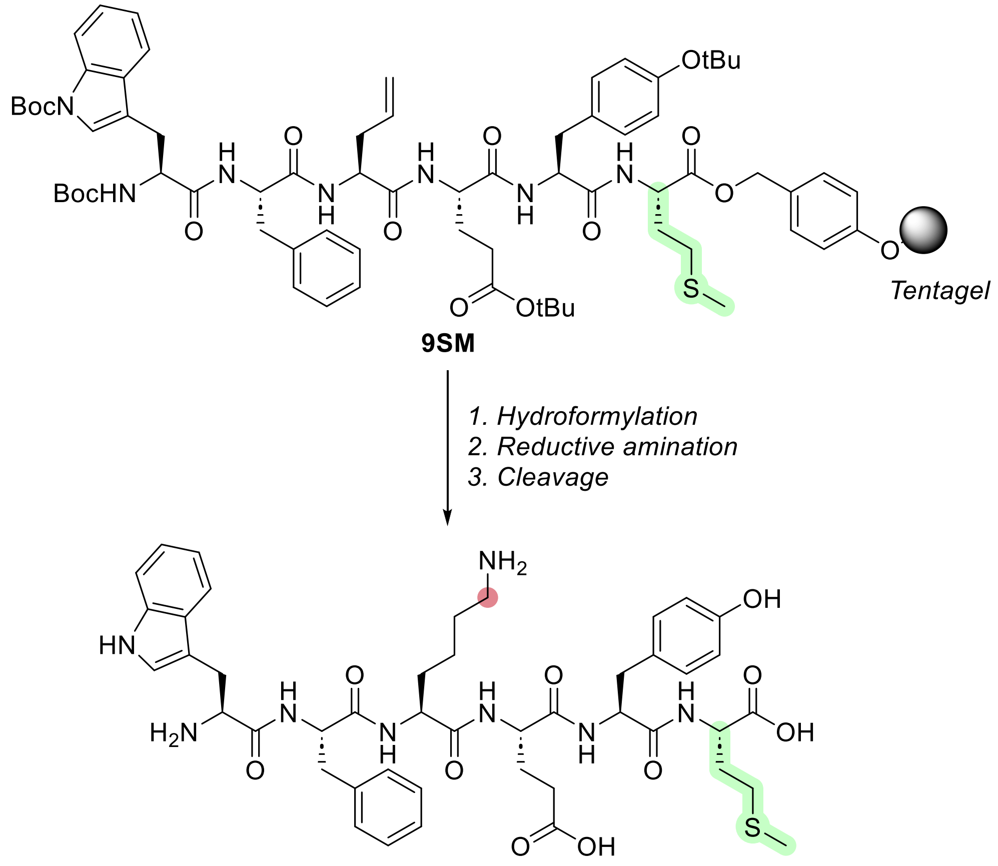

[^{12}C]9C

Chemical Formula: $C_{45}H_{58}N_8O_{10}S$

Exact Mass: 902.3997

The title compound was prepared following general procedure GP3B, employing resin-bound **9SM** (207 mg, ca. 23.6 µmol, 1 equiv., 0.114 mmol/g loading).

Reductive amination was performed according to general procedure GP6B, and was in total performed 1 time, followed by cleavage according to GP2B.

The crude product was purified by preparative HPLC (5% B for 1 min, 5–50% B for 17 min, 50–95% B for 1 min, 95–5% B for 1 min, 5% B for 1 min) to isolate **[^{12}C]9C** as a white solid (15.5 mg, with 2 x TFA salt: 13.7 µmol, yield 58%, 94% UV-purity by LC-MS (2 product isomer signals by UV, respectively 92% and 2%)).

NMR details: spectra attached, not in detail reported.

HRMS (ESI+) (m/z): $[M+H]^+$ calculated for $C_{45}H_{59}N_8O_{10}S^+$ 903.4069; found 903.4075.

[13C]9C

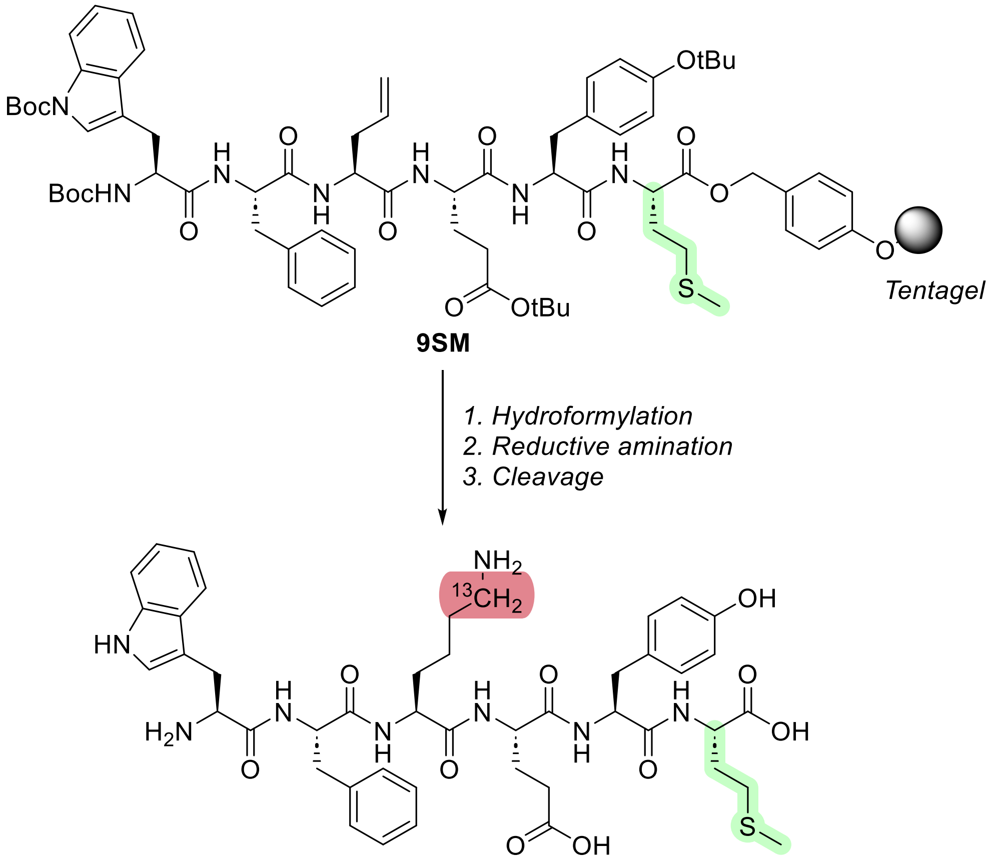

[13C]9C
Chemical Formula: $C_{44}{}^{13}CH_{58}N_8O_{10}S$
Exact Mass: 903.4030

The title compound was prepared following general procedure GP3B, employing resin-bound **9SM** (207 mg, ca. 23.6 µmol, 1 equiv., 0.114 mmol/g loading).

Reductive amination was performed according to general procedure GP6B, and was in total performed 1 time, followed by cleavage according to GP2B.

The crude product was purified by preparative HPLC (5% B for 1 min, 5–50% B for 17 min, 50–95% B for 1 min, 95–5% B for 1 min, 5% B for 1 min) to isolate **[13C]9C** as a white solid (8.4 mg, with 2x TFA salt: 7.4 µmol, yield 31%, isotope incorporation +0.99 ^{13}C/molecule, 95% UV-purity by LC-MS (2 product isomer signals by UV, respectively 92% and 3%)).

NMR details: spectra attached, not in detail reported.
HRMS (ESI+) (m/z): $[M+H]^+$ calculated for $C_{44}{}^{13}CH_{59}N_8O_{10}S^+$ 904.4103; found 904.4138.

9.1.10 Hexapeptide 10C

[^{12}C]10C

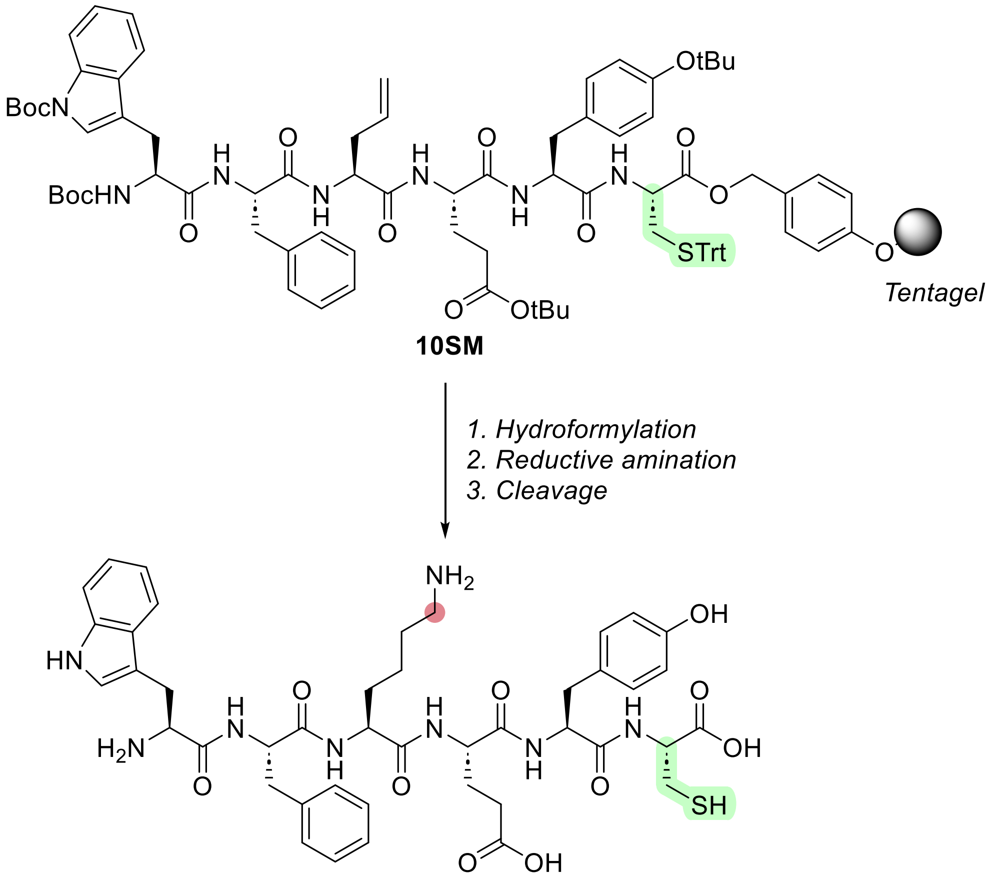

[^{12}C]10C

Chemical Formula: $C_{43}H_{54}N_8O_{10}S$

Exact Mass: 874.3684

The title compound was prepared following general procedure GP3B, employing resin-bound **10SM** (174 mg, ca. 24.9 µmol, 1 equiv., 0.143 mmol/g loading).

Reductive amination was performed according to general procedure GP6B, and was in total performed 1 time, followed by cleavage according to GP2B.

The crude product was purified by preparative HPLC (5% B for 1 min, 5–50% B for 17 min, 50–95% B for 1 min, 95–5% B for 1 min, 5% B for 1 min) to isolate **[^{12}C]10C** as a white solid (19 mg, with 2x TFA salt: 17.2 µmol, yield 69%, 91% UV-purity by LC-MS, (2 product isomer signals by UV, respectively 73% and 18%)).

NMR details: spectra attached, not in detail reported.

HRMS (ESI+) (m/z): $[M+H]^+$ calculated for $C_{43}H_{55}N_8O_{10}S^+$ 875.3756; found 875.3768.

[^{13}C]10C

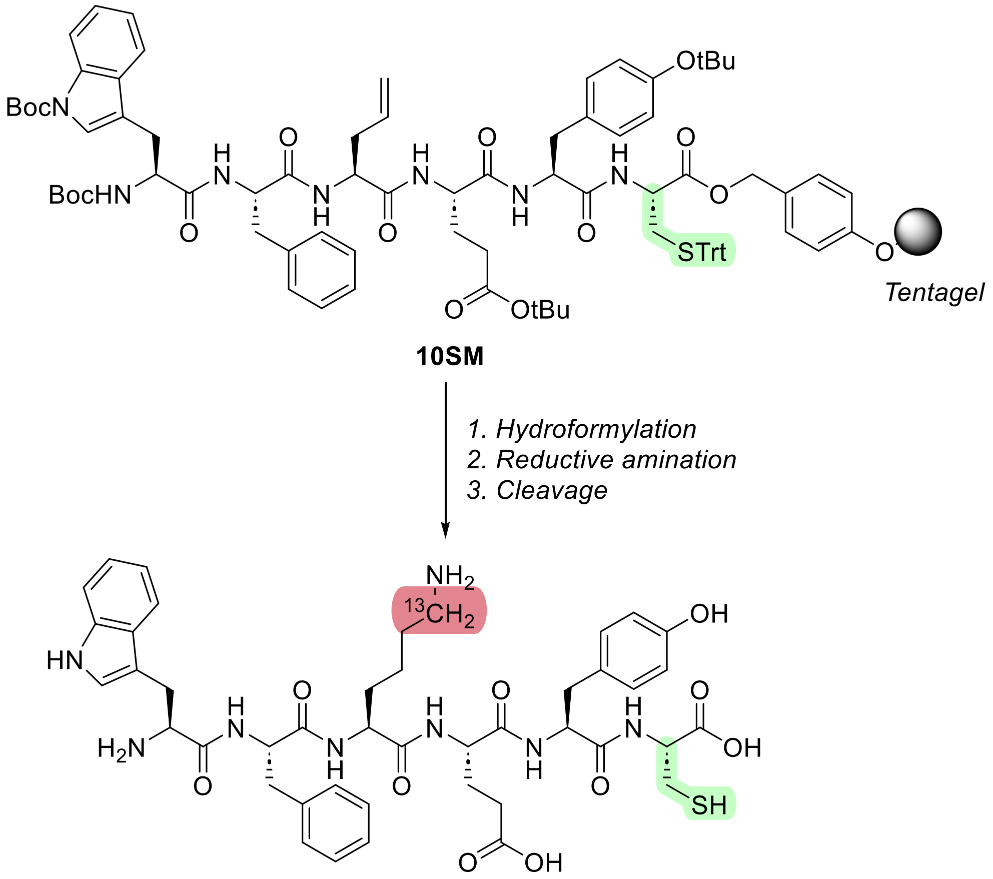

[^{13}C]10C

Chemical Formula: $C_{42}{}^{13}CH_{54}N_8O_{10}S$

Exact Mass: 875.3717

The title compound was prepared following general procedure GP3B, employing resin-bound **10SM** (174 mg, ca. 24.9 µmol, 1 equiv., 0.143 mmol/g loading).

Reductive amination was performed according to general procedure GP6B, and was in total performed 1 time, followed by cleavage according to GP2B.

The crude product was not purified but analyzed by LC-MS (58% UV-purity by LC-MS, (2–3 product isomer signals by UV, respectively 44%, 12% and 2%)).

HRMS of dimer, minor:
HRMS (ESI+) (m/z): $[M+H]^+$ calculated for $C_{84}{}^{13}C_2H_{107}N_{16}O_{20}S_2{}^+$ 1749.7351; found 1749.7356.
HRMS of dimer, major: ca. 1.98 ^{13}C/dimer incorporation
HRMS (ESI+) (m/z): $[M+2H]^{2+}$ calculated for $C_{84}{}^{13}C_2H_{108}N_{16}O_{20}S_2{}^{2+}$ 875.3712; found 875.3736.

9.1.11 11C (STAT3-Hel2A-2)

[^{12}C]11C (with DMSO:H_2O, 95:5)

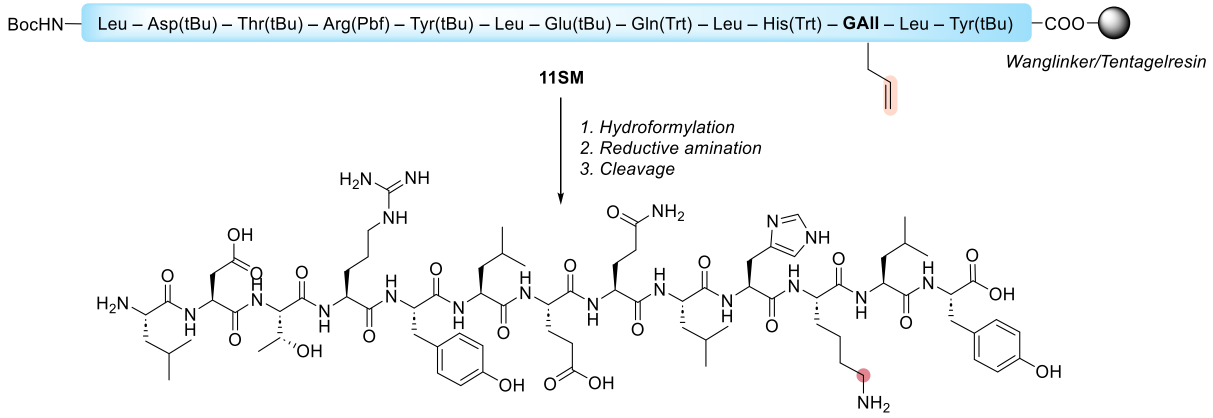

[^{12}C]11C
Chemical Formula: $C_{78}H_{122}N_{20}O_{22}$
Exact Mass: 1690.9043

The title compound was prepared following general procedure GP3B, employing resin-bound **11SM** (192 mg, ca. 25 µmol, 1 equiv., 0.13 mmol/g loading).

Reductive amination was performed according to general procedure GP6B, and was in total performed 1 time, followed by cleavage according to GP2B.

The crude product was purified by preparative HPLC (20% B for 1 min, 20–95% B for 17 min, 95% B for 1 min, 95–20% B for 1 min, 20% B for 1 min) to isolate **[^{12}C]11C** as a white solid (24.2 mg, with 4x TFA salt: 11.3 µmol, yield 45%, 78% UV-purity by LC-MS (2–3 product isomer signals by UV, respectively 74%, 3% and 1%)).

NMR details: spectra attached, not in detail reported.
HRMS: sample decomposed until measurement

[^{13}C]11C (with DMSO:H_2O, 95:5)

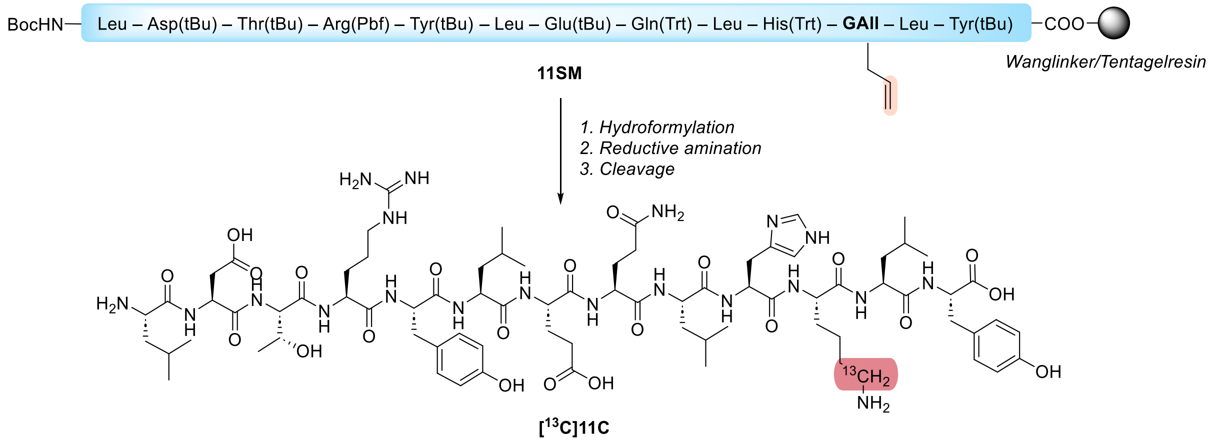

[^{13}C]11C
Chemical Formula: $C_{77}{}^{13}CH_{122}N_{20}O_{22}$
Exact Mass: 1691.9076

The title compound was prepared following general procedure GP3B, employing resin-bound **11SM** (187 mg, ca. 24.3 µmol, 1 equiv., 0.13 mmol/g loading).

Reductive amination was performed according to general procedure GP6B, and was in total performed 1 time, followed by cleavage according to GP2B.

The crude product was purified by preparative HPLC (20% B for 1 min, 20–95% B for 17 min, 95% B for 1 min, 95–20% B for 1 min, 20% B for 1 min) to isolate **[^{13}C]11C** as a white solid (17.5 mg, with 4x TFA salt: 8.2 µmol, yield 34%, 80% UV-purity by LC-MS (2 product isomer signals by UV, respectively 77% and 3%)).

NMR details: spectra attached, not in detail reported.
HRMS (ESI+) (m/z): $[M+2H]^{2+}$ calculated for $C_{77}{}^{13}CH_{124}N_{20}O_{22}{}^{2+}$ 846.9611; found 846.9603.

[^{13}C]11C (with neat DMSO)

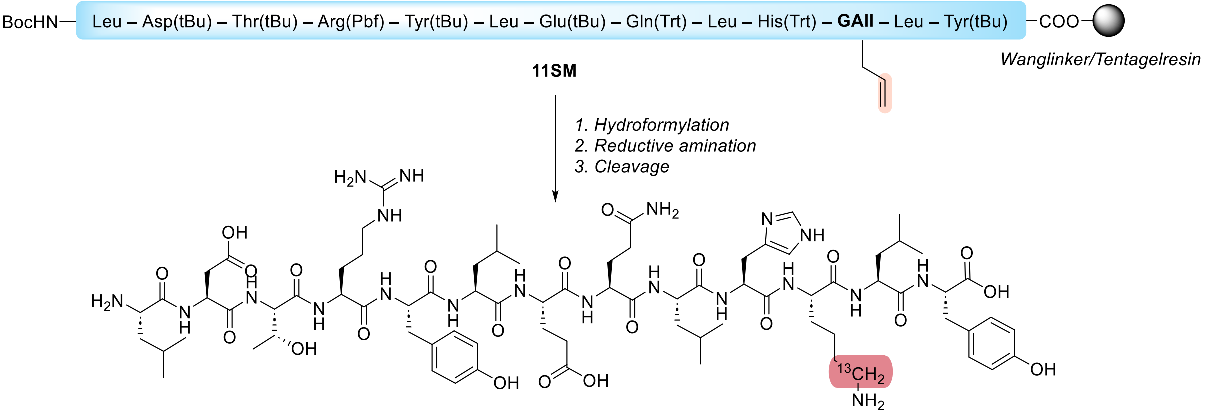

[^{13}C]11C
Chemical Formula: $C_{77}{}^{13}CH_{122}N_{20}O_{22}$
Exact Mass: 1691.9076

The title compound was prepared following general procedure GP3A, employing resin-bound **11SM** (154 mg, ca. 20 µmol, 1 equiv., 0.13 mmol/g loading) in DMSO (2 mL) in chamber B. The two-chamber reactor was shaken at 40 °C for 60 hours.

Reductive amination was performed according to general procedure GP6A, employing a solution of tritylamine in DMSO, 1% acetic acid (2 mL, 31 mg/mL, 0.12 mmol/mL) and a solution of $NaCNBH_3$ in CH_2Cl_2:MeOH, 3:1 (2 mL, 10 mg/mL, 0.15 mmol/mL), followed by cleavage according to GP2B.

The crude product was purified by preparative HPLC (gradient: 5% B for 3 min, 5–20% B for 2 min, 20–40% B for 15 min) to isolate **[^{13}C]11C** as a white solid (17.3 mg, with 4x TFA salt: 8 µmol, 40%, isotope incorporation +0.99 ^{13}C/molecule, 89% UV-purity by LC-MS).

NMR details: spectra attached, not in detail reported.
HRMS (ESI+) (m/z): $[M+2H]^{2+}$ calculated for $C_{77}{}^{13}CH_{124}N_{20}O_{22}{}^{2+}$ 846.9611; found 846.9633.

[^{2}H]11C (with neat DMSO) (not described in main manuscript)

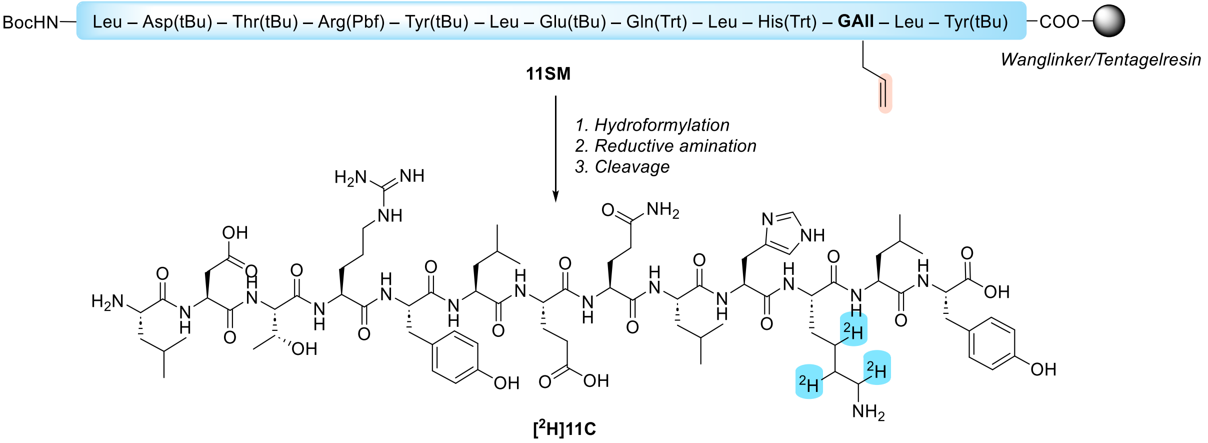

The title compound was prepared following general procedure GP5 (in two separate experiments), employing resin-bound **11SM** (34 mg, ca. 4.4 µmol, 1 equiv., 0.13 mmol/g loading), $Rh(COD)_2BF_4$ (2.8 mg, 6.9 µmol, 1.6 equiv.)/6-DPPon (10 mg, 36 µmol, 8.1 equiv.) in DMSO (1 mL) in chamber B, $^2H_2$-gas (280 mbar, ca. 0.2 mmol, 45 equiv., via manifold), and ^{12}COgen (49 mg, 0.2 mmol, 45 equiv.), $Pd(dba)_2$ (5.8 mg, 10 µmol), $P(tBu)_3$ (10 µL, 10 µmol, 1M in toluene), and DIPEA (52 µL, 0.3 mmol) in diglyme (1 mL, experiment 1) or toluene (1 mL, experiment 2). The modified two-chamber reactor was shaken at 40 °C for 60 hours.

Reductive amination was performed according to general procedure GP6A, employing a solution of tritylamine in DMSO, 1% acetic acid (2 mL, 31 mg/mL, 0.12 mmol/mL) and a solution of $NaCNBH_3$ in CH_2Cl_2:MeOH, 3:1 (2 mL, 10 mg/mL, 0.15 mmol/mL), followed by cleavage according to GP2B.

The crude LC-MS profile was very similar for both entries. They were therefore combined and the crude product was purified by preparative HPLC (gradient: 5% B for 3 min, 5–20% B for 2 min, 20–40% B for 15 min) to isolate **[^{2}H]11C** as a white solid (7.2 mg, with 4x TFA salt: 3.4 µmol, 38% (combined), isotope incorporation +1.15 ^{2}H/molecule, 97% UV-purity by LC-MS).

NMR details: spectra attached, not in detail reported.
HRMS (ESI+) (m/z): $[M+2H]^{2+}$ calculated for $C_{78}H_{124}N_{20}O_{22}^{2+}$ 846.4594; found 846.4605.

[^{3}H]11C (with neat DMSO)

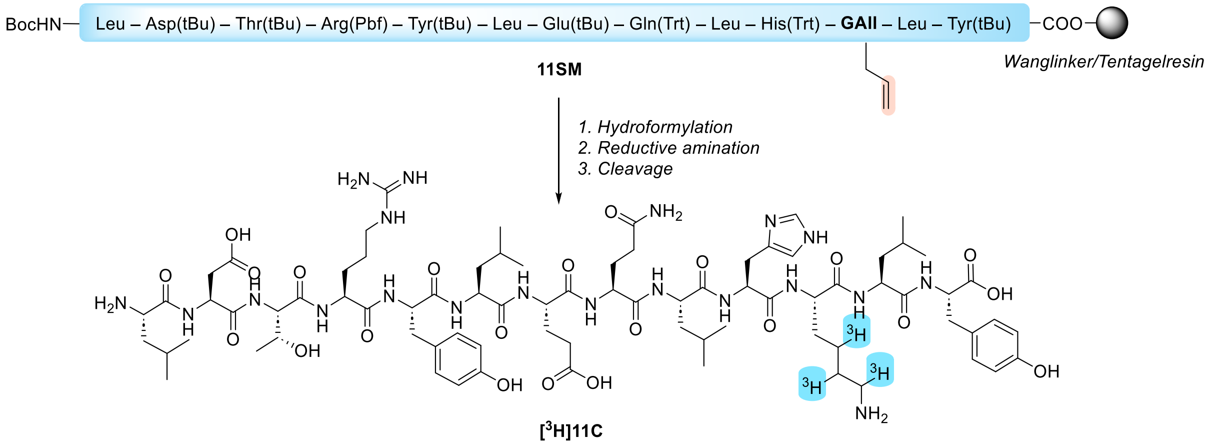

[^{3}H]11C
Chemical Formula: $C_{78}H_{119}T_3N_{20}O_{22}$
Exact Mass: 1696.9289

The title compound was prepared following general procedure GP5, employing resin-bound **11SM** (34 mg, ca. 4.4 µmol, 1 equiv., 0.13 mmol/g loading), $Rh(COD)_2BF_4$ (2.8 mg, 6.9 µmol, 1.6 equiv.)/6-DPPon (10 mg, 36 µmol, 8.1 equiv.) in DMSO (1 mL) in chamber B, $^3H_2$-gas (294 mbar, ca. 0.2 mmol, 45 equiv., via manifold), and ^{12}COgen (49 mg, 0.2 mmol, 45 equiv.), $Pd(dba)_2$ (5.8 mg, 10 µmol), P(tBu)$_3$ (10 µL, 10 µmol, 1M in toluene), and DIPEA (52 µL, 0.3 mmol) in diglyme (1 mL). The modified two-chamber reactor was shaken at 40 °C for 60 hours.

Reductive amination was performed according to general procedure GP6A, employing a solution of tritylamine in DMSO, 1% acetic acid (2 mL, 31 mg/mL, 0.12 mmol/mL) and a solution of $NaCNBH_3$ in CH_2Cl_2:MeOH, 3:1 (2 mL, 10 mg/mL, 0.15 mmol/mL). The reductive amination step was performed twice, followed by cleavage according to GP2B, affording the crude product (ca. 452 MBq).

The crude product was purified by preparative HPLC (gradient: 5% B for 3 min, 5–20% B for 2 min, 20–40% B for 15 min) to isolate **[^{3}H]11C** as a white solid (338 MBq, 0.84 ^{3}H/molecule, specific activity 24 Ci/mmol, 889 MBq/µmol, ca. 8% (0.33 µmol) yield calculated *via* radioactivity, 87% radiochemical purity).

NMR details: spectra attached, not in detail reported.
HRMS (ESI+) (m/z): $[M+2H]^{2+}$ calculated for $C_{78}H_{124}N_{20}O_{22}^{2+}$ 846.4594; found 846.4599.

[^{14}C]11C (with neat DMSO)

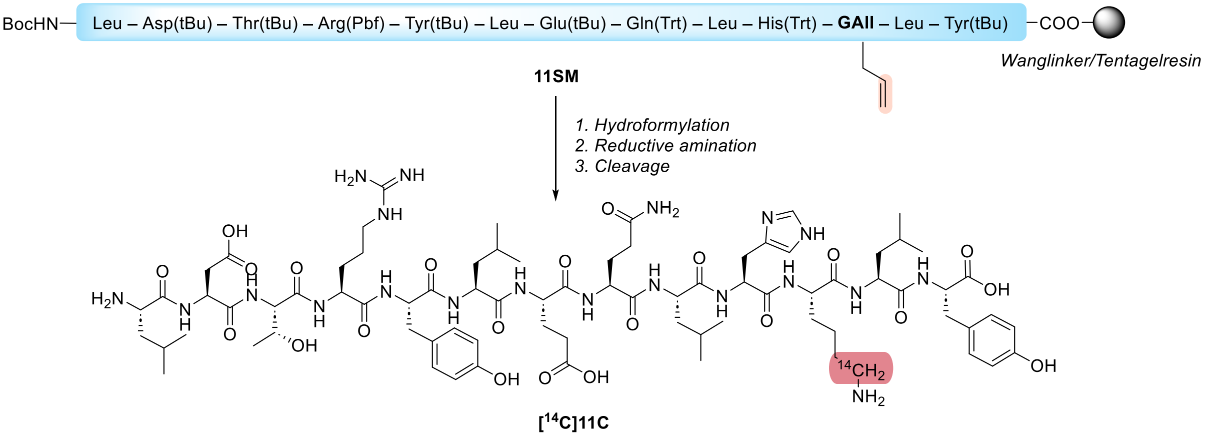

Chemical Formula: $C_{77}{}^{14}CH_{122}N_{20}O_{22}$
Exact Mass: 1692.9075

The title compound was prepared following general procedure GP4, employing resin-bound **11SM** (154 mg, 20 µmol, 1 equiv., 0.13 mmol/g loading), $Rh(COD)_2BF_4$ (7.5 mg, 18.5 µmol, 0.92 equiv.)/6-DPPon (27 mg, 97 µmol, 4.8 equiv.) in DMSO (2 mL) in chamber B, $^{1}H_2$-gas (2.5 mL, ca. 0.1 mmol, 5 equiv., via syringe), and ^{12}COgen:^{14}COgen (95:5, 24 mg, 0.1 mmol, 5 equiv., 10.95 MBq), $Pd(dba)_2$ (5.8 mg, 10 µmol), DIPEA (52 µL, 0.3 mmol), and tri-tert-butylphosphine (10 µL, 10 µmol, 1M in toluene) in diglyme (1 mL) in chamber A. The modified two-chamber reactor was shaken at 40 °C for 60 hours.

Reductive amination was performed according to general procedure GP6A, employing a solution of tritylamine in DMSO, 1% acetic acid (2 mL, 31 mg/mL, 0.12 mmol/mL) and a solution of $NaCNBH_3$ in CH_2Cl_2:MeOH, 3:1 (2 mL, 10 mg/mL, 0.15 mmol/mL). The reductive amination step was performed three times, followed by cleavage according to GP2B, affording the crude product (ca. 452 MBq).

Deviations of cleavage: A cleavage solution of TFA:H_2O:DODT:TiPS, 92.5:2.5:2.5:2.5 (1 mL) was added to the resin and the reaction mixture was shaken at room temperature for 3 hours, and filtered. After 3 h, Et_2O was added to the solution, and the precipitated peptide was washed 5x with Et_2O by precipitating *via* centrifugation for each washing step. DMSO (1.5 mL) was added for analysis. However, the Pbf protection group was partially not deprotected. Therefore, TFA:H_2O:DODT:TiPS, 92.5:2.5:2.5:2.5 (10 mL) was added and shaken for an additional 3 hours. Then, Et_2O was added to the solution, and the precipitated peptide was washed 5x with Et_2O, affording the crude product (1.425 MBq).

The crude product was purified by preparative HPLC (gradient: 5% B for 3 min, 5–20% B for 2 min, 20–40% B for 15 min) to isolate **[^{14}C]11C** as a white solid (0.598 MBq, ca. 0.05 ^{14}C/molecule, specific activity 127 MBq/mmol, 3.43 mCi/mmol, 5.5% RCY, 24% (4.7 µmol) yield calculated *via* radioactivity, >99% radiochemical purity, 96% UV-purity by LC-MS).
Note: After cleavage, acetone (2 mL) was added to the resin and the mixture was stirred at 50 °C overnight. Measurement on the scintillator shows that the total remaining resin has an activity of ca. 0.846 MBq. (not further investigated)

NMR details: spectra attached, not in detail reported.
HRMS (ESI+) (m/z): $[M+2H]^{2+}$ calculated for: $C_{78}H_{124}N_{20}O_{22}{}^{2+}$ 846.4594; found 846.4612.

9.1.12 **12C and 12Cb** (semaglutide analogs)

[^{13}C]**12Cb** (with BIPHEPHOS)

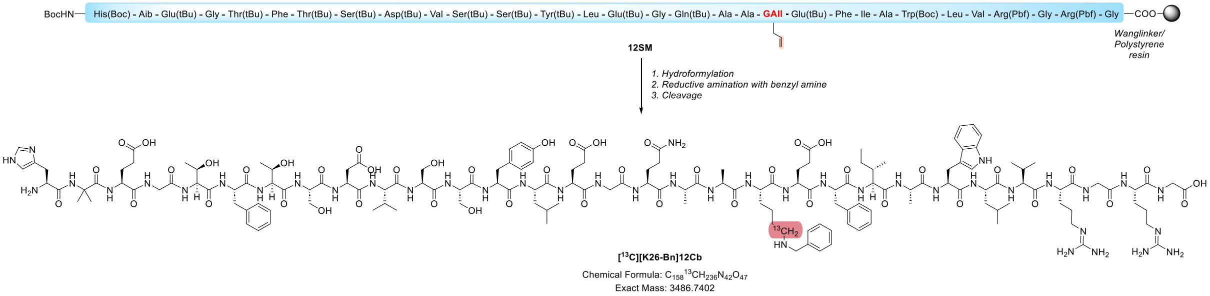

The title compound was prepared following general procedure GP3A, employing **12SM** (150 mg, ca. 12 µmol, 1 equiv., ca. 0.079 mmol/g loading, rather inexact due to impurities), $Rh(COD)_2BF_4$ (5.7 mg, 14 µmol, 1.2 equiv.)/BIPHEPHOS (28 mg, 36 µmol, 3 equiv.) in DMSO:THF, 17:3 (2 mL) in chamber B, and [^{13}C]-SilaCOgen (48 mg, 0.2 mmol, 17 equiv.) and $NaBH_4$ (8 mg, 0.2 mmol, 17 equiv.) in diglyme (1 mL) in chamber A. The two-chamber reactor was shaken at 60 °C for 60 hours.

Reductive amination was performed according to general procedure GP6C, employing a solution of benzylamine in DMF, 1% acetic acid (6 mL, 13 µL/mL, 0.12 mmol/mL) and a solution of $NaCNBH_3$ in CH_2Cl_2:MeOH, 3:1 (6 mL, 10 mg/mL, 0.15 mmol/mL), followed by cleavage according to GP2B.

The crude product was purified by preparative HPLC (gradient: 5% B for 3 min, 5–33% B for 5 min, 33–42% B for 19 min) to afford the desired peptide **[^{13}C]12Cb** (5 mg; with 5x TFA salt: 1.2 µmol, 11%, isotope incorporation +0.99 ^{13}C/molecule, 93% UV-purity by LC-MS) as a white solid.

NMR details: see other batch with 6-DPPon
HRMS (ESI+) (m/z): $[M+3H]^{3+}$ calculated for $C_{158}{}^{13}CH_{239}N_{42}O_{47}{}^{3+}$ 1163.2540; found 1163.2560.

[^{13}C]**12Cb** (not described in main manuscript) (with 6-DPPon)

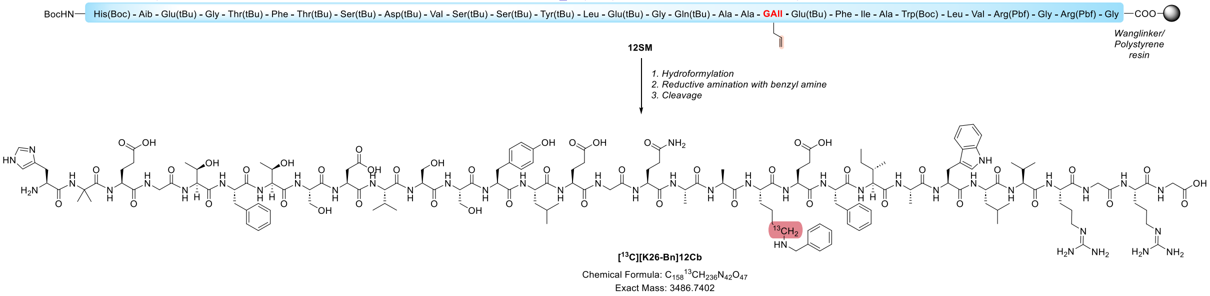

The title compound was prepared following general procedure GP3A, employing **12SM** (150 mg, ca. 12 µmol, 1 equiv., ca. 0.079 mmol/g loading, rather inexact due to impurities), $Rh(COD)_2BF_4$ (5.7 mg, 14 µmol, 1.2 equiv.)/6-DPPon (20 mg, 72 µmol, 6 equiv.) in DMSO (2 mL) in chamber B, and [^{13}C]-SilaCOgen (48 mg, 0.2 mmol, 17 equiv.) and $NaBH_4$ (8 mg, 0.2 mmol, 17 equiv.) in diglyme (1 mL) in chamber A. The two-chamber reactor was shaken at 60 °C for 60 hours.

Reductive amination was performed according to general procedure GP6C, employing a solution of benzylamine in DMF, 1% acetic acid (6 mL, 13 µL/mL, 0.12 mmol/mL) and a

solution of $NaCNBH_3$ in CH_2Cl_2:MeOH, 3:1 (6 mL, 10 mg/mL, 0.15 mmol/mL), followed by cleavage according to GP2B.

The crude product was purified by preparative HPLC (gradient: 5% B for 0.5 min, 5–33% B for 1.5 min, 33–38% B for 14 min, purification deviation: on Waters XBridge C18, 5 µm particle size, 19x150 mm column) to isolate **[^{13}C]12Cb** as a white solid (8.7 mg; with 5x TFA salt: 2.2 µmol, 18%, isotope incorporation +0.99 ^{13}C/molecule, 78% UV-purity by LC-MS).

NMR details: spectra attached, not in detail reported.
HRMS (ESI+) (m/z): $[M+3H]^{3+}$ calculated for $C_{158}{}^{13}CH_{239}N_{42}O_{47}{}^{3+}$ 1163.2540; found 1163.2556.

[^{3}H]12C (with BIPHEPHOS)

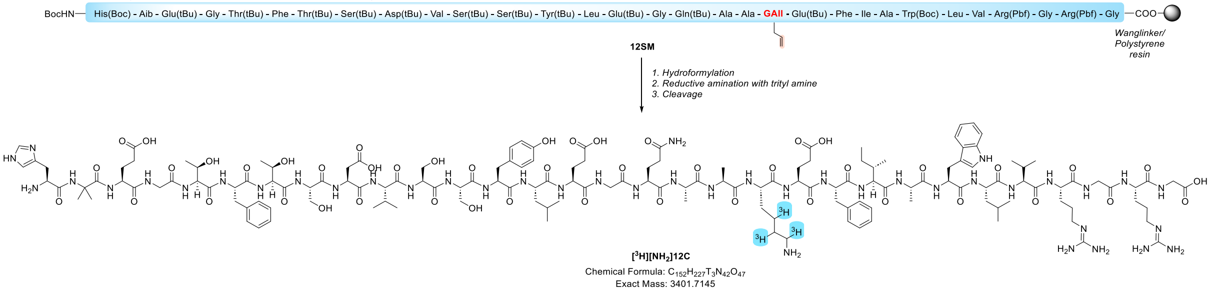

The title compound was prepared following general procedure GP5, employing **12SM** (150 mg, ca. 12 µmol, 1 equiv., ca. 0.079 mmol/g loading, rather inexact due to impurities), $Rh(COD)_2BF_4$ (5.8 mg, 14 µmol, 1.2 equiv.)/BIPHEPHOS (28 mg, 36 µmol, 3 equiv.) in DMSO:THF (17:3, 2 mL) in chamber B, $^3H_2$-gas (280 mbar, ca. 0.2 mmol, 17 equiv., via manifold), and ^{12}COgen (97 mg, 0.4 mmol, 34 equiv.), $Pd(dba)_2$ (11.4 mg, 20 µmol), $P(tBu)_3$ (0.020 mL, 20 µmol, 1 M in toluene), and DIPEA (105 µL, 0.6 mmol) in diglyme (2 mL) in chamber A. The modified two-chamber reactor was shaken at 60 °C for 60 hours.

Reductive amination was performed according to general procedure GP6A, employing a solution of tritylamine in DMSO, 1% acetic acid (2 mL, 31 mg/mL, 0.12 mmol/mL) and a solution of $NaCNBH_3$ in CH_2Cl_2:MeOH, 3:1 (2 mL, 10 mg/mL, 0.15 mmol/mL). The reductive amination procedure was performed three times, followed by cleavage according to GP2B, affording the crude product (1116 MBq).

The crude product was purified by preparative HPLC (gradient: 5% B for 3 min, 5–30% B for 5 min, 30–42% B for 19 min), to isolate **[^{3}H]12C** (86 MBq, isotope incorporation 0.68 ^{3}H/molecule, specific activity 20 Ci/mmol, 719 MBq/µmol, ca. 1% (0.07 µmol) yield calculated *via* radioactivity, 55% radiochemical purity, 45–54% UV-purity by LC-MS).

Note: After cleavage, acetone (2 mL) was added to the resin and the mixture was stirred at 50 °C overnight. Scintillation measurement revealed that the total remaining resin had an activity of ca. 465 MBq. (not further investigated)

NMR details: spectra attached, not in detail reported.
HRMS (ESI+) (m/z): $[M+3H]^{3+}$ calculated for $C_{152}H_{233}N_{42}O_{47}{}^{3+}$ 1132.9039, found: 1132.9060.

[^{2}H]12C (not described in main manuscript) (with BIPHEPHOS)

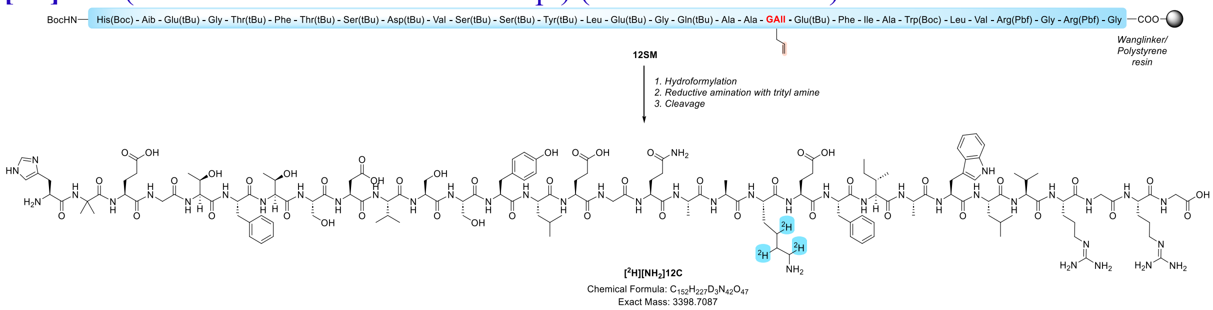

The title compound was prepared following general procedure GP5, employing **12SM** (76 mg, ca. 6 µmol, 1 equiv., ca. 0.079 mmol/g loading, rather inexact due to impurities), Rh(COD)$_2$BF$_4$ (5.8 mg, 14 µmol, 2.4 equiv.)/BIPHEPHOS (28 mg, 36 µmol, 6 equiv.) in DMSO:THF, 17:3 (2 mL) in chamber B, ^{2}H$_2$-gas (280 mbar, ca. 0.2 mmol, ca. 34 equiv., via manifold), and ^{12}COgen (97 mg, 0.4 mmol, 67 equiv.), Pd(dba)$_2$ (11.4 mg, 20 µmol), P(*t*Bu)$_3$ (0.020 mL, 20 µmol, 1 M in toluene), and DIPEA (105 µL, 0.6 mmol) in diglyme (2 mL) in chamber A. The modified two-chamber reactor was shaken at 60 °C for 60 hours.

Reductive amination was performed according to general procedure GP6A, employing a solution of tritylamine in DMSO, 1% acetic acid (3 mL, 31 mg/mL, 0.12 mmol/mL) and a solution of NaCNBH$_3$ in CH$_2$Cl$_2$:MeOH, 3:1 (3 mL, 10 mg/mL, 0.15 mmol/mL). The reductive amination procedure was performed three times, followed by cleavage according to GP2B.

The crude product was purified by preparative HPLC (gradient: 5% B for 3 min, 5–30% B for 5 min, 30–42% B for 19 min), to isolate **[^{2}H]12C** as a white solid (3.2 mg, with 5x TFA salt: 0.8 µmol, 13%, isotope incorporation +0.50 ^{2}H/molecule, 82% UV-purity by LC-MS).

NMR details: spectra attached, not in detail reported.
HRMS (ESI+) (m/z): [M+3H]$^{3+}$ calculated for $C_{152}H_{233}N_{42}O_{47}^{3+}$ 1132.9039, found: 1132.9042.

9.1.13 13G (somatostatin)

[^{12}C]13G (with DMSO:H$_2$O, 95:5)

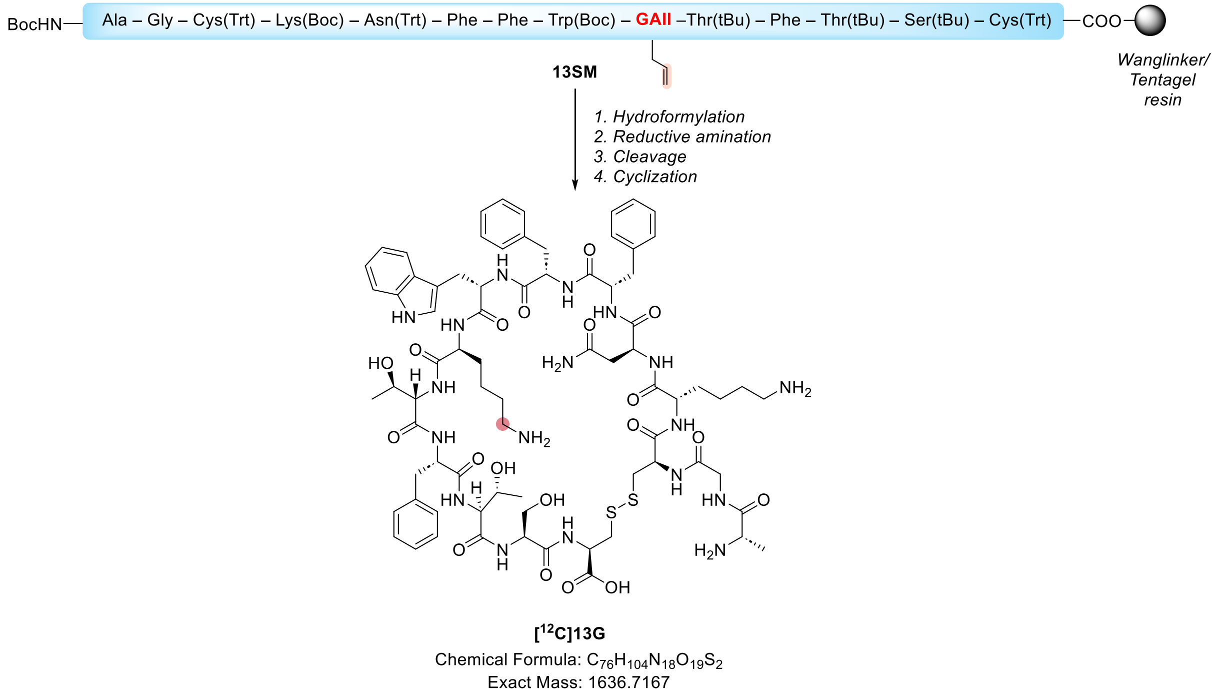

The title compound was prepared following general procedure GP3B, employing resin-bound **13SM** (230 mg, ca. 20.7 µmol, 1 equiv., 0.09 mmol/g loading).

Reductive amination was performed according to general procedure GP6B, and was in total performed 1 time, followed by cleavage according to GP2B.

After cleavage, an oxidative cyclization was performed: The peptide was dissolved in AcOH (100 µL) and H_2O (9 mL), and the pH was adjusted slowly with $(NH_4)_2CO_3$ to pH6. Then, ca. 10% DMSO by volume (1 mL) was added (total volume ca. 10 mL). The reaction mixture was vigorously stirred open to atmosphere for 5 days at RT to allow full oxidation, this was monitored by analytical LC-MS. Following, the reaction mixture was freeze-dried prior to purification.

The crude product was not purified but analyzed by LC-MS (53% UV-purity by LC-MS (2 product isomer signals by UV, respectively 47% and 6%)).

HRMS (ESI+) (m/z): $[M+H]^{2+}$ calculated for $C_{76}H_{106}N_{18}O_{19}S_2^{2+}$ 819.3656; found 819.3643.

[^{13}C]13G (with DMSO:H_2O, 95:5)

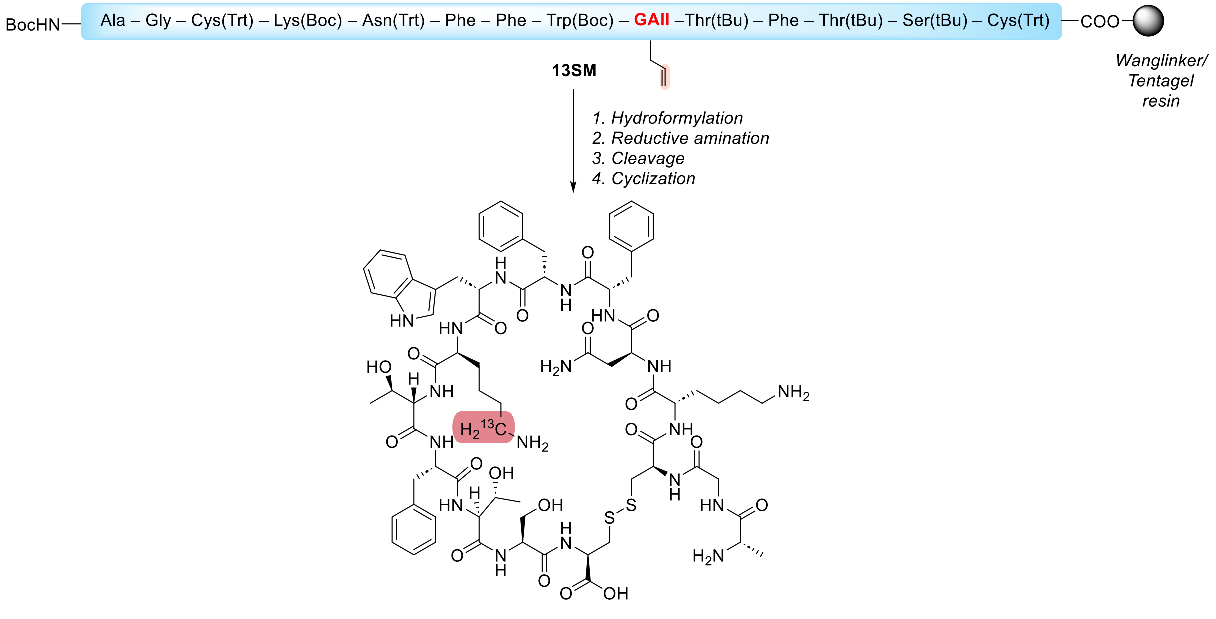

[^{13}C]13G

Chemical Formula: $C_{75}{}^{13}CH_{104}N_{18}O_{19}S_2$

Exact Mass: 1637.7200

The title compound was prepared following general procedure GP3B, employing resin-bound **13SM** (230 mg, ca. 20.7 µmol, 1 equiv., 0.09 mmol/g loading).

Reductive amination was performed according to general procedure GP6B, and was in total performed 1 time, followed by cleavage according to GP2B.

After cleavage, an oxidative cyclization was performed: The peptide was dissolved in AcOH (100 µL) and H_2O (9 mL), and the pH was adjusted slowly with $(NH_4)_2CO_3$ to pH6. Then, ca. 10% DMSO by volume (1 mL) was added (total volume ca. 10 mL). The reaction mixture was vigorously stirred open to atmosphere for 5 days at RT to allow full oxidation, this was

monitored by analytical LC-MS. Following, the reaction mixture was freeze-dried prior to purification.

The crude product was not purified but analyzed by LC-MS (44% UV-purity by LC-MS (2 product isomer signals by UV, respectively 39% and 5%).

HRMS (ESI+) (m/z): $[M+H]^+$ calculated for $C_{75}{}^{13}CH_{106}N_{18}O_{19}S_2{}^{2+}$ 819.8673; found 819.8666.

[^{13}C]13G

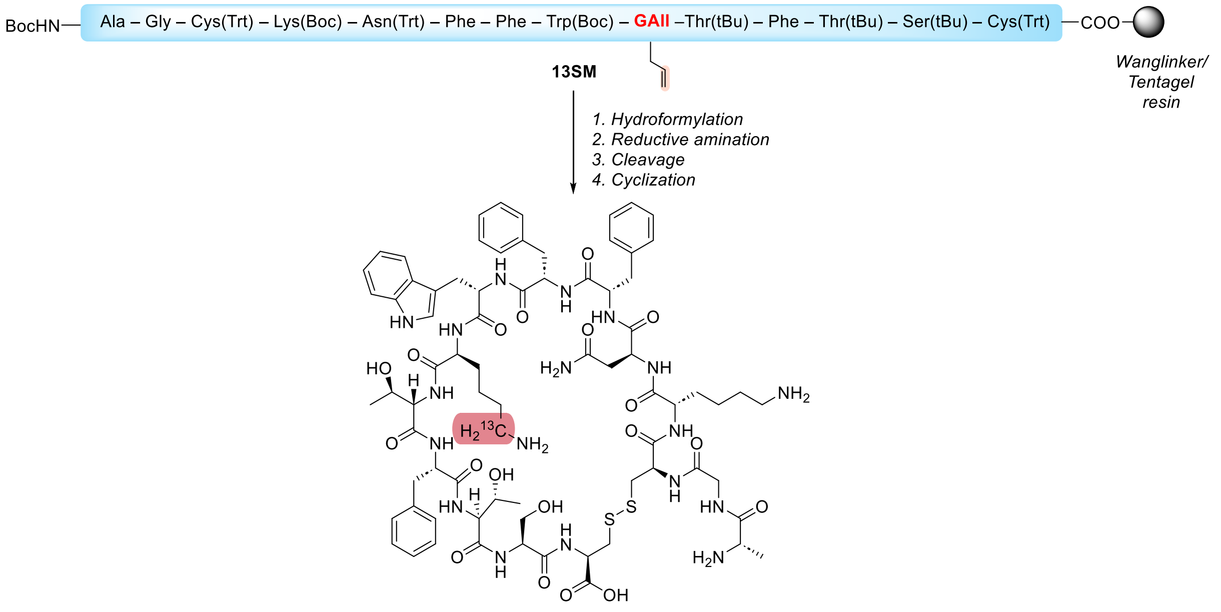

[^{13}C]13G

Chemical Formula: $C_{75}{}^{13}CH_{104}N_{18}O_{19}S_2$

Exact Mass: 1637.7200

The title compound was prepared following general procedure GP3A, employing resin-bound **13SM** (167 mg, ca. 15 µmol, 1 equiv., 0.09 mmol/g loading), in DMSO (2 mL) in chamber B. The two-chamber reactor was shaken at room temperature for 60 hours.

Thereafter, a reductive amination according to general procedure GP6A was performed, followed by cleavage according to GP2B.
<u>Oxidation-experiment:</u> 40% of the resin was subjected to reductive amination with tritylamine, following oxidation;
<u>No-oxidation-experiment:</u> 40% of the resin was subjected to reductive amination with tritylamine, with no oxidation.
Reductive amination was performed according to general procedure GP6A, employing a solution of tritylamine in DMSO, 1% acetic acid (2 mL, 31 mg/mL, 0.12 mmol/mL) and a solution of $NaCNBH_3$ in CH_2Cl_2:MeOH, 3:1 (2 mL, 10 mg/mL, 0.15 mmol/mL).

After cleavage, an oxidative cyclization was performed for the "oxidation-experiment": The pH of the peptide dissolved in an $AcOH/H_2O$ solution was adjusted slowly with $(NH_4)_2CO_3$ to pH6. Then, ca. 10% DMSO by volume were added (total volume ca. 10 mL). The reaction mixture was stirred open to atmosphere for 84 hours at room temperature to allow full oxidation. Following, the reaction mixture was freeze-dried prior to purification.

Oxidation-experiment: The crude product was purified by preparative HPLC (gradient: 5% B for 3 min, 5-30% B for 2 min, 30-40% B for 15 min) to isolate **[^{13}C]13G** as a white solid (1.3 mg; with 3x TFA salt: 1.3 mg, 0.66 µmol, 11% (the use of only 40% is calculated in the yield), isotope incorporation +0.99 ^{13}C/molecule, 90% UV-purity by LC-MS).

No-oxidation-experiment: The crude product was purified by preparative HPLC (gradient: 5% B for 3 min, 5-30% B for 2 min, 30-40% B for 15 min) to afford a mixture of **[^{13}C]13C** (oxidized) and **[^{13}C]13G** (non-oxidated) (1.4 mg; with 3x TFA salt: 1.3 mg, 0.71 µmol, 12% (the use of only 40% is calculated in the yield). The product was not further analyzed because it oxidizes leading to a mixture of both reduced and oxidated peptide.

HRMS of oxidated product **13G** (ESI+) (m/z): $[M+2H]^{2+}$ calculated for: $C_{75}{}^{13}CH_{106}N_{18}O_{19}S_2{}^{2+}$ 819.8673; found 819.8694.
NMR details: spectra attached, not in detail reported.

HRMS of non-oxidated product **13C** (ESI+) (m/z): $[M+2H]^{2+}$ calculated for: $C_{75}{}^{13}CH_{108}N_{18}O_{19}S_2{}^{2+}$, 820.8751; found 819.8781.

9.1.14 14G (terlipressin)

[^{12}C]14G

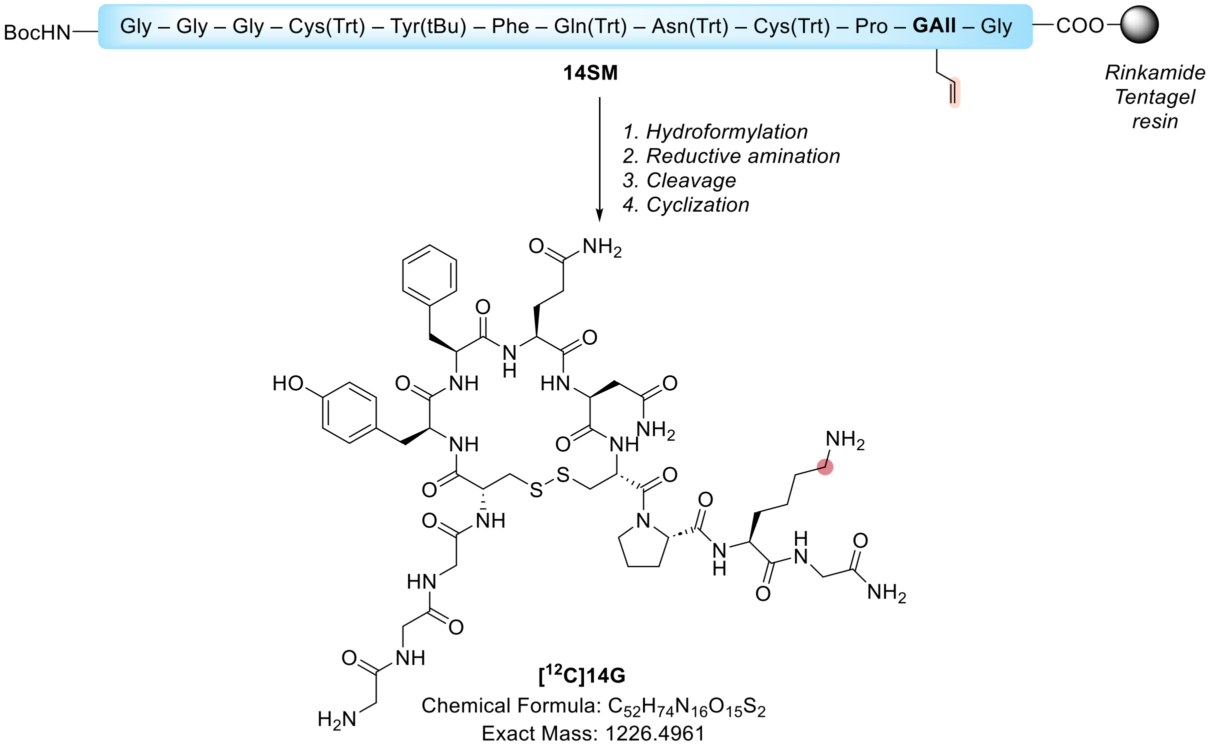

The title compound was prepared following general procedure GP3B, employing resin-bound **14SM** (254 mg, ca. 16 µmol, 1 equiv., 0.063 mmol/g loading).

Reductive amination was performed according to general procedure GP6B, and was in total performed 1 time, followed by cleavage according to GP2B.

After cleavage, an oxidative cyclization was performed: The peptide was dissolved in AcOH (100 µL) and H_2O (9 mL), and the pH was adjusted slowly with $(NH_4)_2CO_3$ to pH 6. Then, ca. 10% DMSO by volume (1 mL) was added (total volume ca. 10 mL). The reaction mixture was

vigorously stirred open to atmosphere for 5 days at RT to allow full oxidation, this was monitored by analytical LC-MS. Following, the reaction mixture was freeze-dried prior to purification.

The crude product was purified by preparative HPLC (5% B for 1 min, 5–35% B for 11 min, 35–95% B for 3 min, 95–5% B for 1 min, 5% B for 1 min) to isolate **[^{12}C]14G** as a white solid (10.9 mg, with 2x TFA salt: 7.5 µmol, yield 47%, 72% UV-purity by LC-MS (2 product isomer signals by UV, respectively 69% and 3%)).

NMR details: spectra attached, not in detail reported.
HRMS (ESI+) (m/z): $[M+H]^+$ calculated for $C_{52}H_{75}N_{16}O_{15}S_2^+$ 1227.5034; found 1227.5088.

[^{13}C]14G

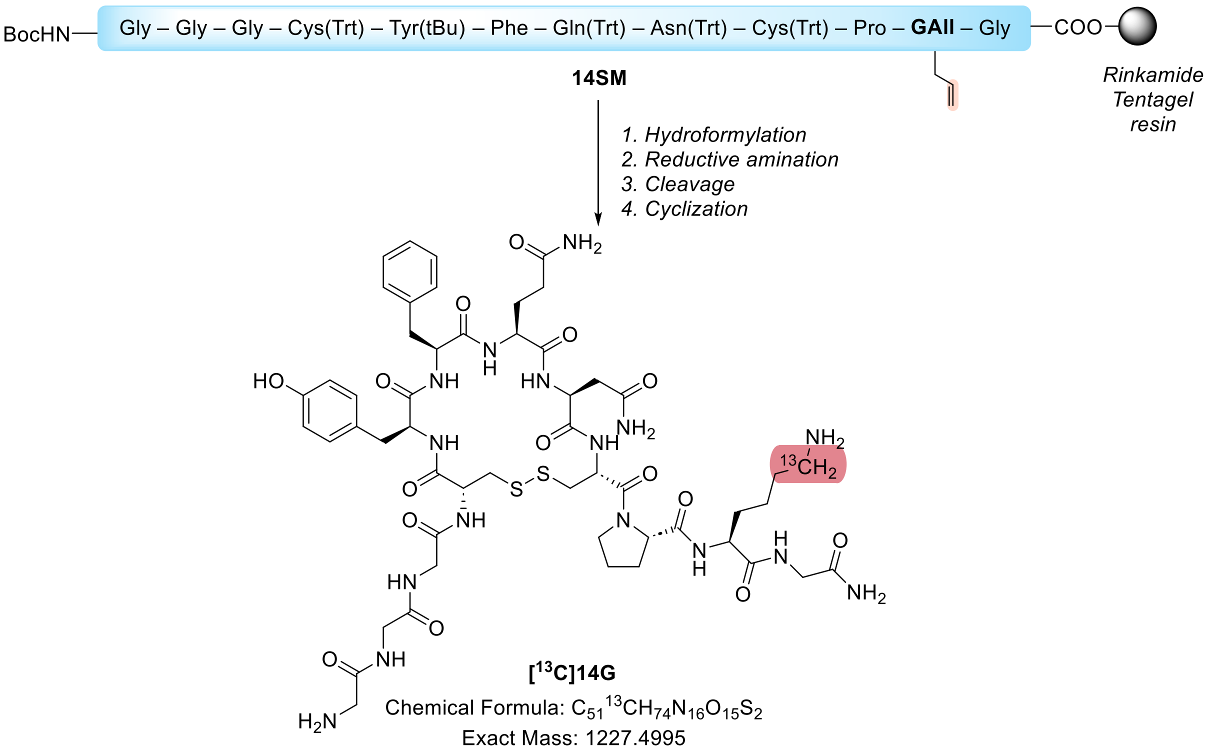

The title compound was prepared following general procedure GP3B, employing resin-bound **14SM** (220 mg, ca. 14 µmol, 1 equiv., 0.063 mmol/g loading).

Reductive amination was performed according to general procedure GP6B, and was in total performed 1 time, followed by cleavage according to GP2B.

After cleavage, an oxidative cyclization was performed: The peptide was dissolved in AcOH (100 µL) and H_2O (9 mL), and the pH was adjusted slowly with $(NH_4)_2CO_3$ to pH6. Then, ca. 10% DMSO by volume (1 mL) was added (total volume ca. 10 mL). The reaction mixture was vigorously stirred open to atmosphere for 5 days at RT to allow full oxidation, this was monitored by analytical LC-MS. Following, the reaction mixture was lyophilized prior to purification.

The crude product was purified by preparative HPLC (5% B for 1 min, 5–35% B for 11 min, 35–95% B for 3 min, 95–5% B for 1 min, 5% B for 1 min) to isolate **[^{13}C]14G** as a white solid (8.0 mg, with 2 x TFA salt: 5.5 µmol, yield 40%, isotope incorporation

+0.99 ^{13}C/molecule, 73% UV-purity by LC-MS (2 product isomer signals by UV, respectively 70% and 3%)).

NMR details: spectra attached, not in detail reported.

HRMS (ESI+) (m/z): $[M+H]^+$ calculated for $C_{51}{}^{13}CH_{75}N_{16}O_{15}S_2^+$ 1228.5067; found 1228.5094. Note: the sample for HRMS was very impure, and side products from decomposition did not reveal a nice isotope pattern of the product as they had an isotope shift of ca 1 Dalton.

The side product could be the deaminated peptide (Gln → Glu, or Asn →Asp): **HRMS** (ESI+) (m/z): $[M+H]^+$ calculated for $C_{51}{}^{13}CH_{74}N_{15}O_{16}S_2^+$ 1229.4907; found 1229.4937. This was however not further investigated, and should be rather unlikely to occur in an LC-MS-vial in the freezer, stored under slight acidic conditions.

9.1.15 **15B** (BIO-11006-analog thioacetal) **and 15A'a, 15A'b, and 15D** (cyclized BIO-11006 analogs)

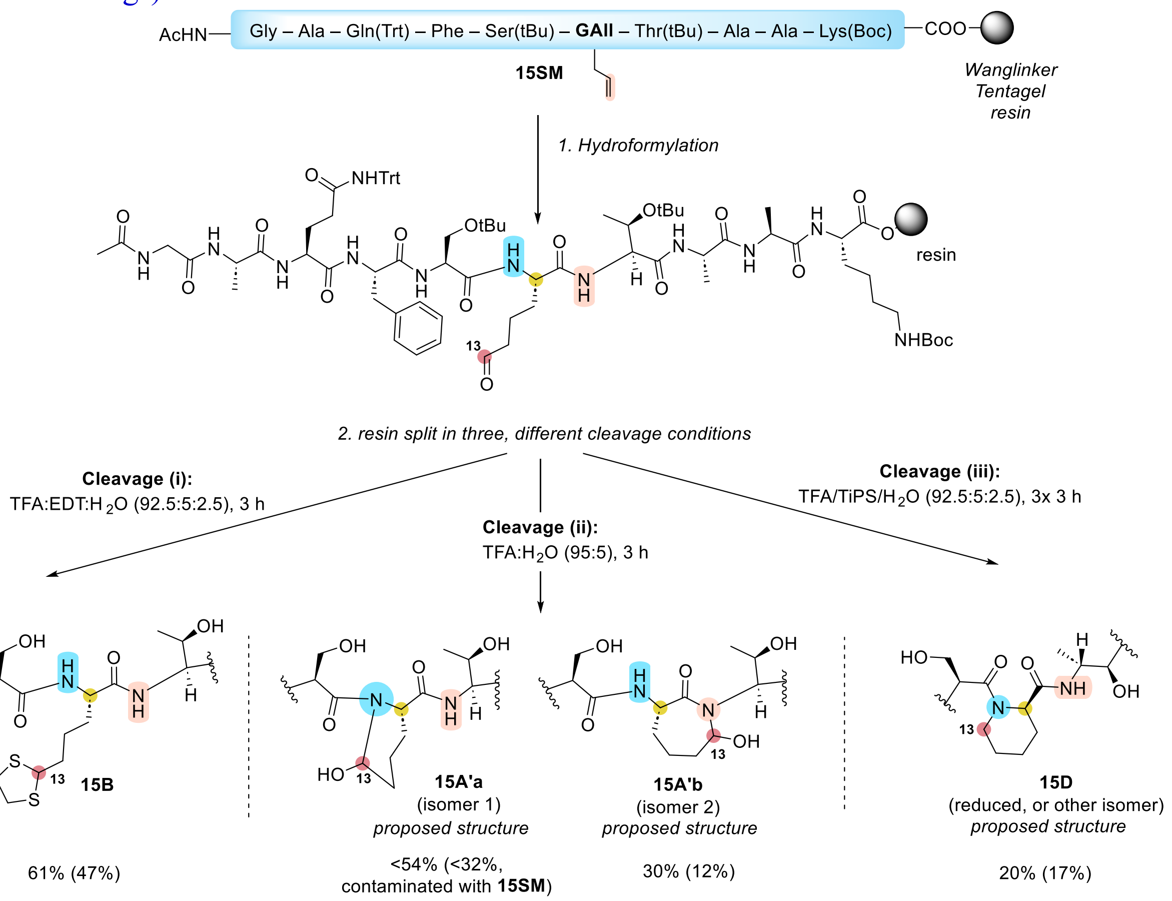

The title compounds were prepared following general procedure GP3A, employing **15SM** (142 mg, ca. 21.3 µmol, 1 equiv., 0.15 mmol/g loading) in DMSO (2 mL) in chamber B. The two-chamber reactor was shaken at RT for 60 hours.

No reductive amination was performed. Instead, the resin was split into three samples and each submitted to a different cleavage procedure:

Cleavage (i): 1/3rd of the resin was shaken in a solution of TFA:EDT:H_2O (92.5:5:2.5) for 3 hours. The crude product was purified by preparative HPLC (gradient: 5% B for 3 min, 5-15% B for 2 min, 15-22% B for 15 min, 22-50% B for 4 min) to isolate **15B** as a white solid

(4.1 mg; with 1x TFA salt: 3.3 µmol, 47%, isotope incorporation +0.99 ^{13}C/molecule, 99% UV-purity by LC-MS).

Cleavage (ii): 1/3rd of the resin was shaken in a solution of TFA:H_2O (95:5) for 3 hours. The crude product was purified by preparative HPLC (gradient: 5% B for 3 min, 5–14% B for 2 min, 14–20% B for 15 min) to isolate **15A'a** as a white solid (2.6 mg: with 1x TFA salt: <2.3 µmol, <32%, isotope incorporation +0.99 ^{13}C/molecule, coelutes with starting material), and to isolate **15A'b** as a white solid (1 mg, with 1x TFA salt: 0.9 µmol, 12%, isotope incorporation +0.99 ^{13}C/molecule, >99% UV-purity by LC-MS).

Cleavage (iii): 1/3rd of the resin was shaken in a solution of TFA:TiPS:H_2O (95:2.5:2.5) for 3 hours. This procedure was performed in total three times until almost complete reduction was observed for (only) one of the isomers. The crude product was purified by preparative HPLC (gradient: 5% B for 3 min, 5–14% B for 2 min, 14–20% B for 15 min) to afford the desired peptide **15D** (1.4 mg; with 1x TFA salt: 1.2 µmol, 17%, isotope incorporation +0.99 ^{13}C/molecule, 88% UV-purity by LC-MS) as a white solid.

15B: *Cleavage (i)*

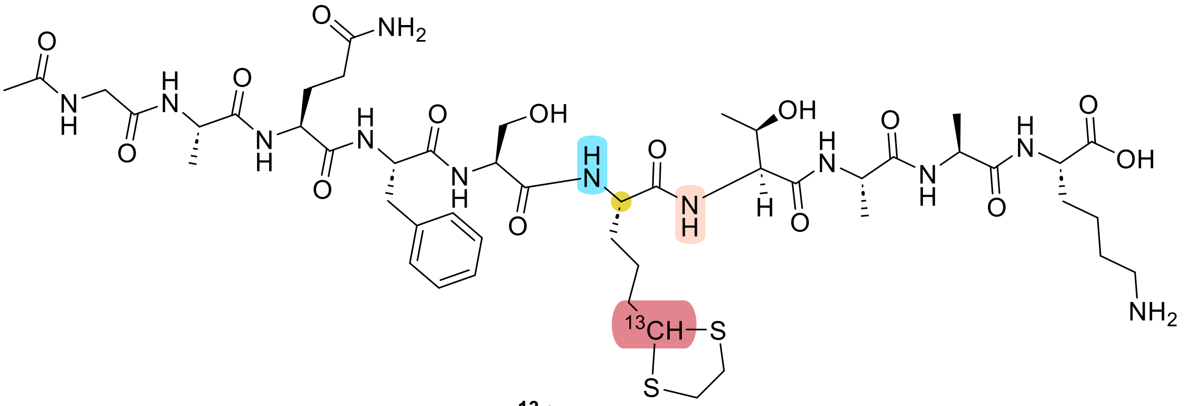

[^{13}C]15B

Chemical Formula: $C_{47}{}^{13}CH_{76}N_{12}O_{15}S_2$

Exact Mass: 1125.5028

NMR details: spectra attached, not in detail reported.

HRMS (ESI+) (m/z): $[M+H]^+$ calculated for $C_{47}{}^{13}CH_{77}N_{12}O_{15}S_2^+$ 1126.5101; found 1126.5105.

15A’a and **15A’b**: *Cleavage (ii)*

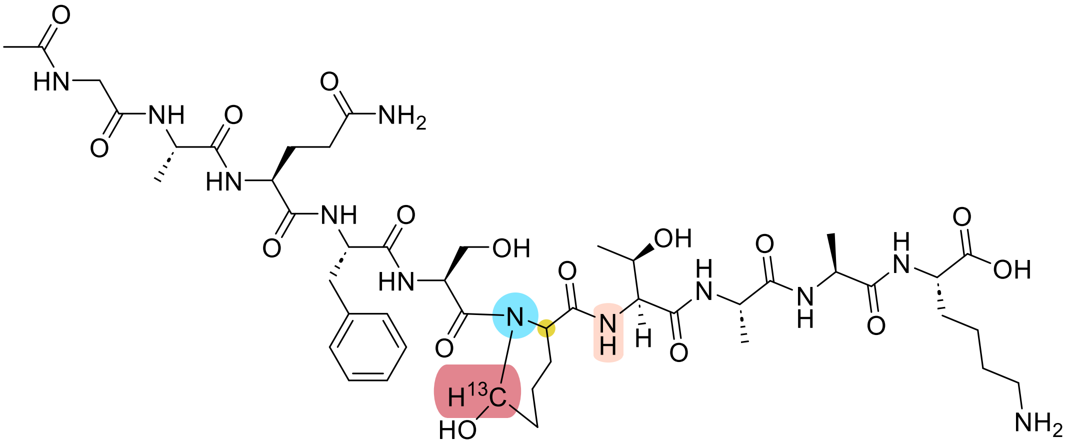

[^{13}C]15A'a

Chemical Formula: $C_{45}{}^{13}CH_{72}N_{12}O_{16}$

Exact Mass: 1049.5223

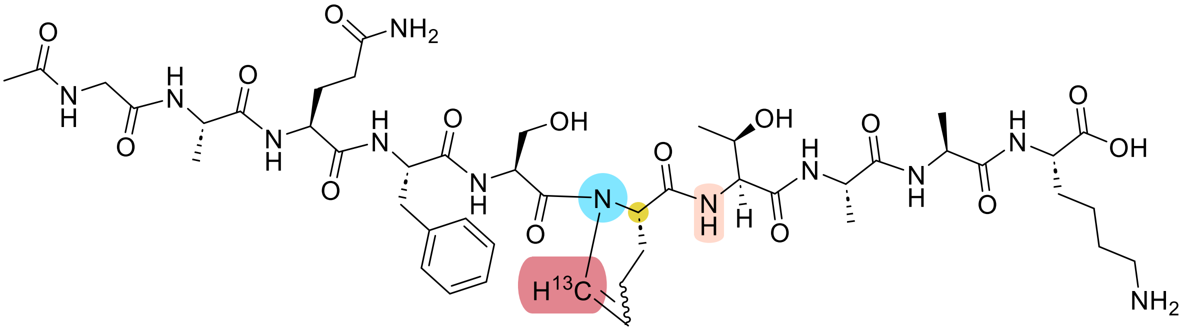

[^{13}C]15A'a

Chemical Formula: $C_{45}{}^{13}CH_{70}N_{12}O_{15}$

Exact Mass: 1031.5117

15A’a: *Cleavage (ii),* isomer 1, mass of enamine (contaminated with starting material due to co-elution)

NMR details: spectra attached, not in detail reported.

HRMS (ESI+) (m/z): $[M+H]^+$ calculated for $C_{45}{}^{13}CH_{71}N_{12}O_{15}{}^+$ 1032.5190; found 1032.5188.

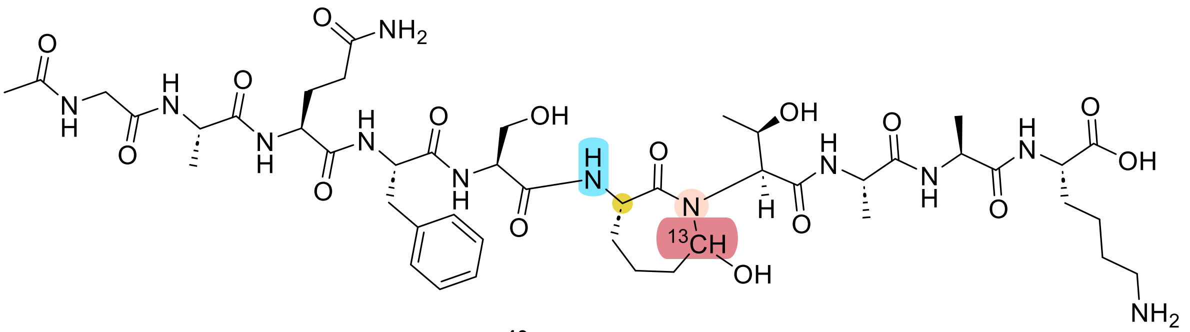

[^{13}C]15A'b
Chemical Formula: $C_{45}{}^{13}CH_{72}N_{12}O_{16}$
Exact Mass: 1049.5223

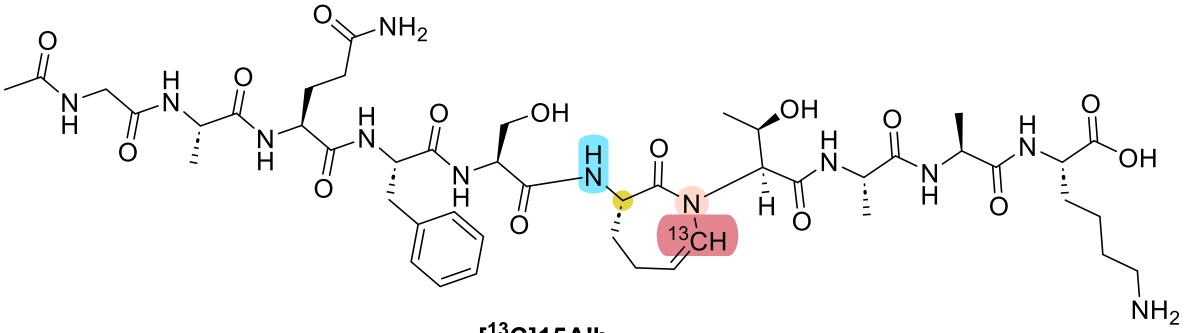

[^{13}C]15A'b
Chemical Formula: $C_{45}{}^{13}CH_{70}N_{12}O_{15}$
Exact Mass: 1031.5117

NMR details: spectra attached, not in detail reported.
15A'b: *Cleavage (ii),* isomer 2, mass of enamine
HRMS (ESI+) (m/z): $[M+H]^+$ calculated for $C_{45}{}^{13}CH_{71}N_{12}O_{15}{}^+$ 1032.5190; found 1032.5200.

15D: *Cleavage (iii)*

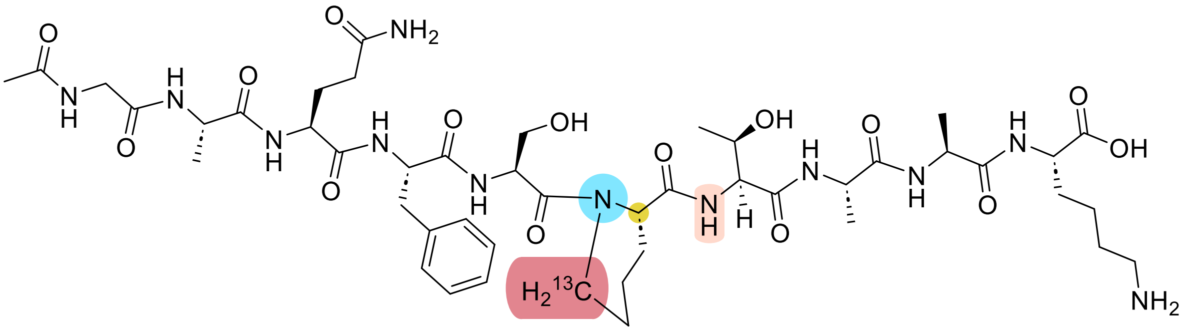

[^{13}C]15D
Chemical Formula: $C_{45}{}^{13}CH_{72}N_{12}O_{15}$
Exact Mass: 1033.5274

NMR details: spectra attached, not in detail reported.
HRMS (ESI+) (m/z): $[M+H]^+$ calculated for $C_{45}{}^{13}CH_{73}N_{12}O_{15}{}^+$ 1034.5346; found 1034.5372.

10.NMR spectra, UPLC-UV, and isotope patterns

10.1 NMR/LC-MS spectra of precursor peptides

10.1.1 SI-3

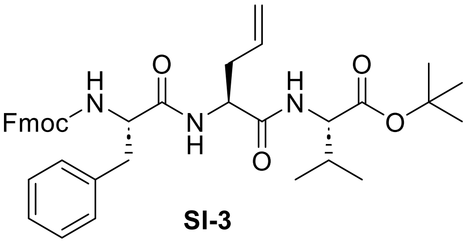

SI-3

Chemical Formula: $C_{38}H_{45}N_3O_6$
Exact Mass: 639.3308

LC-MS of purified product: ca. >99% UV-purity

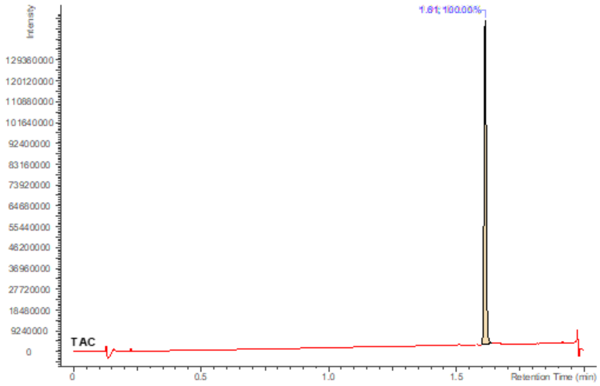

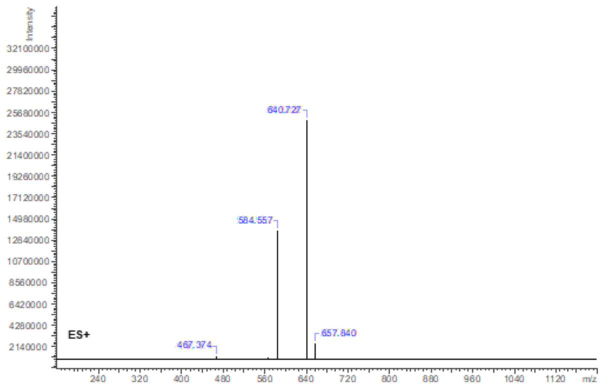

NMR:

^{1}H-NMR (500 MHz, DMSO-d6)

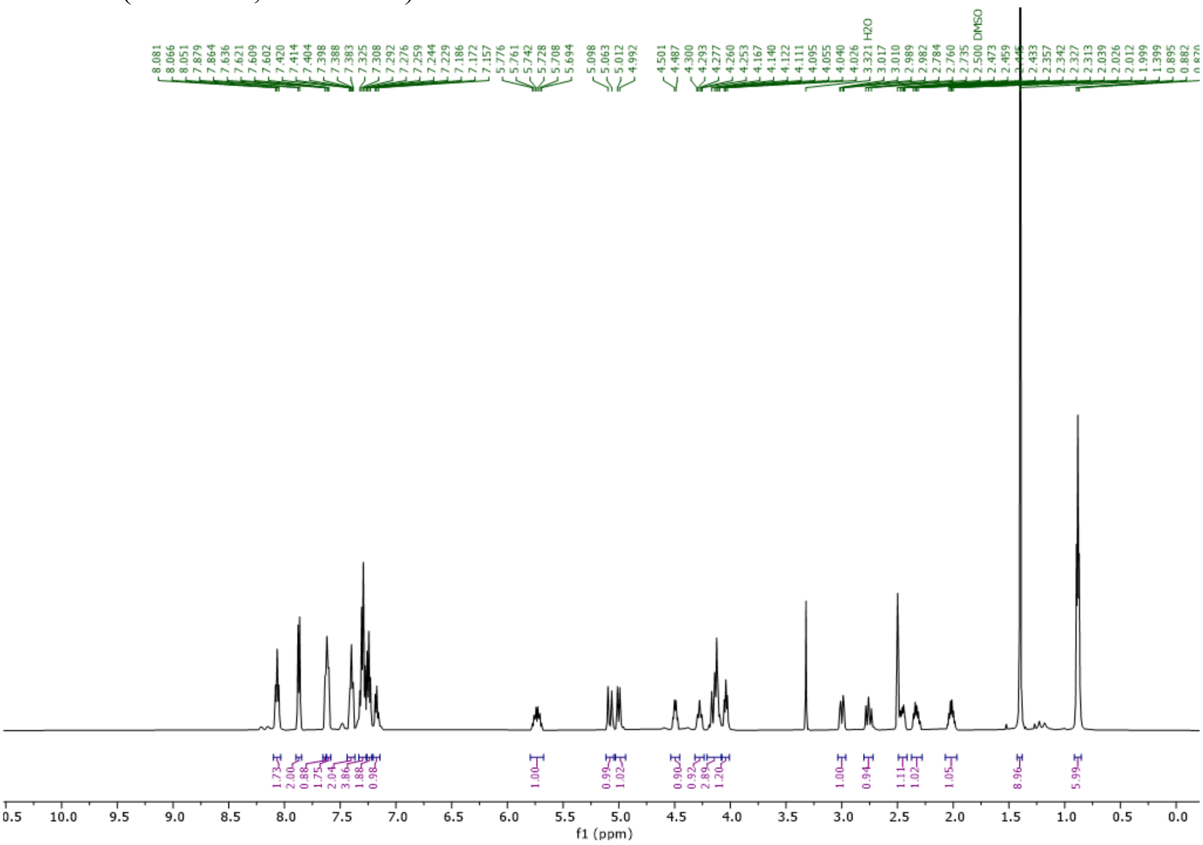

^{13}C-NMR (126 MHz, DMSO-d6)

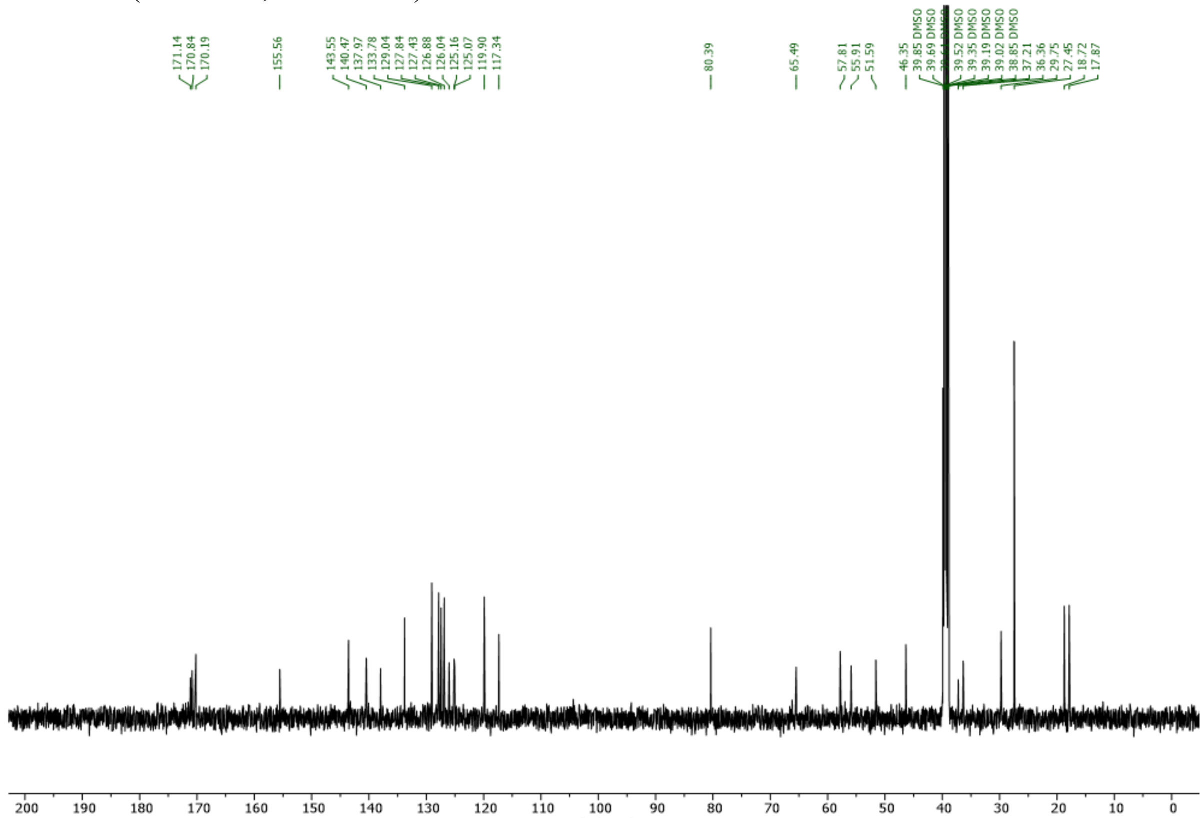

10.1.2 SI-4

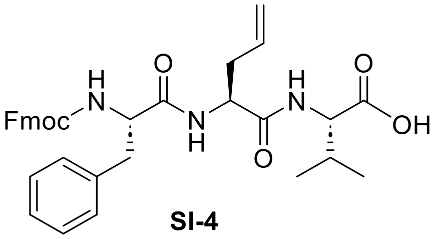

SI-4

Chemical Formula: $C_{34}H_{37}N_3O_6$

Exact Mass: 583.2682

LC-MS of crude: ca. 93% UV-purity

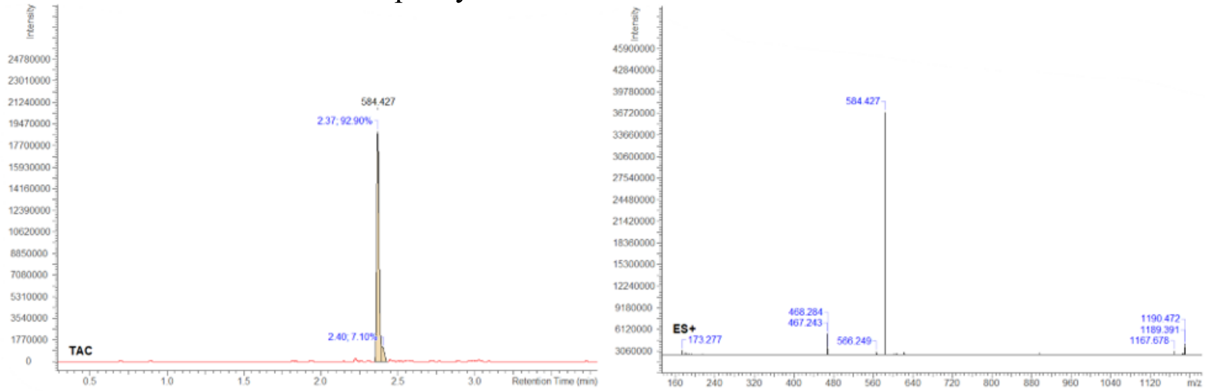

NMR:

^{1}H-NMR (500 MHz, DMSO-d6)

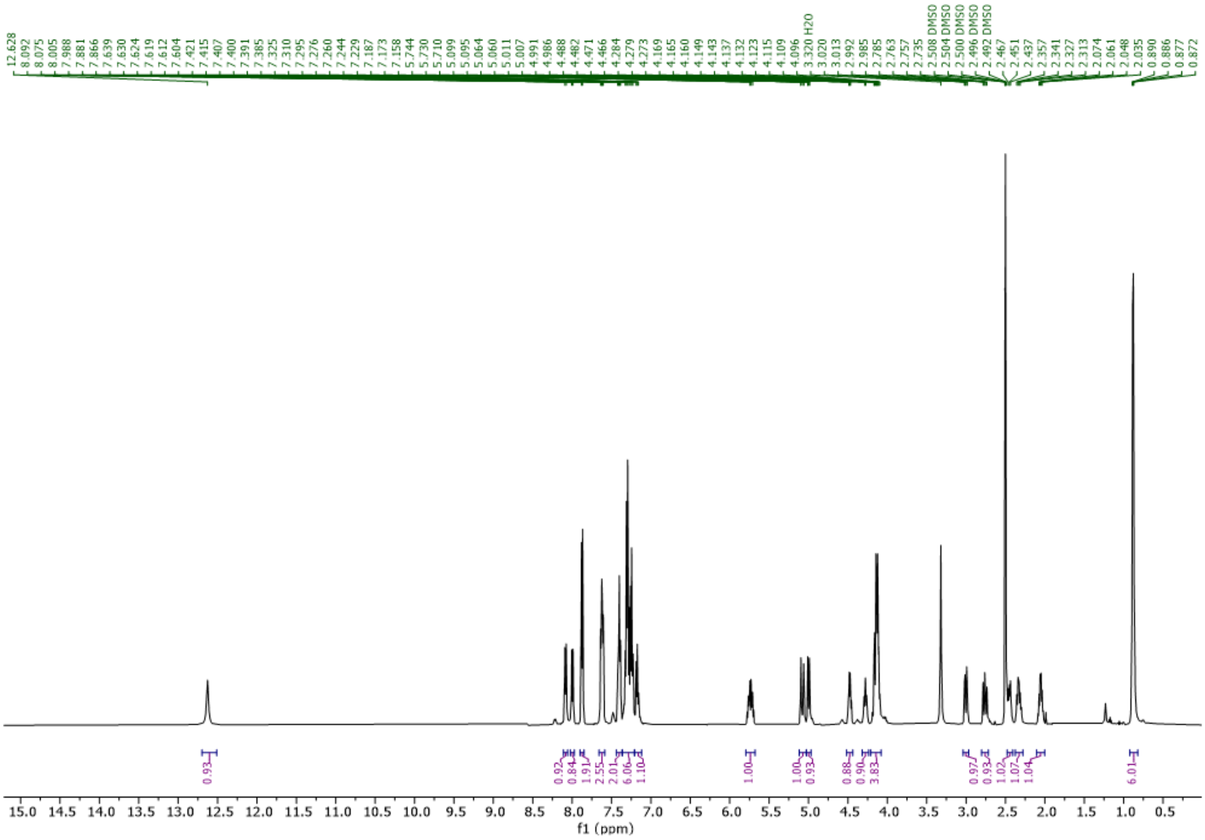

^{13}C-NMR (126 MHz, DMSO-d6)

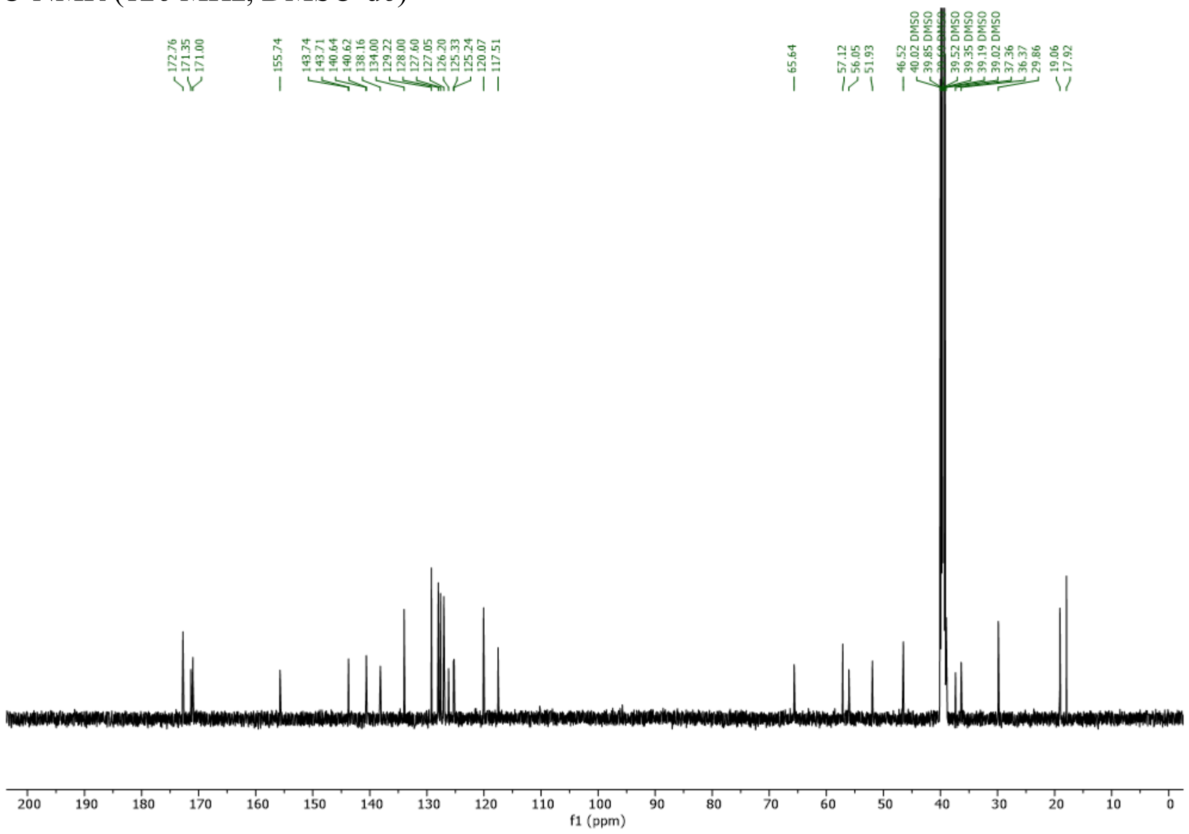

10.1.3 SI-5 and SI-6

SI-5, crude:

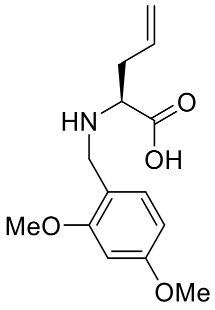

SI-5

Chemical Formula: $C_{14}H_{19}NO_4$

Exact Mass: 265.1314

NMR of crude:
^{1}H-NMR (500 MHz, DMSO-d6)

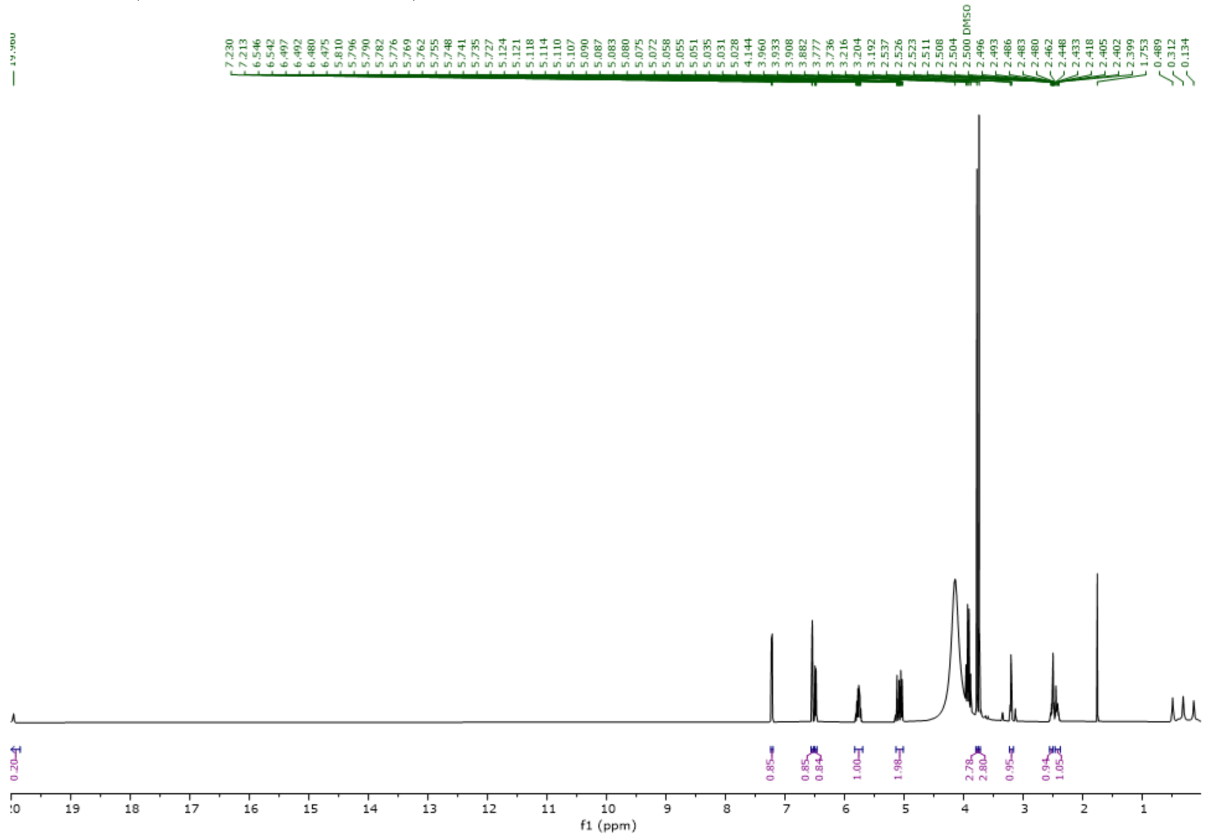

SI-6

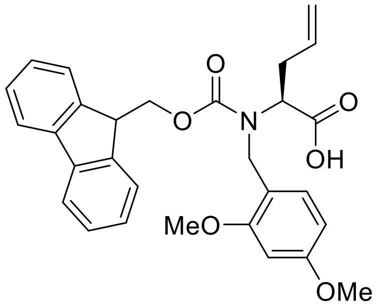

SI-6

Chemical Formula: $C_{29}H_{29}NO_6$

Exact Mass: 487.1995

LC-MS of crude: ca. 68% UV-purity

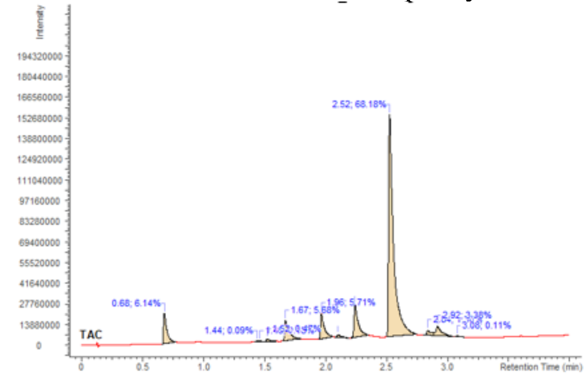

LC-MS of purified product: ca. >99% UV-purity

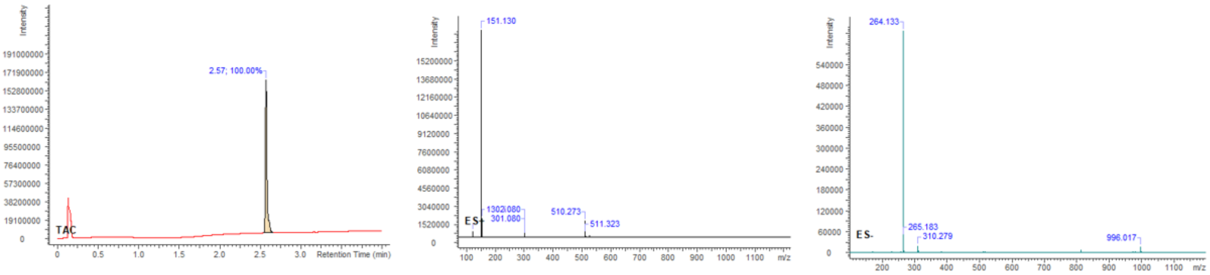

NMR of purified product: ^{1}H-NMR, ^{13}C-NMR, HSQC, HMBC, COSY, ROESY
→ROESY shows indices for presence of rotamers.
→qNMR: ca. 83% purity

^{1}H-NMR (600 MHz, DMSO-d6)

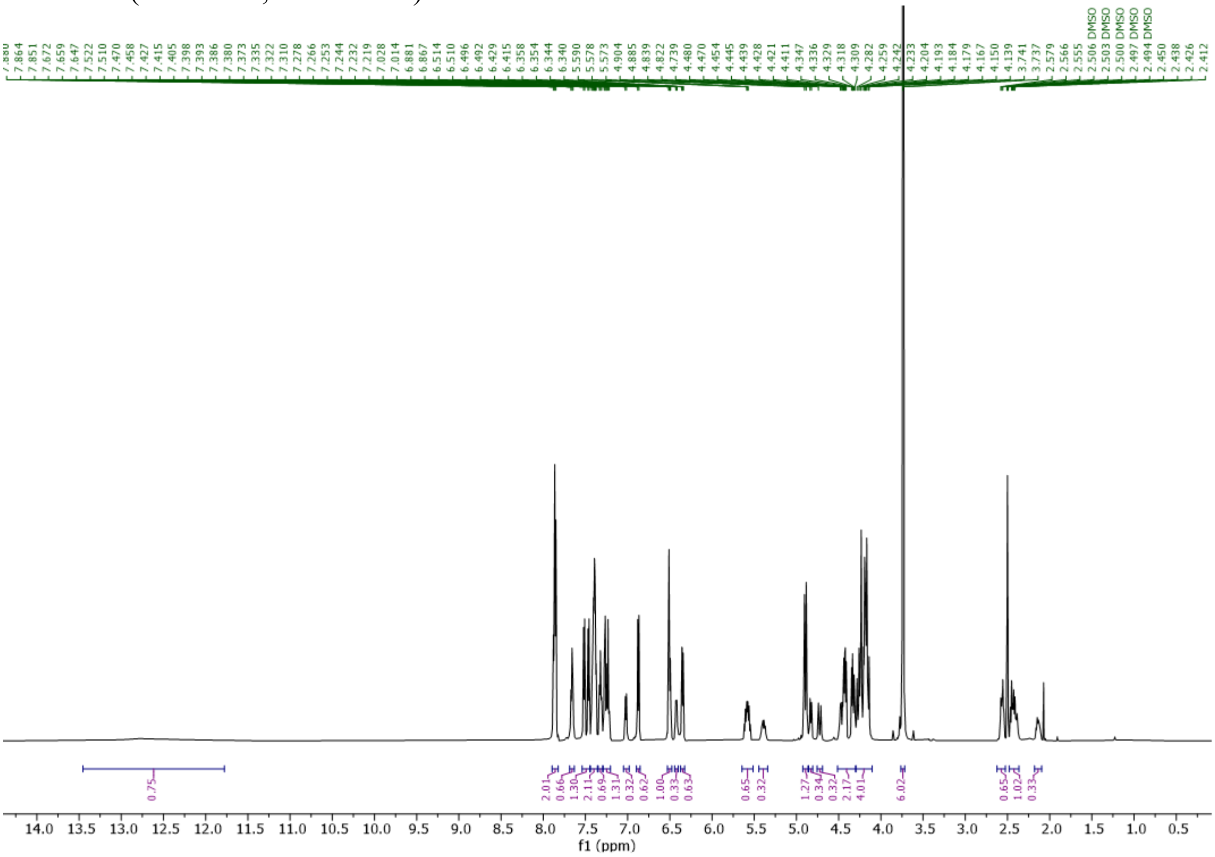

^{13}C-NMR (151 MHz, DMSO-d6)

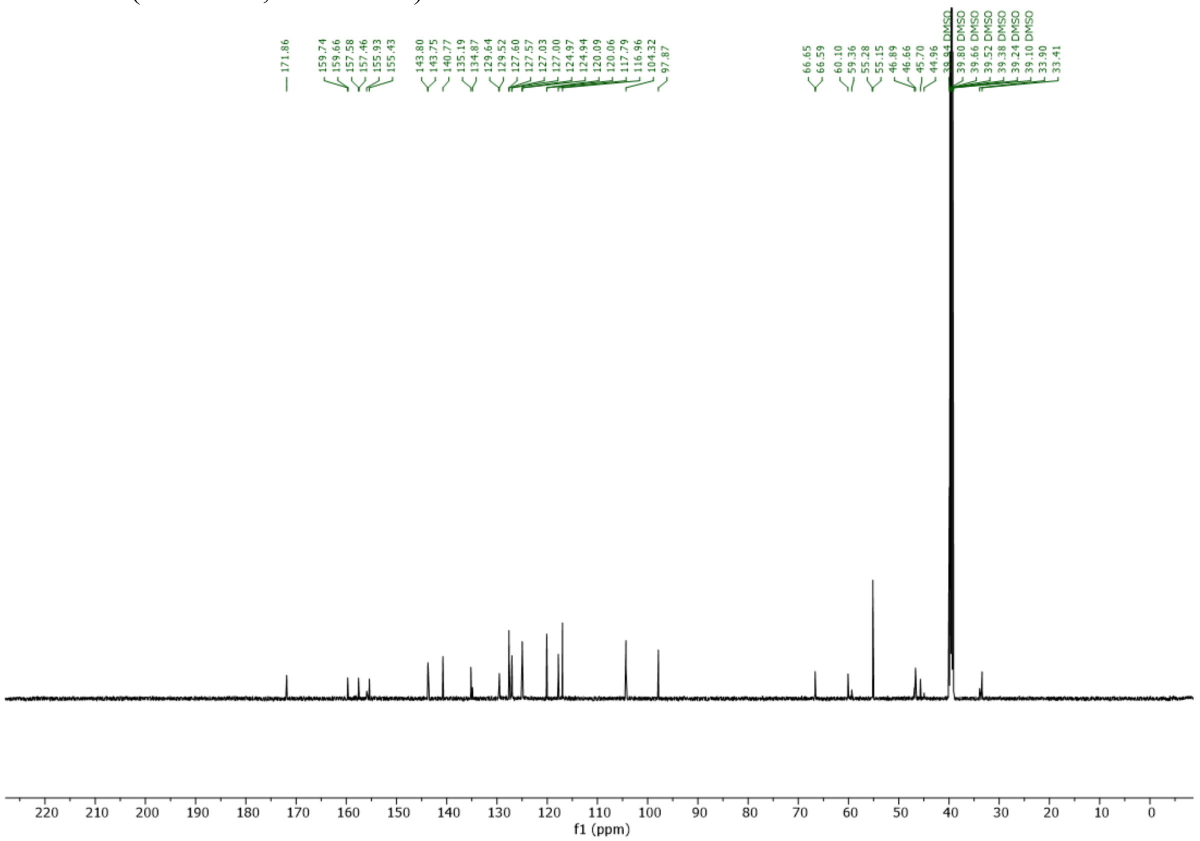

HSQC (600 MHz, DMSO-d6)

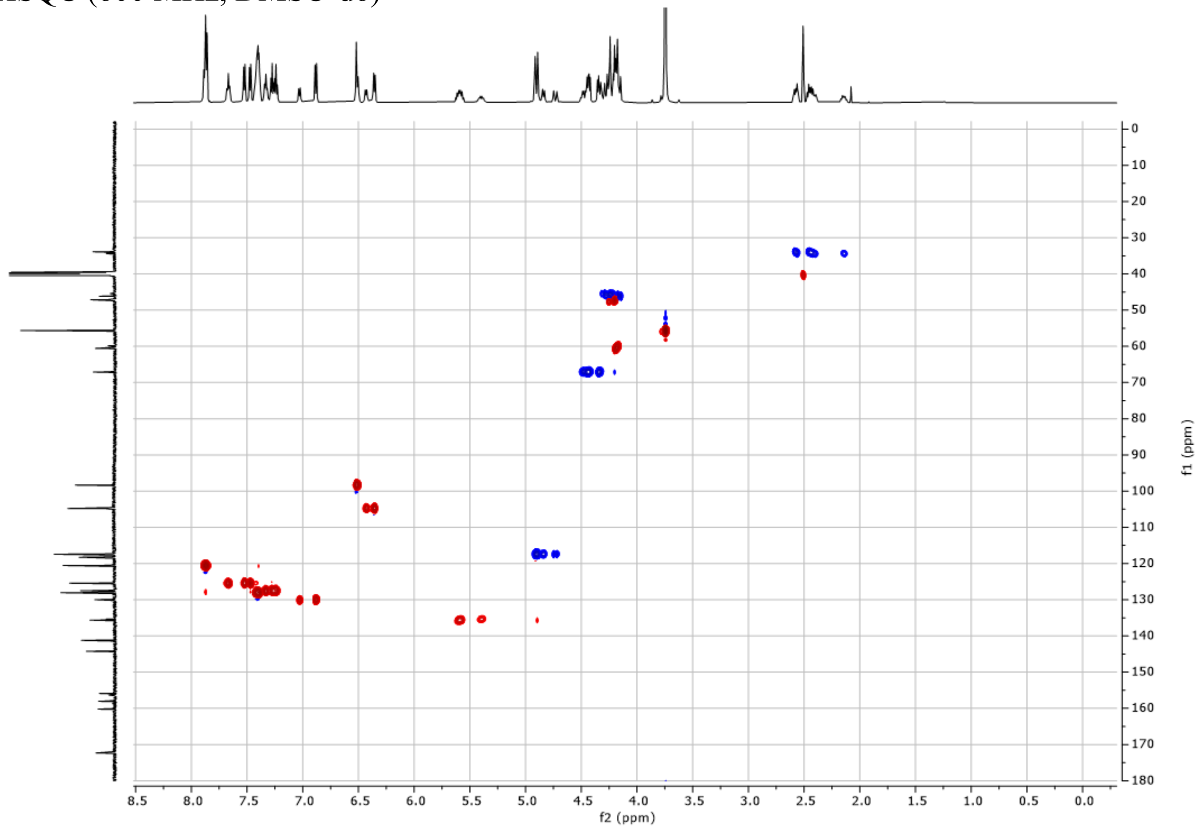

HMBC (600 MHz, DMSO-d6)

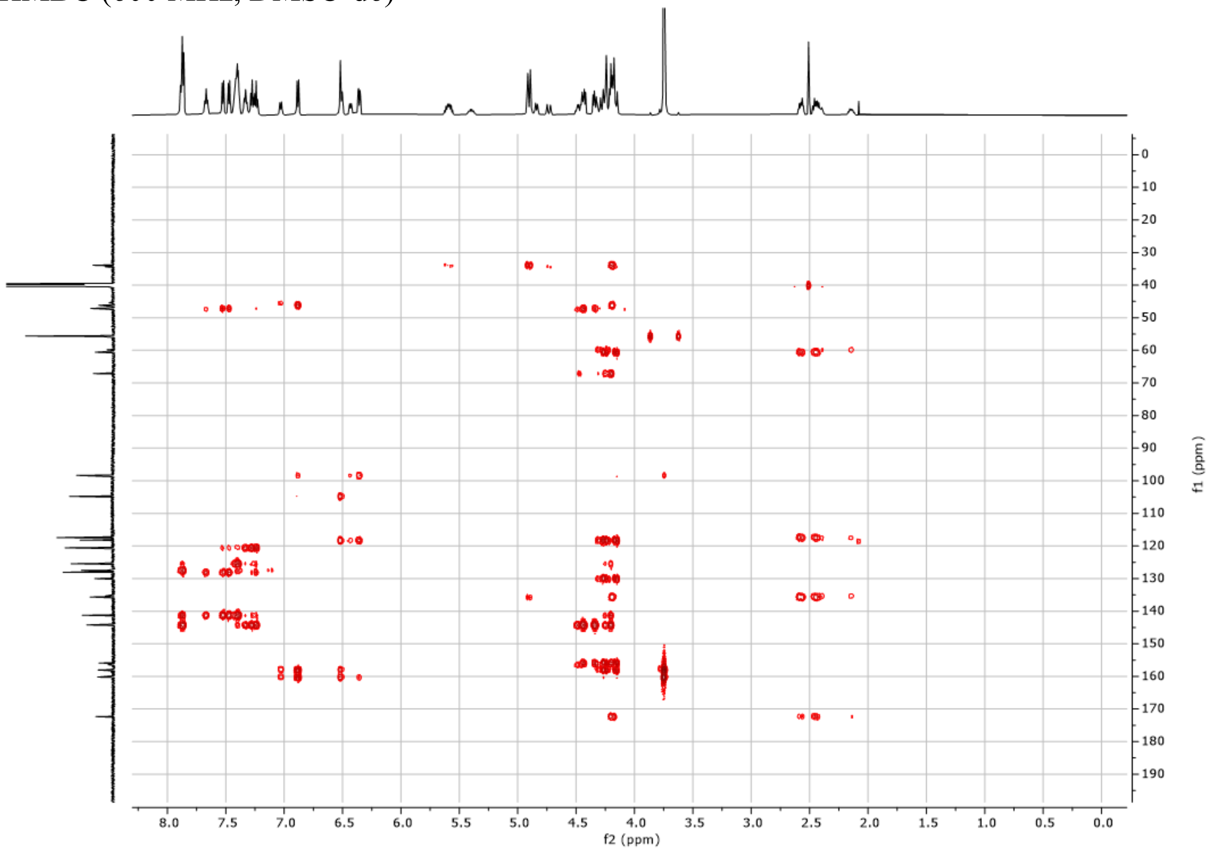

COSY (600 MHz, DMSO-d6)

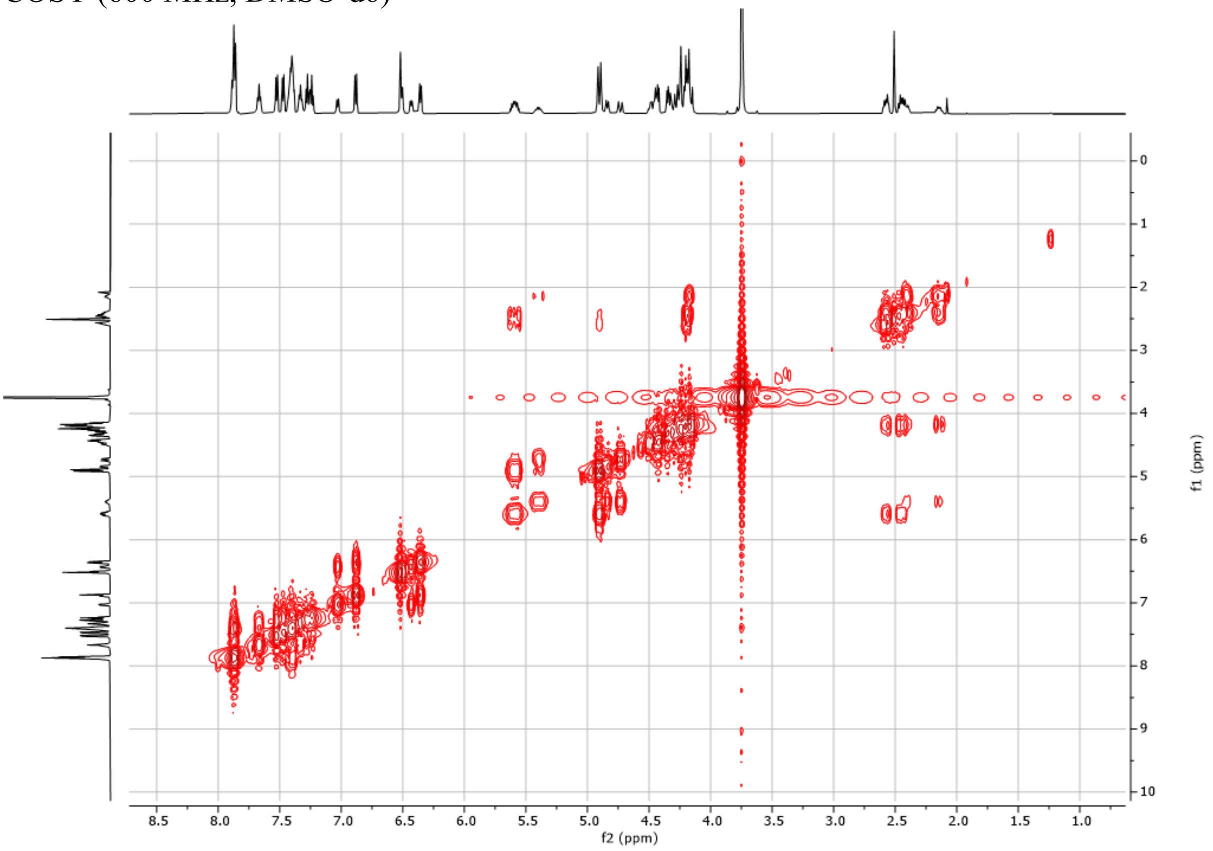

ROESY (600 MHz, DMSO-d6)

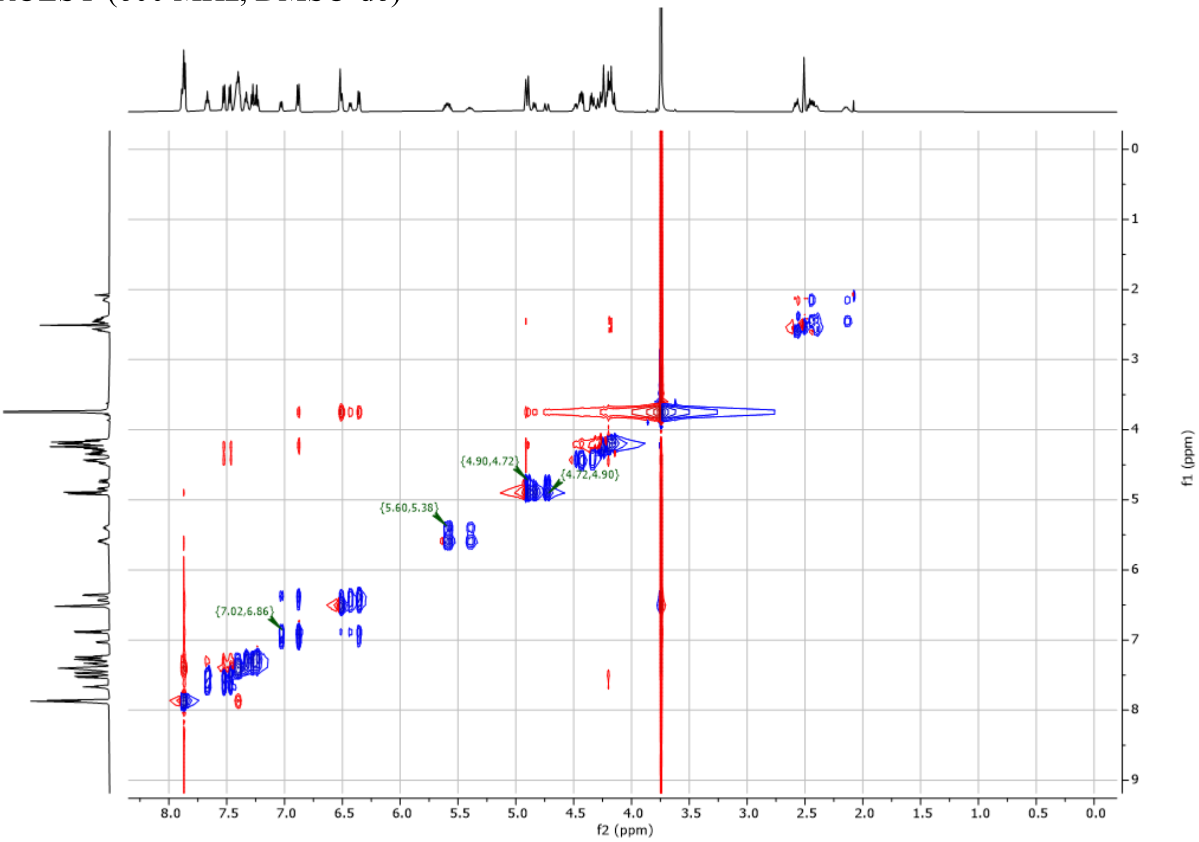

10.1.4 1SMa and 1SMb

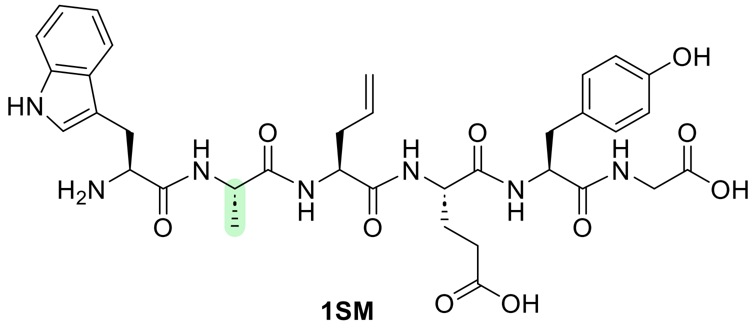

1SM

Chemical Formula: $C_{35}H_{43}N_7O_{10}$

Exact Mass: 721.3071

LC-MS of crude **1SMa**: ca. 98% UV-purity

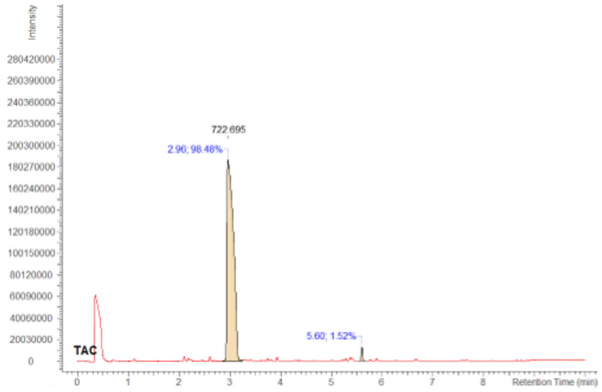

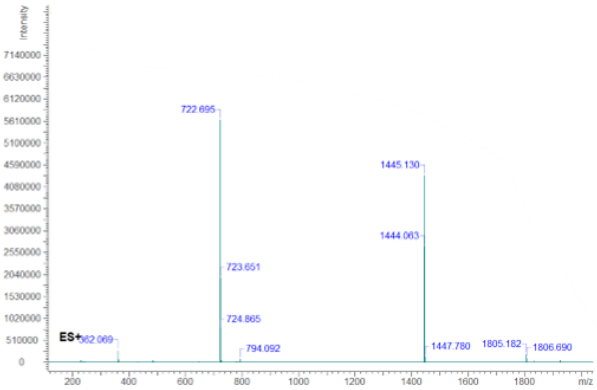

^{1}H-NMR (500 MHz, DMSO-d6): has Et_2O contamination

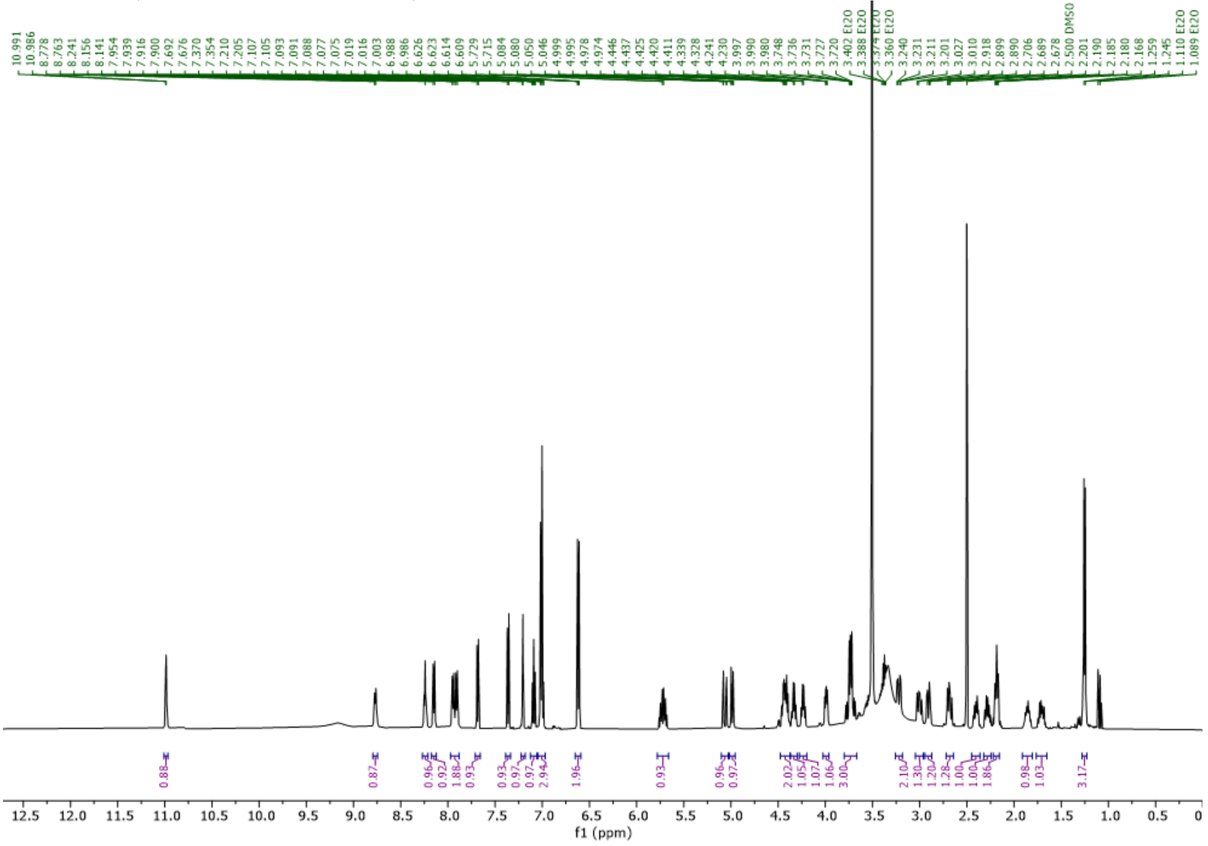

10.1.5 2SM

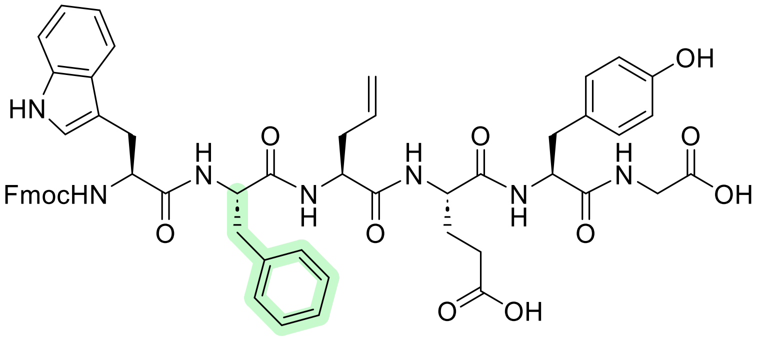

2SM

Chemical Formula: $C_{56}H_{57}N_7O_{12}$

Exact Mass: 1019.4065

LC-MS: ca. 99% UV-purity

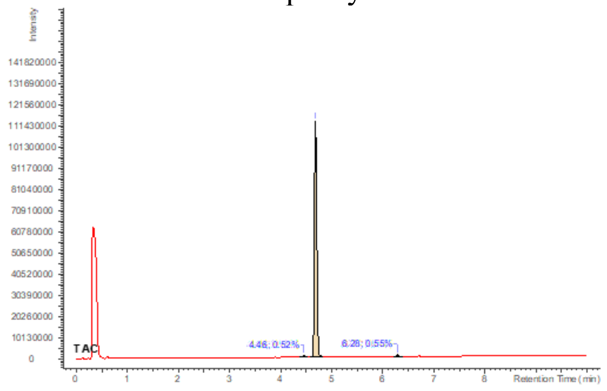

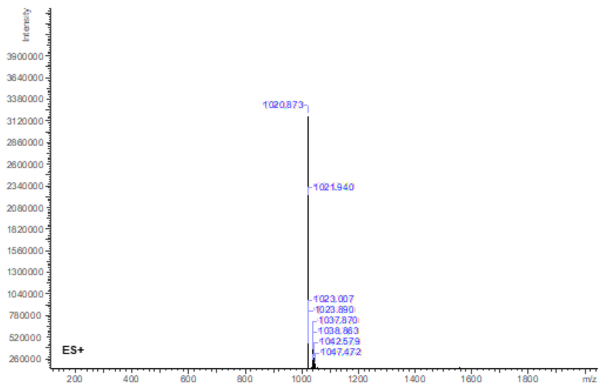

^{1}H-NMR (500 MHz, DMSO-d6): contains internal standard 1,3,5-TMB, and some Et_2O contamination

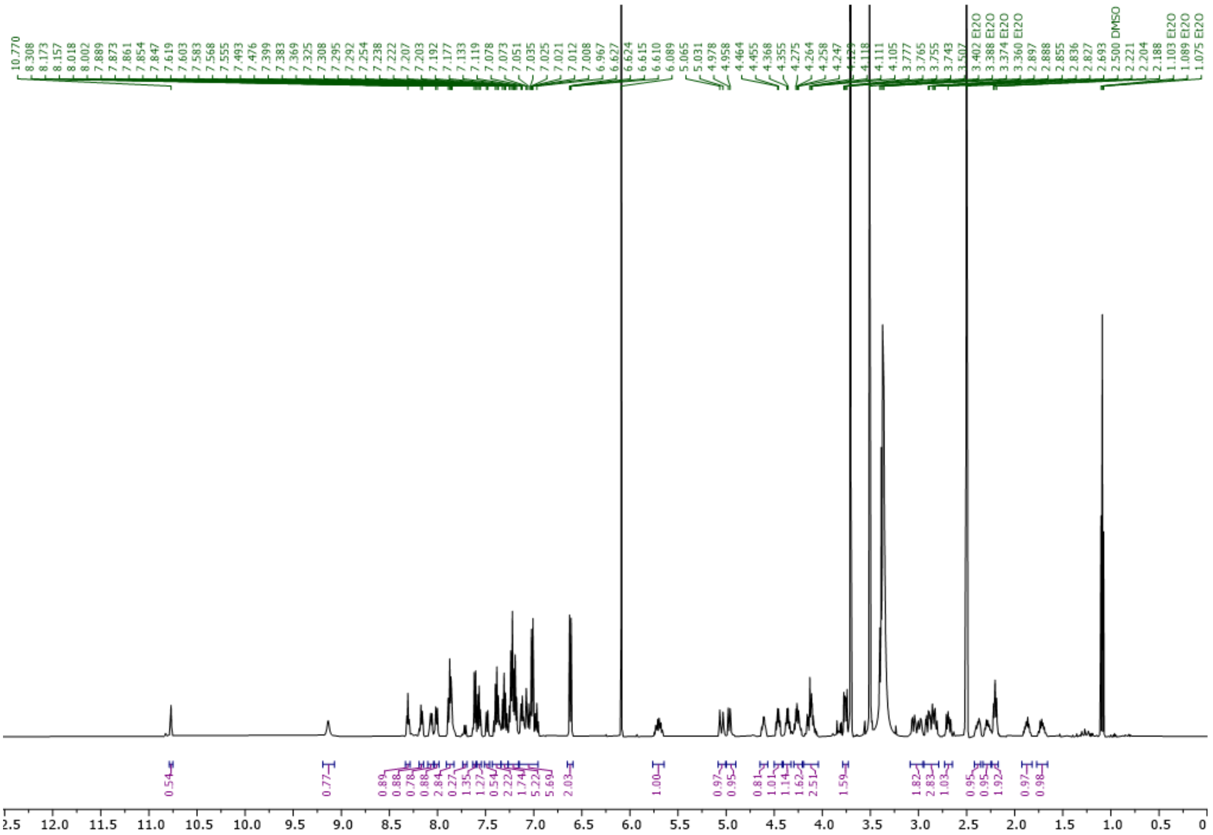

10.1.6 3SM

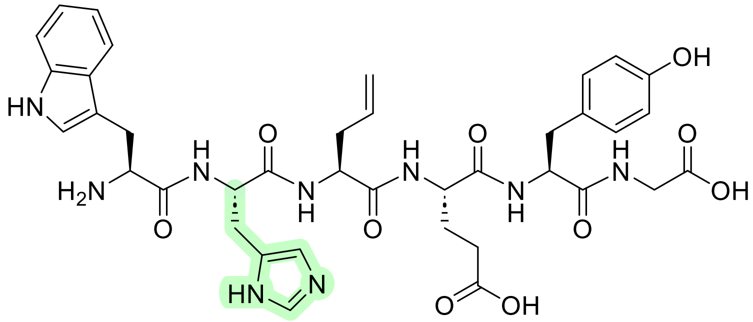

Precursor for Hex-9
Chemical Formula: $C_{38}H_{45}N_9O_{10}$
Exact Mass: 787.3289

LC-MS of crude: ca. 81% UV-purity

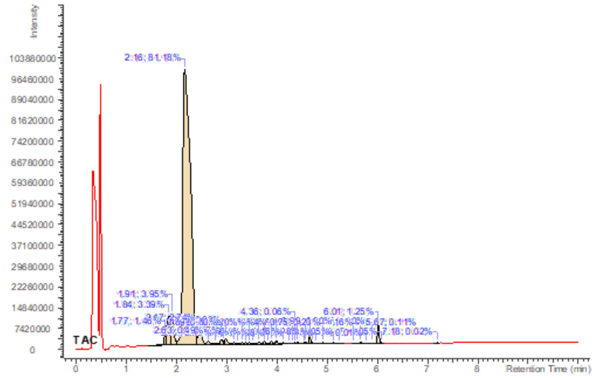

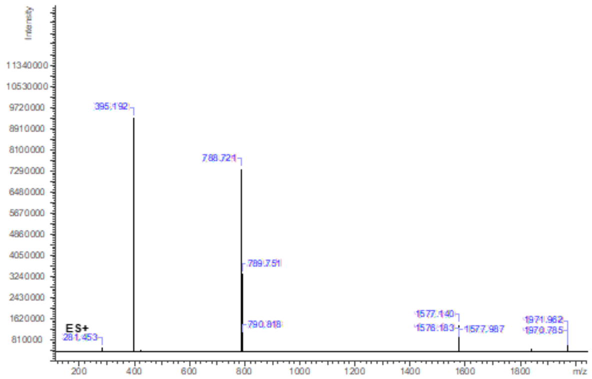

^{1}H-NMR (600 MHz, DMSO-d6): contains internal standard 1,3,5-TMB, and some Et_2O contamination

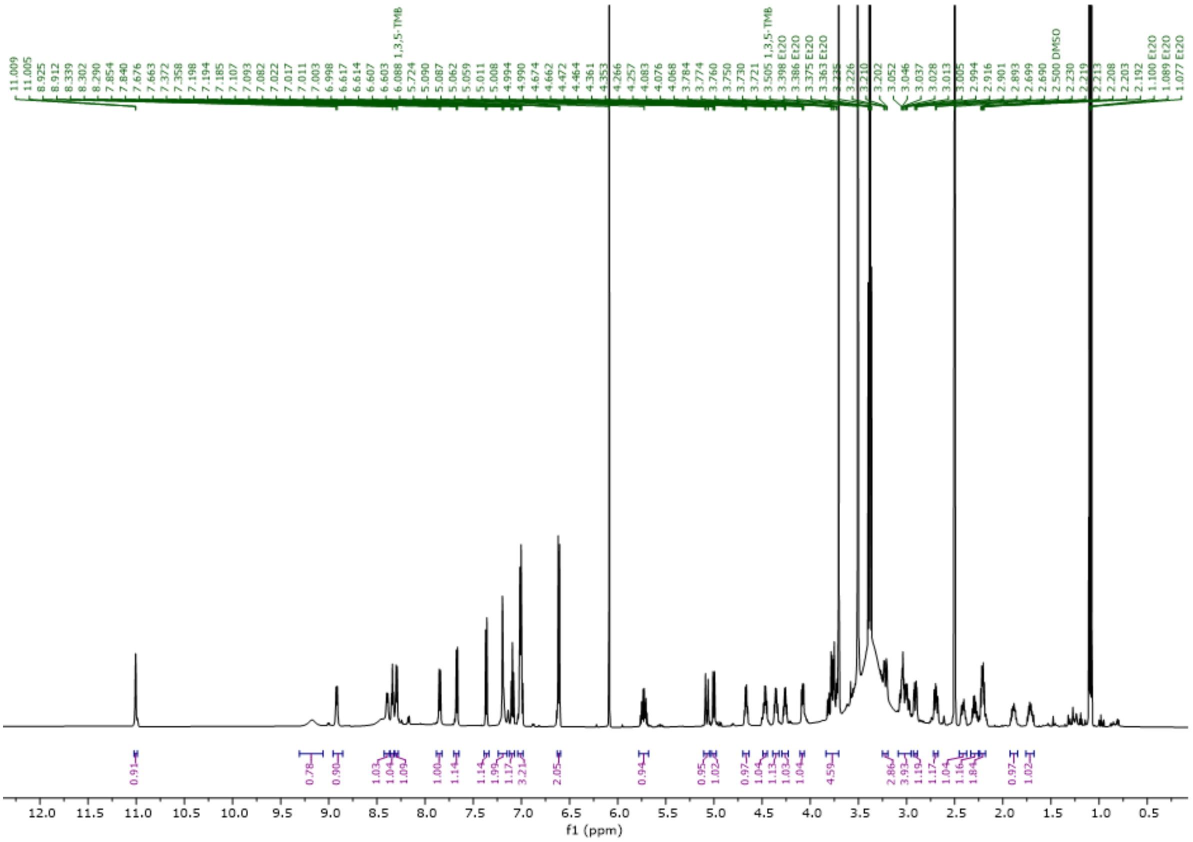

10.1.7 4SM

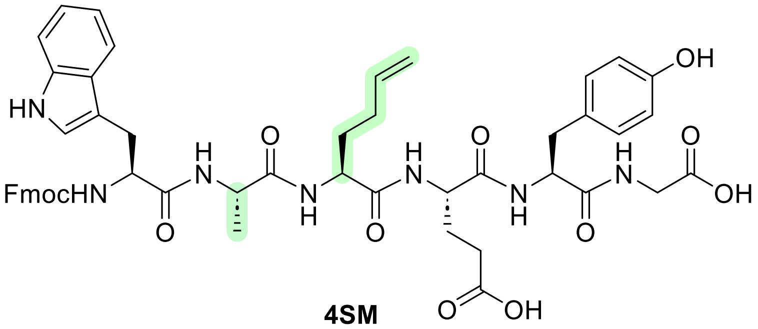

Chemical Formula: $C_{51}H_{55}N_7O_{12}$
Exact Mass: 957.3909

LC-MS of crude: ca. 95% UV-purity

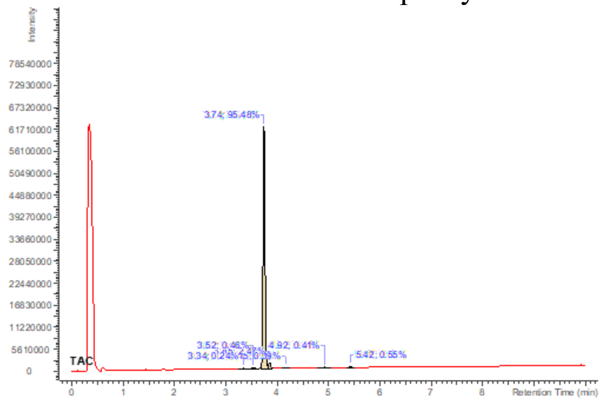

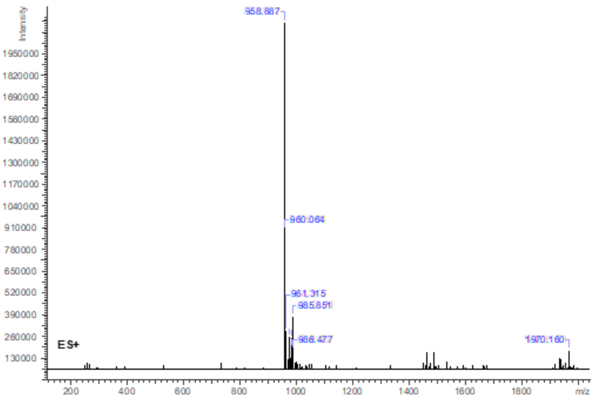

^{1}H-NMR (500 MHz, DMSO-d6): contains internal standard 1,3,5-TMB, and some Et_2O contamination

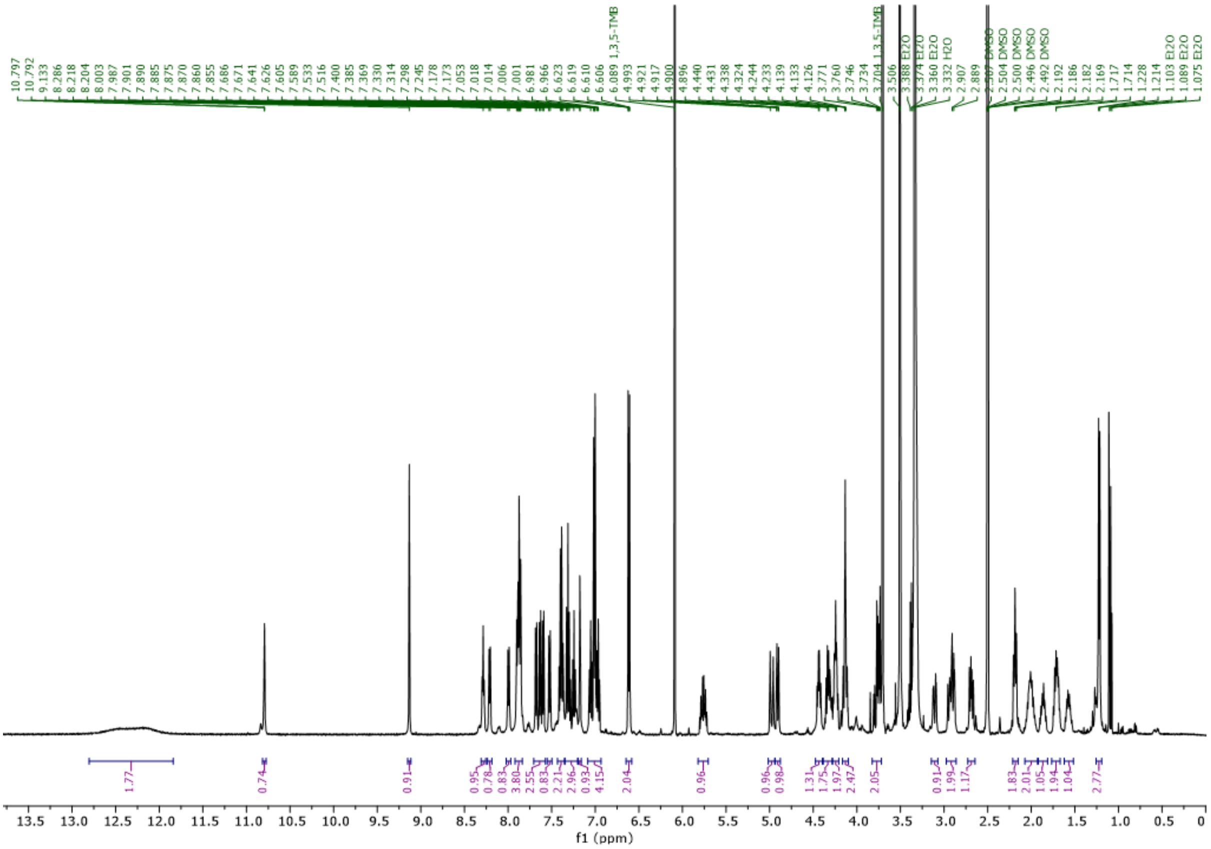

10.1.8 **5SM** (Precursor for **1C** during optimization)

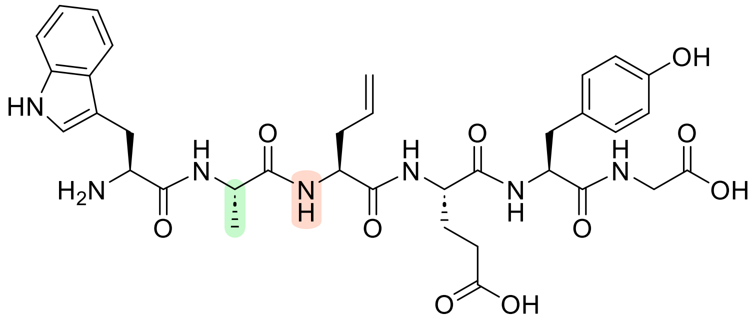

5SM

Chemical Formula: $C_{35}H_{43}N_7O_{10}$

Exact Mass: 721.3071

LC-MS of crude: very impure, ca. 46% UV-purity

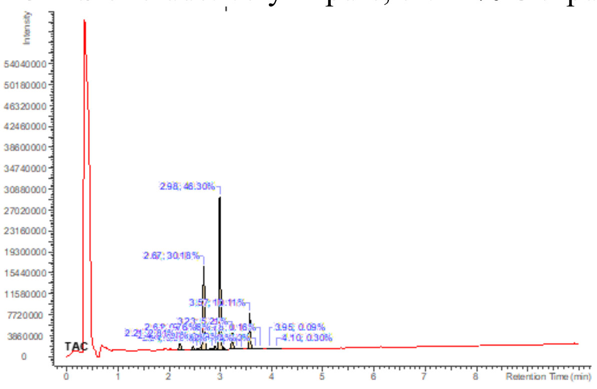

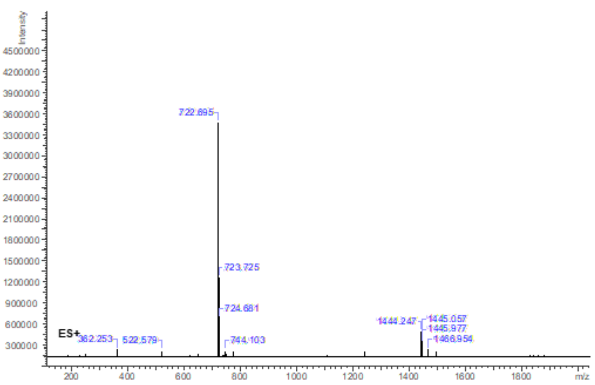

^{1}H-NMR (600 MHz, DMSO-d6): very impure, contains internal standard 1,3,5-TMB, and some Et_2O contamination

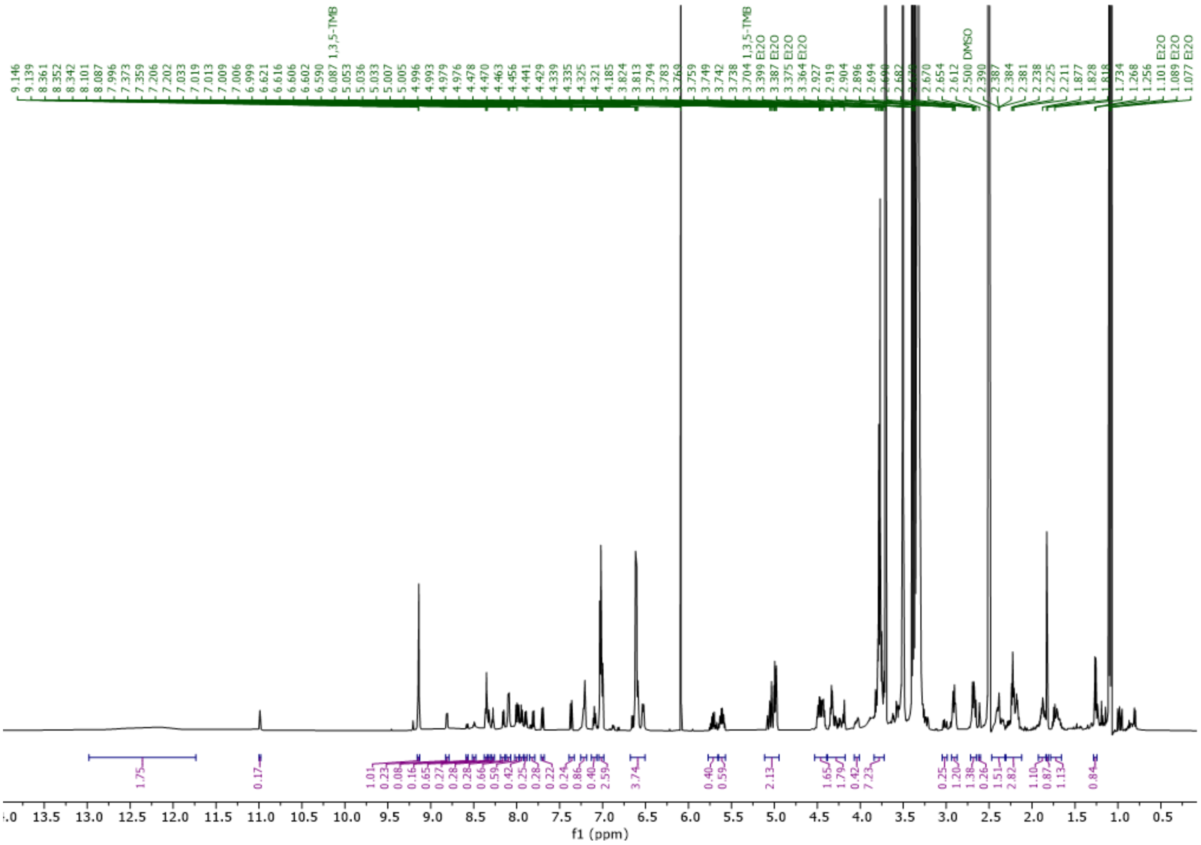

10.1.9 6SM

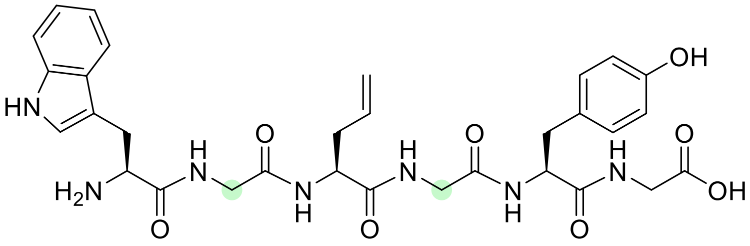

6SM

Chemical Formula: $C_{31}H_{37}N_7O_8$

Exact Mass: 635.2704

LC-MS of crude: ca. 90% UV-purity (Peak 10: Internal Standard (IS, 1,3,5-trimethoxybenzene, TMB)).

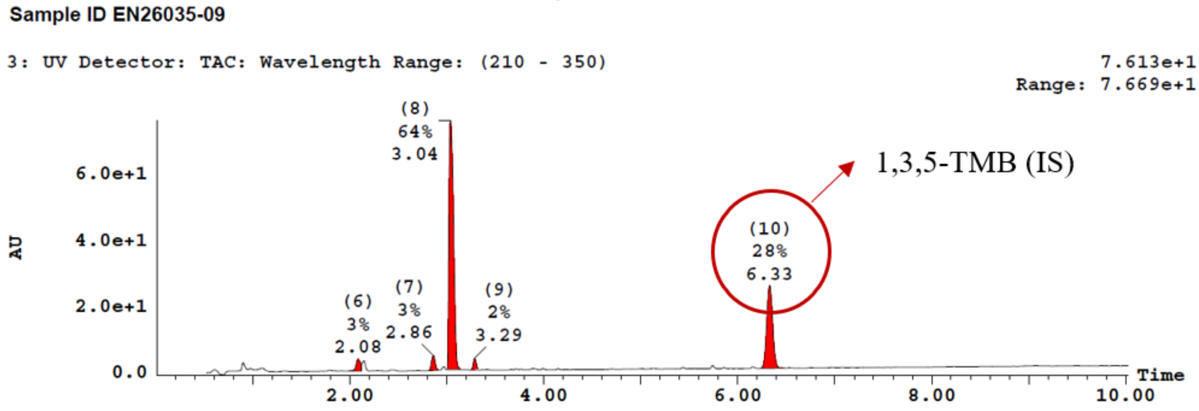

Peak ID	Time	Mass Found
8	3.05	Not Found

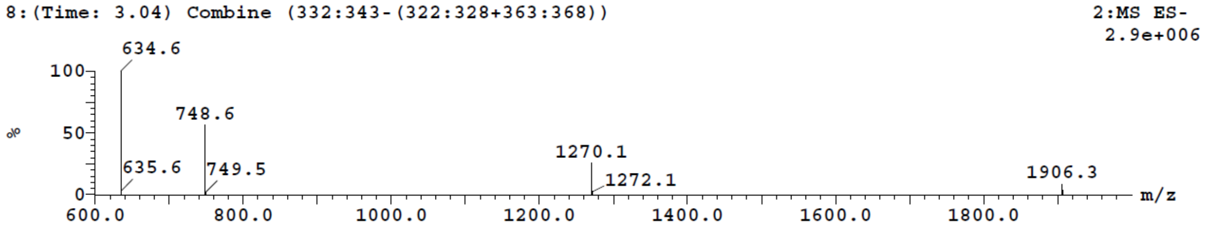

^{1}H-NMR (500 MHz, DMSO-d6): contains 1,3,5-TMB

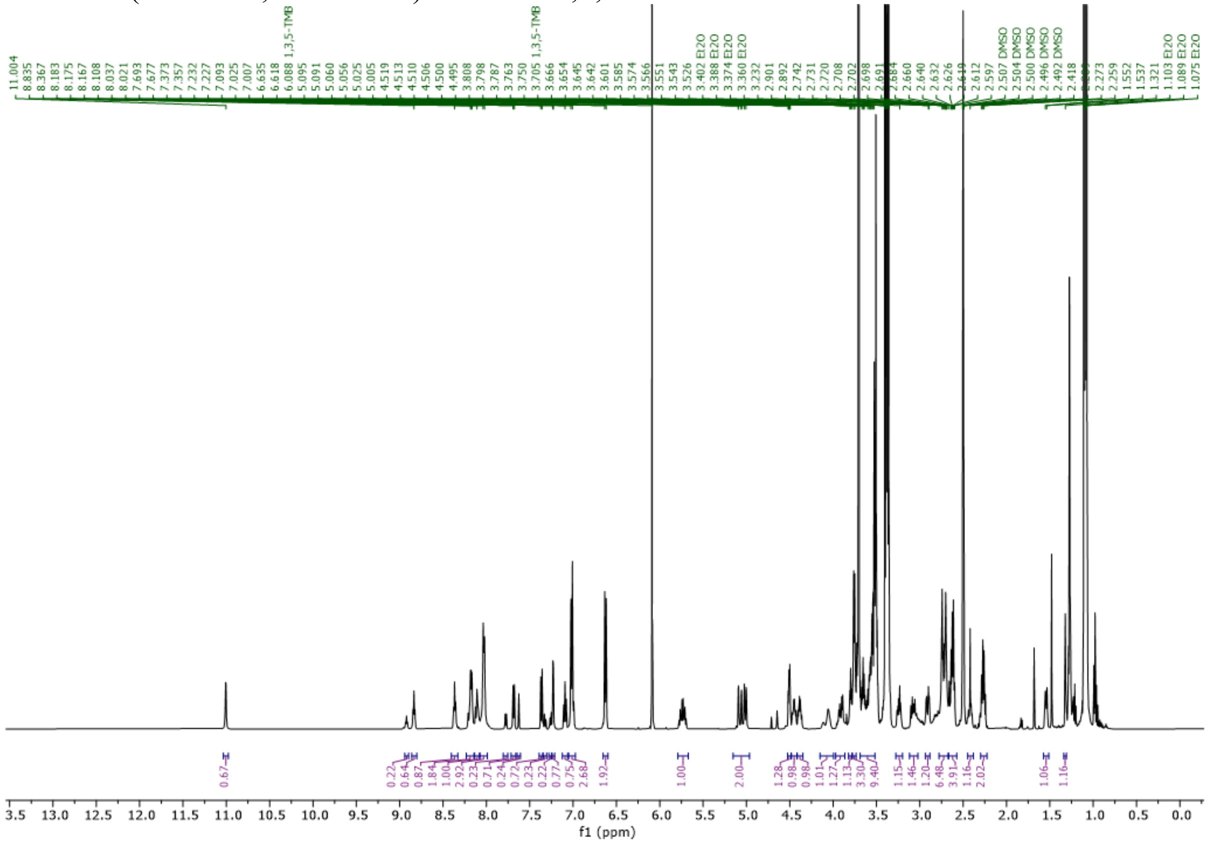

10.1.10 7SM

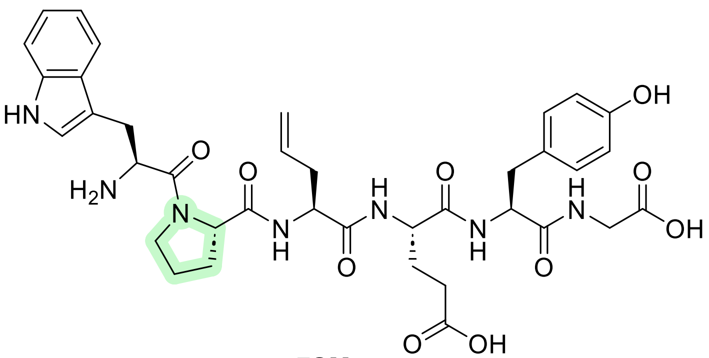

7SM

Chemical Formula: $C_{37}H_{45}N_7O_{10}$

Exact Mass: 747.3228

LC-MS of crude: ca. 95% UV-purity

Sample ID EN26035-05

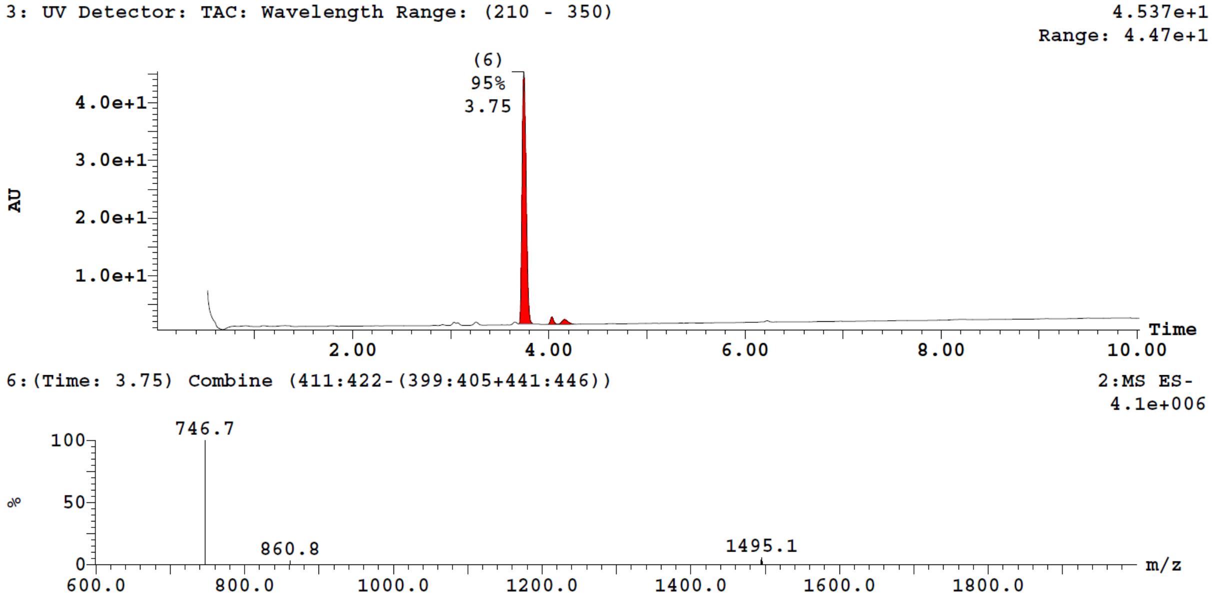

^{1}H-NMR (500 MHz, DMSO-d6): contains 1,3,5-TMB

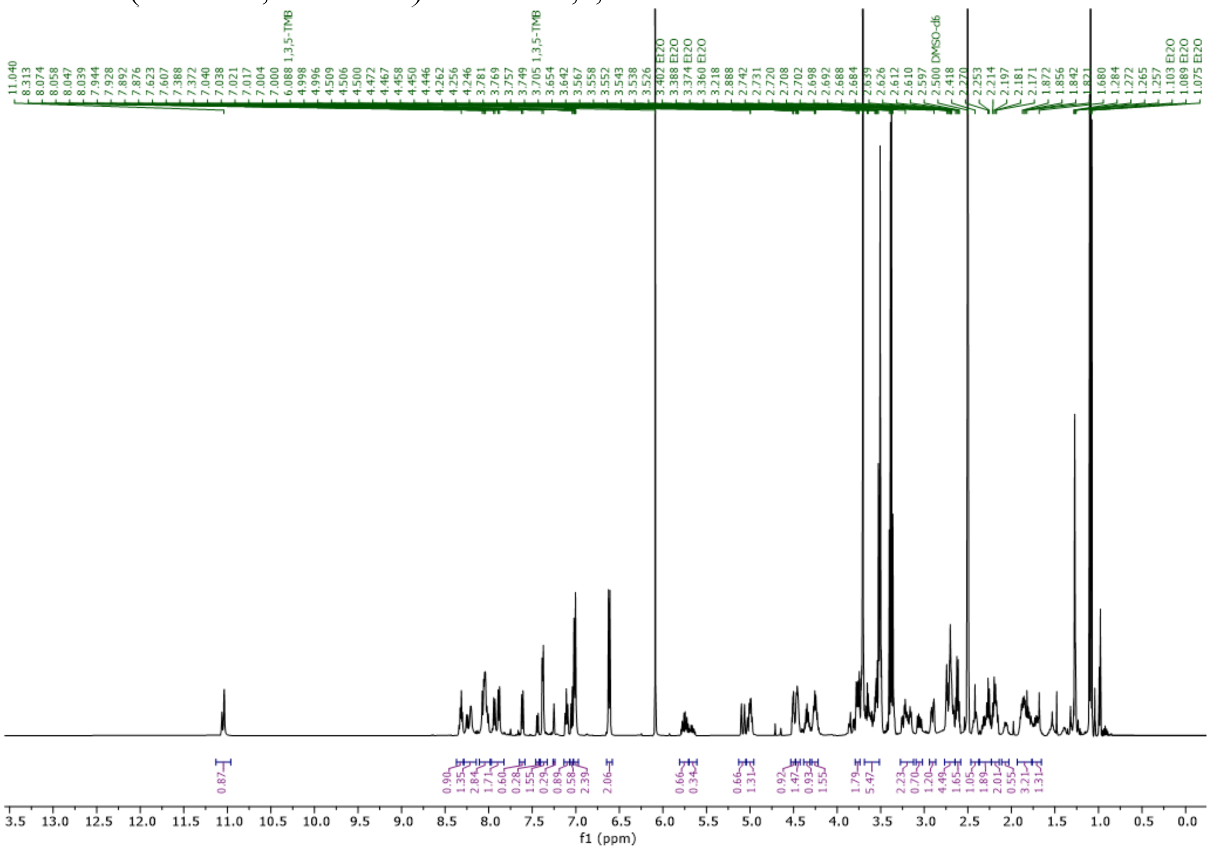

10.1.11 8SM

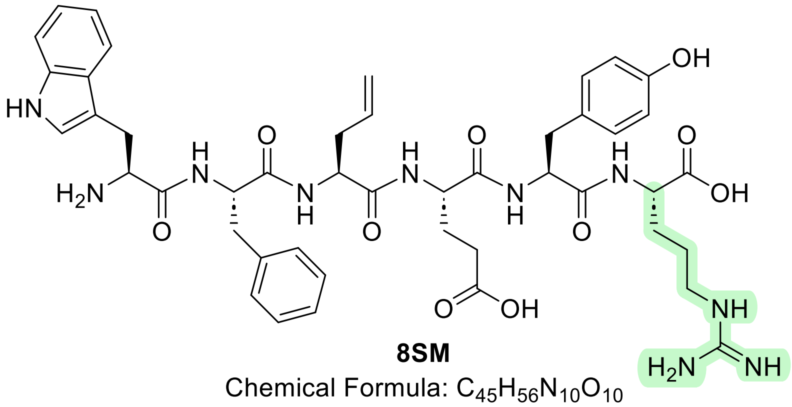

8SM

Chemical Formula: $C_{45}H_{56}N_{10}O_{10}$

Exact Mass: 896.4181

LC-MS of crude: ca. 97% UV-purity (shows m/z of [M+TFA]$^-$), contains 1,3,5-trimethoxybenzene (internal standard) at 6.32 min

Sample ID EN26035-10

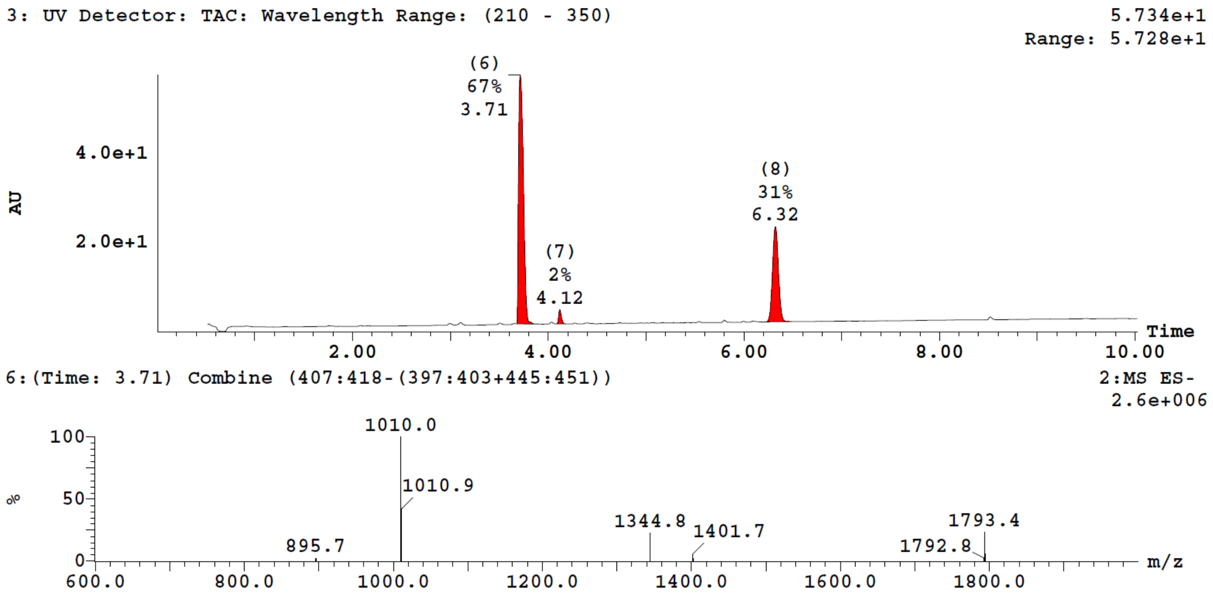

^{1}H-NMR (500 MHz, DMSO-d6): contains 1,3,5-TMB

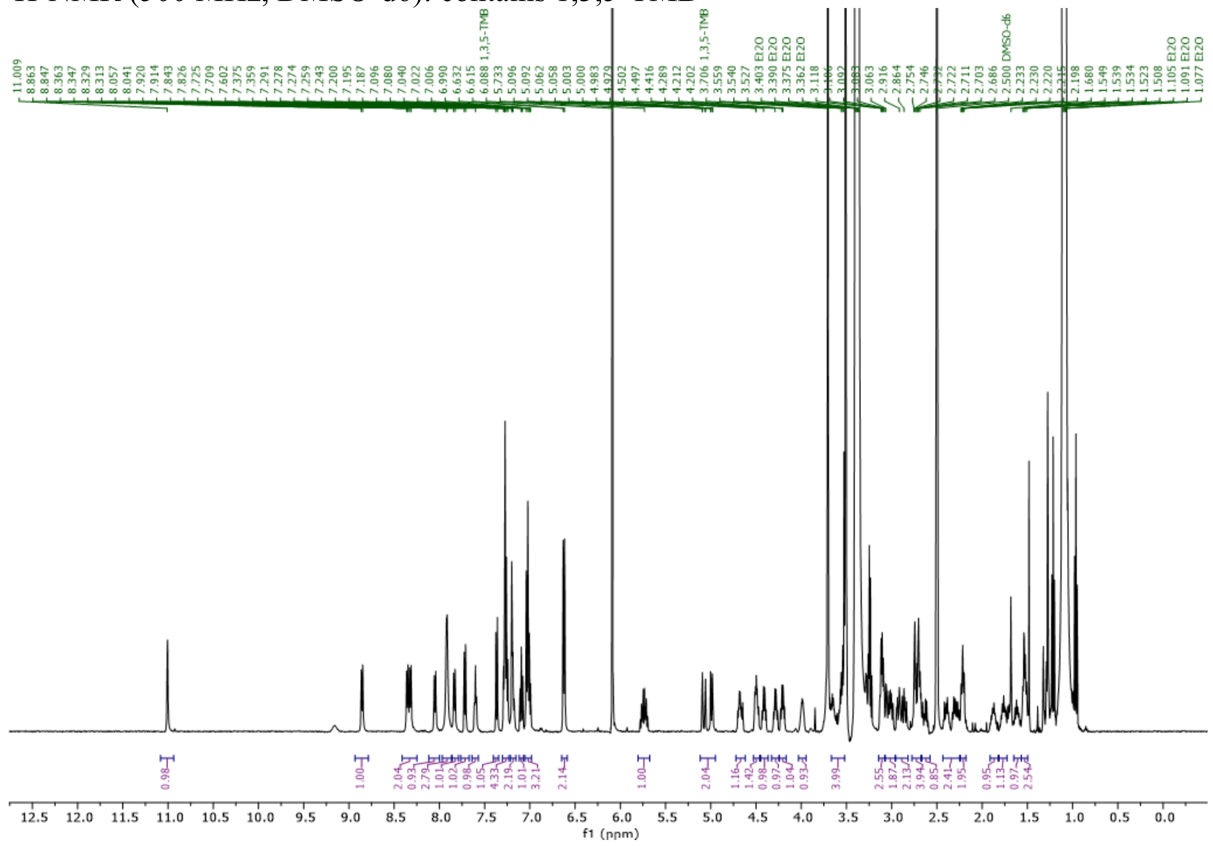

10.1.12 9SM

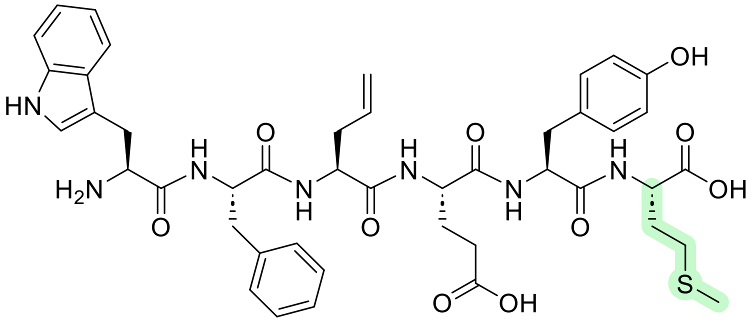

9SM

Chemical Formula: $C_{44}H_{53}N_7O_{10}S$

Exact Mass: 871.3575

LC-MS of crude: ca. 67% UV-purity

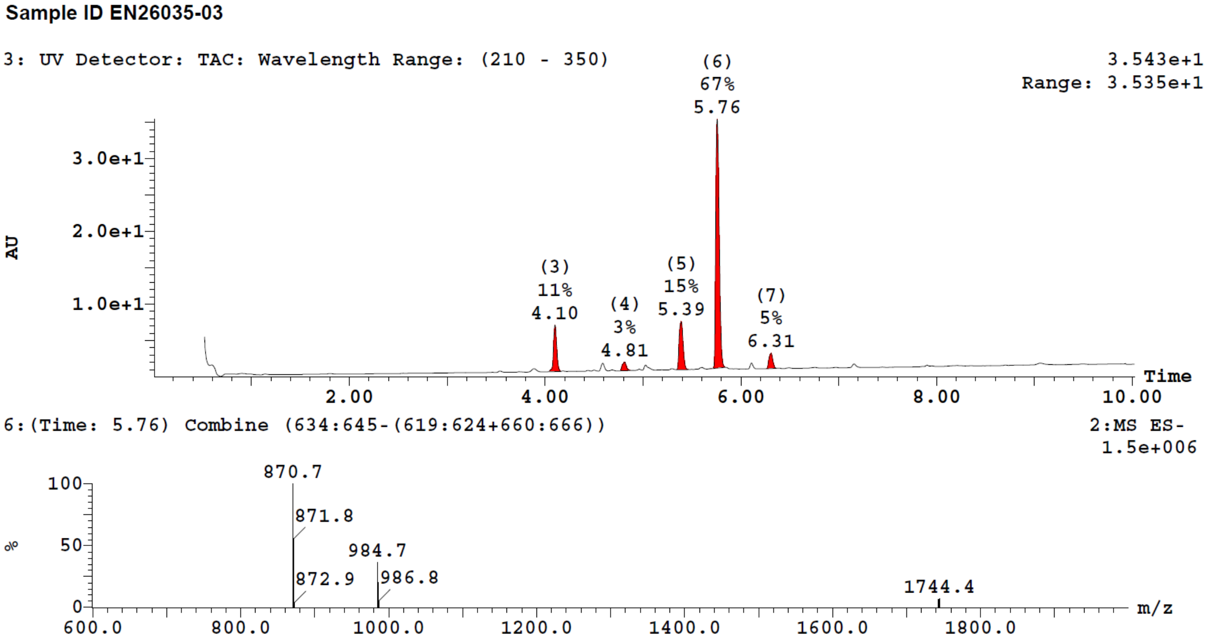

^{1}H-NMR (500 MHz, DMSO-d6): contains 1,3,5-TMB

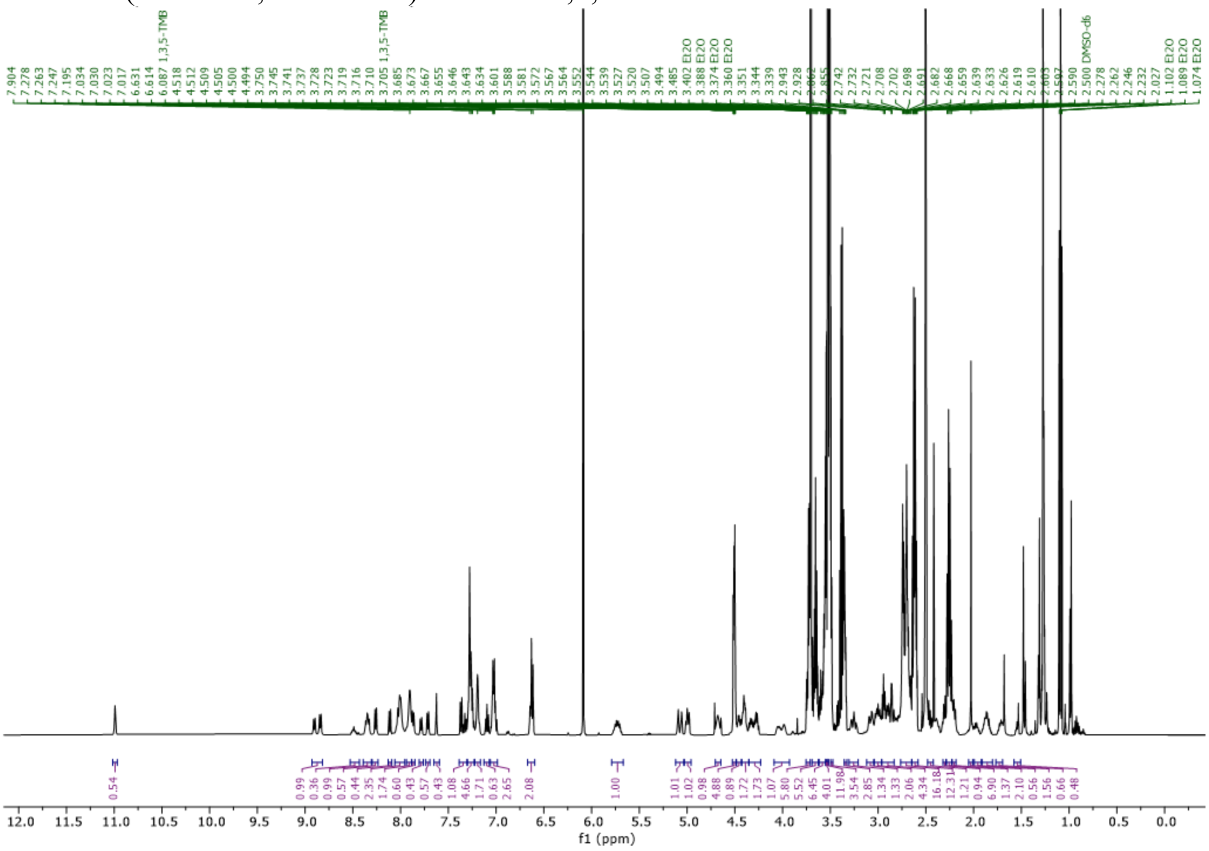

10.1.13 10SM

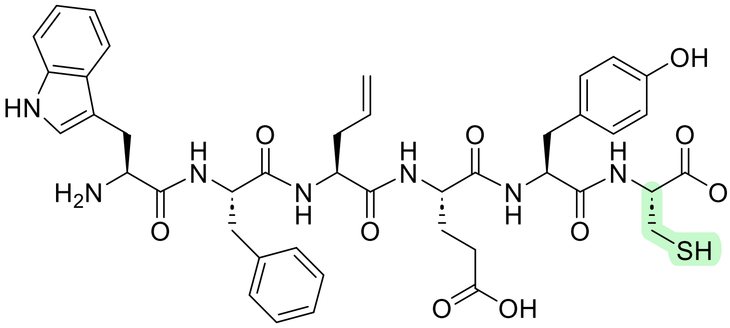

10SM
Chemical Formula: $C_{42}H_{49}N_7O_{10}S$
Exact Mass: 843.3262

LC-MS of crude: ca. 75% UV-purity (the signal at 5.39 min also contains the mass of the product in low-resolution MS, and could be a not-further characterized isomer)

Sample ID EN26035-04

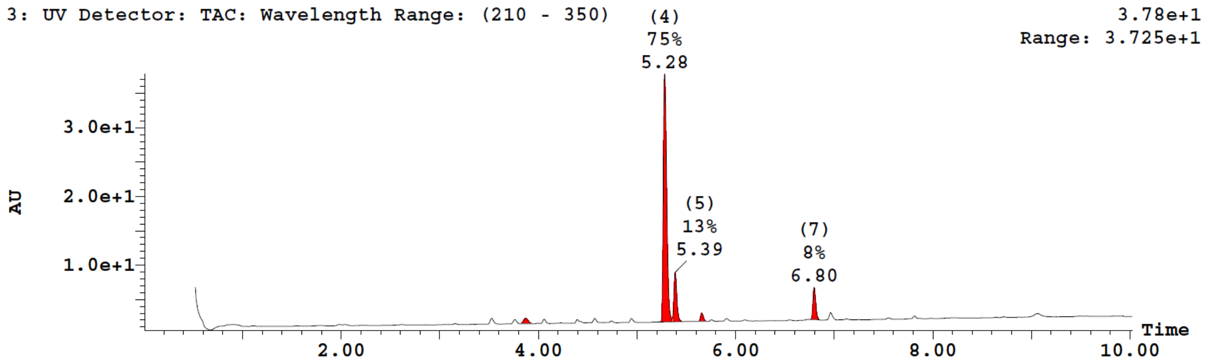

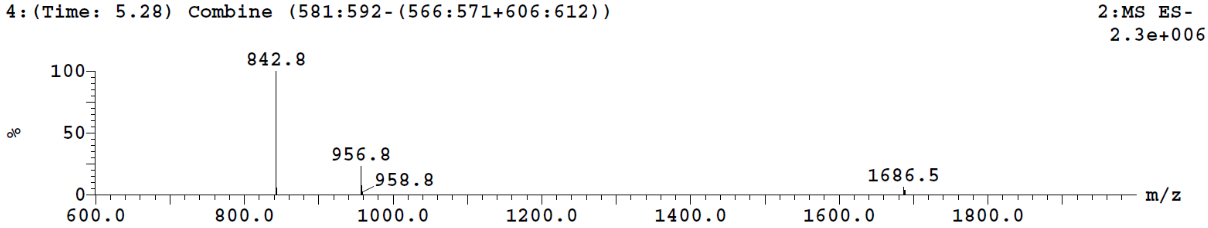

^{1}H-NMR (500 MHz, DMSO-d6): contains 1,3,5-TMB

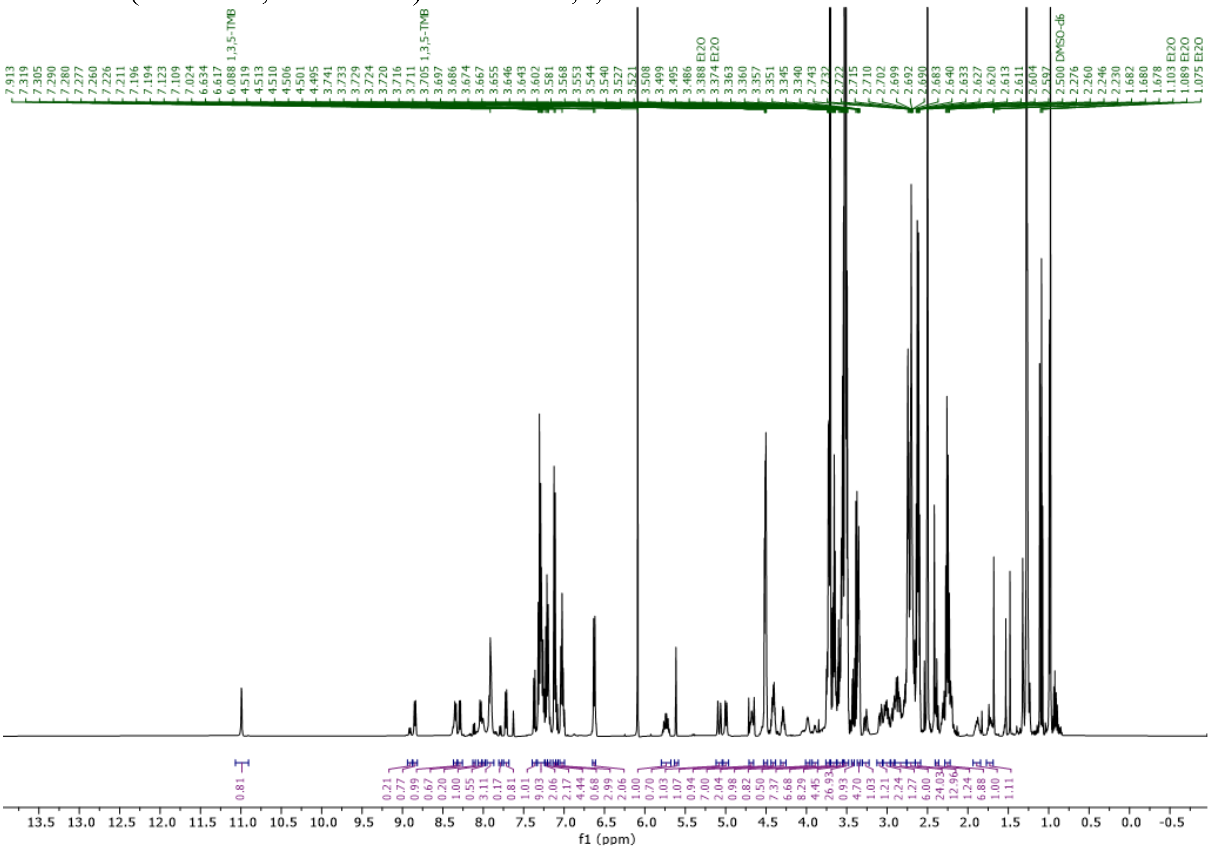

10.1.14 11SM

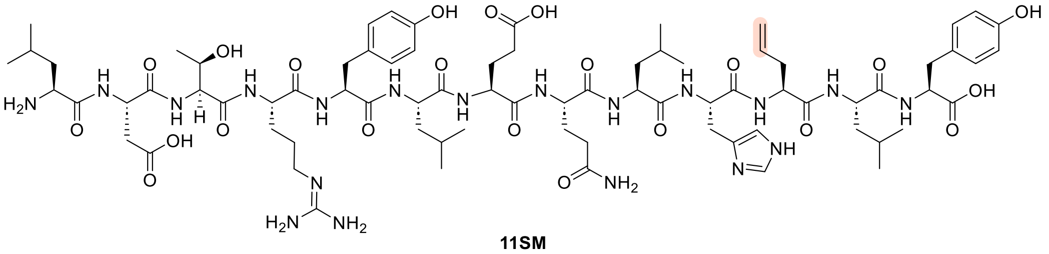

11SM
Chemical Formula: $C_{77}H_{117}N_{19}O_{22}$
Exact Mass: 1659.8621

LC-MS: ca. 88% UV-purity

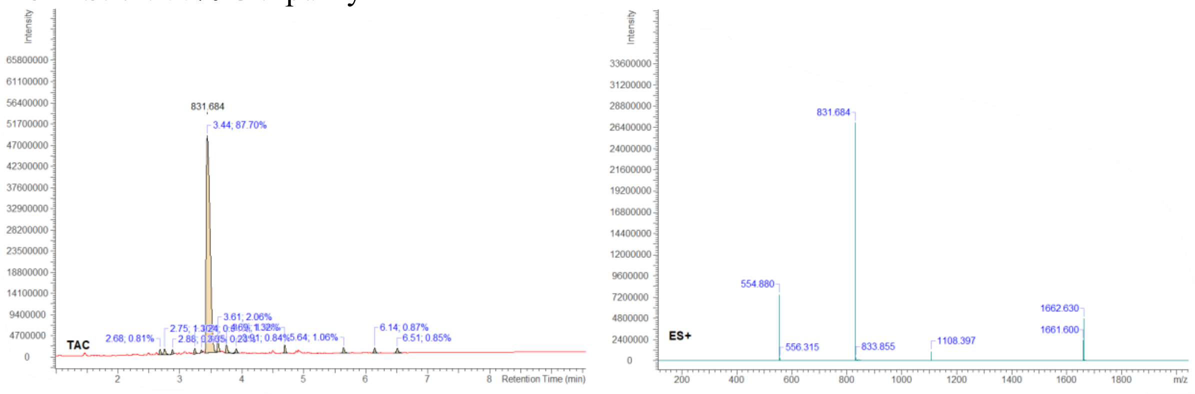

^{1}H-NMR (500 MHz, DMSO-d6): contains internal standard 1,3,5-TMB, and some Et_2O contamination

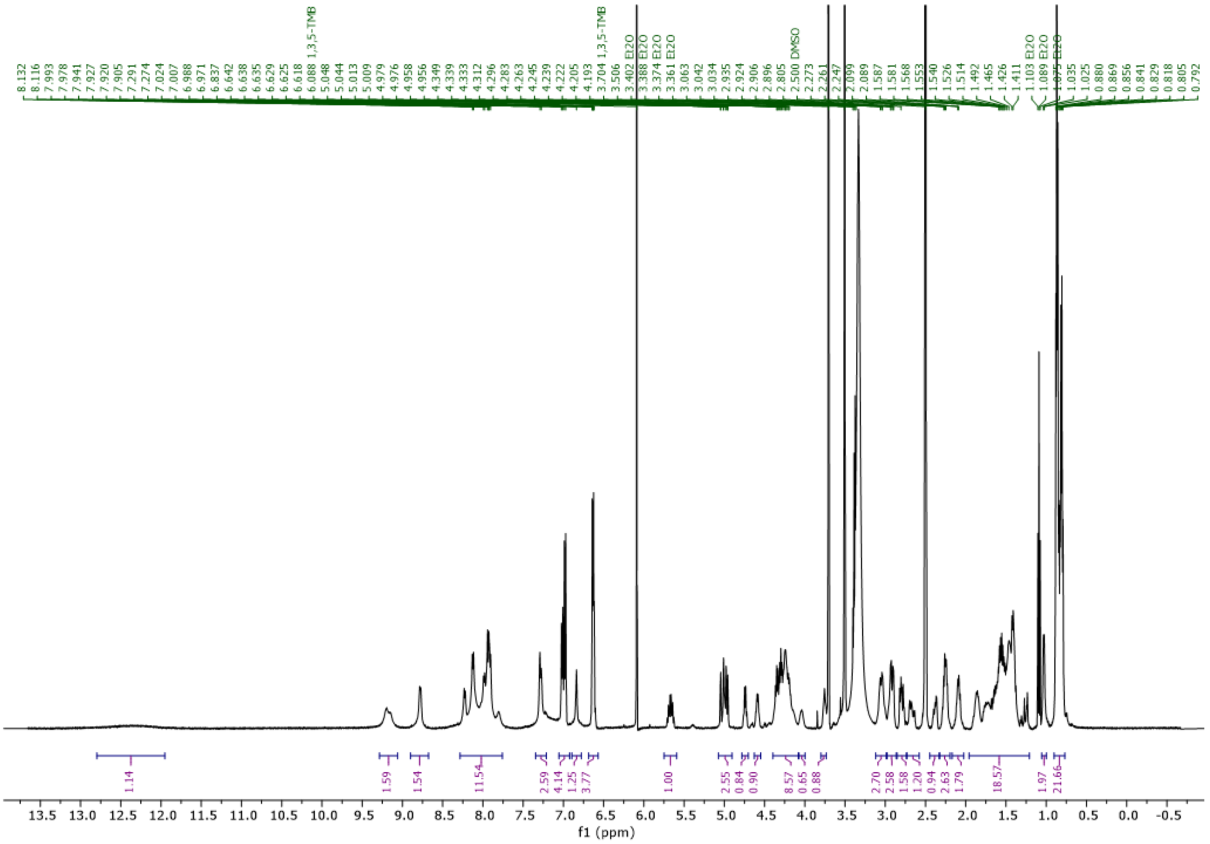

10.1.15 12SM

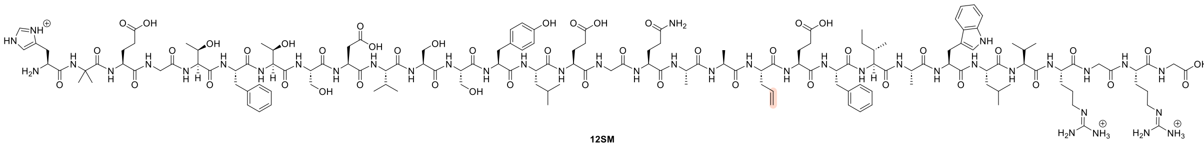

12SM

Chemical Formula: $C_{151}H_{228}N_{41}O_{47}^{3+}$

Exact Mass: 3367.6695

m/z: 1122.8910 (100.0%), 1123.2254 (81.1%), 1122.5565 (61.2%), 1123.5599 (43.6%), 1123.8943 (17.4%), 1123.2233 (15.0%), 1123.5578 (12.2%), 1123.5590 (9.7%), 1122.8889 (9.2%), 1123.8935 (7.8%), 1123.8922 (6.5%), 1123.2246 (5.9%), 1124.2288 (5.5%), 1124.2279 (4.2%), 1123.2264 (2.6%), 1124.2267 (2.6%), 1123.5608 (2.1%), 1123.2257 (1.8%), 1124.5624 (1.7%), 1122.8919 (1.6%), 1123.5602 (1.5%), 1124.5632 (1.4%), 1123.8914 (1.4%), 1124.2258 (1.2%), 1123.8953 (1.1%), 1122.8912 (1.1%), 1123.5557 (1.1%)

LC-MS of crude: ca. <64% UV-purity

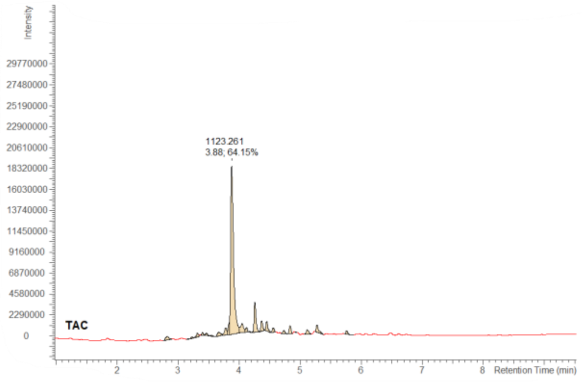

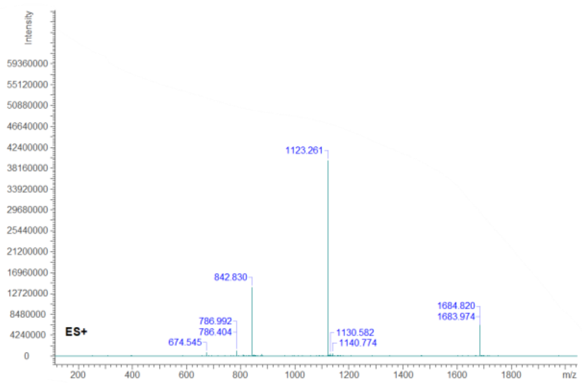

^{1}H-NMR (500 MHz, DMSO-d6) of **12SM**: qNMR with 0.5 equiv. 1,3,5-TMB assuming 0.079 mmol/g loading (actual olefin loading ca. 1.97 x 0.079 mmol/g=0.16 mmol/g, but likely many impurities with the allylglycine motif, therefore estimated loading ca. 0.079 mmol/g)

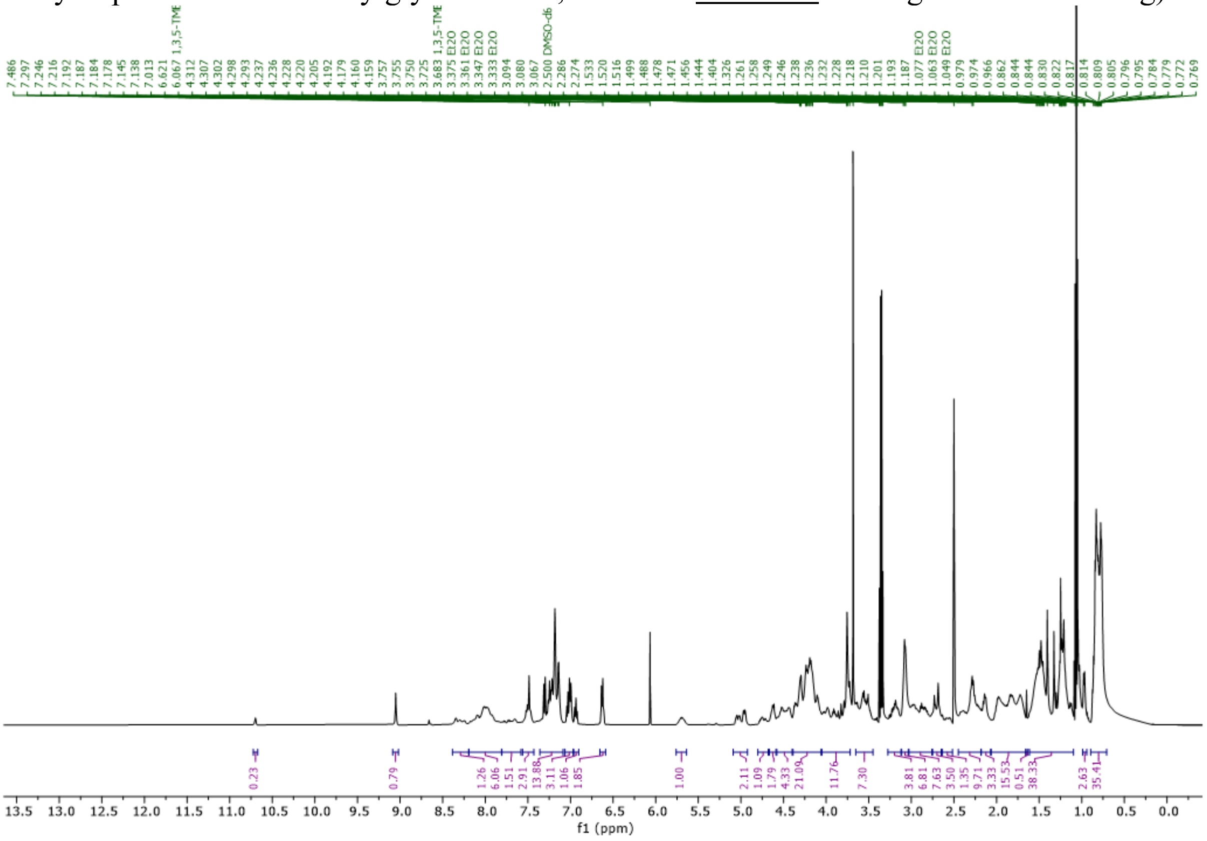

10.1.16 13SM

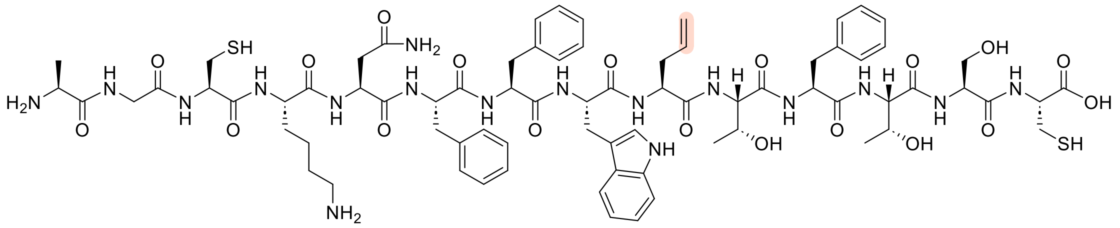

13SM
Chemical Formula: $C_{75}H_{101}N_{17}O_{19}S_2$
Exact Mass: 1607.6901

LC-MS of crude: ca. 87% UV-purity

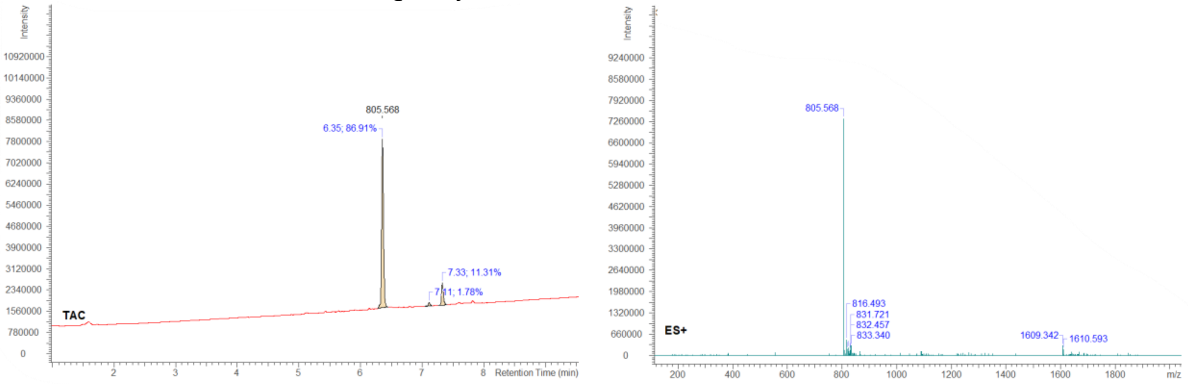

^{1}H-NMR (500 MHz, DMSO-d6): contains internal standard 1,3,5-TMB, and some Et_2O contamination

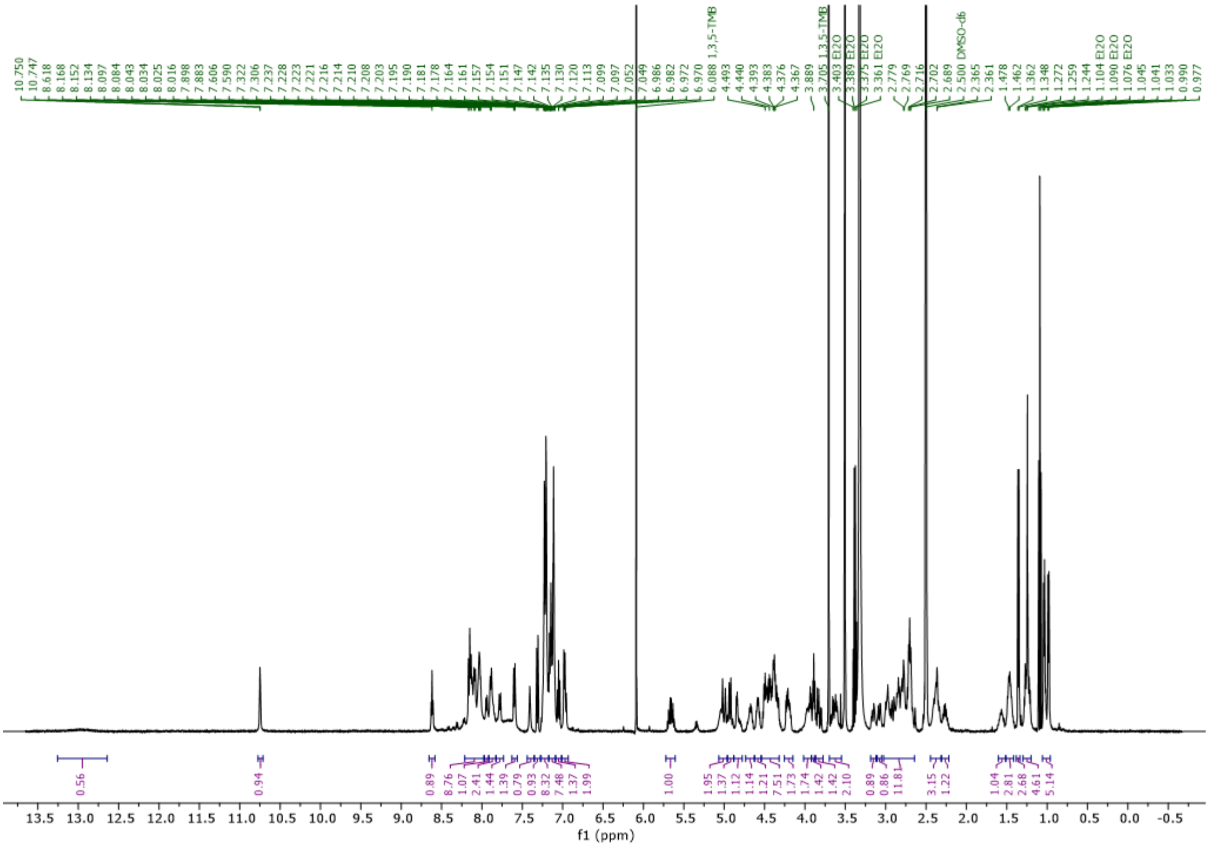

10.1.17 14SM

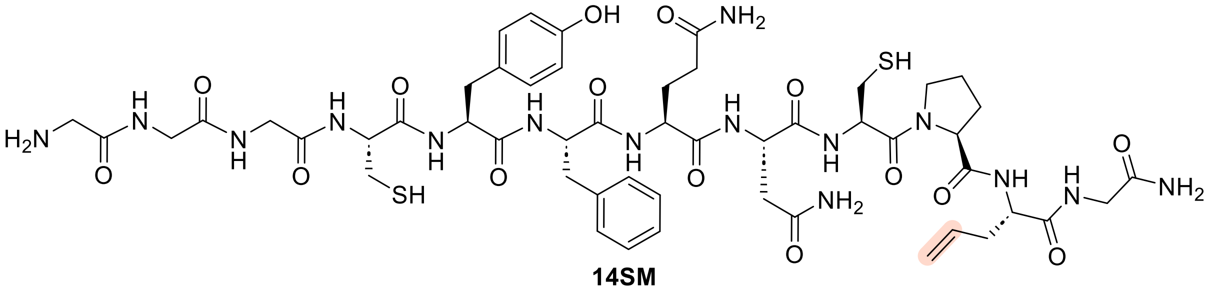

14SM

Chemical Formula: $C_{51}H_{71}N_{15}O_{15}S_2$

Exact Mass: 1197.4695

LC-MS of crude: ca. 89% UV-purity (shows m/z of [M+TFA]$^-$)

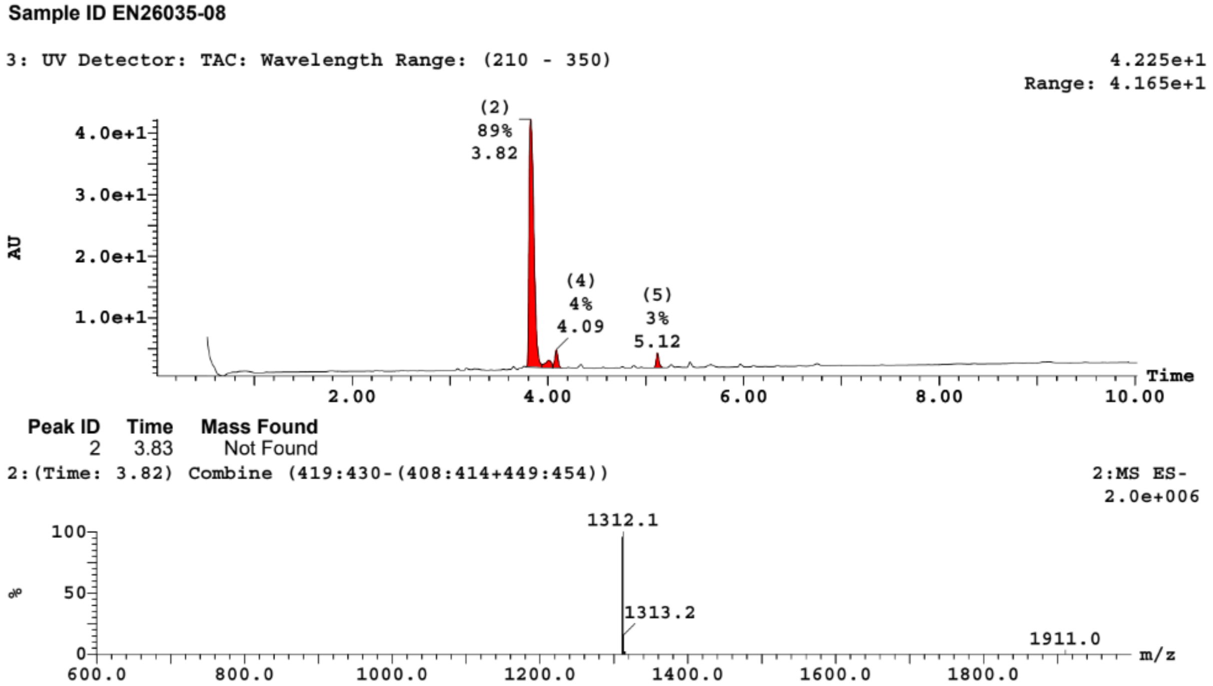

^{1}H-NMR: contains internal standard 1,3,5-TMB, and some Et_2O contamination, and likely many other impurities

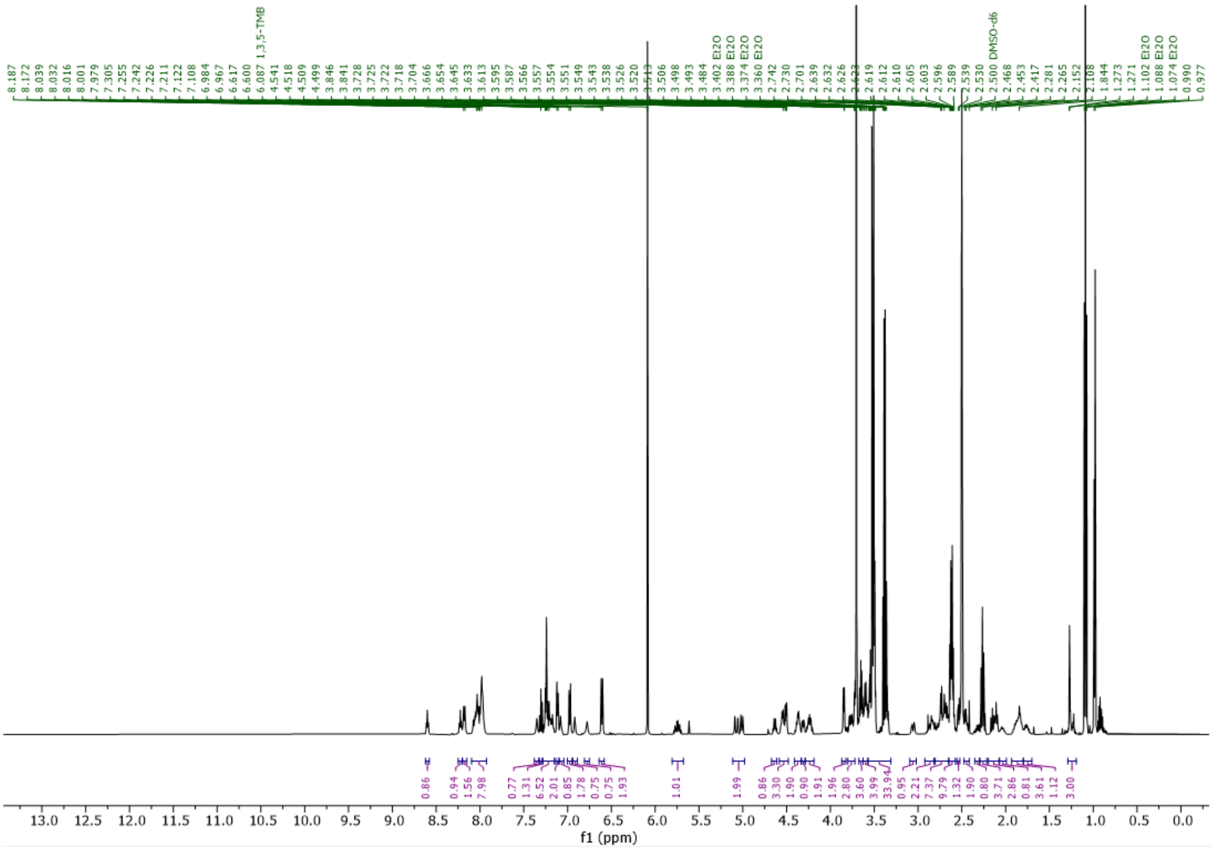

10.1.18 15SM

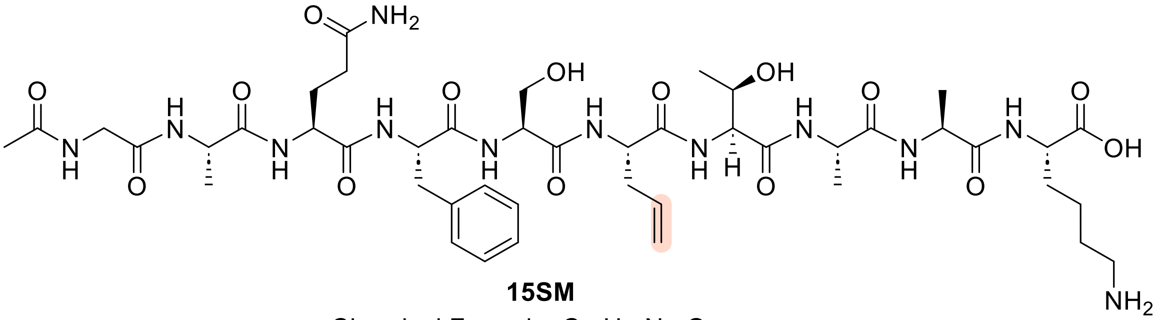

15SM

Chemical Formula: $C_{45}H_{70}N_{12}O_{15}$

Exact Mass: 1018.5084

LC-MS of crude: ca. 98% UV-purity

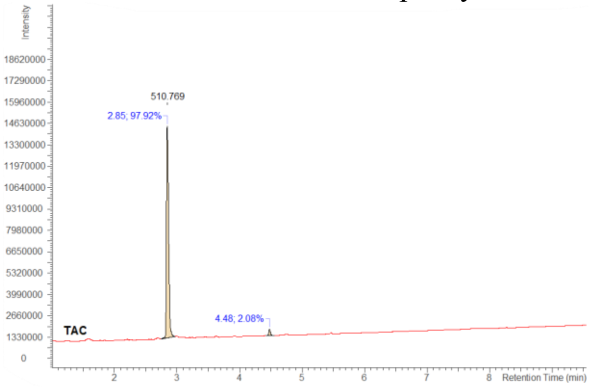

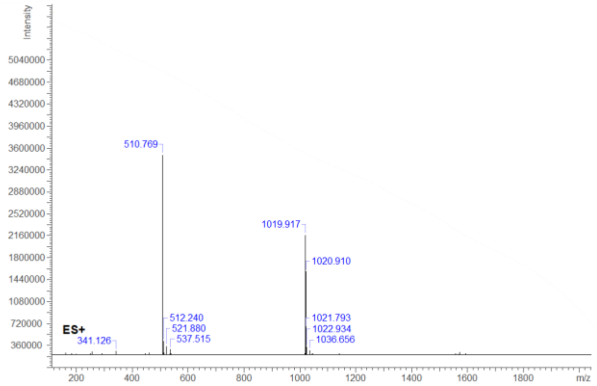

^{1}H-NMR (500 MHz, DMSO-d6): contains internal standard 1,3,5-TMB, and some Et_2O contamination

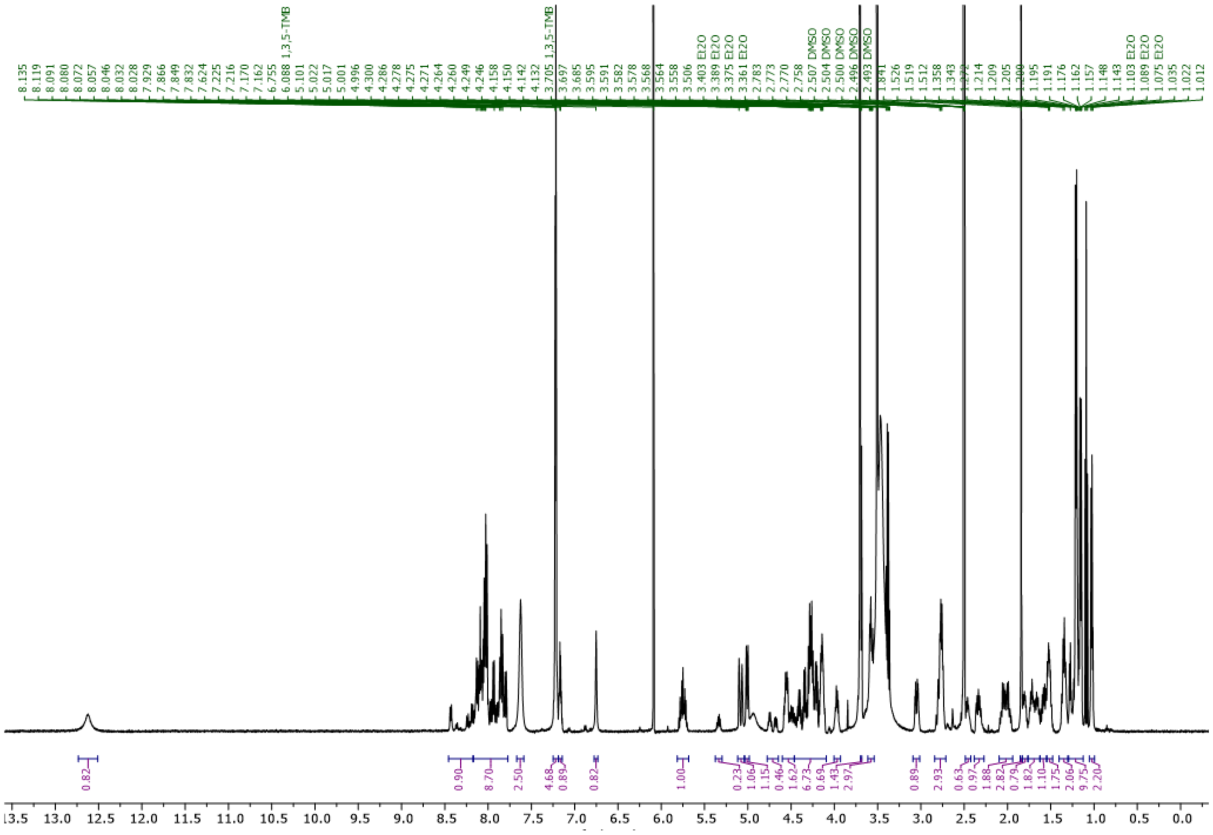

10.2 NMR/LC-MS spectra of final peptides

10.2.1 Tripeptides

SI-3A

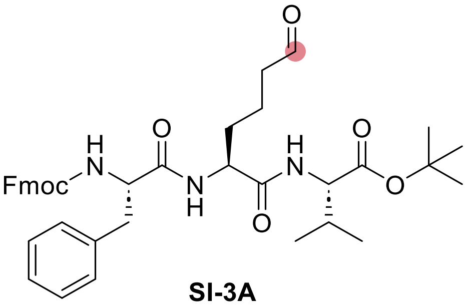

SI-3A

Chemical Formula: $C_{39}H_{47}N_3O_7$

Exact Mass: 669.3414

qNMR:

1,3,5-TMB: 0.012 mmol added to 0.015 mmol sample

*t*Bu normalized to 9H, ca. 86% yield (2x aldehydes, 70% and 16%), conversion ca 90%

^{1}H-NMR (500 MHz, DMSO-d6)

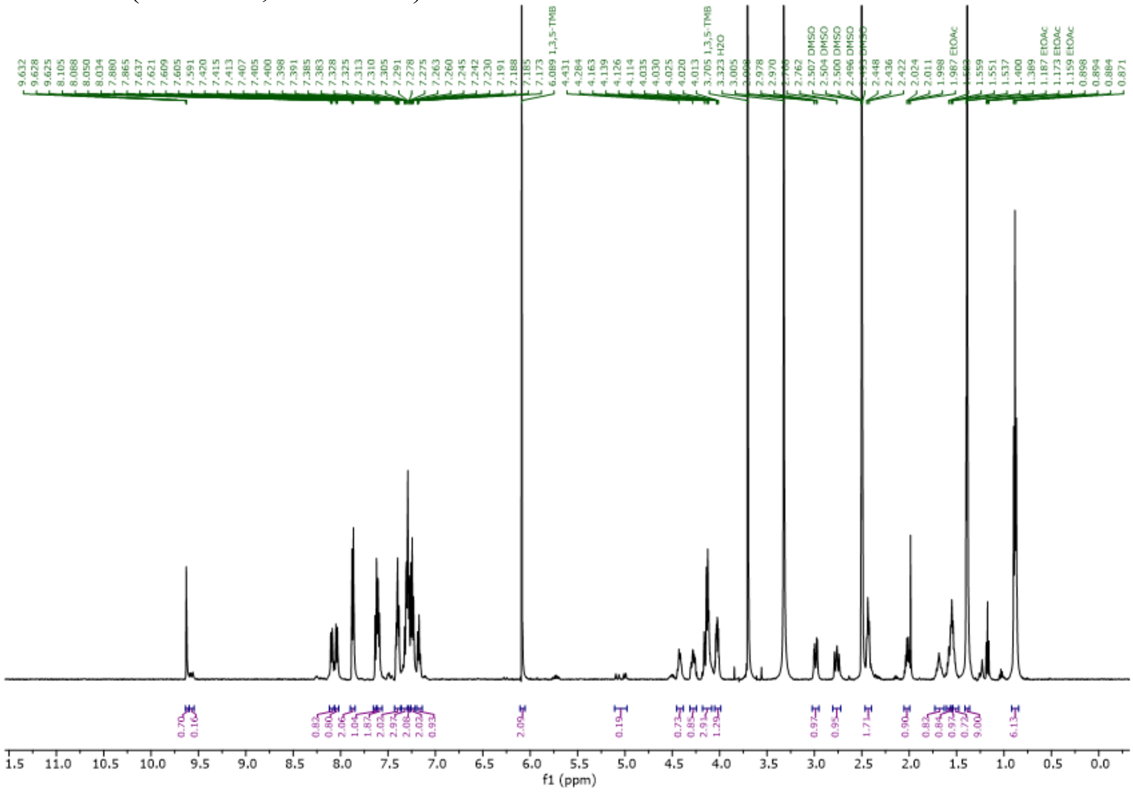

SI-4A

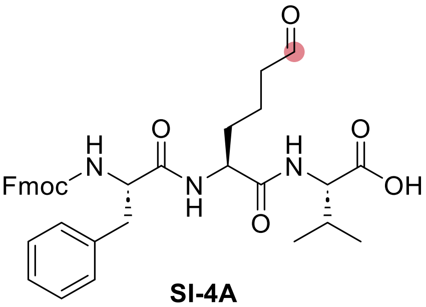

SI-4A

Chemical Formula: $C_{35}H_{39}N_3O_7$

Exact Mass: 613.2788

qNMR: ^{1}H-NMR

1 equiv. of 1,3,5-TMB added, in this spectrum: Valin signal was set to integral=6

^{1}H-NMR (600 MHz, DMSO-d6)

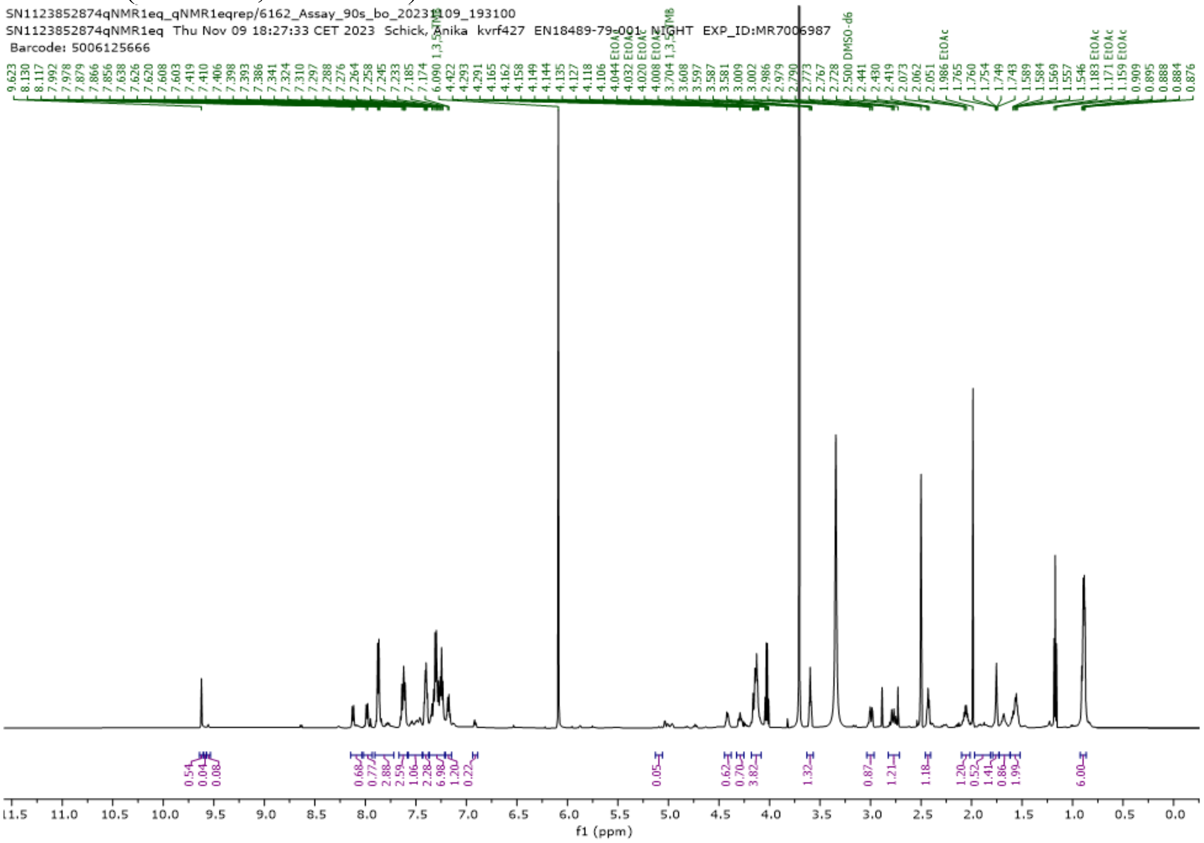

10.2.2 Hexapeptide 1C

[^{12}C]1C

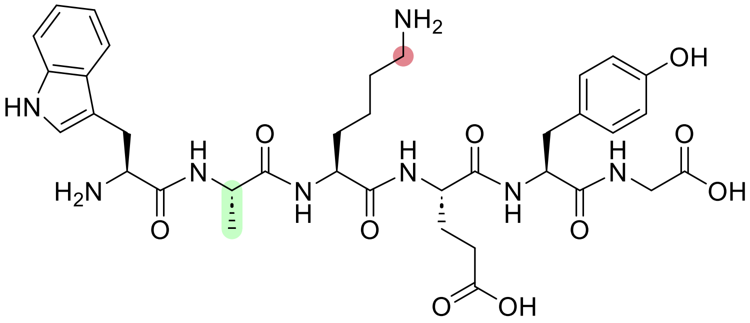

[^{12}C]1C

Chemical Formula: $C_{36}H_{48}N_8O_{10}$

Exact Mass: 752.3493

LC-MS of crude: ca. 82% UV-purity (2x product peak, respectively 76% and 6%)

Sample ID EN6035-61-RADODT2

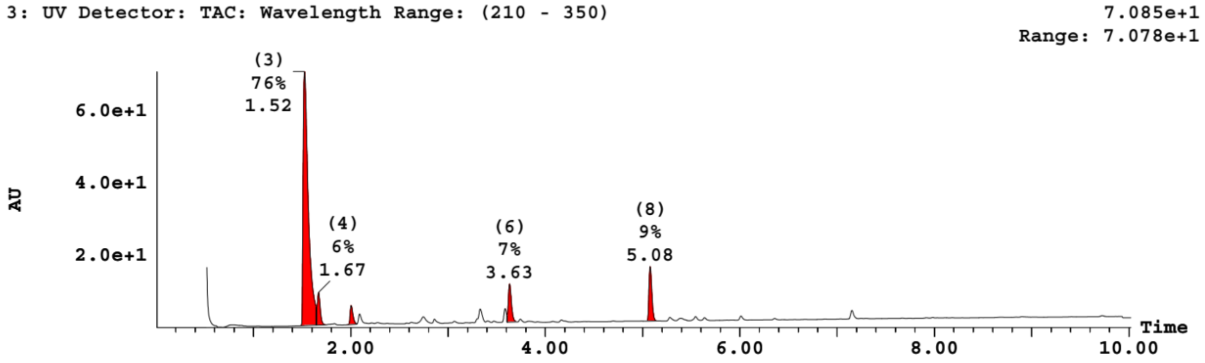

Peak ID	Time	Mass Found
3	1.56	Not Found

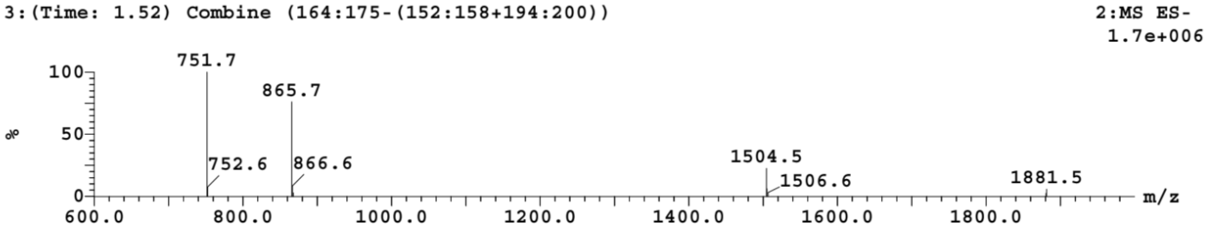

LC-MS of purified product: ca. >99% UV-purity

Sample ID EN6035-61_PURE

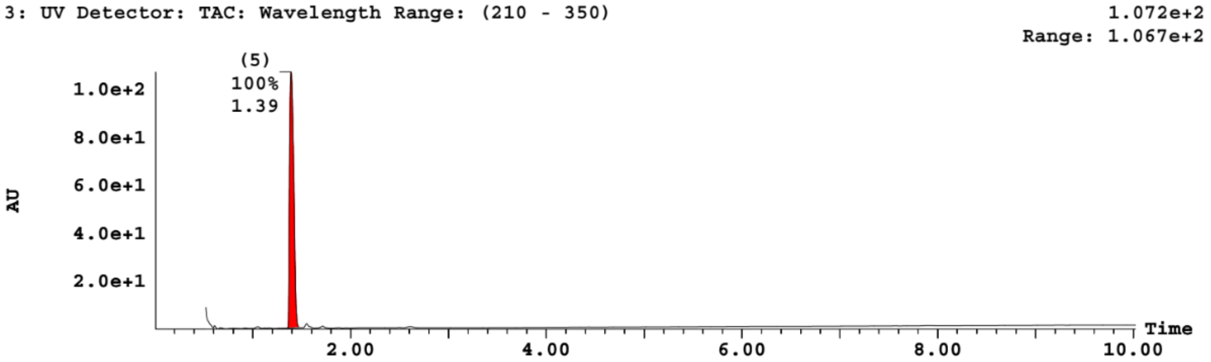

NMR of purified product: ^{1}H-NMR, ^{13}C-NMR

^{1}H-NMR (600 MHz, DMSO-d6)

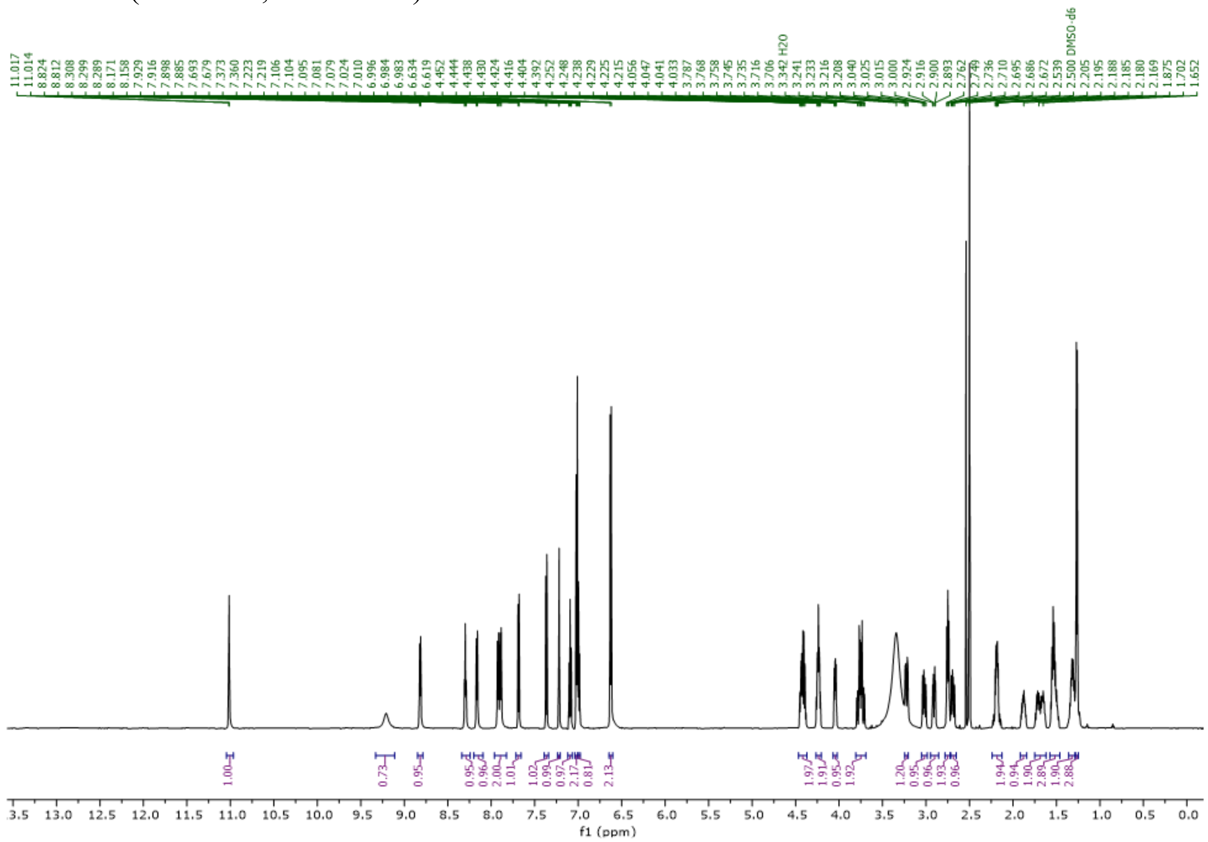

^{13}C-NMR (151 MHz, DMSO-d6)

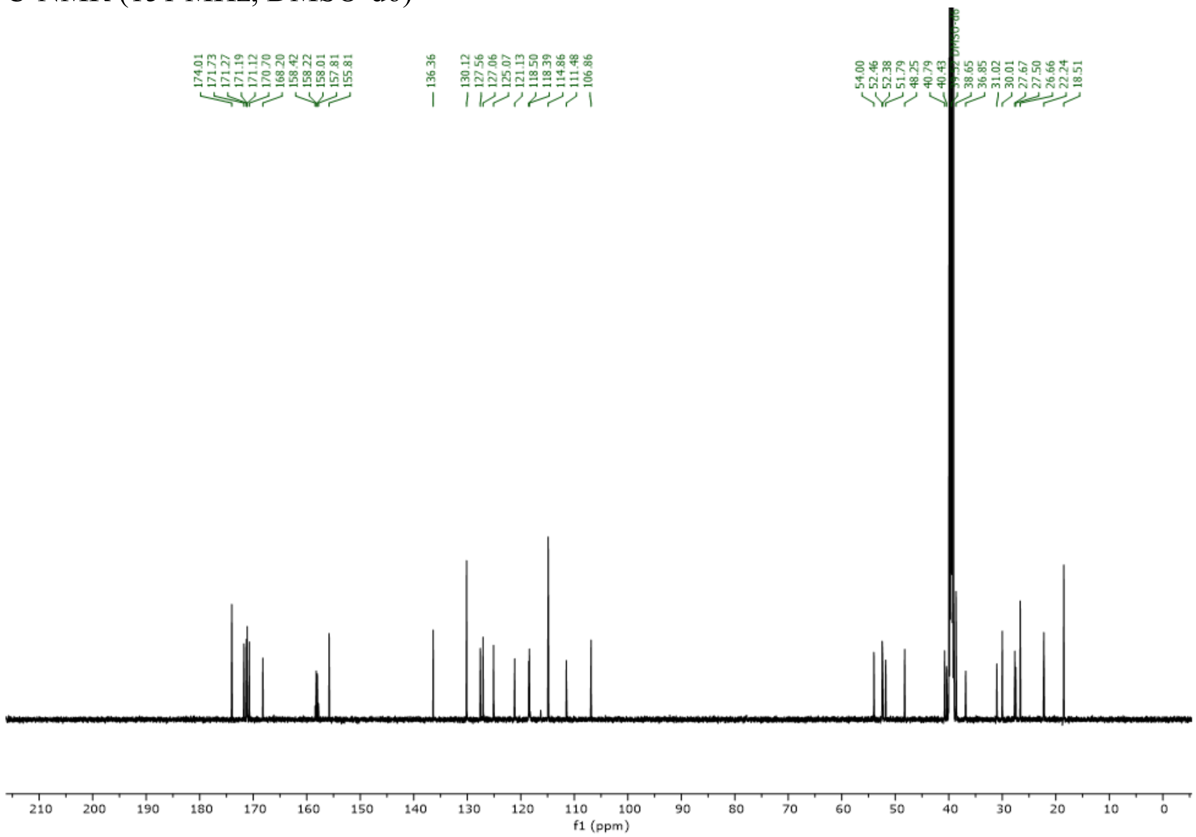

10.2.3 Hexapeptide 2C

[12C]2C

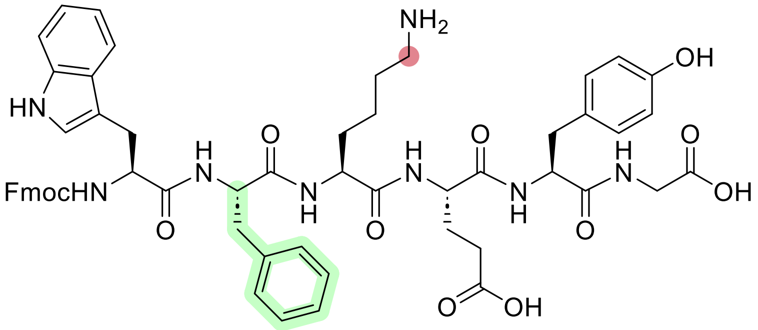

[12C]2C

Chemical Formula: $C_{57}H_{62}N_8O_{12}$

Exact Mass: 1050.4487

LC-MS of crude: ca. 84% UV-purity

Sample ID EN6035-39-RADODT

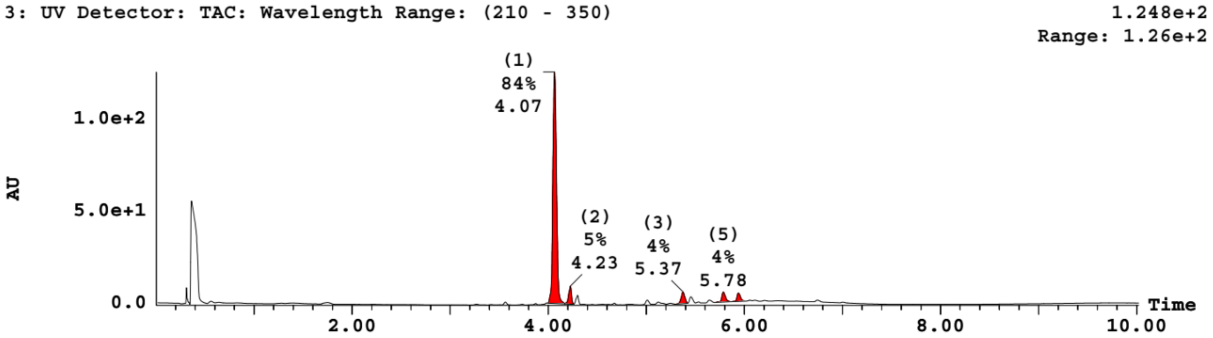

Peak ID	Time	Mass Found
1	4.09	Not Found

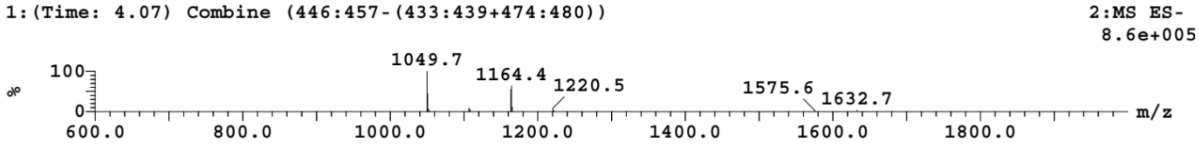

LC-MS of purified product: ca. 96% UV-purity

Sample ID EN6035-39-PURE

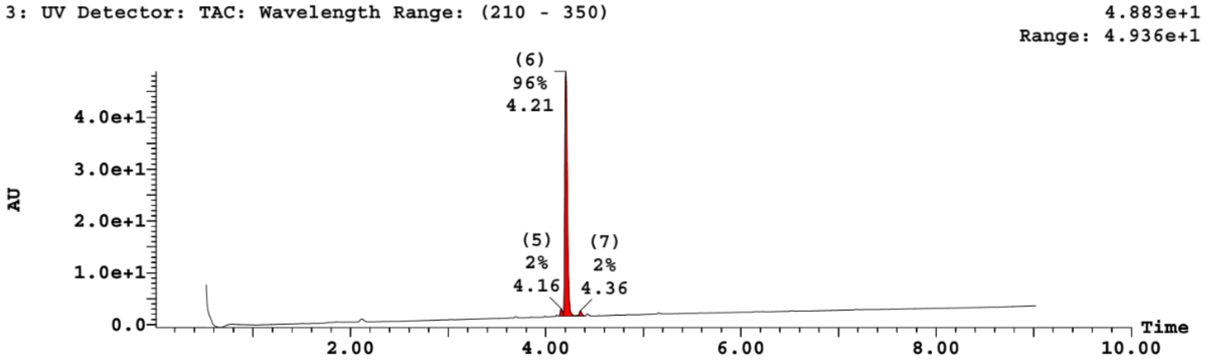

NMR of purified product: ^{1}H-NMR, ^{13}C-NMR

^{1}H-NMR (600 MHz, DMSO-d6), contains PEG signal at 3.5 ppm that could not be removed by purification

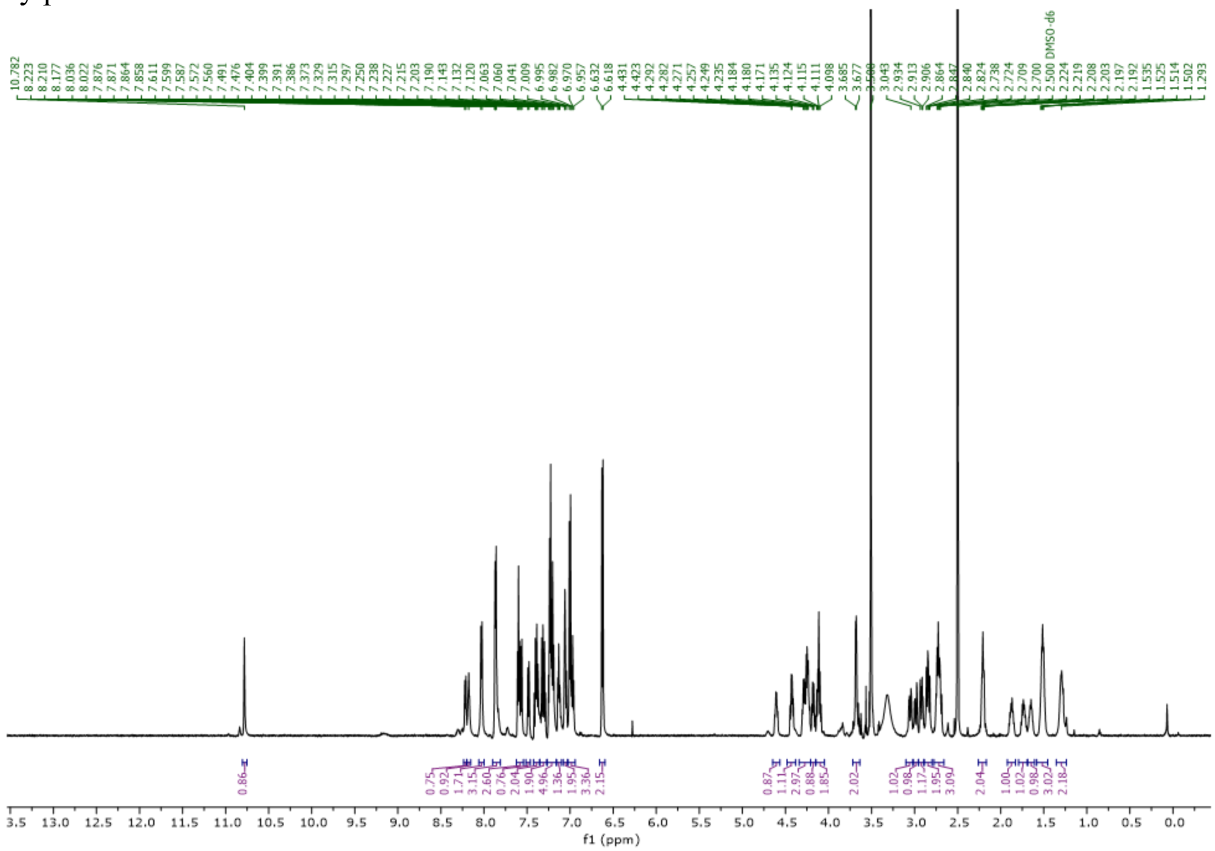

^{13}C-NMR (151 MHz, DMSO-d6), PEG signal at 69.79 ppm

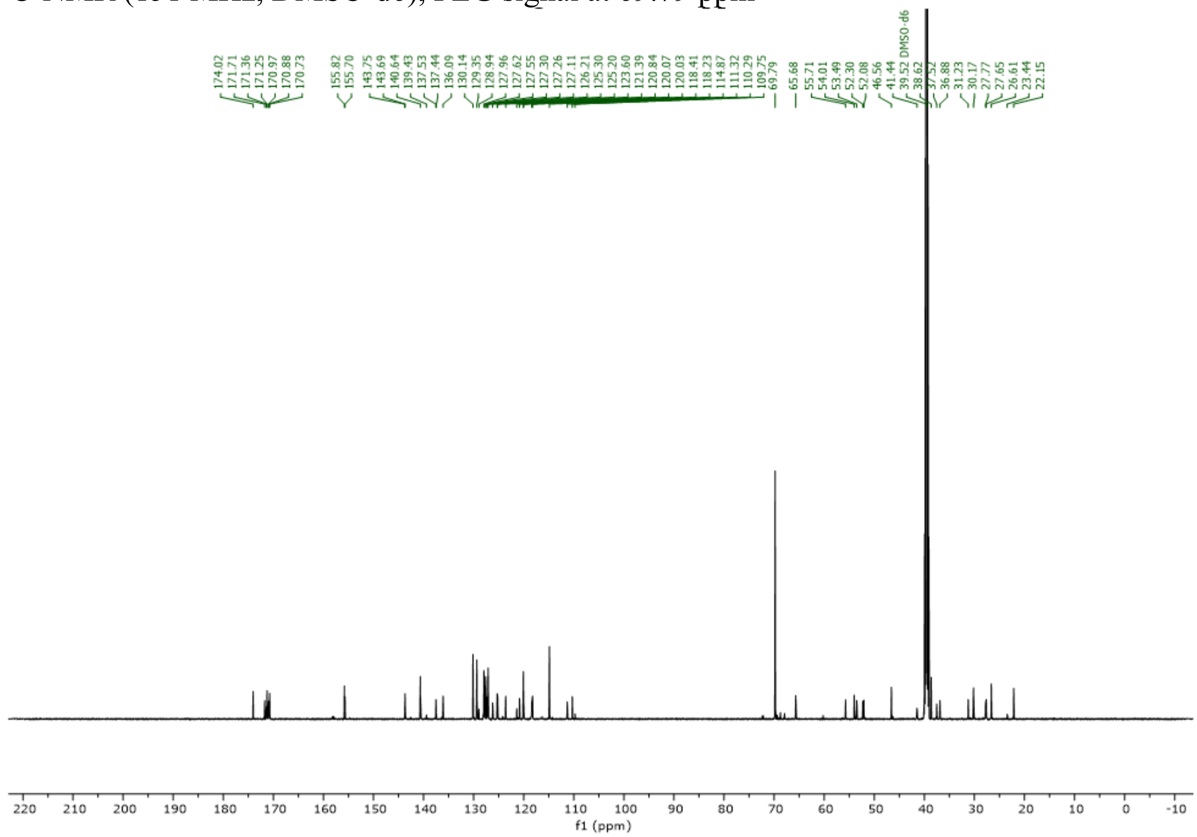

[^{13}C]2C

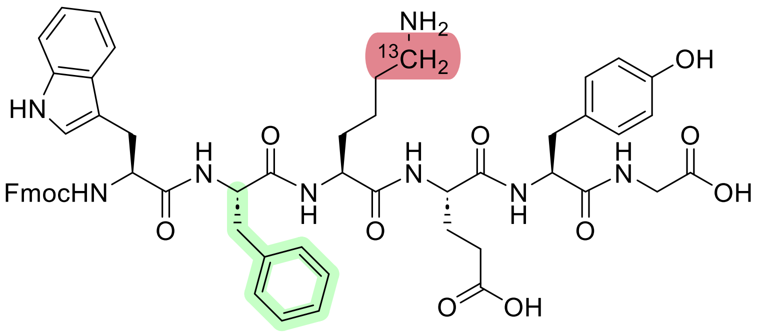

[^{13}C]2C

Chemical Formula: $C_{56}{}^{13}CH_{62}N_8O_{12}$

Exact Mass: 1051.4521

LC-MS of crude: ca. 79% UV-purity

Sample ID EN6035-55-RADODT

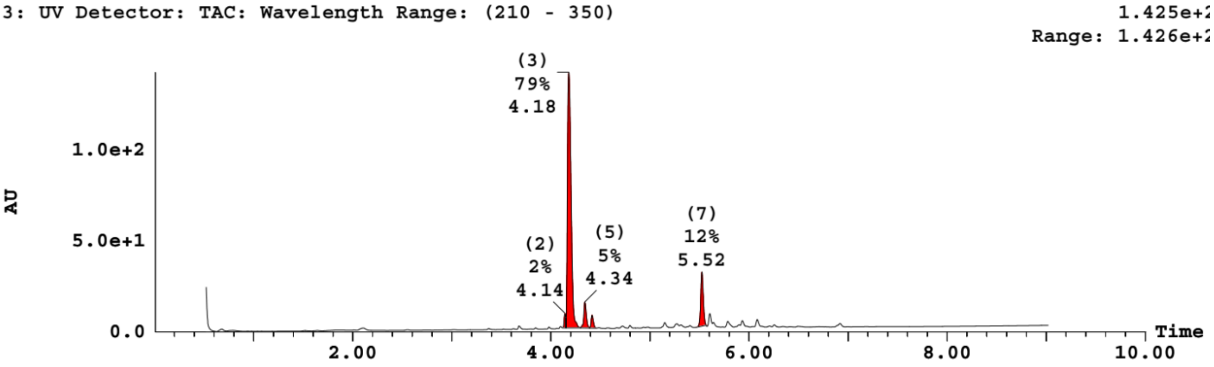

Peak ID	Time	Mass Found
3	4.19	Not Found

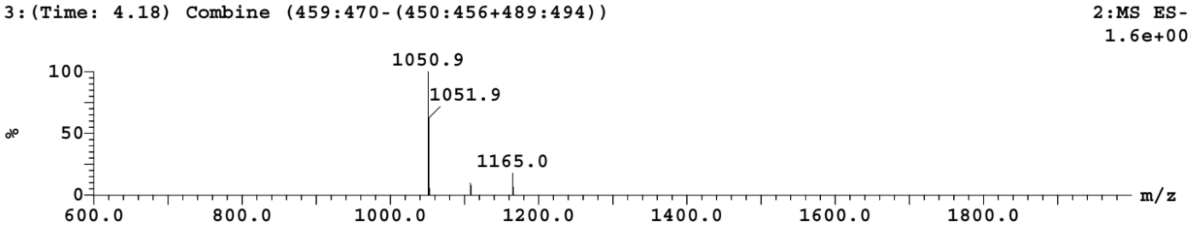

LC-MS of purified product: ca. >99% UV-purity

Sample ID EN6035-55_PURE2

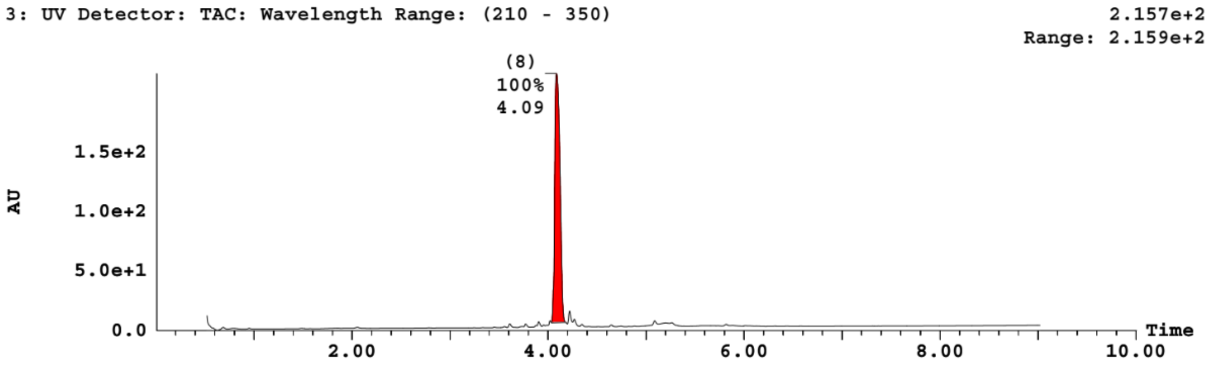

Isotope distribution comparison: Predicted non-labeled vs labeled peptide, ca. >0.93 ^{13}C/molecule incorporation according to calculations (actual incorporation should be 0.99)

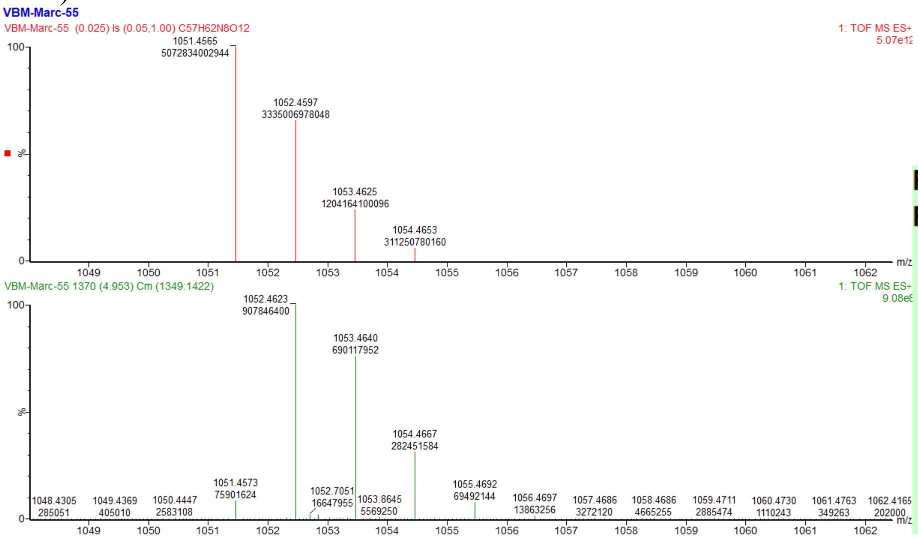

Results	
Relative amounts [%]	
unlabeled	7.3
1-label	82.8
2-label	10.4
3-label	0.3
4-label	-1.0
5-label	0.0
6-label	0.2
7-label	0.0
8-label	0.0

^{1}H-NMR (600 MHz, DMSO-d6), contains PEG signal at 3.5 ppm that could not be removed by purification

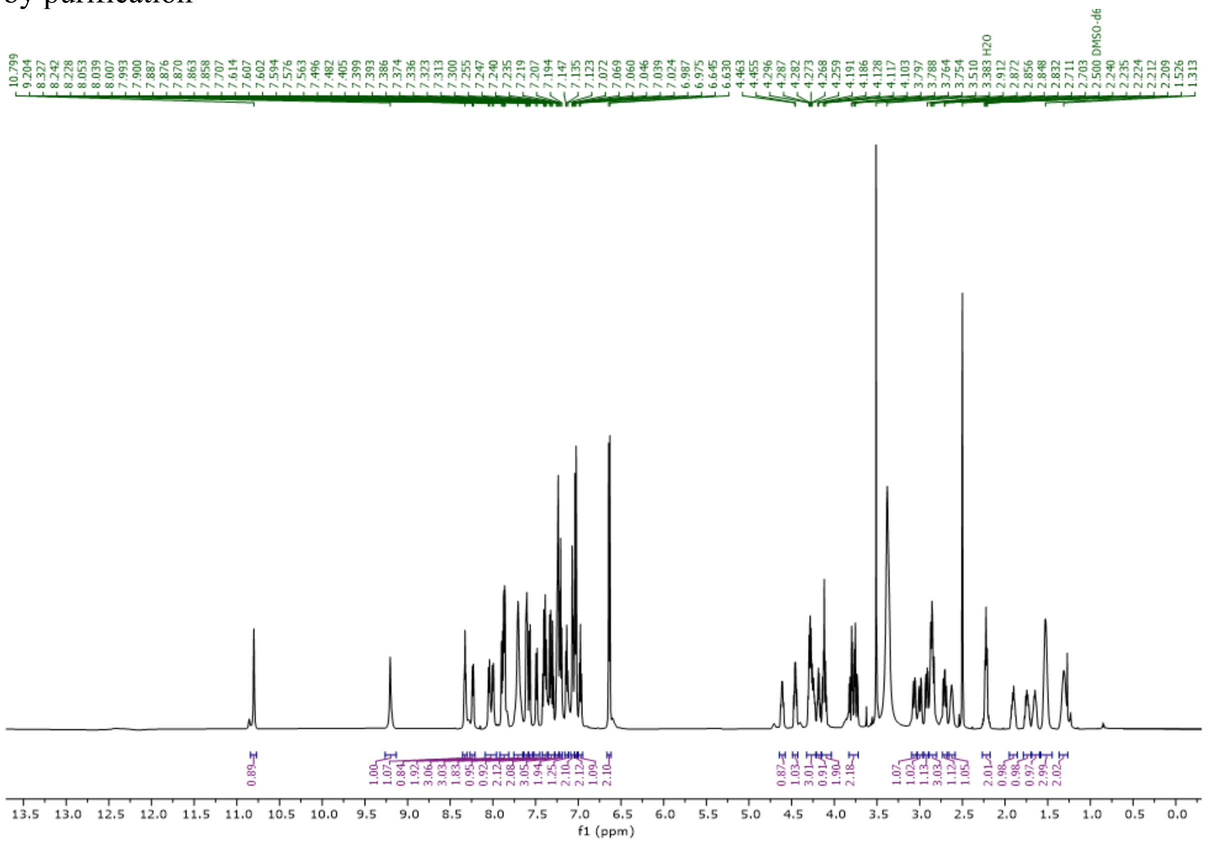

^{13}C-NMR (151 MHz, DMSO-d6): ^{13}C-label at 38.72 ppm, PEG signal at 69.81 ppm

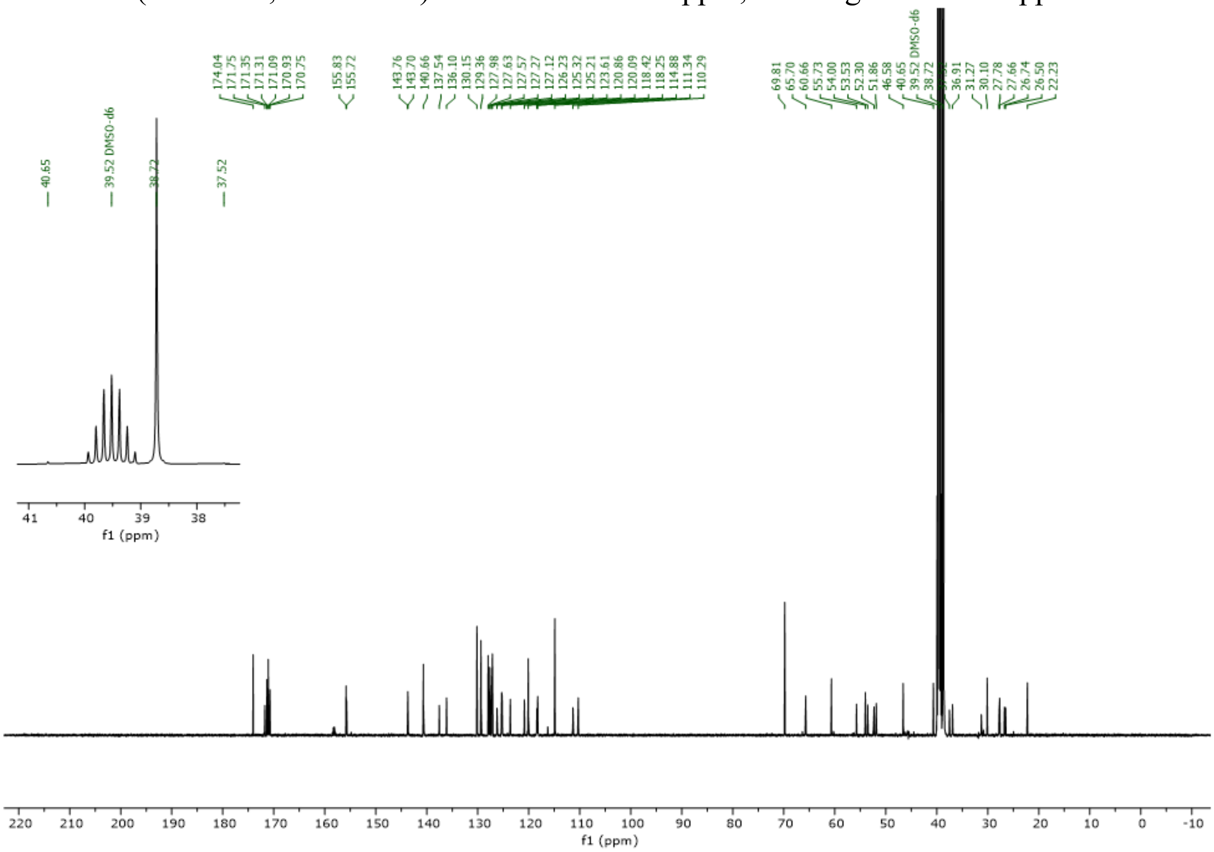

[^{13}C]2C (0.36 equiv. cat., not described in main manuscript, but includes 2D-NMR data)

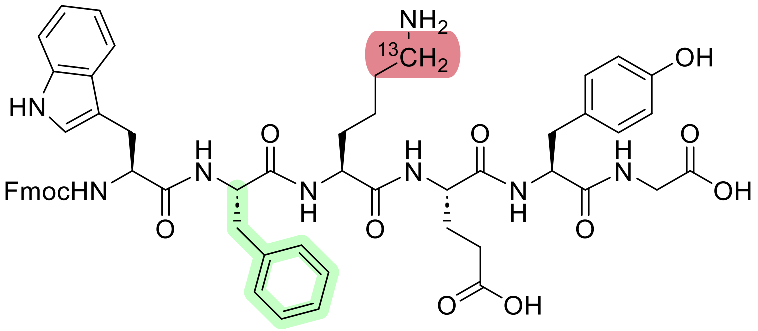

[^{13}C]2C

Chemical Formula: $C_{56}{}^{13}CH_{62}N_8O_{12}$

Exact Mass: 1051.4521

LC-MS of crude: ca. 58% UV-purity

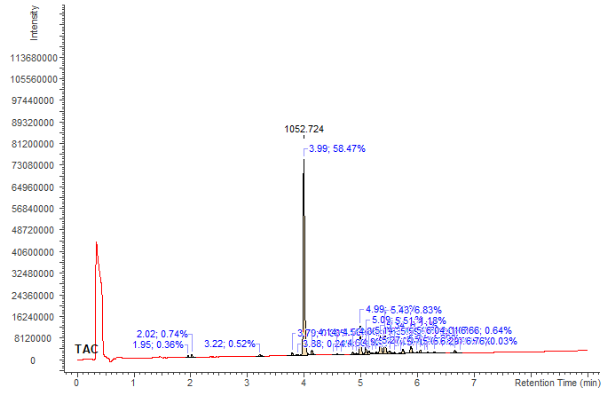

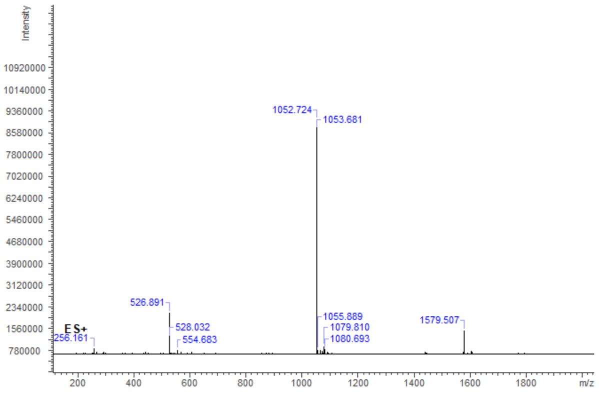

LC-MS of purified product: ca. 98% UV-purity

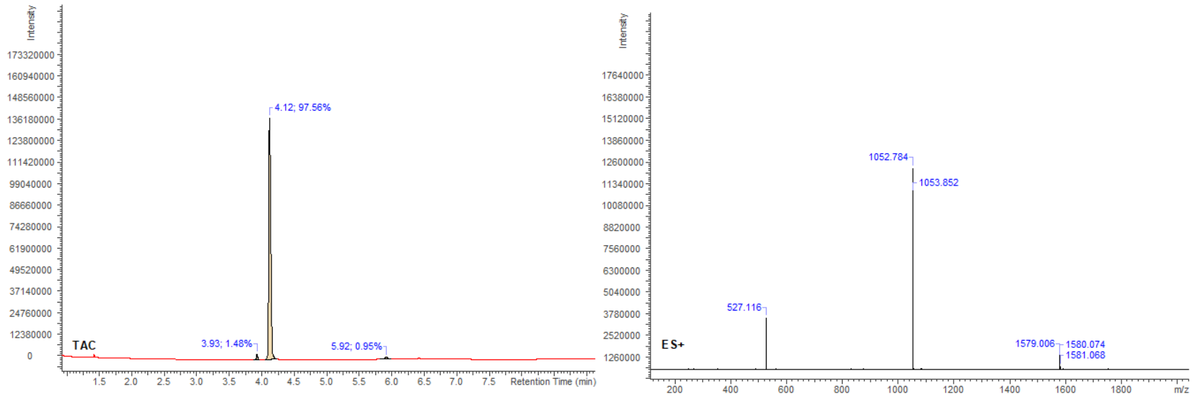

Isotope distribution comparison: Predicted non-labeled vs labeled peptide, ca. 0.99 ^{13}C/molecule incorporation

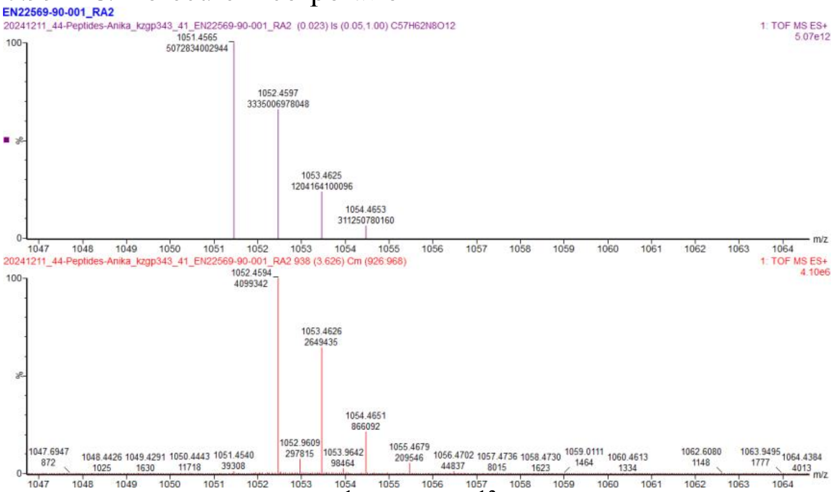

Results	
Relative amounts [%]	
unlabeled	1.0
1-label	101.0
2-label	-0.9
3-label	-1.9
4-label	0.5
5-label	1.3
6-label	-0.9

NMR of purified product: ^{1}H-NMR, ^{13}C-NMR, HSQC, HMBC, COSY

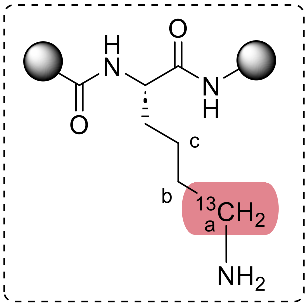

→it should be noted that the product was instable at room temperature in DMSO, leading to Fmoc-deprotection

a: ^{13}C at 38.58 ppm, ^{1}H crosslink at 2.73 ppm (HSQC)
b and **c**: ^{1}H at 1.54 ppm and 1.30 ppm (HMBC)

^{1}H-NMR (500 MHz, DMSO-d6), contains PEG signal at 3.5 ppm that could not be removed by purification

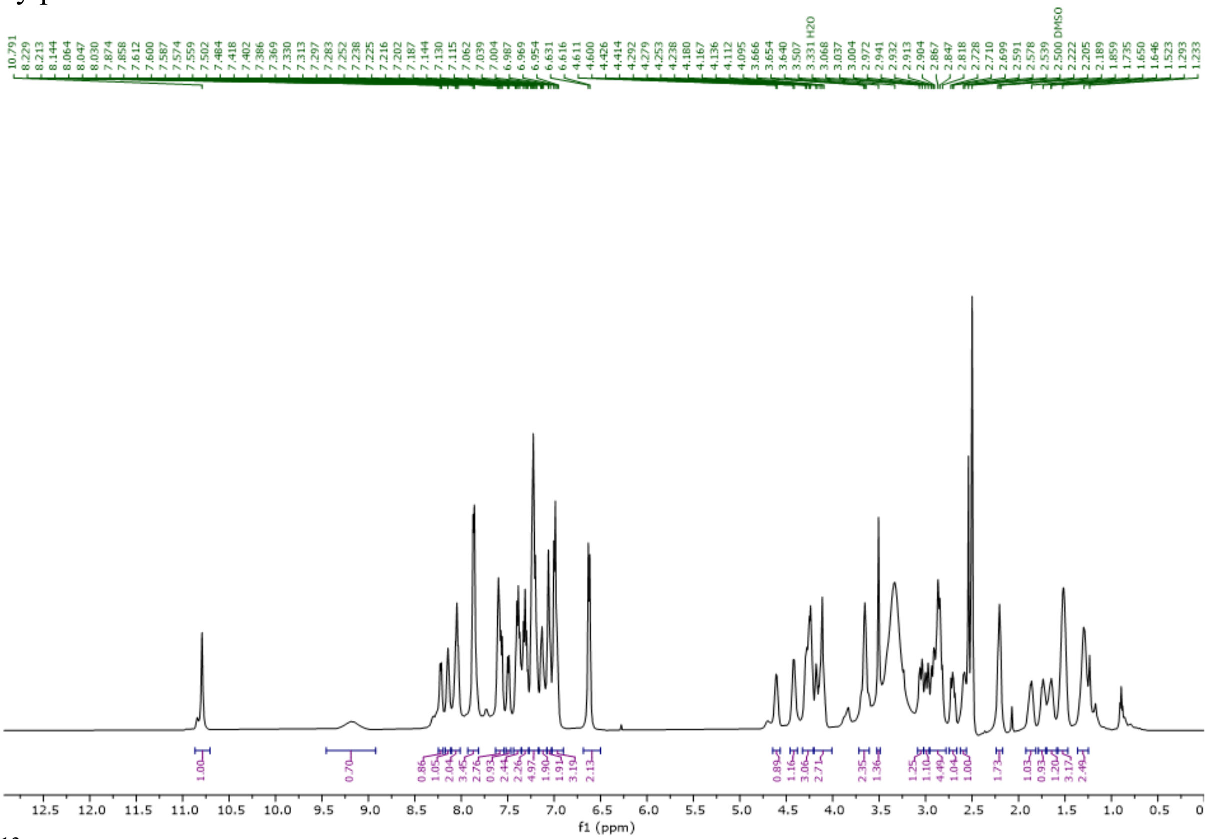

^{13}C-NMR (126 MHz, DMSO-d6)

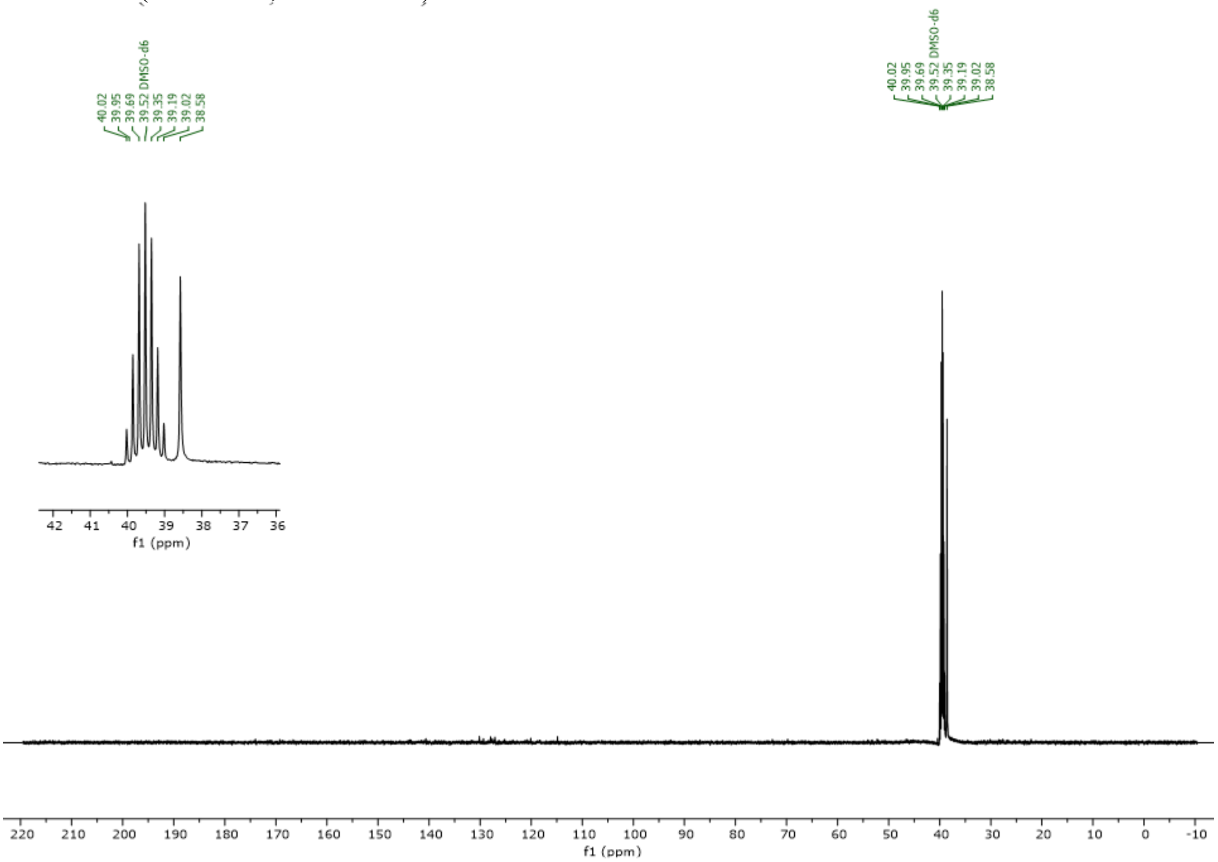

HSQC (500 MHz, DMSO-d6) →some Fmoc-deprotection visible

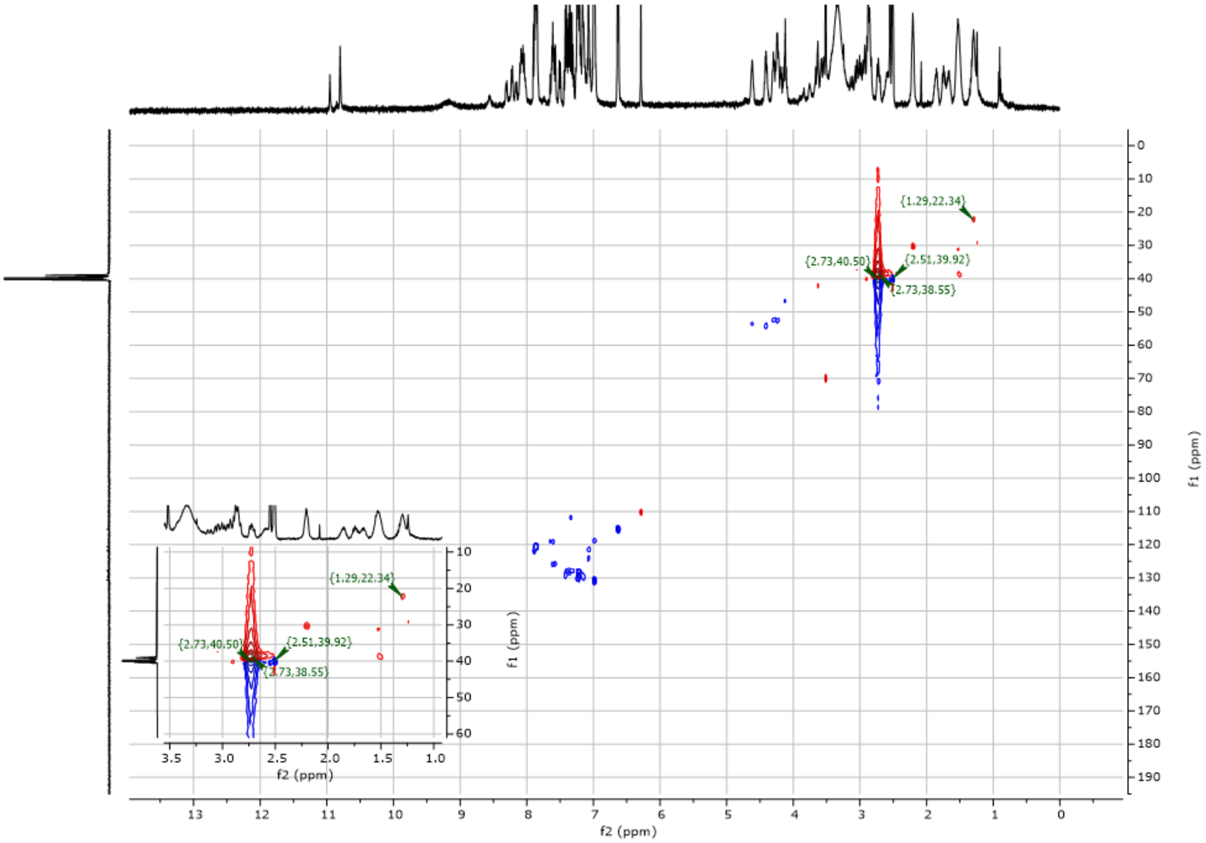

HMBC (500 MHz, DMSO-d6) →some Fmoc-deprotection visible

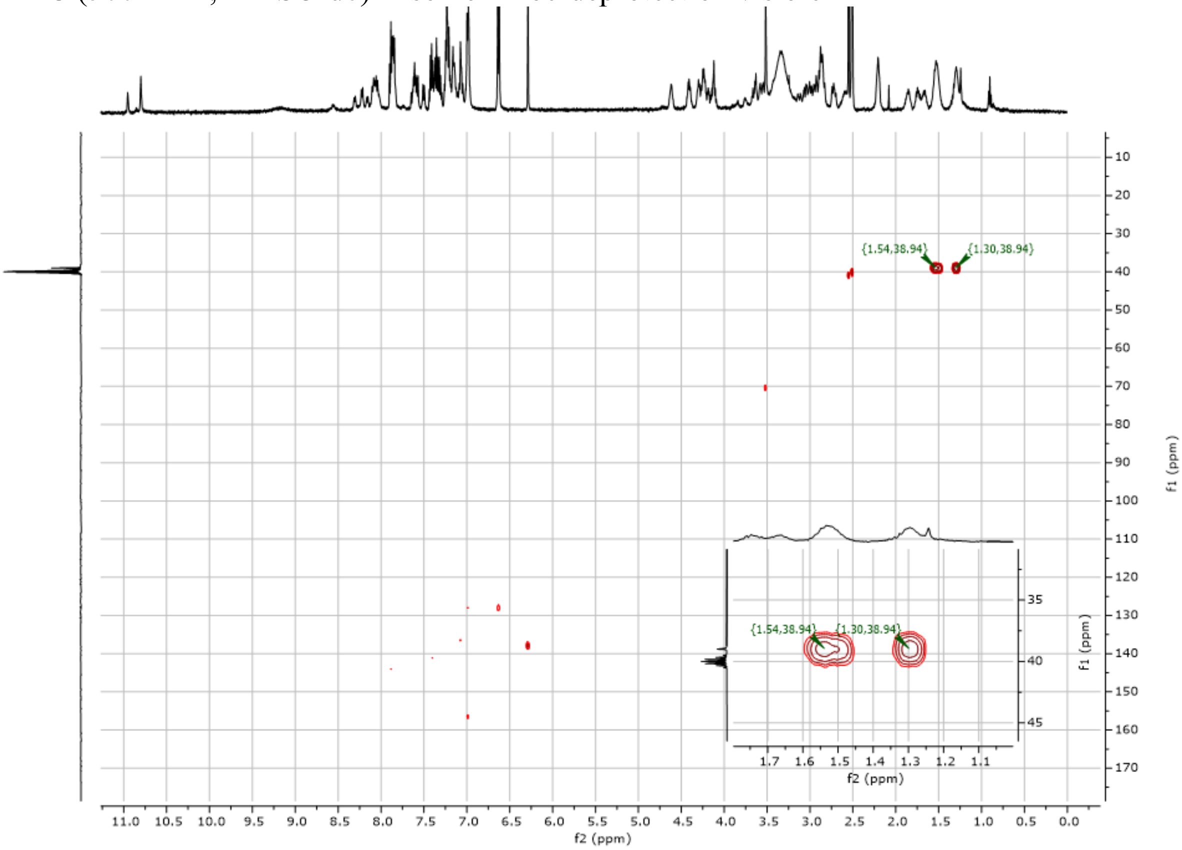

COSY (500 MHz, DMSO-d6) →some Fmoc-deprotection visible

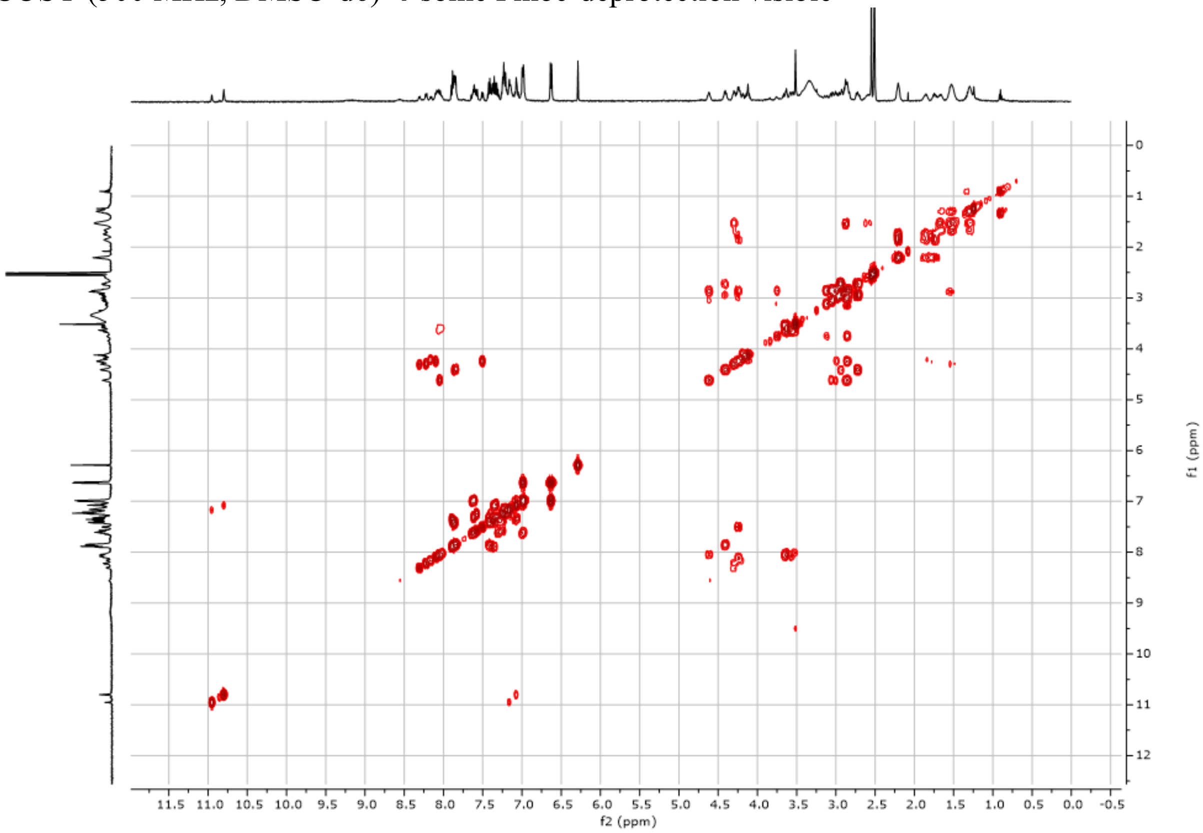

[^{14}C]2C

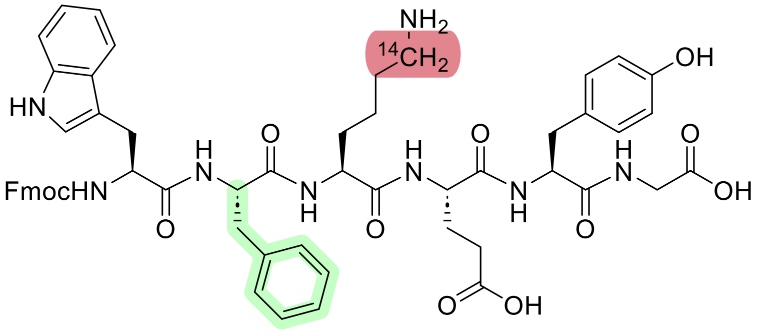

[^{14}C]2C

Chemical Formula: $C_{56}{}^{14}CH_{62}N_8O_{12}$

Exact Mass: 1052.4520

LC-MS of crude: ca. 33% UV-purity
(Note: UV chromatogram shows test portion of crude resin cleaved with TFA:EDT:H2O, 92.5:5:2.5)

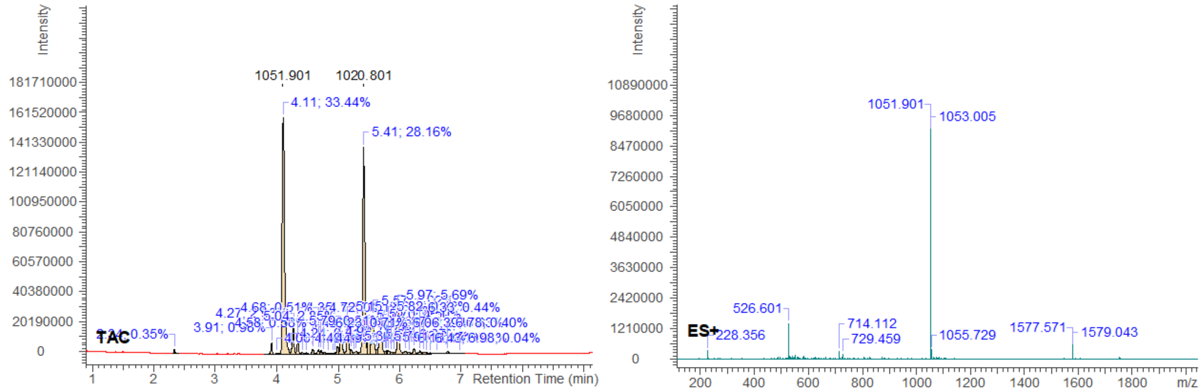

Radio-HPLC of crude: 62% radiochemical purity

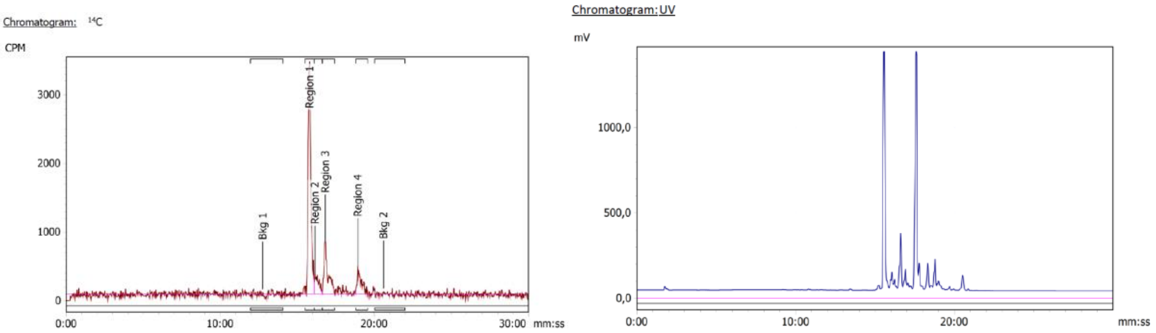

LC-MS of purified product: 96% UV-purity

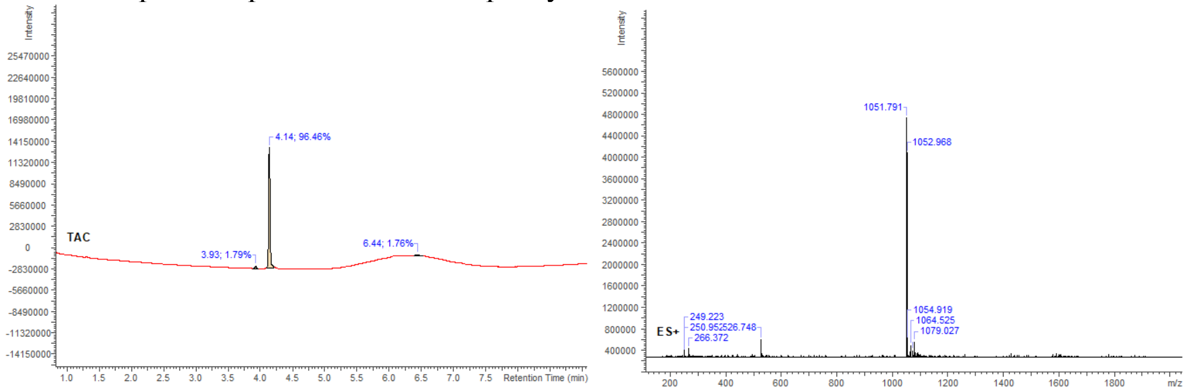

Isotope distribution comparison: Predicted non-labeled vs labeled peptide, ca. 0.05 ^{14}C/molecule incorporation (with peak separation=1)

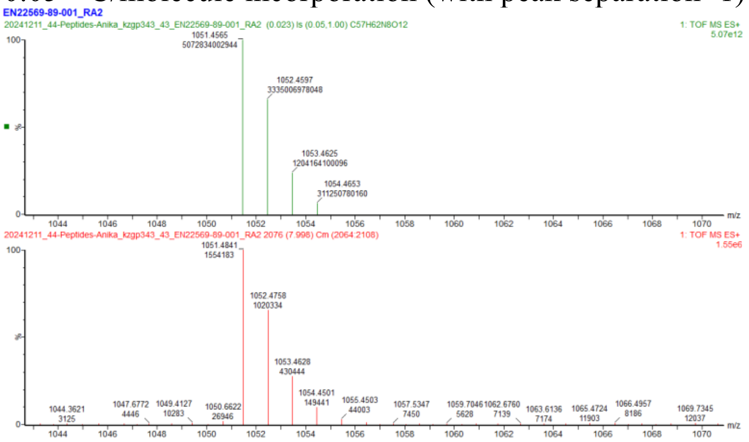

Results	
Relative amounts [%]	
unlabeled	95.0
0.5-label	-0.1
1-label	3.8
1.5-label	0.8
2-label	1.3
2.5-label	-1.3
3-label	0.5

Radio-HPLC of purified product: 94% radiochemical purity

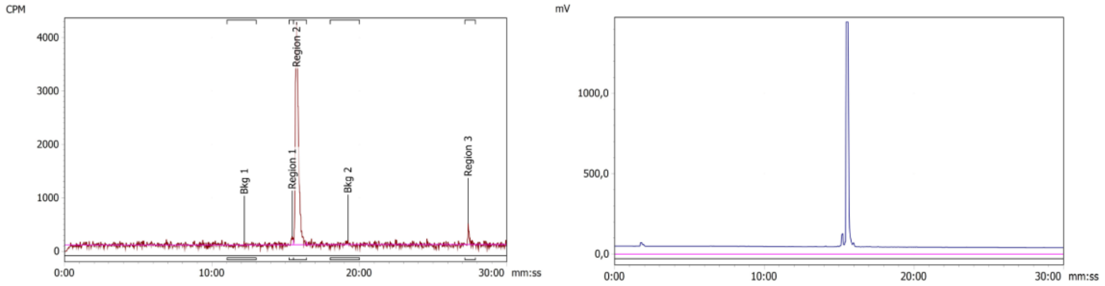

NMR of purified product: ^{1}H-NMR, ^{13}C-NMR, HSQC, HMBC, COSY

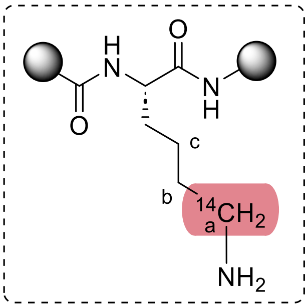

^{1}H-NMR (500 MHz, DMSO-d6): impurity PEG at 3.5 ppm that could not be removed by purification

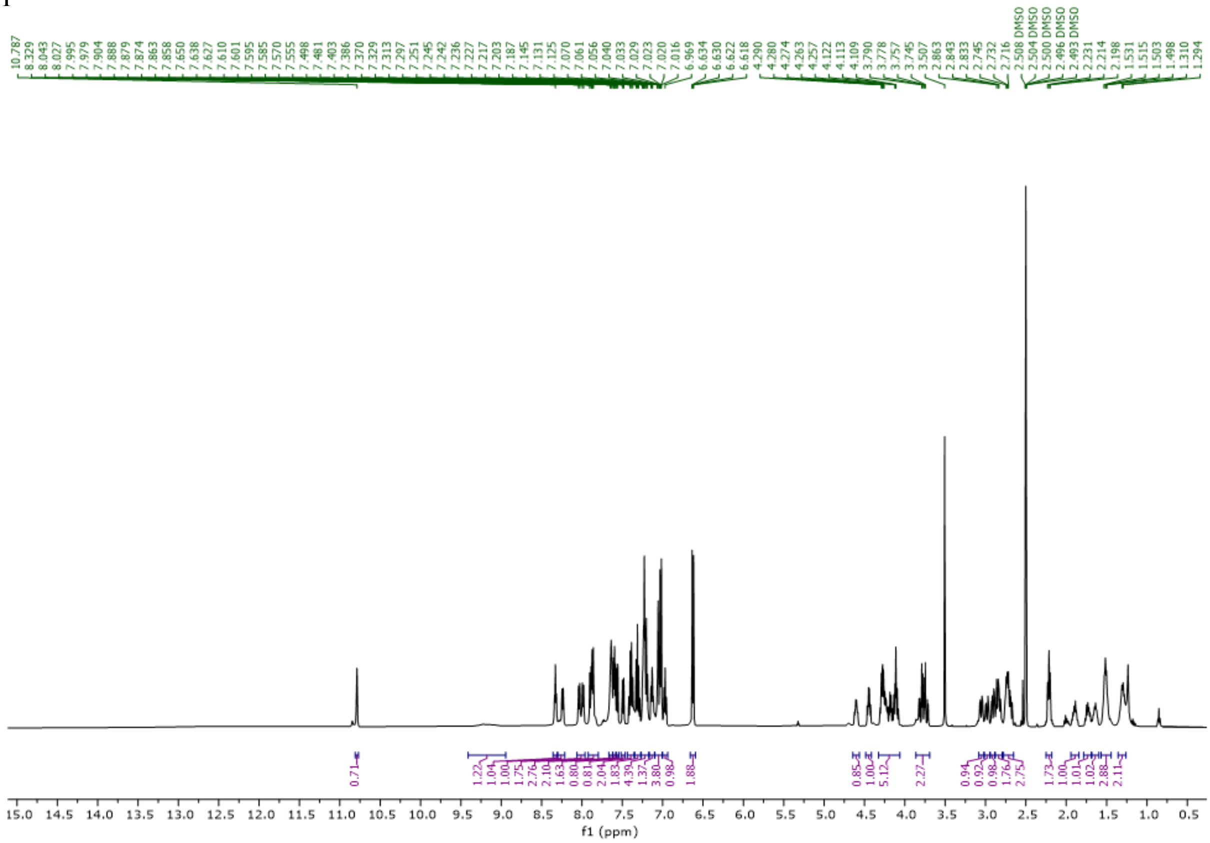

^{13}C-NMR (126 MHz, DMSO-d6)

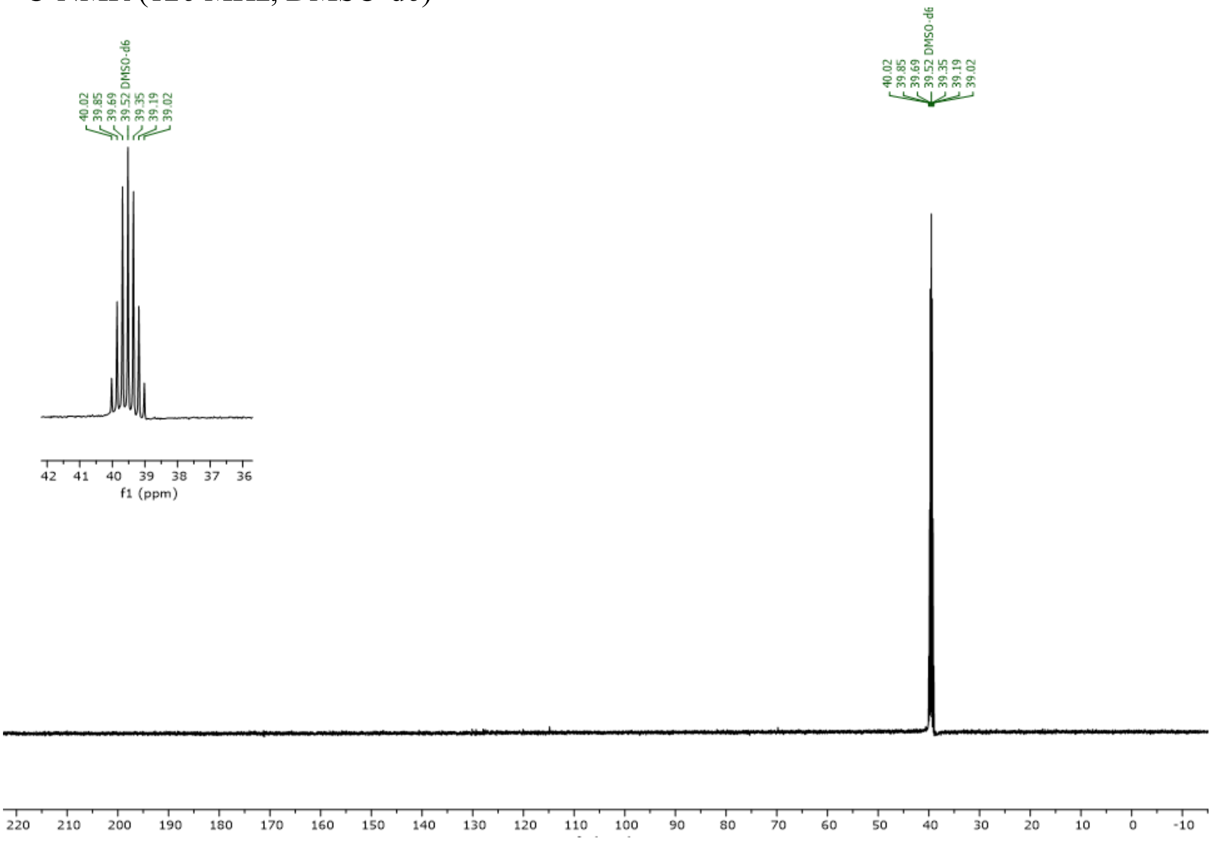

HSQC (500 MHz, DMSO-d6)

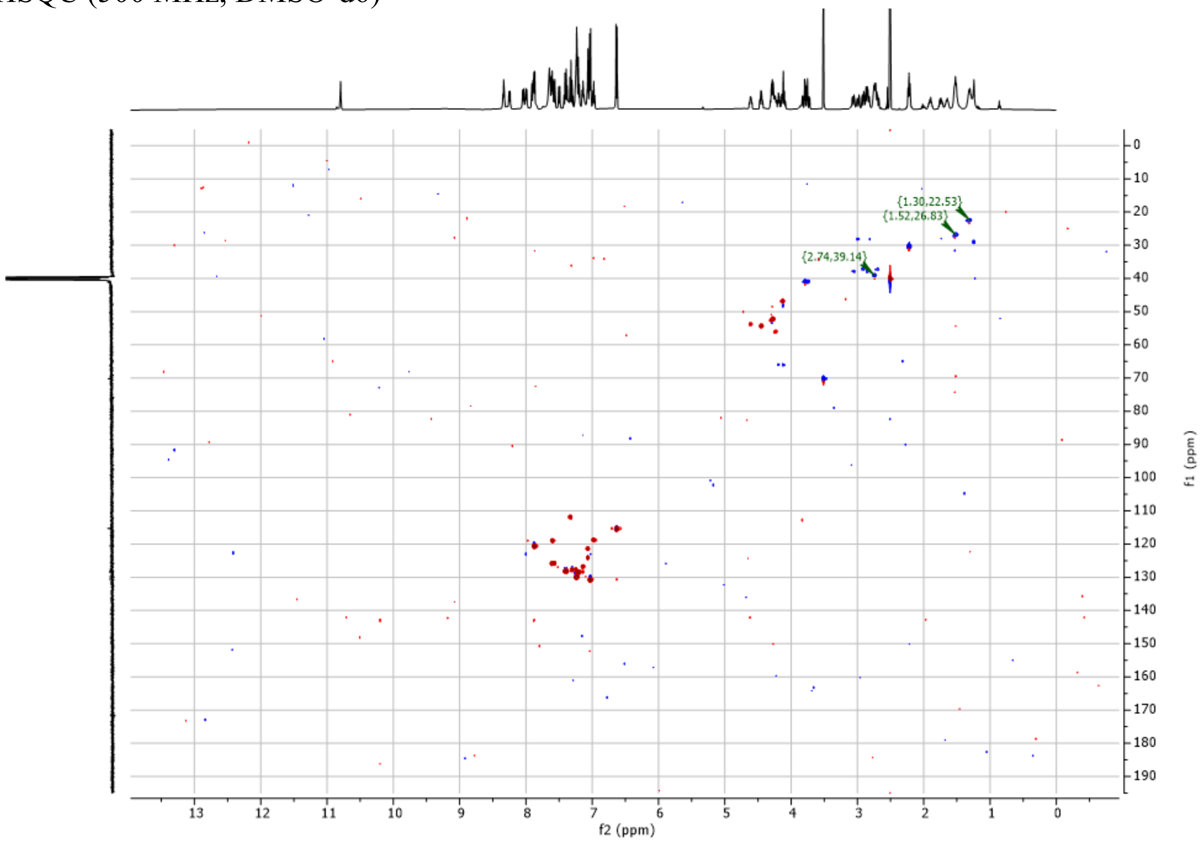

10.2.4 Hexapeptide 3C

[^{12}C]3C

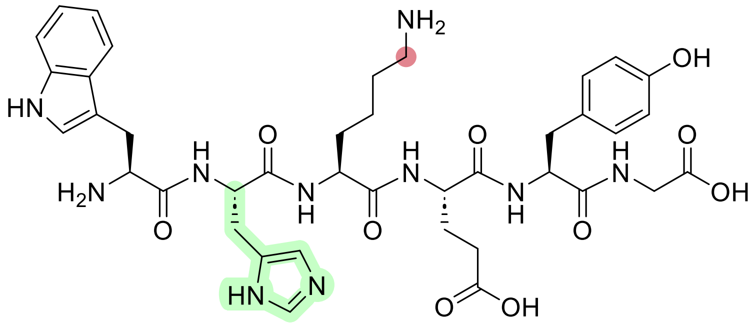

[^{12}C]3C

Chemical Formula: $C_{39}H_{50}N_{10}O_{10}$

Exact Mass: 818.3711

LC-MS of crude: ca. 75% UV-purity (3x product peak, respectively 64%, 5% and 6%); bottom-left: TIC

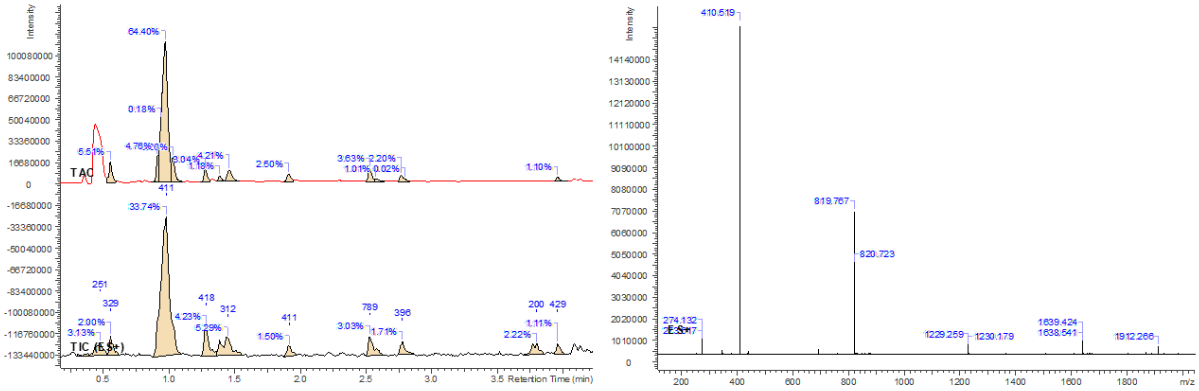

LC-MS of purified product: ca. 93% UV-purity

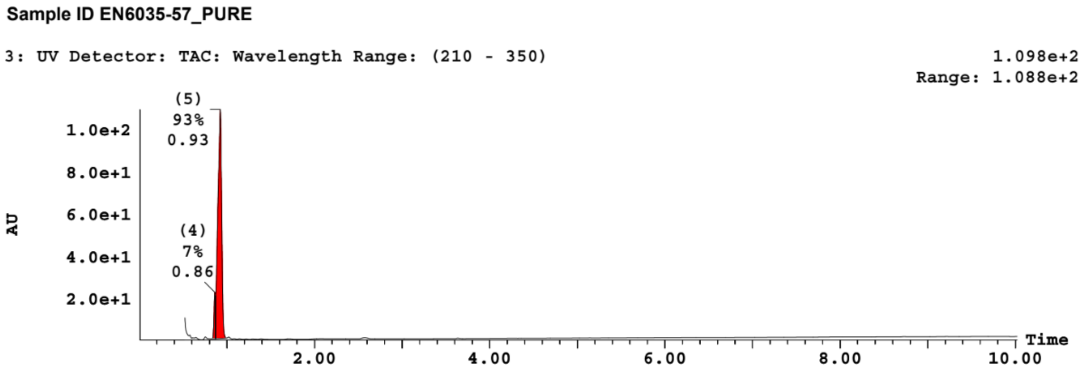

NMR of purified product: ^{1}H-NMR, ^{13}C-NMR

^{1}H-NMR (600 MHz, DMSO-d6)

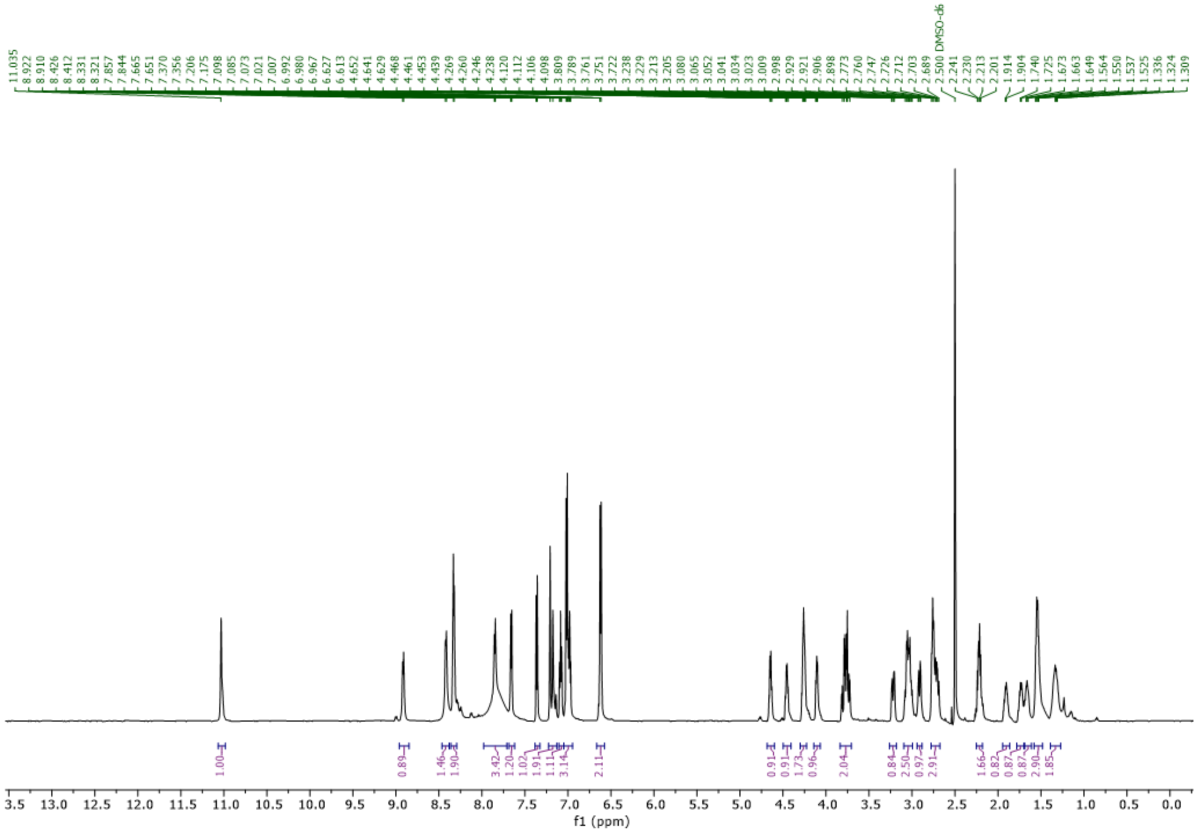

^{13}C-NMR (151 MHz, DMSO-d6)

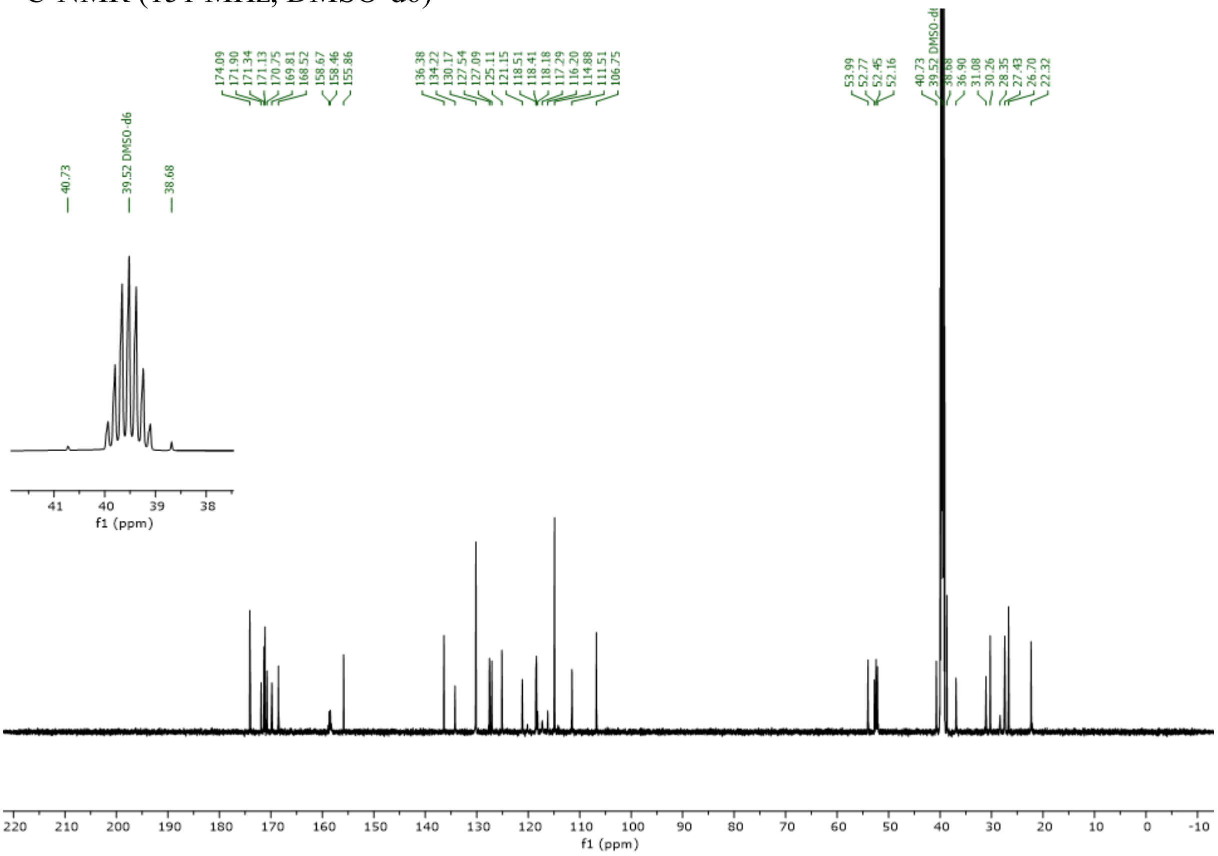

[^{13}C]3C

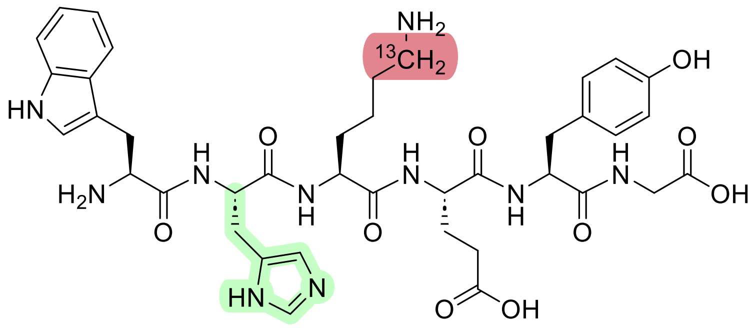

[^{13}C]3C

Chemical Formula: $C_{38}{}^{13}CH_{50}N_{10}O_{10}$

Exact Mass: 819.3745

LC-MS of crude: ca. 85% UV-purity (2-3x product peak, respectively 75%, 8% and 2%)

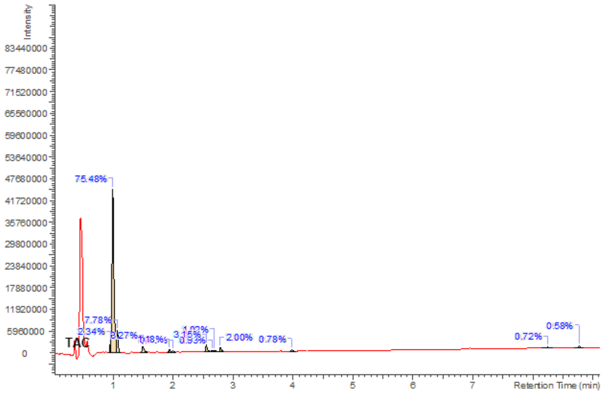

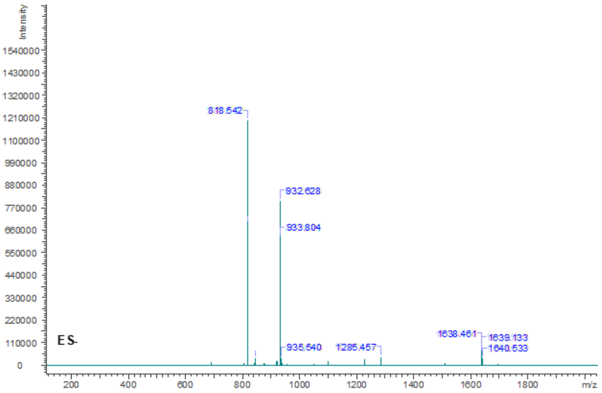

LC-MS of purified product: ca. >99% UV-purity

Sample ID EN6035-59_PURE

3: UV Detector: TAC: Wavelength Range: (210 - 350)

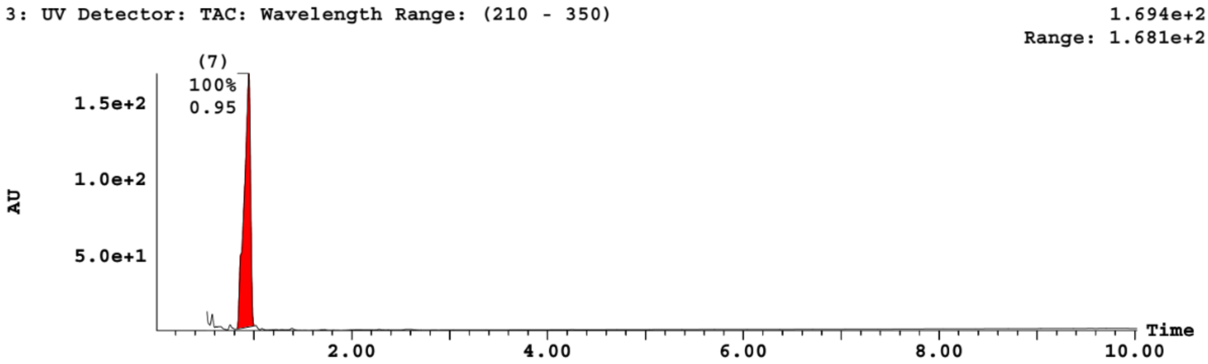

Isotope distribution comparison: Predicted non-labeled vs labeled peptide, ca. 0.99 ^{13}C/molecule incorporation

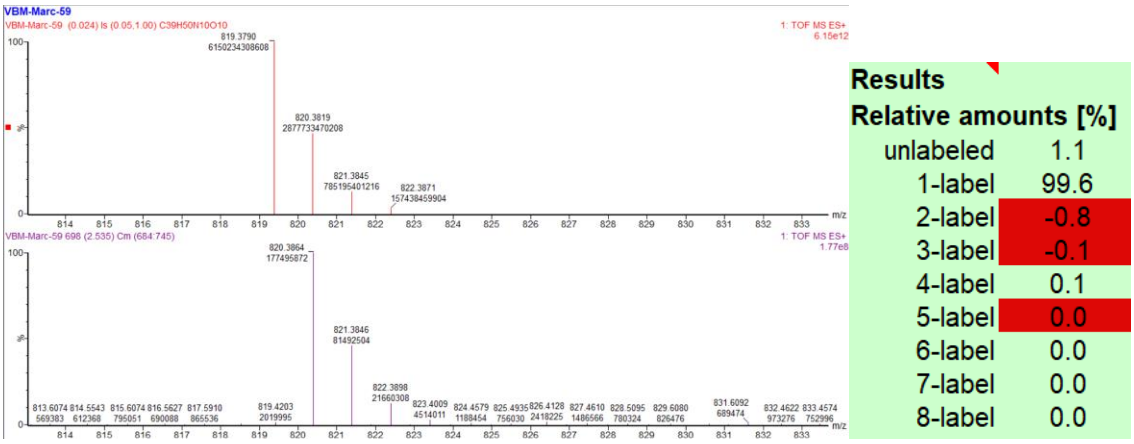

Results	
Relative amounts [%]	
unlabeled	1.1
1-label	99.6
2-label	-0.8
3-label	-0.1
4-label	0.1
5-label	0.0
6-label	0.0
7-label	0.0
8-label	0.0

^{1}H-NMR (600 MHz, DMSO-d6)

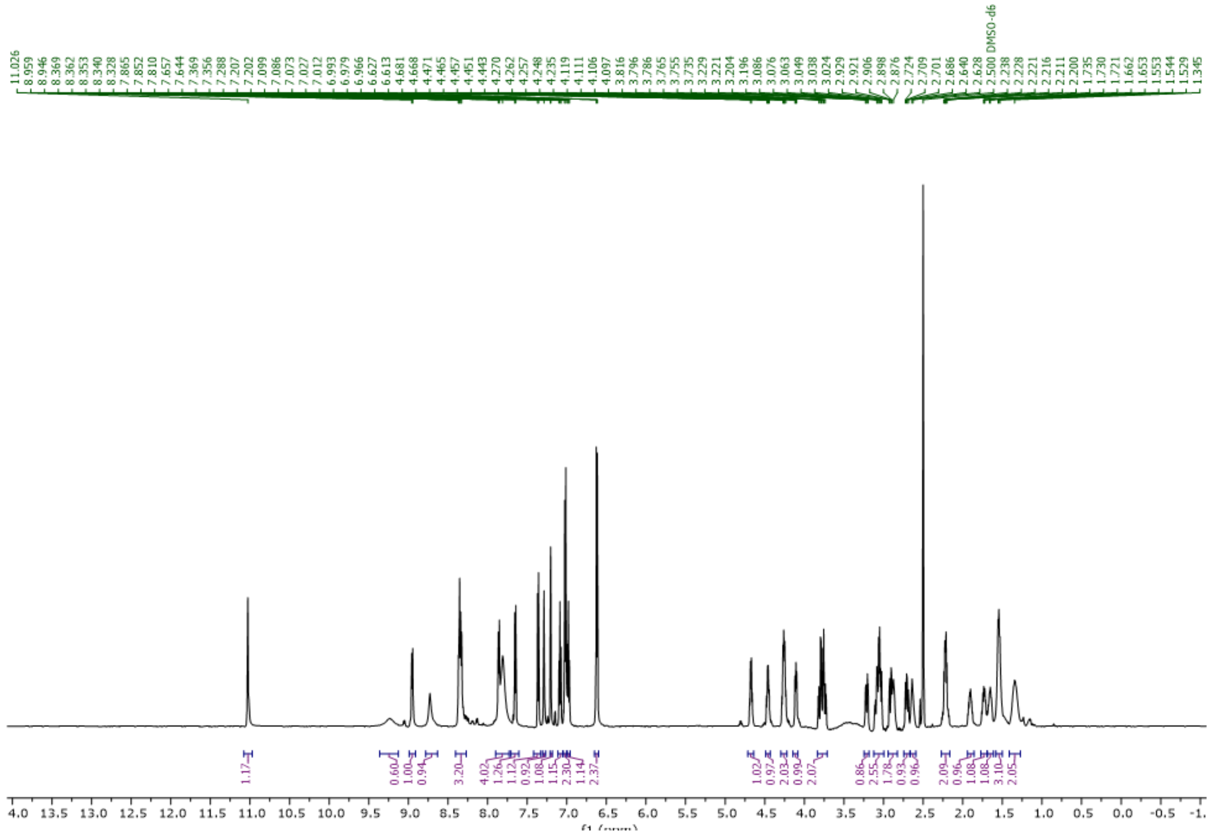

^{13}C-NMR (151 MHz, DMSO-d6): ^{13}C-label at 38.68 ppm.

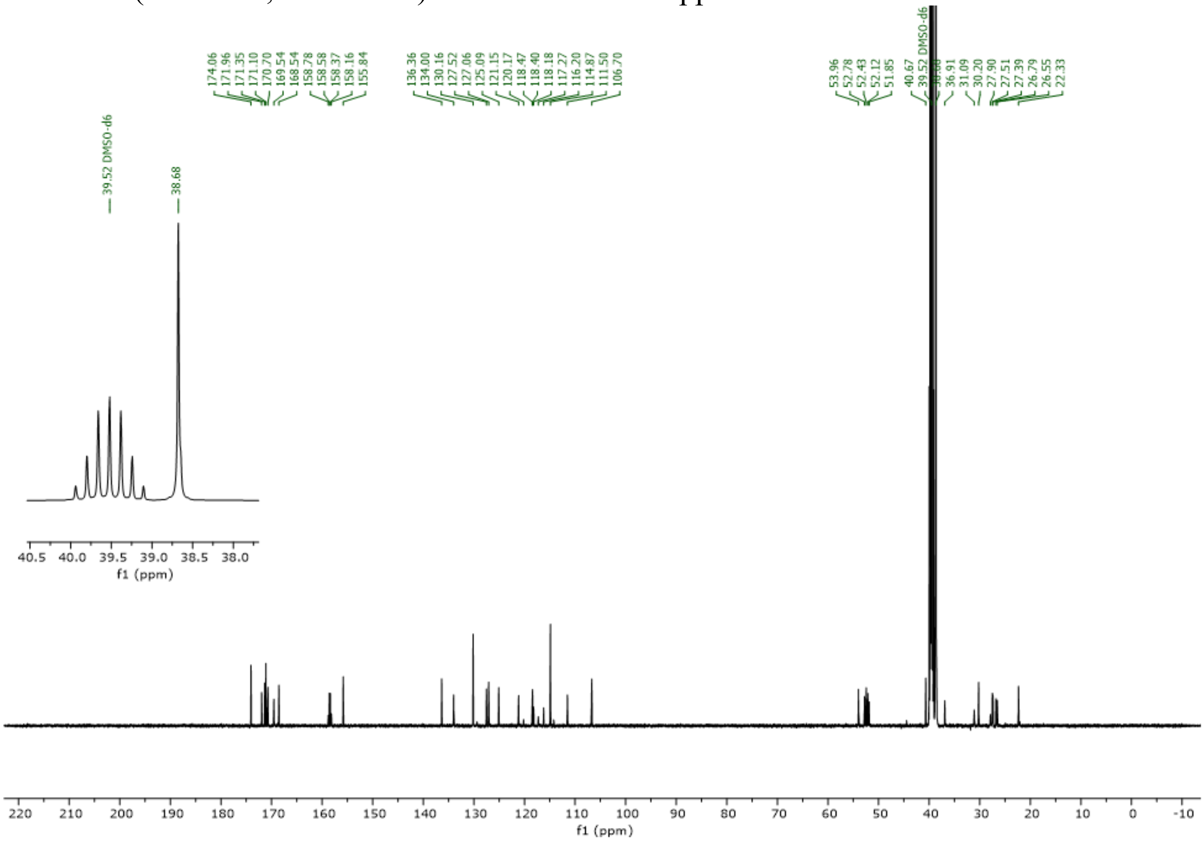

10.2.5 Hexapeptide 4C

[^{12}C]4C

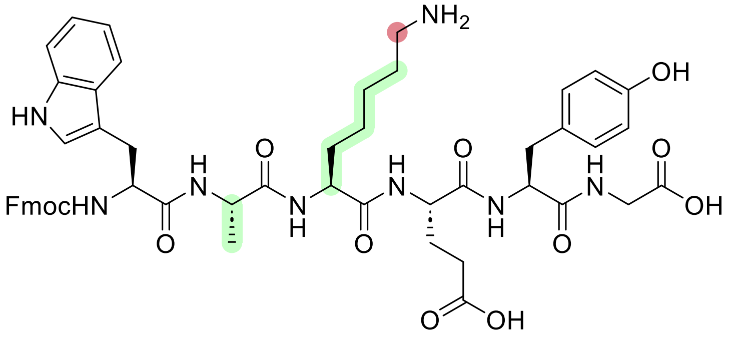

[^{12}C]4C
Chemical Formula: $C_{52}H_{60}N_8O_{12}$
Exact Mass: 988.4331

LC-MS of crude: ca. 92% UV-purity

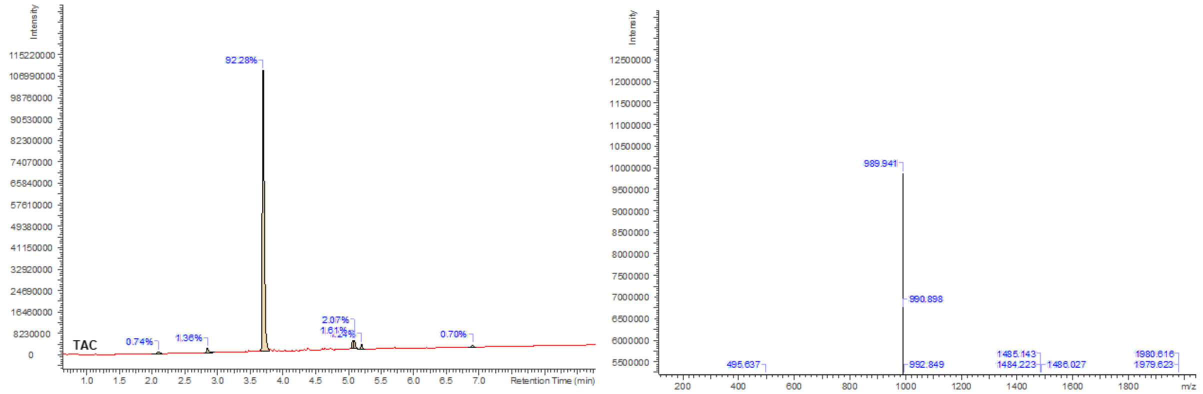

LC-MS of purified product: 90% purity

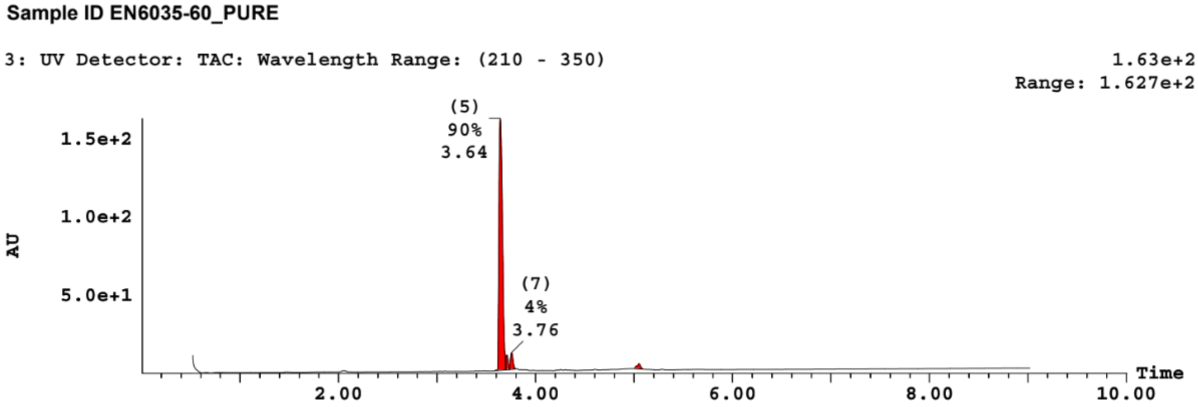

NMR of purified product: ^{1}H-NMR, ^{13}C-NMR

^{1}H-NMR (600 MHz, DMSO-d6), contains PEG signal at 3.5 ppm that could not be removed by purification

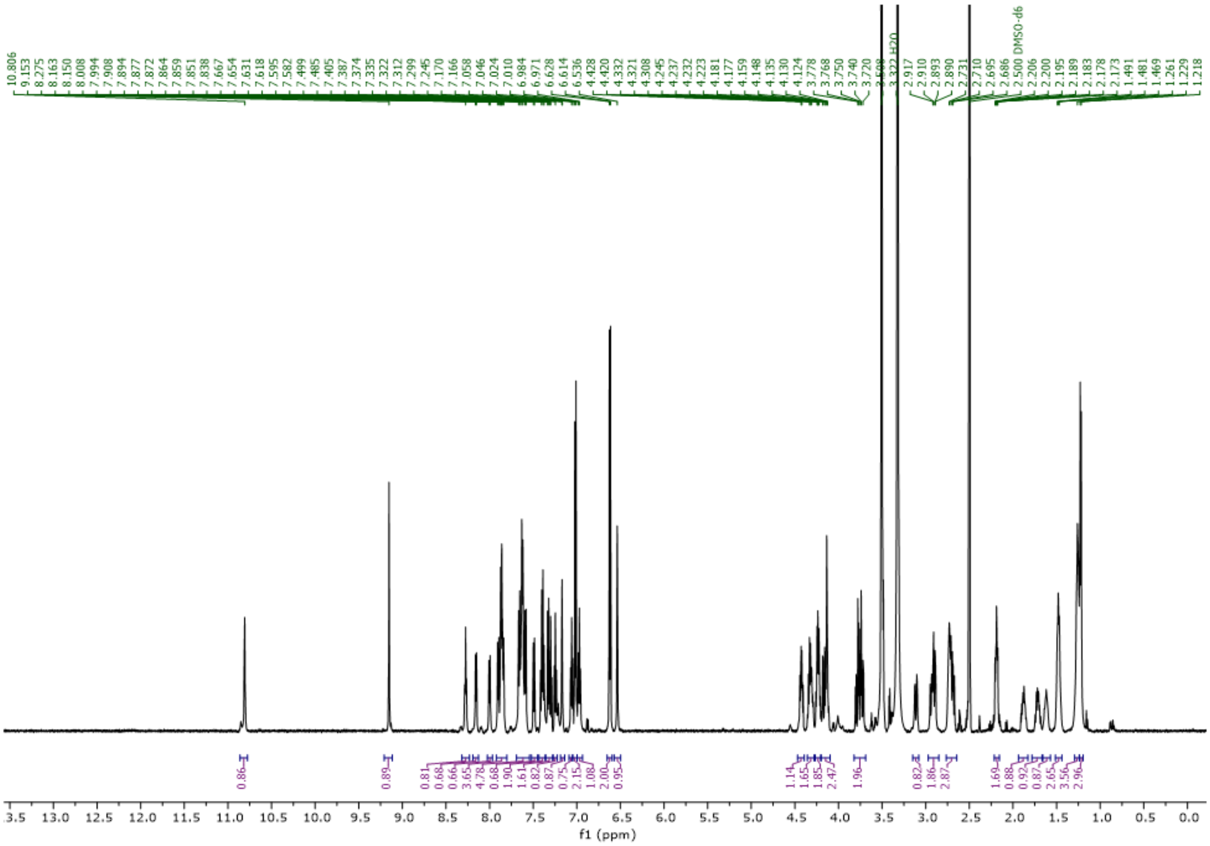

^{13}C-NMR (151 MHz, DMSO-d6), PEG signal at 69.77 ppm

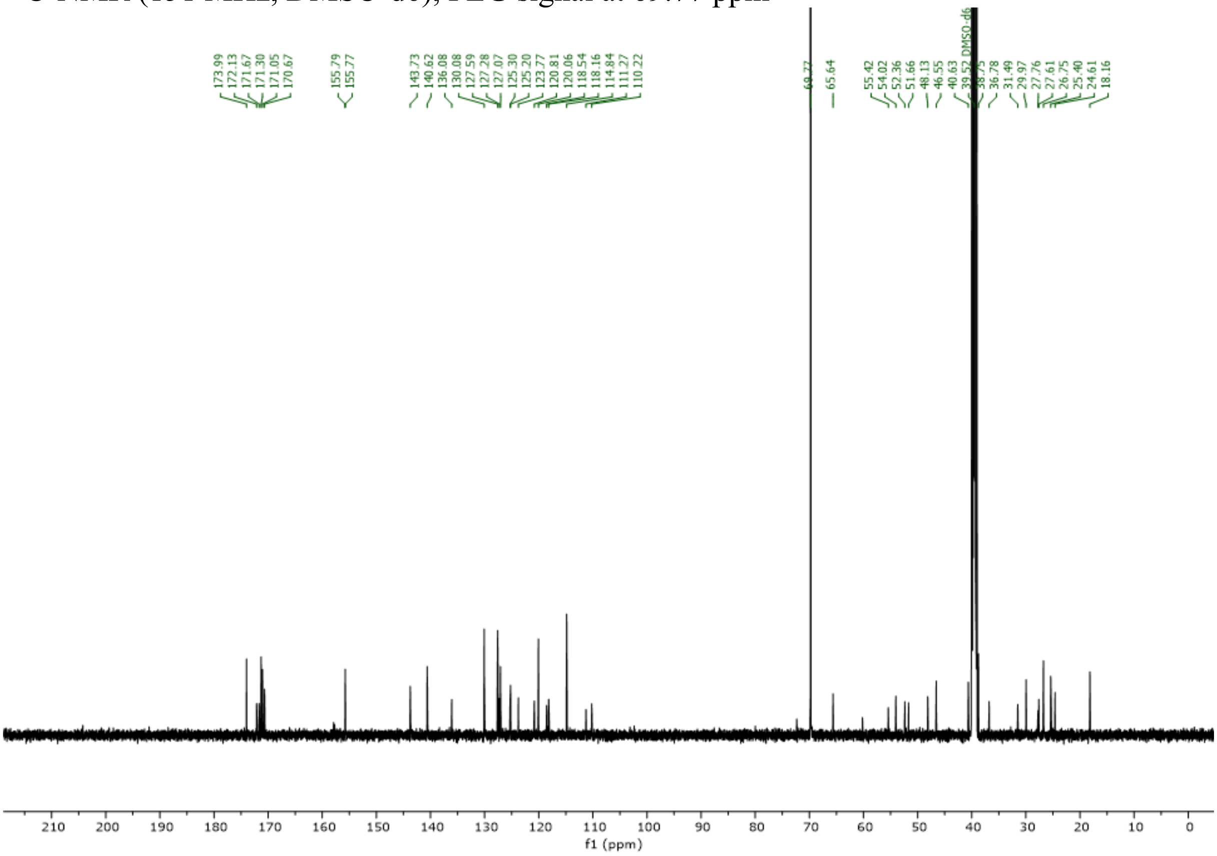

10.2.6 Hexapeptide 6C

[^{12}C]6C

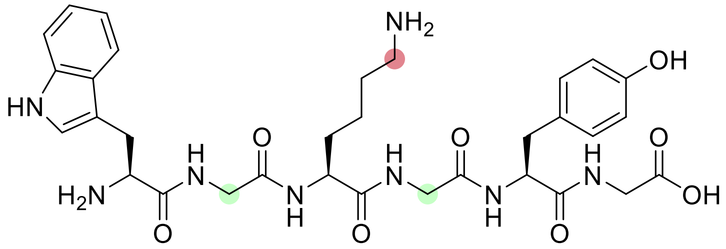

[^{12}C]6C

Chemical Formula: $C_{32}H_{42}N_8O_8$

Exact Mass: 666.3126

LC-MS of crude (spectra below LC-MS is the TIC): ca. 55% UV-purity (2x product peak, respectively 37% and 18%; and low-resolution MS indicates ca. 6-9% alcohol);
The double product signal might be an artifact, or otherwise be a poor linear:branched ratio.

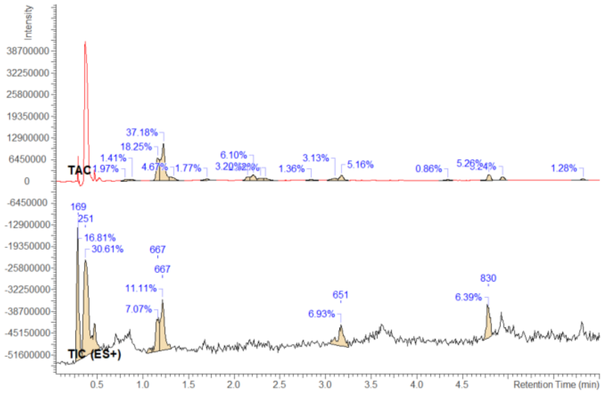

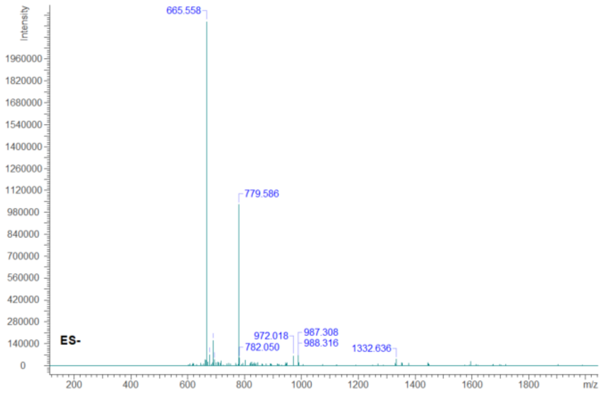

LC-MS of purified product: ca. 92% purity (2x product peak, respectively 83% and 9%)

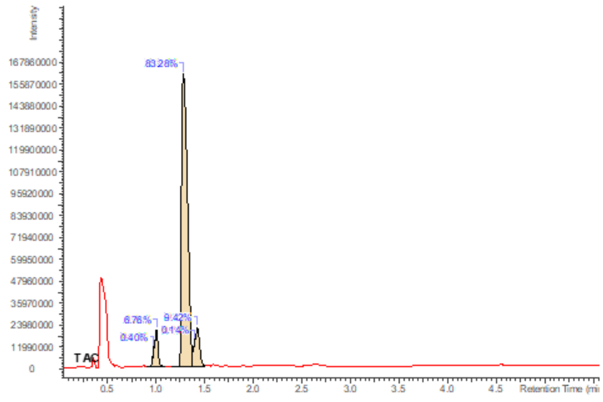

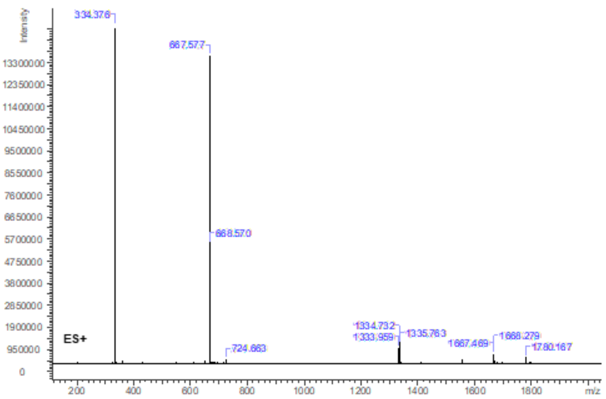

NMR of purified product: ^{1}H-NMR, ^{13}C-NMR

^{1}H-NMR (600 MHz, DMSO-d6)

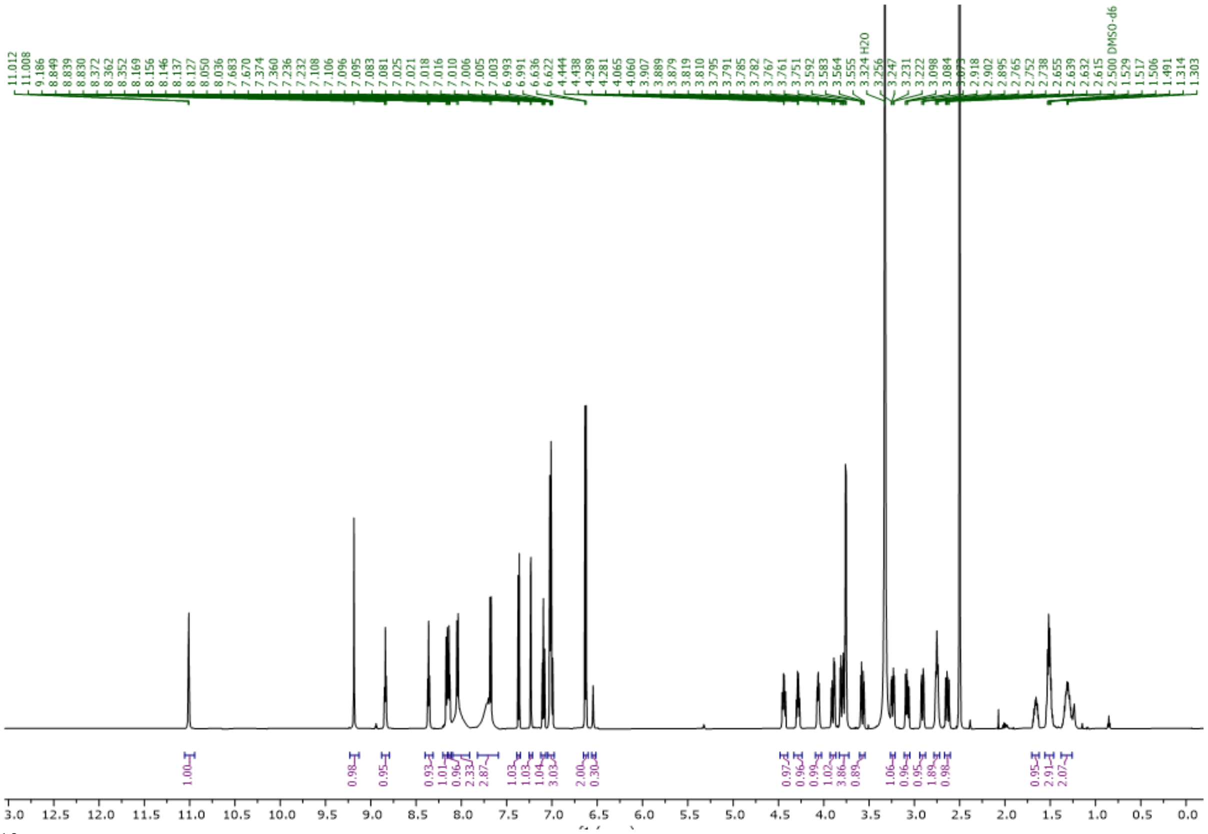

^{13}C-NMR (151 MHz, DMSO-d6)

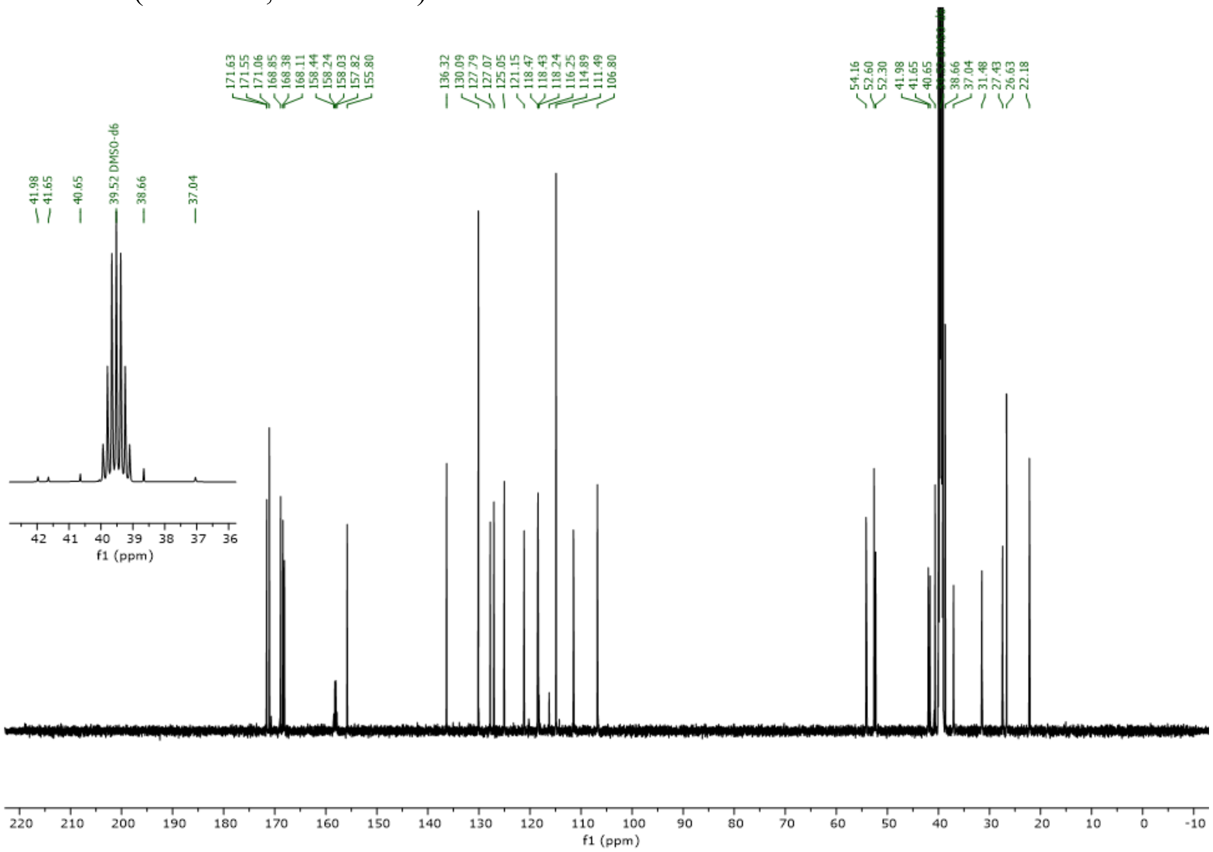

[^{13}C]6C

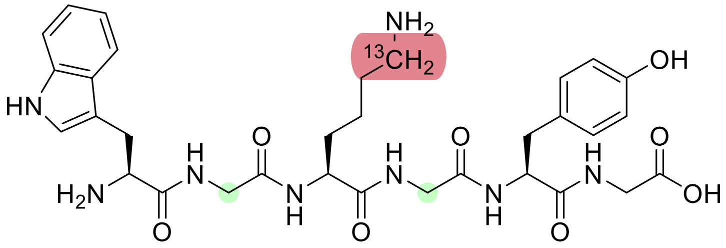

[^{13}C]6C

Chemical Formula: $C_{31}{}^{13}CH_{42}N_8O_8$

Exact Mass: 667.3159

LC-MS of crude: ca. 48% UV-purity (2.3x product peak, respectively 39%, 7% and 2%); low-resolution MS indicates ca. 11% of alcohol present in addition at 2.3 min

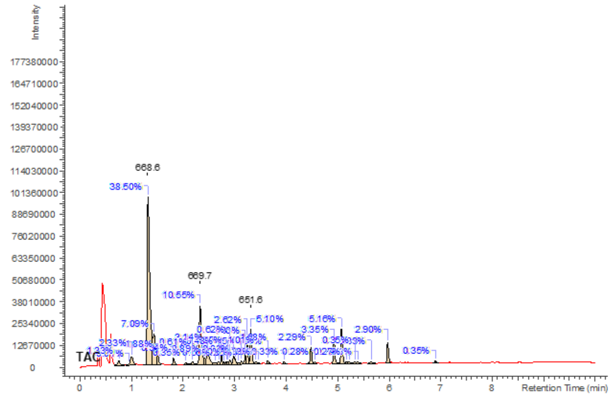

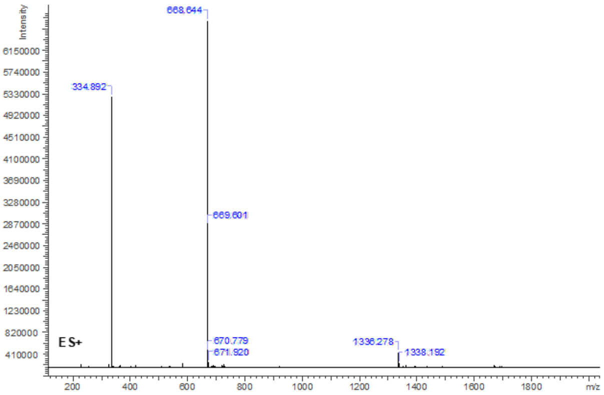

LC-MS of purified product: 85% (2x product peak, respectively 76% and 9%)

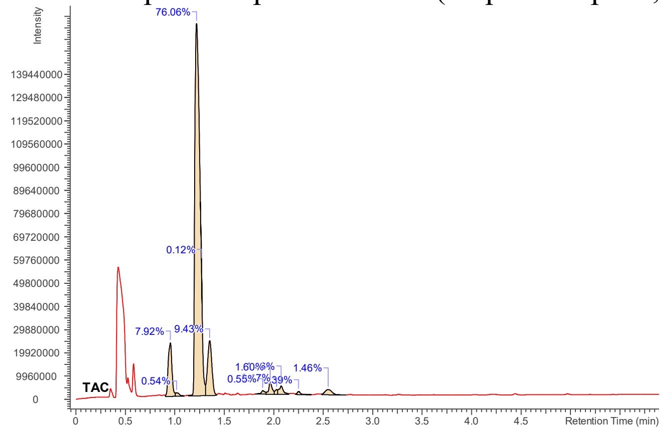

Isotope distribution comparison: Predicted non-labeled vs labeled peptide, ca. 0.99 ^{13}C/molecule incorporation

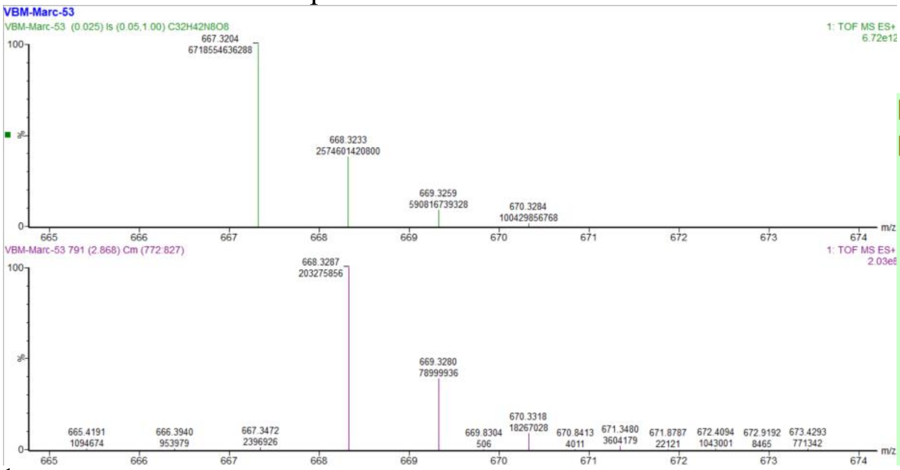

Results	
Relative amounts [%]	
unlabeled	1.2
1-label	98.1
2-label	0.6
3-label	0.0
4-label	0.2
5-label	-0.1
6-label	0.0
7-label	0.0
8-label	0.0

^{1}H-NMR (600 MHz, DMSO-d6)

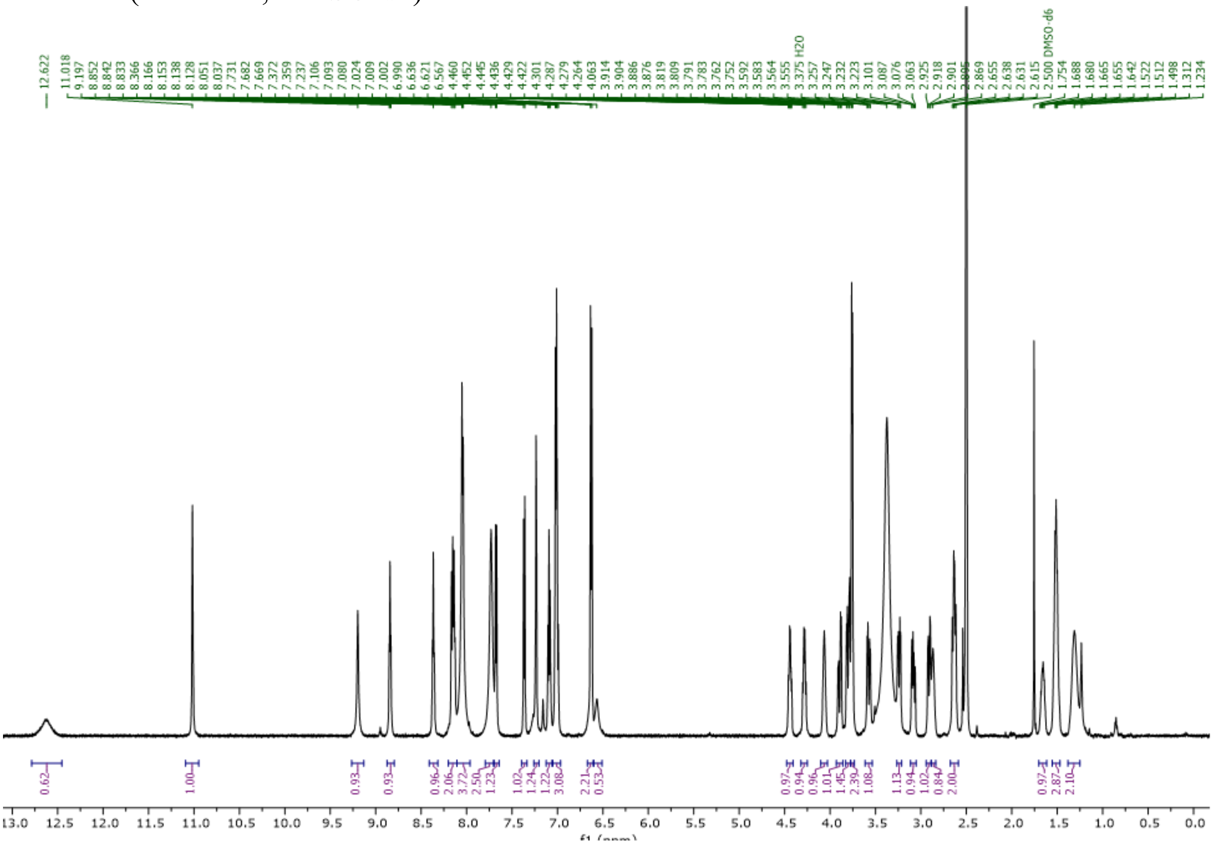

^{13}C-NMR (151 MHz, DMSO-d6): ^{13}C-label at 38.64 ppm.

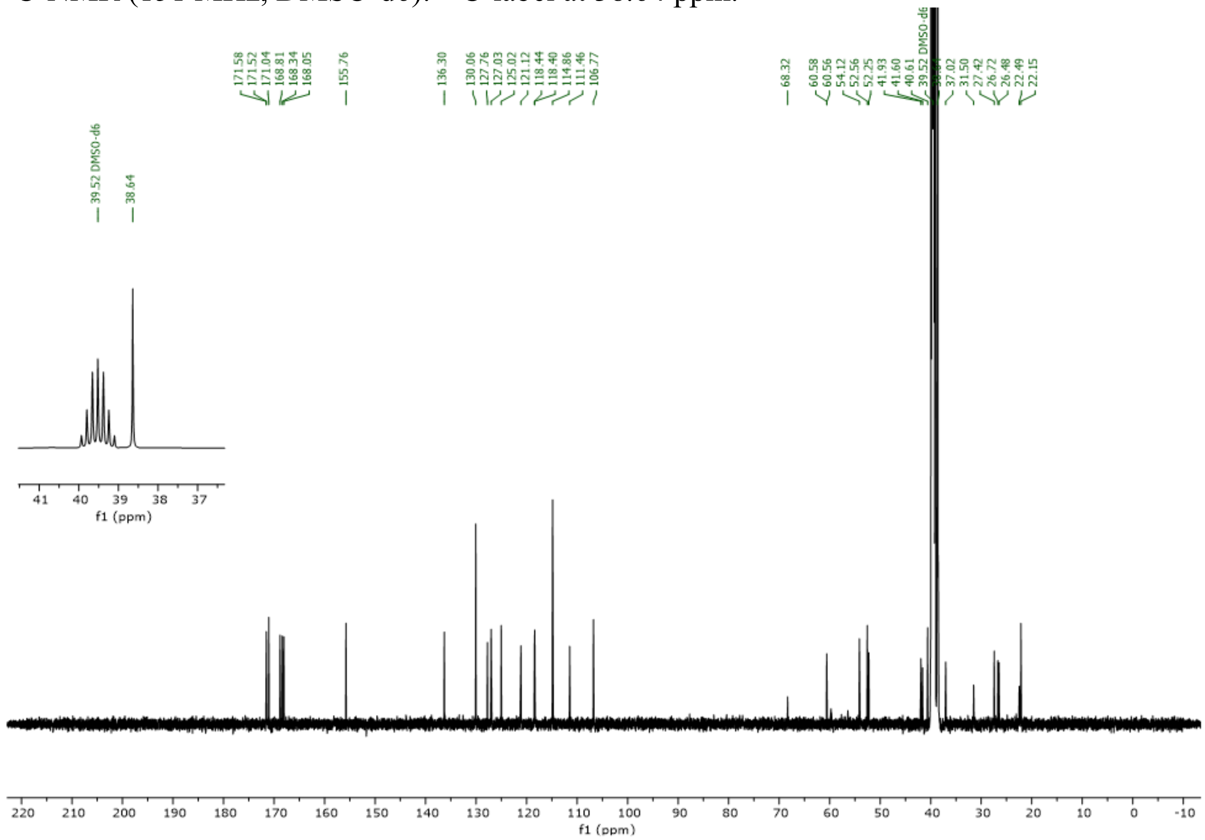

10.2.7 Hexapeptide 7C

[^{12}C]7C

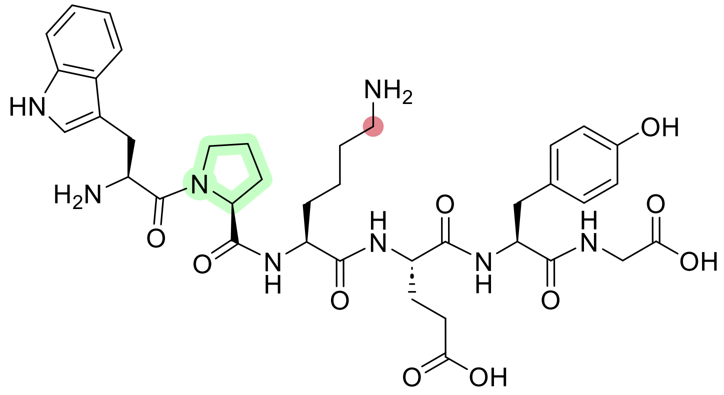

[^{12}C]7C

Chemical Formula: $C_{38}H_{50}N_8O_{10}$

Exact Mass: 778.3650

LC-MS of crude: ca. 72% UV-purity

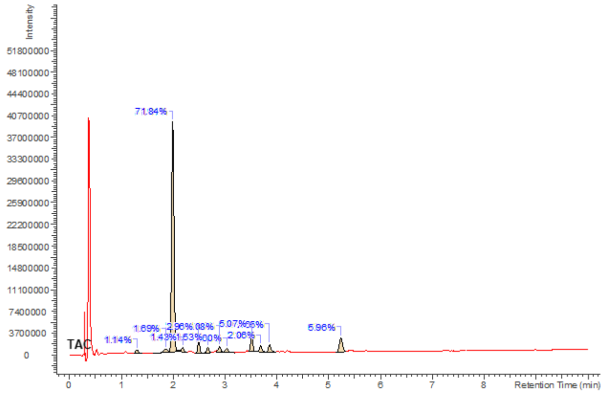

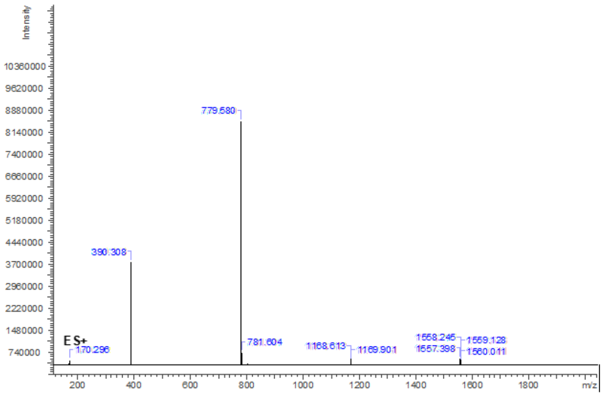

LC-MS of purified product: ca. 95% UV-purity (2-3x product peak, respectively 88%, 2% and 5%)

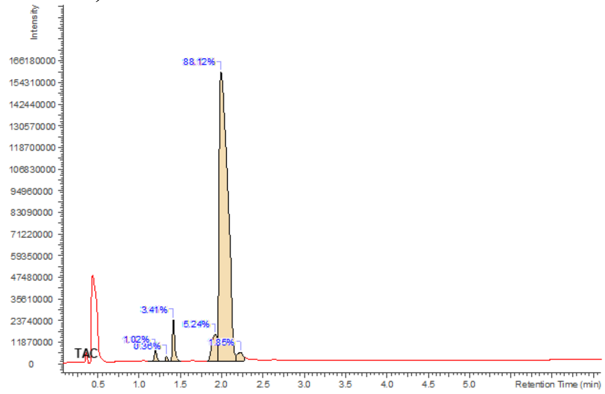

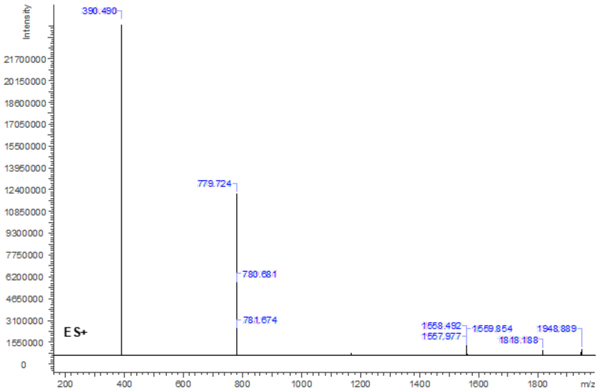

NMR of purified product: ^{1}H-NMR, ^{13}C-NMR

^{1}H-NMR (600 MHz, DMSO-d6)

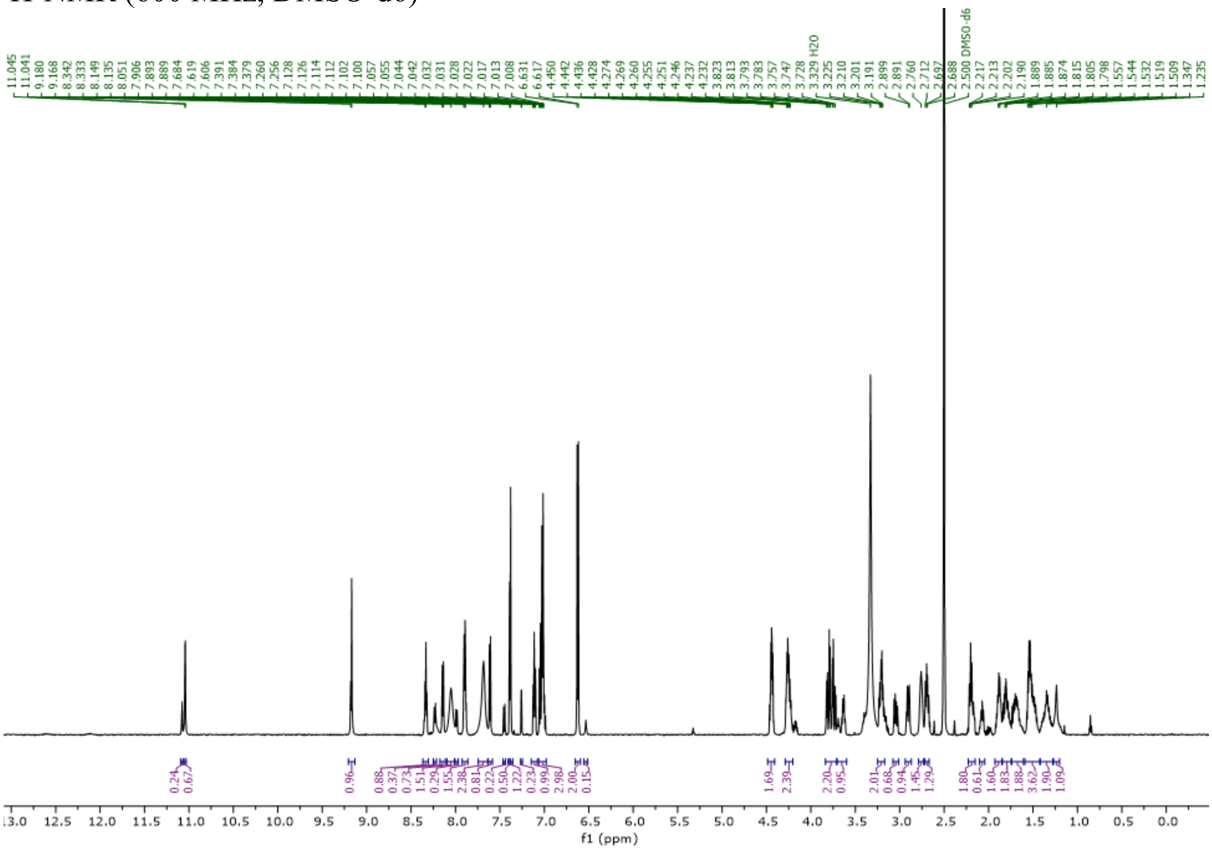

^{13}C-NMR (151 MHz, DMSO-d6)

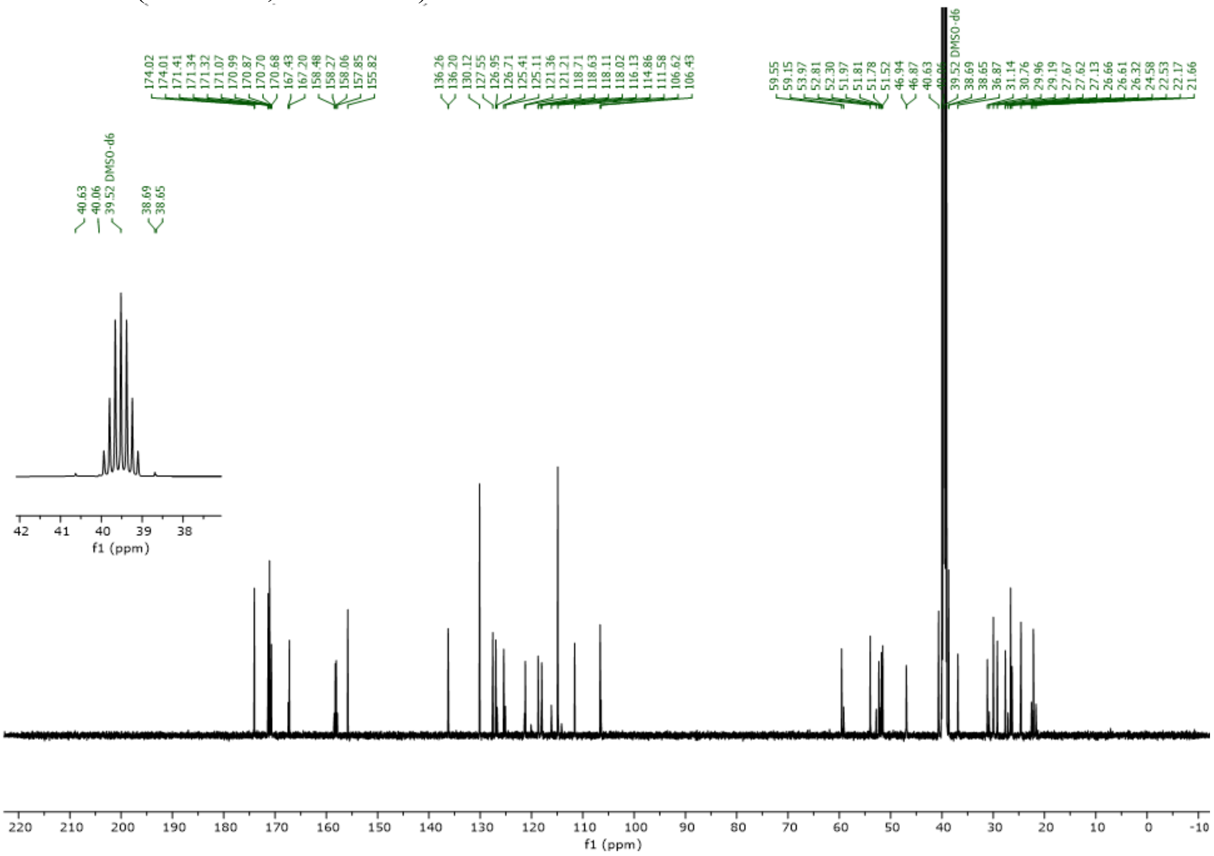

[^{13}C]7C

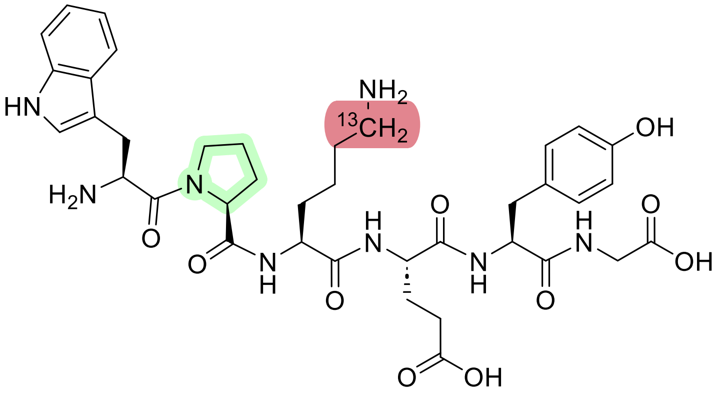

[^{13}C]7C

Chemical Formula: $C_{37}{}^{13}CH_{50}N_8O_{10}$

Exact Mass: 779.3683

LC-MS of crude: ca. 48% UV-purity (2x product peak, respectively 45% and 3%)

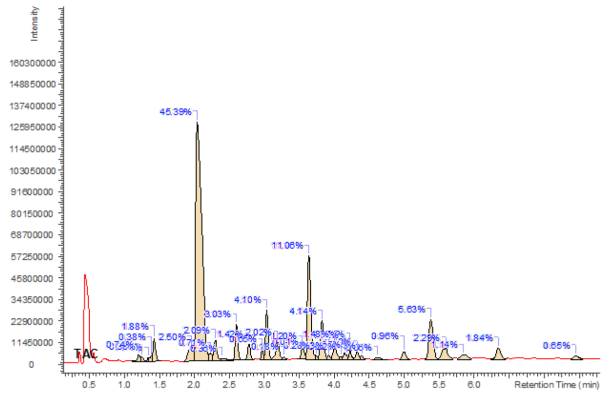

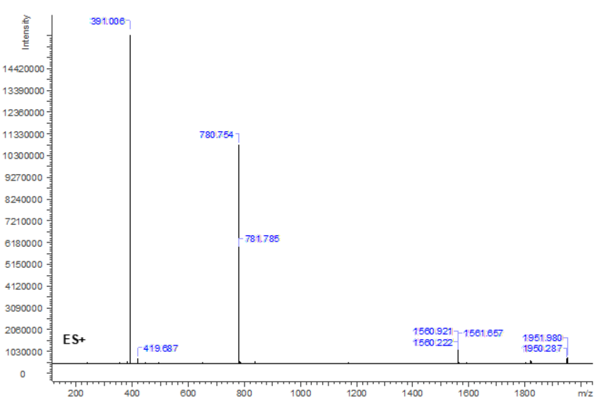

Isotope distribution comparison: Predicted non-labeled vs labeled peptide, ca. 0.99 ^{13}C/molecule% incorporation

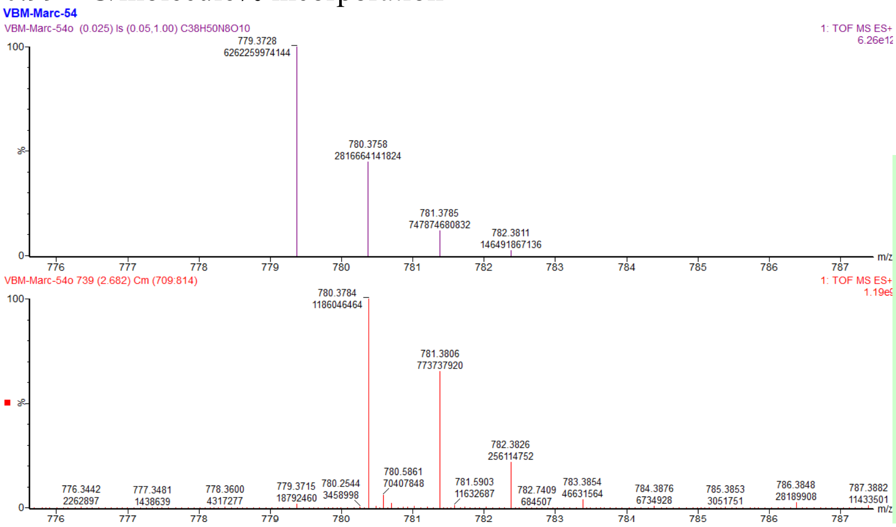

Results	
Relative amounts [%]	
unlabeled	1.3
1-label	82.2
2-label	16.9
3-label	0.4
4-label	-0.9
5-label	-0.1
6-label	0.1
7-label	0.0
8-label	0.0

10.2.8 Hexapeptide 8C

[^{12}C]8C

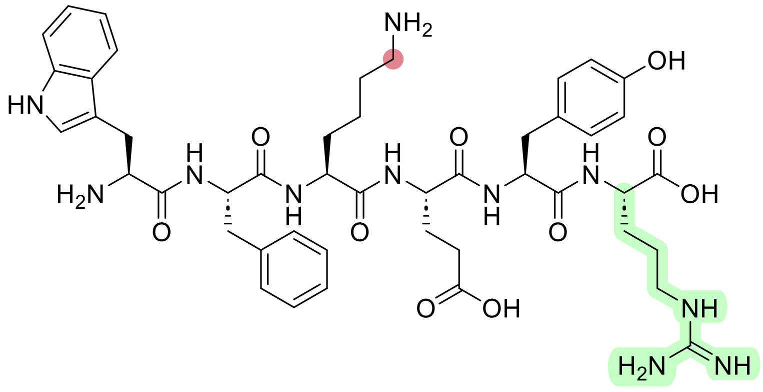

[^{12}C]8C

Chemical Formula: $C_{46}H_{61}N_{11}O_{10}$

Exact Mass: 927.4603

LC-MS of crude: ca. 72% UV-purity

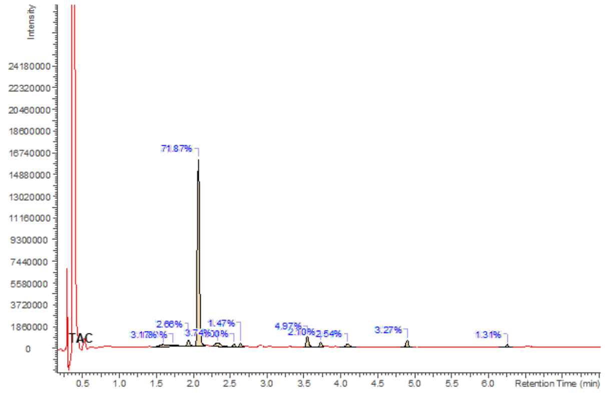

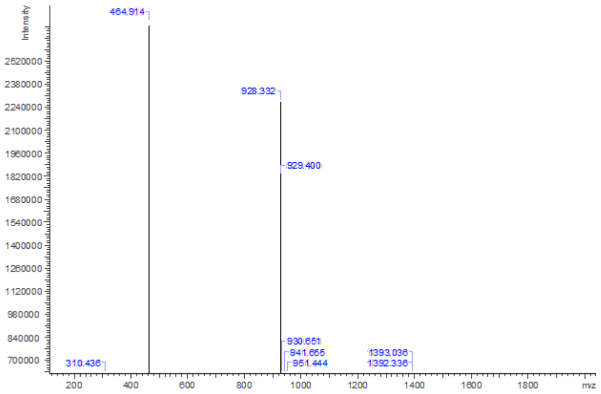

LC-MS of purified product: ca. 93% UV-purity

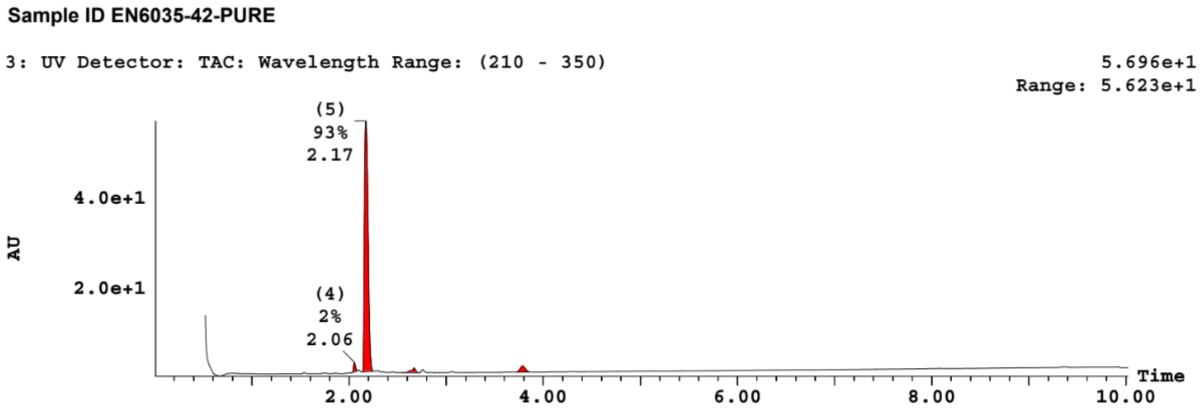

NMR of purified product: ^{1}H-NMR, ^{13}C-NMR

^{1}H-NMR (600 MHz, DMSO-d6)

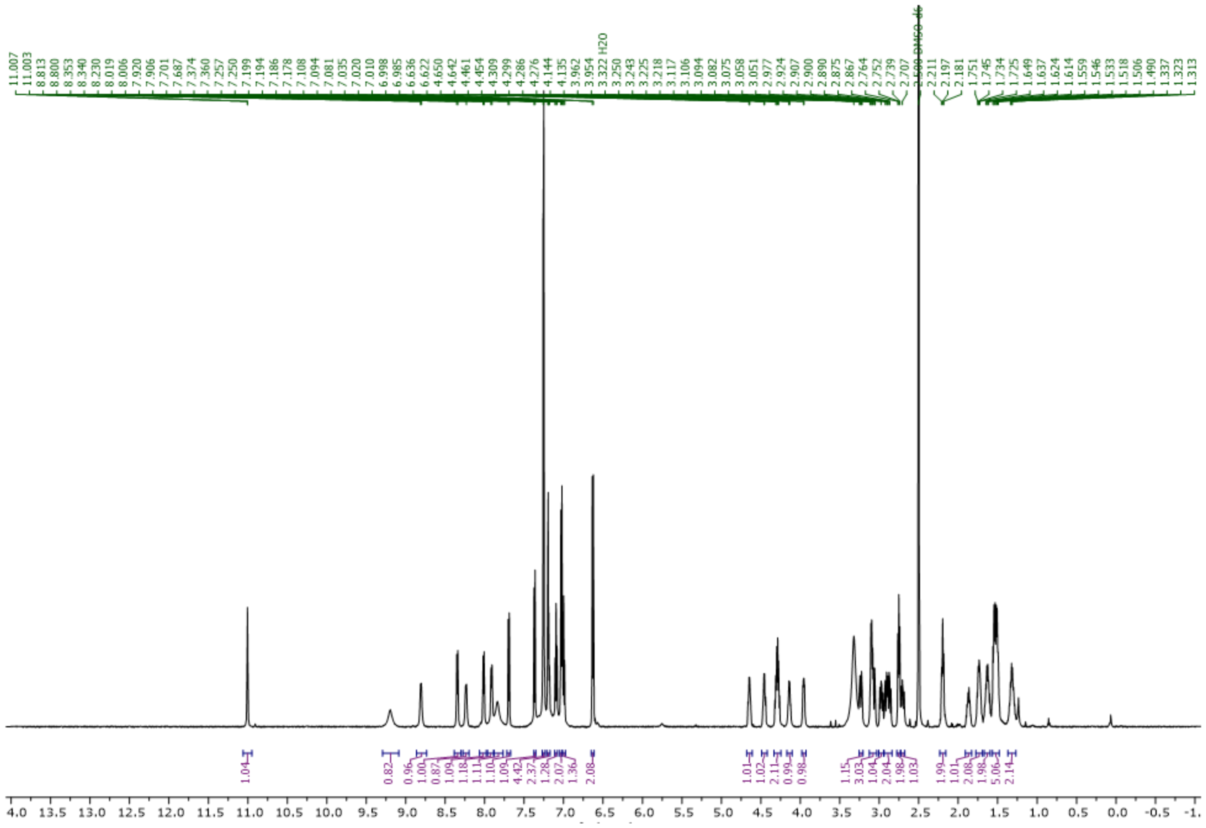

^{13}C-NMR (151 MHz, DMSO-d6)

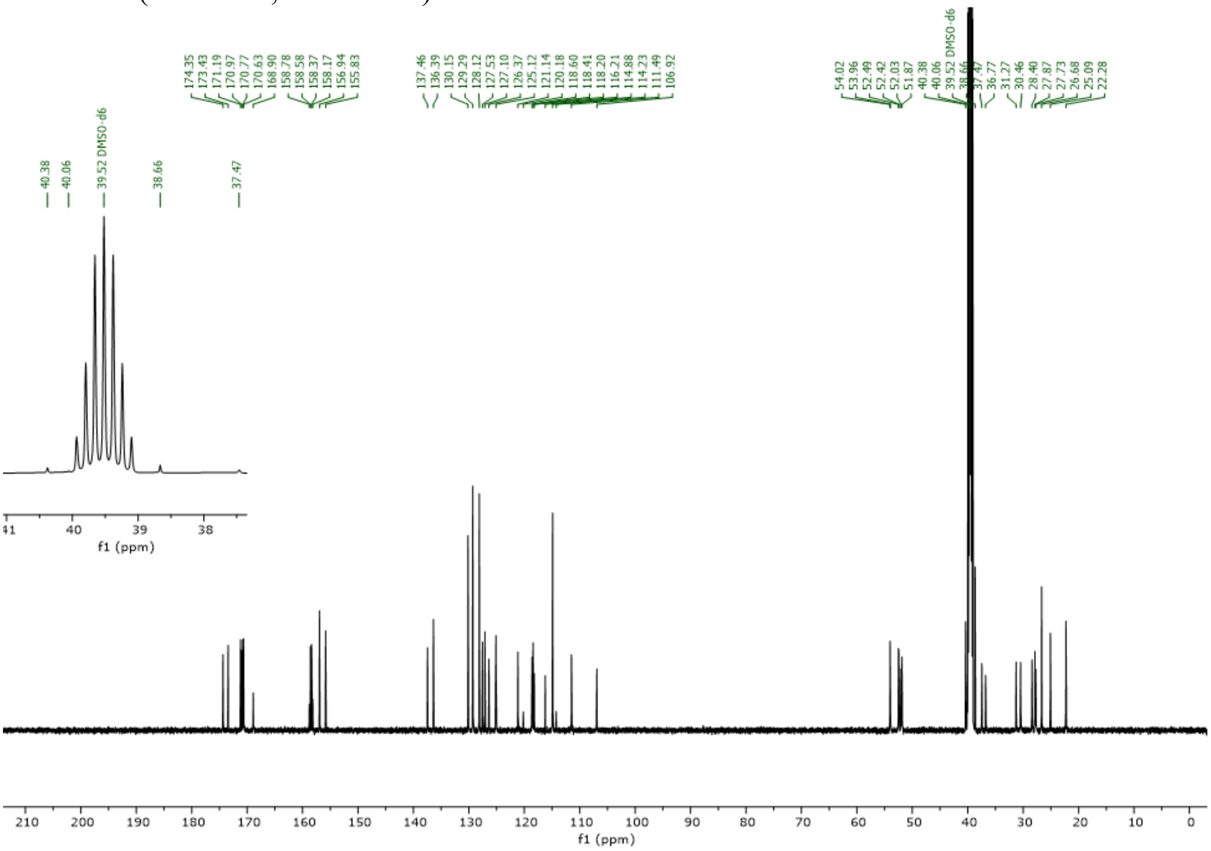

[^{13}C]8C

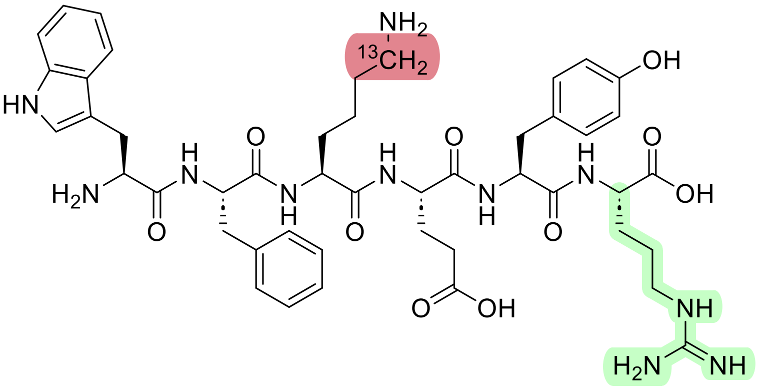

[^{13}C]8C

Chemical Formula: $C_{45}{}^{13}CH_{61}N_{11}O_{10}$

Exact Mass: 928.4636

LC-MS of crude: ca. 71% UV-purity

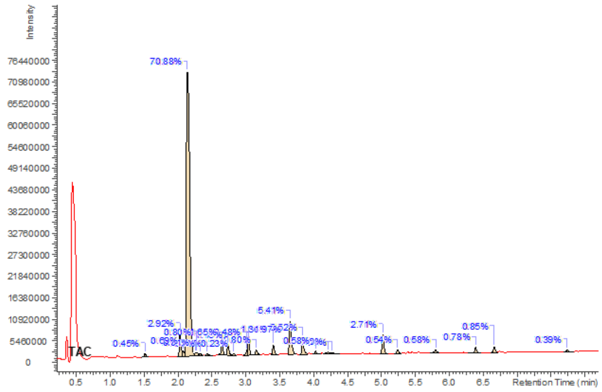

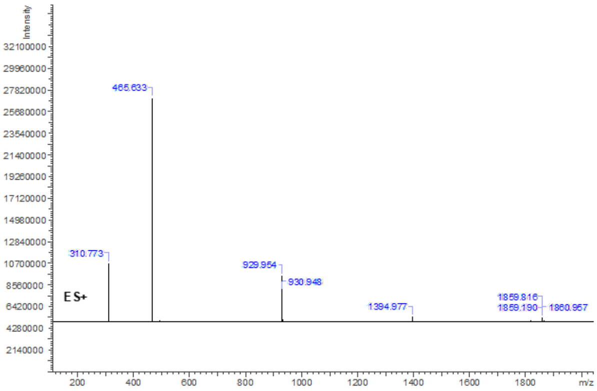

LC-MS of purified product: ca. 86% UV-purity (2x product peak, respectively 80% and 6%)

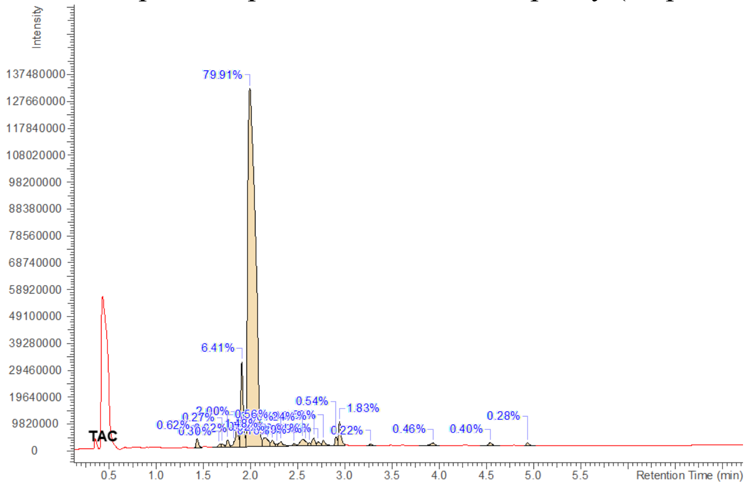

Isotope distribution comparison: Predicted non-labeled vs labeled peptide, 0.99 ^{13}C/molecule incorporation

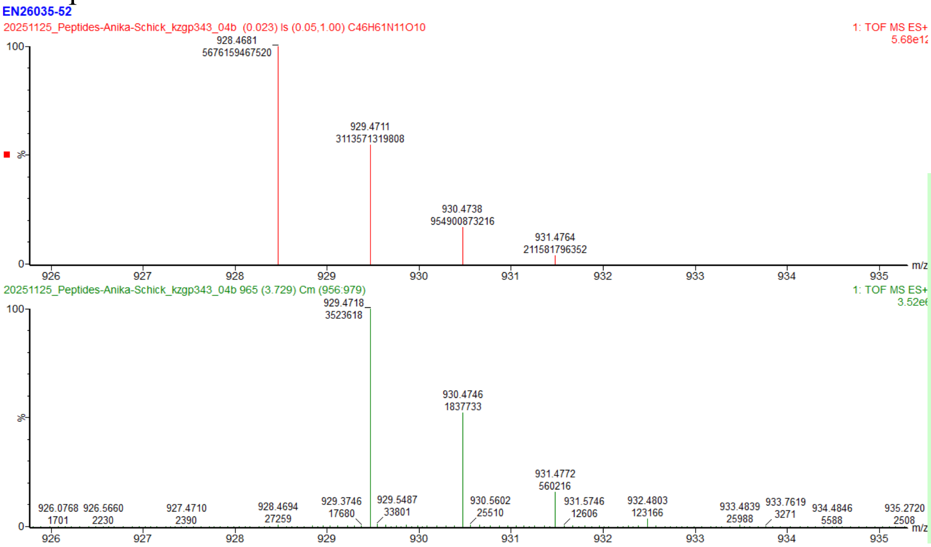

Results	
Relative amounts [%]	
unlabeled	0.8
1-label	101.4
2-label	-2.6
3-label	0.6
4-label	-0.1
5-label	0.0
6-label	0.0
7-label	0.0
8-label	0.0

^{1}H-NMR (600 MHz, DMSO-d6)

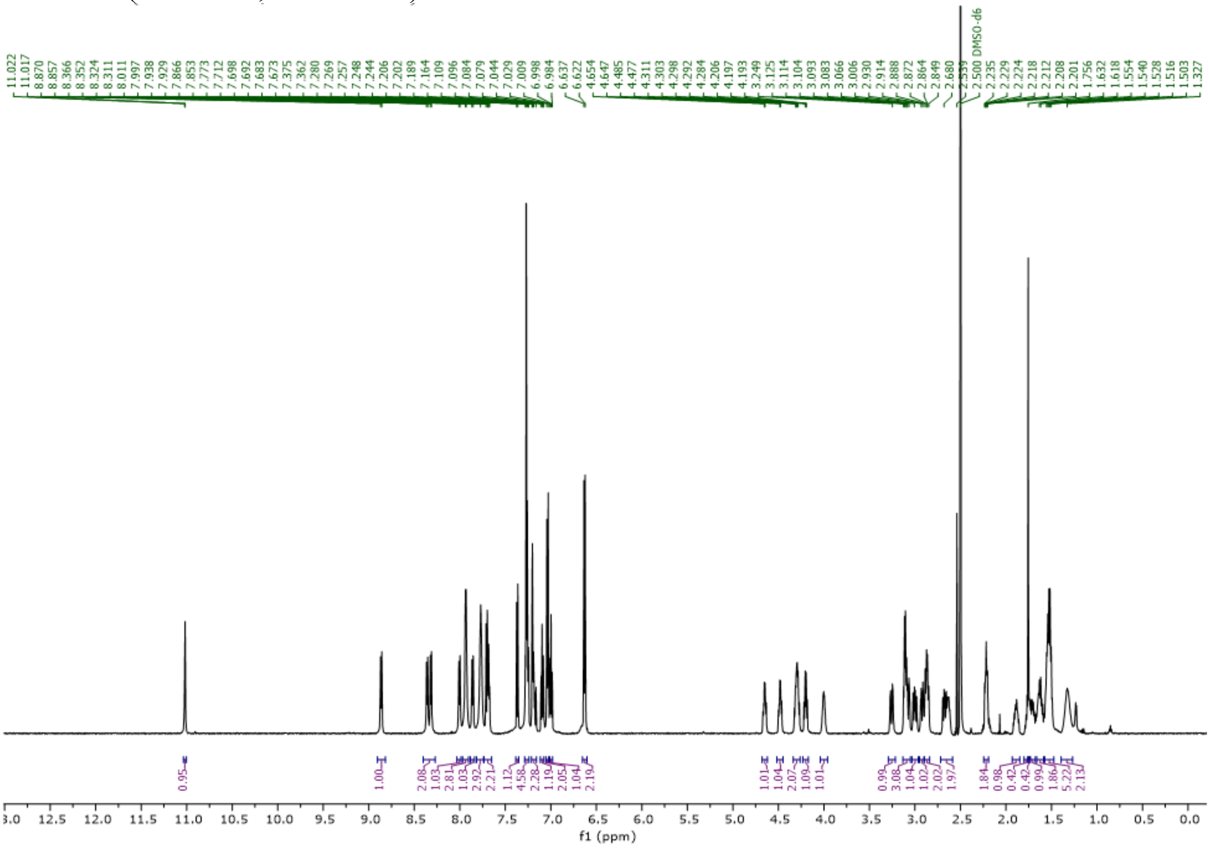

^{13}C-NMR (151 MHz, DMSO-d6): ^{13}C-label at 38.67 ppm.

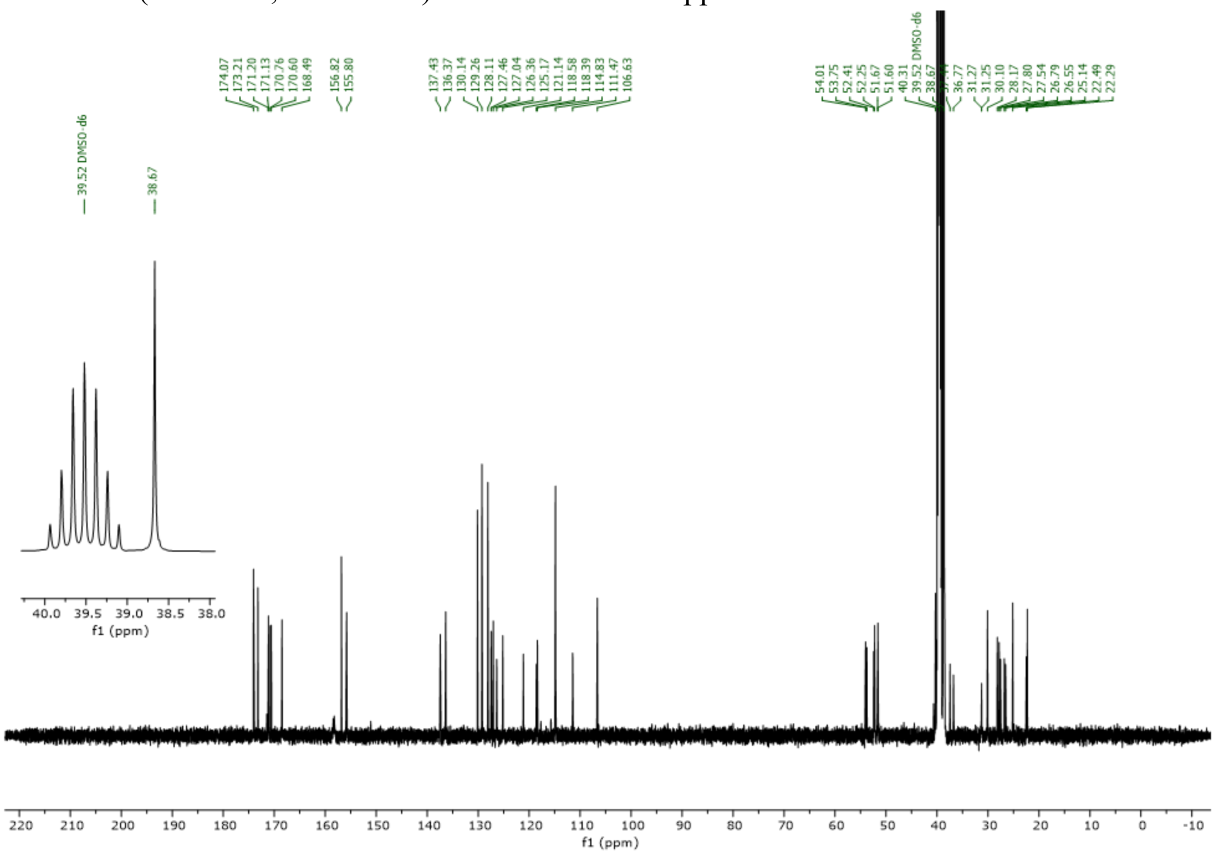

10.2.9 Hexapeptide 9C

[^{12}C]9C

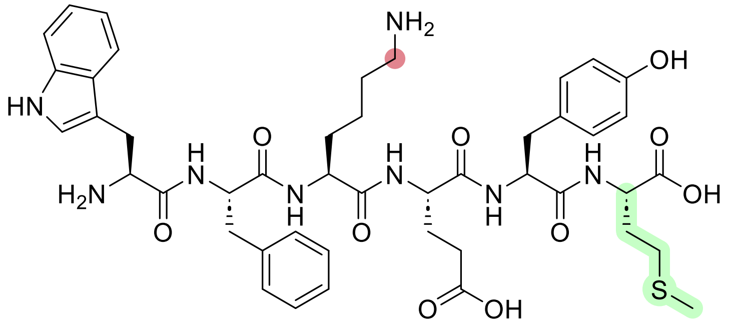

[^{12}C]9C

Chemical Formula: $C_{45}H_{58}N_8O_{10}S$

Exact Mass: 902.3997

LC-MS of crude: ca. 70% UV-purity (2-3x product peak, respectively 54%, 8% and 8%)

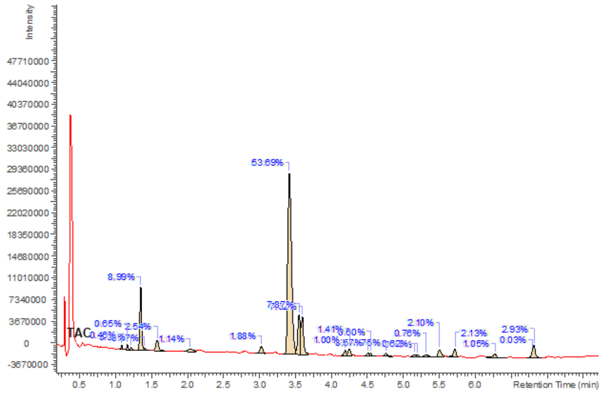

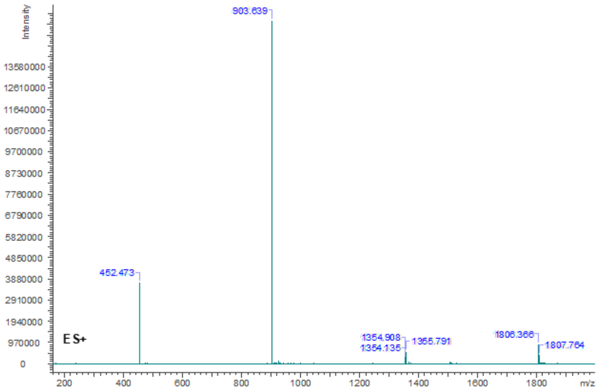

LC-MS of purified product: ca. 94% UV-purity (2x product peak, respectively 92% and 2%)

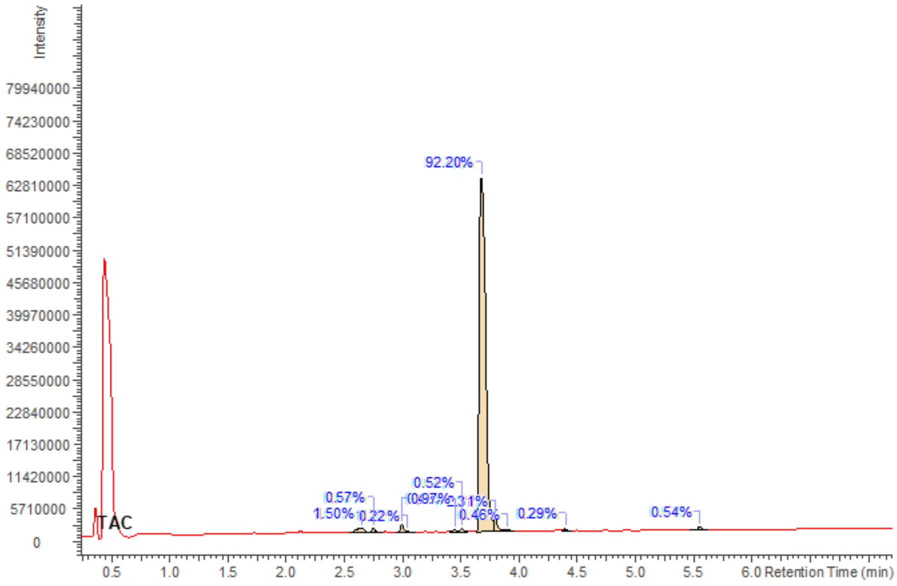

NMR of purified product: ^{1}H-NMR, ^{13}C-NMR

^{1}H-NMR (600 MHz, DMSO-d6), contains PEG signal at 3.5 ppm that could not be removed by purification

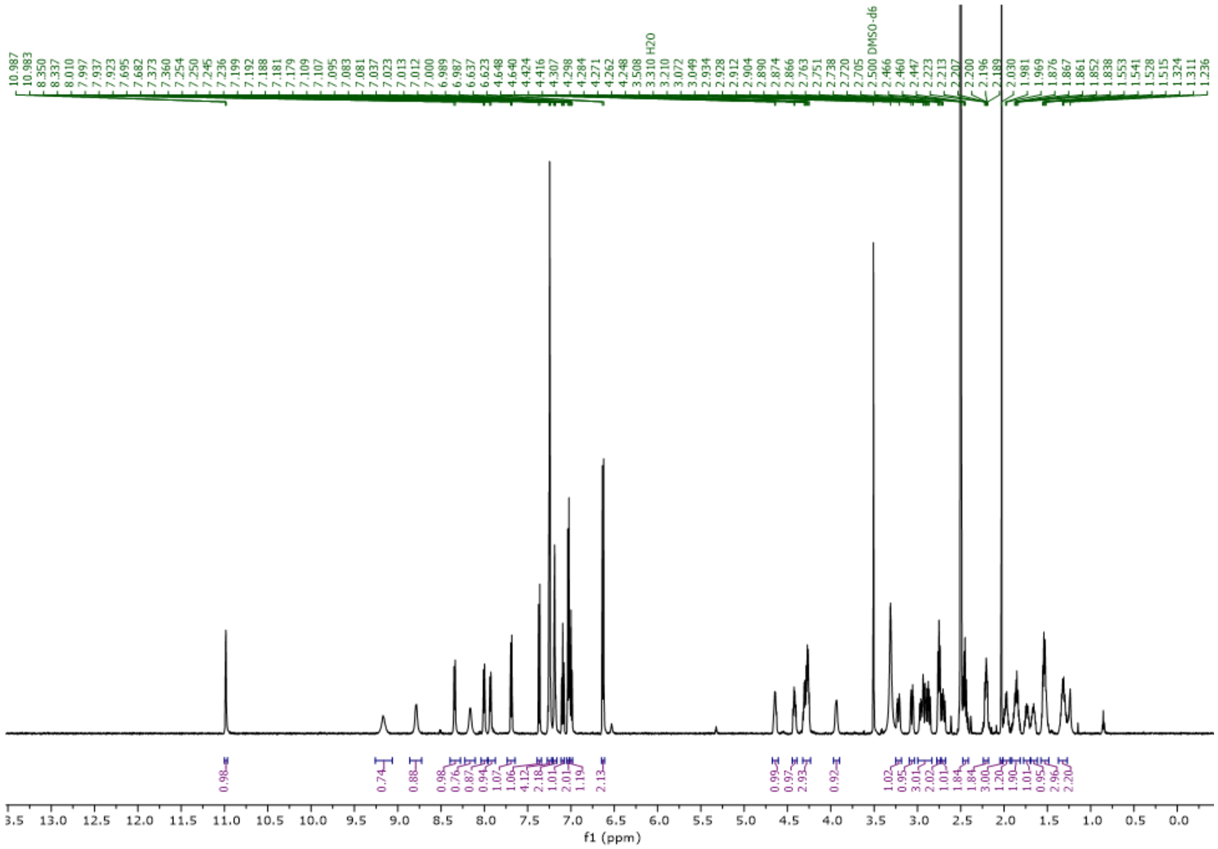

^{13}C-NMR (151 MHz, DMSO-d6), PEG signal at 69.79 ppm

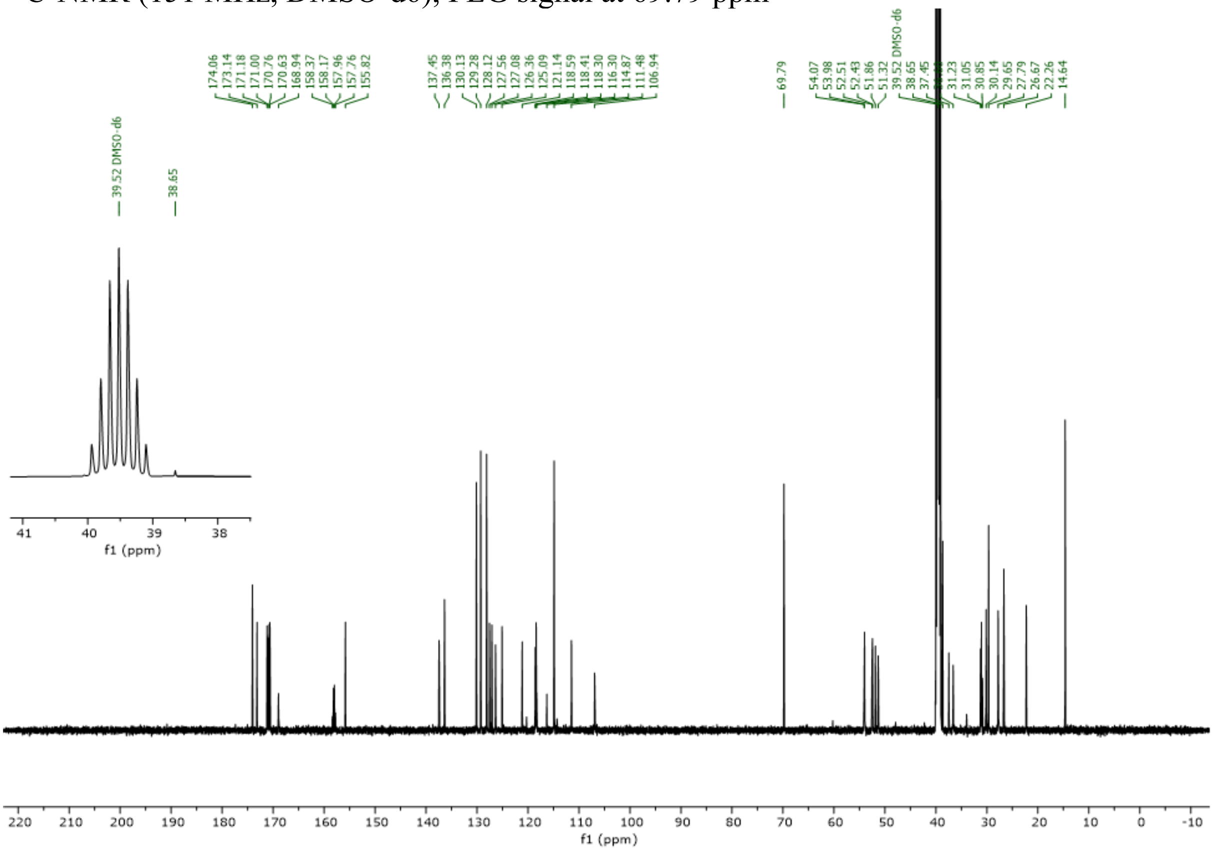

[13C]9C

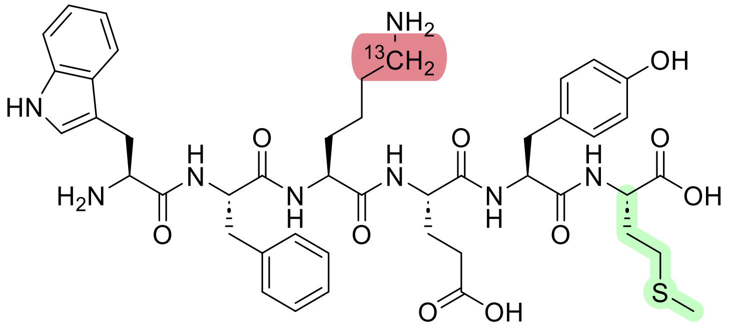

[13C]9C

Chemical Formula: $C_{44}{}^{13}CH_{58}N_8O_{10}S$

Exact Mass: 903.4030

LC-MS of crude: ca. 55% purity (ca. 3-4x product peaks, respectively 36%, 9% and 8%, maybe also 2%), below LC-MS is TIC of the product

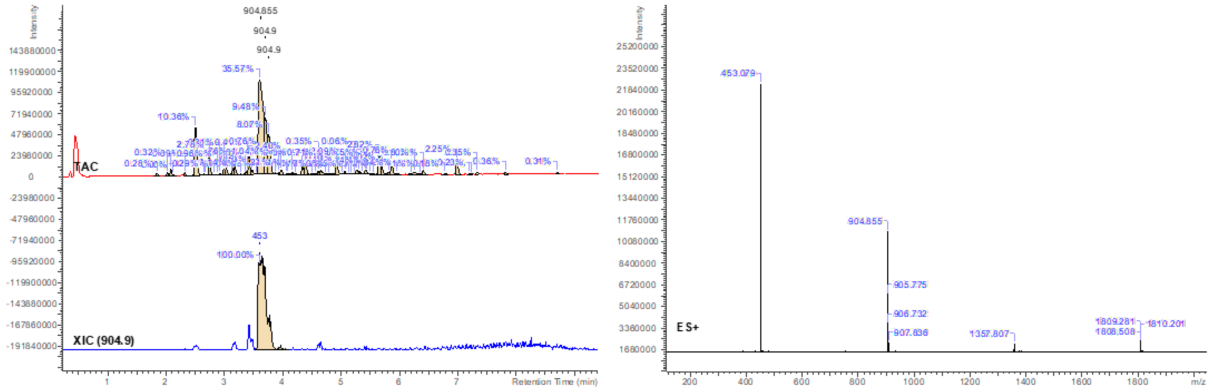

LC-MS of purified product: ca. 95% UV-purity (2x product peak, respectively 92% and 3%)

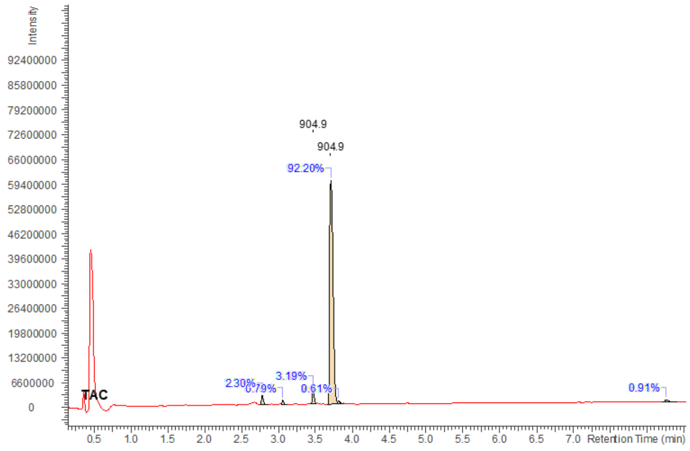

NMR

^{1}H NMR (600 MHz, DMSO-d6)

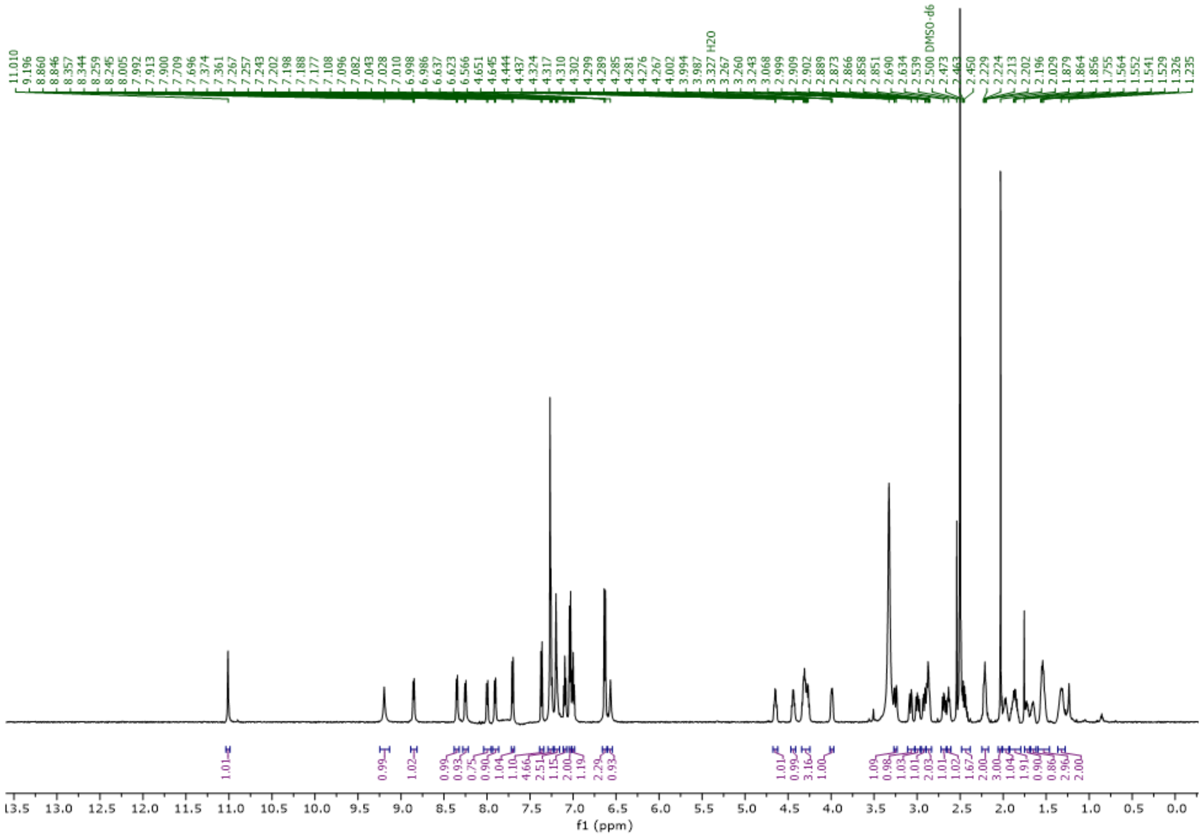

^{13}C-NMR (151 MHz, DMSO-d6): ^{13}C-label at 38.67 ppm.

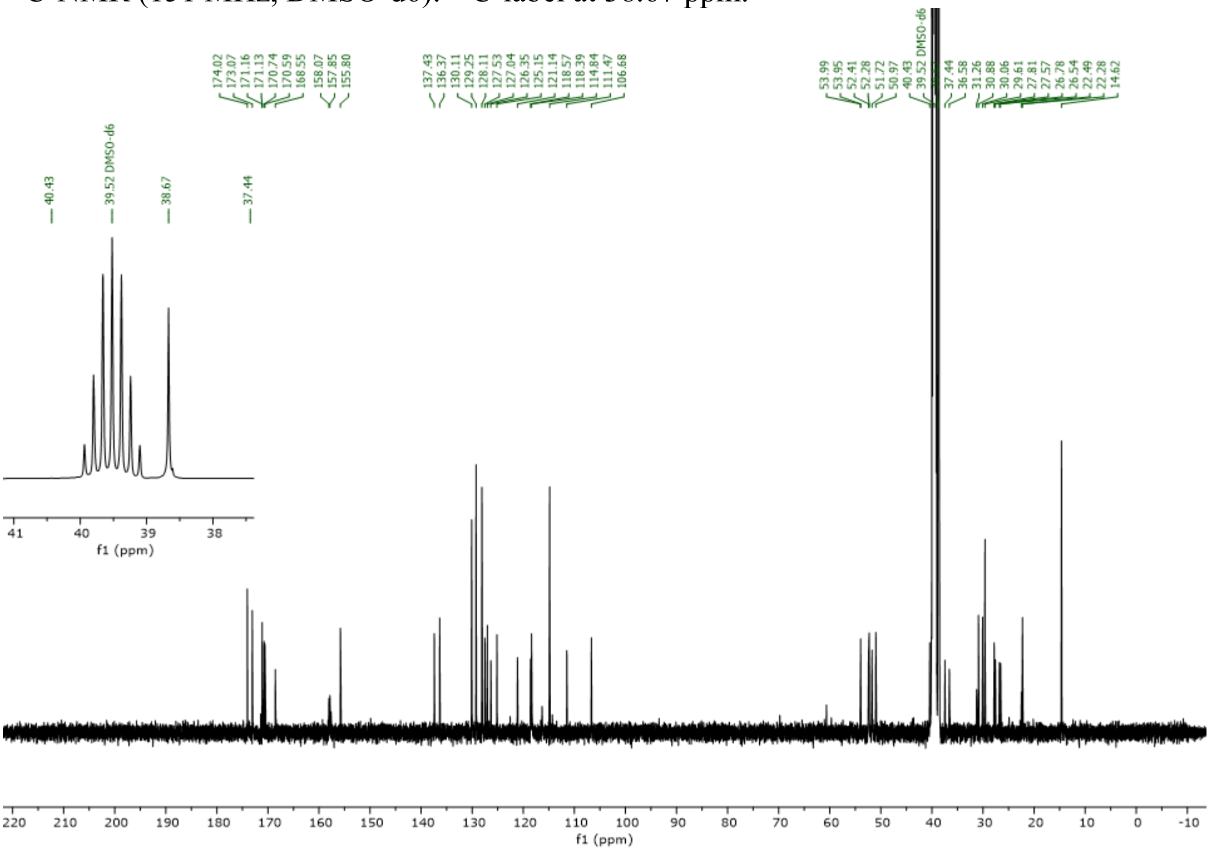

10.2.10 Hexapeptide 10C

[^{12}C]10C

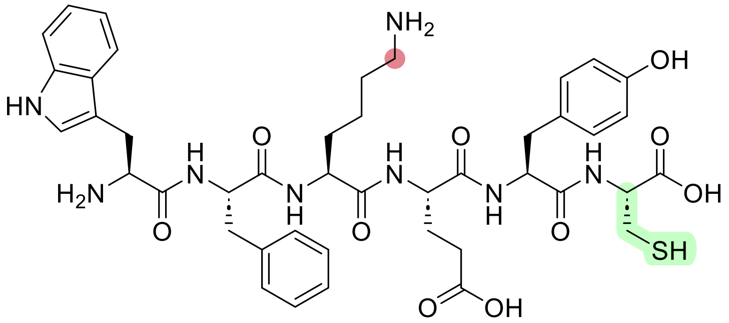

[^{12}C]10C

Chemical Formula: $C_{43}H_{54}N_8O_{10}S$

Exact Mass: 874.3684

LC-MS of crude: ca. 62% UV-purity (2x product peak, respectively 50% and 12%)

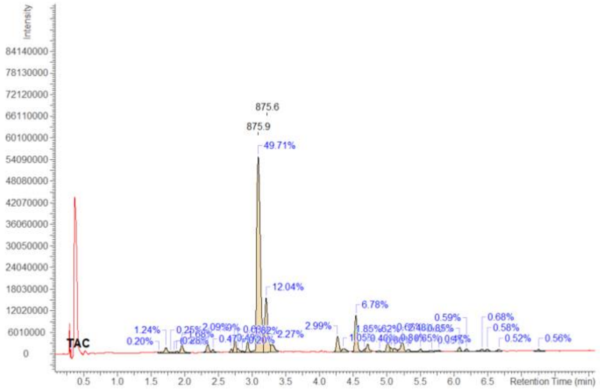

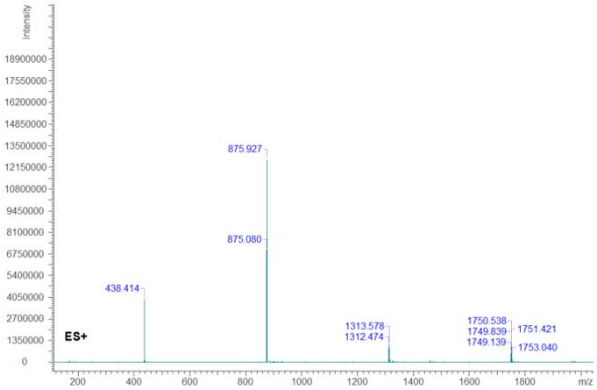

LC-MS of purified product: ca. 91% UV-purity (2x product peak, respectively 73% and 18%)

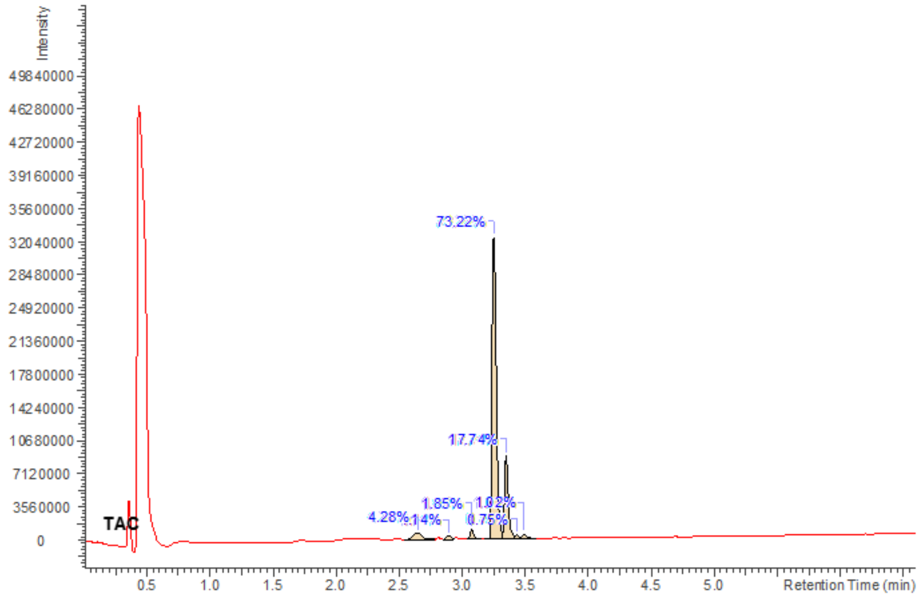

NMR of purified product: ^{1}H-NMR, ^{13}C-NMR

^{1}H-NMR (600 MHz, DMSO-d6), contains PEG signal at 3.5 ppm that could not be removed by purification

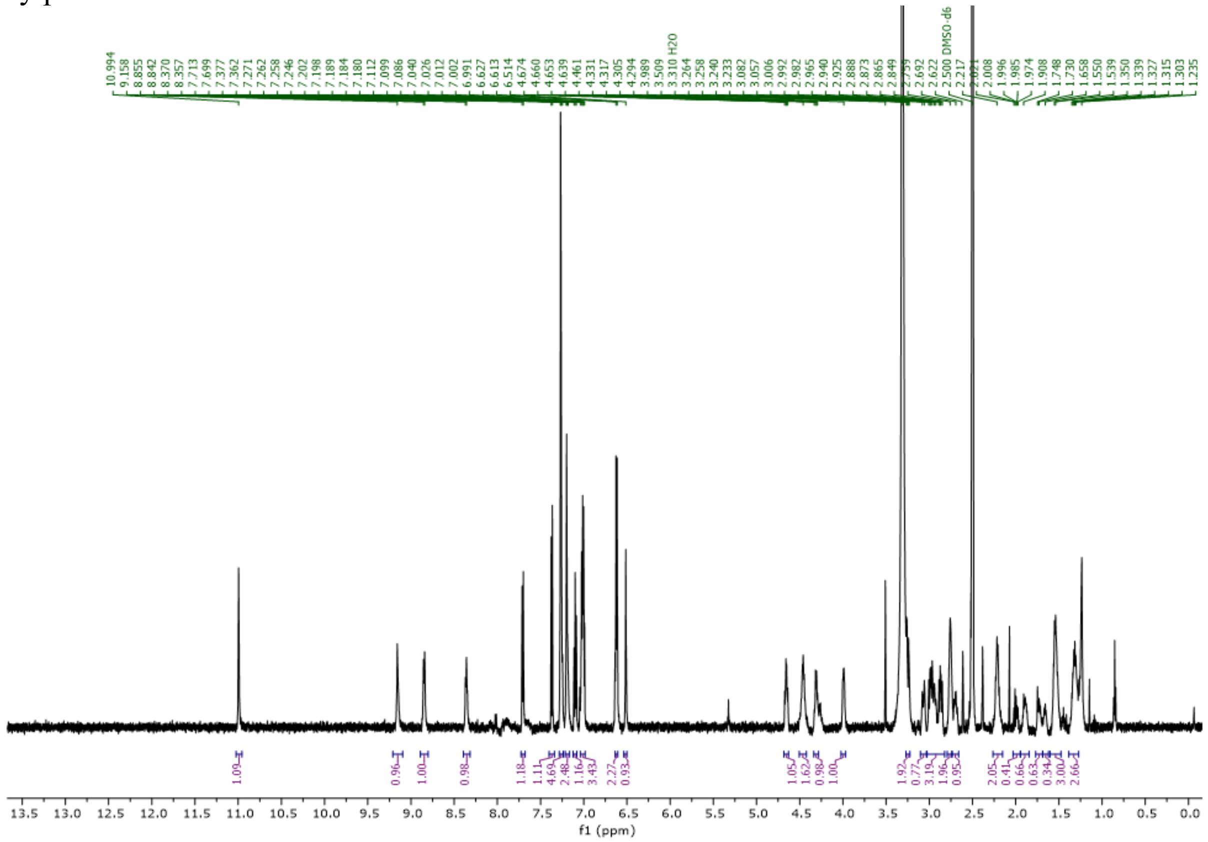

^{13}C-NMR (151 MHz, DMSO-d6), small PEG signal at 69.77 ppm

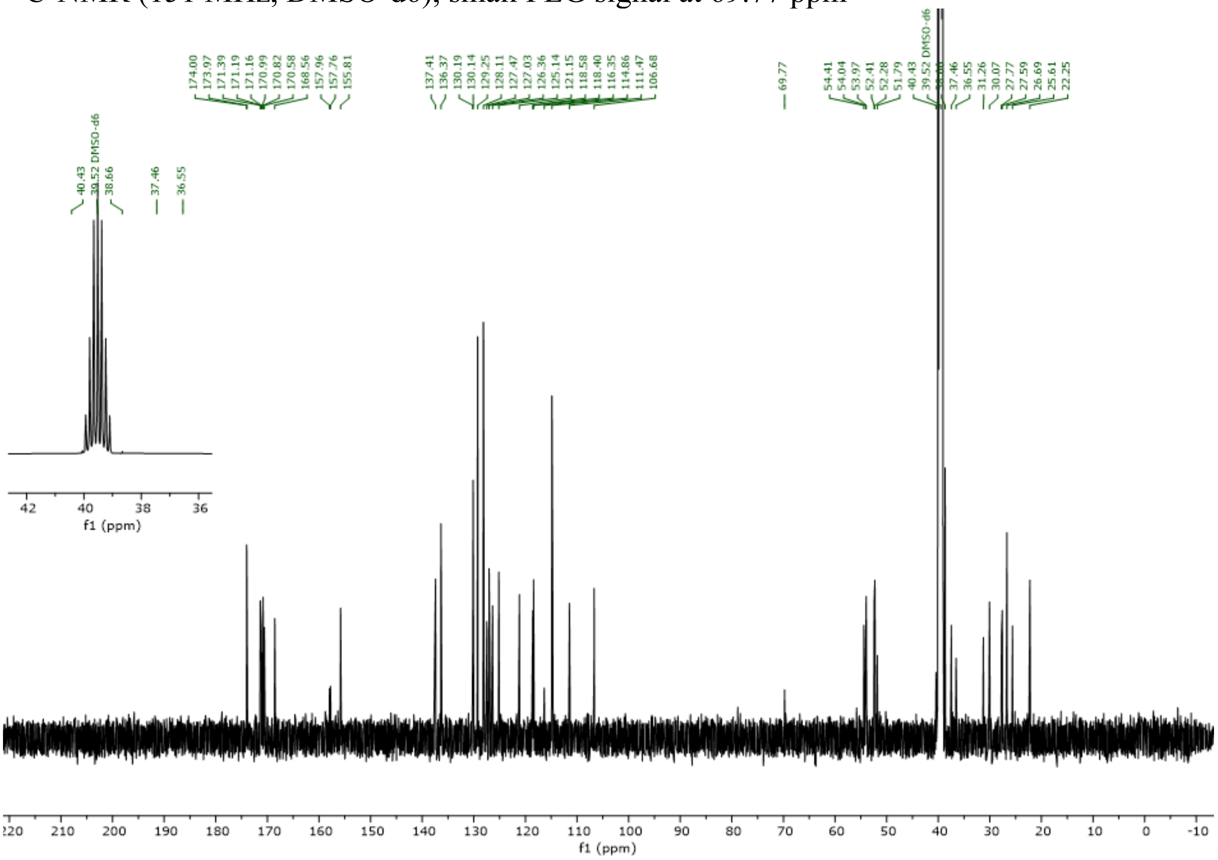

[^{13}C]10C

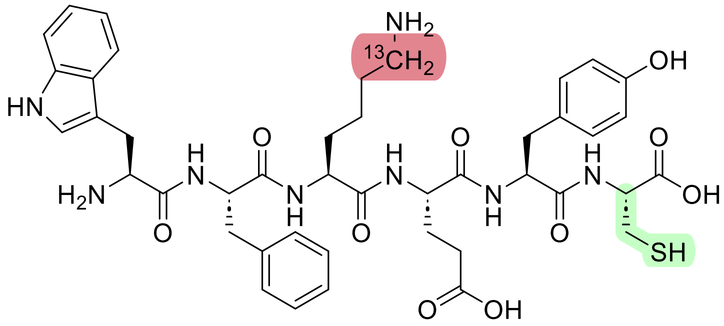

[^{13}C]10C

Chemical Formula: $C_{42}{}^{13}CH_{54}N_8O_{10}S$

Exact Mass: 875.3717

LC-MS of crude: ca. 58% UV-purity (2-3x product peak, respectively 44%, 12% and 2%)

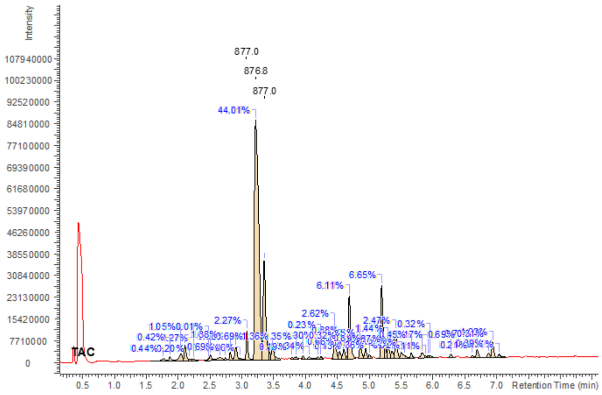

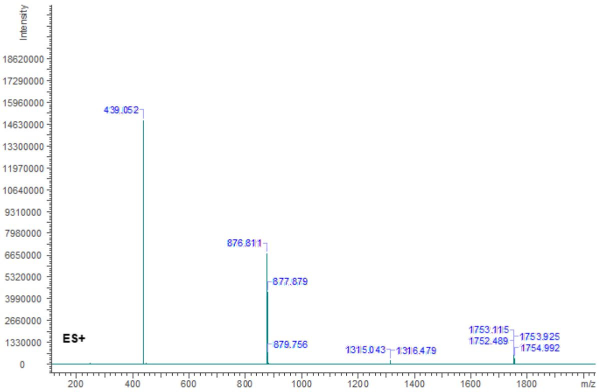

Isotope distribution comparison of the dimerized product: Predicted non-labeled vs labeled peptide, ca. 1.98 ^{13}C/dimer incorporation

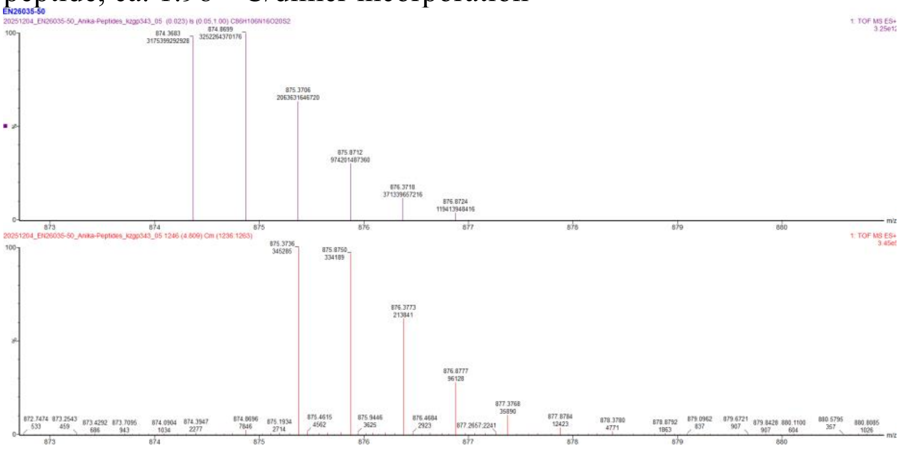

Results	
Relative amounts [%]	
unlabeled	0.7
1-label	1.7
2-label	101.2
3-label	-4.9
4-label	2.7
5-label	-2.0
6-label	0.7

10.2.11 11C (STAT3-Hel2A-2)

[¹²C]11C (with DMSO:H_2O, 95:5)

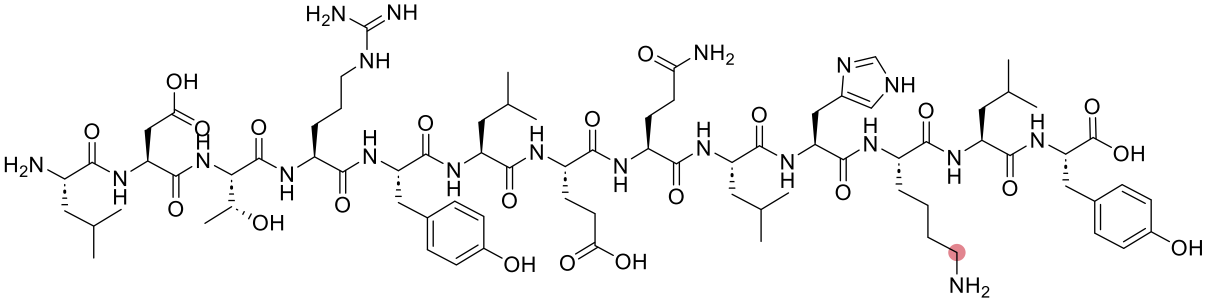

[¹²C]11C

Chemical Formula: $C_{78}H_{122}N_{20}O_{22}$

Exact Mass: 1690.9043

LC-MS of crude: ca. 67% UV-purity (2x product peaks, respectively 65% and 2%)

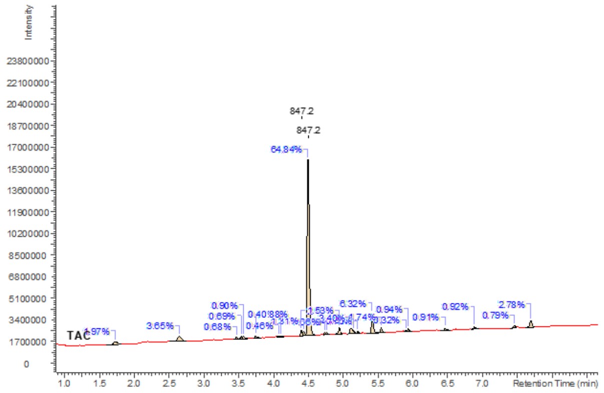

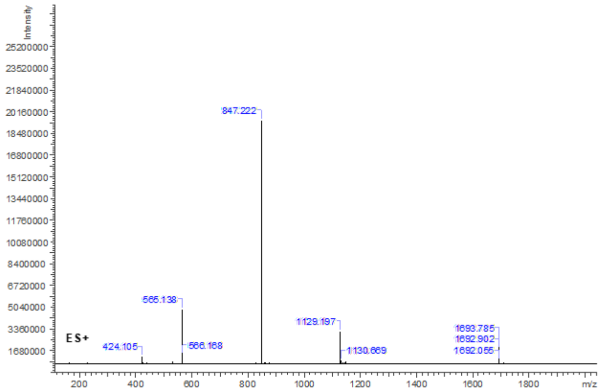

LC-MS of purified product: ca. 78% UV-purity (2-3x product peaks, respectively 74%, 3% and 1%); 15% of peptide that has an additional mass of ca. 1 Da at 4.6 min

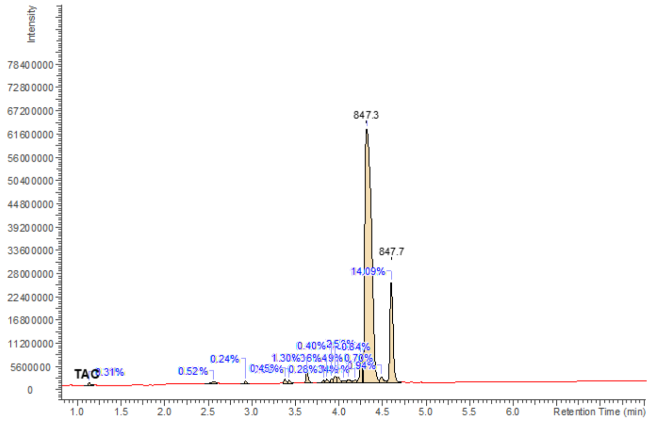

NMR of purified product: ^{1}H-NMR, ^{13}C-NMR

^{1}H-NMR (600 MHz, DMSO-d6)

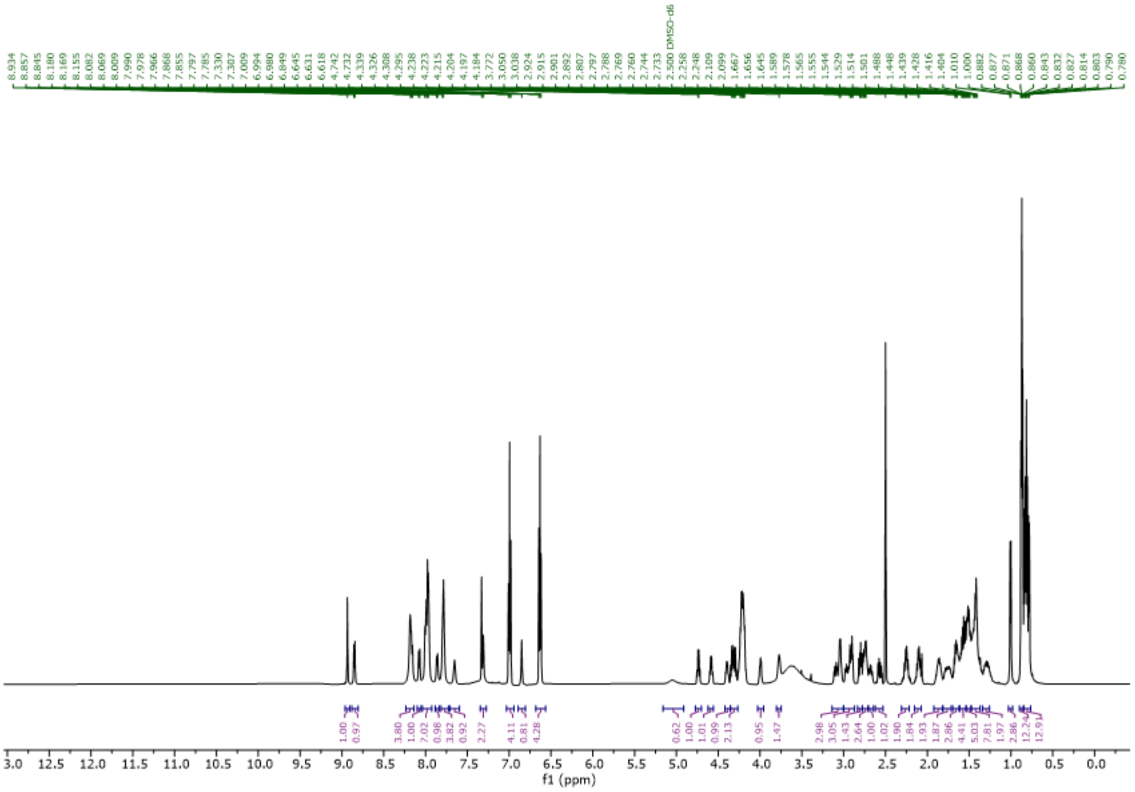

^{13}C-NMR (151 MHz, DMSO-d6)

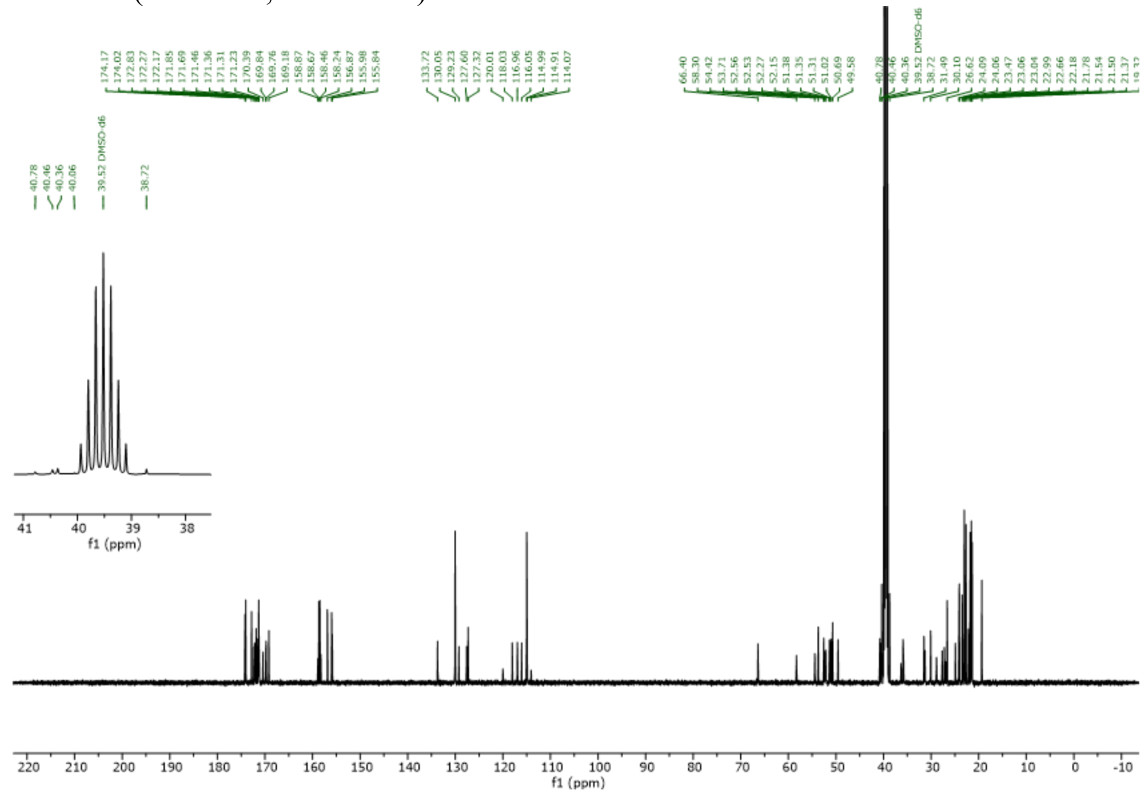

[^{13}C]11C (with DMSO:H_2O, 95:5)

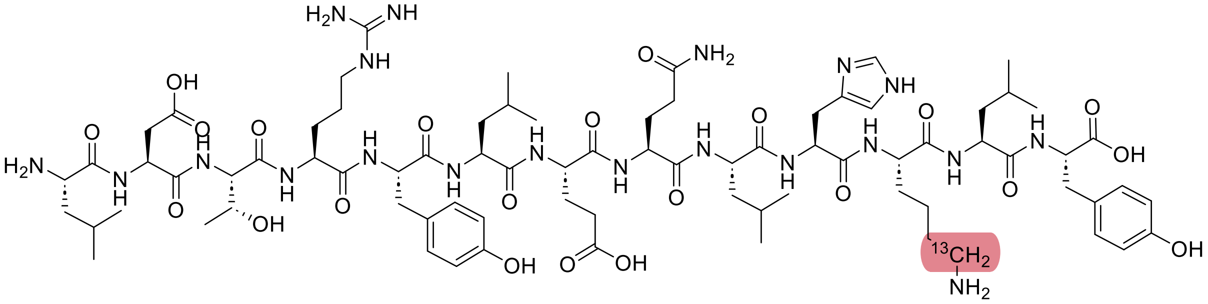

[^{13}C]11C

Chemical Formula: $C_{77}{}^{13}CH_{122}N_{20}O_{22}$

Exact Mass: 1691.9076

LC-MS of crude: ca. 67% UV-purity (2-3x product peaks, respectively 64%, 2% and 1%)

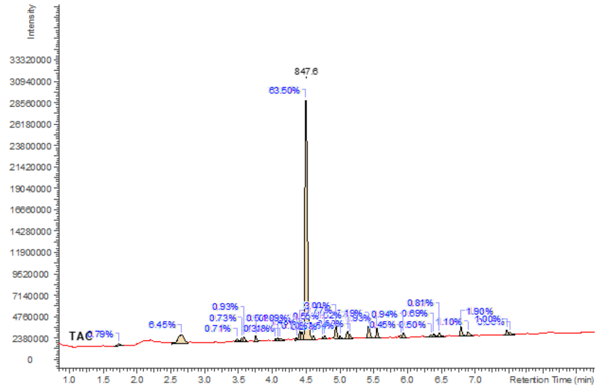

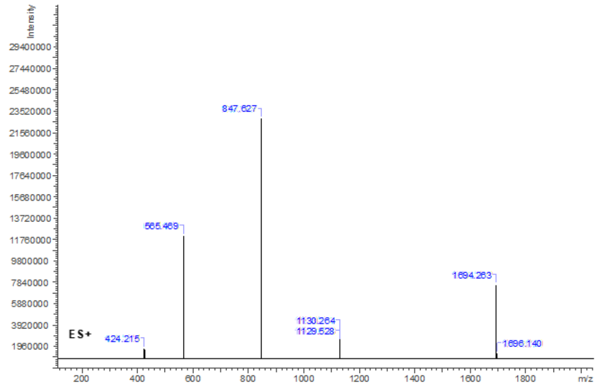

LC-MS of purified product: ca. 80% UV-purity (2x product peaks, respectively 77% and 3%); 8% of peptide that has an additional mass of ca. 1 Da at 4.6 min

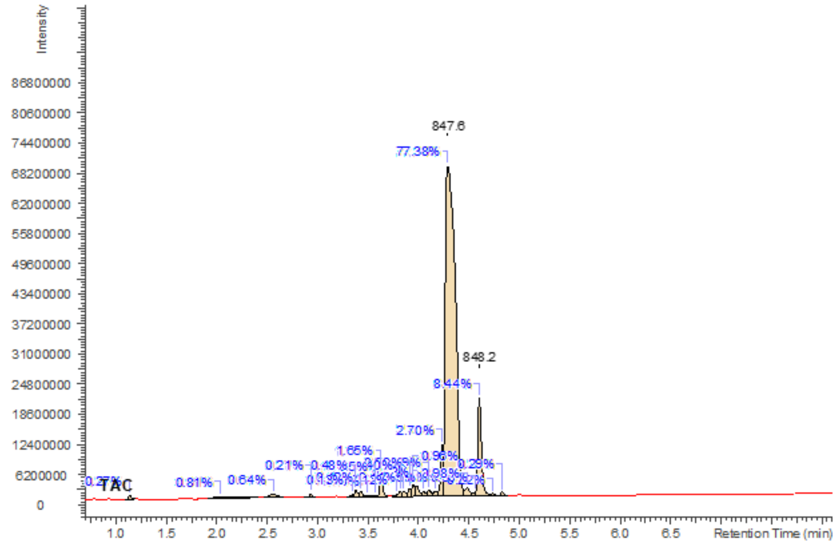

Isotope distribution comparison: Predicted non-labeled vs labeled peptide, ca. 0.99 ^{13}C/molecule incorporation

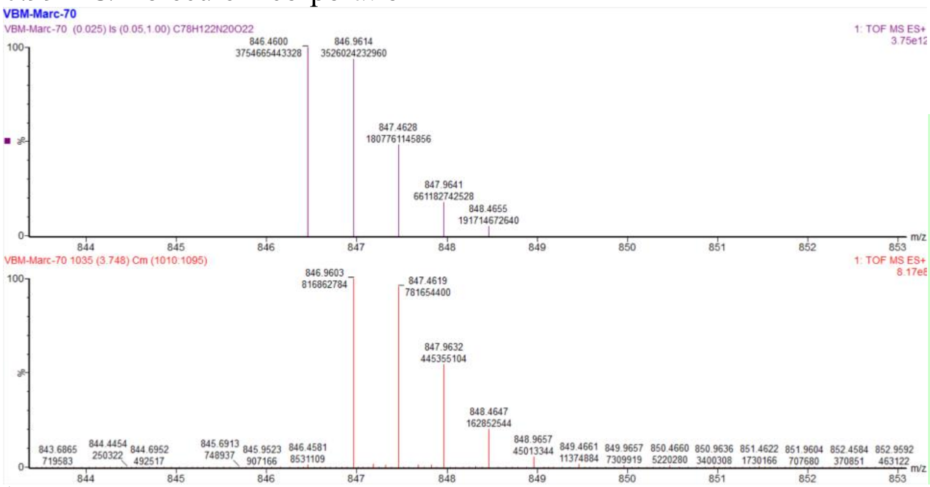

Results	
Relative amounts [%]	
unlabeled	1.0
1-label	93.5
2-label	2.1
3-label	4.3
4-label	-2.8
5-label	0.6
6-label	1.2
7-label	0.0
8-label	0.0

^{1}H-NMR (600 MHz, DMSO-d6)

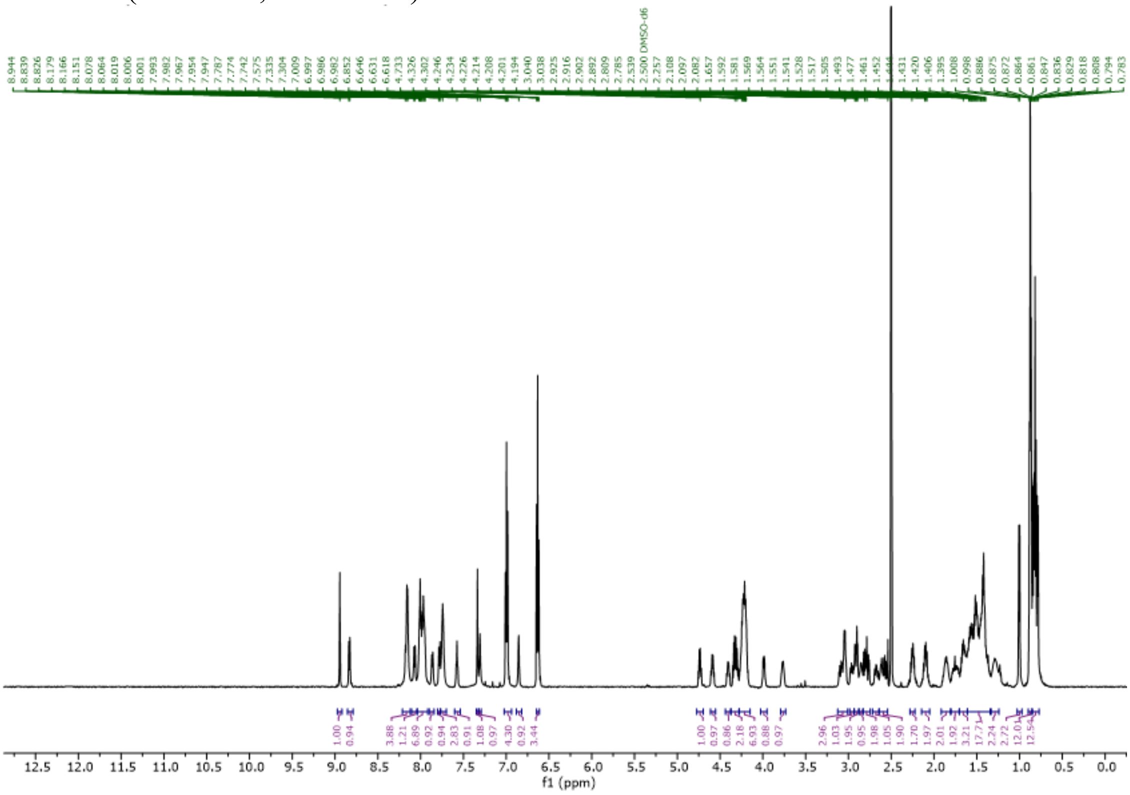

^{13}C-NMR (151 MHz, DMSO-d6): ^{13}C-label at 38.71 ppm.

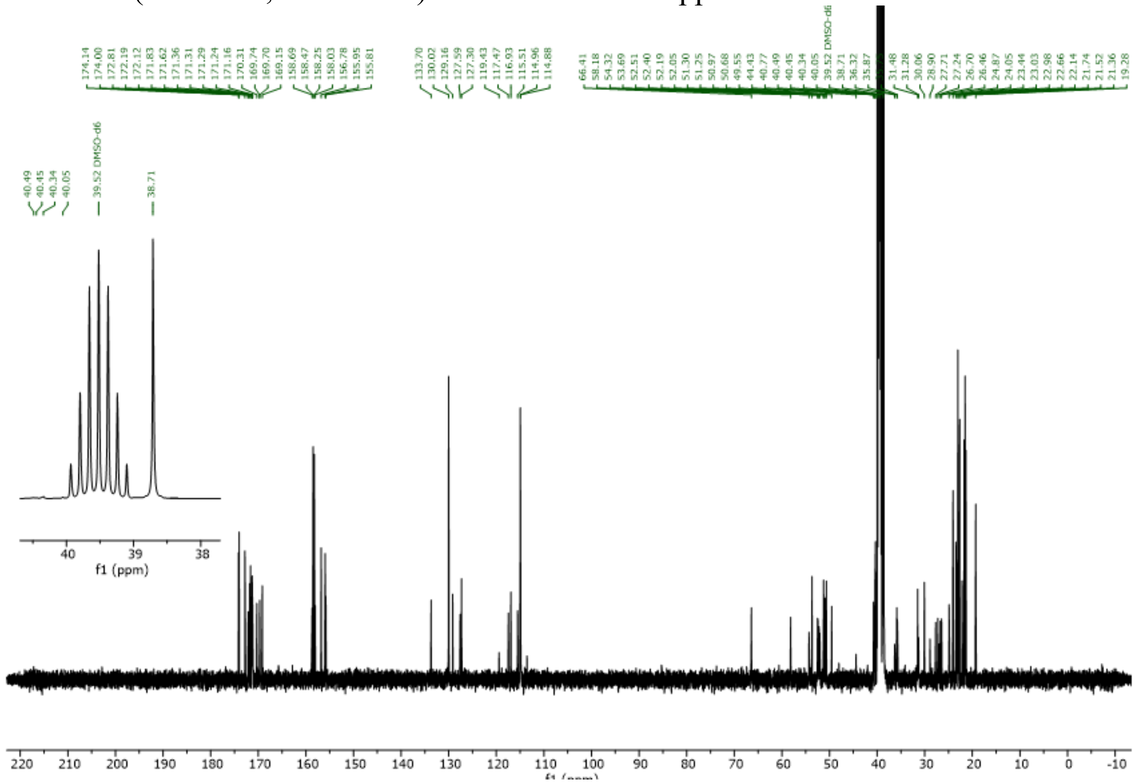

[^{13}C]11C (with neat DMSO)

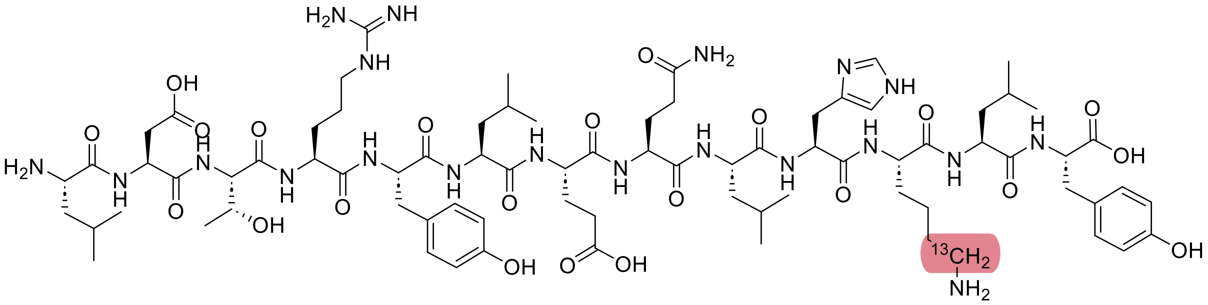

[^{13}C]11C

Chemical Formula: $C_{77}{}^{13}CH_{122}N_{20}O_{22}$

Exact Mass: 1691.9076

LC-MS of crude: ca. 76% UV-purity

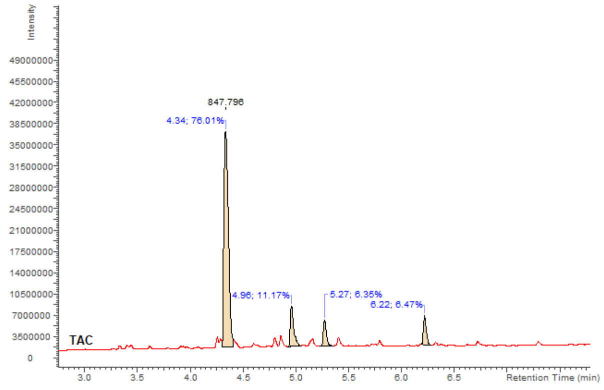

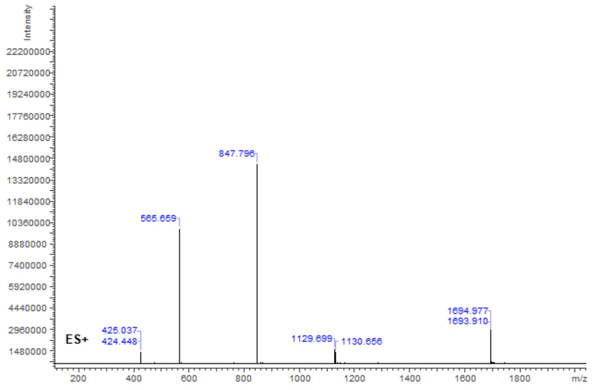

LC-MS of purified product: ca. 89% UV-purity

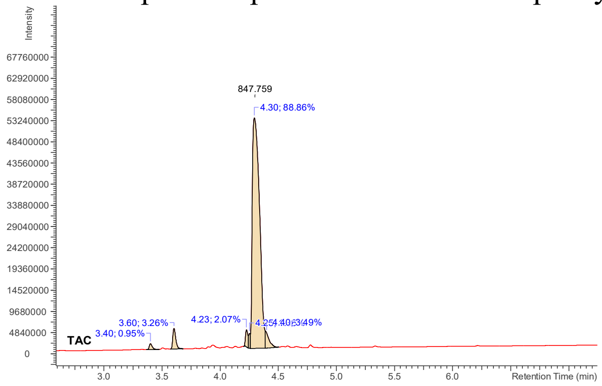

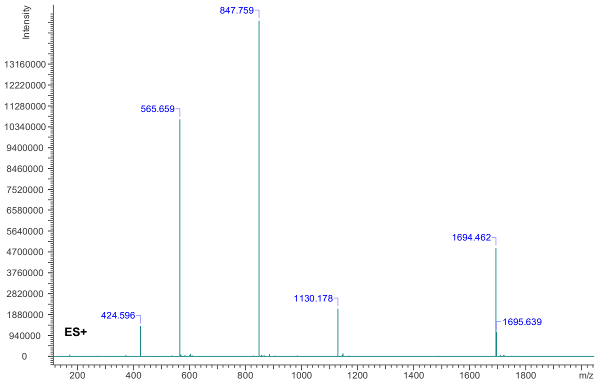

Isotope distribution comparison: Predicted non-labeled vs labeled peptide, ca. 0.99 ^{13}C/molecule incorporation

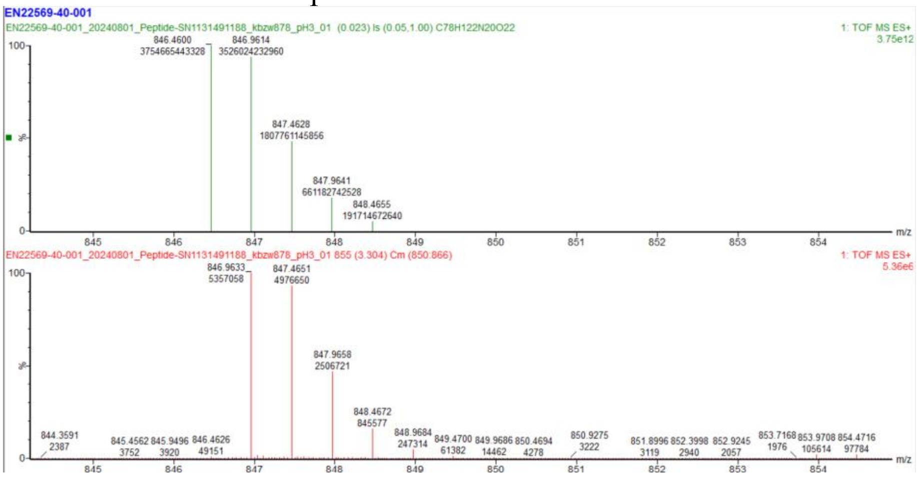

Results	
Relative amounts [%]	
unlabeled	0.9
1-label	99.4
2-label	-0.6
3-label	-0.5
4-label	-0.9
5-label	0.8
6-label	1.0

NMR of purified product: ^{1}H-NMR, ^{13}C-NMR, HSQC, HMBC, COSY

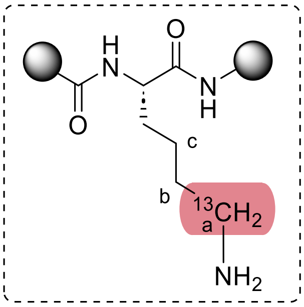

a: ^{13}C at 38.80 ppm, ^{1}H medium at 2.74 ppm (HSQC), split to 2.84 and 2.62 ppm (HMBC)
b and **c**: ^{1}H at 1.51 ppm and 1.27 ppm (HMBC)
NH_2: ^{1}H at 7.73 ppm (HMBC)

^{1}H-NMR (600 MHz, DMSO-d6)

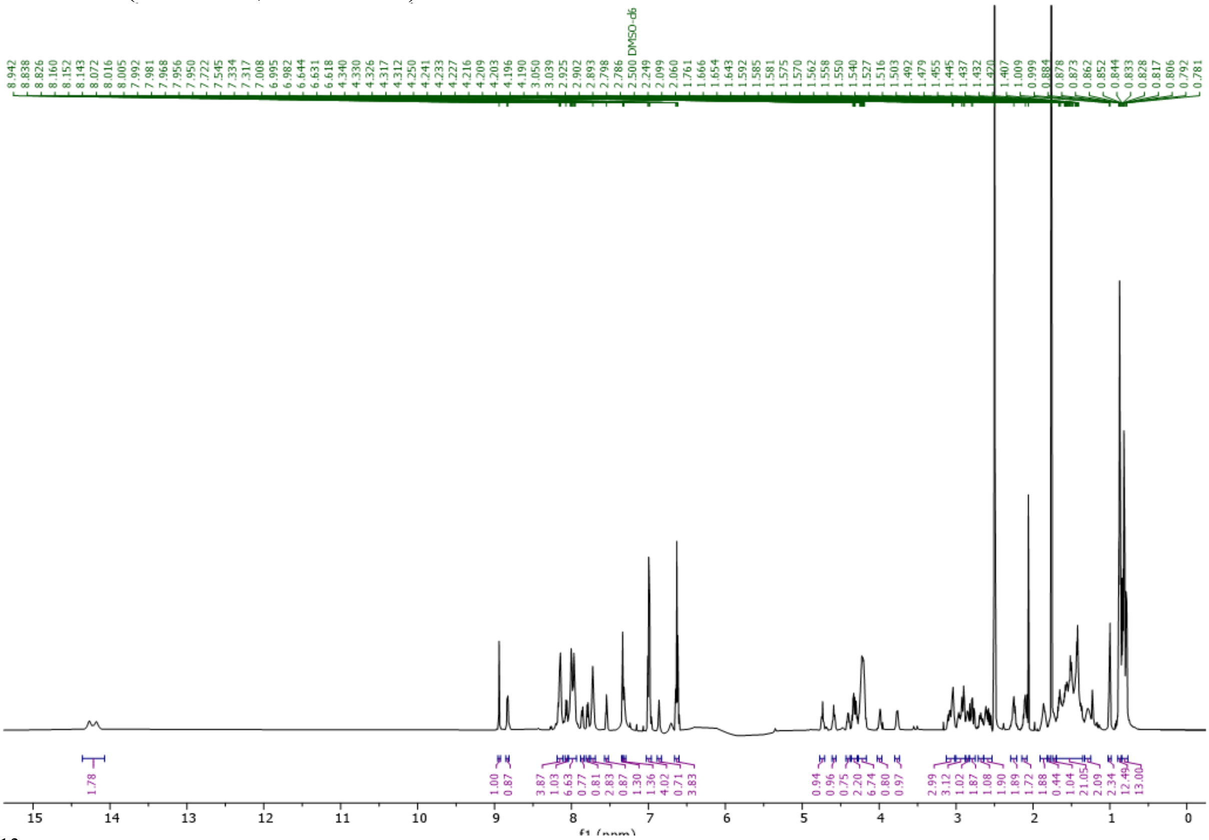

^{13}C-NMR (151 MHz, DMSO-d6)

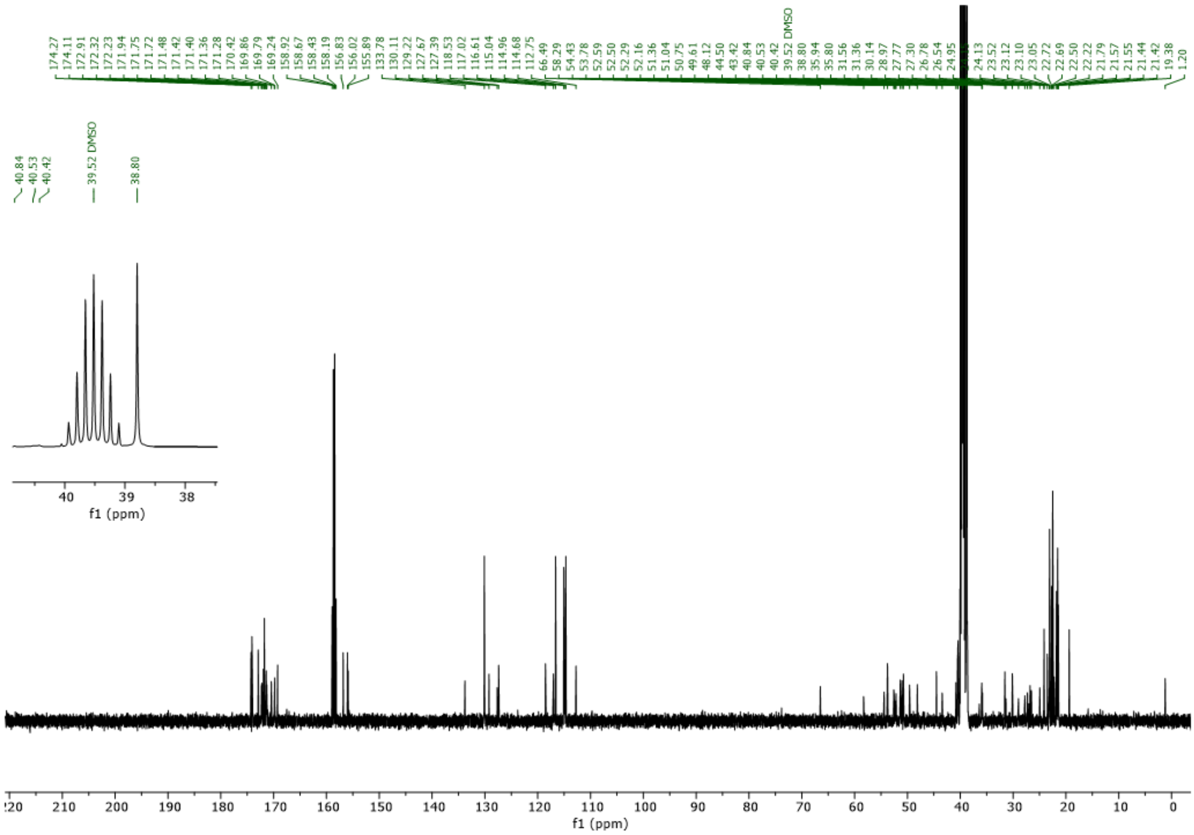

HSQC (600 MHz, DMSO-d6)

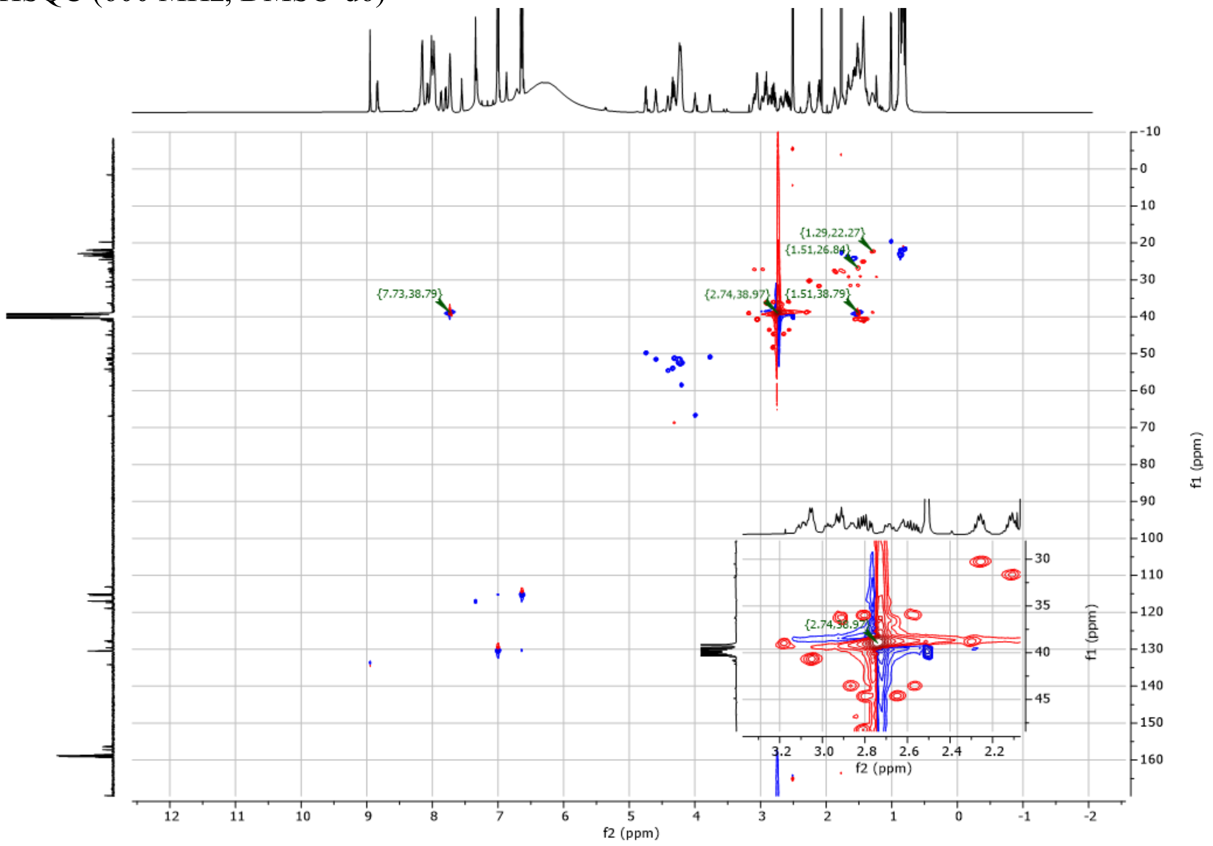

HMBC (600 MHz, DMSO-d6)

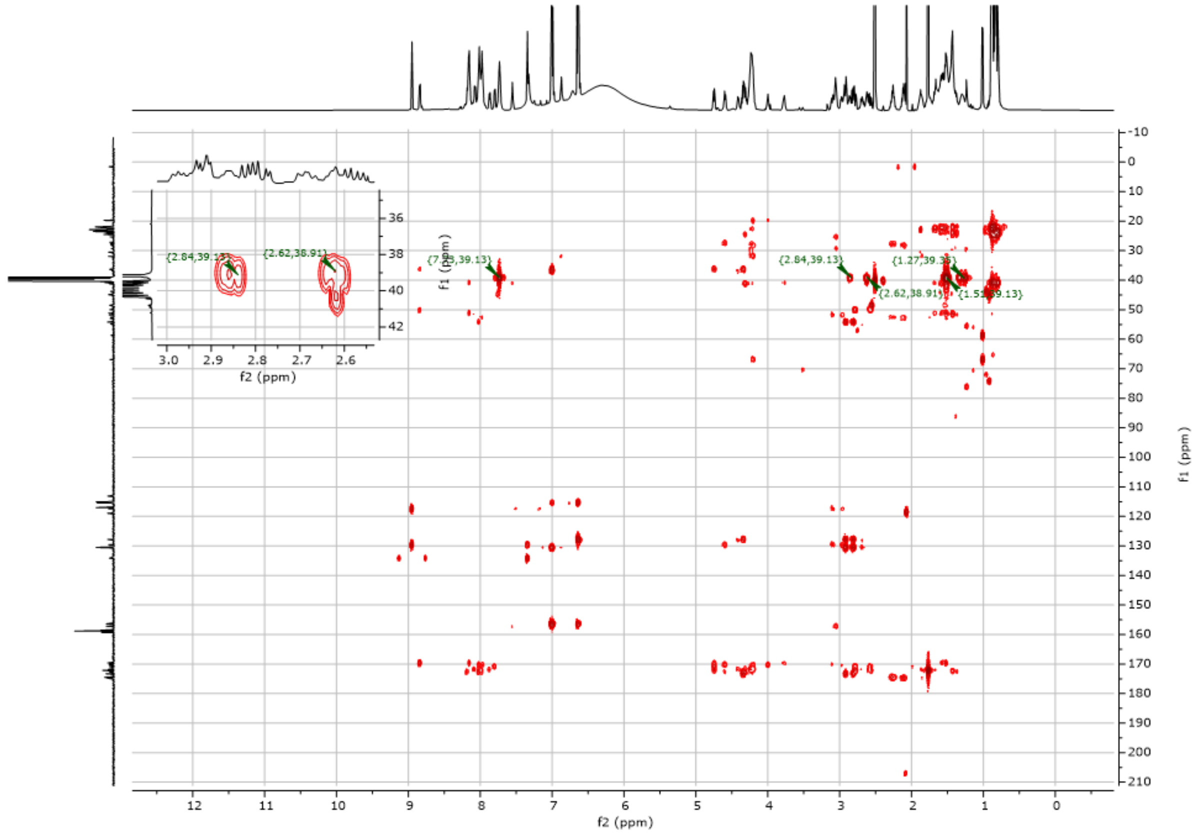

COSY (600 MHz, DMSO-d6)

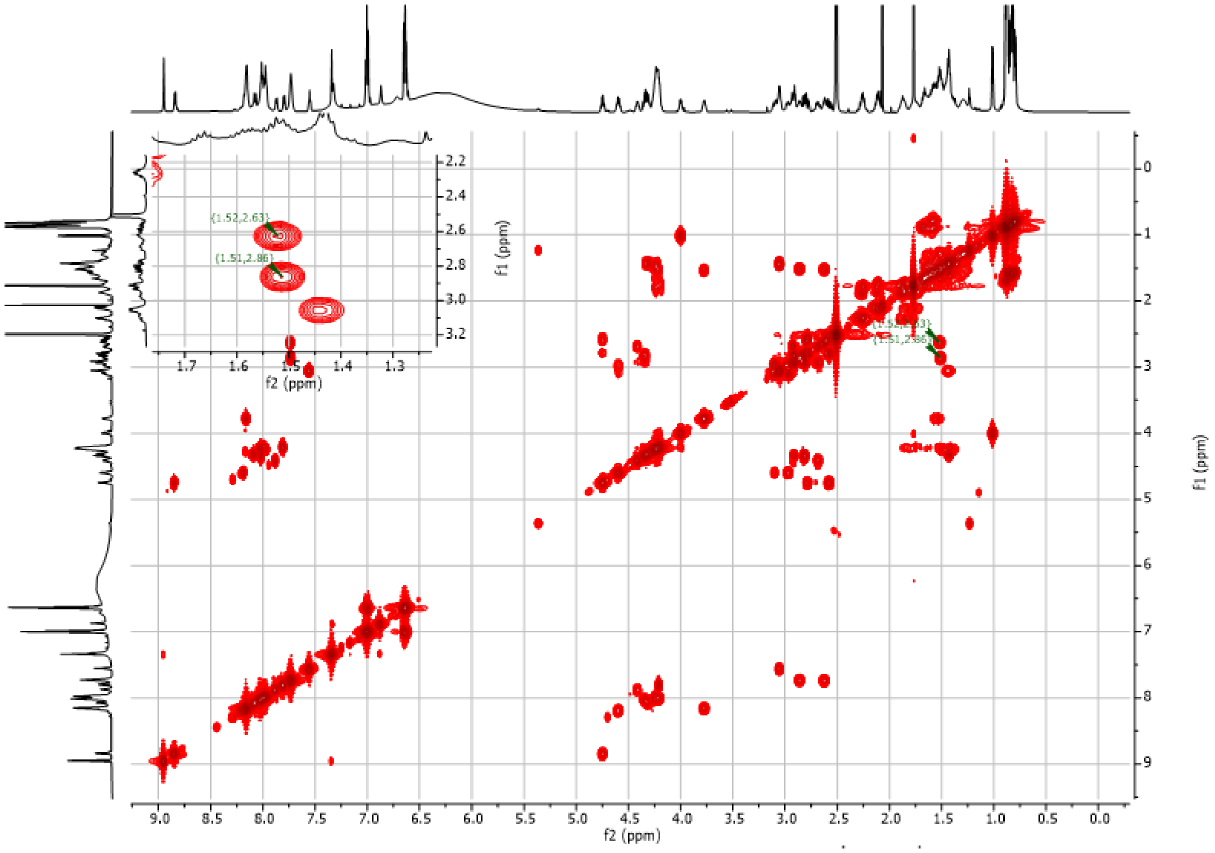

[^{2}H]11C (with neat DMSO)

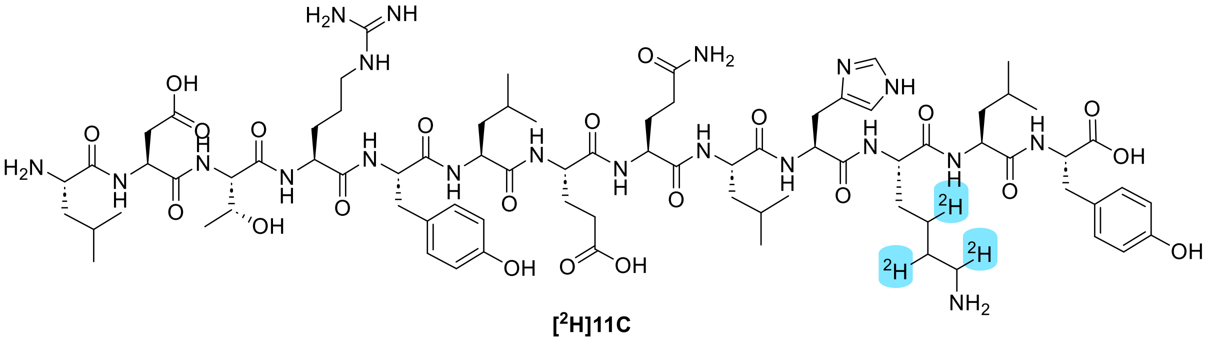

[^{2}H]11C

Chemical Formula: $C_{78}H_{119}D_3N_{20}O_{22}$

Exact Mass: 1693.9231

LC-MS of crude (both batches combined): ca. 46% UV-purity

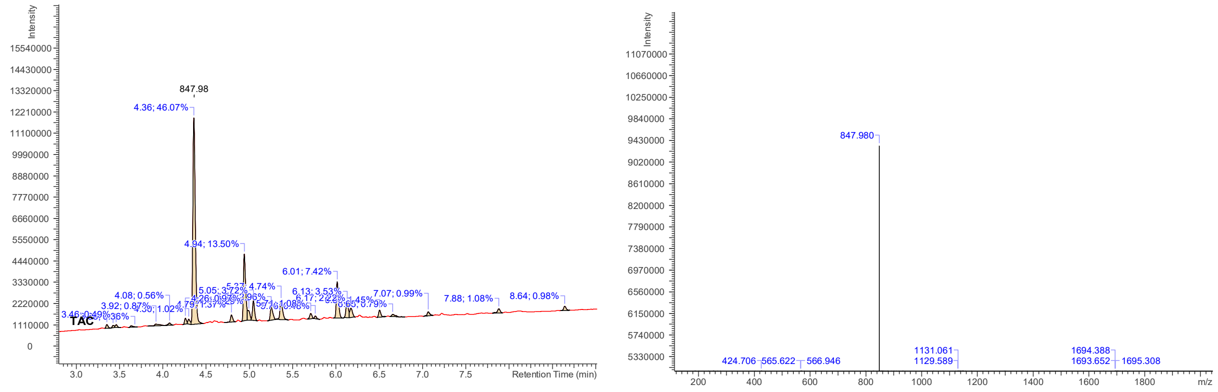

LC-MS of purified product: ca. 97% UV-purity

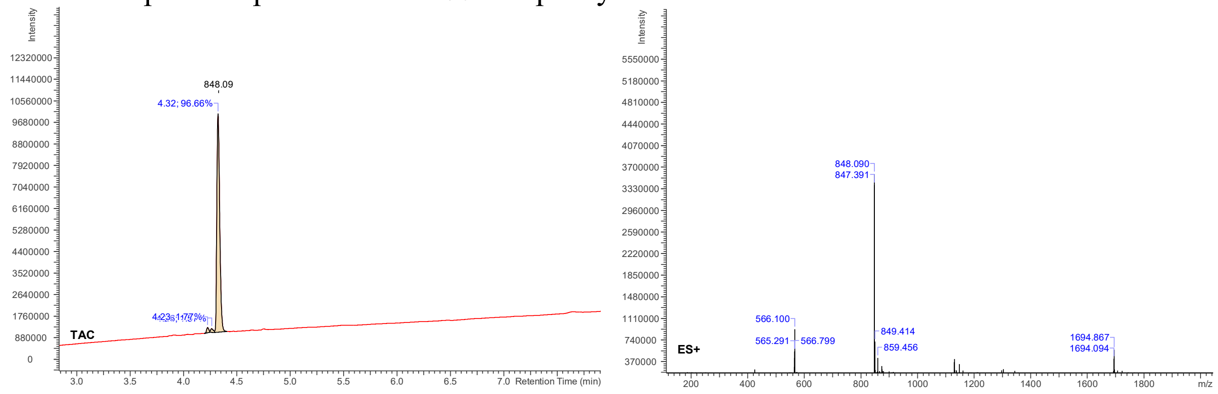

Isotope distribution comparison: Predicted non-labeled vs labeled peptide, ca. 1.15 ^{2}H/molecule incorporation

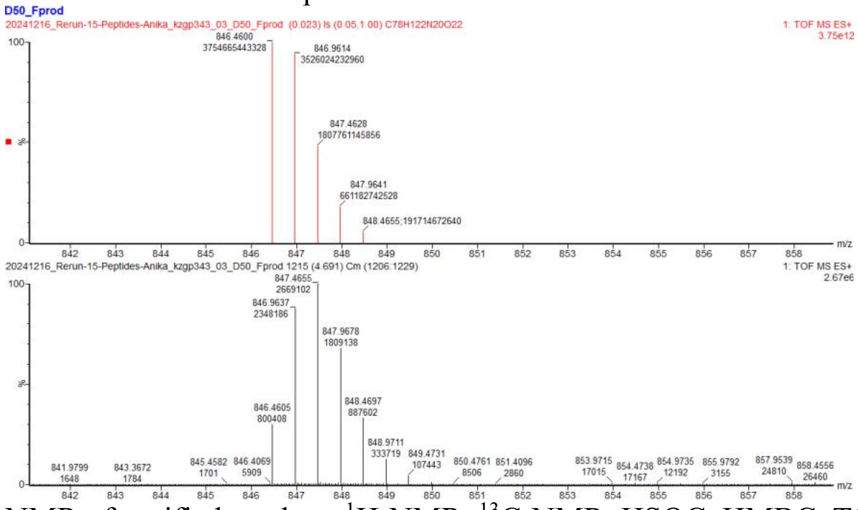

Results	
Relative amounts [%]	
unlabeled	23.5
1-label	46.9
2-label	23.1
3-label	4.8
4-label	1.0
5-label	0.1
6-label	0.6

NMR of purified product: ^{1}H-NMR, ^{13}C-NMR, HSQC, HMBC, TOCSY

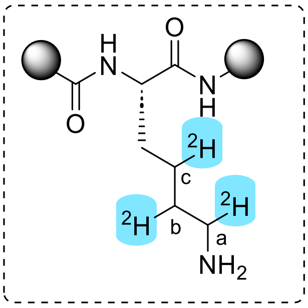

^{1}H-NMR (600 MHz, DMSO-d6)

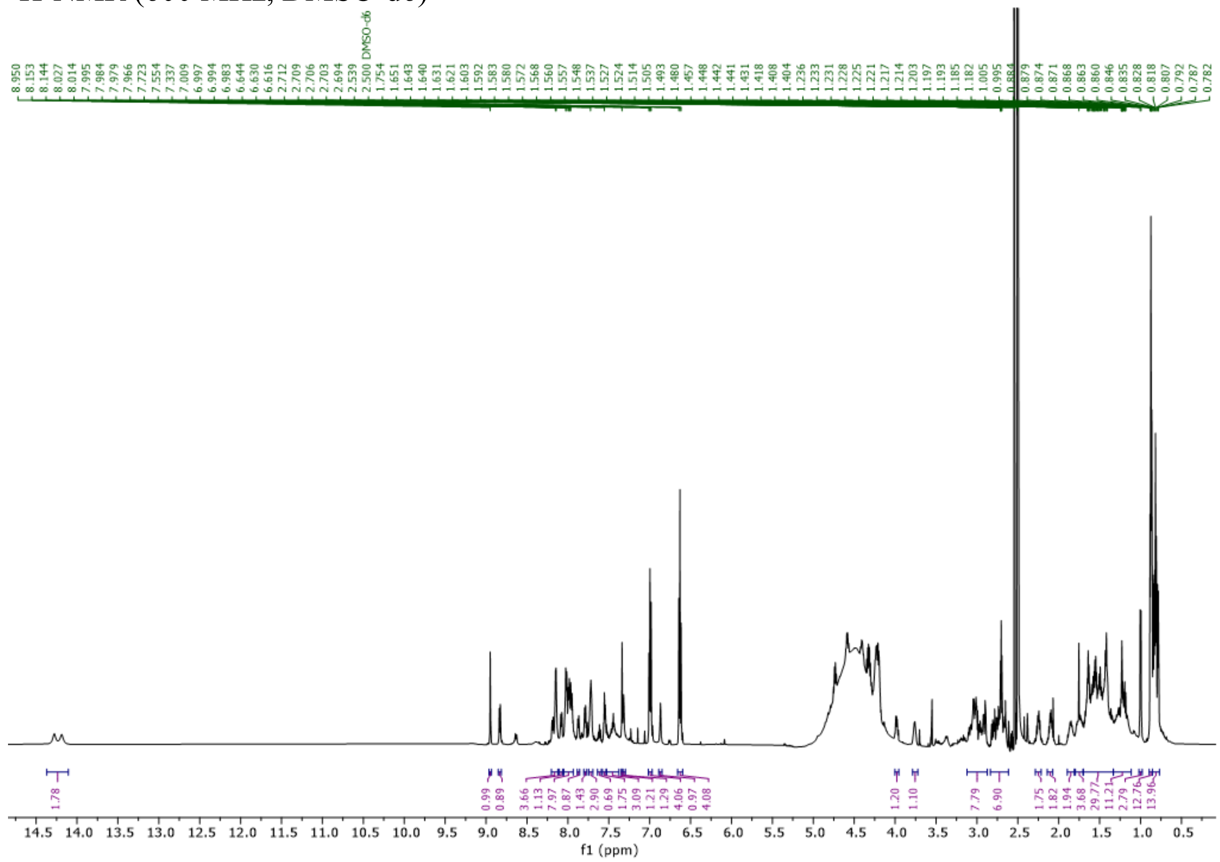

^{13}C-NMR (151 MHz, DMSO-d6)

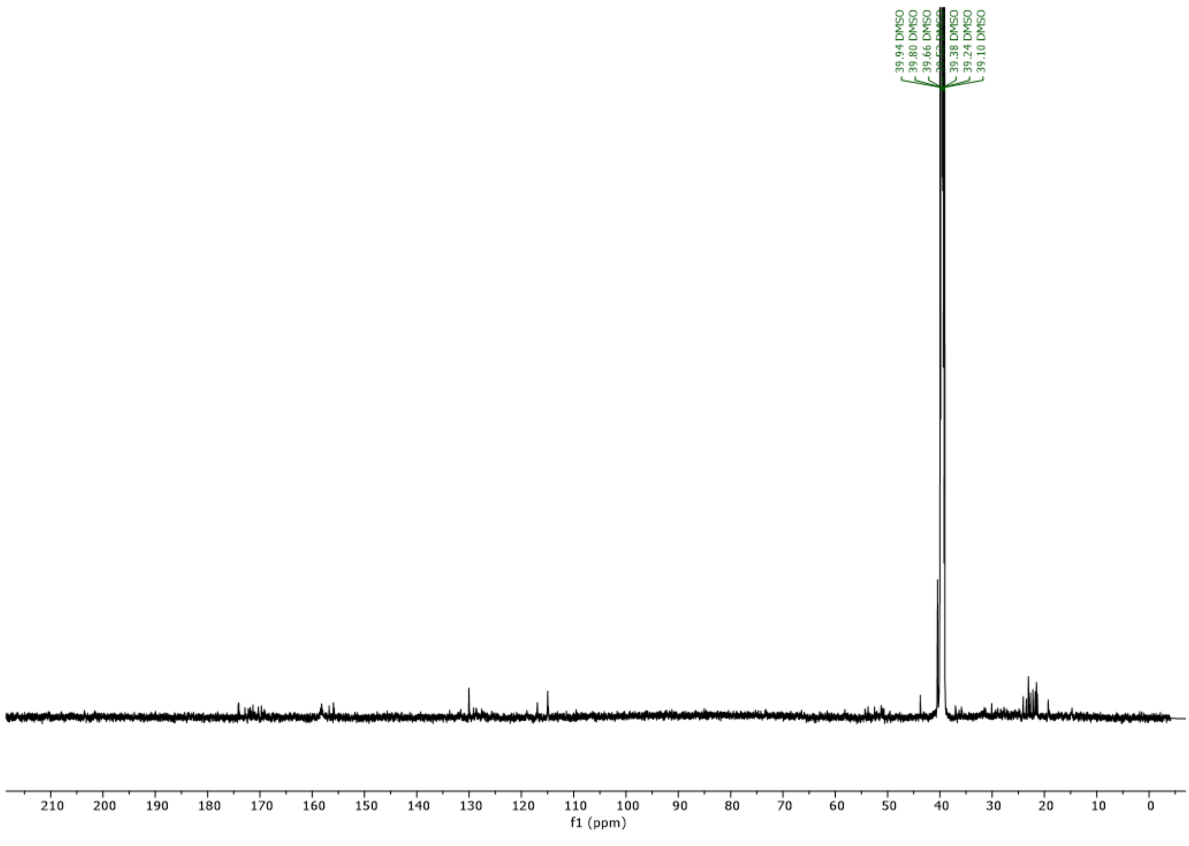

HSQC (600 MHz, DMSO-d6)

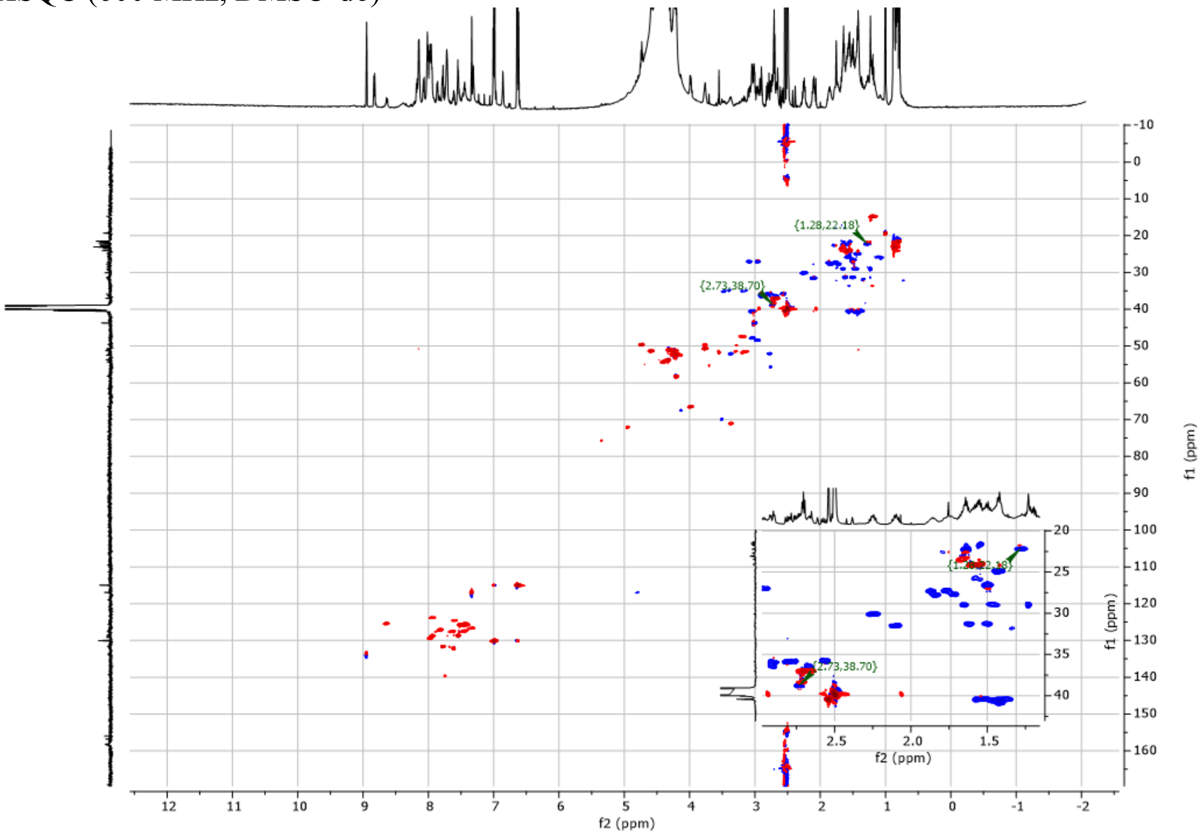

HMBC (600 MHz, DMSO-d6)

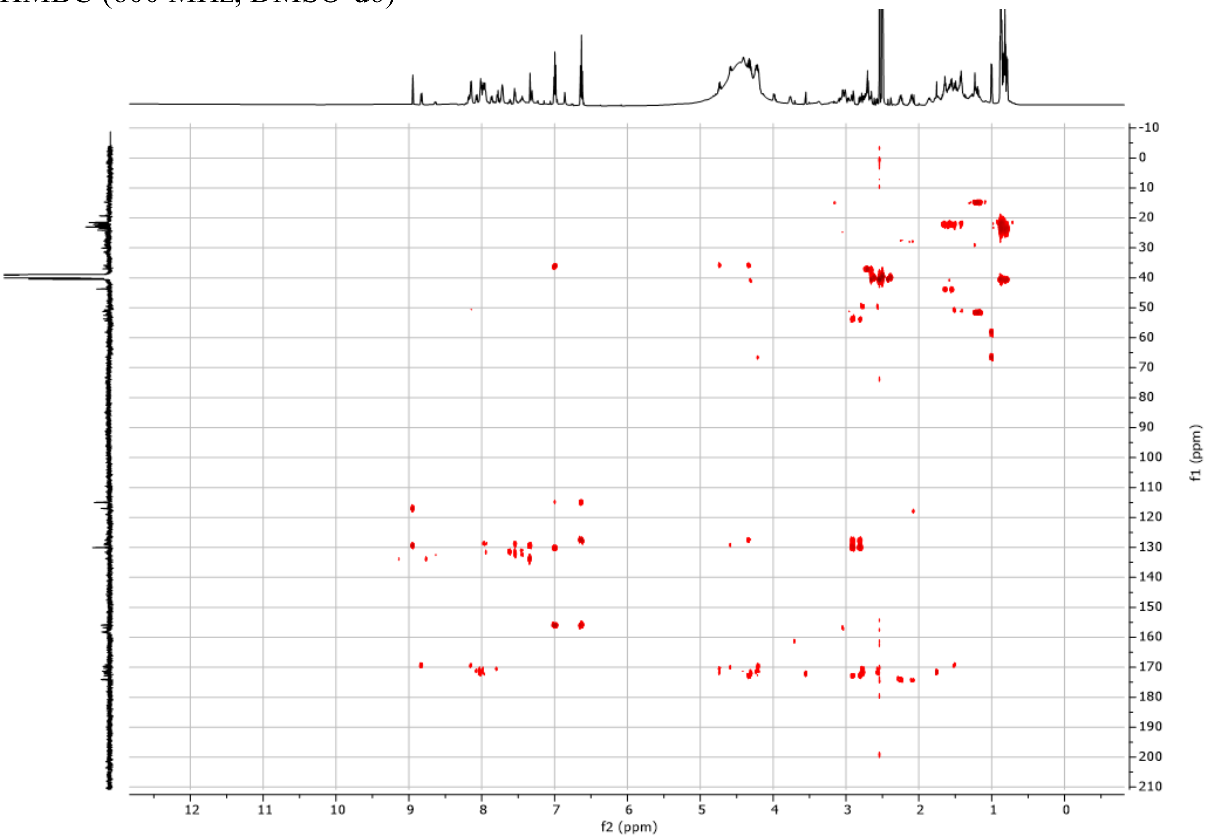

TOCSY (600 MHz, DMSO-d6)

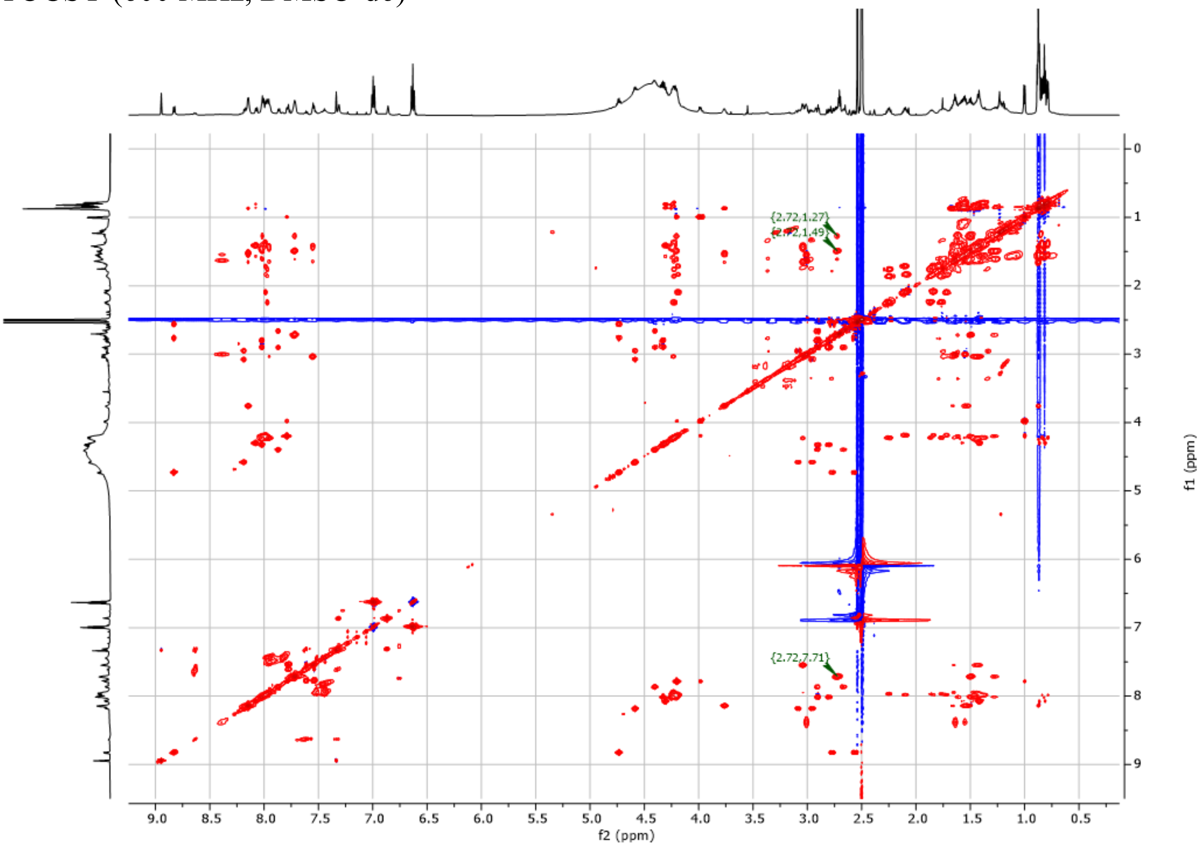

[^{3}H]11C (with neat DMSO)

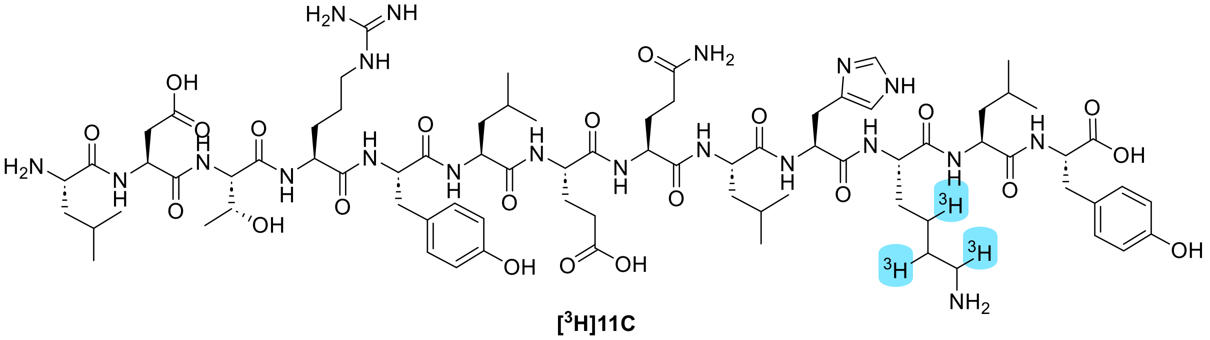

[^{3}H]11C

Chemical Formula: $C_{78}H_{119}T_3N_{20}O_{22}$

Exact Mass: 1696.9289

LC-MS of crude: ca. 47% UV-purity

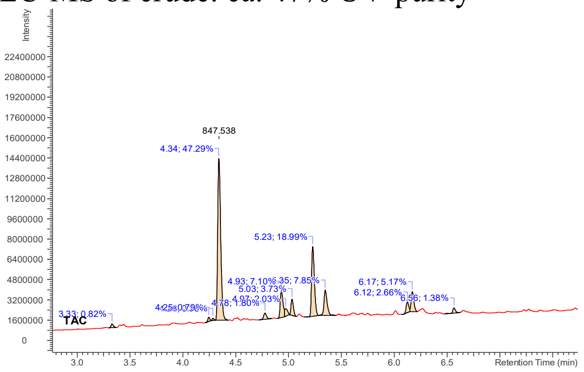

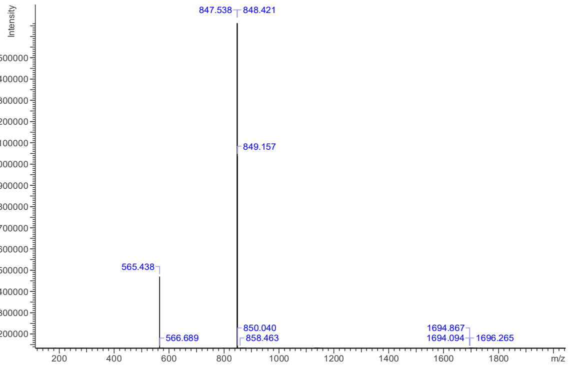

Radio-HPLC of crude: radiochemical purity 31%

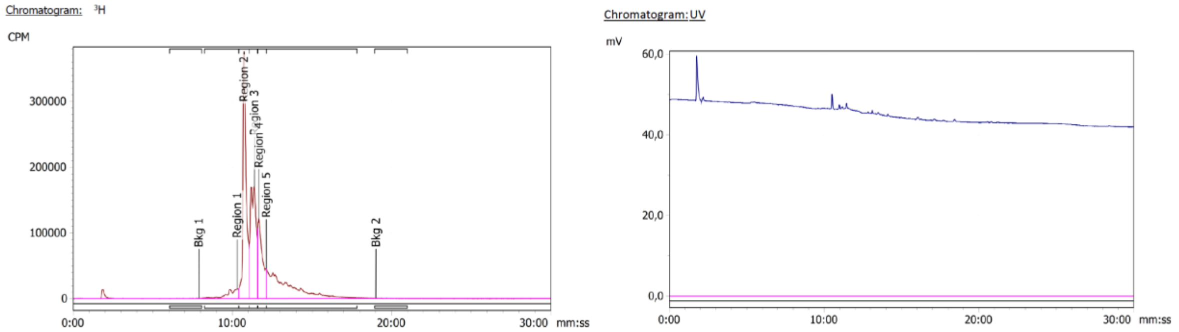

LC-MS of purified product: ca. 47% UV-purity

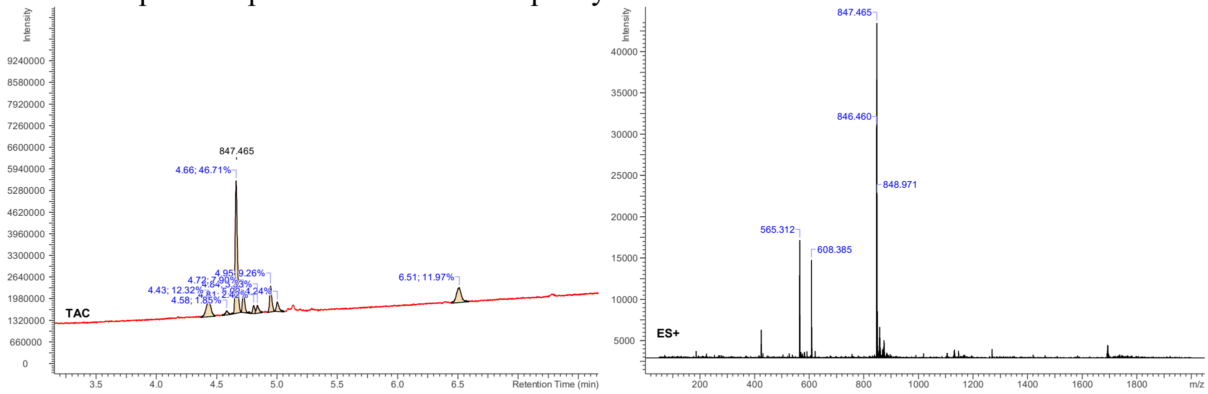

Radio-HPLC of purified product: 87% radiochemical purity

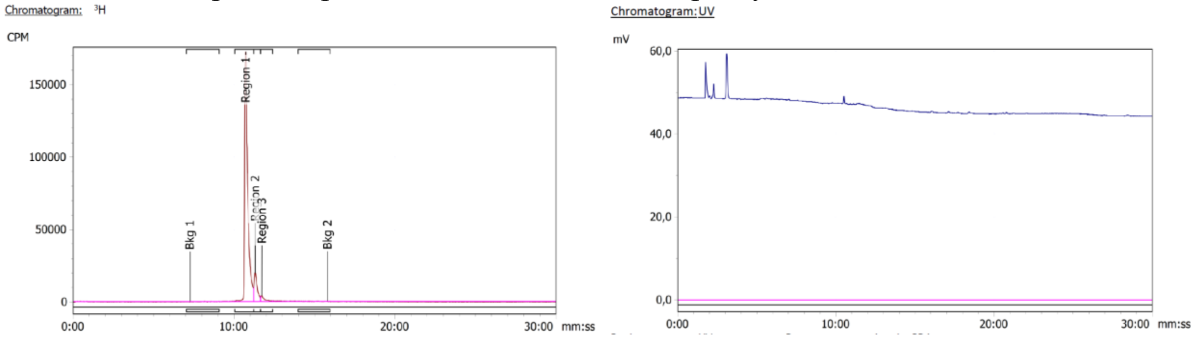

Isotope distribution comparison: Predicted non-labeled vs labeled peptide, ca. 0.84 ^{3}H/molecule

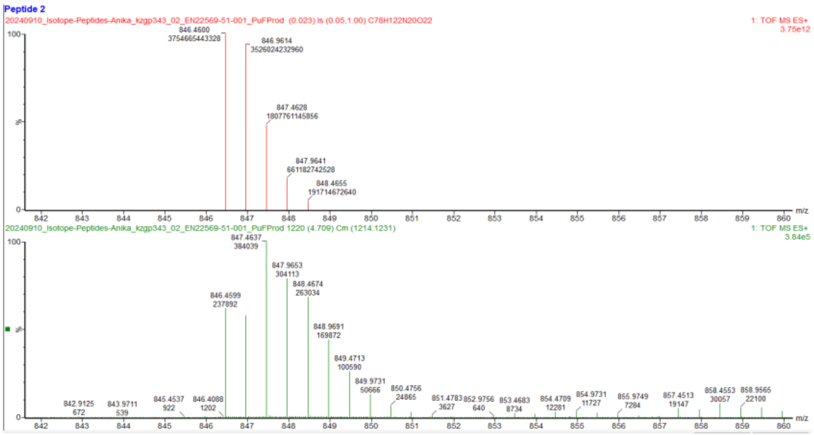

Results Relative amounts [%]	
unlabeled	36.0
0.5-label	-0.2
1-label	40.9
1.5-label	1.3
2-label	17.1
2.5-label	1.9
3-label	2.9

NMR of purified product: ^{1}H-NMR, ^{3}H-NMR coupled, ^{3}H-NMR decoupled

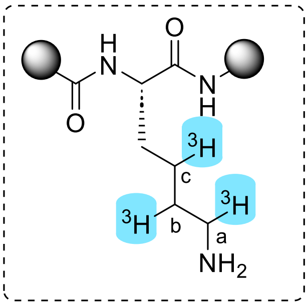

a: ^{3}H at 2.72 ppm (^{3}H-NMR)

b and **c**: ^{3}H at 1.47 ppm and 1.25 ppm (^{3}H-NMR)

^{1}H-NMR (500 MHz, DMSO-d6)

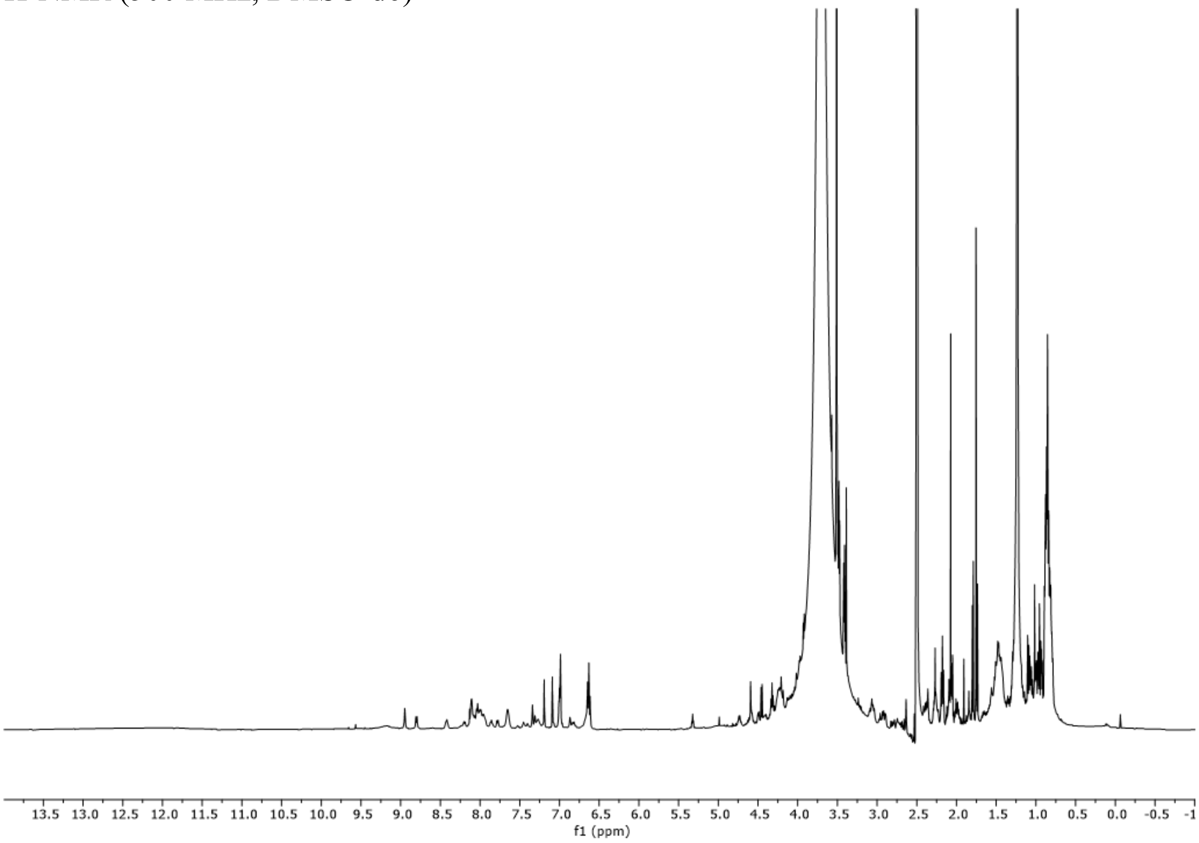

^{3}H-NMR coupled (533 MHz, DMSO-d6)

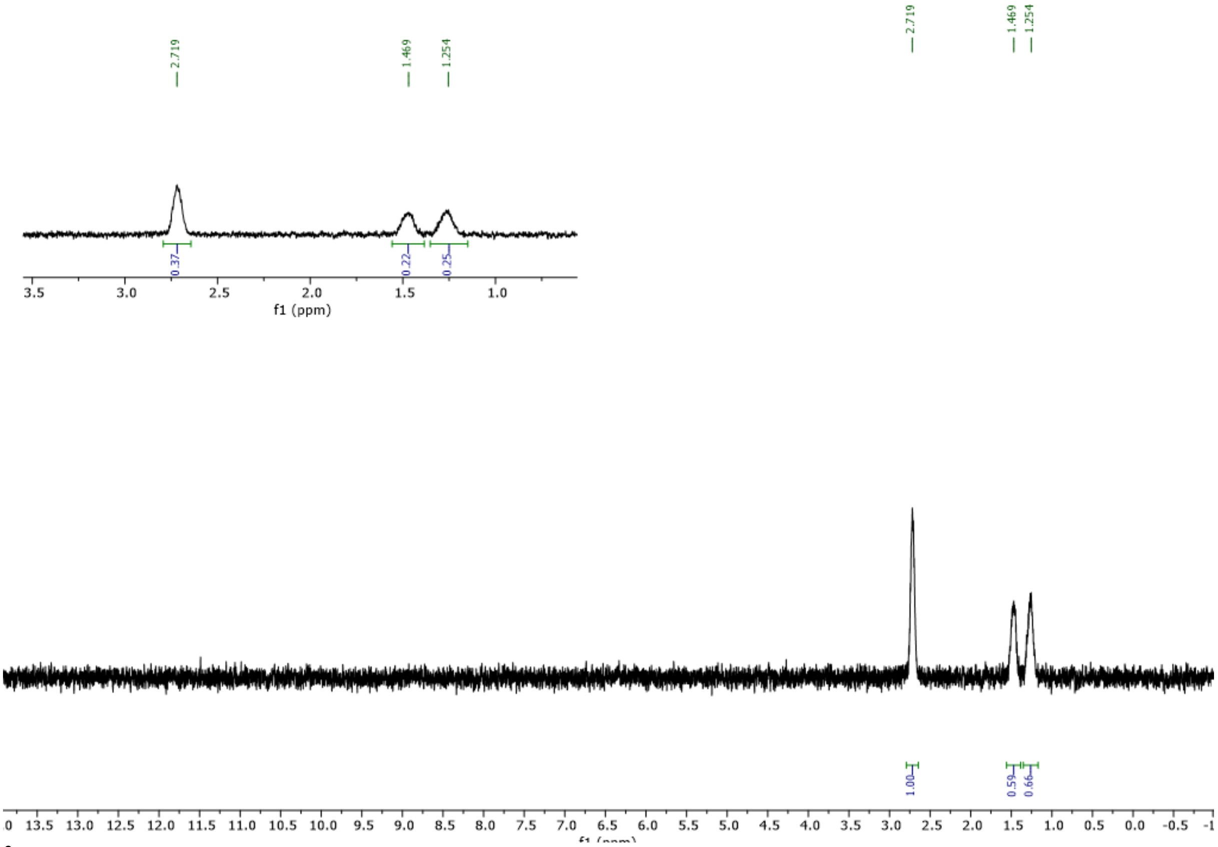

^{3}H-NMR decoupled (533 MHz, DMSO-d6)

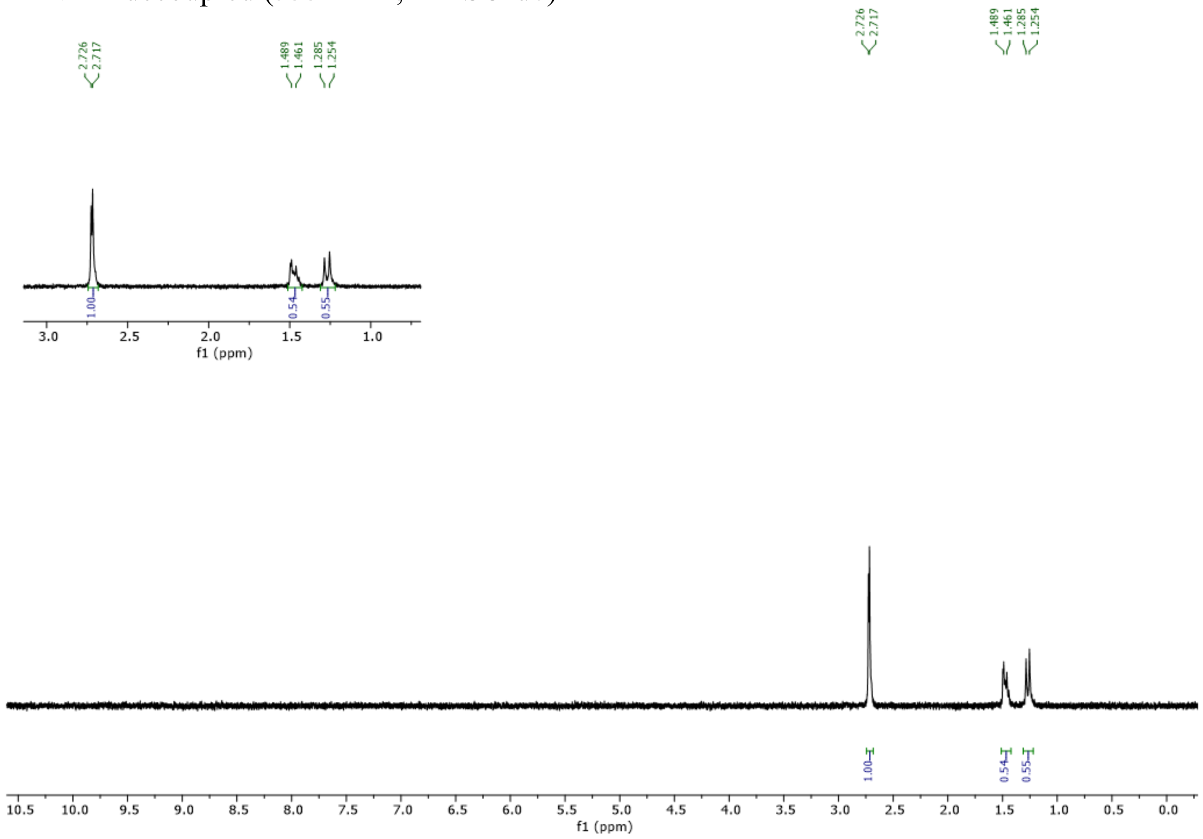

[¹⁴C]11C (with neat DMSO)

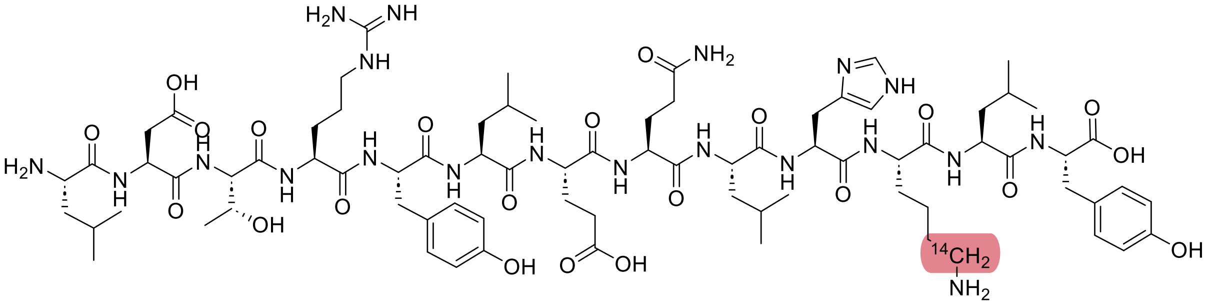

[¹⁴C]11C

Chemical Formula: $C_{77}{}^{14}CH_{122}N_{20}O_{22}$

Exact Mass: 1692.9075

LC-MS of crude: ca. 60% UV-purity

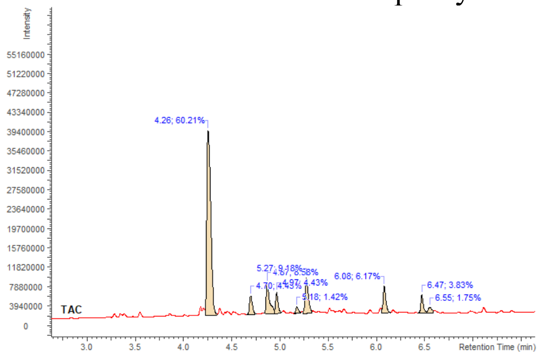

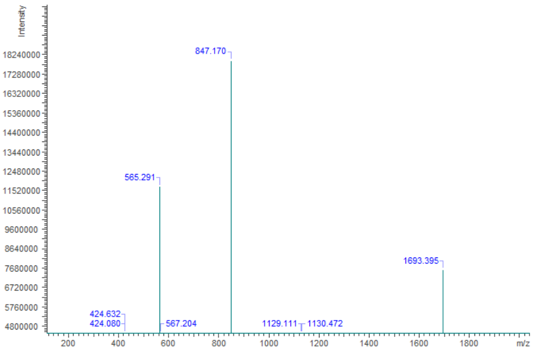

Radio-HPLC of crude: radiochemical purity 48%

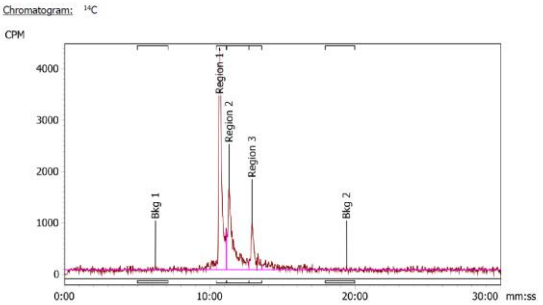

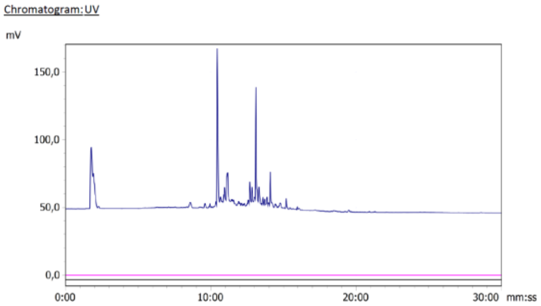

LC-MS of purified product: ca. 96% UV-purity

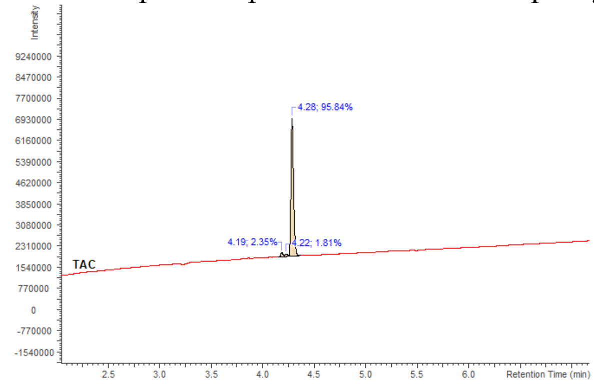

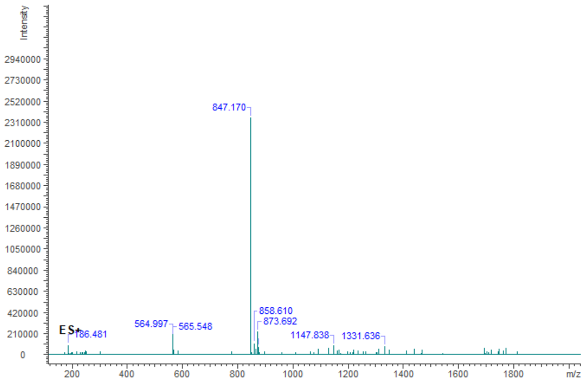

Radio-HPLC of purified product: >99% radiochemical purity

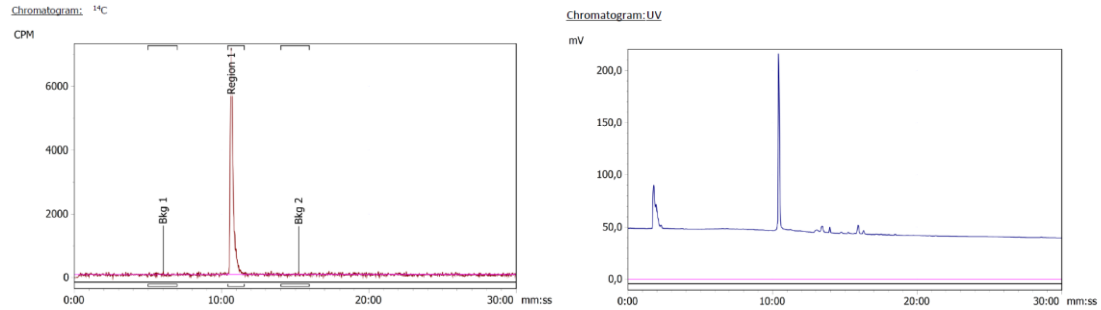

Isotope distribution comparison: Predicted non-labeled vs labeled peptide, ca. 0.05 ^{14}C/molecule incorporation

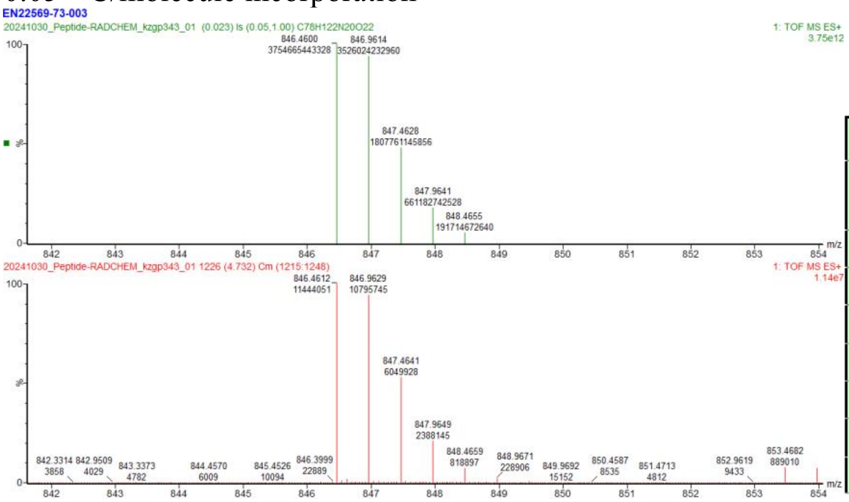

Results	
Relative amounts [%]	
unlabeled	95.4
0.5-label	0.5
1-label	4.1
1.5-label	-0.9
2-label	0.8
2.5-label	0.9
3-label	-0.7

NMR of purified product: ^{1}H-NMR, ^{13}C-NMR, HSQC

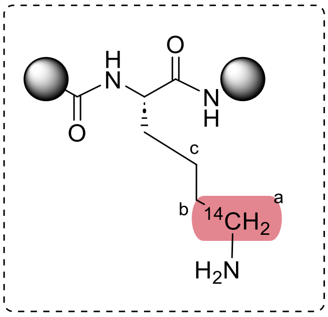

^{1}H-NMR (500 MHz, DMSO-d6)

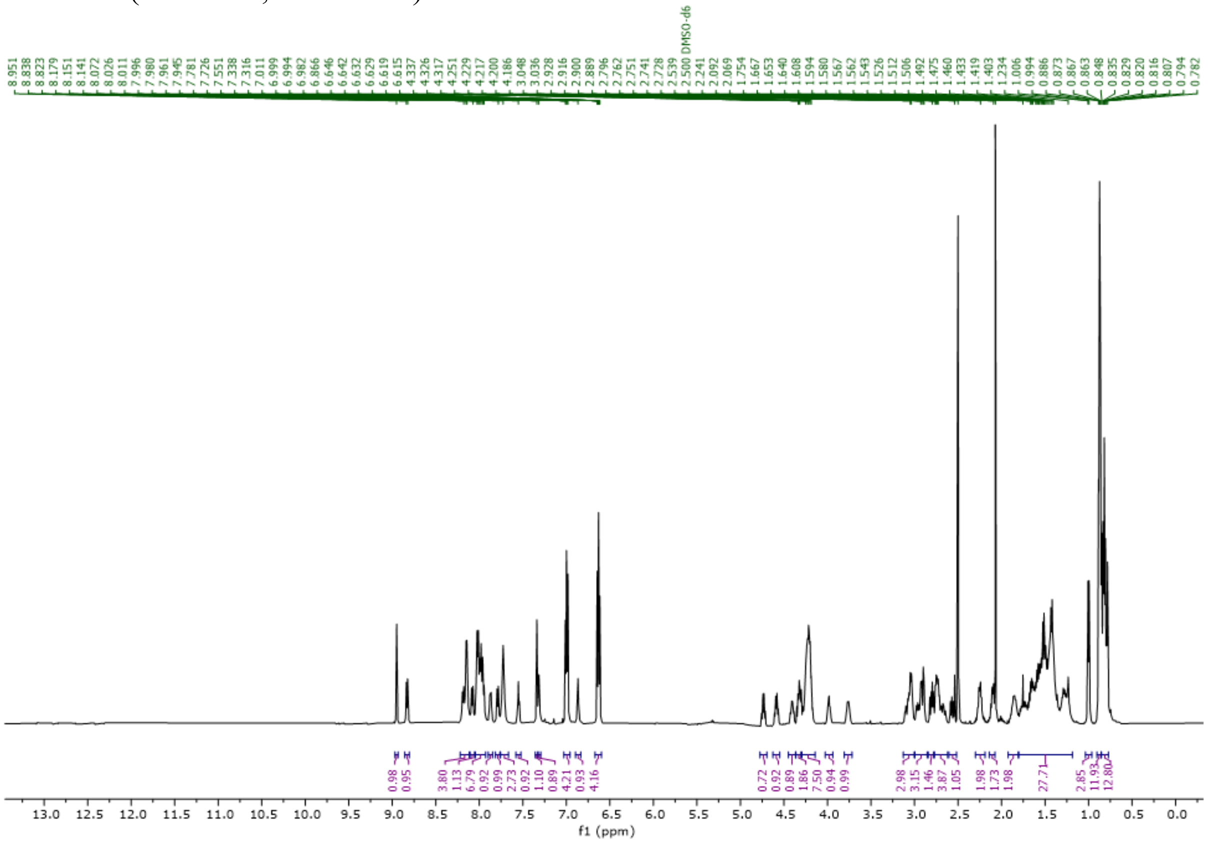

^{13}C-NMR (126 MHz, DMSO-d6)

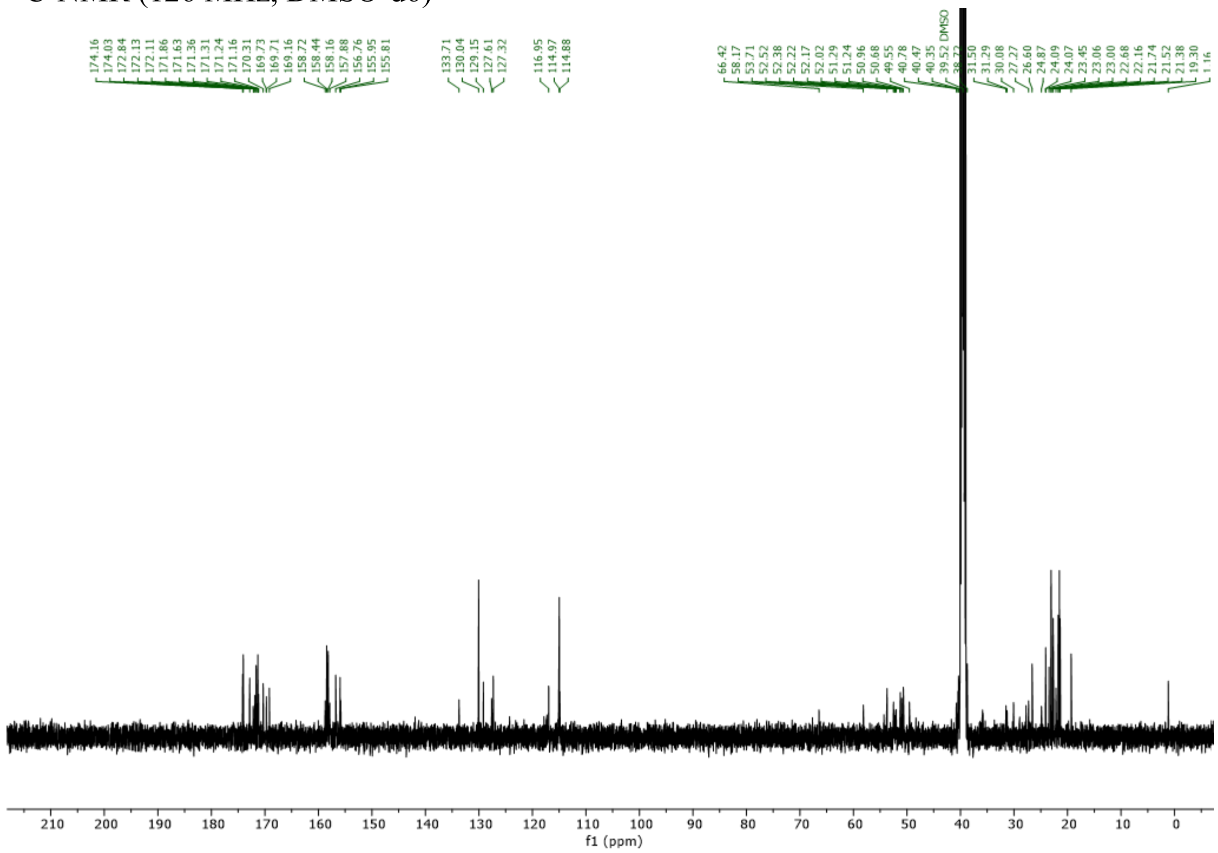

HSQC (500 MHz, DMSO-d6)

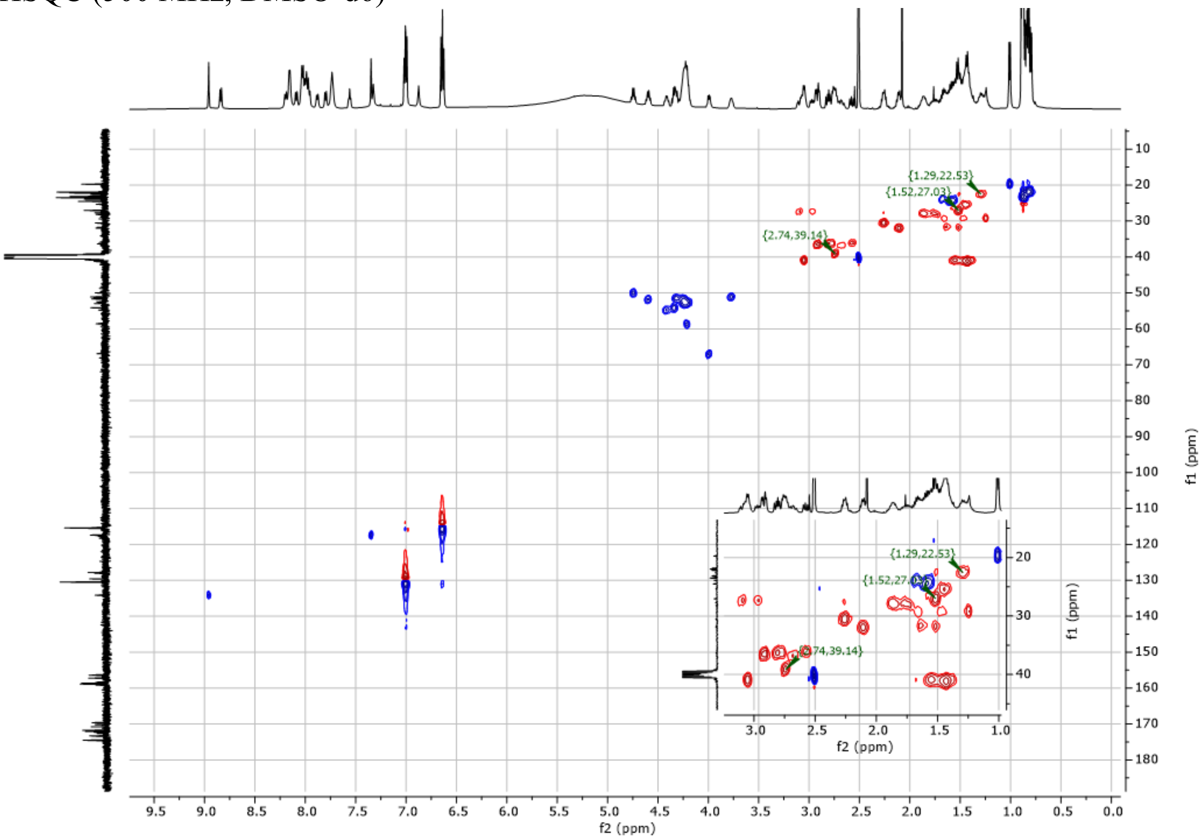

10.2.12 12Ca and 12Cb (semaglutide analogs)

[13C]12Cb (with BIPHEPHOS)

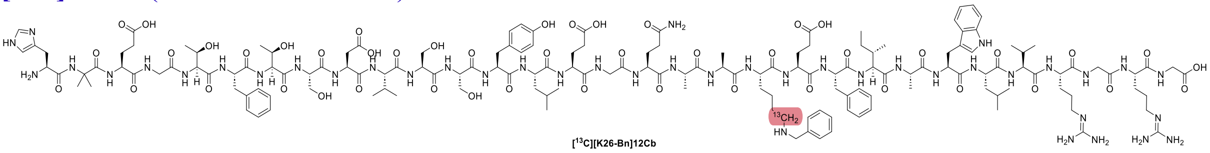

[13C][K26-Bn]12Cb
Chemical Formula: $C_{158}{}^{13}CH_{236}N_{42}O_{47}$
Exact Mass: 3486.7402

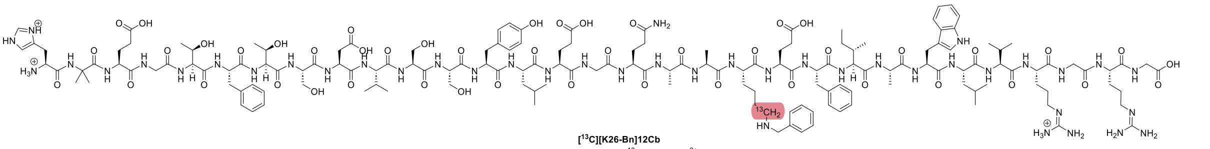

[13C][K26-Bn]12Cb
Chemical Formula: $C_{158}{}^{13}CH_{239}N_{42}O_{47}{}^{3+}$
Exact Mass: 3489.7620

m/z: 1163.5885 (100.0%), 1163.9229 (84.9%), 1163.2540 (58.5%), 1164.2574 (47.8%), 1164.5918 (20.0%), 1163.9208 (15.3%), 1164.2553 (13.0%), 1164.2565 (9.7%), 1163.5864 (9.0%), 1164.5910 (8.2%), 1164.5897 (7.3%), 1164.9263 (6.7%), 1163.9221 (5.7%), 1164.9254 (4.6%), 1164.9242 (3.1%), 1163.9239 (2.7%), 1164.2583 (2.3%), 1165.2599 (1.9%), 1165.2607 (1.8%), 1163.9232 (1.8%), 1163.5894 (1.6%), 1164.2577 (1.5%), 1164.5889 (1.5%), 1164.5928 (1.3%), 1164.9233 (1.3%), 1164.2532 (1.1%), 1163.5888 (1.0%), 1165.2586 (1.0%), 1164.5876 (1.0%)

Comparable with **[13C]12Cb** using 6-DPPon as ligand, but more selective isomer formation
LC-MS of crude: ca. 16% UV-purity (middle: XIC)

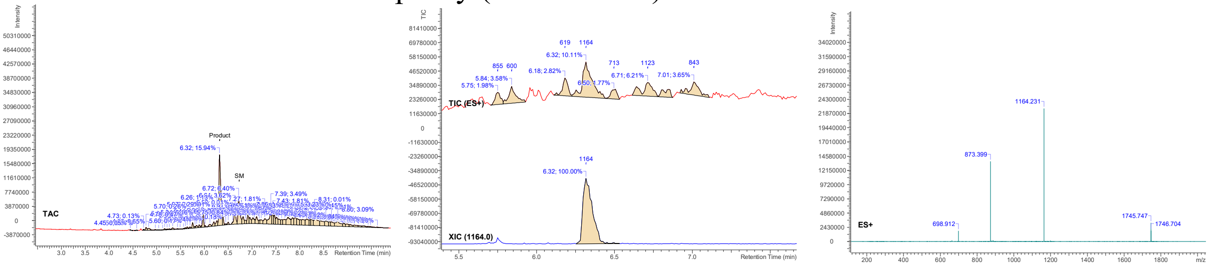

LC-MS of purified product: ca. 93% UV-purity

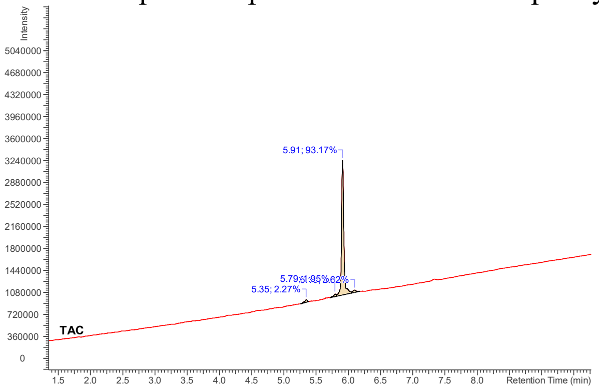

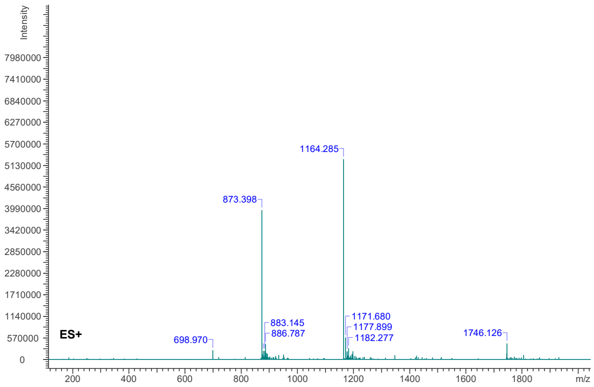

Isotope distribution comparison: Predicted non-labeled vs labeled peptide, ca. +0.99 ^{13}C/molecule incorporation (comparison to predicted distribution spectrum seems to contain some error, see negative values)

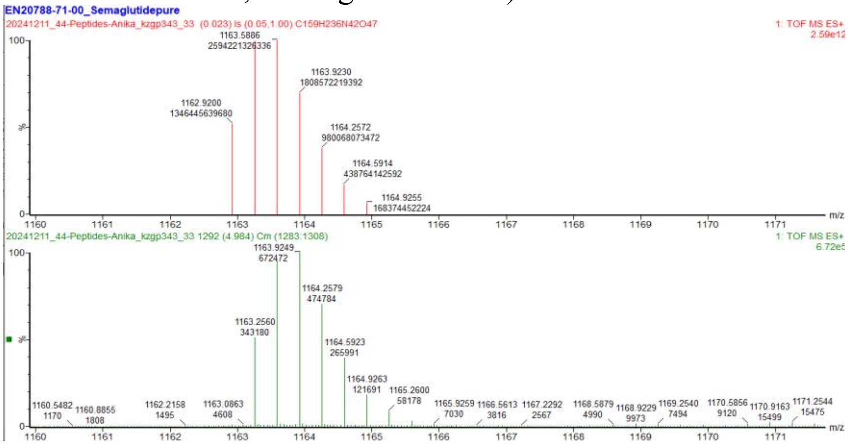

Results	
Relative amounts [%]	
unlabeled	1.2
1-label	97.3
2-label	-2.9
3-label	11.6
4-label	-10.5
5-label	7.6
6-label	-4.4

NMR not given for this batch, see for **[^{13}C]12Cb** using 6-DPPon

[^{13}C]12Cb (with 6-DPPon)

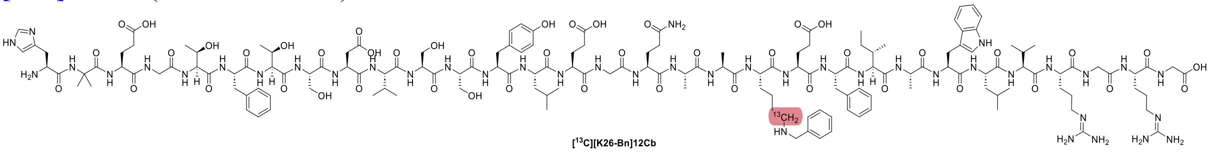

[^{13}C][K26-Bn]12Cb
Chemical Formula: $C_{158}{}^{13}CH_{236}N_{42}O_{47}$
Exact Mass: 3486.7402

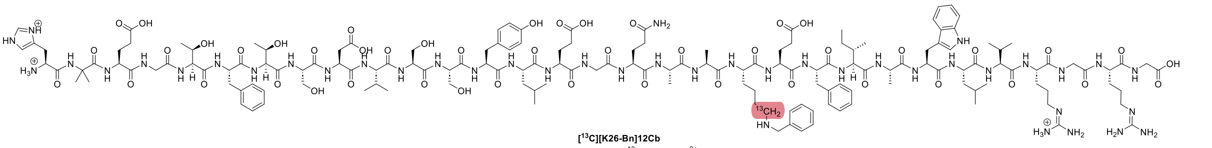

[^{13}C][K26-Bn]12Cb
Chemical Formula: $C_{158}{}^{13}CH_{239}N_{42}O_{47}{}^{3+}$
Exact Mass: 3489.7620
m/z: 1163.5885 (100.0%), 1163.9229 (84.9%), 1163.2540 (58.5%), 1164.2574 (47.8%), 1164.5918 (20.0%), 1163.9208 (15.3%), 1164.2553 (13.0%), 1164.2565 (9.7%), 1163.5864 (9.0%), 1164.5910 (8.2%), 1164.5897 (7.3%), 1164.9263 (6.7%), 1163.9221 (5.7%), 1164.9254 (4.6%), 1164.9242 (3.1%), 1163.9239 (2.7%), 1164.2583 (2.3%), 1165.2599 (1.9%), 1165.2607 (1.8%), 1163.9232 (1.8%), 1163.5894 (1.6%), 1164.2577 (1.5%), 1164.5889 (1.5%), 1164.5928 (1.3%), 1164.9233 (1.3%), 1164.2532 (1.1%), 1163.5888 (1.0%), 1165.2586 (1.0%), 1164.5876 (1.0%)

LC-MS of crude: ca. 14% UV-purity (middle: XIC)

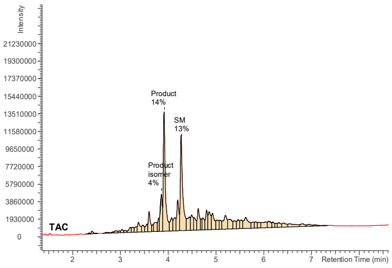

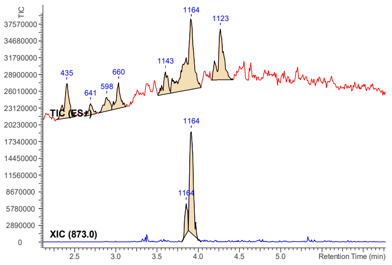

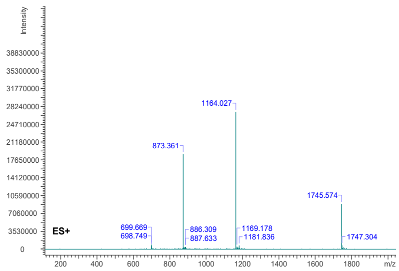

LC-MS of purified product: ca. 78% UV-purity

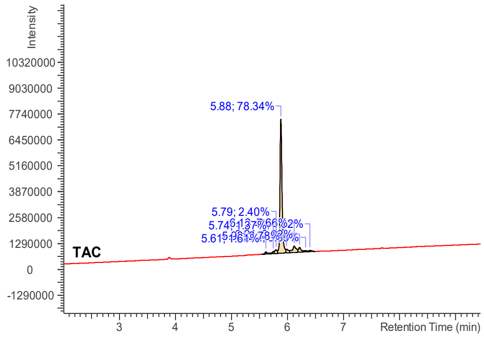

Isotope distribution comparison: Predicted non-labeled vs labeled peptide, ca. +0.99 ^{13}C/molecule incorporation (comparison to predicted distribution spectrum seems to contain some error)

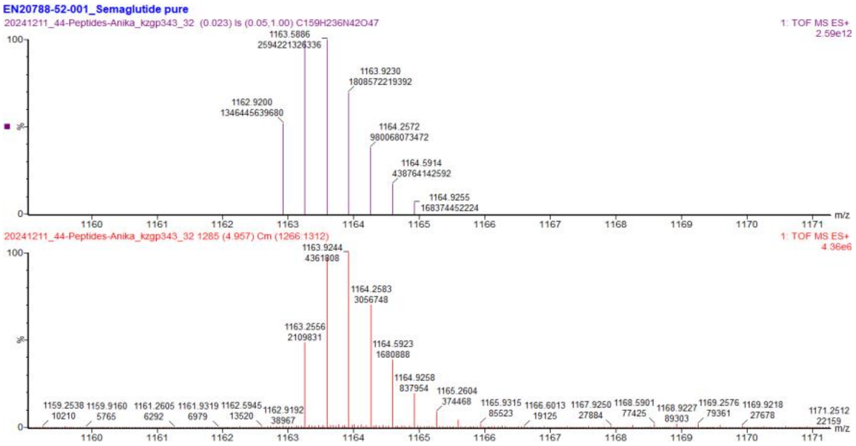

Results Relative amounts [%]	
unlabeled	1.7
1-label	88.1
2-label	11.0
3-label	-4.3
4-label	-0.2
5-label	2.0
6-label	1.7

NMR of purified product: ^{1}H-NMR, ^{13}C-NMR, HSQC, HMBC

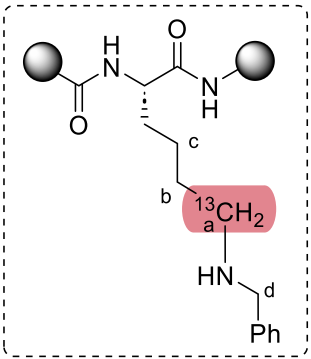

a: ^{13}C at 46.49 ppm, ^{1}H medium at 2.86 ppm (HSQC), split protons not visible in HMBC
b and **c**: ^{1}H at 1.57 ppm and 1.29/1.24 ppm (HMBC)
d: ^{1}H at 4.13 ppm
NH$_2$: ^{1}H at 8.79 ppm (HSQC artifact presumably, but not visible in HMBC)

^{1}H-NMR (500 MHz, DMSO-d6)

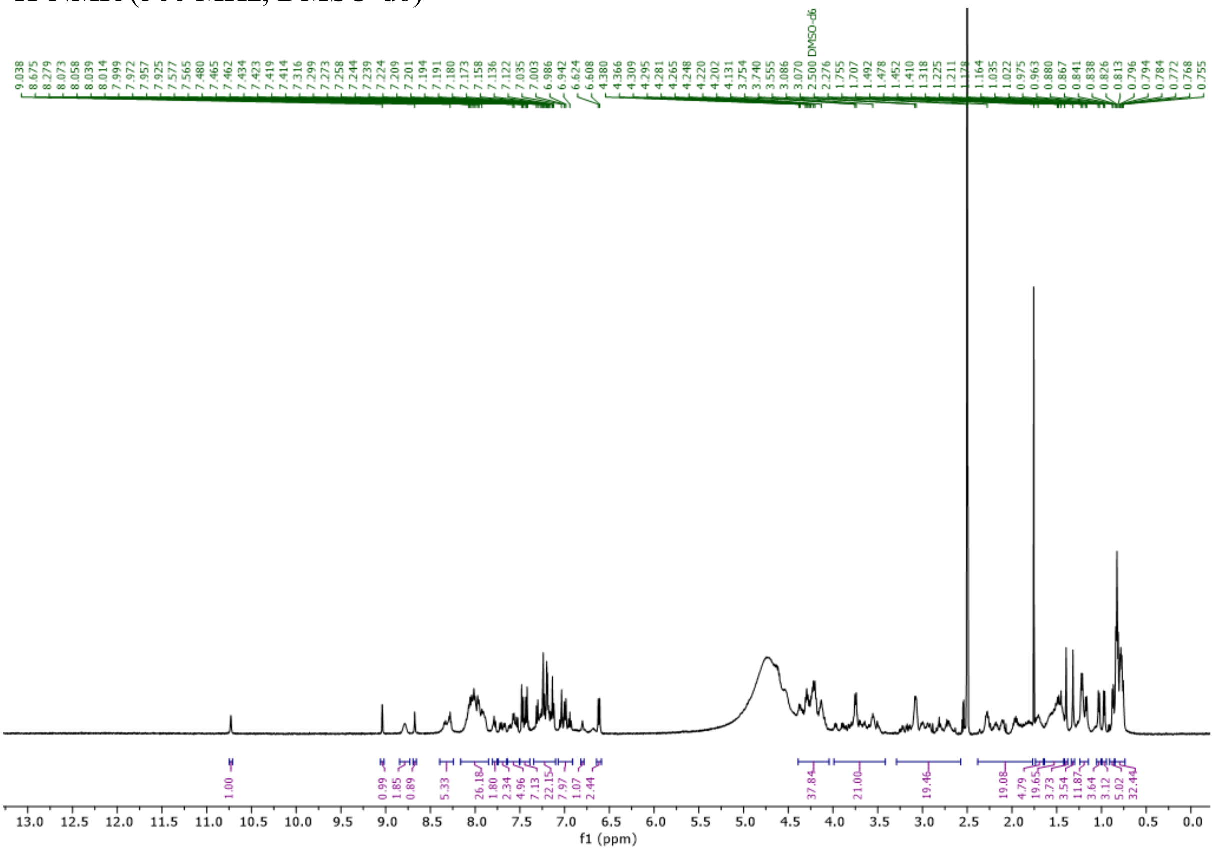

^{13}C-NMR (126 MHz, DMSO-d6)

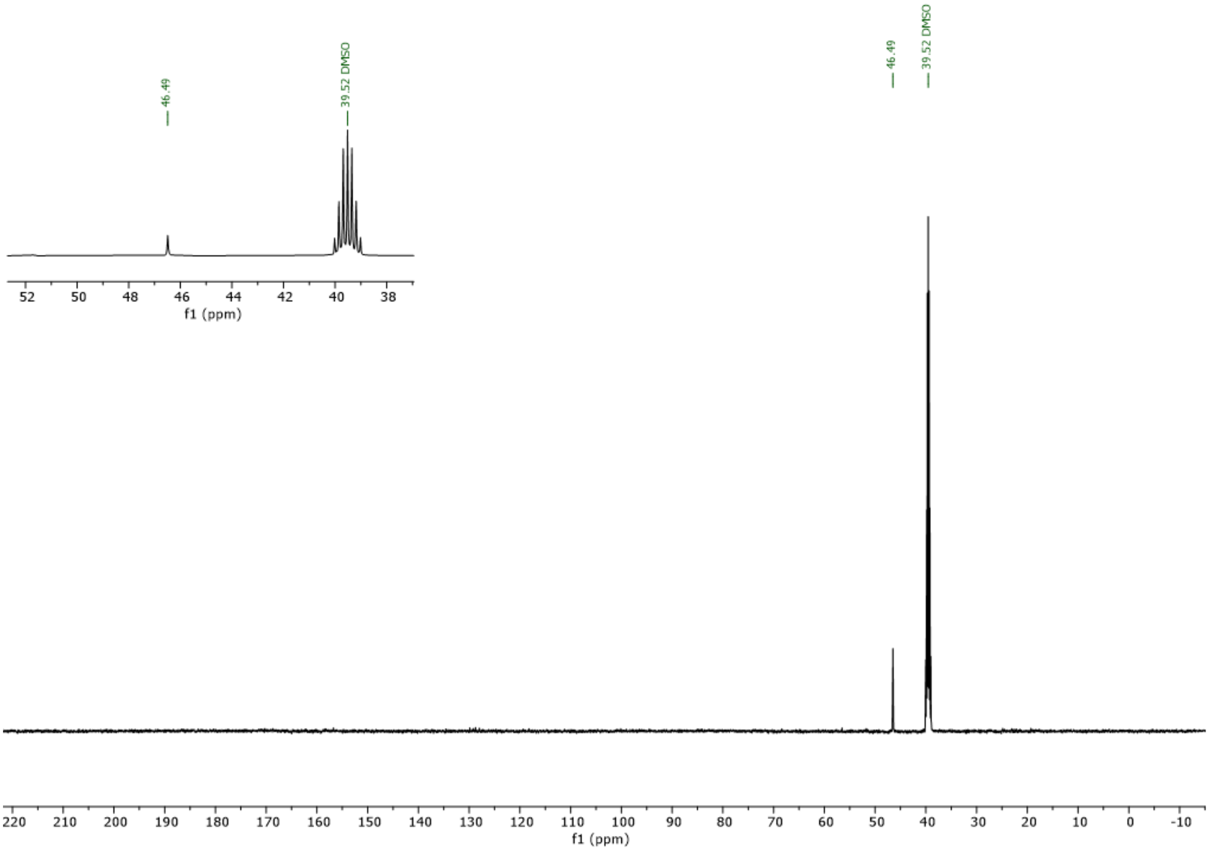

HSQC (500 MHz, DMSO-d6)

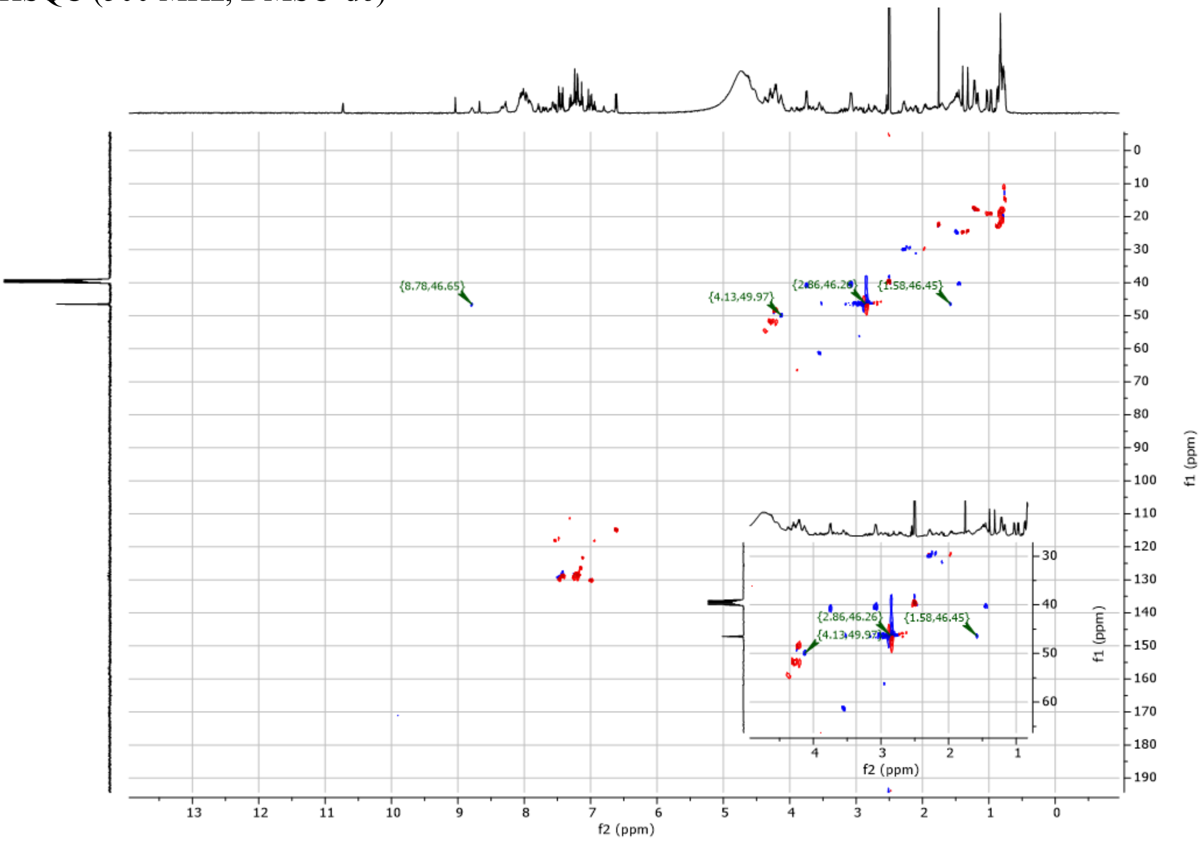

HMBC (500 MHz, DMSO-d6)

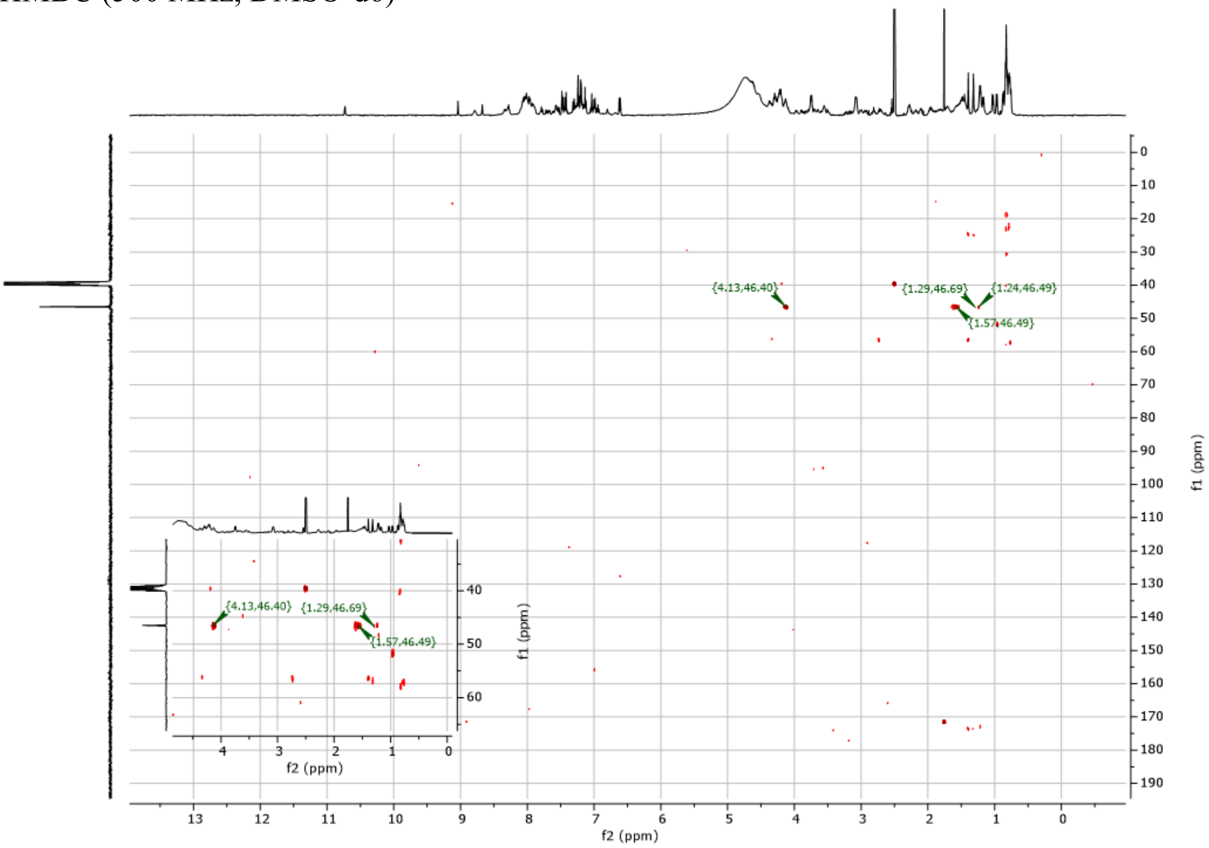

[^{3}H]12C (with BIPHEPHOS)

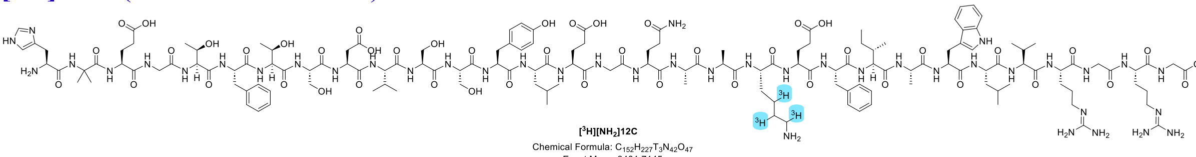

[^{3}H][NH$_2$]12C
Chemical Formula: $C_{152}H_{227}T_3N_{42}O_{47}$
Exact Mass: 3401.7145

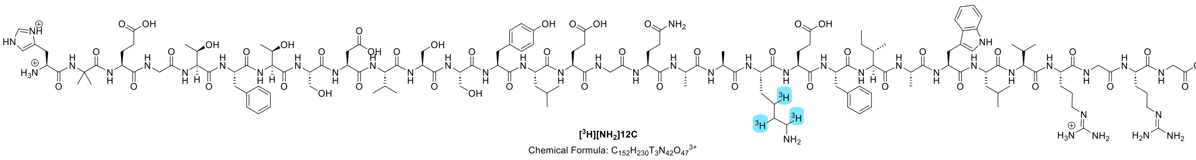

[^{3}H][NH$_2$]12C
Chemical Formula: $C_{152}H_{230}T_3N_{42}O_{47}^{3+}$
Exact Mass: 3404.7364
m/z: 1135.2466 (100.0%), 1135.5810 (81.7%), 1134.9121 (60.8%), 1135.9155 (44.2%), 1136.2499 (17.8%), 1135.5789 (15.3%), 1135.9134 (12.5%), 1135.9147 (9.7%), 1135.2445 (9.3%), 1136.2491 (7.9%), 1136.2478 (6.8%), 1135.5802 (5.9%), 1136.5844 (5.7%), 1136.5836 (4.3%), 1136.5823 (2.7%), 1135.5820 (2.6%), 1135.9165 (2.2%), 1135.5813 (1.8%), 1136.9180 (1.7%), 1135.2476 (1.6%), 1136.9188 (1.5%), 1136.2470 (1.5%), 1135.9158 (1.5%), 1136.5815 (1.2%), 1136.2509 (1.2%), 1135.9113 (1.1%), 1135.2469 (1.1%)

LC-MS of crude: reductive amination less efficient with TrtNH$_2$, leads to decomposition, only ca. 5% UV-purity

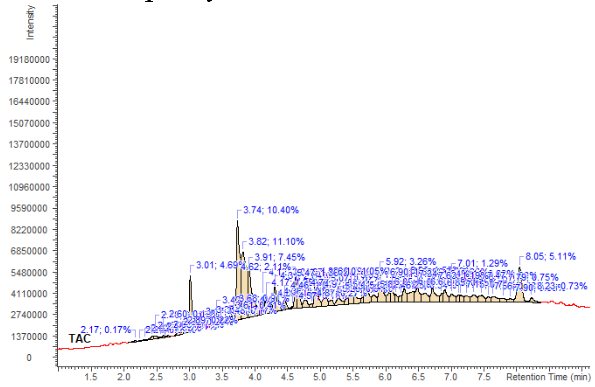

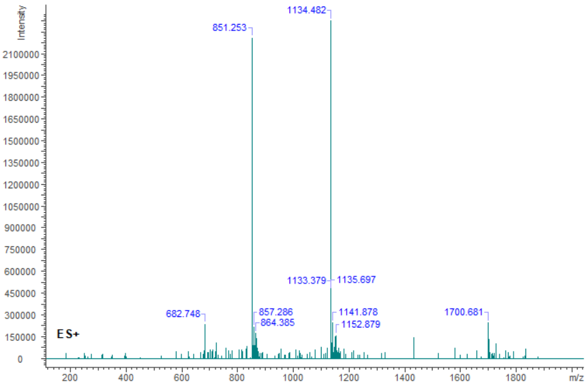

Radio-HPLC of crude: no clear signal, not visible

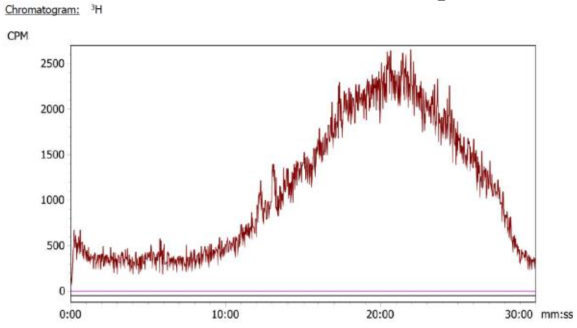

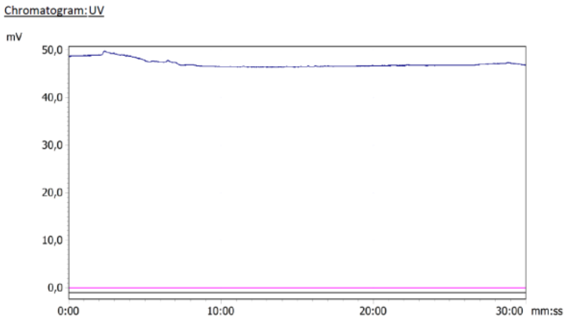

Isotope distribution comparison: Predicted non-labeled vs labeled peptide, ca. +0.68 ^{3}H/molecule incorporation

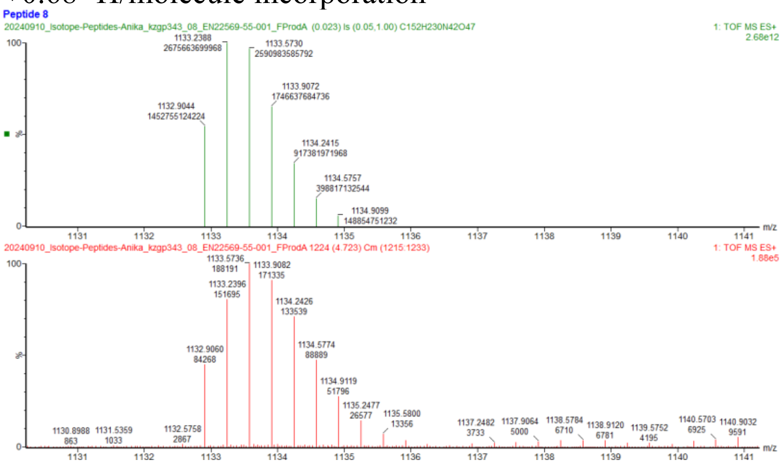

Results	
Relative amounts [%]	
unlabeled	63.0
0.5-label	-2.6
1-label	33.2
1.5-label	-4.1
2-label	11.6
2.5-label	-3.1
3-label	2.0

LC-MS of purified product (two LC-MS runs shown): ca. 45-54% UV-purity

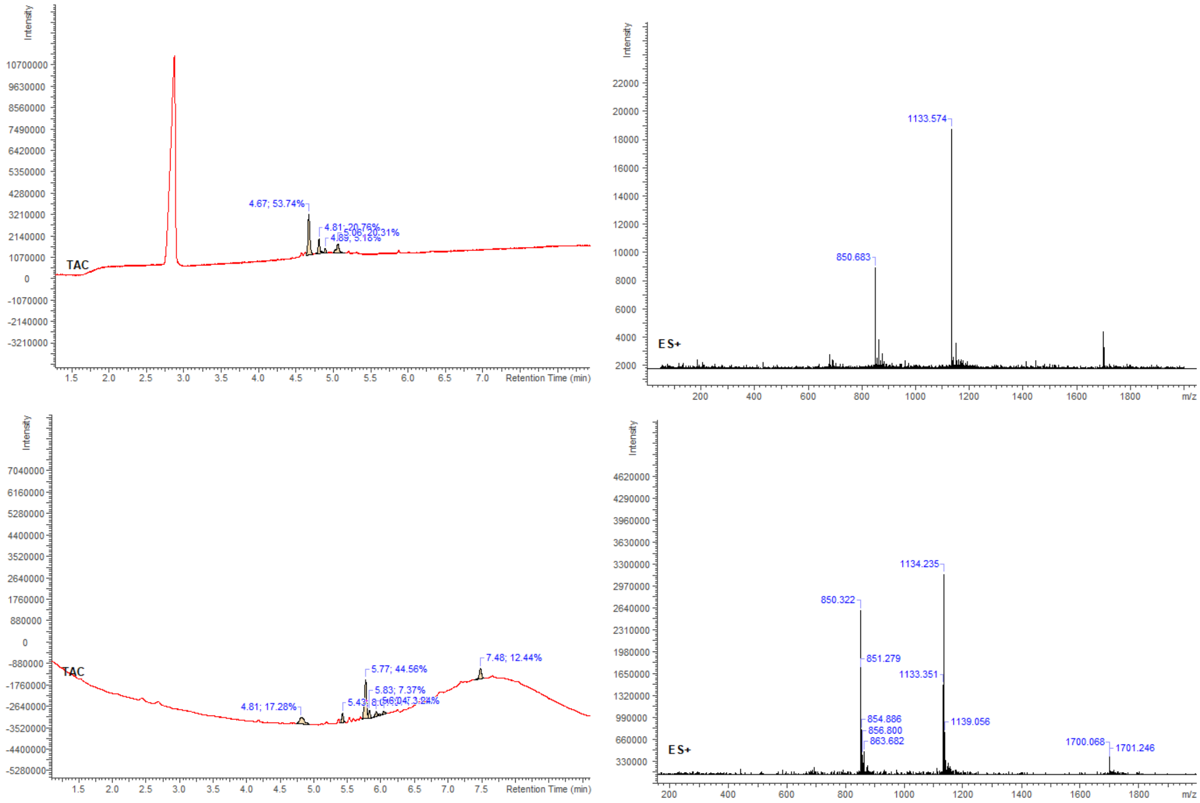

Radio-HPLC of purified product: 55% radiochemical purity

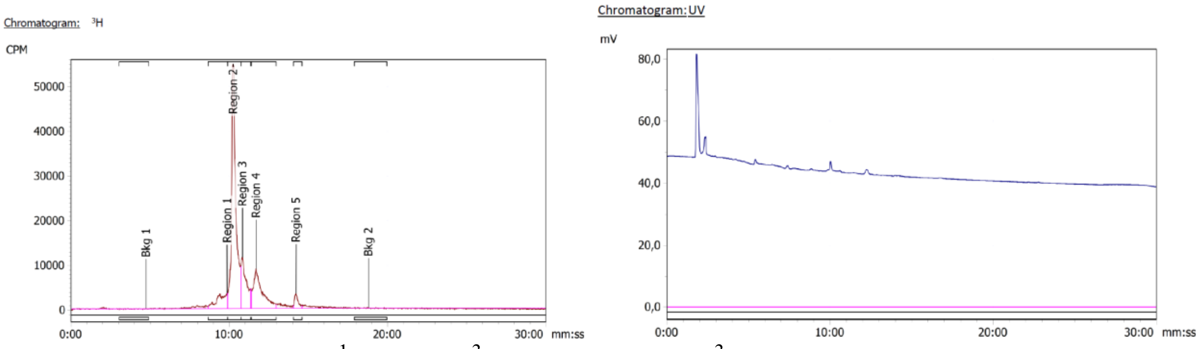

NMR of purified product: ^{1}H-NMR, ^{3}H-NMR coupled, ^{3}H-NMR decoupled

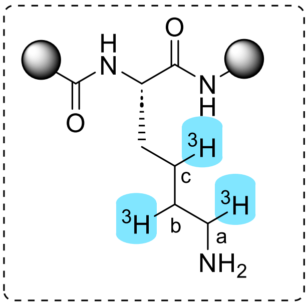

a: ^{3}H at 2.72 ppm (^{3}H-NMR)
b and **c**: ^{3}H at 1.47 ppm and 1.23 ppm (^{3}H-NMR)

^{1}H-NMR (500 MHz, DMSO-d6), impurity at 7.218 ppm, 7.115 ppm, 7.014 ppm.

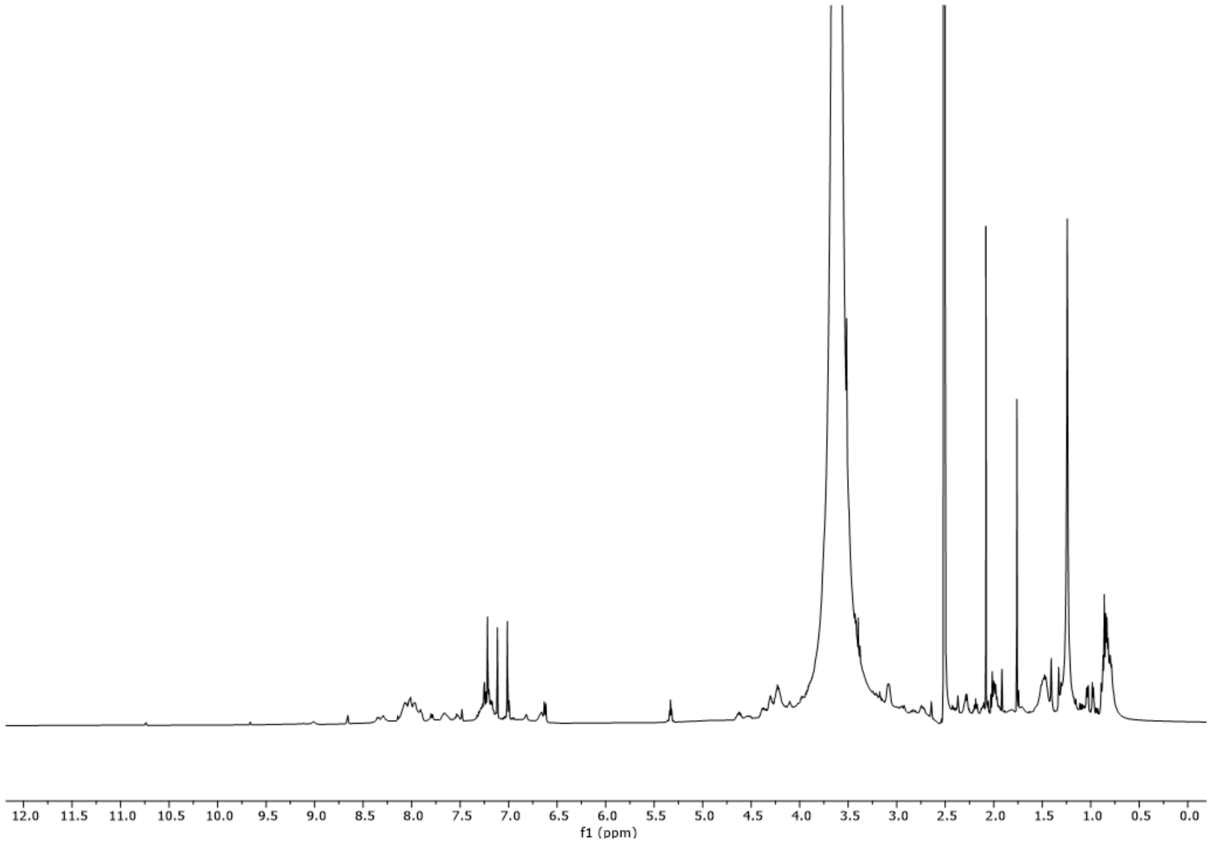

[3]H-NMR coupled (533 MHz, DM SO-d6)

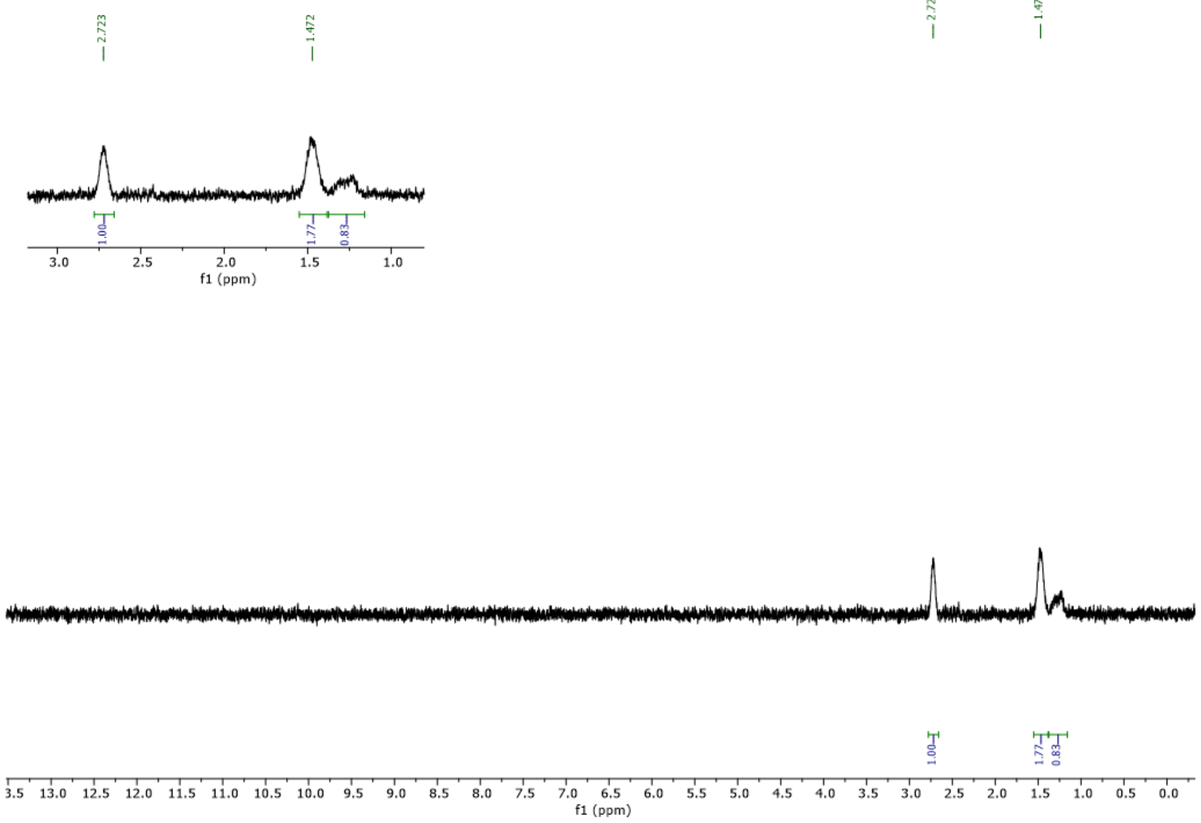

^{3}H-NMR decoupled (533 MHz, DMSO-d6)

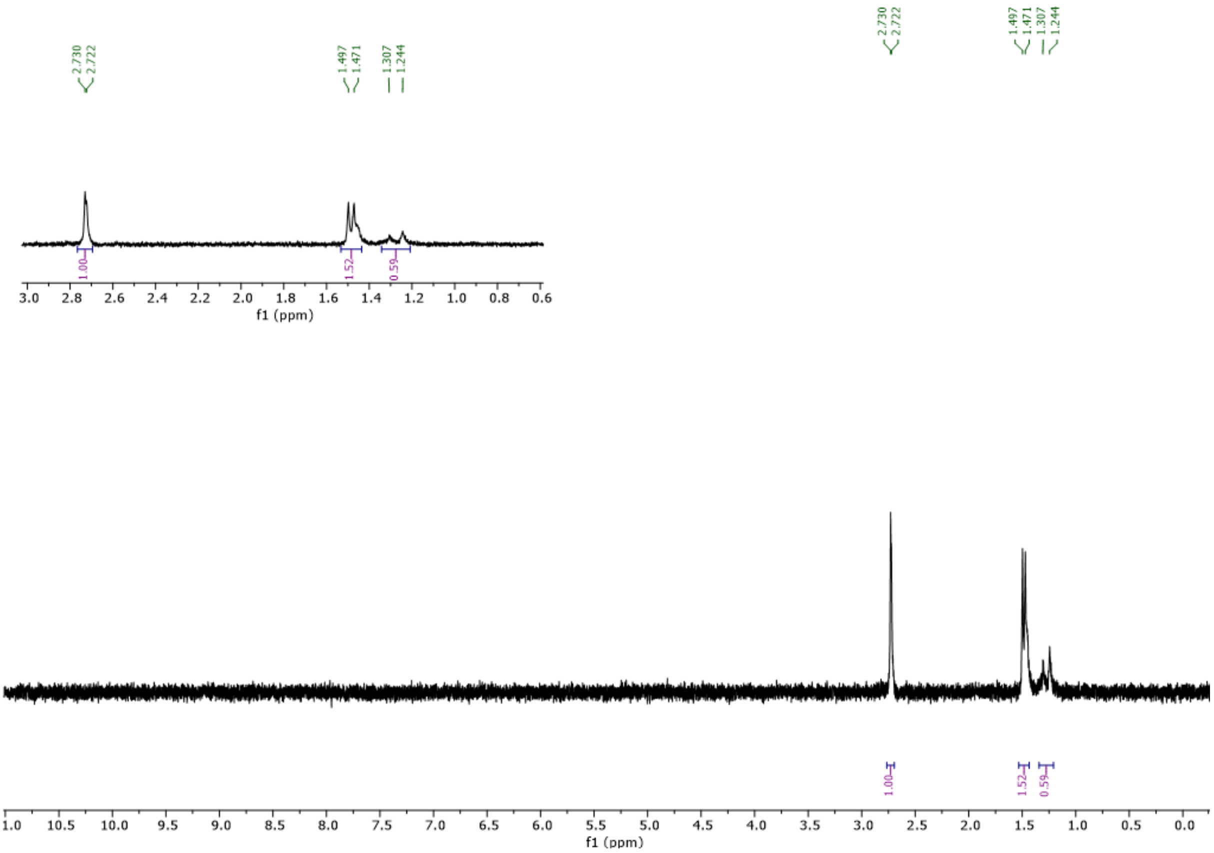

[2H]12C (with BIPHEPHOS)

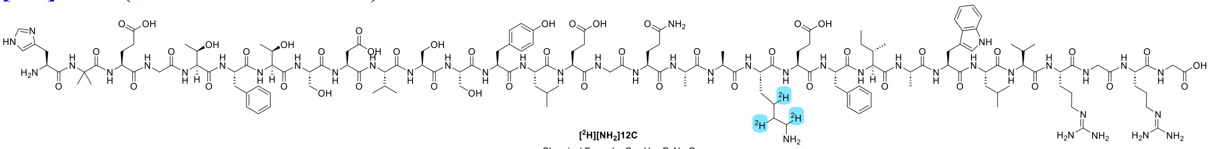

[2H][NH2]12C
Chemical Formula: $C_{152}H_{227}D_3N_{42}O_{47}$
Exact Mass: 3398.7087

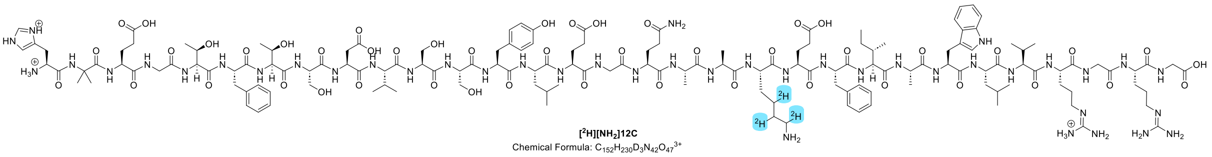

[2H][NH2]12C
Chemical Formula: $C_{152}H_{230}D_3N_{42}O_{47}^{3+}$
Exact Mass: 3401.7305
m/z: 1134.2446 (100.0%), 1134.5791 (81.7%), 1133.9102 (60.8%), 1134.9135 (44.2%), 1135.2480 (17.8%), 1134.5770 (15.3%), 1134.9114 (12.5%), 1134.9127 (9.7%), 1134.2425 (9.3%), 1135.2472 (7.9%), 1135.2459 (6.8%), 1134.5783 (5.9%), 1135.5824 (5.7%), 1135.5816 (4.3%), 1135.5803 (2.7%), 1134.5801 (2.6%), 1134.9145 (2.2%), 1134.5794 (1.8%), 1135.9161 (1.7%), 1134.2456 (1.6%), 1135.9169 (1.5%), 1135.2451 (1.5%), 1134.9138 (1.5%), 1135.5795 (1.2%), 1135.2490 (1.2%), 1134.9093 (1.1%), 1134.2449 (1.1%)

LC-MS of crude: ca. 8% UV-purity, reductive amination less efficient with $TrtNH_2$, leads to decomposition

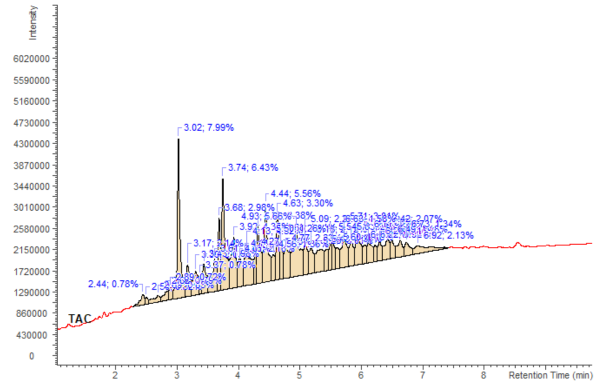

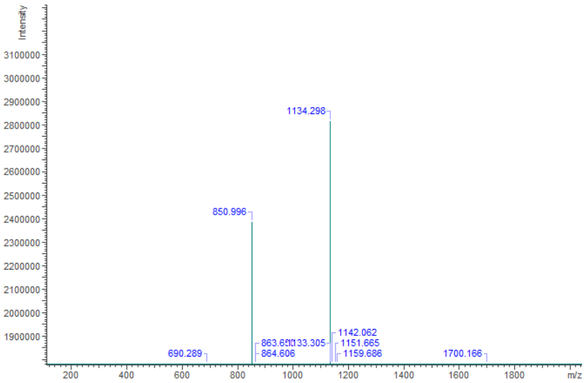

LC-MS of purified product: ca. 82% UV-purity

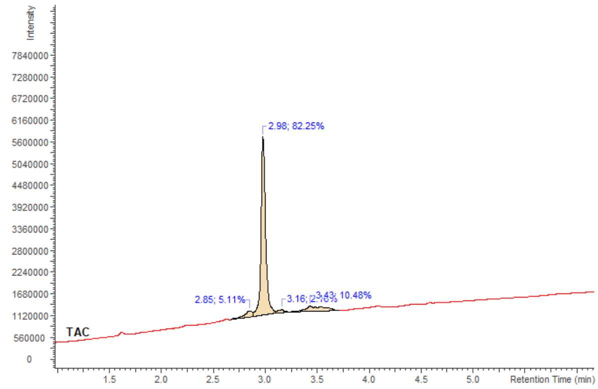

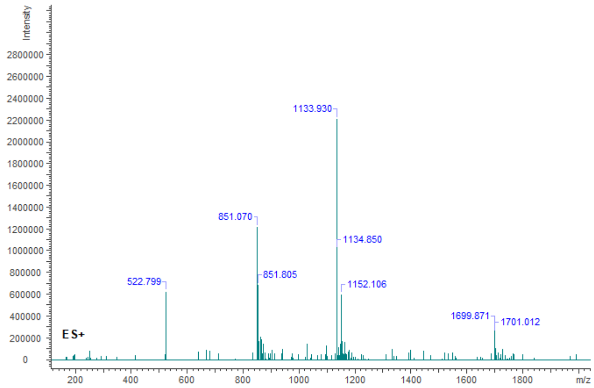

Isotope distribution comparison: Predicted non-labeled vs labeled peptide, +0.50 ^{2}H/molecule incorporation

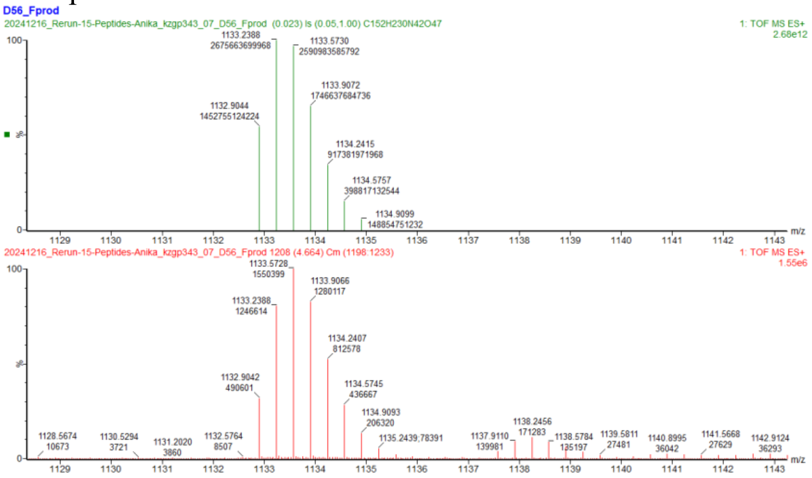

Results	
Relative amounts [%]	
unlabeled	54.9
1-label	38.4
2-label	4.8
3-label	-0.1
4-label	1.7
5-label	0.8
6-label	-0.5

NMR of purified product: ^{1}H-NMR, ^{13}C-NMR, HSQC, HMBC, COSY

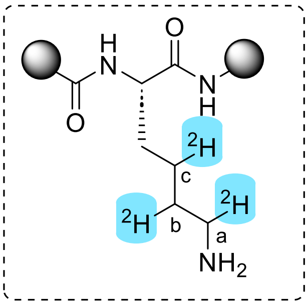

^{1}H-NMR (600 MHz, DMSO-d6), impurity at 7.239 ppm, 7.154 ppm, 7.069 ppm.

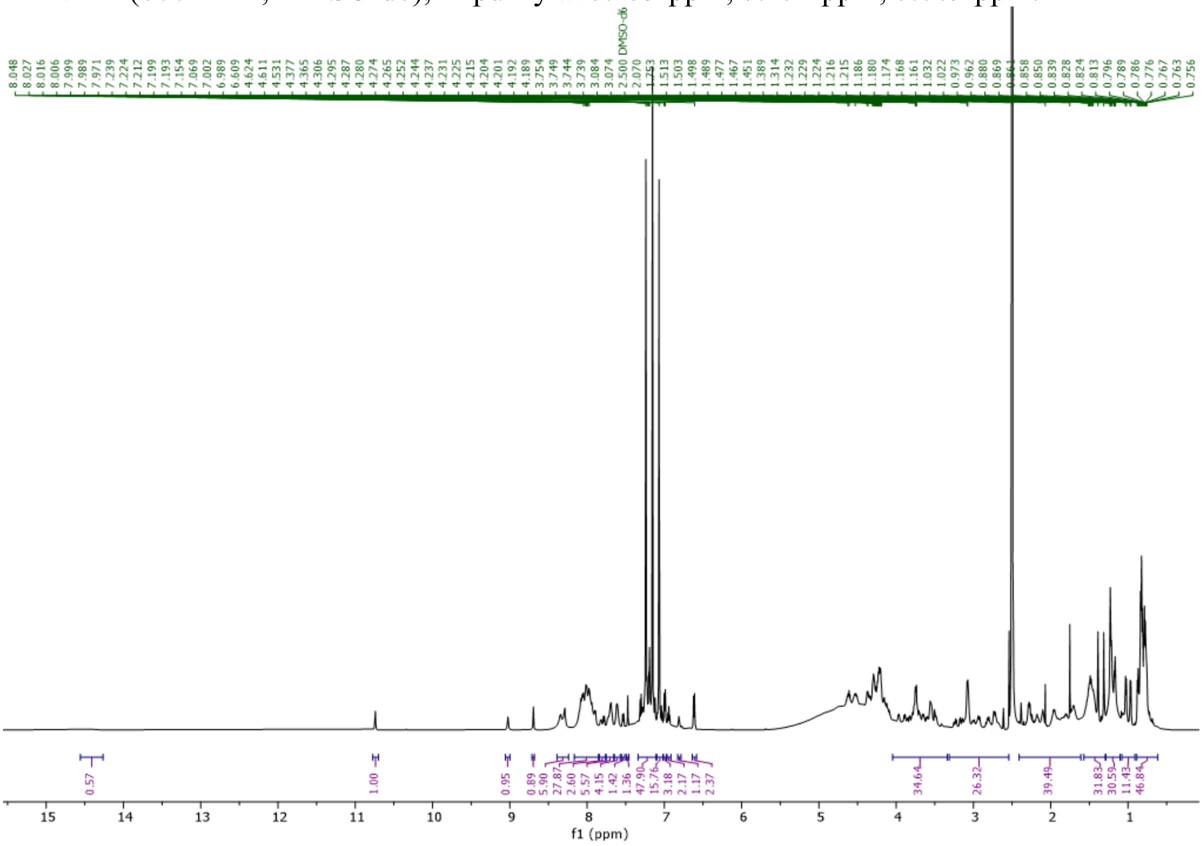

^{13}C-NMR (151MHz, DMSO-d6)

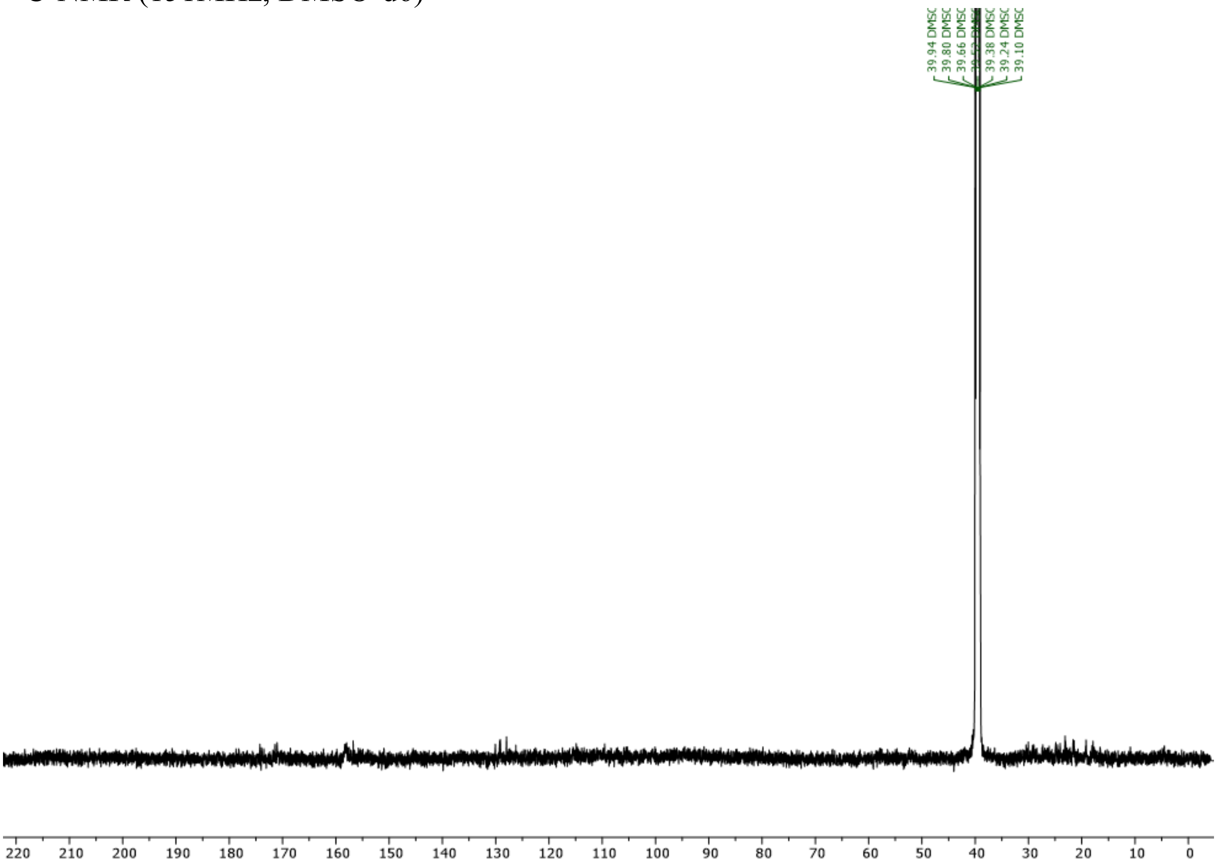

HSQC (600 MHz, DMSO-d6)

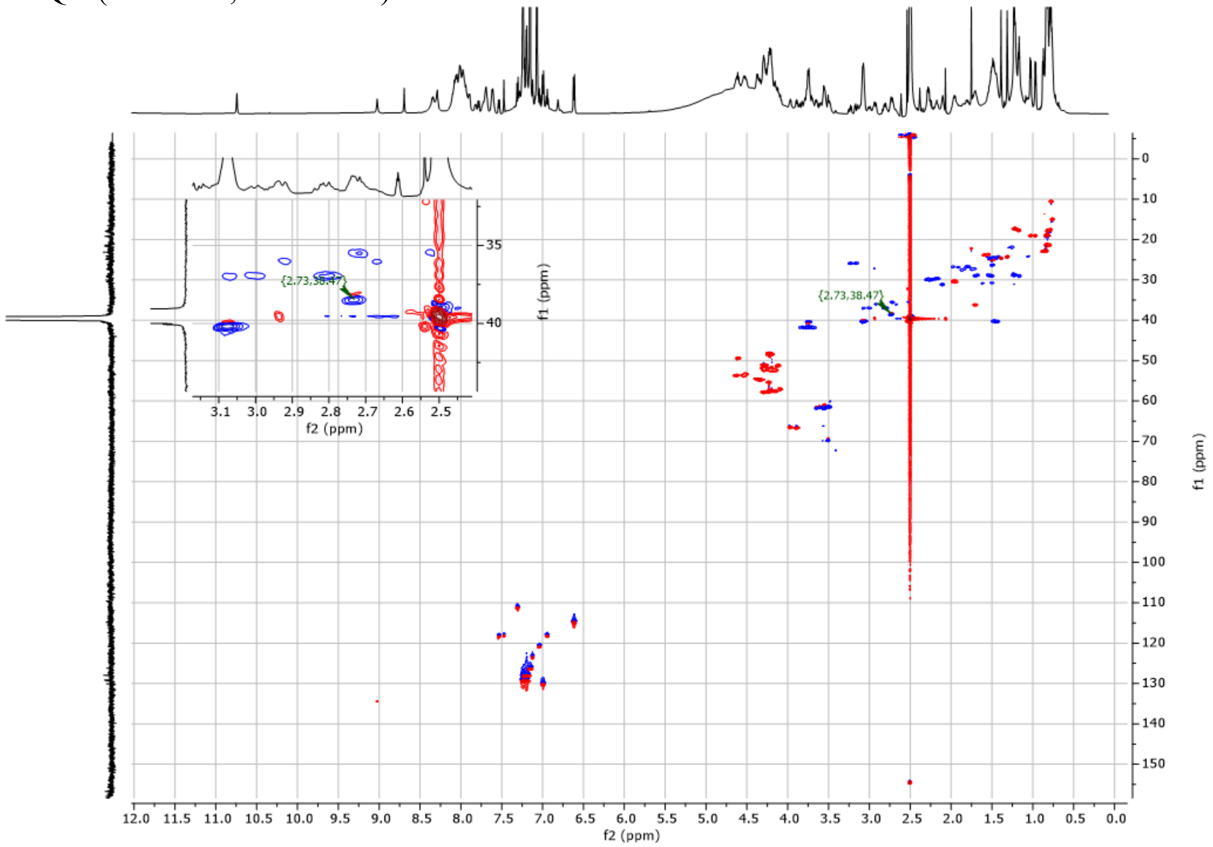

HMBC (600 MHz, DMSO-d6)

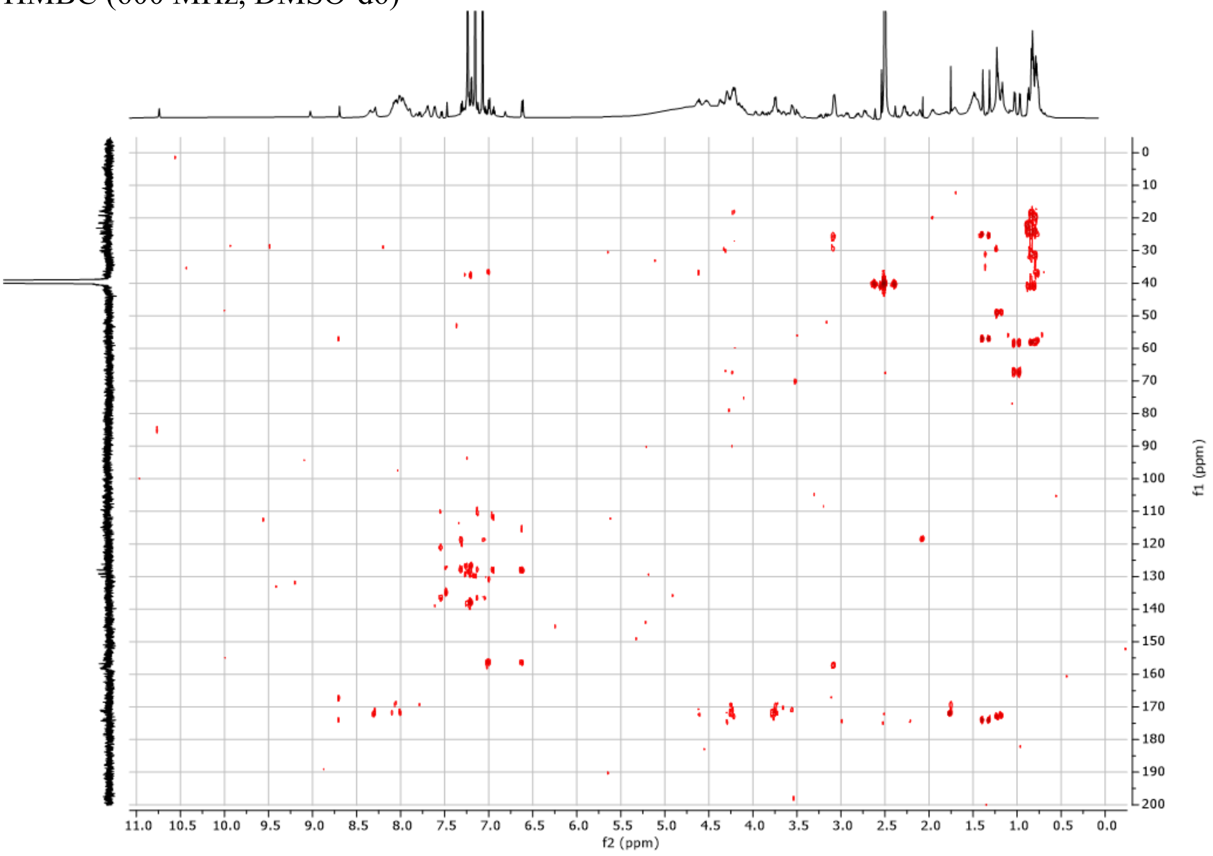

COSY (600 MHz, DMSO-d6)

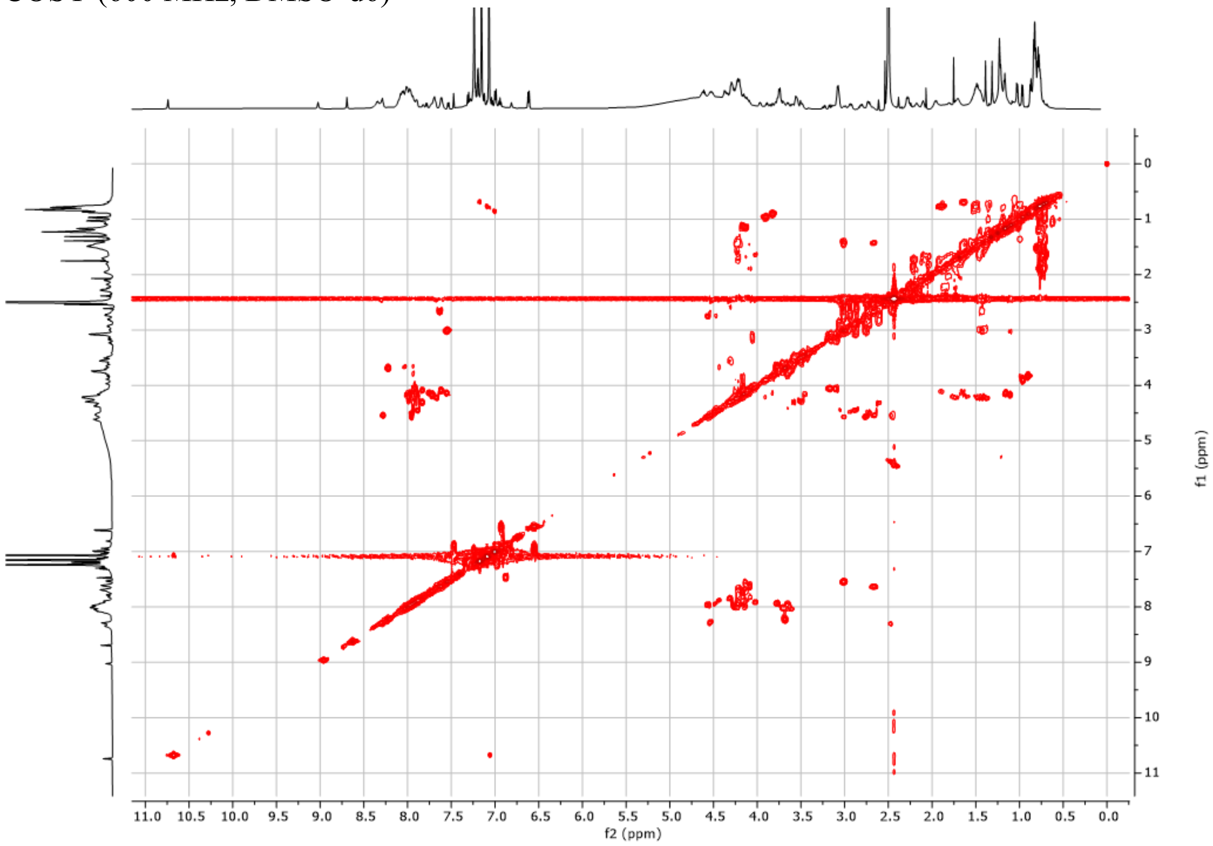

10.2.13 13G (somatostatin)

[^{12}C]13G (with DMSO:H_2O, 95:5)

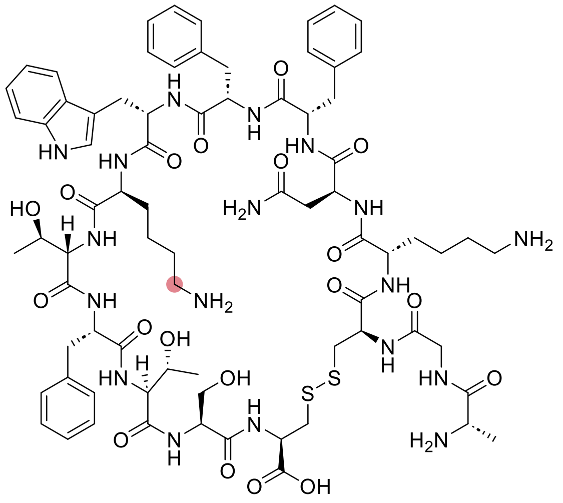

[^{12}C]13G
Chemical Formula: $C_{76}H_{104}N_{18}O_{19}S_2$
Exact Mass: 1636.7167

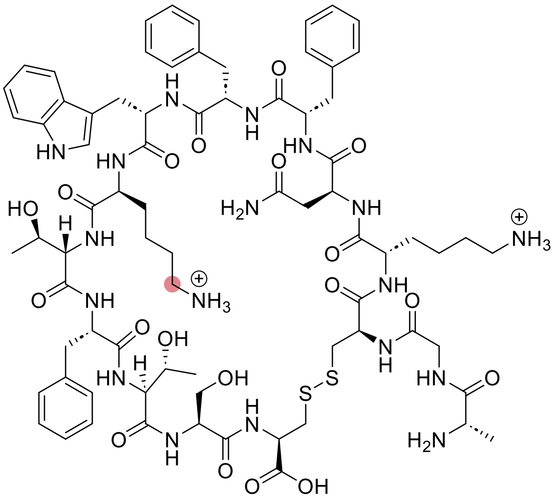

[^{12}C]13G
Chemical Formula: $C_{76}H_{106}N_{18}O_{19}S_2^{2+}$
Exact Mass: 1638.7312
m/z: 819.3656 (100.0%), 819.8673 (82.2%), 820.3690 (33.3%), 820.3635 (8.9%), 820.8707 (8.9%), 820.8652 (7.4%), 819.8641 (6.6%), 820.3658 (5.4%), 820.3678 (3.9%), 820.8694 (3.2%), 821.3669 (3.0%), 820.8675 (2.2%), 821.3723 (1.8%), 819.8653 (1.6%), 821.3711 (1.3%), 820.3670 (1.3%), 819.8688 (1.2%), 820.3704 (1.0%)

LC-MS of crude, before oxidation: ca. 58% UV-purity

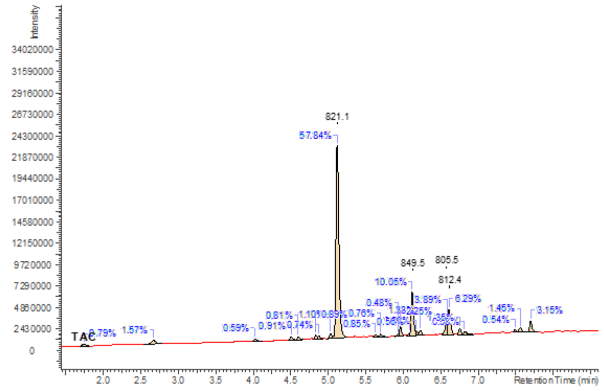

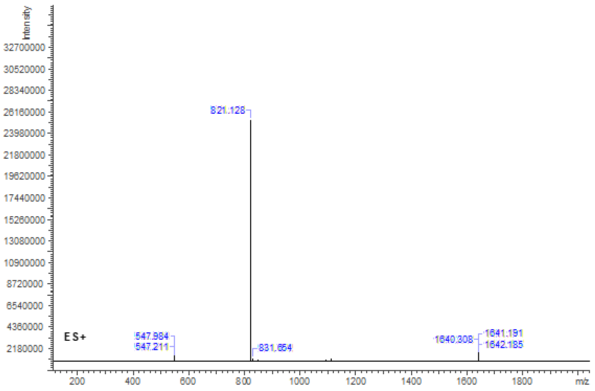

LC-MS of crude, after oxidation: ca. 53% UV-purity (2x product peak, respectively 47% and 6%)

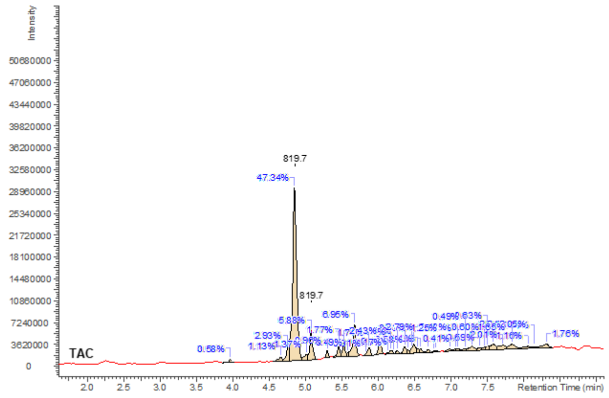

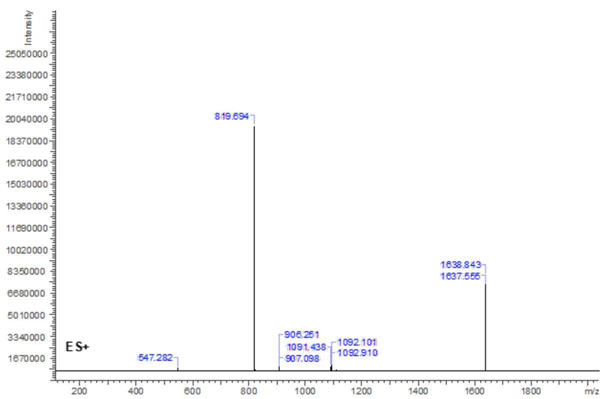

The product was not purified.

[13C]13G (with DMSO:H_2O, 95:5)

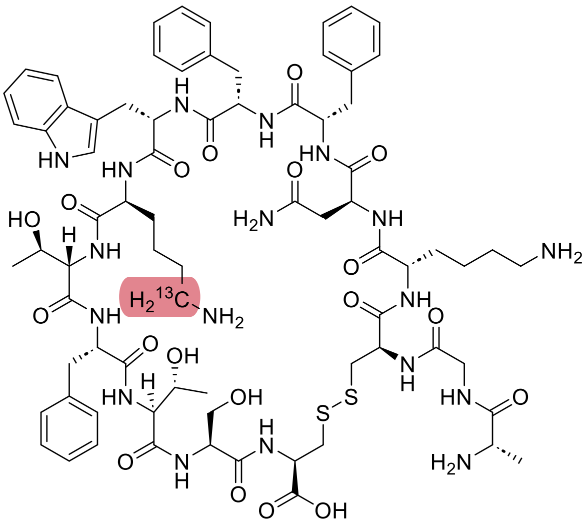

[13C]13G

Chemical Formula: $C_{75}{}^{13}CH_{104}N_{18}O_{19}S_2$
Exact Mass: 1637.7200

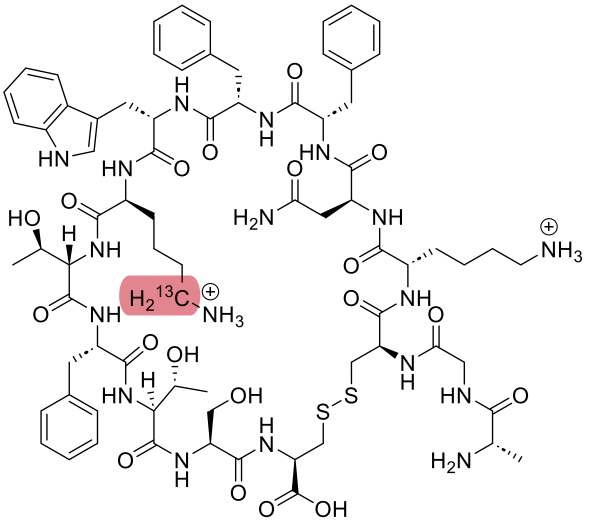

[13C]13G

Chemical Formula: $C_{75}{}^{13}CH_{106}N_{18}O_{19}S_2{}^{2+}$
Exact Mass: 1639.7346
m/z: 819.8673 (100.0%), 820.3690 (81.1%), 820.8707 (32.5%), 820.8652 (8.9%), 821.3723 (8.5%), 821.3669 (7.3%), 820.3658 (6.6%), 820.8675 (5.3%), 820.8694 (3.9%), 821.3711 (3.2%), 821.8686 (2.9%), 821.3692 (2.1%), 821.8740 (1.7%), 820.3670 (1.6%), 820.8687 (1.3%), 821.8728 (1.3%), 820.3704 (1.2%), 820.8721 (1.0%)

LC-MS of crude, before oxidation: ca. 52% UV-purity

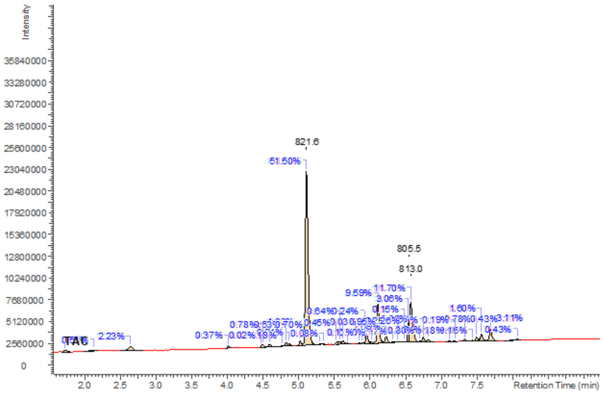

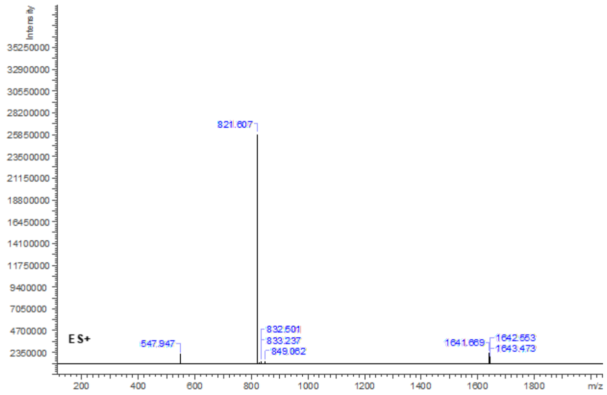

LC-MS of crude, after oxidation: ca. 44% UV-purity (2x product peaks, respectively 39% and 5%)

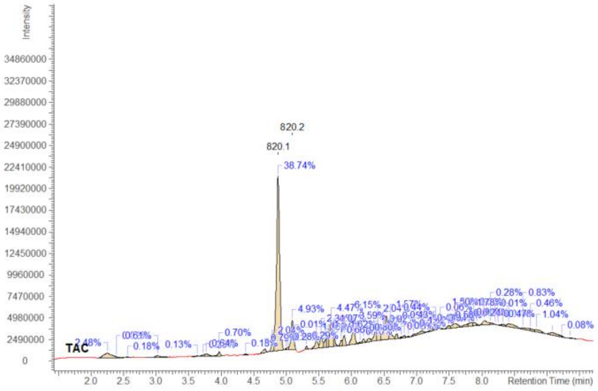

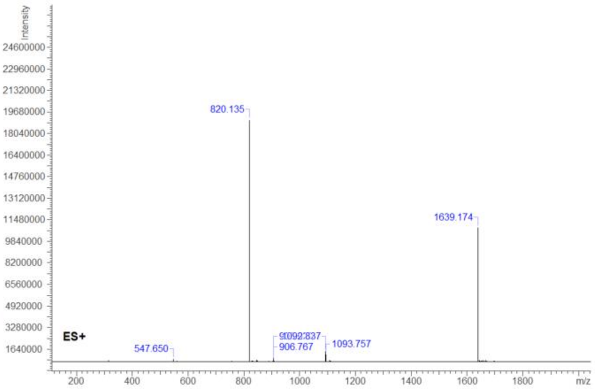

Isotope distribution comparison: Predicted non-labeled vs labeled peptide, should be ca. 0.99 ^{13}C/molecule incorporation (contains some error in analysis)

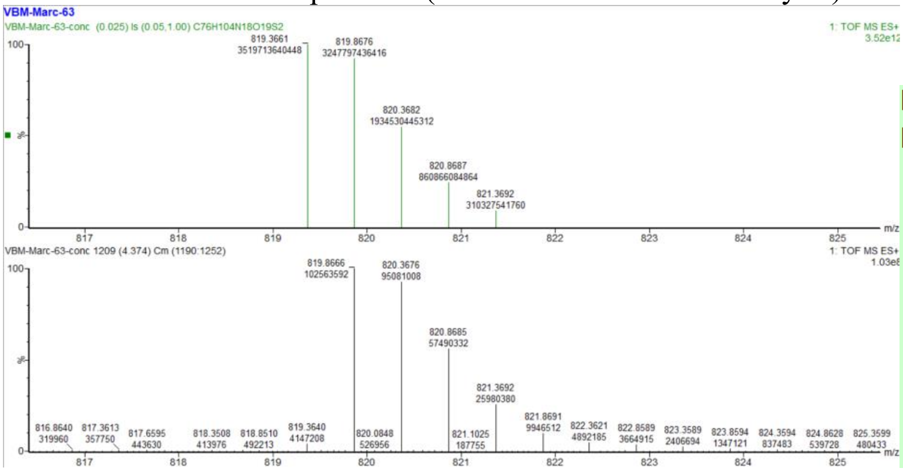

Results	
Relative amounts [%]	
unlabeled	3.8
1-label	89.9
2-label	1.5
3-label	0.6
4-label	-0.1
5-label	0.5
6-label	3.8
7-label	0.0
8-label	0.0

The product was not purified.

[^{13}C]13G (neat DMSO)

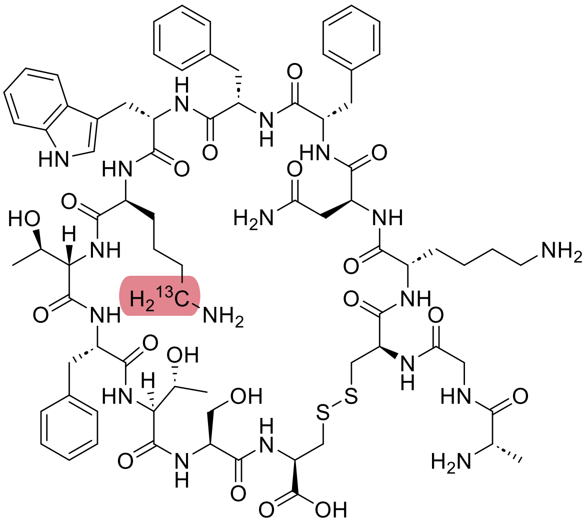

[^{13}C]13G

Chemical Formula: $C_{75}{}^{13}CH_{104}N_{18}O_{19}S_2$

Exact Mass: 1637.7200

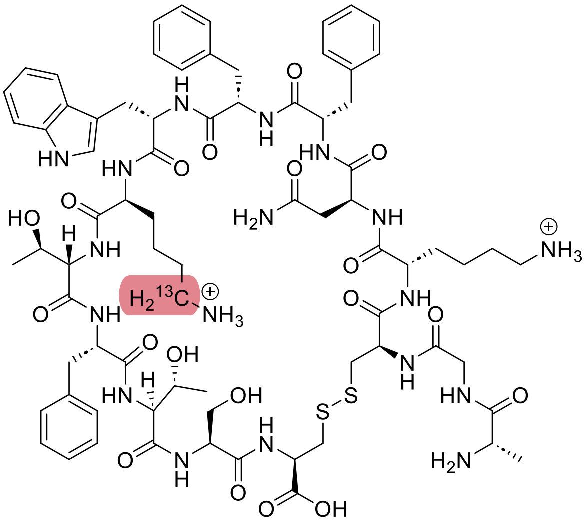

[^{13}C]13G

Chemical Formula: $C_{75}{}^{13}CH_{106}N_{18}O_{19}S_2{}^{2+}$

Exact Mass: 1639.7346

m/z: 819.8673 (100.0%), 820.3690 (81.1%), 820.8707 (32.5%), 820.8652 (8.9%), 821.3723 (8.5%), 821.3669 (7.3%), 820.3658 (6.6%), 820.8675 (5.3%), 820.8694 (3.9%), 821.3711 (3.2%), 821.8686 (2.9%), 821.3692 (2.1%), 821.8740 (1.7%), 820.3670 (1.6%), 820.8687 (1.3%), 821.8728 (1.3%), 820.3704 (1.2%), 820.8721 (1.0%)

LC-MS of crude after oxidation: ca. 40% UV-purity

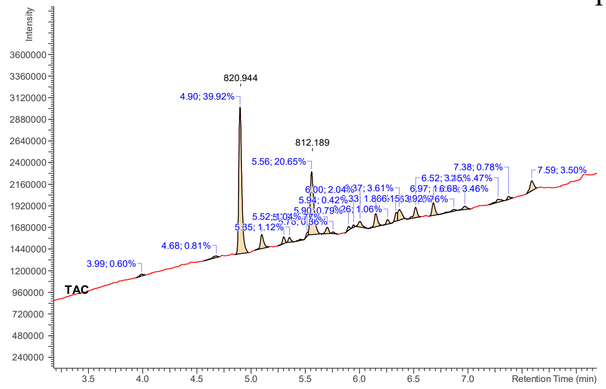

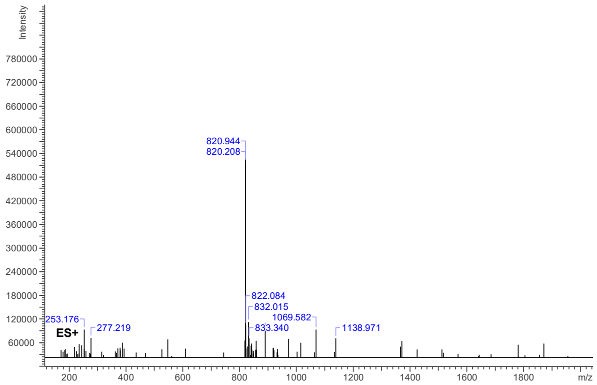

LC-MS of purified oxidated product (on HRMS column): ca. 90% UV-purity

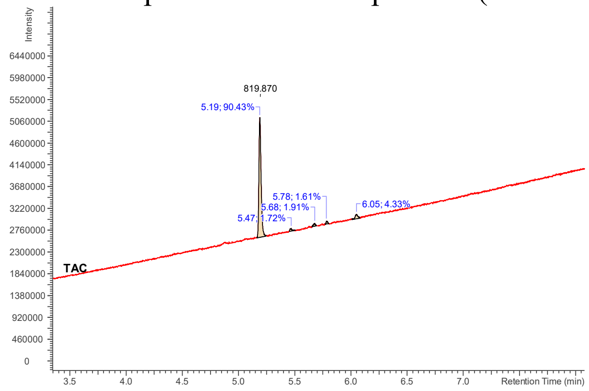

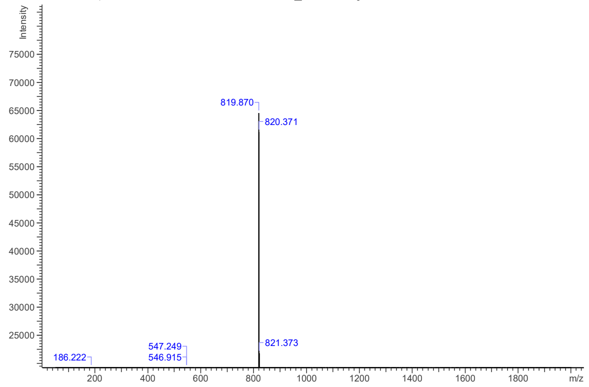

Isotope distribution comparison of oxidated product: Predicted non-labeled vs. labeled peptide, 0.99 ^{13}C/molecule incorporation

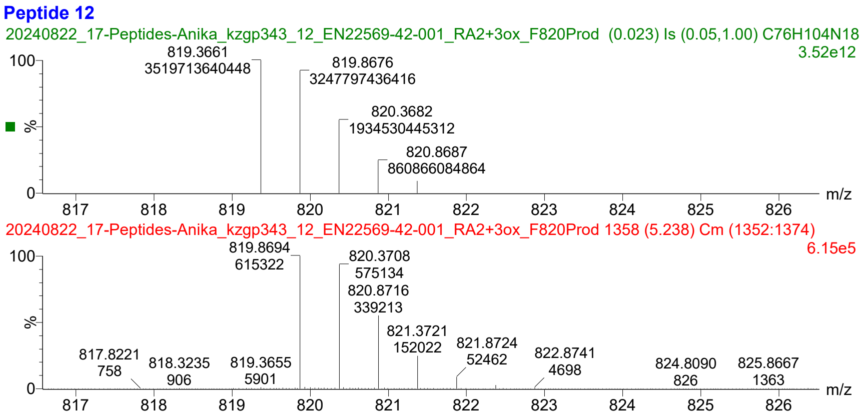

Results	
Relative amounts [%]	
unlabeled	1.0
1-label	98.3
2-label	1.5
3-label	-1.0
4-label	0.4
5-label	-0.5
6-label	0.3

NMR of purified product: ^{1}H-NMR, ^{13}C-NMR, HSQC, HMBC, COSY, TOCSY

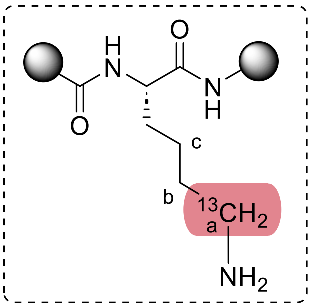

a: ^{13}C at 38.91 ppm, ^{1}H medium at 2.72 ppm (HSQC), should be split but not visible in HMBC

b and **c**: ^{1}H at 1.49/1.44 ppm and 1.24 ppm (HMBC)

NH$_2$: ^{1}H at 7.59 ppm (HMBC)

^{1}H-NMR (600 MHz, DMSO-d6)

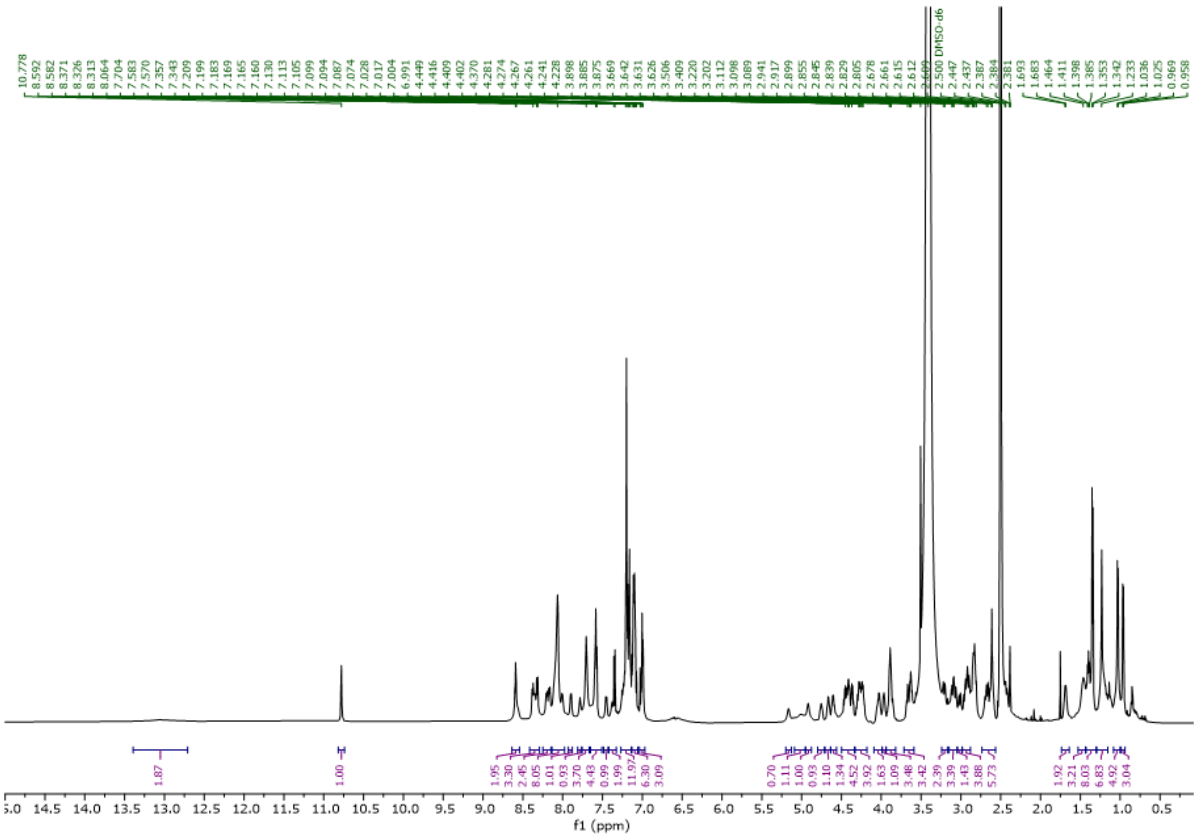

^{13}C-NMR (151 MHz, DMSO-d6)

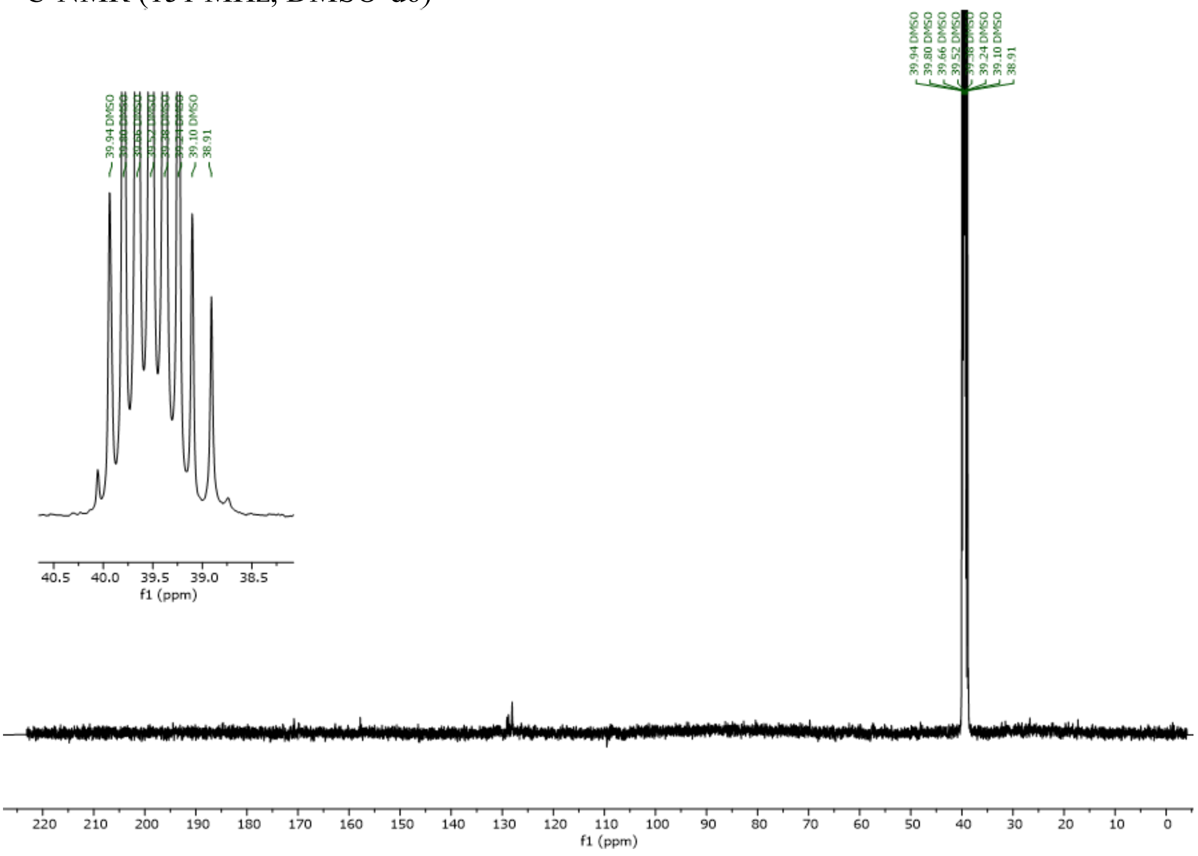

HSQC (600 MHz, DMSO-d6)

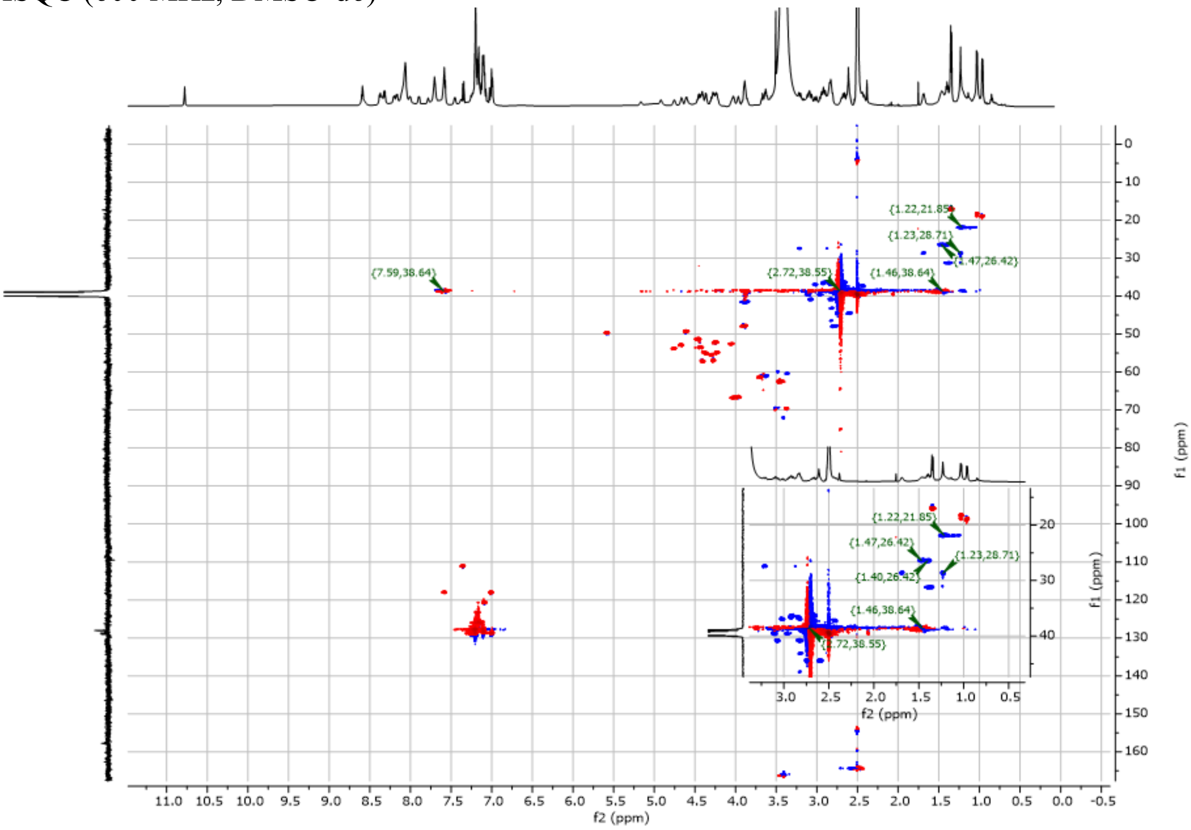

HMBC (600 MHz, DMSO-d6)

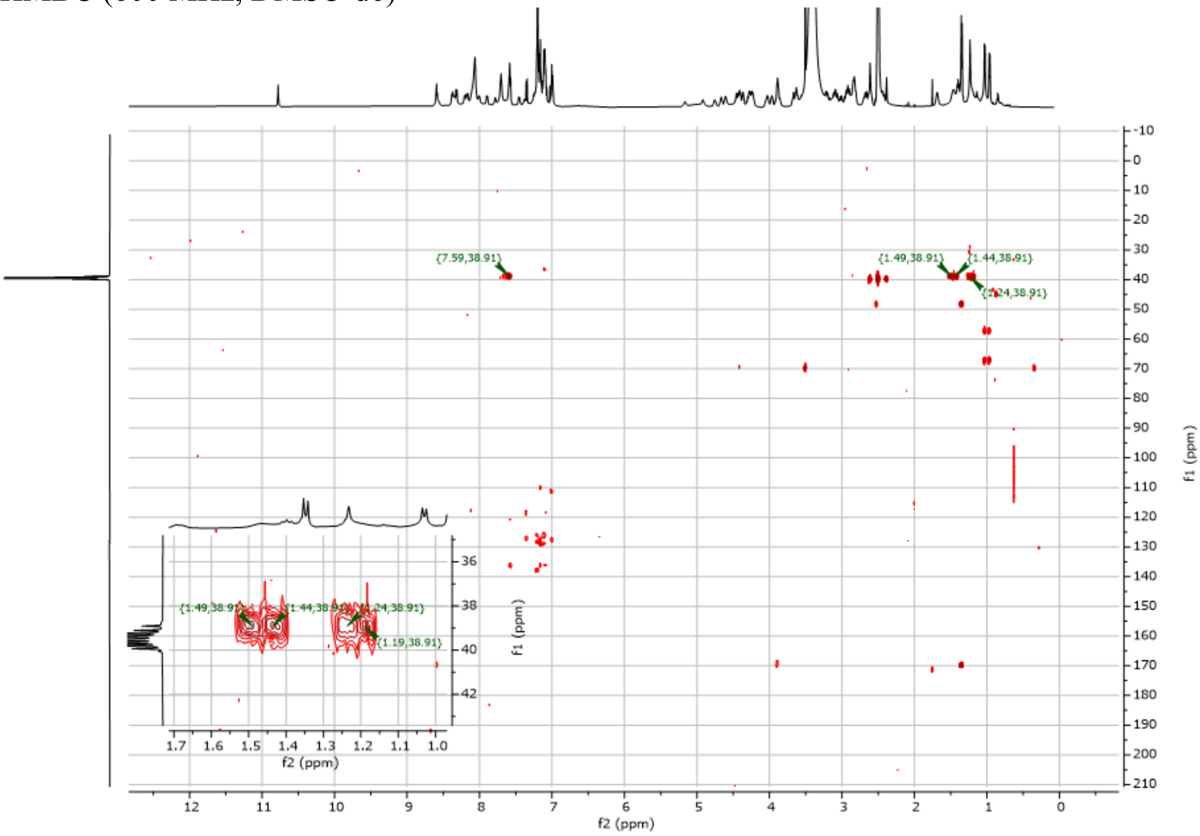

COSY (600 MHz, DMSO-d6)

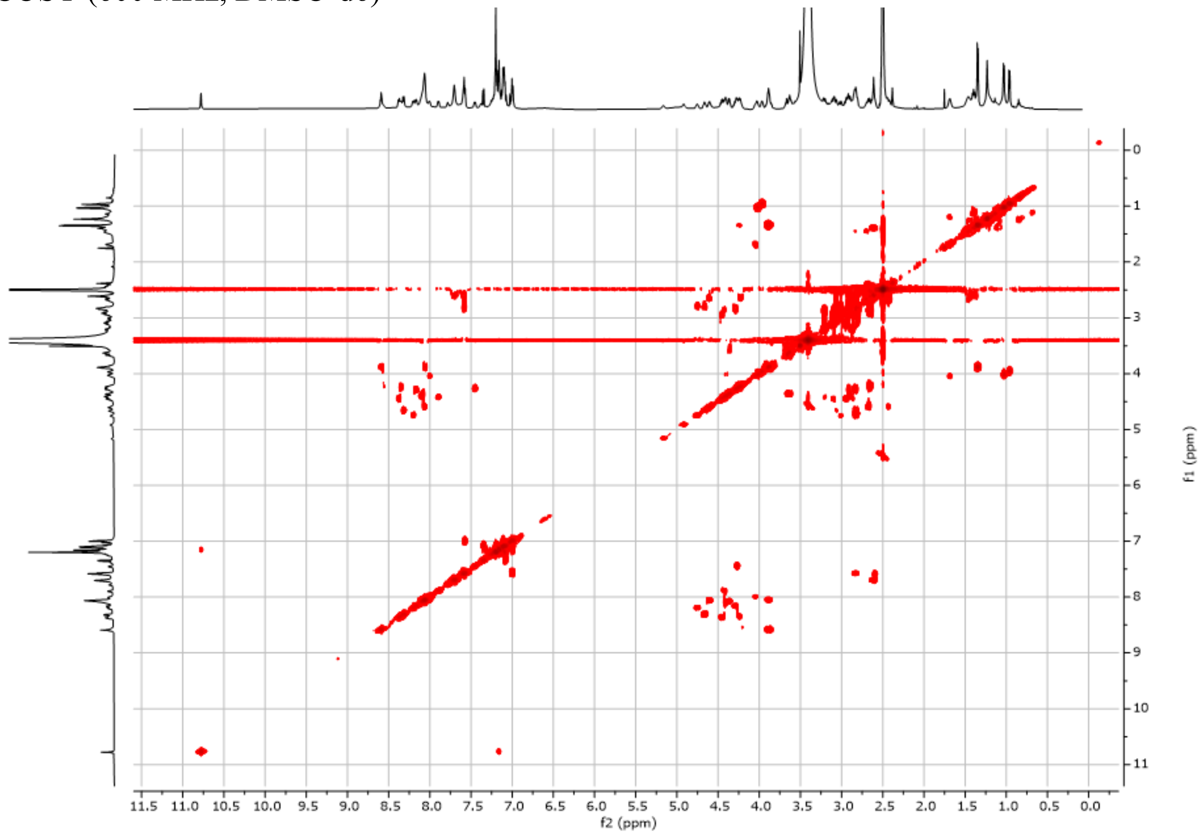

TOCSY (600 MHz, DMSO-d6)

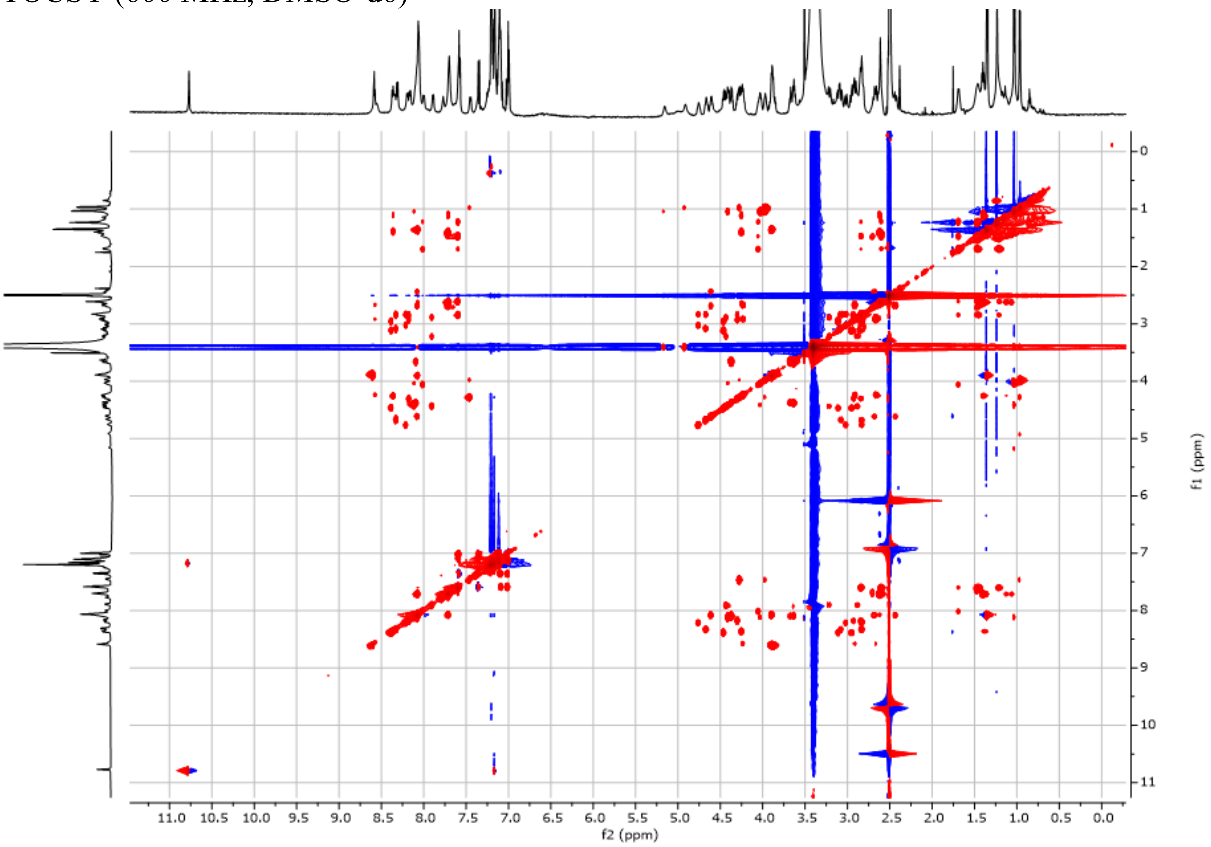

HRMS of cyclized side product after oxidative cyclization (we propose cyclization under the neat DMSO conditions, exact site of first cyclisation not further analyzed; and second oxidative disulfide bridge formation) (ESI+) (m/z): $[M+2H]^{2+}$ calculated for: $C_{75}{}^{13}CH_{101}N_{17}O_{19}S_2$, 811.3540; found 811.3563.

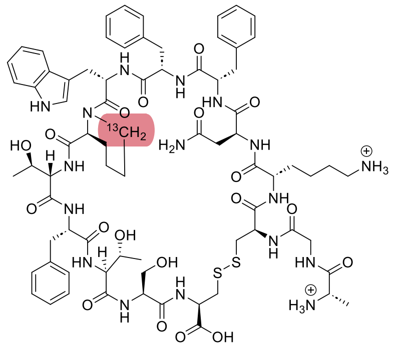

side product

Chemical Formula: $C_{75}{}^{13}CH_{103}N_{17}O_{19}S_2{}^{2+}$

Exact Mass: 1622.7080

m/z: 811.3540 (100.0%), 811.8557 (81.1%), 812.3574 (32.5%), 812.3519 (8.9%), 812.8591 (8.5%), 812.8536 (7.3%), 811.8525 (6.2%), 812.3542 (5.0%), 812.3562 (3.9%), 812.8578 (3.2%), 813.3553 (2.9%), 812.8559 (2.0%), 813.3607 (1.7%), 811.8537 (1.6%), 812.3554 (1.3%), 813.3595 (1.3%), 811.8572 (1.2%), 812.3588 (1.0%)

Peptide 13

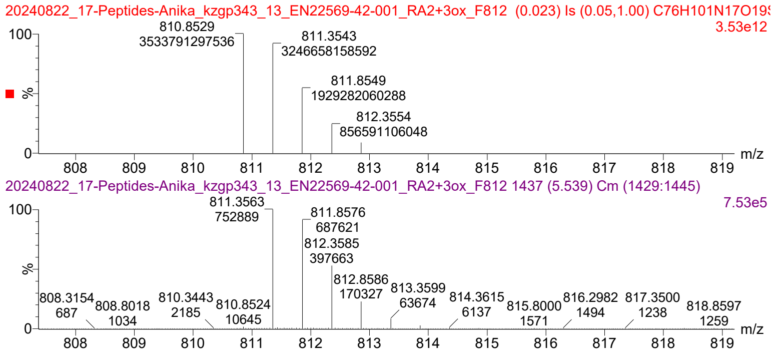

10.2.14 14D (terlipressin)

[12C]14G

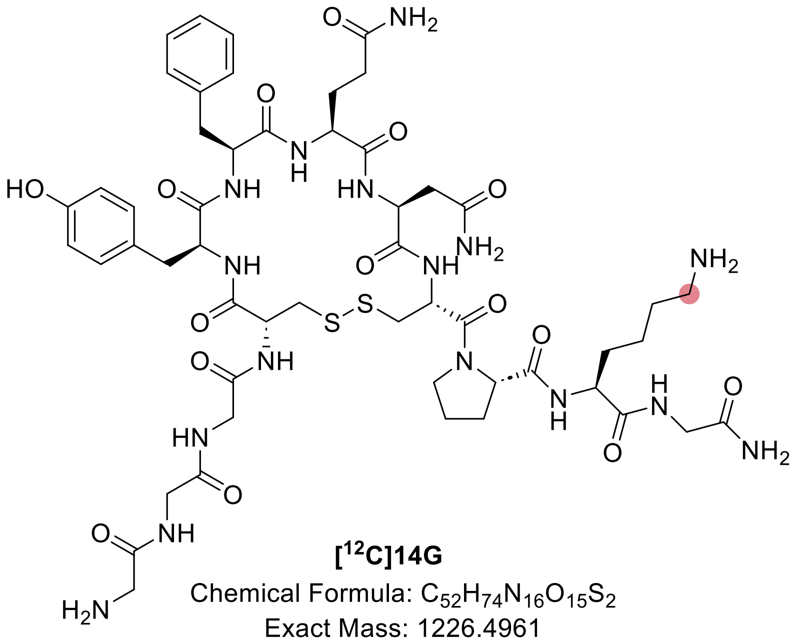

[12C]14G

Chemical Formula: $C_{52}H_{74}N_{16}O_{15}S_2$

Exact Mass: 1226.4961

LC-MS of crude before oxidation: ca. 47% UV-purity (2x product peak, respectively 44% and 3%)

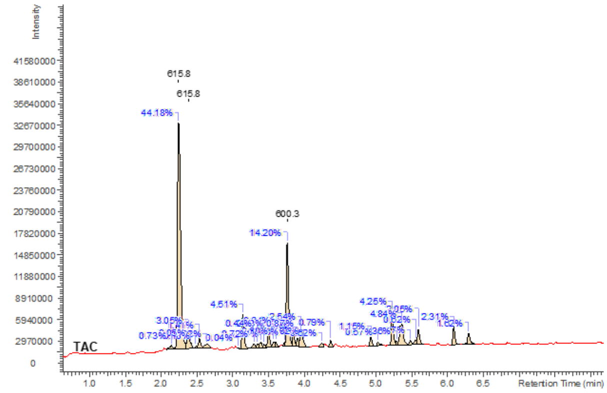

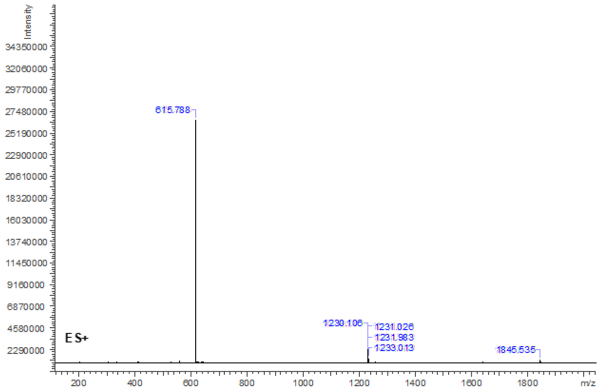

LC-MS of crude after oxidation: ca. 48% UV-purity

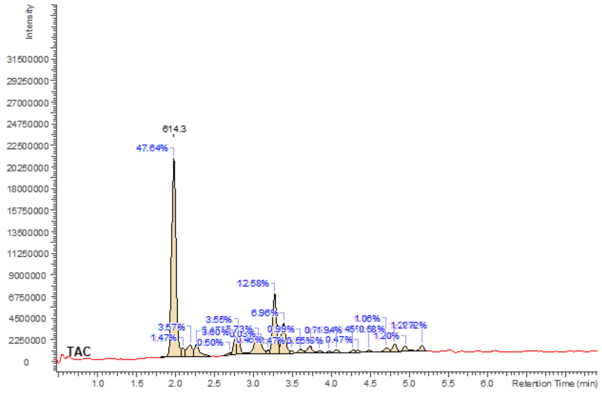

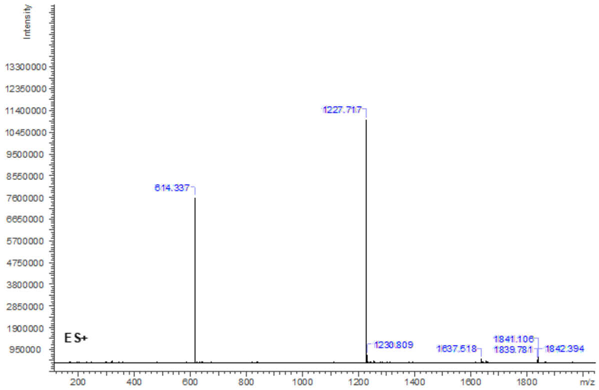

LC-MS of purified product: ca. 72% UV-purity (2x product peak, respectively 69% and 3%)

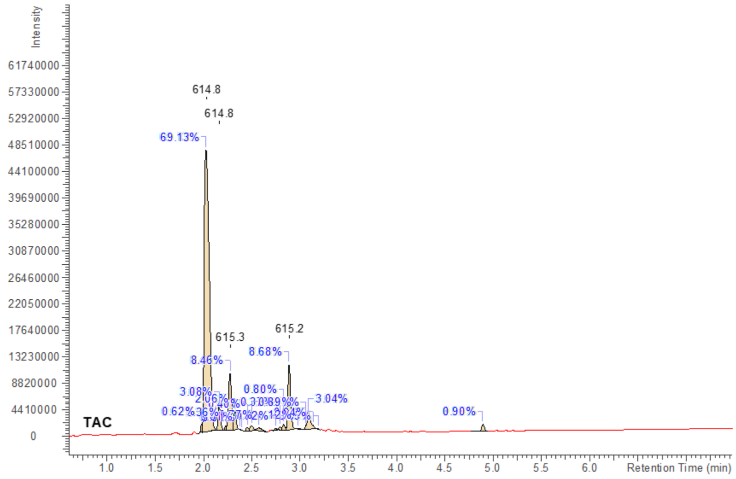

NMR of purified product: ^{1}H-NMR, ^{13}C-NMR

^{1}H-NMR (600 MHz, DMSO-d6)

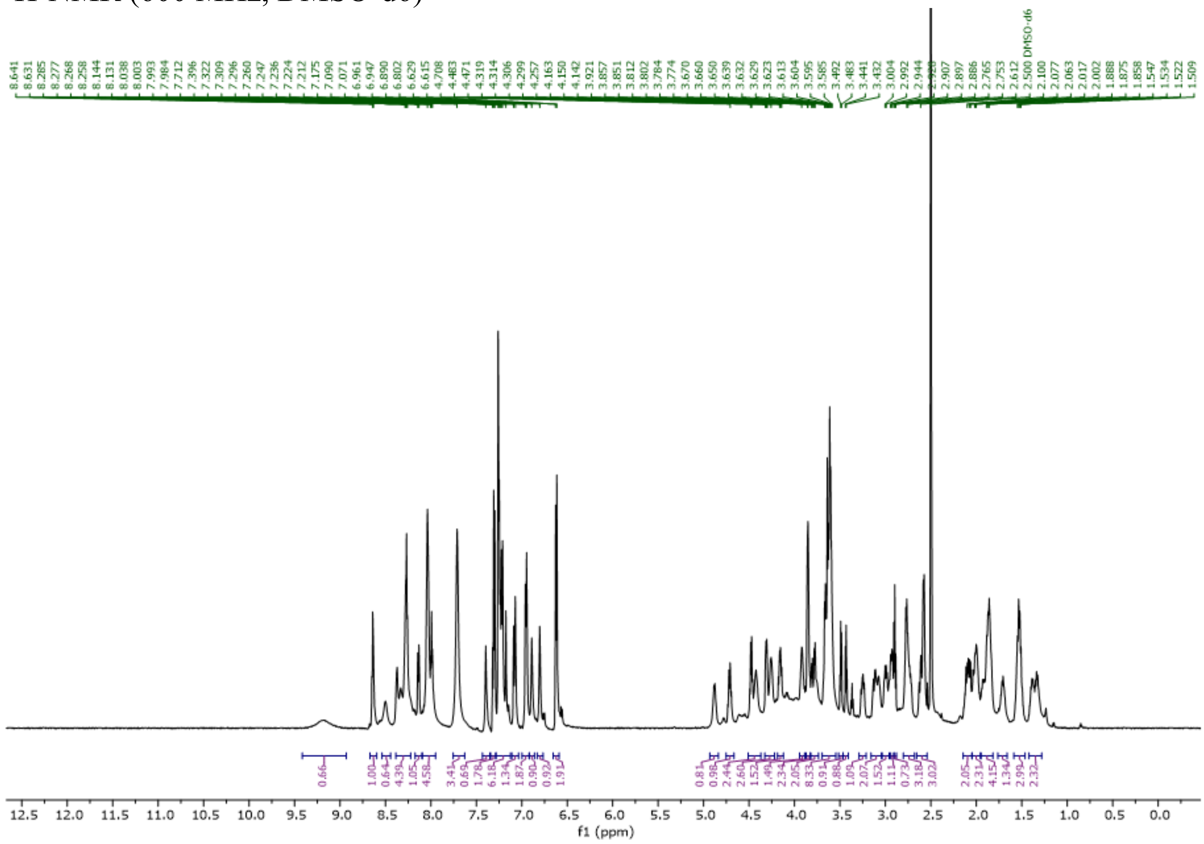

^{13}C-NMR (151 MHz, DMSO-d6)

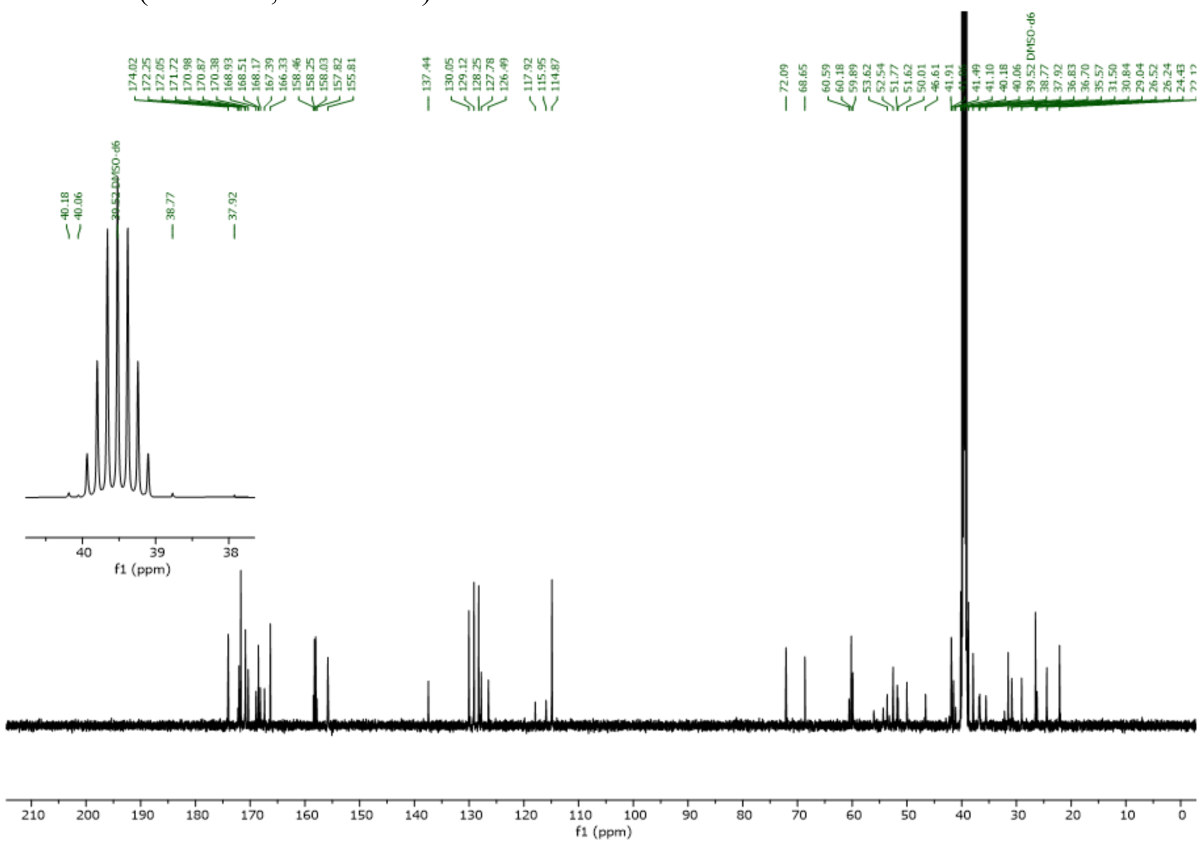

[13C]14G

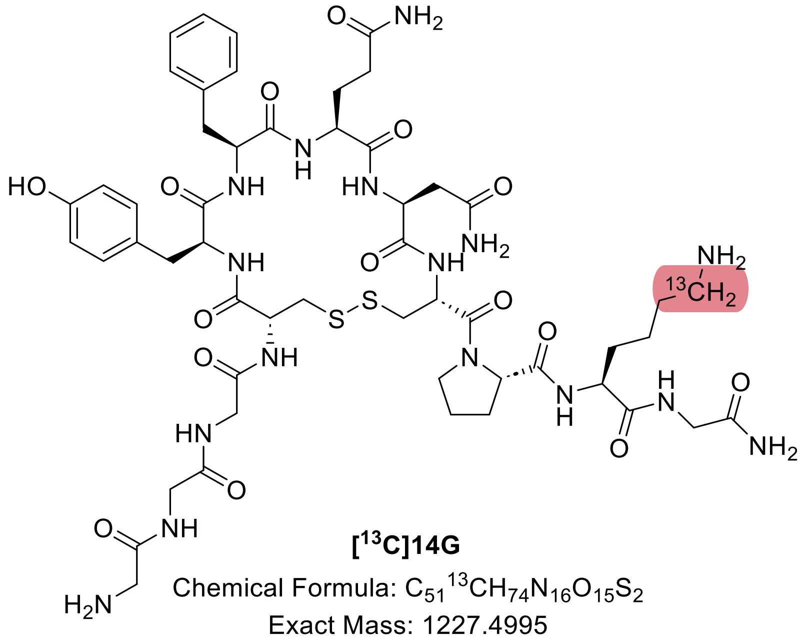

[13C]14G

Chemical Formula: $C_{51}{}^{13}CH_{74}N_{16}O_{15}S_2$

Exact Mass: 1227.4995

LC-MS of crude before oxidation: ca. 48% UV-purity

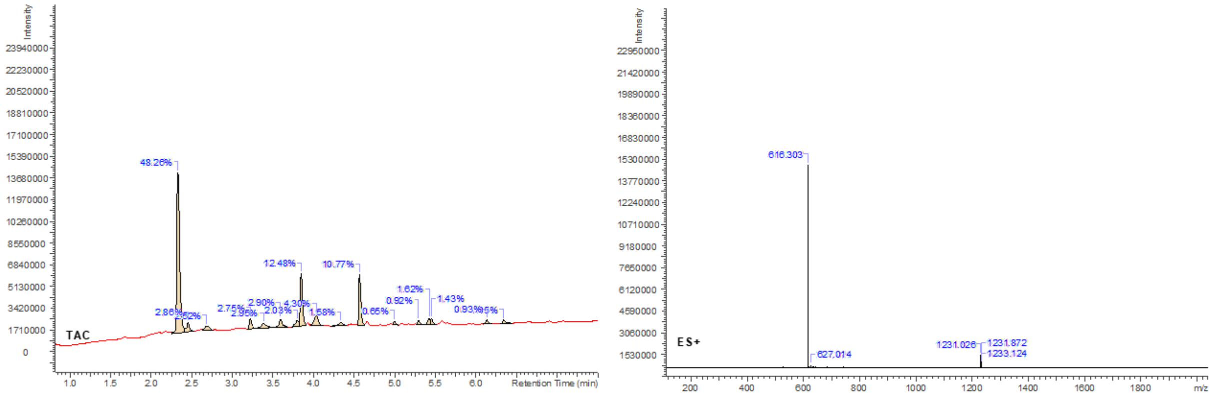

LC-MS of crude after oxidation: ca. 44% UV-purity

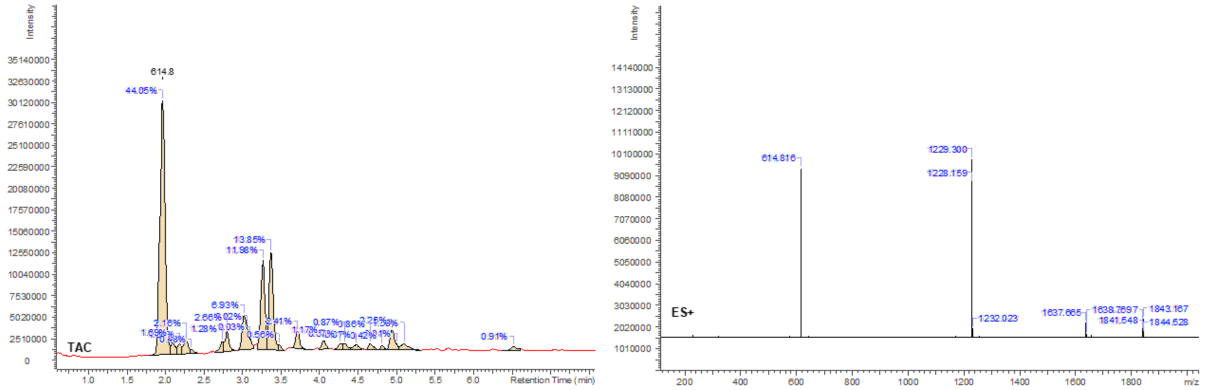

LC-MS of purified product: ca. 73% UV-purity (2x product peaks, respectively 70% and 3%)

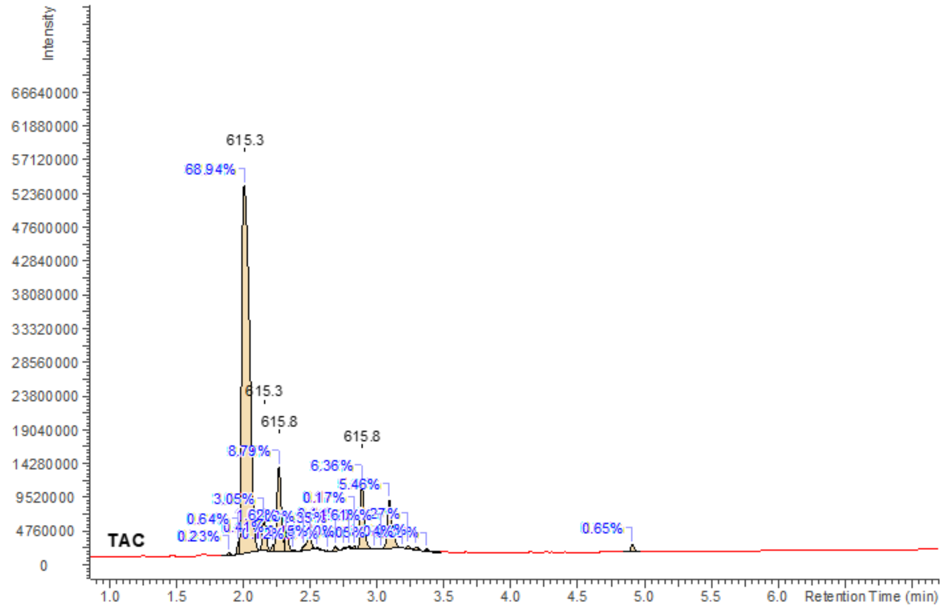

Isotope distribution comparison: Predicted non-labeled vs labeled peptide, incorporation is assumed to be +0.99 ^{13}C/moleule, but could not be calculated because the sample for HRMS was very impure, and side products from decomposition did not reveal a nice isotope pattern of the product because the co-eluting side product had an isotope shift of ca +1.

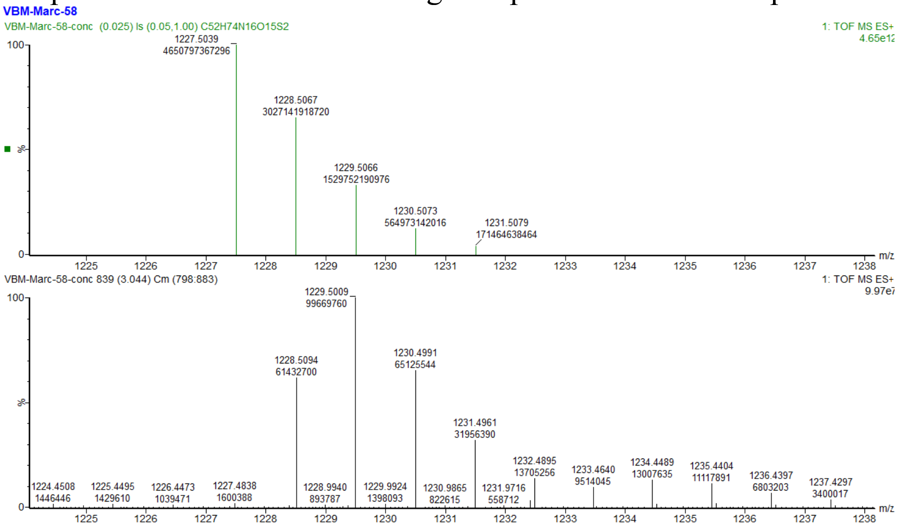

Side-product isotope pattern: could be the deaminated peptide (Gln → Glu, or Asn →Asp), but was not further characterized

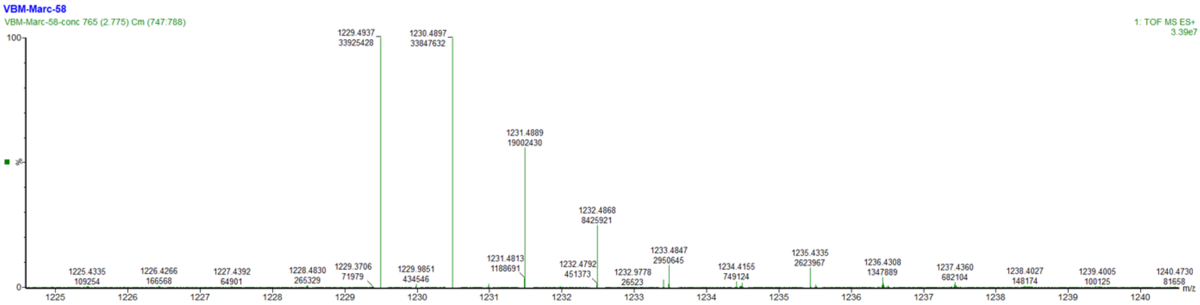

^{1}H-NMR (600 MHz, DMSO-d6)

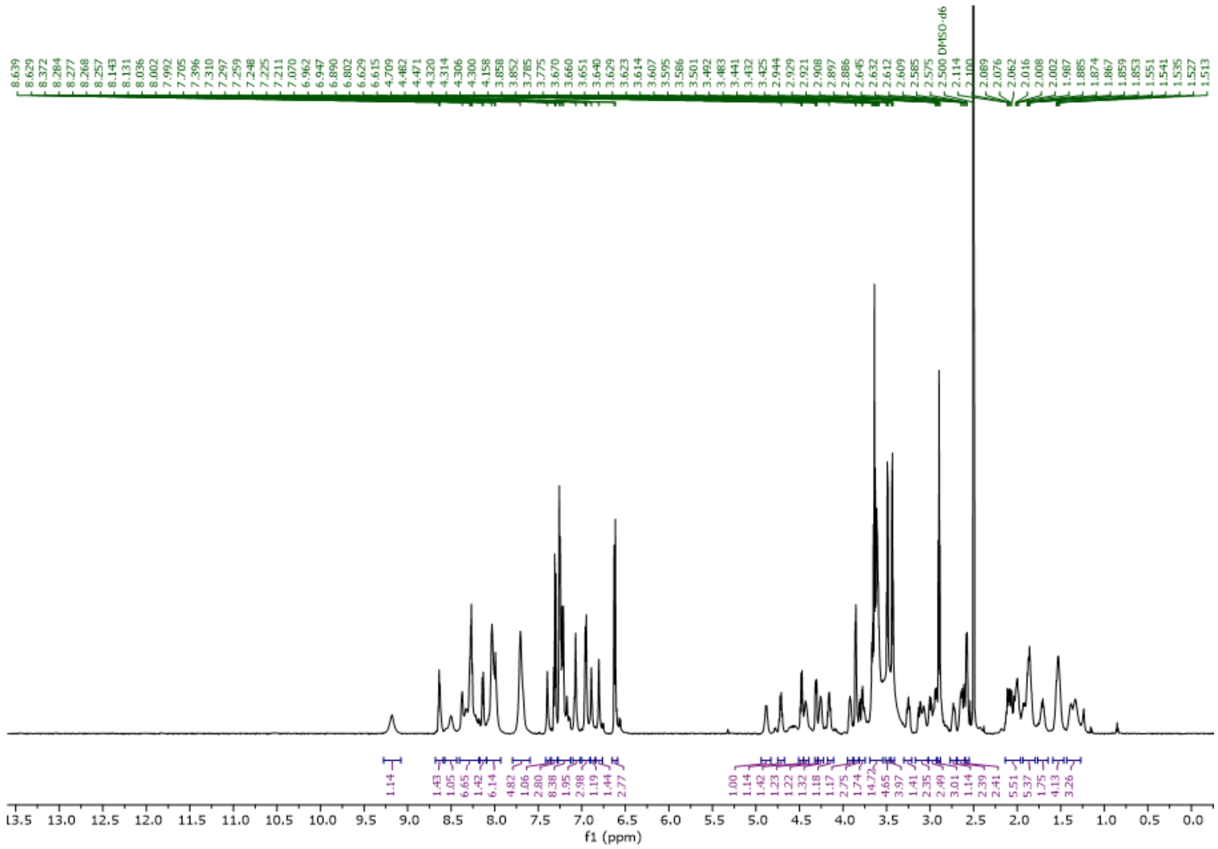

^{13}C-NMR (151 MHz, DMSO-d6): ^{13}C-label at 38.77 ppm.

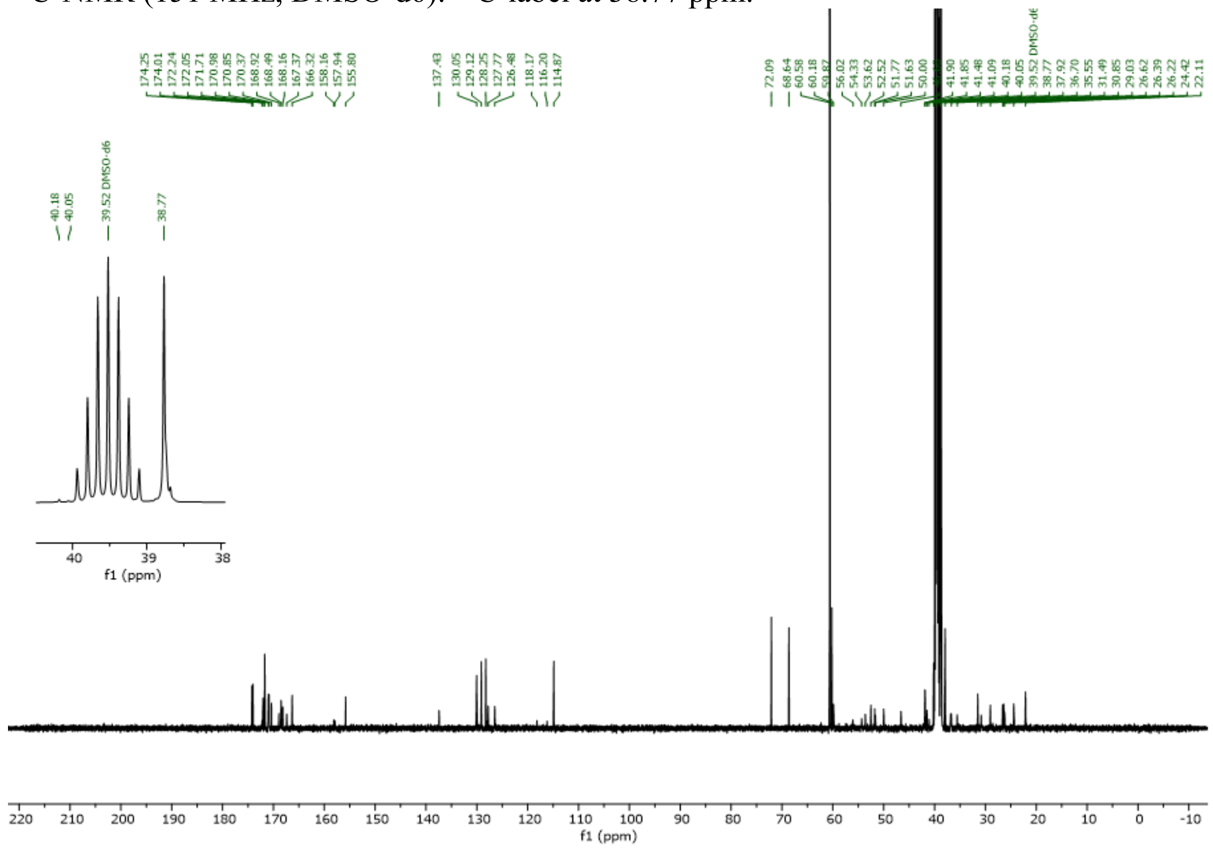

10.2.15 **15B** (BIO-11006 analog thioacetal) and **15A'a**, **15A'b**, **15D** (cyclized BIO-11006 analogs)

15B (Cleavage i)

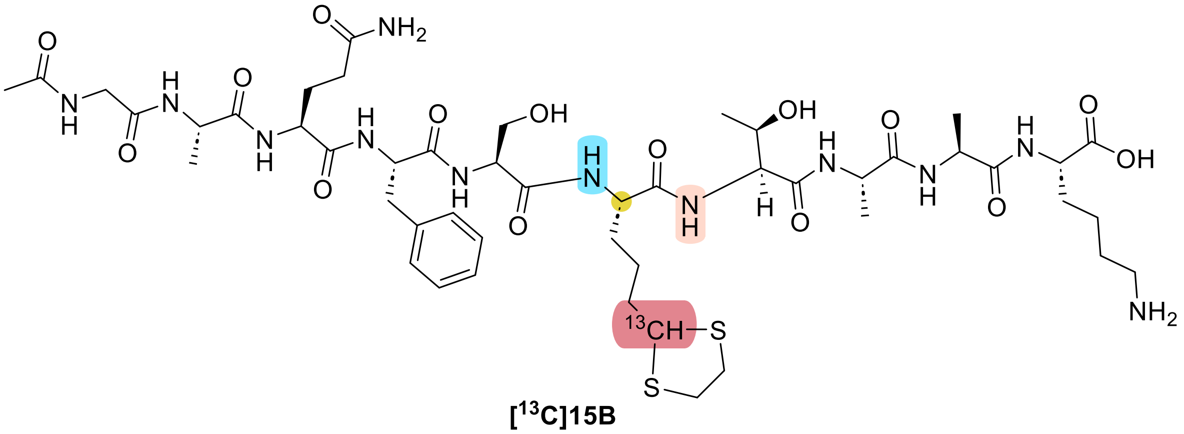

[^{13}C]15B

Chemical Formula: $C_{47}{}^{13}CH_{76}N_{12}O_{15}S_2$

Exact Mass: 1125.5028

LC-MS of crude: ca. 61% UV-purity

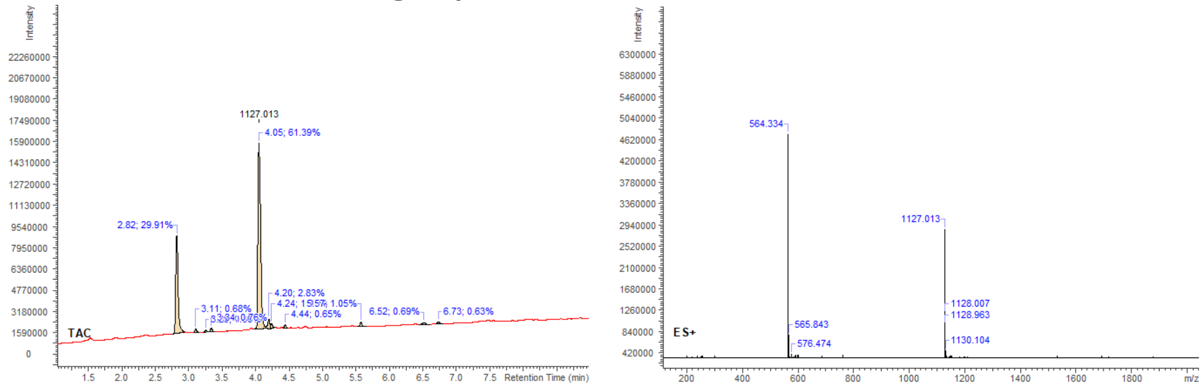

Isotope distribution comparison: Predicted non-labeled vs. labeled peptide, ca. +0.99 ^{13}C/molecule incorporation

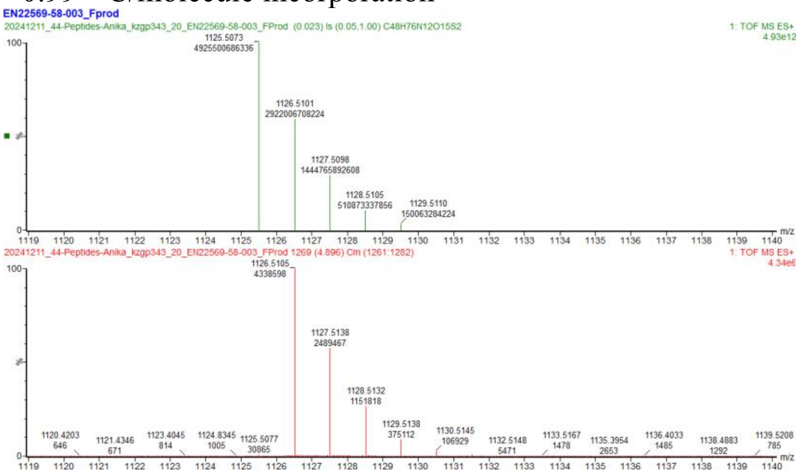

Results	
Relative amounts [%]	
unlabeled	0.7
1-label	102.1
2-label	-2.0
3-label	-1.6
4-label	-0.2
5-label	0.2
6-label	0.8

LC-MS of purified product: ca. 99% UV-purity

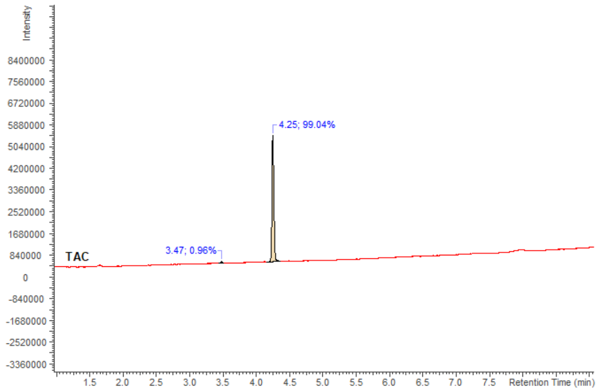

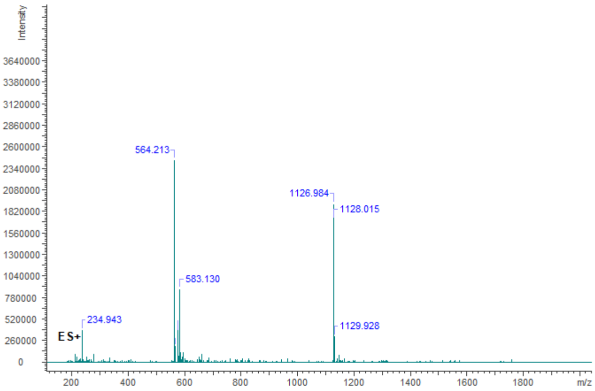

NMR of purified product: ^{1}H-NMR, ^{13}C-NMR, HSQC, HMBC

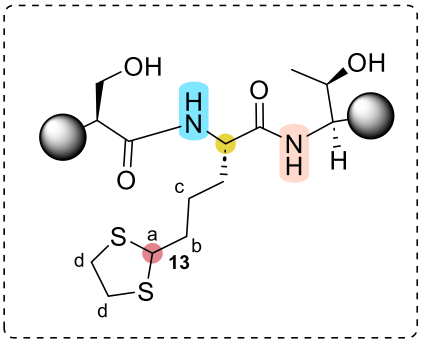

a: ^{13}C at 52.99 ppm, ^{1}H medium at 4.45 ppm (HSQC), split protons at 4.58 ppm and 4.32 ppm (HMBC)

b and **c**: ^{1}H at 1.77/1.74/1.73/1.70 ppm and at 1.42 ppm (HMBC)

d: ^{1}H at 3.23/3.21/3.15/3.14 ppm (HMBC)

^{1}H-NMR (600 MHz, DMSO-d6)

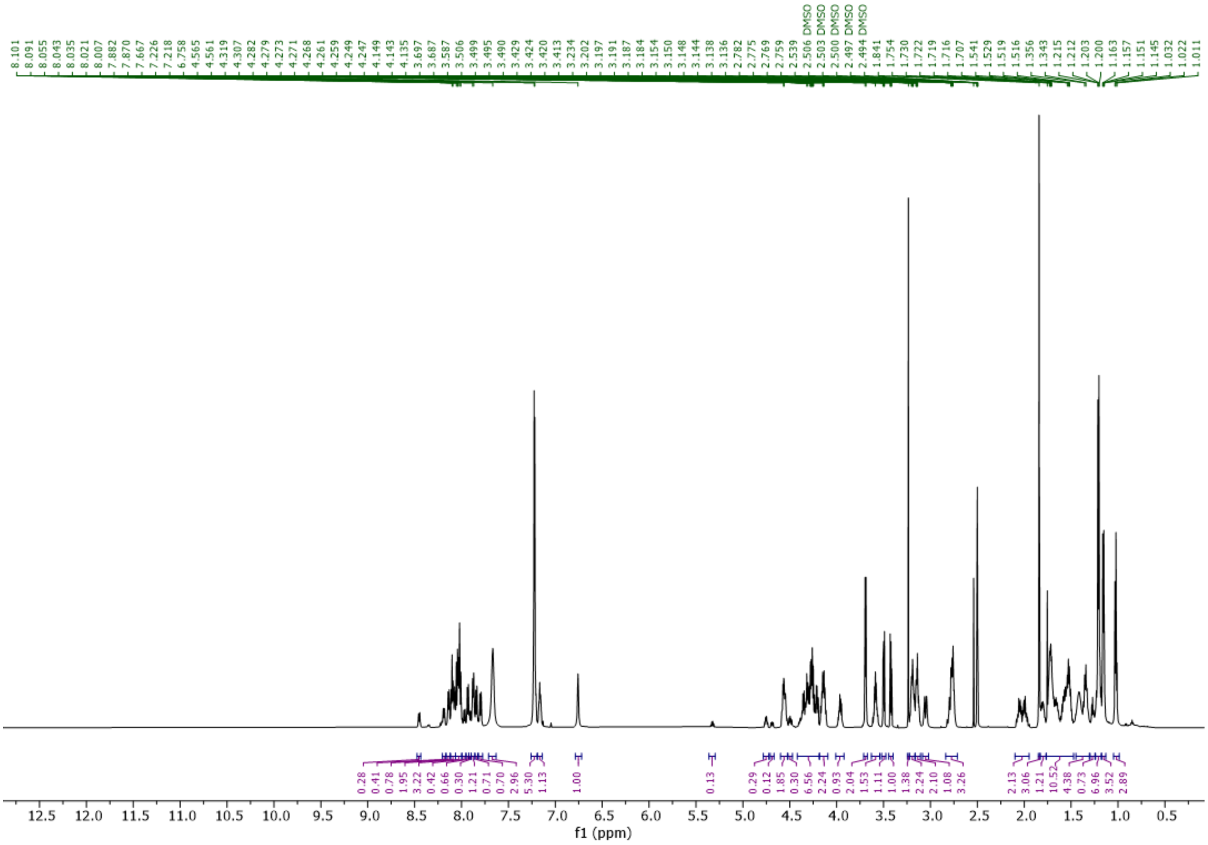

^{13}C-NMR (151 MHz, DMSO-d6)

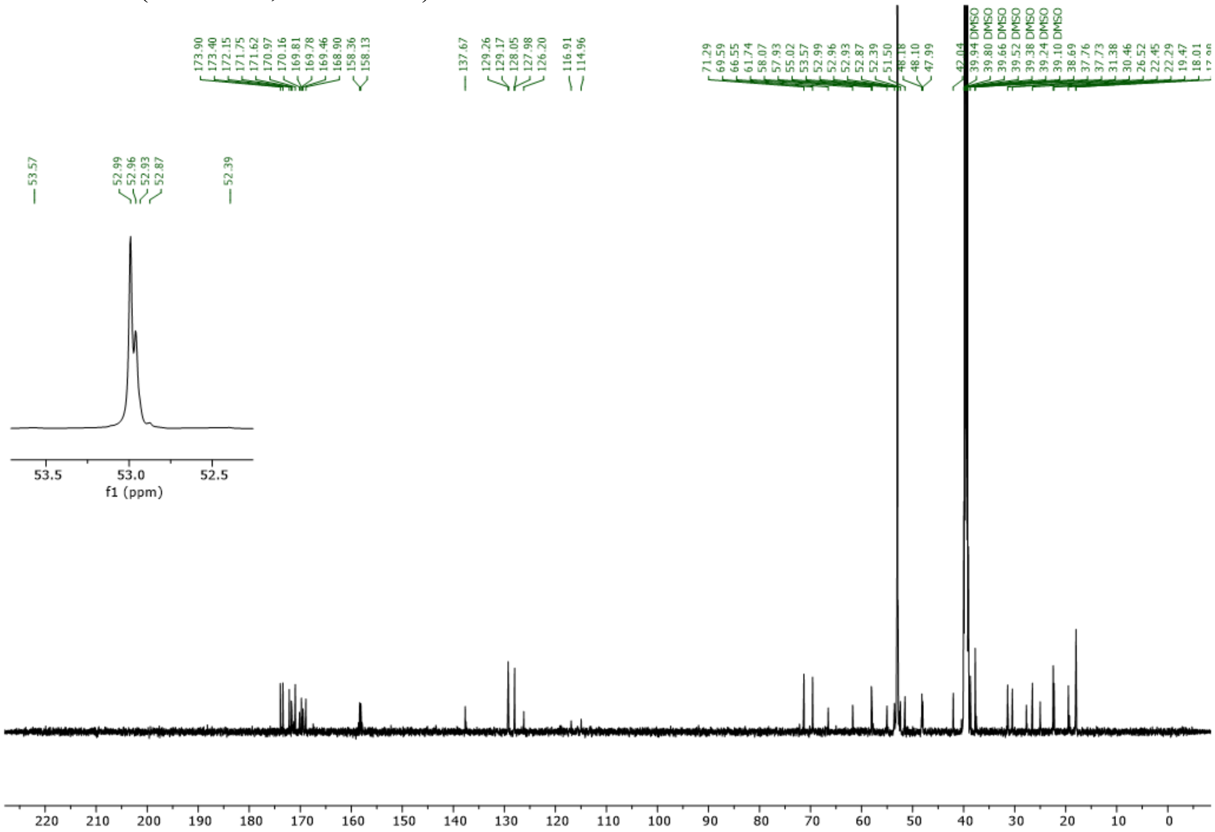

HSQC (600 MHz, DMSO-d6)

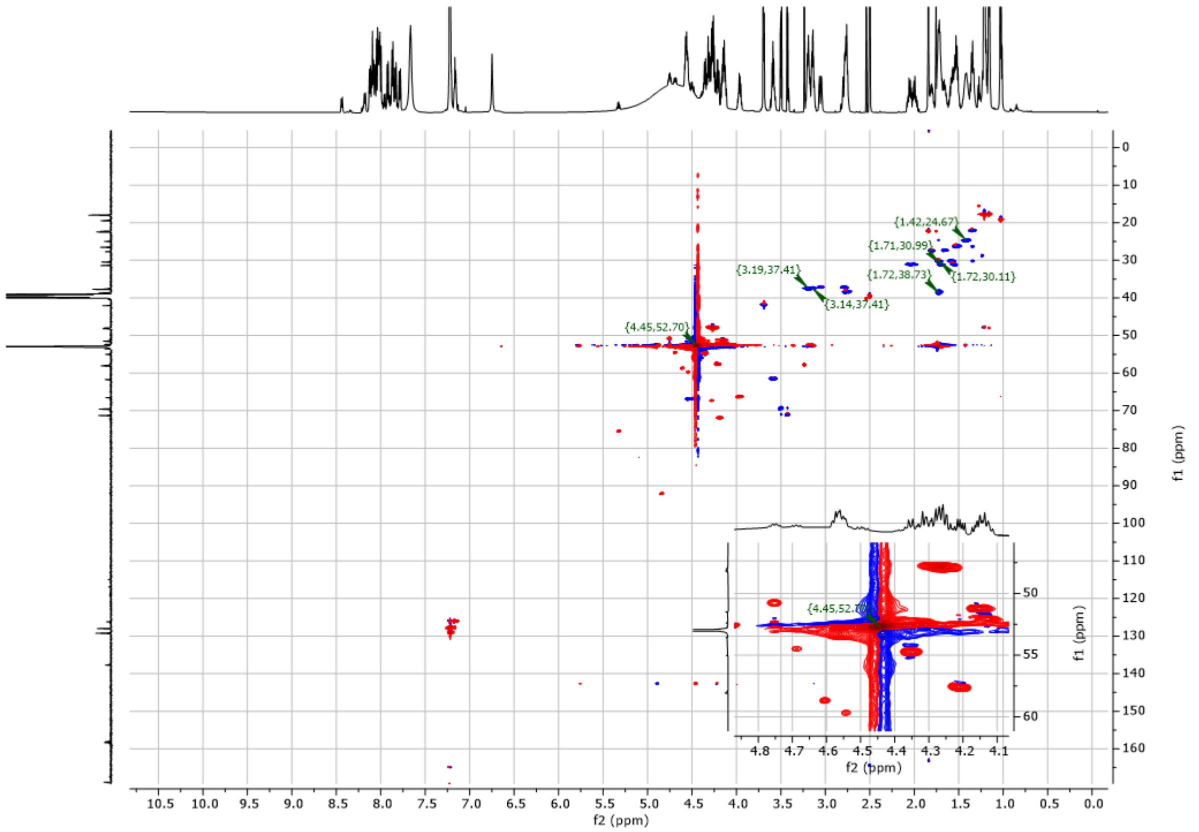

HMBC (600 MHz, DMSO-d6)

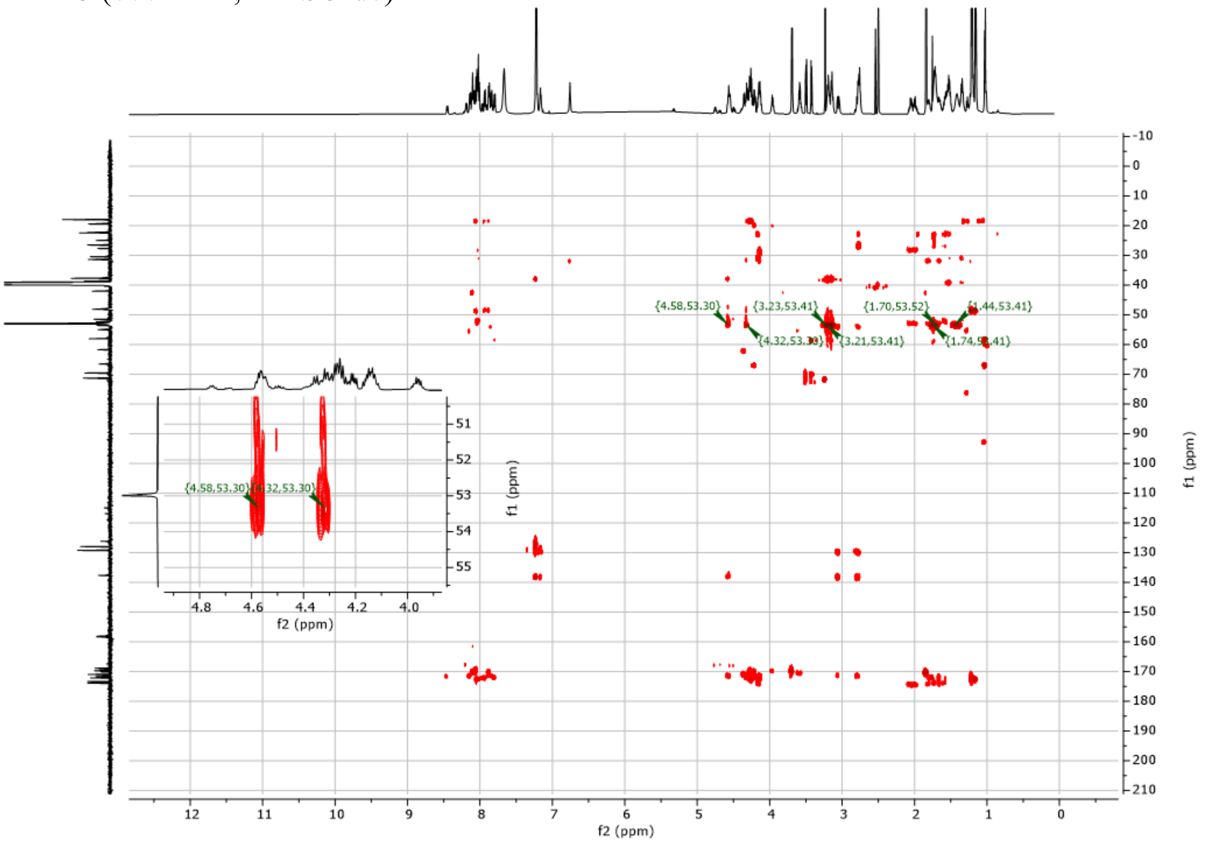

15A'a, proposed structure (Cleavage ii) (coelutes/contaminated with SM)

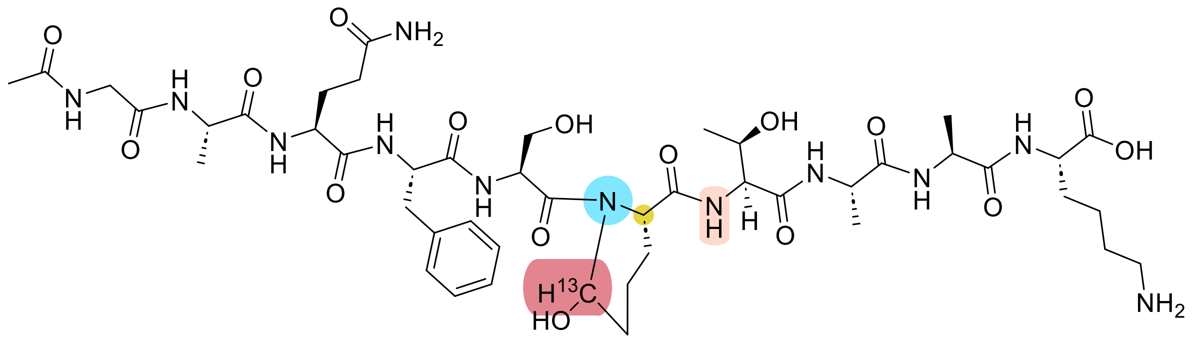

[^{13}C]15A'a

Chemical Formula: $C_{45}{}^{13}CH_{72}N_{12}O_{16}$

Exact Mass: 1049.5223

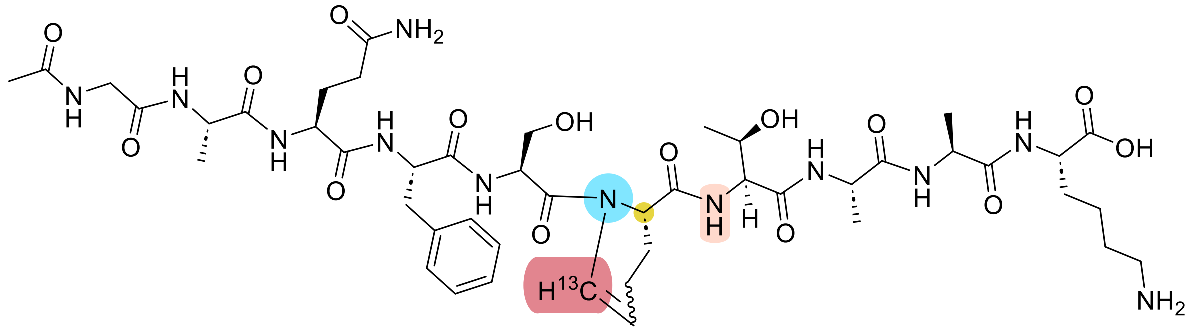

[^{13}C]15A'a

Chemical Formula: $C_{45}{}^{13}CH_{70}N_{12}O_{15}$

Exact Mass: 1031.5117

LC-MS of crude: ca. <54% UV-purity of **[^{13}C]15A'a** (co-elutes with **15SM**), and ca. 30% UV-purity of **[^{13}C]15A'b**

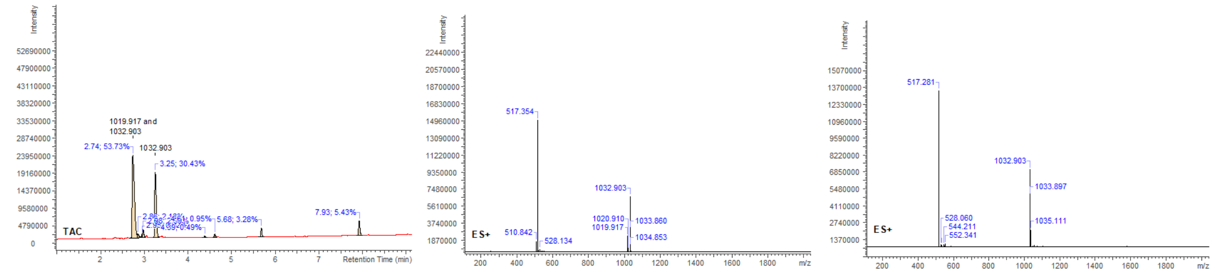

LC-MS of purified product: co-elutes with starting material **15SM** (purity can't be analyzed), <88% UV-purity

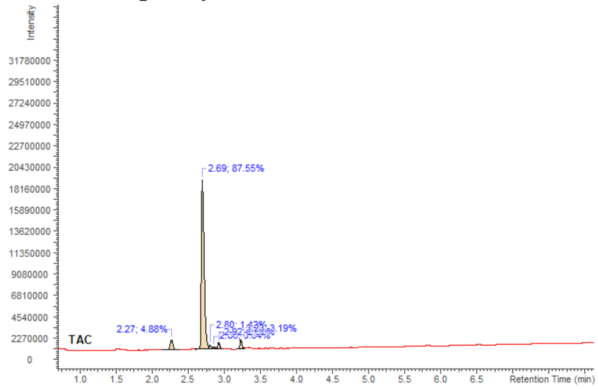

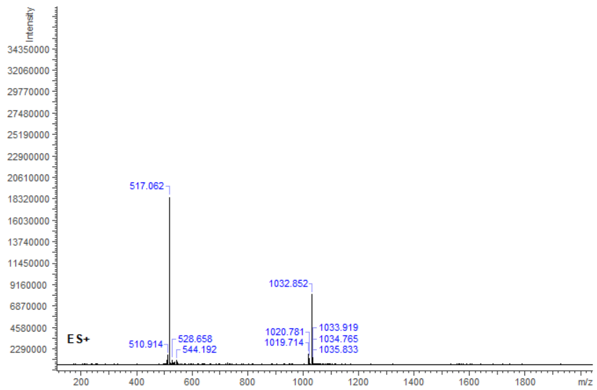

Isotope distribution comparison: Predicted non-labeled vs labeled peptide, +0.99 ^{13}C/molecule incorporation

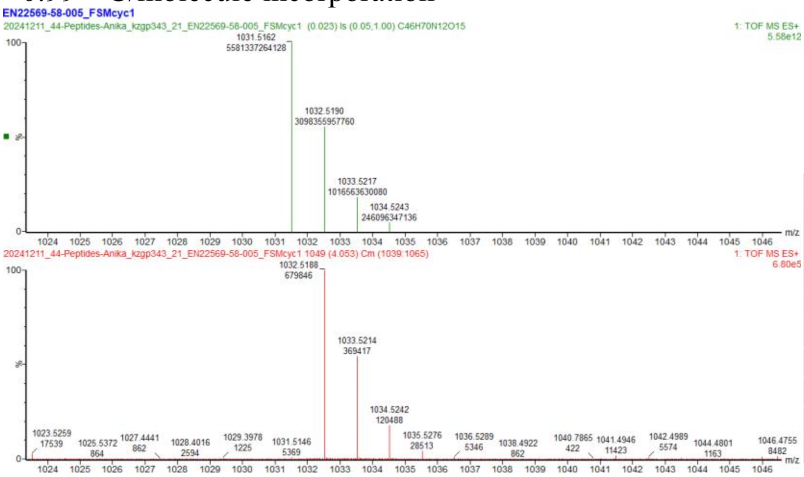

Results	
Relative amounts [%]	
unlabeled	0.8
1-label	99.8
2-label	-1.1
3-label	0.1
4-label	-0.1
5-label	0.9
6-label	-0.5

MSe analysis:

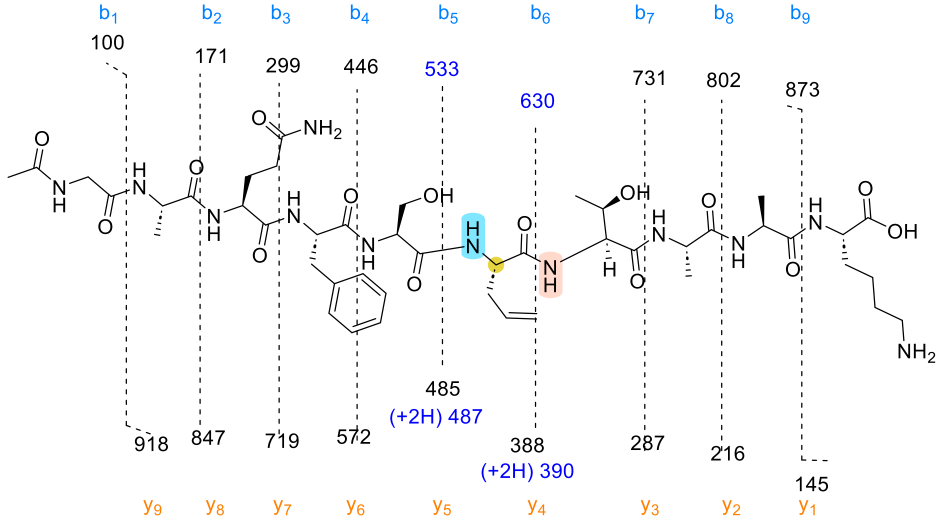

15SM, b5 calculated for $C_{24}H_{33}N_6O_8$+ 533.2354, found 533.2333.
15SM, b6 calculated for $C_{29}H_{40}N_7O_9$+ 630.2882, found 630.2701 (minor signal, accurate mass not found).
15SM, y4 calculated for $C_{16}H_{32}N_5O_6$+ 390.2347, found 390.2326.
15SM, y5 calculated for $C_{21}H_{39}N_6O_7^+$ 487.2875, found 487.2740 (minor signal, accurate mass not found).

[13C]15A'a

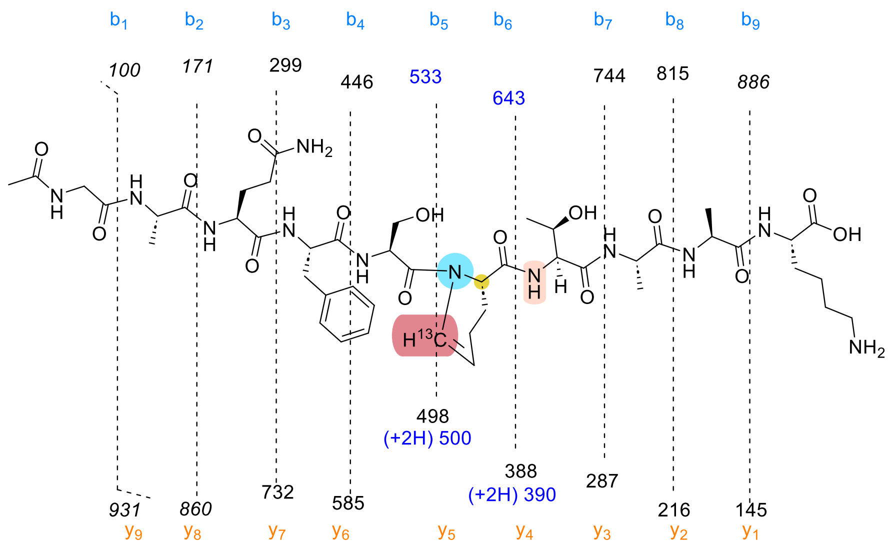

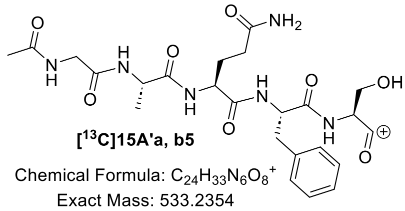

[13C]15A'a, b5

Chemical Formula: $C_{24}H_{33}N_6O_8^+$
Exact Mass: 533.2354

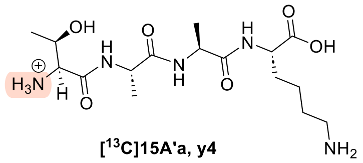

[13C]15A'a, y4

Chemical Formula: $C_{16}H_{32}N_5O_6^+$
Exact Mass: 390.2347

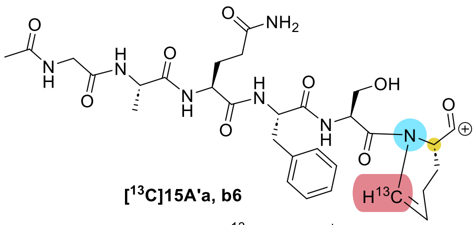

[13C]15A'a, b6

Chemical Formula: $C_{29}{}^{13}CH_{40}N_7O_9^+$
Exact Mass: 643.2916

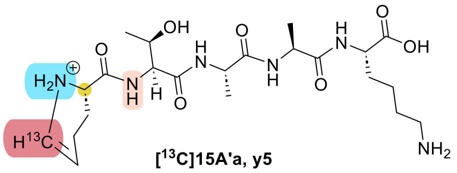

[13C]15A'a, y5

Chemical Formula: $C_{21}{}^{13}CH_{39}N_6O_7^+$
Exact Mass: 500.2908

[^{13}C]15A'a, b5 calculated for $C_{24}H_{33}N_6O_8^+$ 533.2354, found 533.2333.
[^{13}C]15A'a, b6 calculated for $C_{29}{}^{13}CH_{40}N_7O_9^+$ 643.2916, found 643.2788 (minor signal, accurate mass not found).
[^{13}C]15A'a, y4 calculated for $C_{16}H_{32}N_5O_6^+$ 390.2347, found 390.2326
[^{13}C]15A'a, y5 calculated for $C_{21}{}^{13}CH_{39}N_6O_7^+$ 500.2908, found 500.2908.
→the presence of fragment **b6**, in combination with b5 fragmentation indicates six-membered ring formation

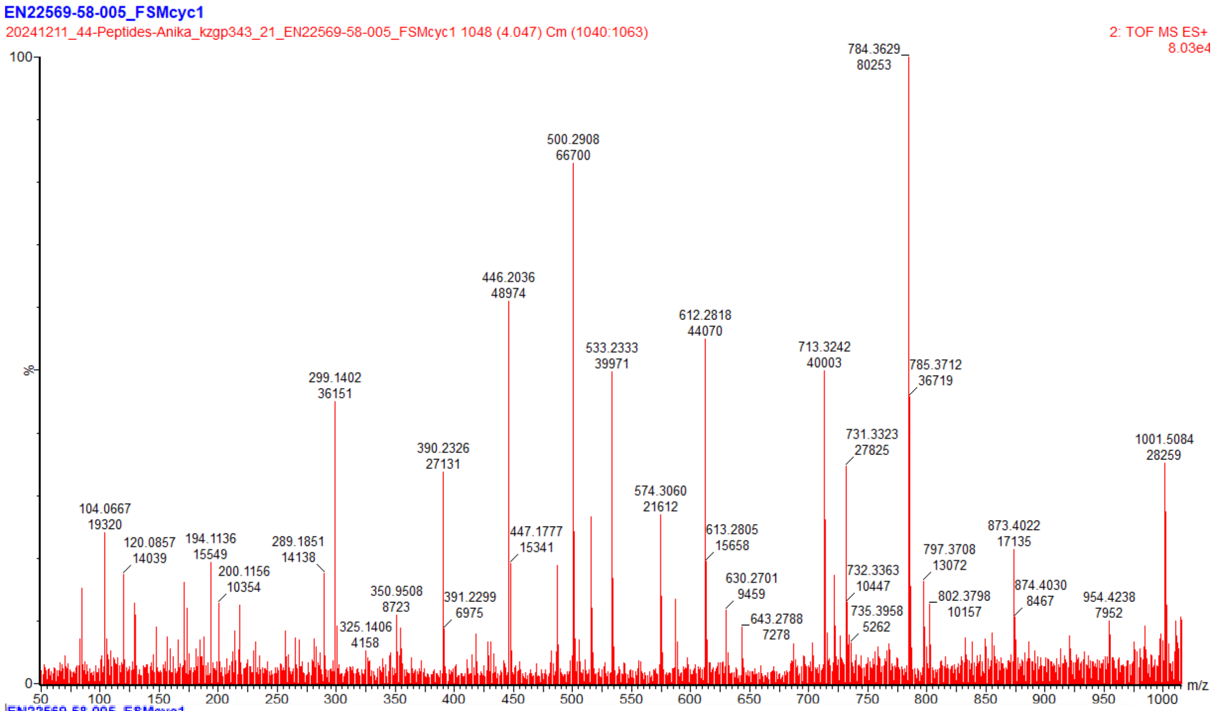
EN22569-58-005_FSMcyc1
20241211_44-Peptides-Anika_kzgp343_21_EN22569-58-005_FSMcyc1 1048 (4.047) Cm (1040:1063)
2: TOF MS ES+
8.03e4
784.3629
80253
500.2908
66700
446.2036
48974
612.2818
44070
533.2333
39971
713.3242
40003
785.3712
36719
299.1402
36151
390.2326
27131
731.3323
27825
1001.5084
28259
574.3060
21612
104.0667
19320
m/z

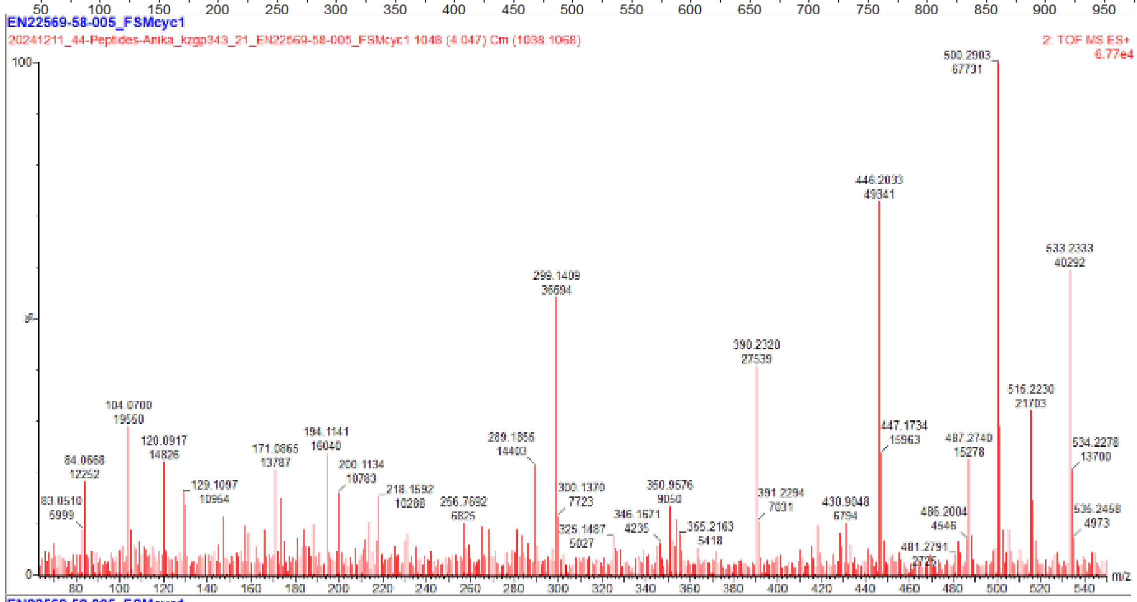
EN22569-58-005_FSMcyc1
20241211_44-Peptides-Anika_kzgp343_21_EN22569-58-005_FSMcyc1 1048 (4.047) Cm (1038:1068)
2: TOF MS ES+
6.77e4
500.2903
67731
446.2033
49341
533.2333
40292
299.1409
36694
390.2320
27539
515.2230
21703
m/z

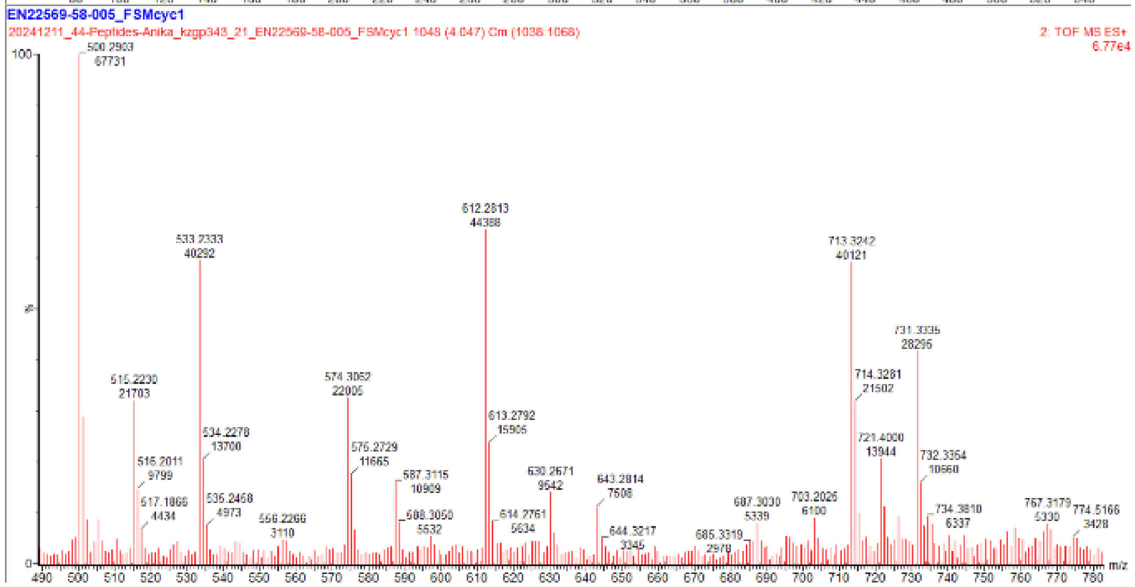
EN22569-58-005_FSMcyc1
20241211_44-Peptides-Anika_kzgp343_21_EN22569-58-005_FSMcyc1 1048 (4.047) Cm (1038:1068)
2: TOF MS ES+
6.77e4
500.2903
67731
612.2813
44388
713.3242
40121
533.2333
40292
731.3335
28296
574.3062
22005
515.2230
21703
714.3281
21502
m/z

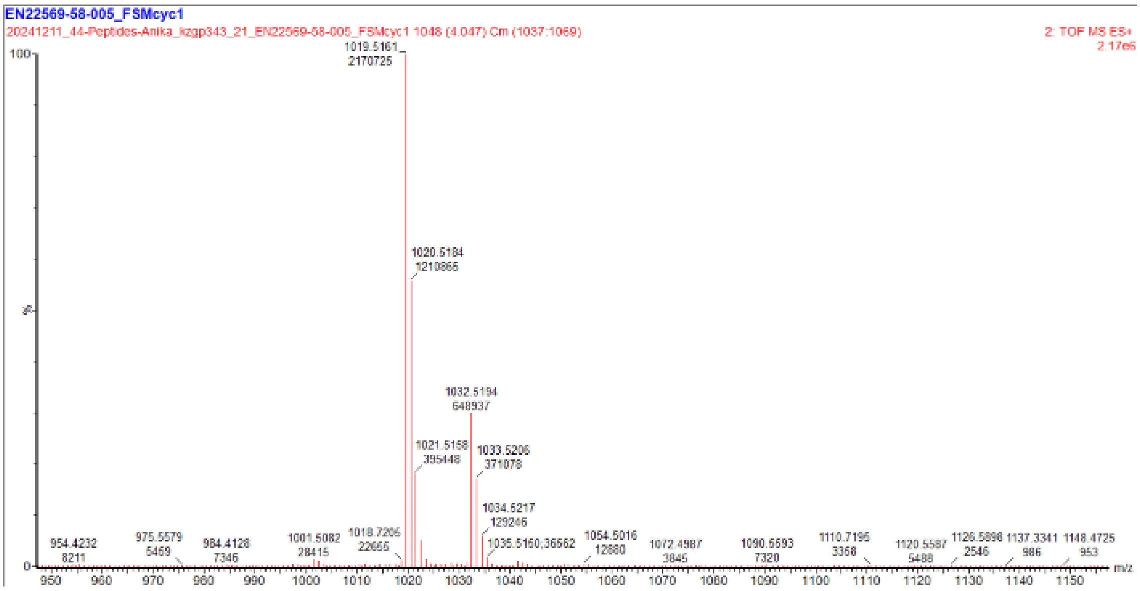

NMR of purified product (contains also **15SM)**: ^{1}H-NMR (2x), ^{13}C-NMR, HSQC, HMBC, COSY, ROESY, TOCSY, NHSQC, NHMBC

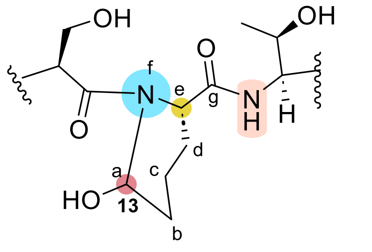

[^{13}C]15A'a

a: ^{13}C at 82.75 ppm, ^{1}H medium at 5.10 ppm (HSQC), split protons at 5.23 ppm and 4.97 ppm (HMBC)

^{1}H-NMR (600 MHz, DMSO-d6) (phase correction incorrect)

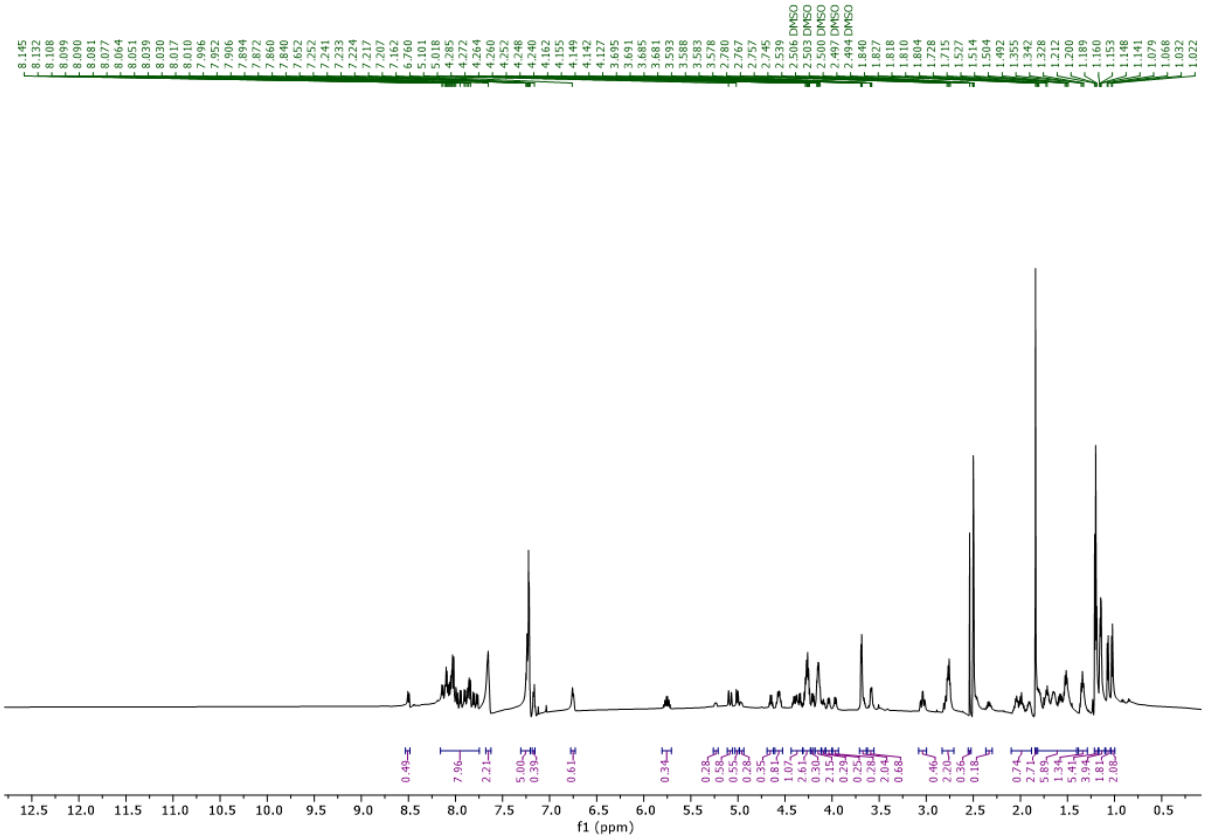

^{1}H-NMR (500 MHz, DMSO-d6), correct phase but incorrect baseline correction in 3.5-5 ppm region, acquired with other spectrometer

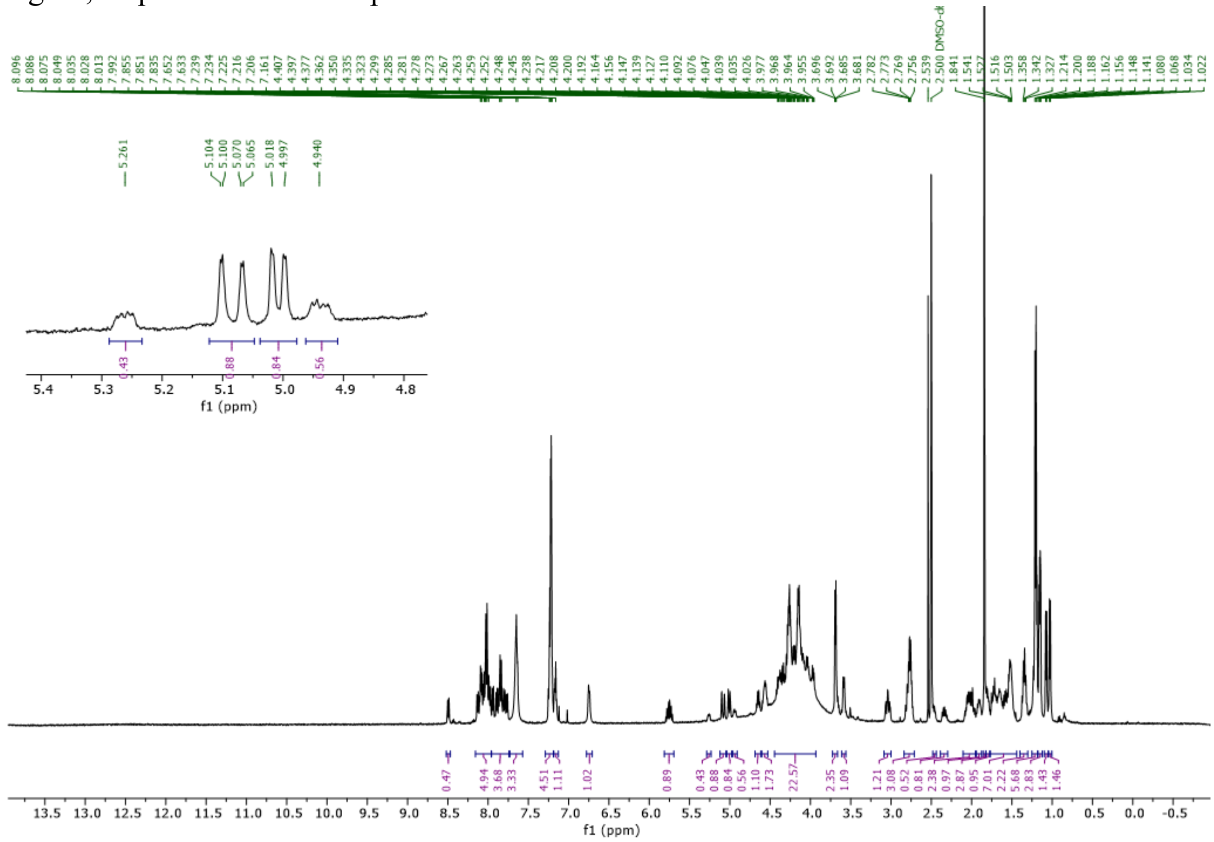

^{13}C-NMR (151 MHz, DMSO-d6)

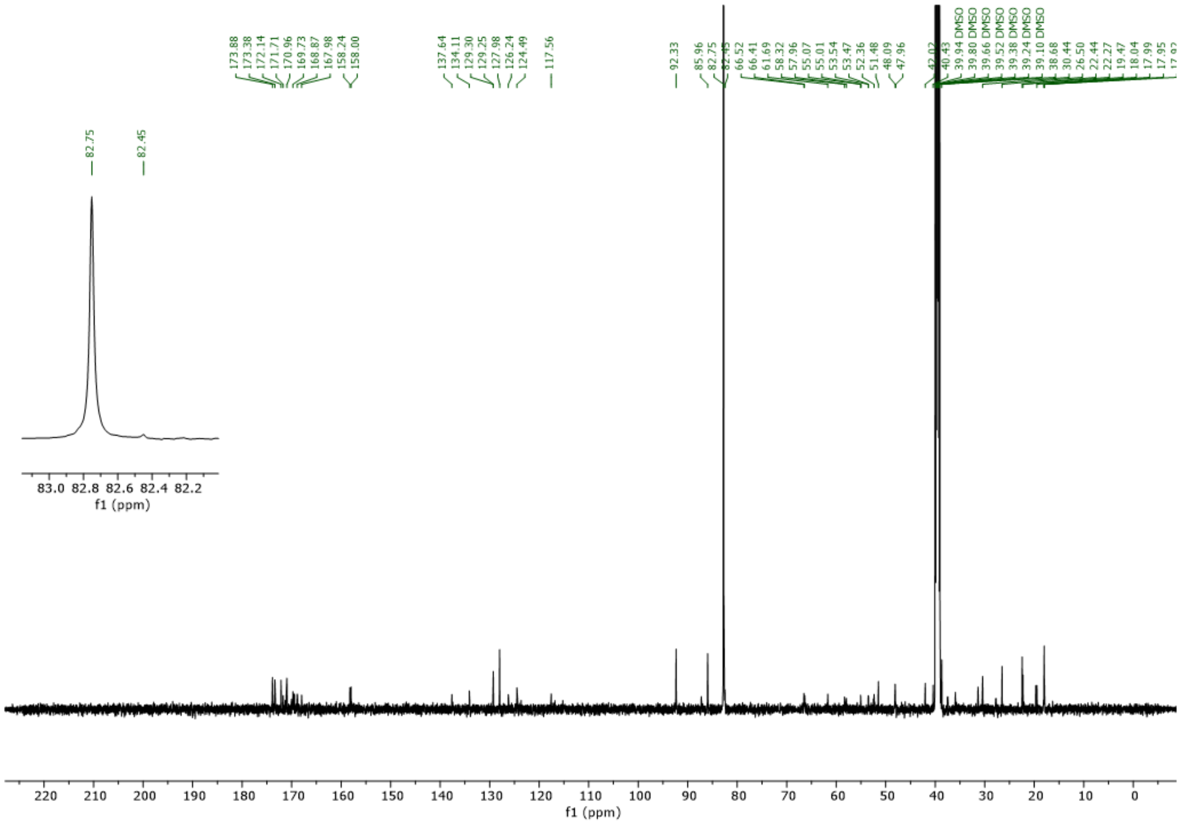

HSQC (600 MHz, DMSO-d6)

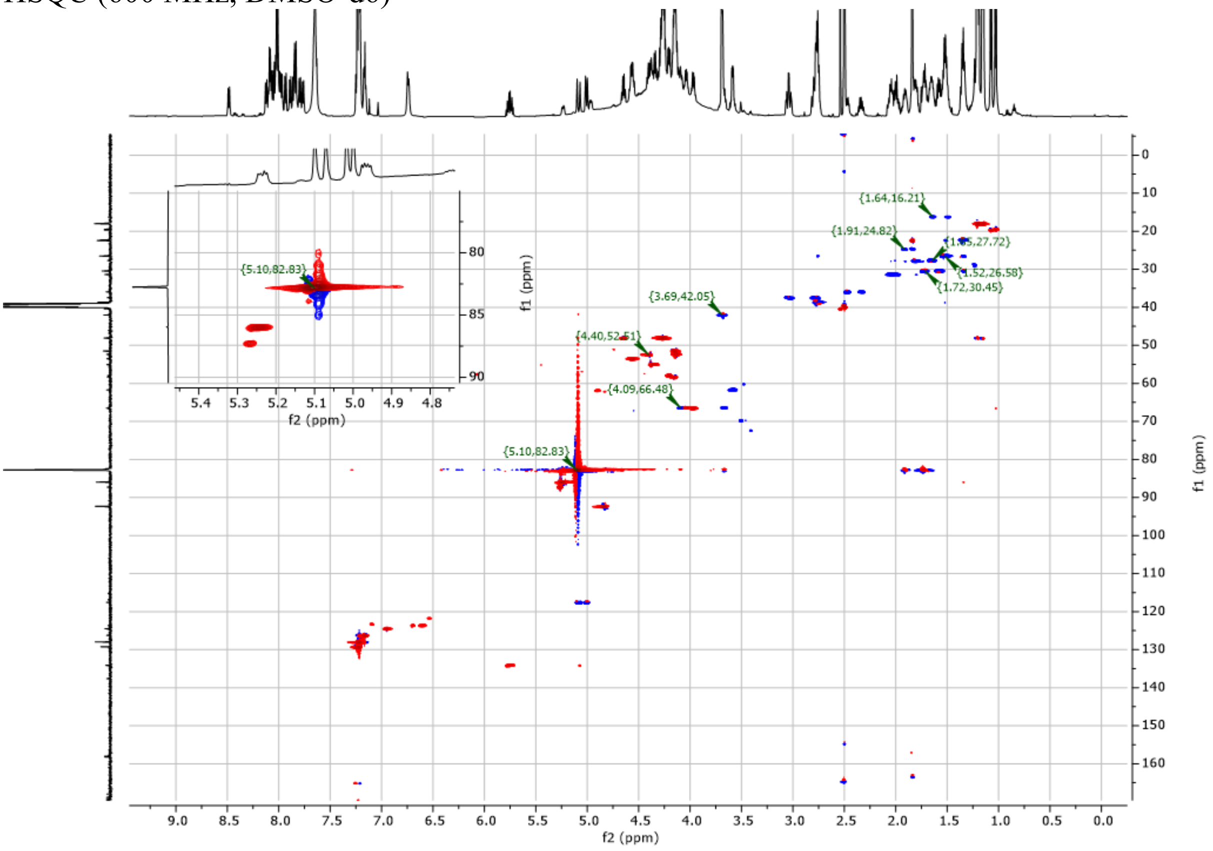

HMBC (600 MHz, DMSO-d6)

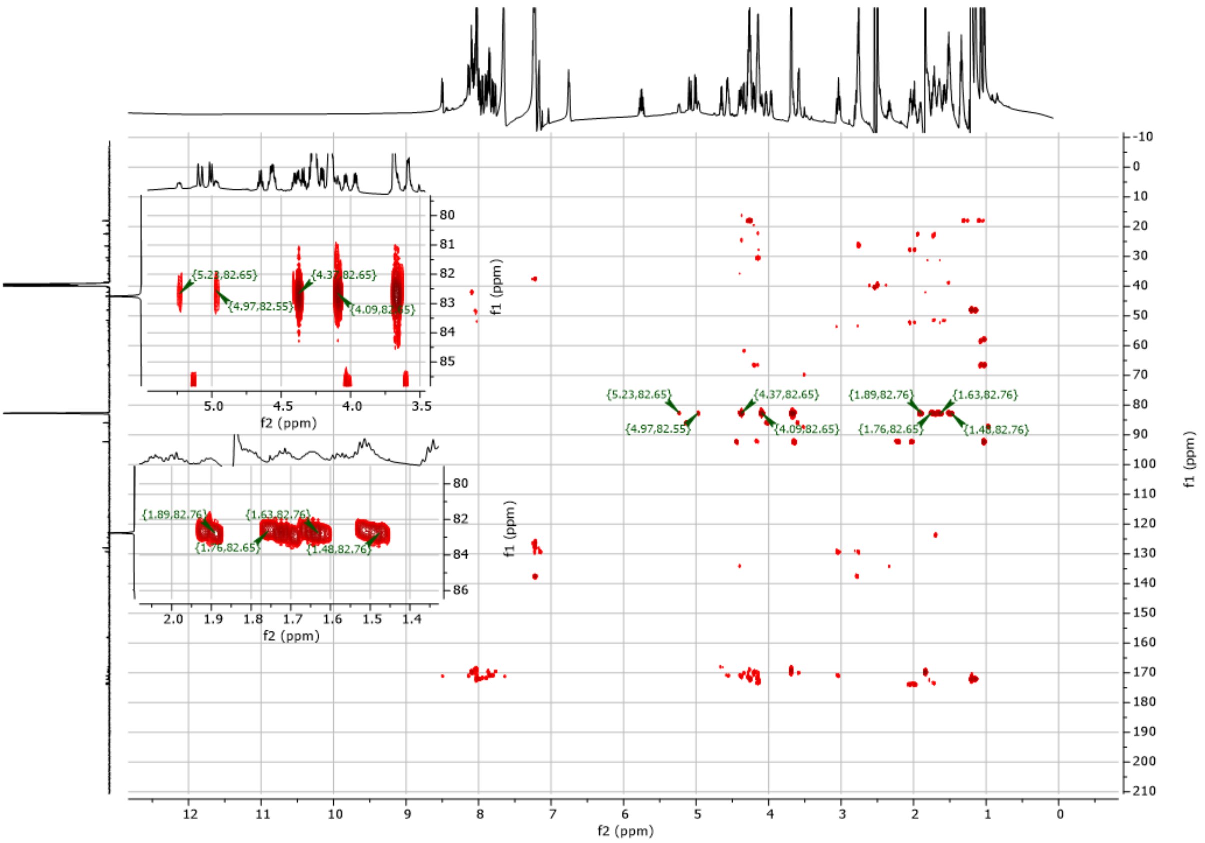

COSY (500 MHz, DMSO-d6)

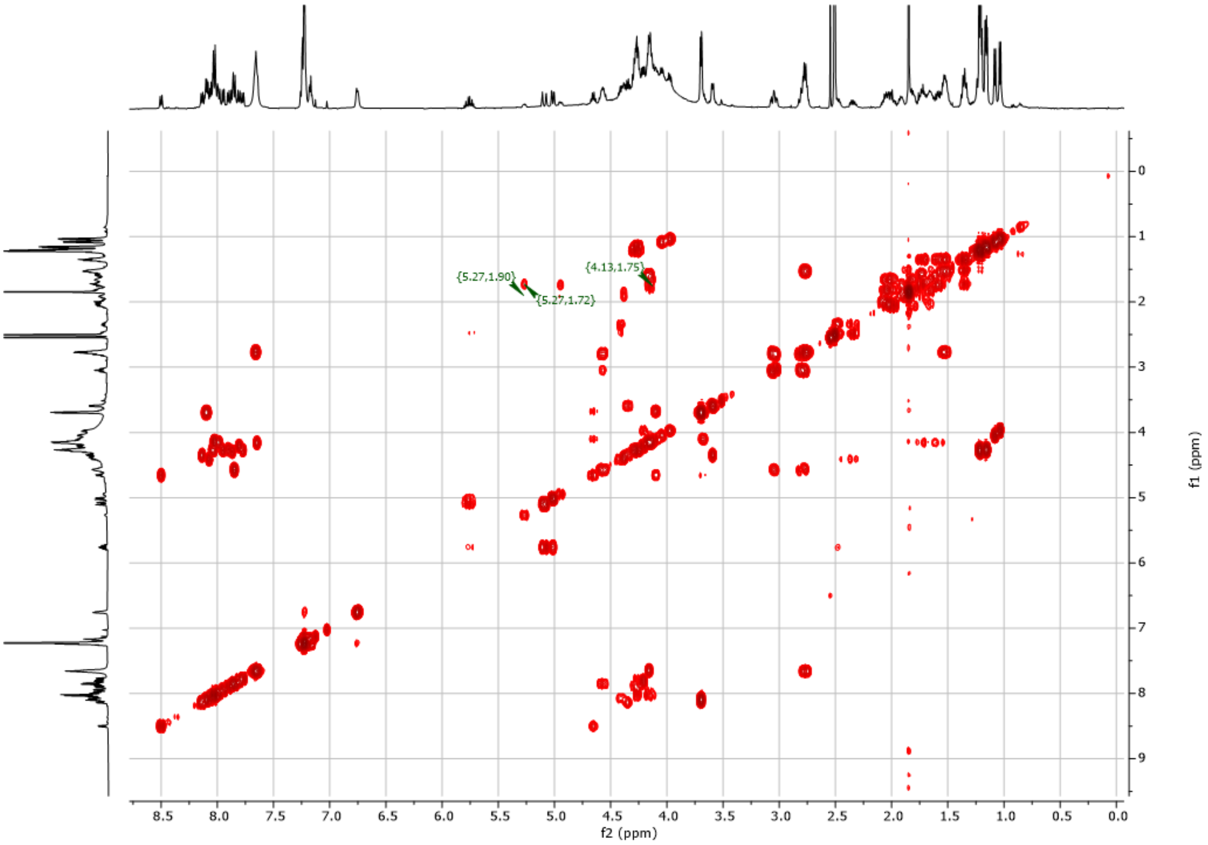

ROESY (500 MHz, DMSO-d6)

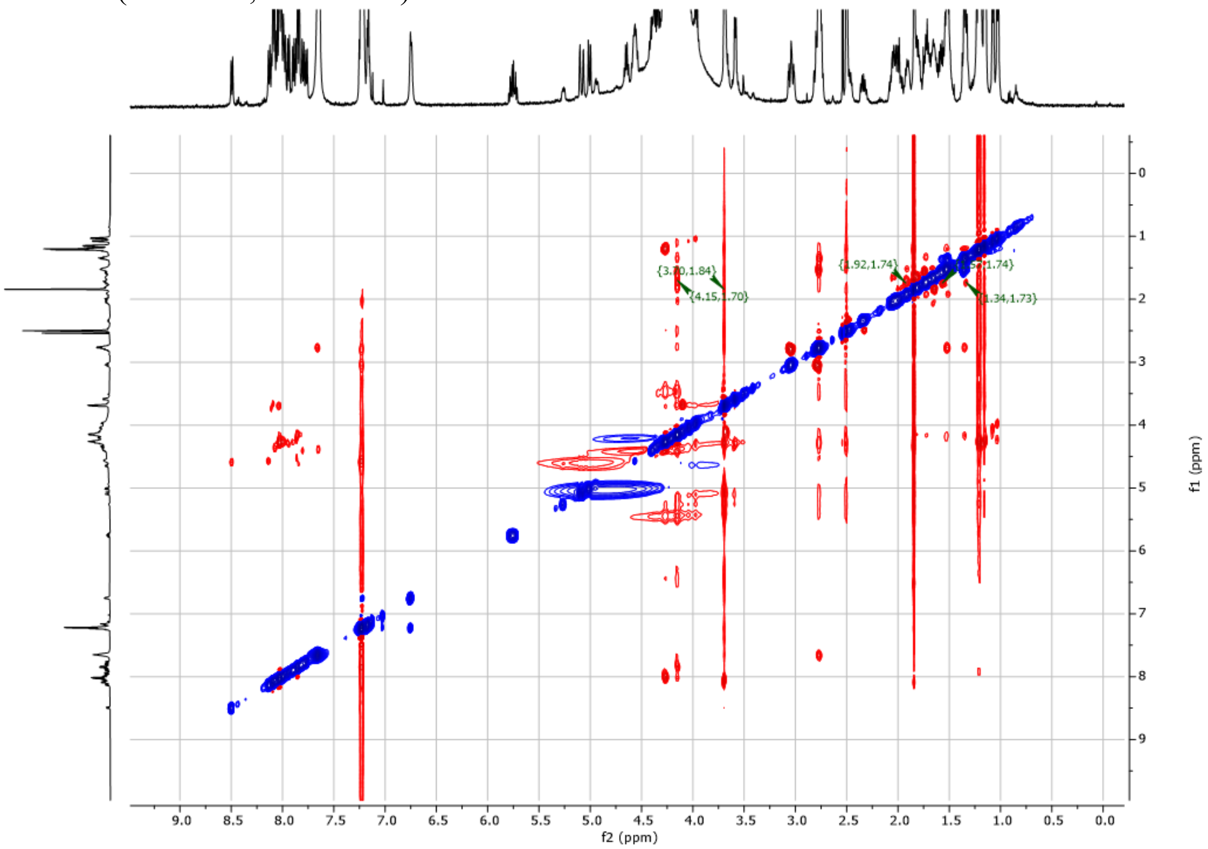

TOCSY (600 MHz, DMSO-d6)

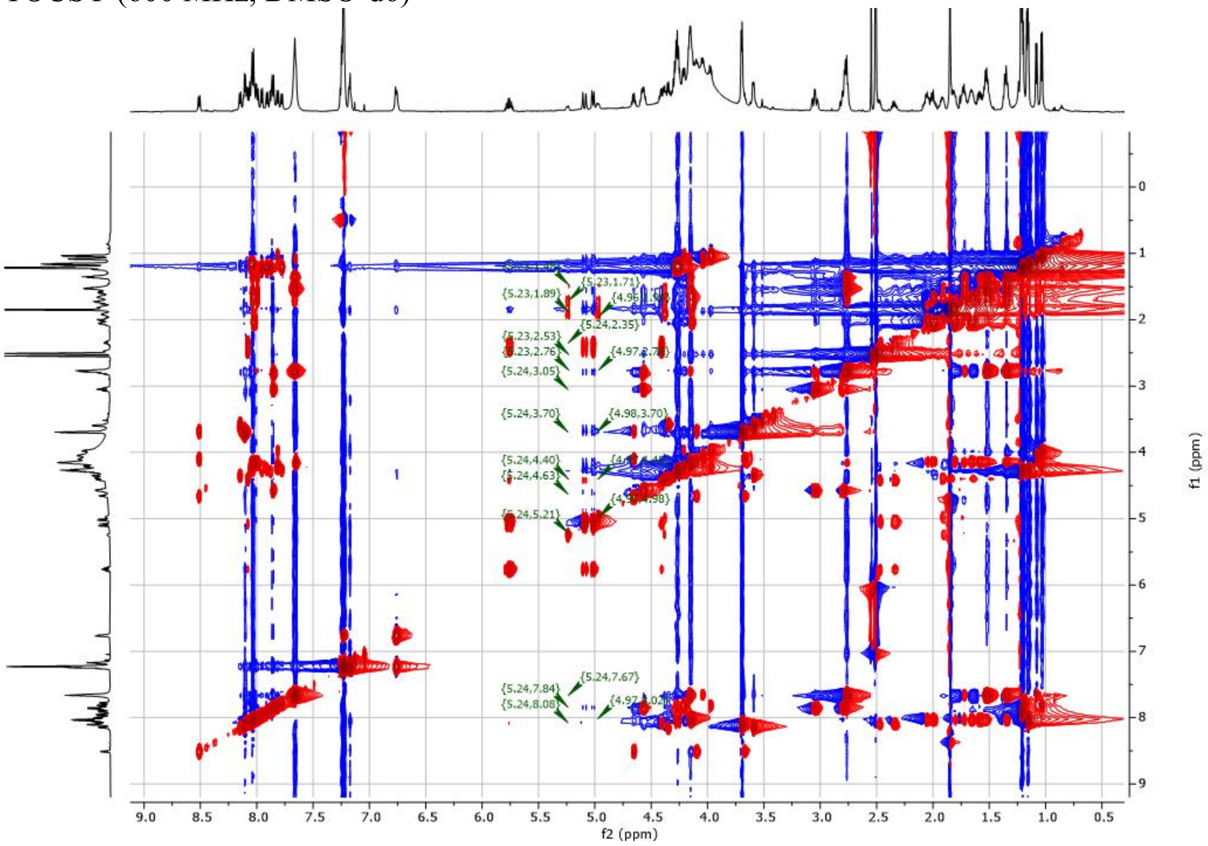

NHSQC (600 MHz, DMSO-d6)

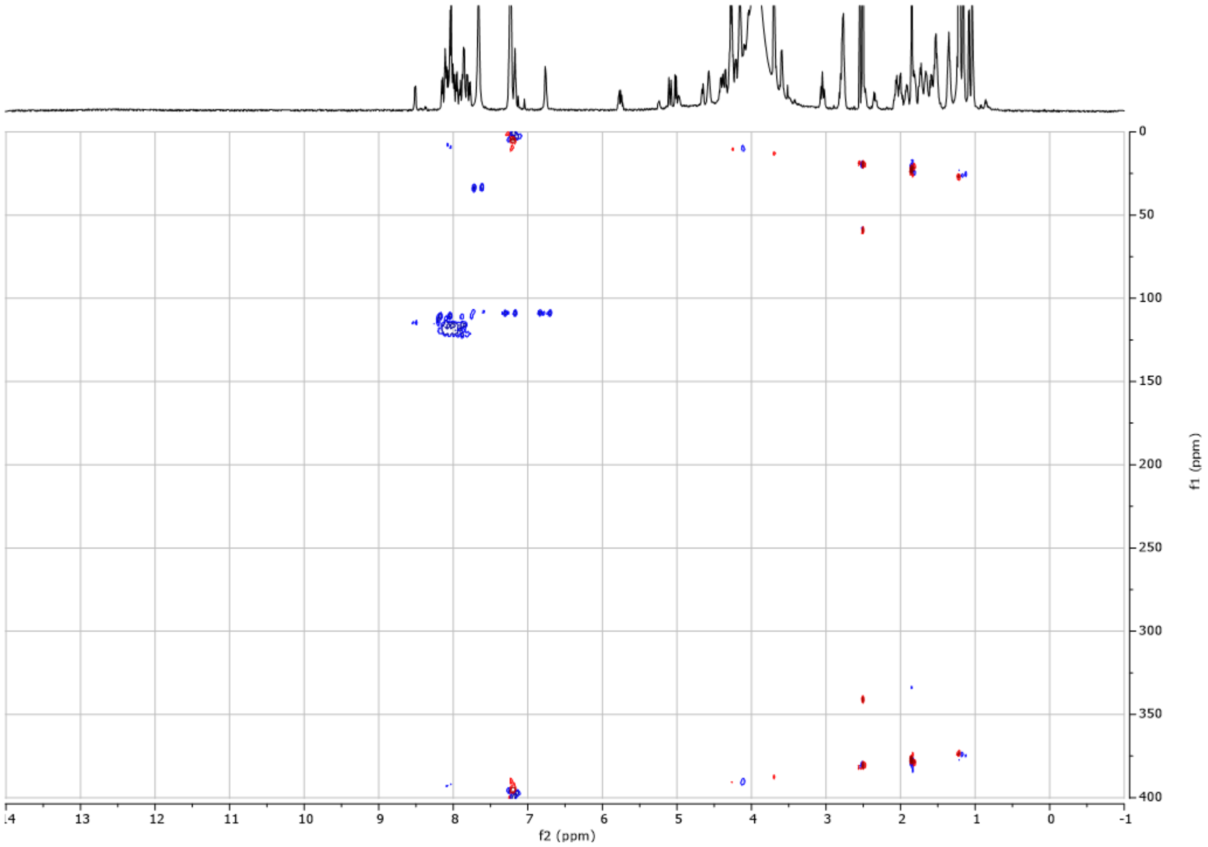

NHMBC (600 MHz, DMSO-d6)

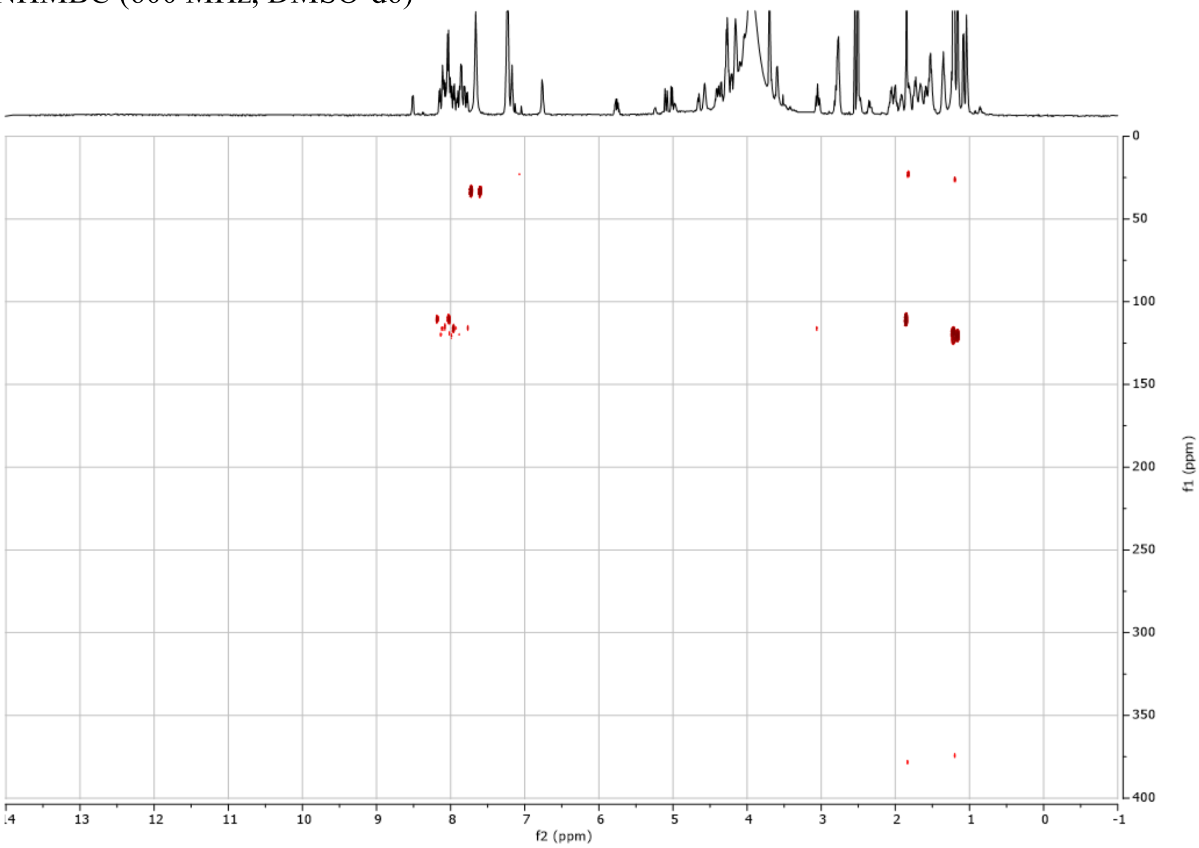

15A’b, proposed structure (Cleavage ii)

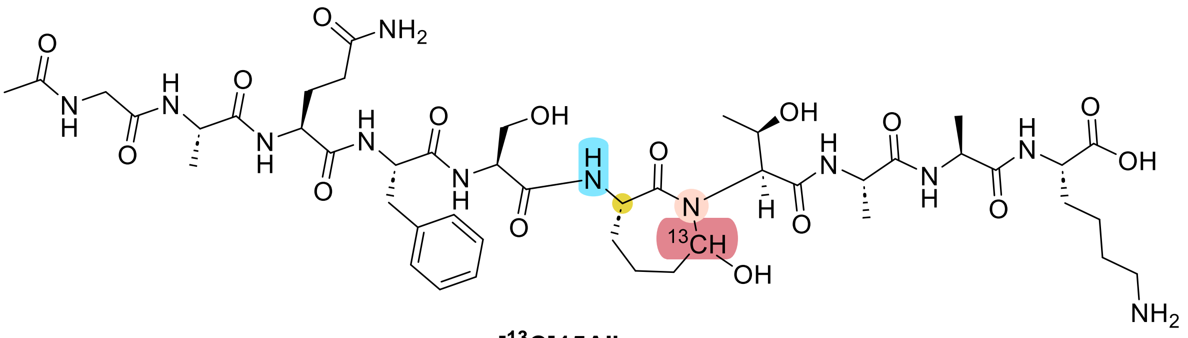

[^{13}C]15A'b

Chemical Formula: $C_{45}{}^{13}CH_{72}N_{12}O_{16}$

Exact Mass: 1049.5223

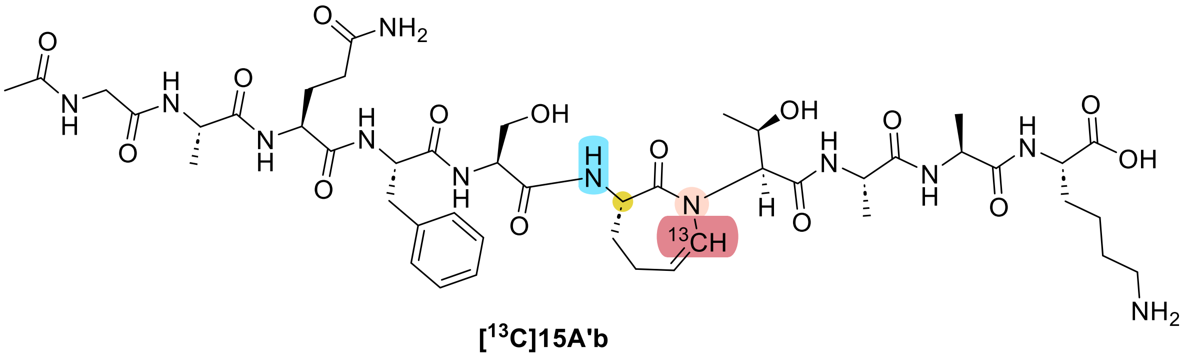

[^{13}C]15A'b

Chemical Formula: $C_{45}{}^{13}CH_{70}N_{12}O_{15}$

Exact Mass: 1031.5117

LC-MS of crude: <54% UV-purity of **[^{13}C]15A’a** (co-elutes with **15SM**), and ca. 30% UV-purity of **[^{13}C]15A’b**

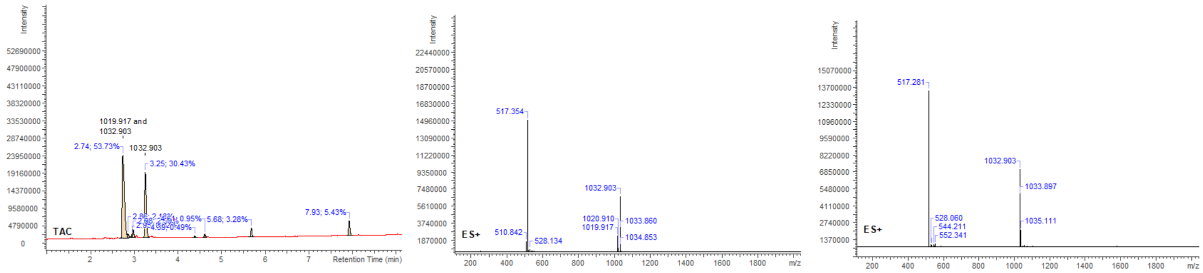

Isotope distribution comparison: Predicted non-labeled vs labeled peptide, 0.99 ^{13}C/molecule incorporation

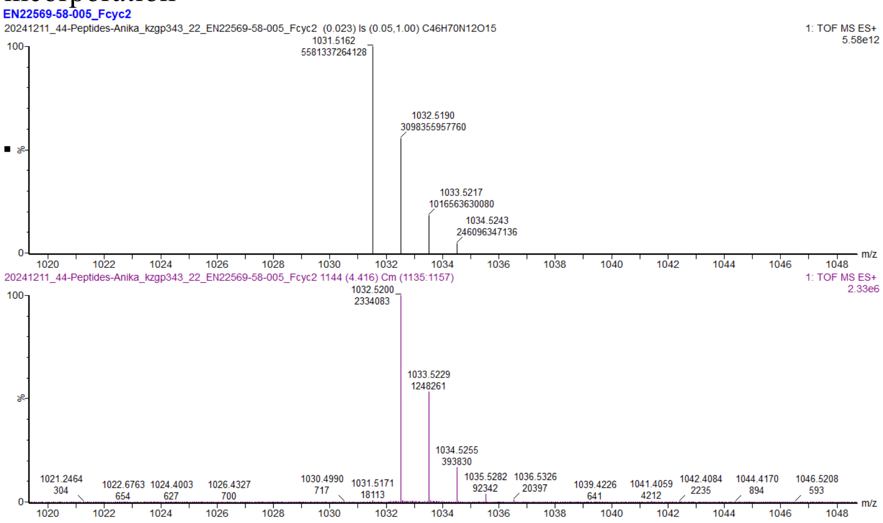

Results	
Relative amounts [%]	
unlabeled	0.8
1-label	100.9
2-label	-2.0
3-label	-0.2
4-label	0.0
5-label	1.0
6-label	-0.5

LC-MS of purified product: ca. >99% UV-purity

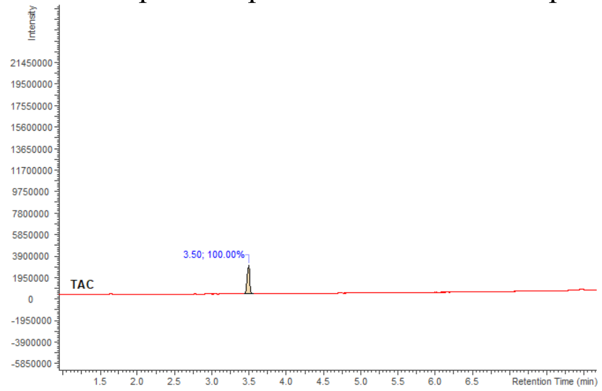

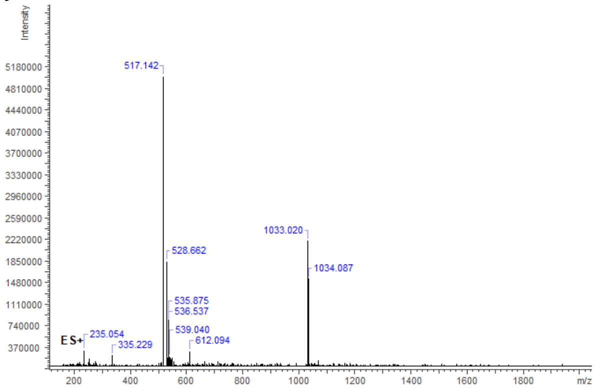

MSe analysis:

[^{13}C]15A'b

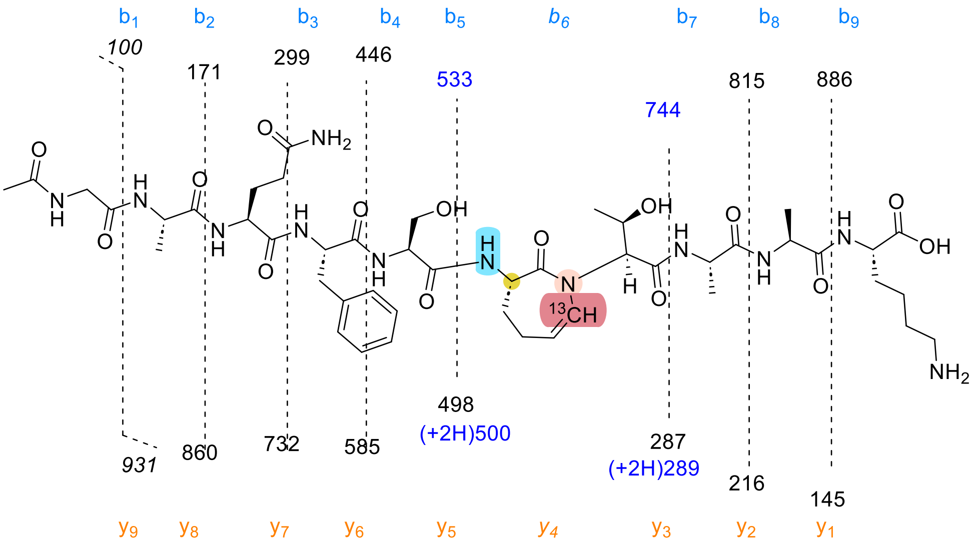

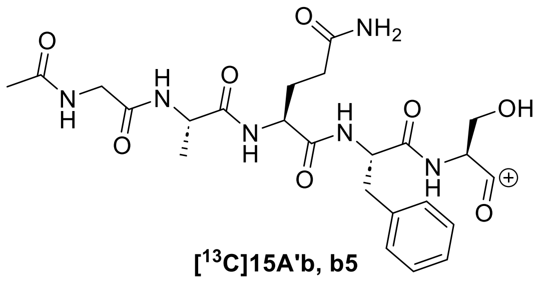

[^{13}C]15A'b, b5

Chemical Formula: $C_{24}H_{33}N_6O_8^+$

Exact Mass: 533.2354

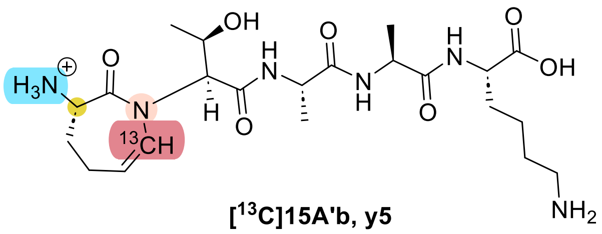

[^{13}C]15A'b, y5

Chemical Formula: $C_{21}{}^{13}CH_{39}N_6O_7^+$

Exact Mass: 500.2908

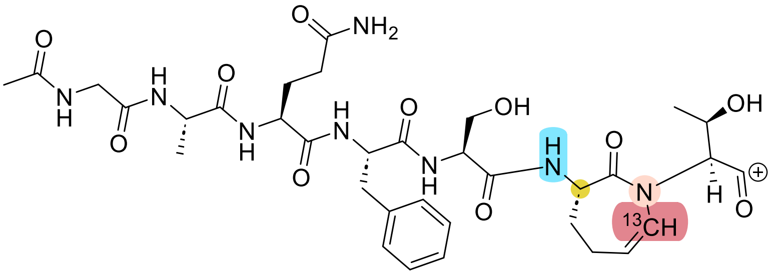

[^{13}C]15A'b, b7

Chemical Formula: $C_{33}{}^{13}CH_{47}N_8O_{11}^+$

Exact Mass: 744.3392

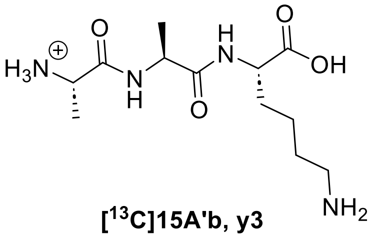

[^{13}C]15A'b, y3

Chemical Formula: $C_{12}H_{25}N_4O_4^+$

Exact Mass: 289.1870

[^{13}C]15A'b, b5 calculated for $C_{24}H_{33}N_6O_8^+$ 533.2354, found 533.2424.
[^{13}C]15A'b, b7 calculated for $C_{33}{}^{13}CH_{47}N_8O_{11}^+$ 744.3392, found 744.3523 (minor signal).
[^{13}C]15A'b, y5 calculated for $C_{21}{}^{13}CH_{39}N_6O_7^+$ 500.2908, found 500.2886.
[^{13}C]15A'b, y3 calculated for $C_{12}H_{25}N_4O_4^+$ 289.1870, found 289.1903 (minor signal).

In this spectrum, there is no signal as in **[^{13}C]15A'a, b5** (calculated for $C_{29}{}^{13}CH_{40}N_7O_9^+$ 643.2916, found 643.2788) which was characteristic for the six-membered ring formation; in this case MS/MS analysis could have likely helped in confirming the seven-membered ring cyclization; the presence of b6 and b7; as well as y5 and y3 however support our proposal of a seven-membered ring system.

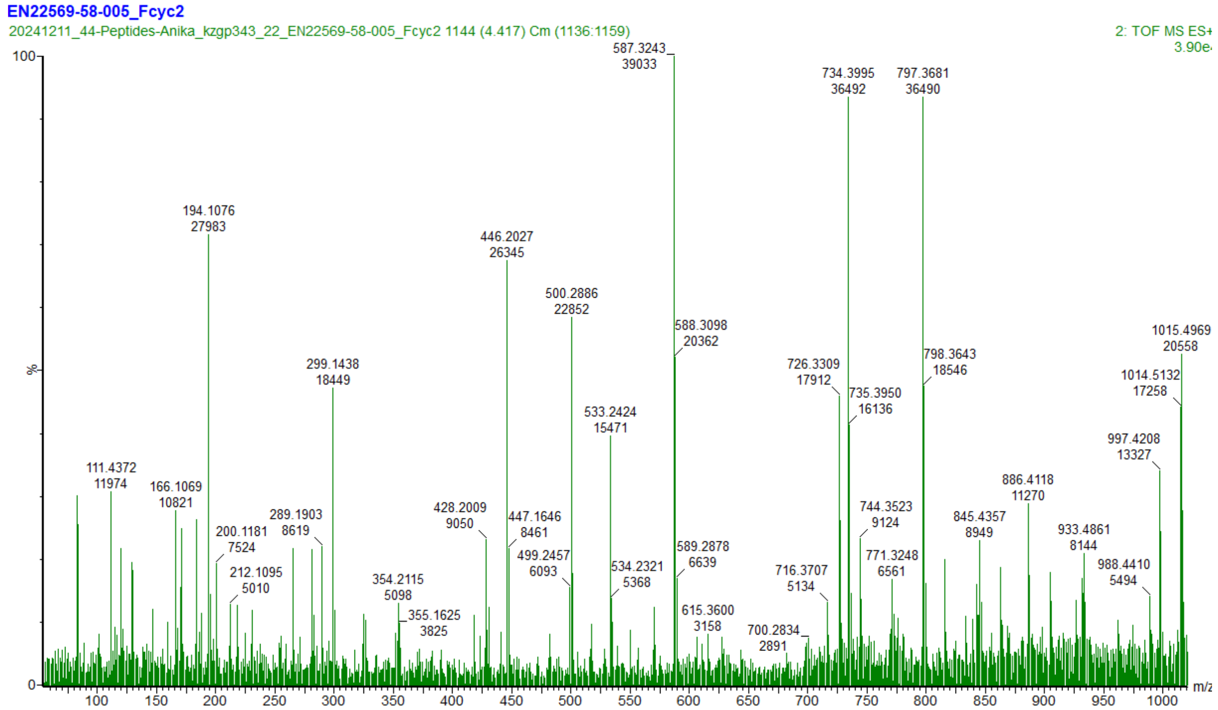

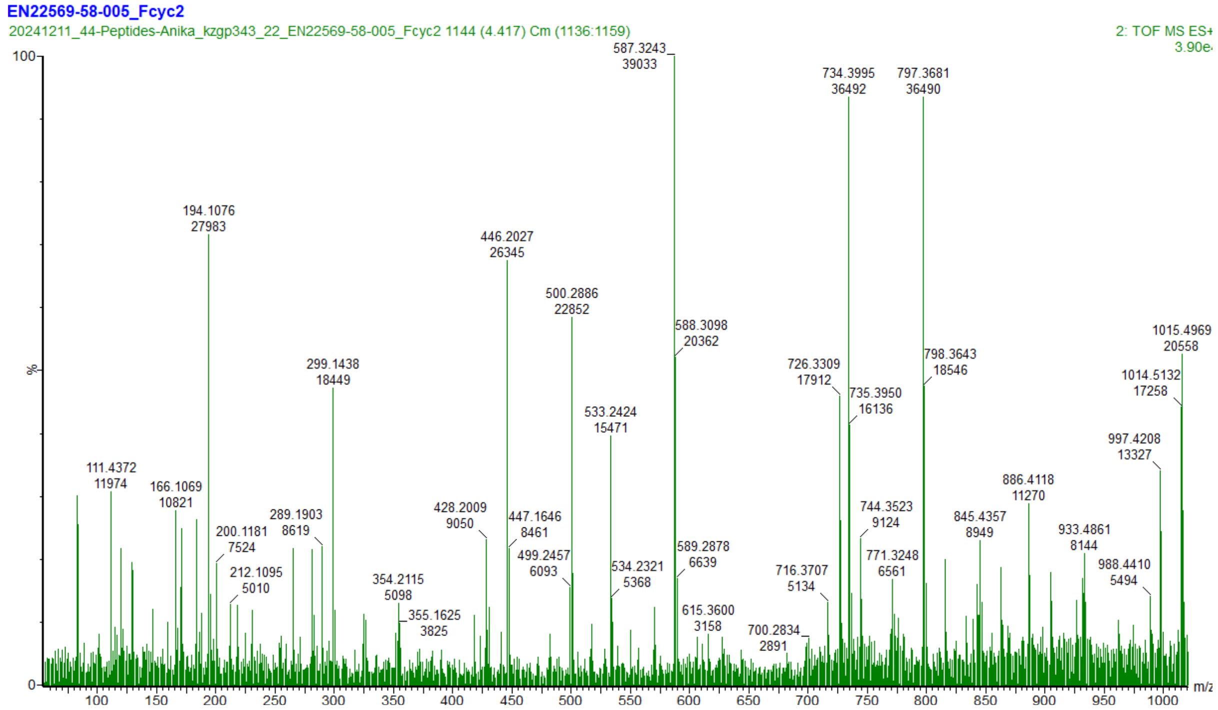
EN22569-58-005_Fcyc2
20241211_44-Peptides-Anika_kzgp343_22_EN22569-58-005_Fcyc2 1144 (4.417) Cm (1136:1159)
2: TOF MS ES+
3.90e4

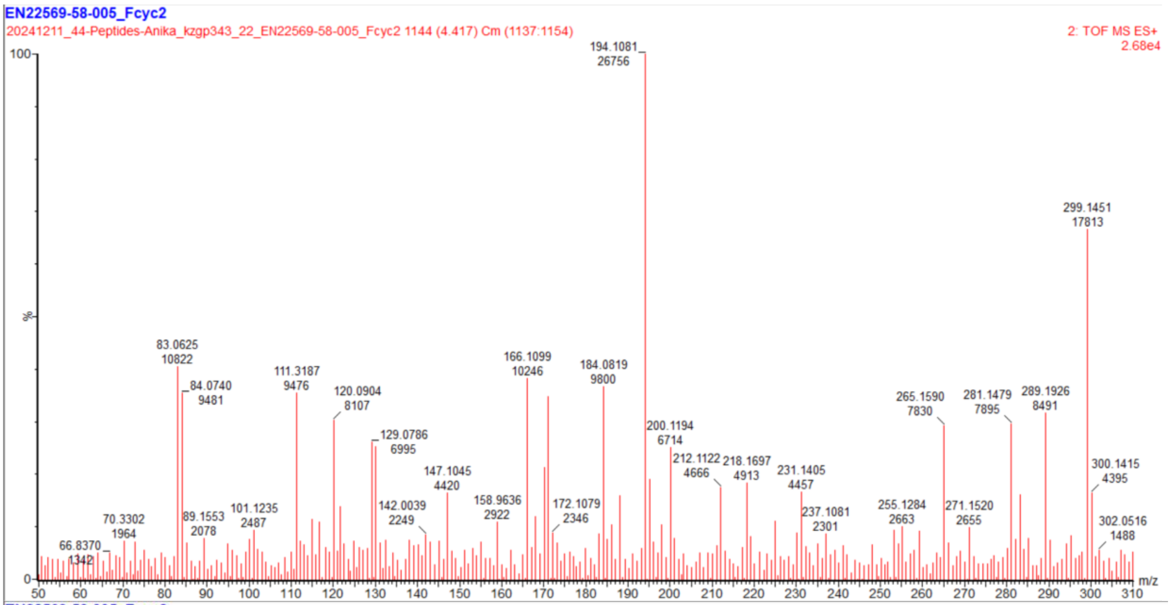
EN22569-58-005_Fcyc2
20241211_44-Peptides-Anika_kzgp343_22_EN22569-58-005_Fcyc2 1144 (4.417) Cm (1137:1154)
2: TOF MS ES+
2.68e4
194.1081
26756
299.1451
17813
83.0625
10822
84.0740
9481
111.3187
9476
120.0904
8107
129.0786
6995
166.1099
10246
184.0819
9800
200.1194
6714
212.1122
4666
218.1697
4913
231.1405
4457
265.1590
7830
281.1479
7895
289.1926
8491
300.1415
4395
147.1045
4420
142.0039
2249
158.9636
2922
172.1079
2346
237.1081
2301
255.1284
2663
271.1520
2655
302.0516
1488
101.1235
2487
89.1553
2078
70.3302
1964
66.8370
1342
m/z

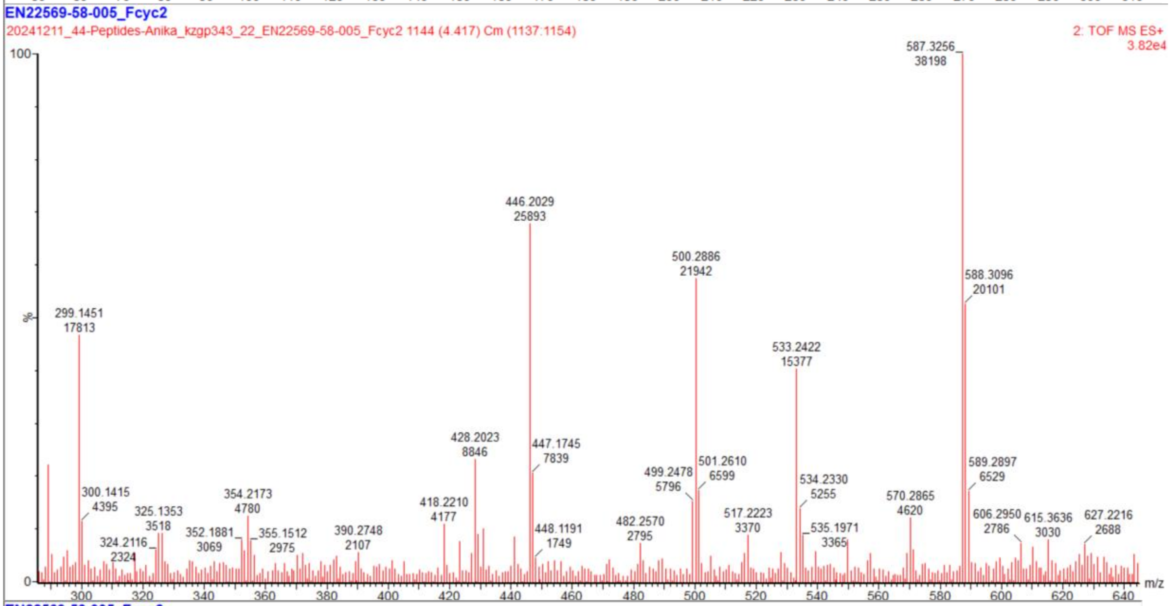
EN22569-58-005_Fcyc2
20241211_44-Peptides-Anika_kzgp343_22_EN22569-58-005_Fcyc2 1144 (4.417) Cm (1137:1154)
2: TOF MS ES+
3.82e4
587.3256
38198
446.2029
25893
500.2886
21942
588.3096
20101
299.1451
17813
533.2422
15377
428.2023
8846
447.1745
7839
499.2478
5796
501.2610
6599
589.2897
6529
534.2330
5255
570.2865
4620
300.1415
4395
354.2173
4780
418.2210
4177
325.1353
3518
352.1881
3069
355.1512
2975
324.2116
2324
390.2748
2107
448.1191
1749
482.2570
2795
517.2223
3370
535.1971
3365
606.2950
2786
615.3636
3030
627.2216
2688
m/z

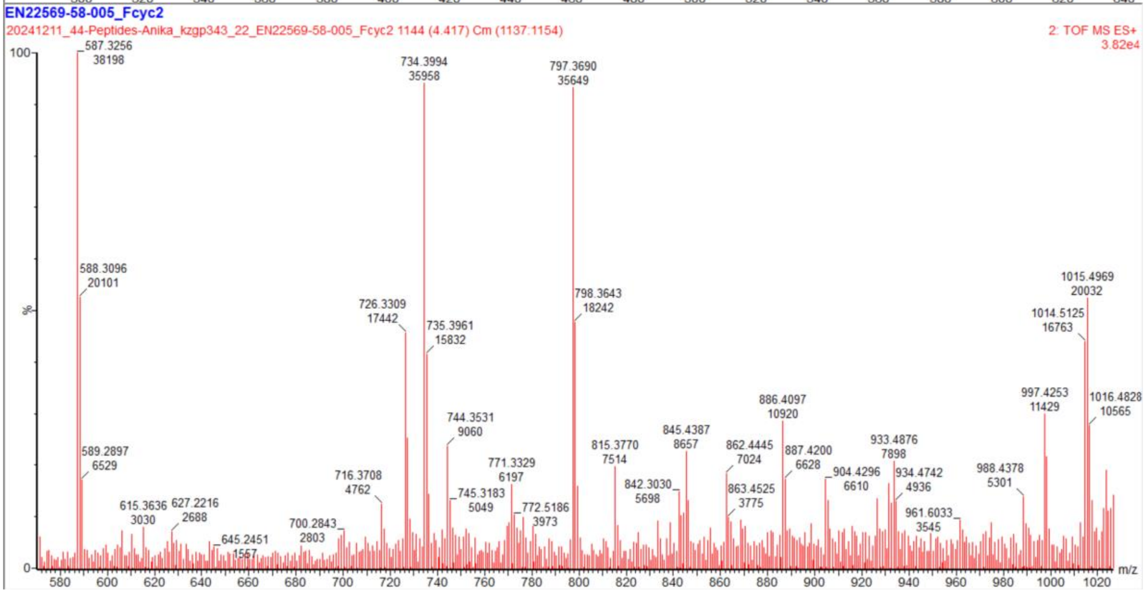
EN22569-58-005_Fcyc2
20241211_44-Peptides-Anika_kzgp343_22_EN22569-58-005_Fcyc2 1144 (4.417) Cm (1137:1154)
2: TOF MS ES+
3.82e4
587.3256
38198
734.3994
35958
797.3690
35649
588.3096
20101
726.3309
17442
735.3961
15832
798.3643
18242
1015.4969
20032
1014.5125
16763
997.4253
11429
1016.4828
10565
886.4097
10920
744.3531
9060
845.4387
8657
815.3770
7514
862.4445
7024
887.4200
6628
933.4876
7898
904.4296
6610
934.4742
4936
988.4378
5301
771.3329
6197
716.3708
4762
745.3183
5049
772.5186
3973
842.3030
5698
863.4525
3775
961.6033
3545
589.2897
6529
615.3636
3030
627.2216
2688
645.2451
1557
700.2843
2803
m/z

NMR of purified product: ^{1}H-NMR, ^{13}C-NMR, HSQC, HMBC, COSY, ROESY, TOCSY, NHSQC, NHMBC

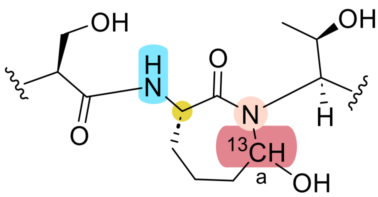

[^{13}C]15A'b

a: ^{13}C at 86.41 ppm, ^{1}H medium at 5.25 ppm (HSQC), split protons likely at 5.4 ppm and 5.1 ppm (splitting not shown in HMBC)

^{1}H-NMR (600 MHz, DMSO-d6)

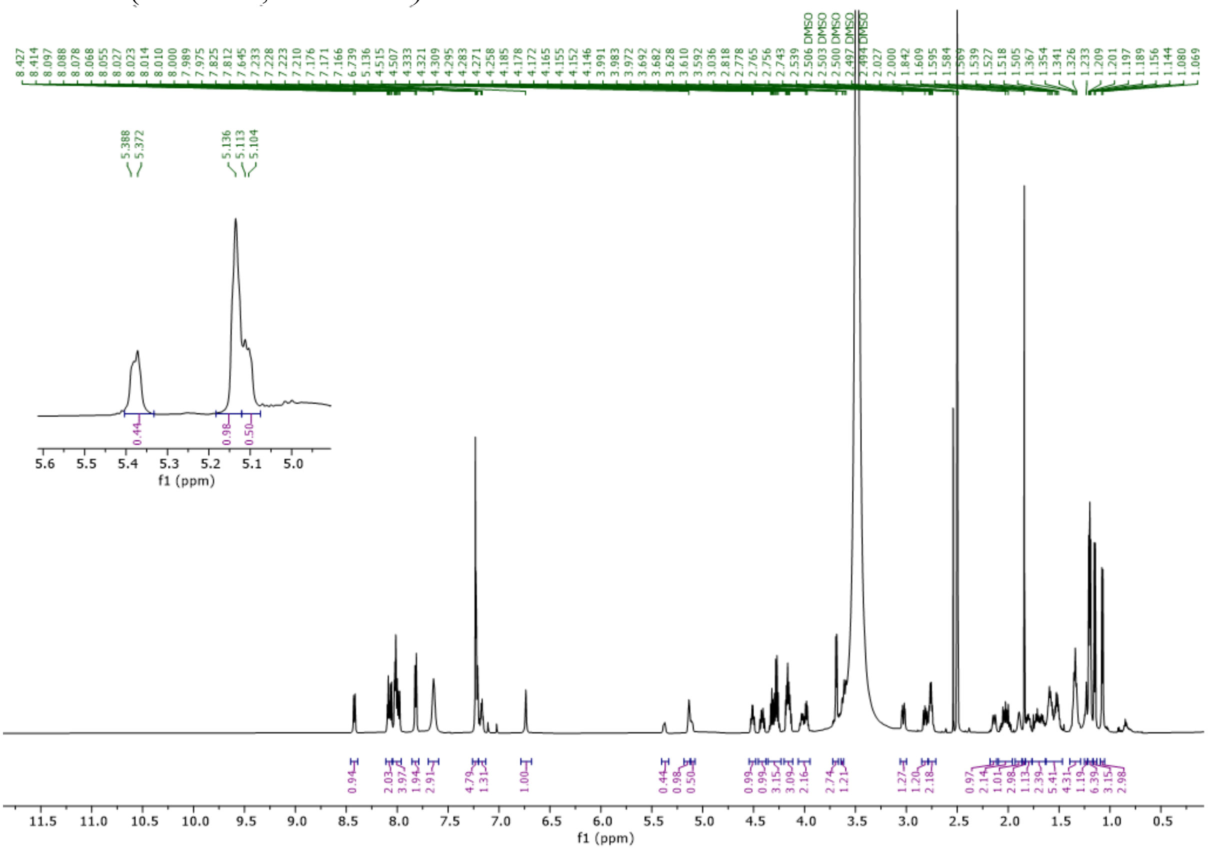

^{13}C-NMR (126 MHz, DMSO-d6)

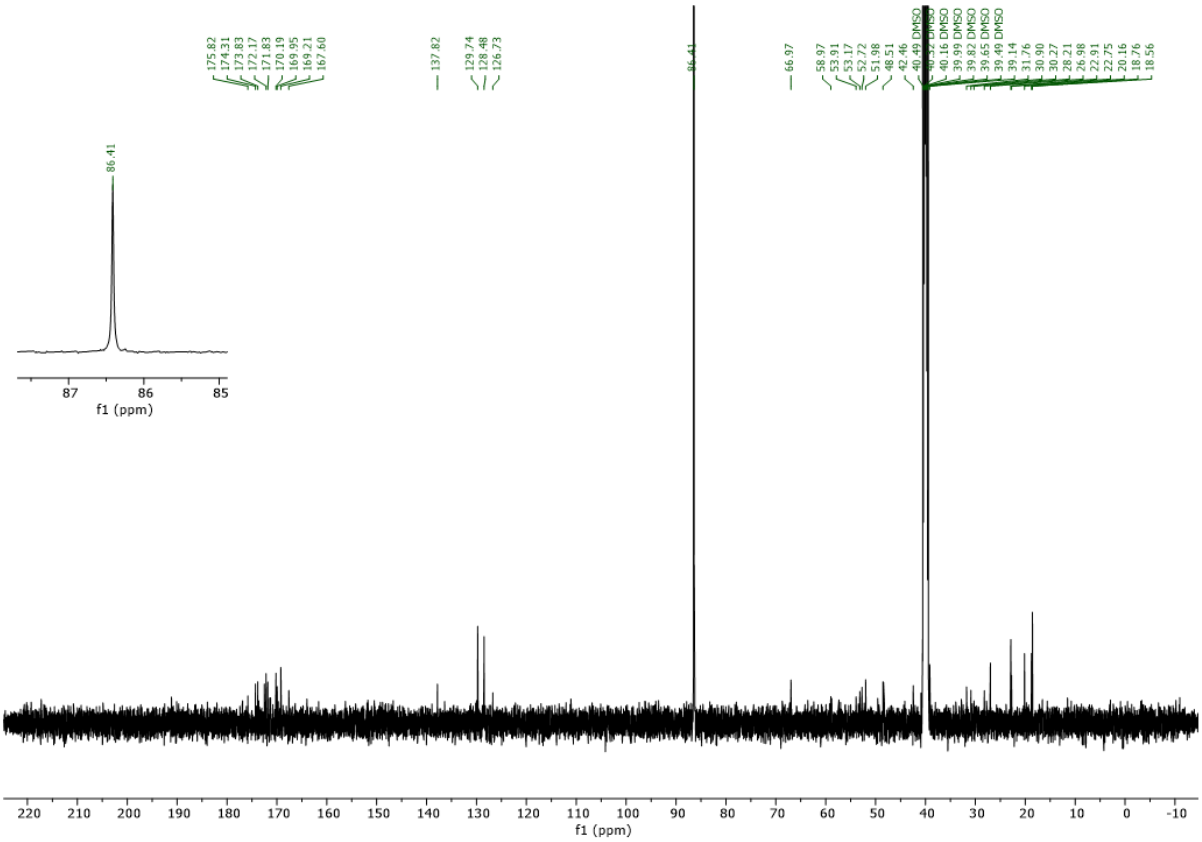

HSQC (500 MHz, DMSO-d6)

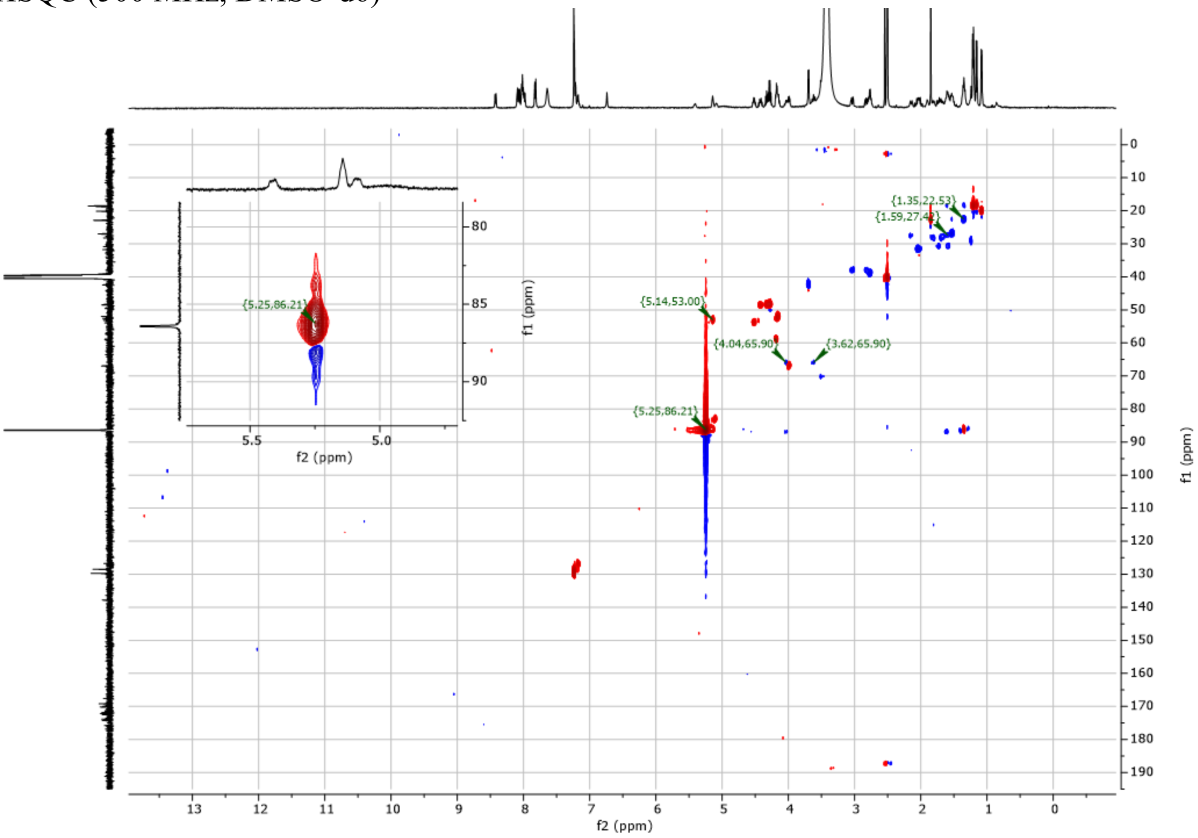

HMBC (500 MHz, DMSO-d6)

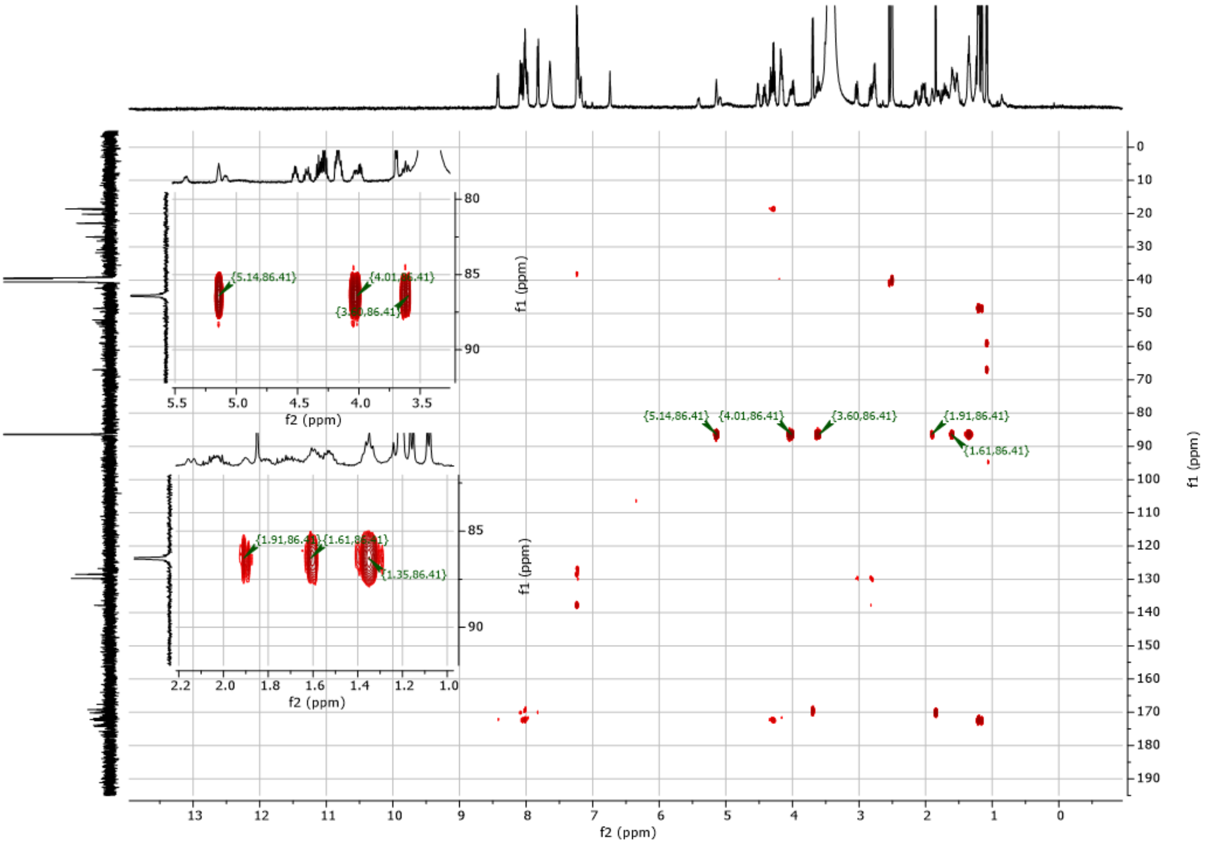

COSY (600 MHz, DMSO-d6)

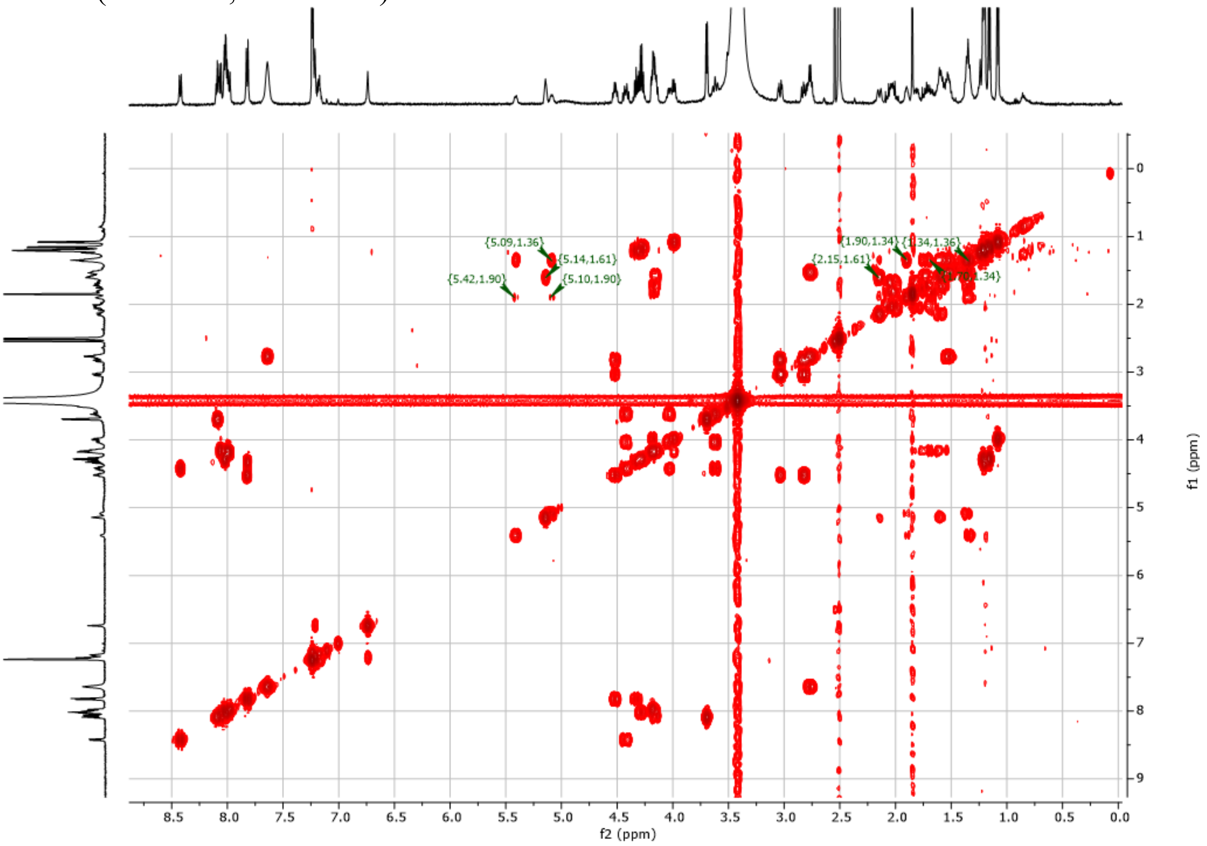

ROESY (600 MHz, DMSO-d6)

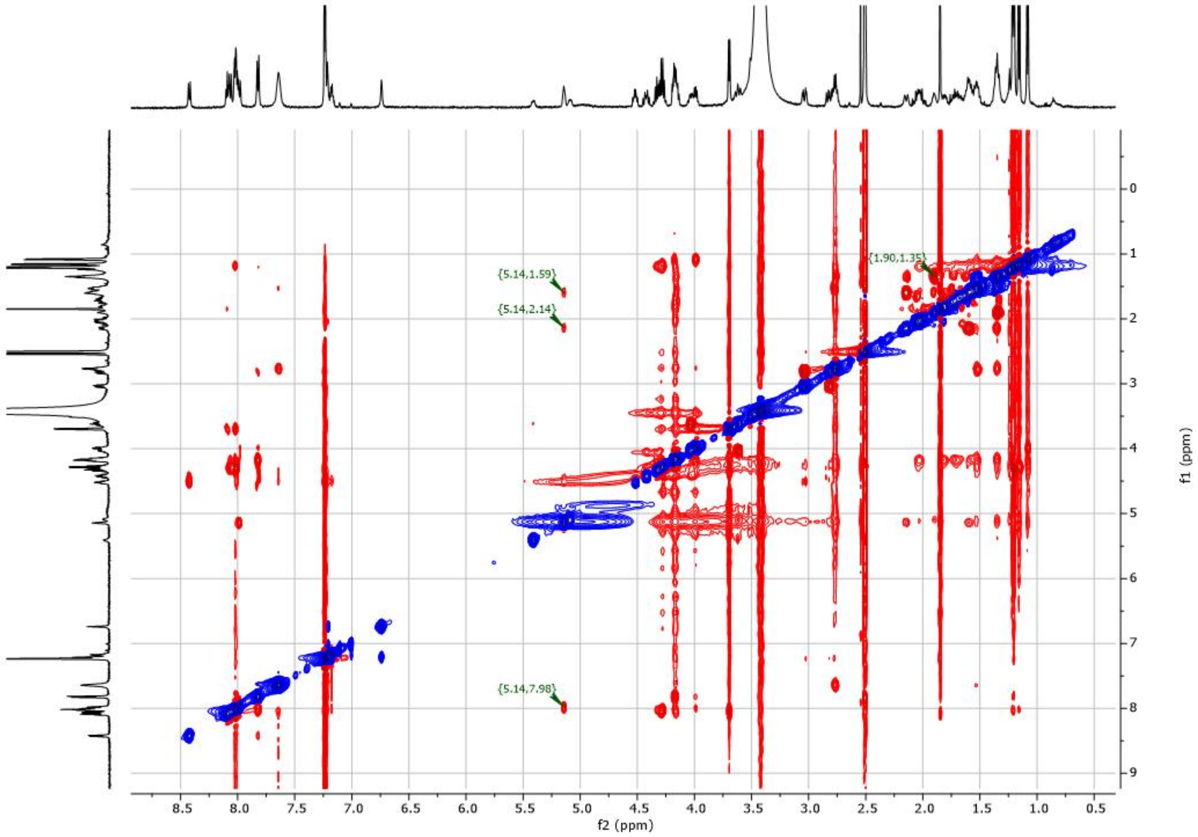

TOCSY (600 MHz, DMSO-d6)

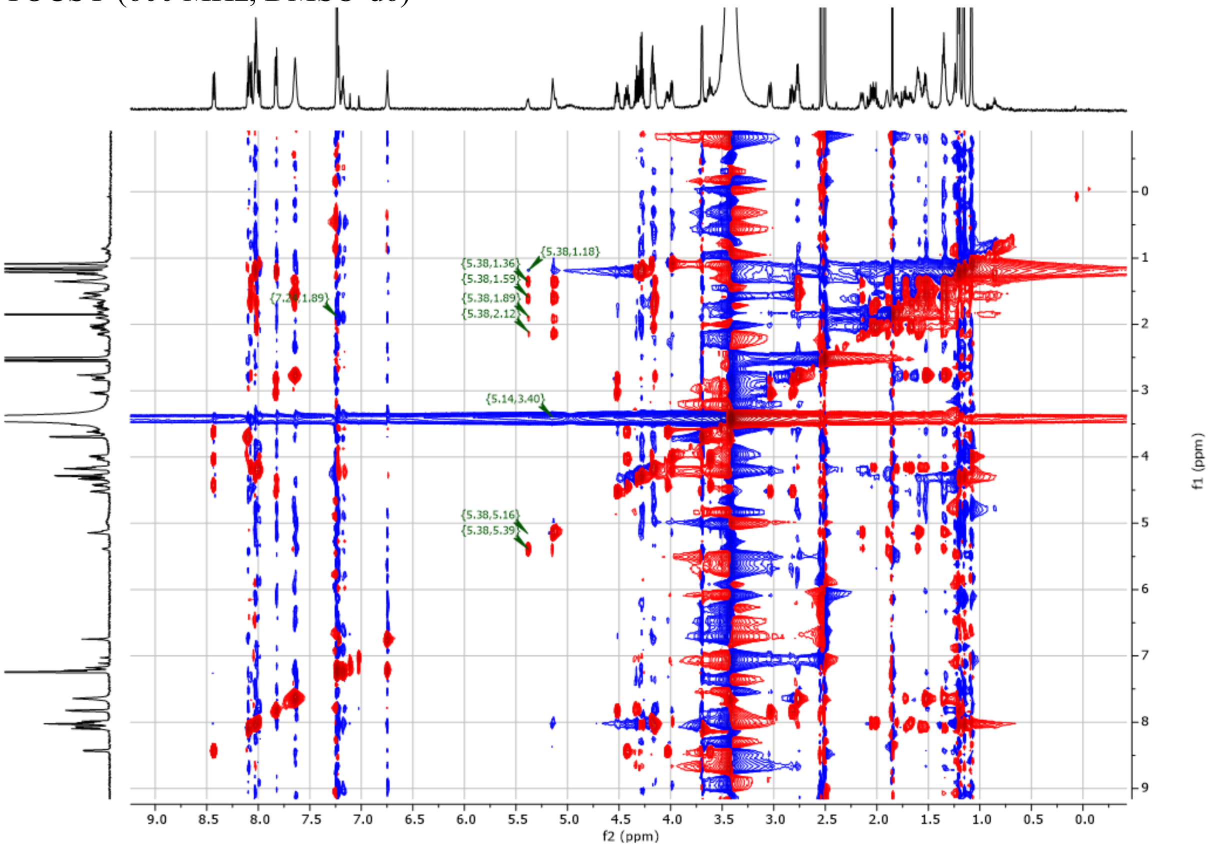

NHSQC (600 MHz, DMSO-d6)

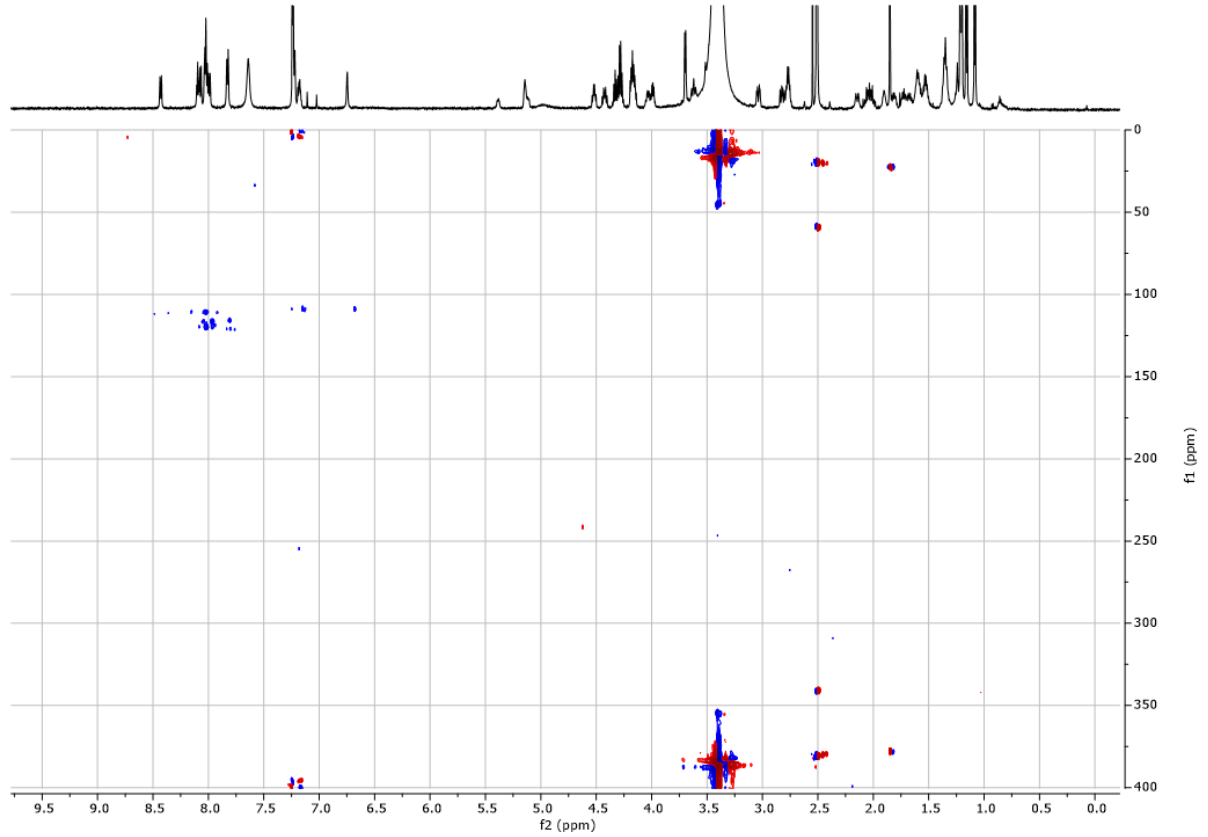

NHMBC (600 MHz, DMSO-d6)

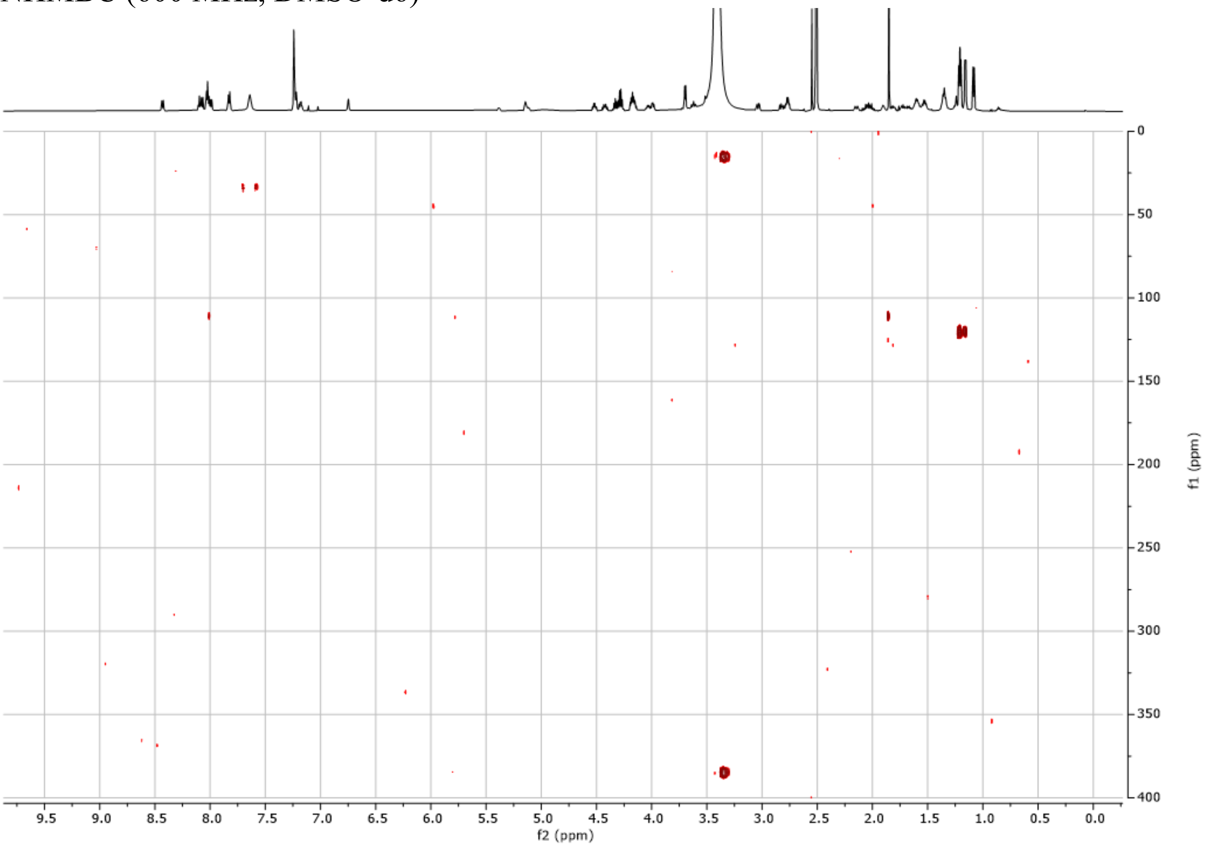

[^{13}C]15D, proposed structure (Cleavage iii)

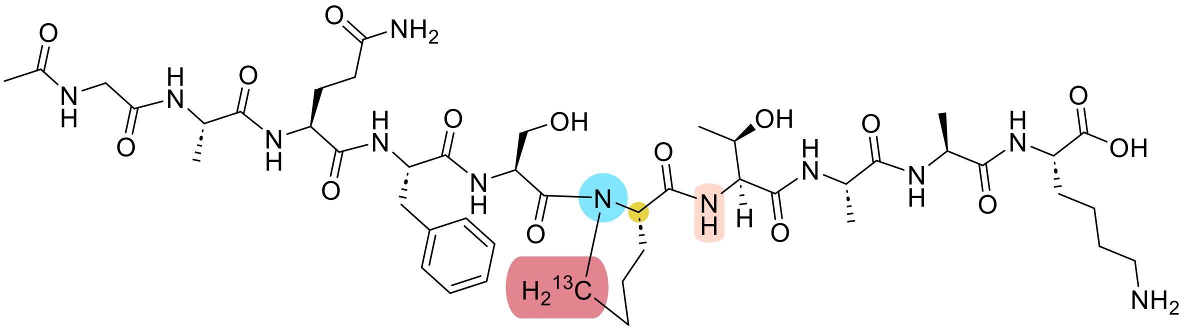

[^{13}C]15D

Chemical Formula: $C_{45}{}^{13}CH_{72}N_{12}O_{15}$

Exact Mass: 1033.5274

LC-MS of crude: ca. 20% UV-purity

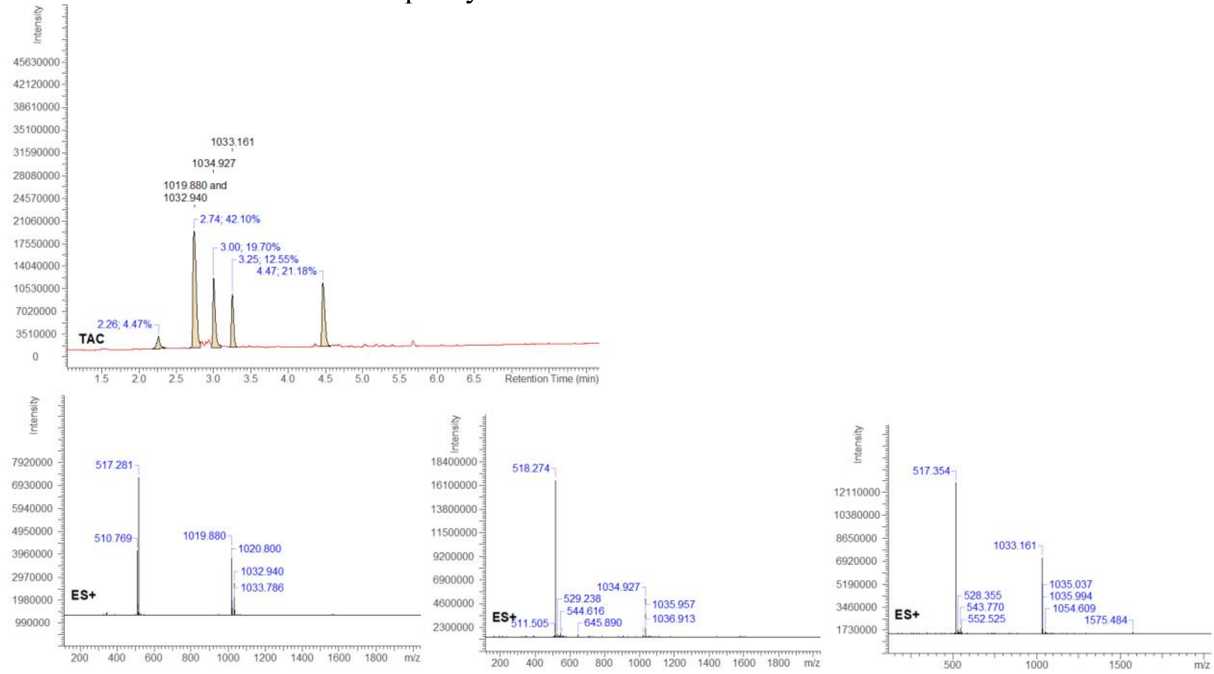

LC-MS of purified product: ca. 88% UV-purity

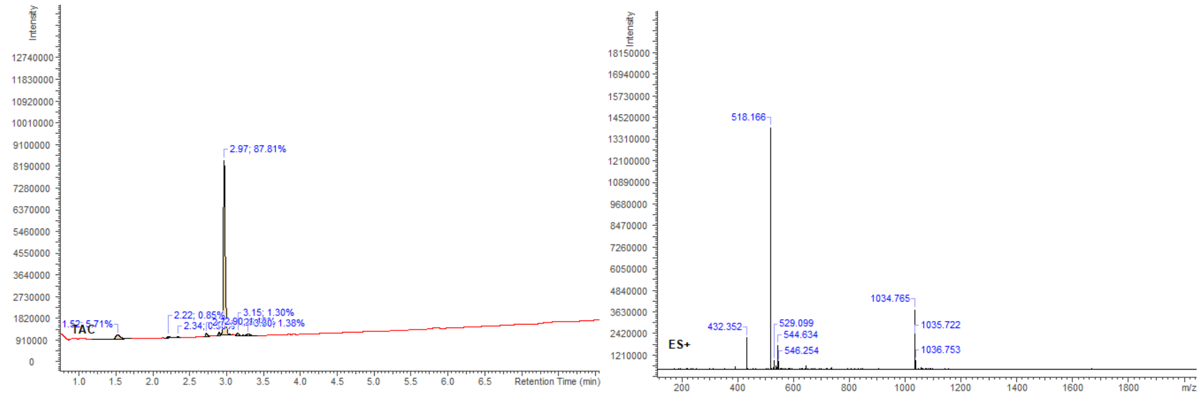

Isotope distribution comparison: Predicted non-labeled vs labeled peptide, ca. +0.99 ^{13}C/molecule incorporation

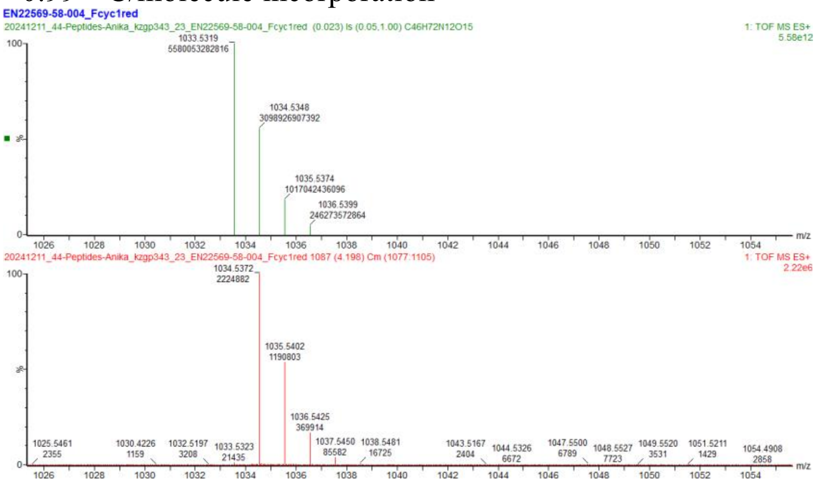

Results	
Relative amounts [%]	
unlabeled	1.0
1-label	101.0
2-label	-1.9
3-label	-0.5
4-label	0.1
5-label	0.9
6-label	-0.5

MSe analysis:

[13C]15D

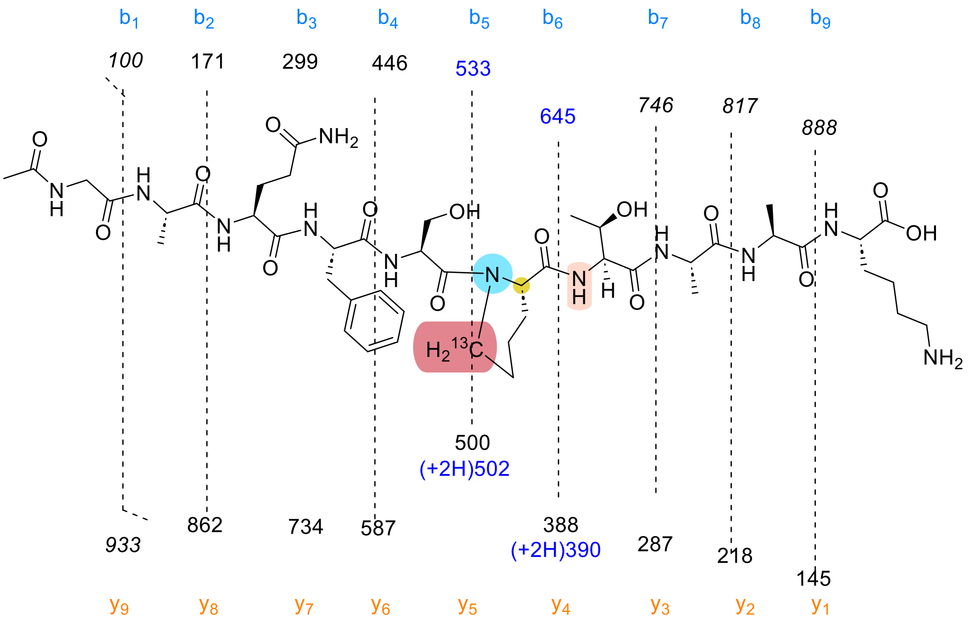

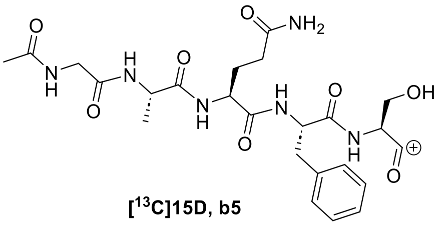

[^{13}C]15D, b5
Chemical Formula: $C_{24}H_{33}N_6O_8^+$
Exact Mass: 533.2354

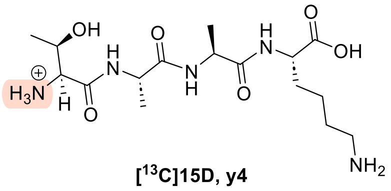

[^{13}C]15D, y4
Chemical Formula: $C_{16}H_{32}N_5O_6^+$
Exact Mass: 390.2347

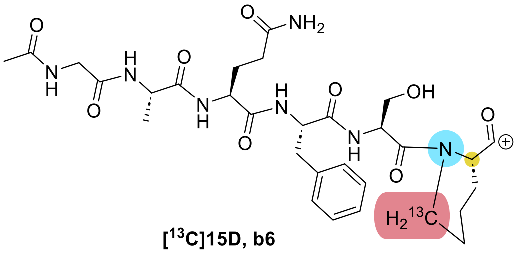

[^{13}C]15D, b6
Chemical Formula: $C_{29}{}^{13}CH_{42}N_7O_9^+$
Exact Mass: 645.3072

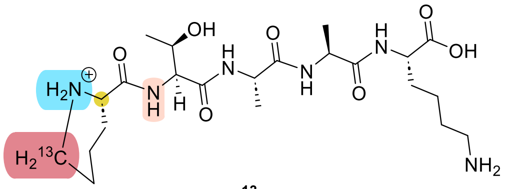

[^{13}C]15D, y5
Chemical Formula: $C_{21}{}^{13}CH_{41}N_6O_7^+$
Exact Mass: 502.3065

[^{13}C]15D, b5 calculated for $C_{24}H_{33}N_6O_8^+$ 533.2354, found 533.2296.
[^{13}C]15D, b6 calculated for $C_{29}{}^{13}CH_{42}N_7O_9^+$ 645.3072, found 645.3113.
[^{13}C]15D, y4 calculated for $C_{16}H_{32}N_5O_6^+$ 390.2347, found 390.2399.
[^{13}C]15D, y5 calculated for $C_{21}{}^{13}CH_{41}N_6O_7^+$ 502.3065, found 502.3102.
→the presence of fragment **b6**, in combination with **b5** and **y5** fragmentation indicates six-membered ring formation

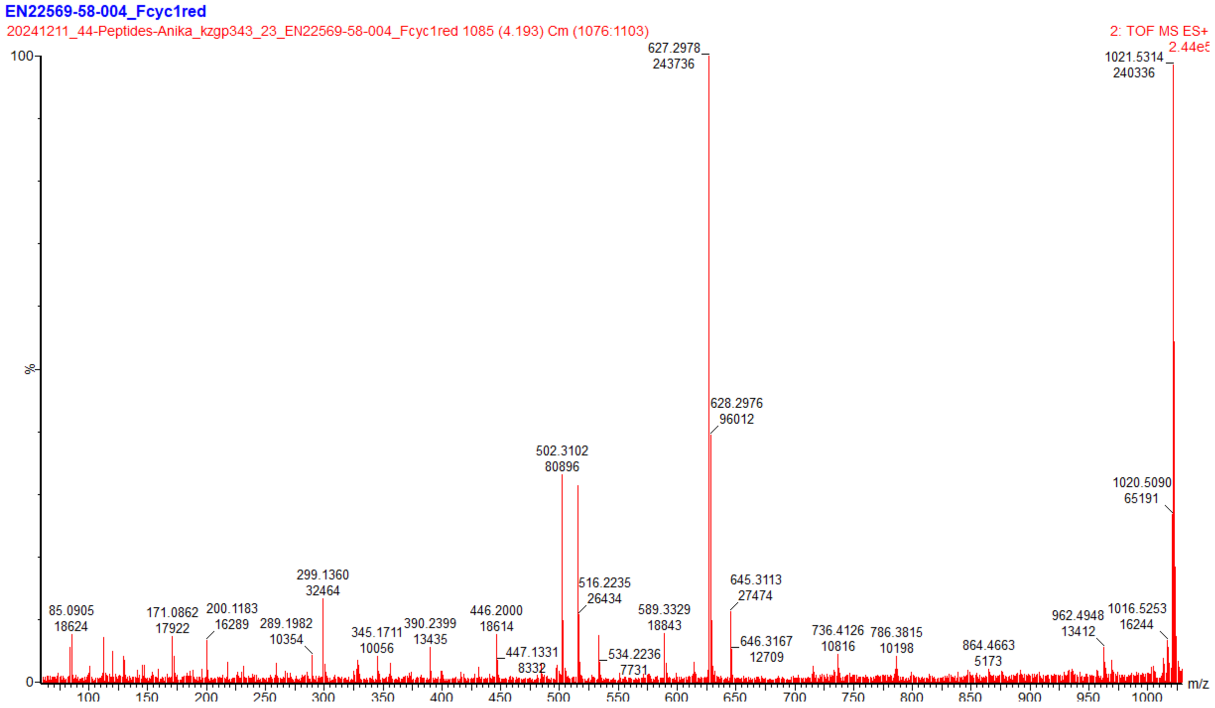

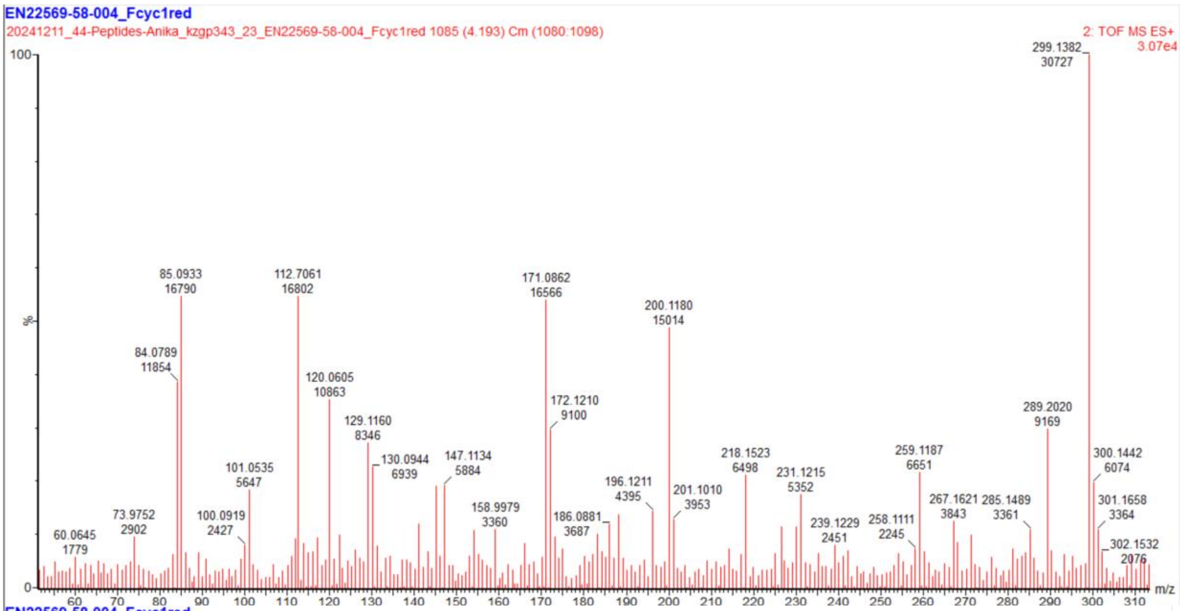
EN22569-58-004_Fcyc1red
20241211_44-Peptides-Anika_kzgp343_23_EN22569-58-004_Fcyc1red 1085 (4.193) Cm (1080:1098)
2: TOF MS ES+
3.07e4
299.1382 30727
85.0933 16790
112.7061 16802
171.0862 16566
200.1180 15014
84.0789 11854
120.0605 10863
172.1210 9100
129.1160 8346
130.0944 6939
147.1134 5884
289.2020 9169
300.1442 6074
101.0535 5647
218.1523 6498
259.1187 6651
231.1215 5352
196.1211 4395
201.1010 3953
267.1621 3843
285.1489 3361
301.1658 3364
73.9752 2902
100.0919 2427
60.0645 1779
158.9979 3360
186.0881 3687
239.1229 2451
258.1111 2245
302.1532 2076
m/z

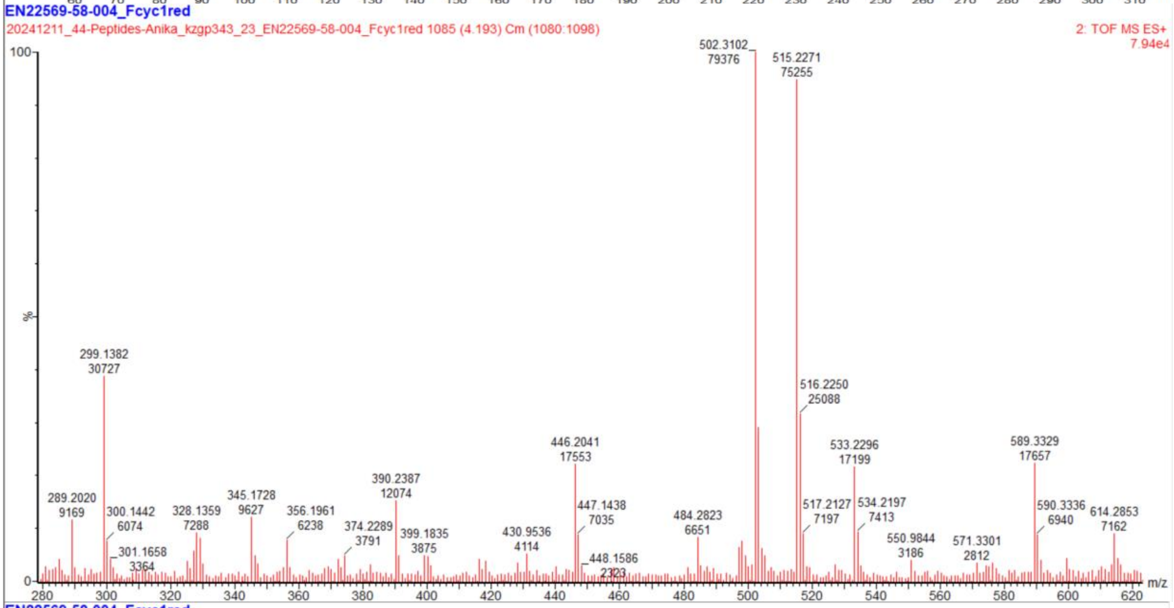
EN22569-58-004_Fcyc1red
20241211_44-Peptides-Anika_kzgp343_23_EN22569-58-004_Fcyc1red 1085 (4.193) Cm (1080:1098)
2: TOF MS ES+
7.94e4
502.3102 79376
515.2271 75255
299.1382 30727
516.2250 25088
446.2041 17553
533.2296 17199
589.3329 17657
390.2387 12074
289.2020 9169
300.1442 6074
328.1359 7288
345.1728 9627
356.1961 6238
374.2289 3791
399.1835 3875
430.9536 4114
447.1438 7035
448.1586 2323
484.2823 6651
517.2127 7197
534.2197 7413
550.9844 3186
571.3301 2812
590.3336 6940
614.2853 7162
301.1658 3364
m/z

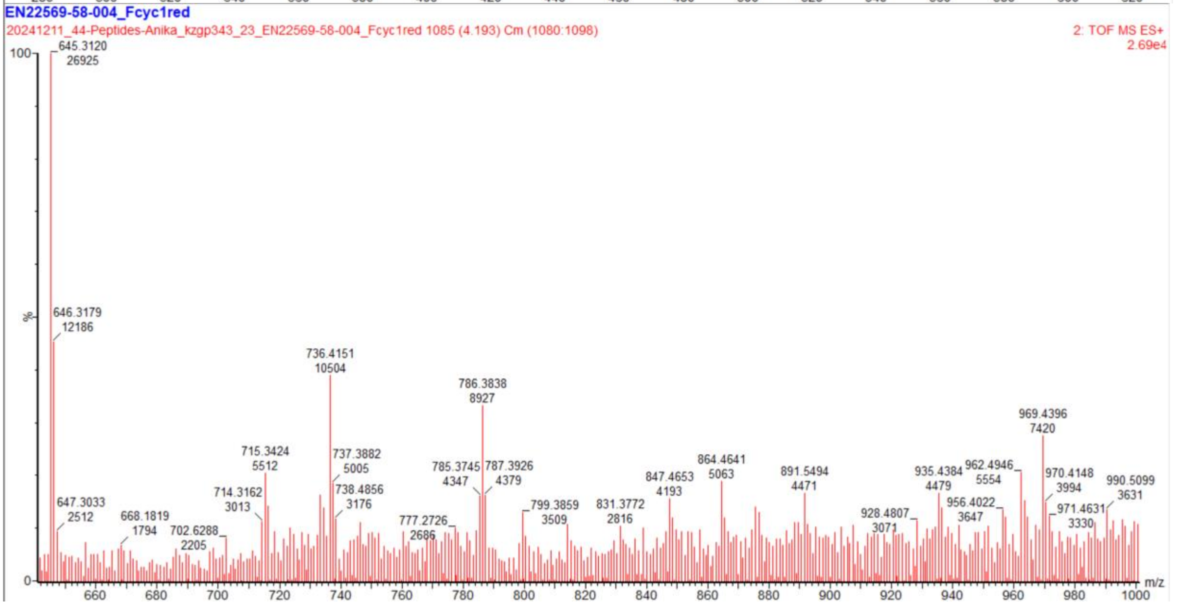
EN22569-58-004_Fcyc1red
20241211_44-Peptides-Anika_kzgp343_23_EN22569-58-004_Fcyc1red 1085 (4.193) Cm (1080:1098)
2: TOF MS ES+
2.69e4
645.3120 26925
646.3179 12186
736.4151 10504
786.3838 8927
969.4396 7420
715.3424 5512
737.3882 5005
738.4856 3176
785.3745 4347
787.3926 4379
714.3162 3013
647.3033 2512
668.1819 1794
702.6288 2205
777.2726 2686
799.3859 3509
831.3772 2816
847.4653 4193
864.4641 5063
891.5494 4471
928.4807 3071
935.4384 4479
962.4946 5554
956.4022 3647
970.4148 3994
971.4631 3330
990.5099 3631
m/z

NMR of purified product: ^{1}H-NMR, ^{13}C-NMR, HSQC, HMBC, COSY, ROESY, TOCSY, NHSQC, NHMBC

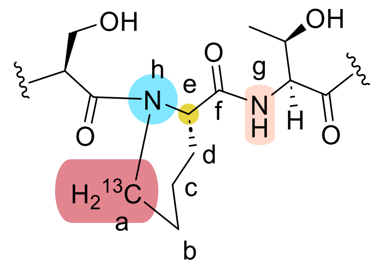

[^{13}C]15D

a: ^{13}C at 43.37 ppm, crosspeak in HSQC with ^{1}H 2 x at 3.95 ppm and 3.20 ppm (HSQC, maybe due to diastereomers, different conformations etc., not further investigated), split protons not visible in HMBC

^{1}H-NMR (600 MHz, DMSO-d6)

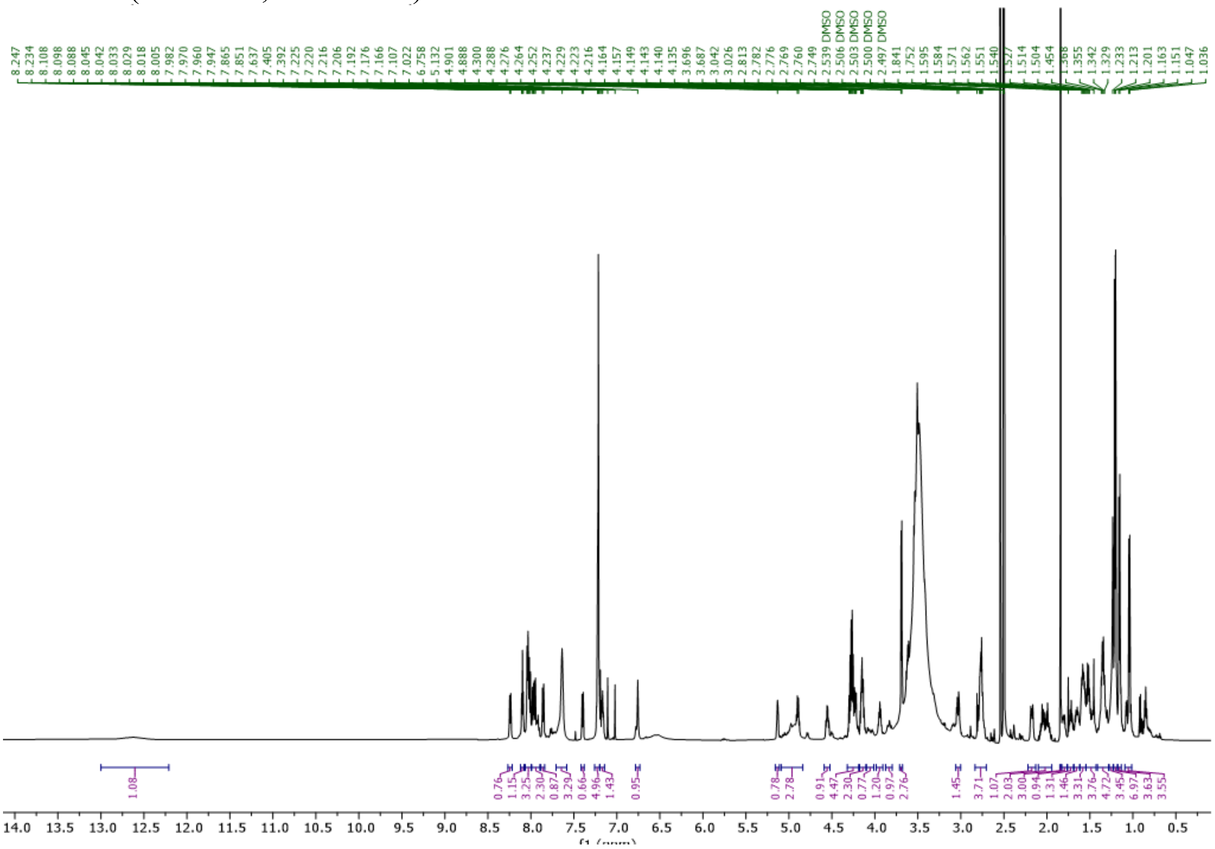

^{13}C-NMR (151 MHz, DMSO-d6)

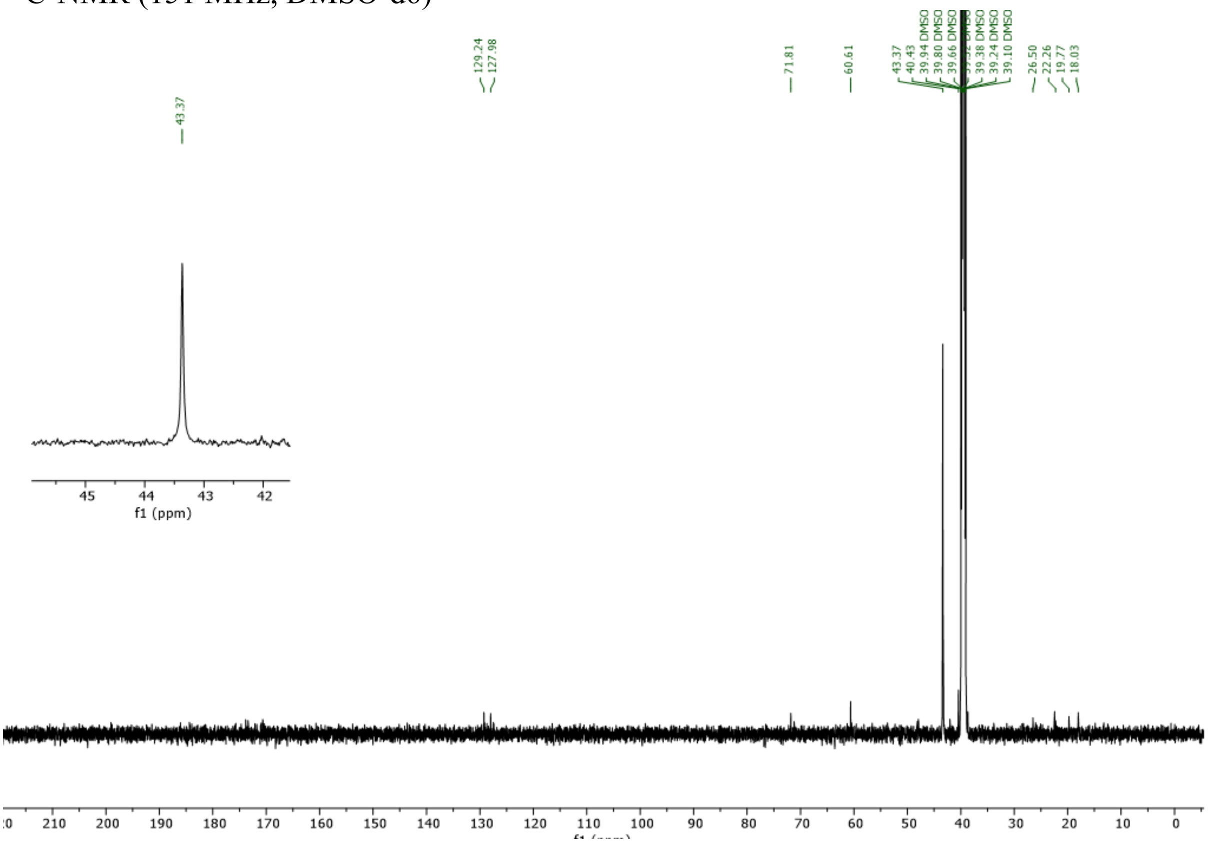

HSQC (600 MHz, DMSO-d6)

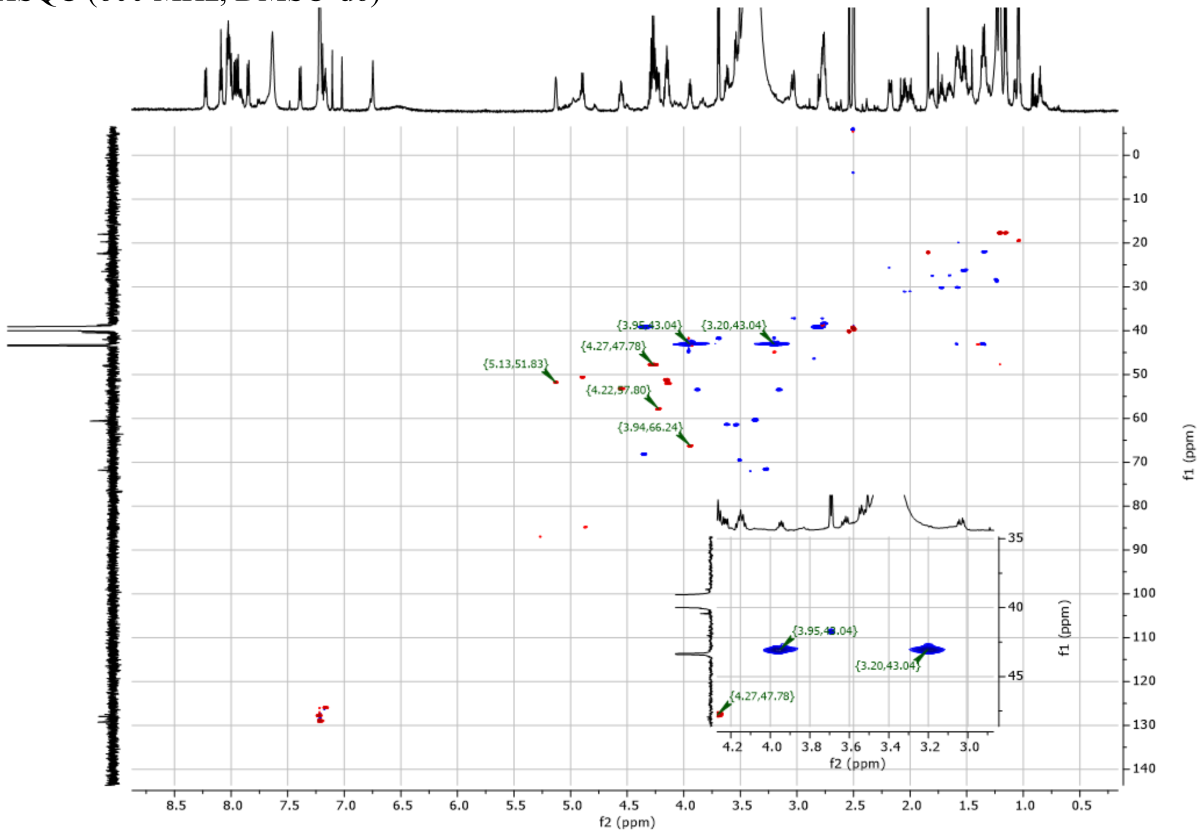

HMBC (600 MHz, DMSO-d6)

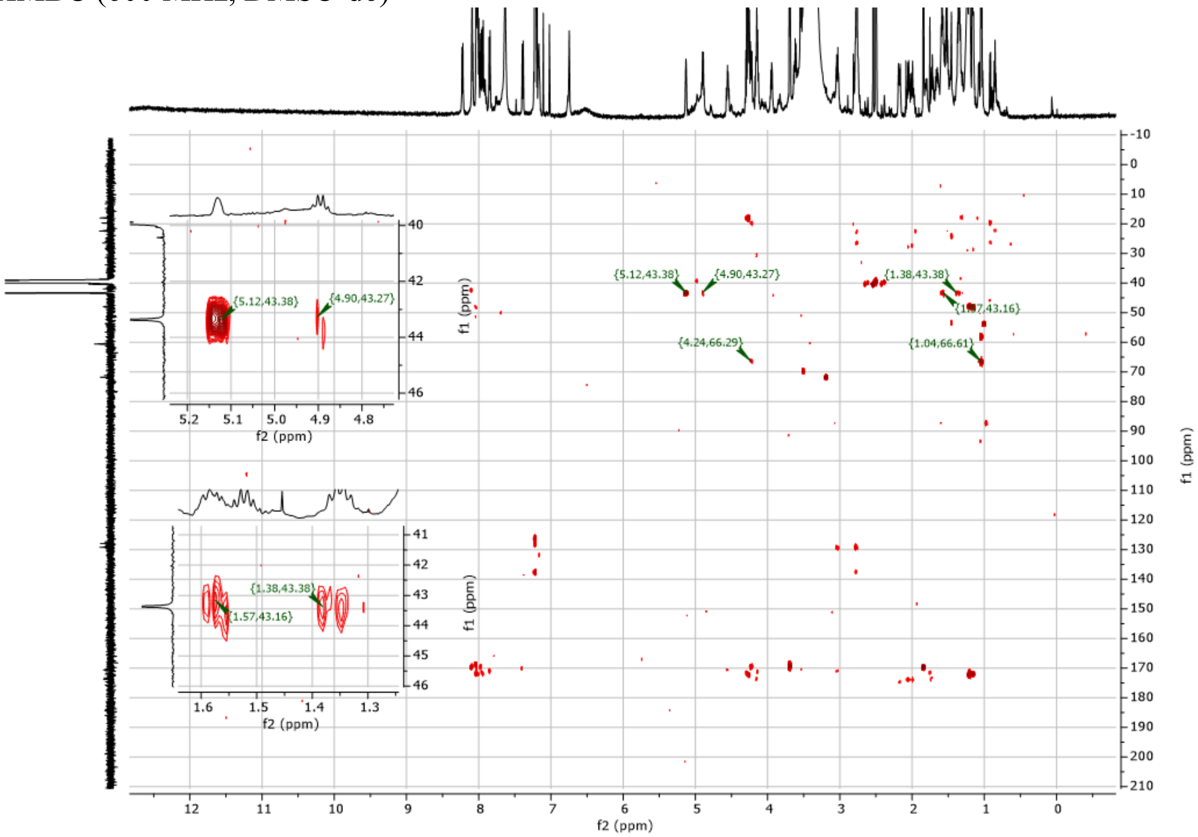

COSY (500 MHz, DMSO-d6)

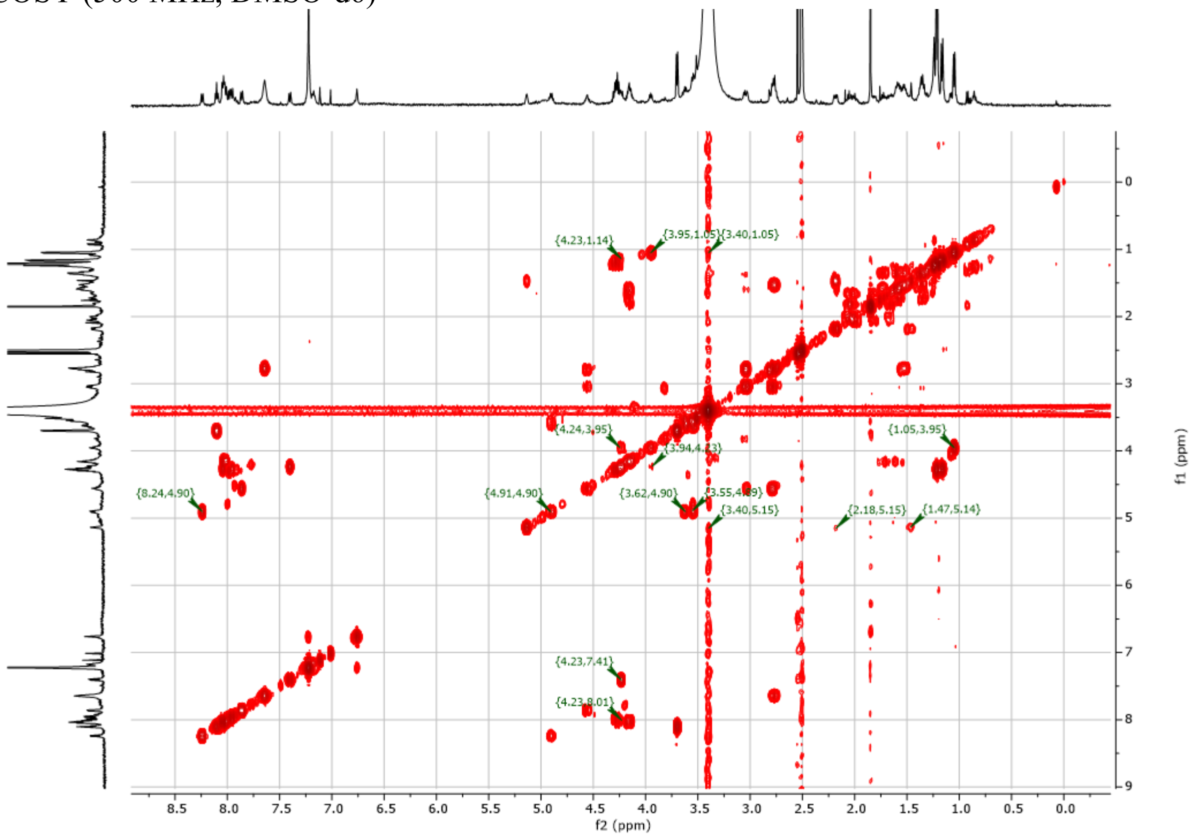

ROESY (500 MHz, DMSO-d6)

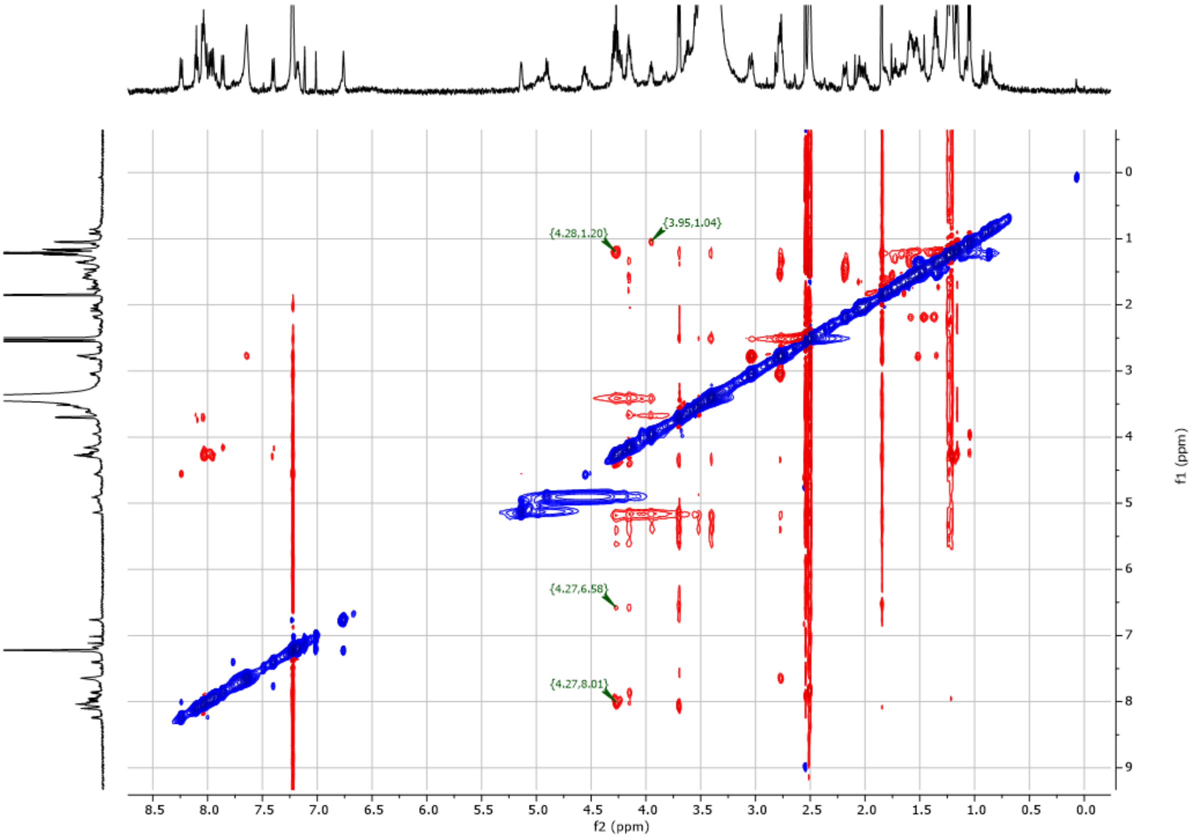

TOCSY (600 MHz, DMSO-d6)

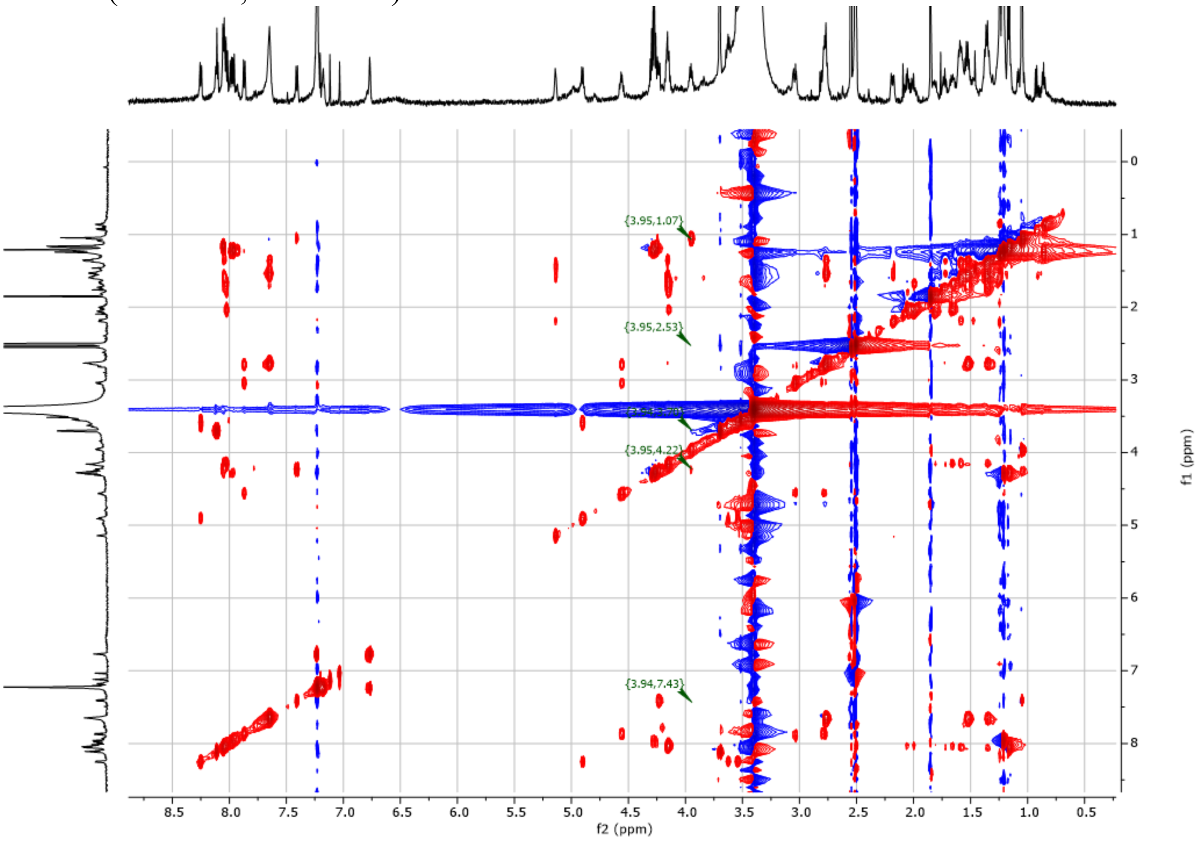

NHSQC (600 MHz, DMSO-d6)

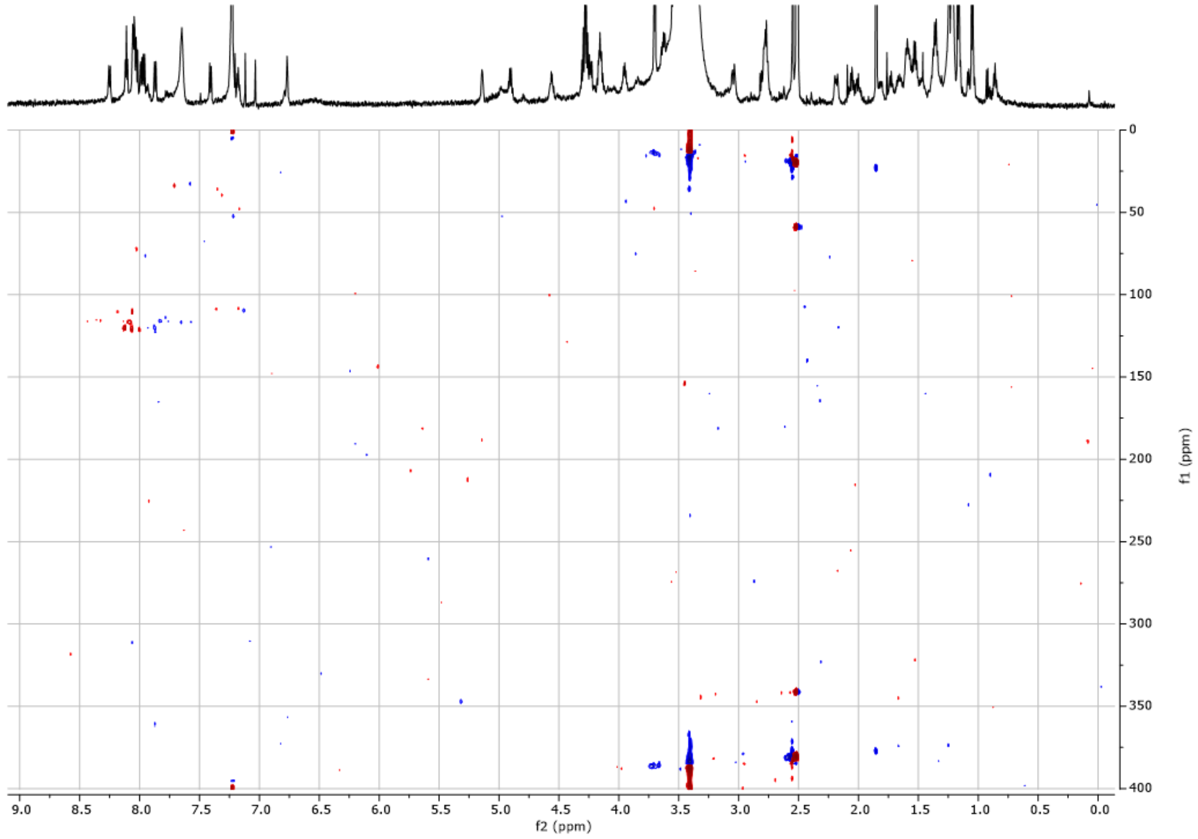

11. Supplementary references

1 Gruber, C. C. *et al.* An Algorithm for the Deconvolution of Mass Spectrosopic Patterns in Isotope Labeling Studies. Evaluation for the Hydrogen−Deuterium Exchange Reaction in Ketones. *J. Org. Chem.* **72**, 5778–5783 (2007).

2 Elmore, C. S., Schenk, D. J., Arent, R. & Kingston, L. Evaluation of UV–HPLC and mass spectrometry methods for specific activity determination. *J. Labelled Compd. Radiopharm.* **57**, 645–651 (2014).

3 Hermange, P. *et al.* Ex Situ Generation of Stoichiometric and Substoichiometric 12CO and 13CO and Its Efficient Incorporation in Palladium Catalyzed Aminocarbonylations. *J. Am. Chem. Soc.* **133**, 6061–6071 (2011).

4 Lindhardt, A. T. *et al.* 14Carbon monoxide made simple – novel approach to the generation, utilization, and scrubbing of 14carbon monoxide. *J. Labelled Compd. Radiopharm.* **55**, 411–418 (2012).

5 Yin, Q. *et al.* An Inhaled Inhibitor of Myristoylated Alanine-Rich C Kinase Substrate Reverses LPS-Induced Acute Lung Injury in Mice. *Am. J. Respir. Cell Mol. Biol.* **55**, 617–622 (2016).

6 Breit, B. & Seiche, W. Hydrogen Bonding as a Construction Element for Bidentate Donor Ligands in Homogeneous Catalysis: Regioselective Hydroformylation of Terminal Alkenes. *J. Am. Chem. Soc.* **125**, 6608–6609 (2003).

7 Zhang, B., Peña Fuentes, D. & Börner, A. Hydroformylation. *ChemTexts* **8**, 2 (2021).

8 Franke, R., Selent, D. & Börner, A. Applied hydroformylation. *Chem. Rev.* **112**, 5675–5732 (2012).

9 Sang, R. *et al.* A practical concept for catalytic carbonylations using carbon dioxide. *Nat. Commun.* **13**, 4432 (2022).

10 Friis, S. D., Taaning, R. H., Lindhardt, A. T. & Skrydstrup, T. Silacarboxylic Acids as Efficient Carbon Monoxide Releasing Molecules: Synthesis and Application in Palladium-Catalyzed Carbonylation Reactions. *J. Am. Chem. Soc.* **133**, 18114–18117 (2011).

11 Nicolás, E., Pujades, M., Bacardit, J., Giralt, E. & Albericio, F. A new approach to Hmb-backbone protection of peptides: Synthesis and reactivity of Nα-Fmoc-Nα-(Hmb)amino acids. *Tetrahedron Lett.* **38**, 2317–2320 (1997).
