# Supplementary material for: Late-stage generation of 14C/3H-radiolabeled lysine residues via hydroformylation of peptides
Source: Nat Commun. 2026 Jun 12;17:5307. doi: 10.1038/s41467-026-74115-8 (PMC13272800; doi:10.1038/s41467-026-74115-8)
Supplement: Supplementary file 2 — Transparent Peer Review file [file 41467_2026_74115_MOESM2_ESM.pdf]

nature portfolio

Peer Review File

Late-Stage Generation of 14C/3H-Radiolabeled Lysine Residues via Hydroformylation of Peptides

Corresponding Author: Ms Anika Schick

This file contains all reviewer reports in order by version, followed by all author rebuttals in order by version.

Version 0:

Reviewer comments:

Reviewer #1

(Remarks to the Author)
The manuscript by Elmore and co-workers reports on the development of a novel, on-resin strategy for the installation of 14C or 3H-radiolabeled lysine residues directly onto a synthetic peptide backbone via sequential hydroformylation/reductive amination of an allylglycine moiety. The setup enables flexible isotope introduction through use of 14CO from solid precursors and 3H2 from standard tritium manifolds. As proof of concept, the methodology was applied to the preparation of radiolabelled analogues of a number of therapeutically relevant peptide, including, somatostatin and terlipressin. Overall the manuscript is very well written and clearly presented and the methodology does appear to offer facile access to radiolabeled peptides suitable for biological study. The manuscript is suitable for publication in Nat. Commun. subject to minor corrections.

Comments:
1. Is use of DMSO as a solvent/co-solvent a limitation of the methodology, challenging to remove and often unsuitable for biological applications? Please comment.
2. Could the authors comment on how use of 5% water in DMSO solvent system suppresses backbone cyclization, relative to neat DMSO?
3. The authors outline the numerous advantages of radiolabeled peptides which are well established, however, it would be useful demonstrate an application of one of the radiolabeled substates prepared via the reported methodology to clearly demonstrate the method gives access to substrates suitable for biological evaluation/Med Chem applications.

Reviewer #2

(Remarks to the Author)
This manuscript reports procedures for the Rh-catalyzed hydroformylation of allylglycine using isotopically labeled CO and H_2. The motivation for the study is to develop improved methods for the late-stage incorporation of isotope tracers into peptides as part of drug development campaigns. Developing a successful and reasonably general reaction is highly challenging because (1) relatively low loadings of labeled CO and H_2 must be used; and (2) the complex nature of peptide structures may inhibit the reaction, poison the catalyst, or lead to unexpected, sequence-dependent results. Schick and co-workers address these challenges in this paper. While I have some suggestions for the authors to consider, I believe the work is of high quality and suitable for publication in this journal.

Specific Comments

1. The reaction uses a large excess of RhCODCl rather than $Rh(CO)_2acac$ to avoid CO dilution. After catalyst activation, substantial amounts of COD will be present; the authors should test whether COD hydroformylation products are detected, potentially consuming valuable labeled syngas. A possible workaround would be to use Rh(ethylene)Cl as the precatalyst.

2. The SI clearly states that branched and linear isomers are generated in the hydroformylation and explains the otherwise unusual HPLC assignments. This information should be included directly in the main manuscript.

3. In my opinion, the manuscript uses excessive and non-standard abbreviations. Some are not defined (e.g., TiPS, DODT),

and some appear to be used inconsistently (e.g., CD vs DC). In many cases, writing out the full terms in the text or figures would improve readability.

4. The first paragraph on page 11 appears to describe a key finding that water improves reactivity, but no specific data are shown, and it is not clear on which substrate this was confirmed. The relevant data appear to be provided only in the SI. If this is an important conclusion, representative data should be included in the main text.

5. Reactions are conducted at low concentrations (~5–10 µM), often in 1–2 mL of solvent. I did not see this parameter varied in the SI. Increasing the concentration might improve reaction rates and lead to full substrate conversion with fewer side reactions. Is this limited by the use of specialized glassware or other experimental constraints?

6. The results shown in Figure 6 and the corresponding discussion could potentially be removed from the paper. The Wittig reaction does not appear optimized, is not particularly surprising, and seems to have limited immediate relevance to the isotope-labeling work.

7. It is not clear from the data or discussion whether the extent of $^{3}H/^{14}C$ labeling of the targets is sufficient for the applications described in the introduction. If this is sufficient, that should be explicitly stated, if not this limitation should be acknowledged.

Reviewer #3

(Remarks to the Author)
Late-Stage 14C/3H- Radiolabeling of Lysine Residues via 1 Hydroformylation of Peptides

Schick et al.

The authors present a neat method for the radiolabelling of peptides, involving an on-resin hydroformylation-reductive amination late-stage modification cascade. Peptides containing allyl- or homoallylglycine residues were synthesised using solid-phase peptide synthesis and then subjected to 1) hydroformylation with syngas in a two-chamber setup, followed by 2) reductive amination of the resulting allysine. The reaction conditions were carefully optimised and demonstrated to be applicable to a variety of peptides containing all relevant functional groups. These conditions were then used for radiolabelling, albeit with slight adaptations due to the use of 3H2 gas instead of an H2 gas surrogate. Radiolabelling of complex peptides such as 13-mer STAT3-Hel2A-2 and 31-mer semaglutide has also been demonstrated. In the final chapter, the authors discuss extending their reaction cascade to include a hydroformylation-reductive amination-Wittig reaction cascade for the late-stage diversification of resin-bound peptides.
I am somewhat conflicted about this study. I really like the first part, the hydroformylation-reductive amination cascade and its use for the radiolabeling of peptides. The conditions were carefully optimized and described in detail in the extensive supplementary material. It is really important that the authors provided such detailed information on the reaction conditions and setups. This will certainly enable others to successfully apply this method to their peptides. Only minor points need to be addressed in this part. However, the last section on late-stage diversification is very weak! My advice to the authors: invest more time and present a more mature study on the hydroformylation-reductive amination-Wittig reaction cascade, or omit this part entirely. With this study included, I cannot recommend the manuscript for publication in Nature Communications without really extensive revisions. However, if the authors decide to omit this part, I would be happy to recommend publication in Nature Communications, as the first part is of great importance.
Please see below for the points that require improvement prior to publication:
1) The phrase 'late-stage radiolabelling of lysine', as used in the title, is misleading, since it is technically the allylglycine residues that are modified. Perhaps the authors should revise the title to 'Late-Stage 14C/3H Radiolabelling of Resin-Bound Peptides via a Hydroformylation–Reductive Amination Cascade on Allylglycine Residues'.
2) The authors annotate the introduced C1 building block with 12/13C in large parts of the manuscript. At first, it seemed to me that this was a mixture of both isotopes. However, if I understand correctly, it means 12C and 13C, since both are produced using the same method. This nomenclature should perhaps be clarified in one place or formulated more clearly in general.
3) The authors had to modify the conditions considerably for semaglutide to make the reaction work. Are these modified conditions generally superior to other conditions, or are they only optimal for semaglutide? Could these reaction conditions also be used for the other test peptides, for which the yield and selectivity were low?
4) On p. 19, the authors mention that Raj et al. presented allysine as a handle for reductive amination. That is correct, but Luo et al., Chem. Commun. 2012, 48, 1514-1516, did so before them, to my knowledge. Admittedly, on a smaller scope, but also using allysine. However, they achieved this through the oxidation of hydroxynorleucine with Dess-Martin periodinate. Nonetheless, this should be mentioned and cited.
5) These were the minor points of criticism regarding the first part. I find the second part, or rather the last results chapter, to be very premature. The late-stage diversification using a hydroformylation-reductive amination-Wittig reaction cascade was only demonstrated for a very small scope of peptides and, at best, with a moderate yield. This stands in stark contrast to the excellently executed work presented in the first part of the manuscript. No optimization of reaction conditions, no HPLC traces in the manuscript, etc. In its current state, this is not publishable! Either the authors invest more time and present a mature study here, or they leave this part out entirely and publish it separately. Honestly, I consider the manuscript without this part to be publishable and of higher quality.

Version 1:

Reviewer comments:

Reviewer #1

(Remarks to the Author)
All points have been fully addressed in the revised manuscript, the manuscript is now suitable for publication in Nat. Commun.

Reviewer #2

(Remarks to the Author)
The revised manuscript and comments in the response letter help to clarify/resolve the issues brought up in the first round of review. I support publication of this high quality work.

Reviewer #3

(Remarks to the Author)
The authors have addressed all my concerns to my full satisfaction. The only comment is regarding the first sentence of the Conclusion. Without the on-resin Wittig reaction, the authors only address 'critical bottlenecks in radiolabelling', not 'site-selective diversification'. I would recommend removing 'site-selective diversification' from the first sentence of the conclusion.

Open Access This Peer Review File is licensed under a Creative Commons Attribution 4.0 International License, which permits use, sharing, adaptation, distribution and reproduction in any medium or format, as long as you give appropriate credit to the original author(s) and the source, provide a link to the Creative Commons license, and indicate if changes were made.
In cases where reviewers are anonymous, credit should be given to 'Anonymous Referee' and the source.
The images or other third party material in this Peer Review File are included in the article's Creative Commons license, unless indicated otherwise in a credit line to the material. If material is not included in the article's Creative Commons license and your intended use is not permitted by statutory regulation or exceeds the permitted use, you will need to obtain permission directly from the copyright holder.
To view a copy of this license, visit https://creativecommons.org/licenses/by/4.0/

Reviewer comments and answers of authors

Reviewer #1 (Remarks to the Author):

The manuscript by Elmore and co-workers reports on the development of a novel, on-resin strategy for the installation of 14C or 3H-radiolabeled lysine residues directly onto a synthetic peptide backbone via sequential hydroformylation/reductive amination of an allylglycine moiety. The setup enables flexible isotope introduction through use of 14CO from solid precursors and 3H2 from standard tritium manifolds. As proof of concept, the methodology was applied to the preparation of radiolabelled analogues of a number of therapeutically relevant peptide, including, somatostatin and terlipressin. Overall the manuscript is very well written and clearly presented and the methodology does appear to offer facile access to radiolabeled peptides suitable for biological study. The manuscript is suitable for publication in Nat. Commun. subject to minor corrections.

Comments:

1. Is use of DMSO as a solvent/co-solvent a limitation of the methodology, challenging to remove and often unsuitable for biological applications? Please comment.

Answer: Since the hydroformylation reaction is carried out on solid support, the solvents (including DMSO) and all excess reagents are removed by simple filtration and resin washing. DMSO is a frequently employed solvent in peptide chemistry, especially for peptides prone to aggregation. For large biologics (like antibodies), DMSO as a (main) solvent would be unsuitable as it may denature them. However, those are beyond the intended scope of our method which covers peptides (up to 31 amino acids) on solid support. Maintaining the tertiary structure is not a constraint for the on-resin reactions. For downstream use (biological applications such as dosing the radiolabeled peptide to an organism), the product peptide is purified by HPLC (which removes any possible DMSO) and would be formulated in the according formulation buffer required for the studies. Therefore, DMSO is not carried into *in vitro* or *in vivo* dosing formulations.

2. Could the authors comment on how use of 5% water in DMSO solvent system suppresses backbone cyclization, relative to neat DMSO?

Answer: This is an interesting question that we also wondered about. The mechanism remains however unproven.

For our reaction system, we found that the tendency for cyclization depends on structural features, reaction time and temperature, as well as on the ligands of the Rh-catalyst in neat DMSO as solvent. Addition of water mitigated formation of cyclization products (Supporting Information, Chapter 2.3). Plausible explanations could be that addition of water may lead to formation of a less reactive hydrate compared to the aldehyde, or that the amide backbone would become less nucleophilic under aqueous conditions. While acid-catalyzed cyclization of allysine acetals has precedent (reference: Botman et al. *Org. Lett.* **2004**, *6* (26), 4941–4944.), and solvent effects on allysine hydration are likely, we did not perform additional experiments to back up the possible explanations and therefore did not detail this in the manuscript.

3. The authors outline the numerous advantages of radiolabeled peptides which are well established, however, it would be useful demonstrate an application of one of the radiolabeled

substates prepared via the reported methodology to clearly demonstrate the method gives access to substrates suitable for biological evaluation/Med Chem applications.

Answer: A modified version of the reported method was used by AstraZeneca to synthesize a ^{14}C-labeled small peptide (2-mer) in 2024–2025 and this compound was used in a QWBA study. However, that compound is proprietary and cannot be disclosed.

We specifically designed this current manuscript to avoid proprietary AZ compounds so that it would be able to be published in a timely manner. However, the use of peptides containing ^{14}C-labeled amino acids is well precedented in DMPK studies especially for human ADME studies with Accelerator Mass Spec (AMS) detection (reference 1 with ORG 37462: Wiegerinck et al. *J. Labelled Compd. Radiopharm.* **51**, 195–201 (2008); reference 2 with pasireotide: Lin et al. *Cancer Chemother. Pharmacol.* **72**, 181-188 (2013); reference 3 with etelcalcetide: Subramanian et al. *Clin Pharmacokinet* **56**, 179–192 (2017)). The example of [^{14}C]pasireotide is especially relevant since a natural amino acid (Phe) was used in labeled form. A white paper has been published covering the metabolism and excretion of therapeutic peptides (reference: He et al. *Drug Metab. Dispos.* **51**, 1436–1450 (2023)). This paper reports that 14 of 31 drugs approved by the EMEA and FDA were labeled with ^{14}C and 3 of 31 were labeled with ^{3}H. The white paper does highlight that care must be taken when labeled amino acids which can be incorporated into peptide synthesis are used; the ^{14}C-labeled peptide could be metabolized to give the constituent ^{14}C-labeled amino acid and that ^{14}C-labeled peptide ([^{14}C]lysine in this work) could enter peptide synthesis. This could give a high prolonged radioactive dose to a subject. Thus, this risk needs to be properly assessed prior to dosing a human; however, this risk assessment will not need to occur for use *in vitro* studies, non-human *in vivo* studies, or for human studies with AMS detection. Studies using AMS detection use a very low amount of ^{14}C-label and thus no dosimetry study is required and the radioactive burden to the subject is considered to be zero.

AstraZeneca ran a QWBA in 2025 on a large therapeutic peptide labeled with tritium at a similar molar activity albeit not using this methodology (the project was initiated prior to the development of this methodology). The ability to rapidly label a peptide for binding studies or localization studies is a great asset in the discovery phase.

Reviewer #2 (Remarks to the Author):

This manuscript reports procedures for the Rh-catalyzed hydroformylation of allylglycine using isotopically labeled CO and H_2. The motivation for the study is to develop improved methods for the late-stage incorporation of isotope tracers into peptides as part of drug development campaigns. Developing a successful and reasonably general reaction is highly challenging because (1) relatively low loadings of labeled CO and H_2 must be used; and (2) the complex nature of peptide structures may inhibit the reaction, poison the catalyst, or lead to unexpected, sequence-dependent results. Schick and co-workers address these challenges in this paper. While I have some suggestions for the authors to consider, I believe the work is of high quality and suitable for publication in this journal.

Specific Comments

1. The reaction uses a large excess of RhCODCl rather than Rh(CO)$_2$acac to avoid CO dilution. After catalyst activation, substantial amounts of COD will be present; the authors should test whether COD hydroformylation products are detected, potentially consuming valuable labeled syngas. A possible workaround would be to use Rh(ethylene)Cl as the precatalyst.

Answer: As Reviewer 2 highlights, the optimized conditions for the hydroformylation reactions (Manuscript, Figure 4) use a large amount of rhodium-“catalyst”, i.e. 0.72 equiv. of the pre-catalyst

$Rh(COD)_2BF_4$, which would correlate to effective 1.44 equiv. of cyclooctadiene or 2.88 equiv. of COD-derived double-bond functional groups relative to the terminal alkene of the allyl glycine residue. Therefore, the question of Reviewer 2 is highly relevant. We agree that 1,5-COD could, in principle, consume labeled syngas.

In general, terminal olefins are considered to be more reactive compared to internal olefins (reference: Franke et al. *Chem. Rev.* **112**, 5675–5732 (2012)). Based on this general reactivity we assumed that the terminal allylglycine residue should react faster than the internal olefin of COD. However, there are a few reported hydroformylations of 1,5-cyclooctadiene using harsher conditions but low loadings of catalyst, e.g. 0.6 mol% Rh-catalyst at 80 °C and 10 bar, yielding mainly cyclooctanecarboxyaldehyde, cyclooct-4-enecarboxyaldehyde, as well as isomerized 1,3-cyclooctadiene (reference: Trzeciak et al. *Organomet. Chem.* **479**, 213–216 (1994)). In another report, 1,5-cyclooctadiene undergoes reductive hydroformylation using Co-catalysis at 170 °C and 40 bar, to form the respective cyclooctyl-methanol (reference: Zhang et al. *Catalysis Science & Technology* **15**, 2295–2302 (2025)). Based on these reports, we agree with Reviewer 2 that the formation of 1,5-COD hydroformylation products needed to be investigated in more detail. The following three experiments address this (with the detailed descriptions shown in the upcoming pages):

- Experiment 1: uses the standard hydroformylation conditions (manuscript, Figure 3a) on hexapeptide **2SM**, but using ^{13}CO and deuterated solvents DMSO-d_6:D_2O (95:5). ^{13}C-NMR of the wash solution showed no ^{13}C-aldehyde signals near 200 ppm and therefore no evidence for COD-derived hydroformylation products. Of the initial 36 µmol 1,5-COD only ca. 14 µmol were recovered by qNMR, likely due to the loss of COD into the gas phase. The additional presence of non-identified COD-derived products can also not be excluded.
- Experiment 2: uses the standard hydroformylation conditions but only 1,5-COD as starting material in larger amounts (286 µmol/2 mL) and using 0.1 mmol syngas (H_2, ^{13}CO) (in non-deuterated solvents). Minor ^{13}C-aldehydes were detected by qNMR (^{1}H-NMR) and ^{13}C-NMR (total ca. 16 µmol). This indicates that COD can be hydroformylated, especially without the competitive allylglycine residue as substrate present and with the employed higher concentration of 1,5-COD. We also detected formation of substantial isomerization to 1,3-COD, which may contribute to isotope dilution for hydrogen isotope labeling experiments. The latter was however not examined.
- Experiment 3: uses the more forcing conditions with the ligand BIPHEPHOS at 60 °C, and using 0.2 mmol syngas (H_2, ^{13}CO) on the semaglutide analog as substrate. The reaction was performed on a small 2.7 µmol reaction scale, previously used for optimization (Supporting Information, Table S11). No ^{13}C-aldehydes were detected by NMR. Further, GC-MS analyses did not reveal formation of aldehyde derivatives, cyclooctane, or COD (-isomers). Admittedly, the latter could have been lost in the gas phase and thereby not detected, and the presence of non-identified COD-derived products can't be excluded.
- Experiment 4: uses more forcing hydroformylation conditions at 60 °C and 0.2 mmol syngas (^{12}CO,H_2), without any substrate other than the 1,5-COD already present in the Rh-precatalyst (ca. 36 µmol 1,5-COD). In two parallel experiments, the ligands 6-DPPon and BIPHEPHOS were compared. The reaction using 6-DPPon revealed formation of ca. 2 and 4 µmol aldehyde species, 4–5 µmol 1,5-COD recovery, and 4–5 µmol 1,3-COD formation. When using the ligand BIPHEPHOS, major formation of isomerization product 1,3-COD was found (14 µmol). This experiment reveals that the ligands lead to major conversion of 1,5-COD, and respectively to different COD-hydroformylation product compositions.
- Finally, increasing Rh-catalyst loading (and thus COD ligand) improved peptide conversion (manuscript, Figure 3a), which argues against considerable COD competition under the conditions employed.

Using di-µ-chlorotetraethylene dirhodium(I) is an interesting suggestion of Reviewer 2. This could especially be relevant if ethylene as a gas would not be as present as a substrate in the reaction

medium compared to the high-boiling COD. However, based on a previous study of Skrydstrup and co-workers, ethylene was readily hydroformylated using the Rh/6-DPPon catalyst conditions in a two-chamber setup (reference: Hammershøj et al. *Nat. Synth.* **2**, 243–250 (2023)). Therefore, if COD were to be problematic as a competitive substrate, we would favor a completely olefin-free Rh-precursor catalyst under these scenarios. Alternatively, a pre-mixed solution of pre-catalyst di-μ-chlorotetraethylene dirhodium(I) and ligand (6-DPPon) in DMSO:H_2O (95:5) should be well ventilated/degassed with nitrogen gas to remove any ethylene before adding it to the hydroformylation reaction chamber. However, we did not investigate this possibility or alternative Rh-precatalysts for the purpose of this revision.

<u>Changes in the Manuscript:</u> We added the following to the paragraph before the Radiolabeling setup: "We were further interested in whether the COD-ligand of the pre-catalyst can act as a competitive substrate and thereby consume valuable syngas. When analyzing the resin-wash solutions of ^{13}C-labeling experiments, no ^{13}C- signals in the aldehyde region could be detected by ^{13}C-NMR. However, in reactions without substrates other than the 1,5-COD of the precatalyst present, aldehyde signals were detected, alongside the isomerization product 1,3-COD (see Supporting Information, Section 2.8 for details). Since increased Rh-catalyst loadings (and thus increased COD ligands) overall improved peptide conversion substantially (Figure 3a, entry 2–4), no considerable influence of COD on the peptide yield is expected. A completely olefin-free Rh-precatalyst would be optimal but was not further envisioned for this project."

<u>Changes in the Supporting Information:</u> We added a new chapter 2.8 to the Supporting Information which details the "Experiment 1 and 4" (title marked with track changes, but the content of the chapter and experiments were added without track changes).

<u>Details for Experiment 1:</u>

In the first experiment, the standard conditions (as shown in the manuscript, Figure 3a) were repeated on the hexapeptide-model substrate **2SM** but using ^{13}CO to facilitate detection of formylation products by ^{13}C-NMR. In addition, deuterated solvents (DMSO-d_6:D_2O, 95:5) were used during the hydroformylation step. The reaction is shown in Scheme 1, together with the potential side products that may form with 1,5-COD.

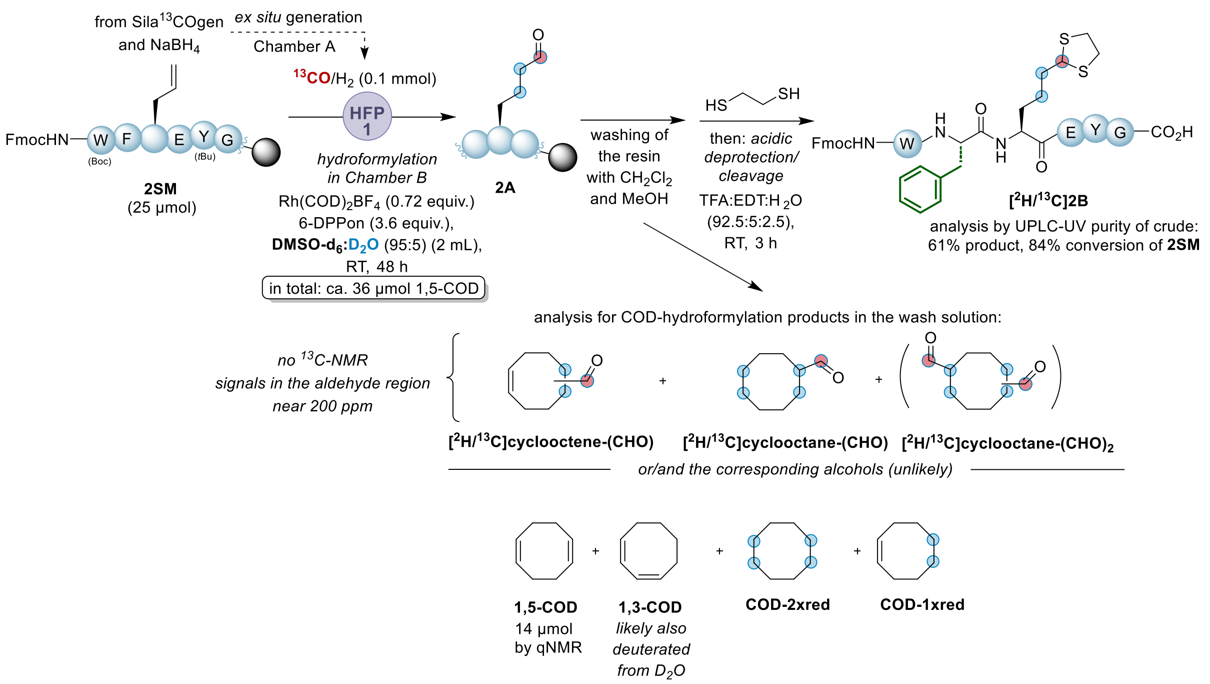

Scheme 1. Hydroformylation reaction with **2SM** using $^{13}CO/H_2$ in DMSO-d_6:D_2O, 95:5; and analysis for 1,5-COD hydroformylation products in the resin-wash solution by ^{1}H-NMR, ^{13}C-NMR and GC-MS.

The reaction gave a slightly lower conversion of the starting material **2SM** compared to the results shown in the manuscript (reason not investigated further) and yielded 61% of **[^{13}C, ^{2}H]2B**. ^{13}C-NMR analysis revealed the characteristic ^{13}C-shift of the [^{13}C]thioacetal **2B** at 52.9 ppm (Figure 1).

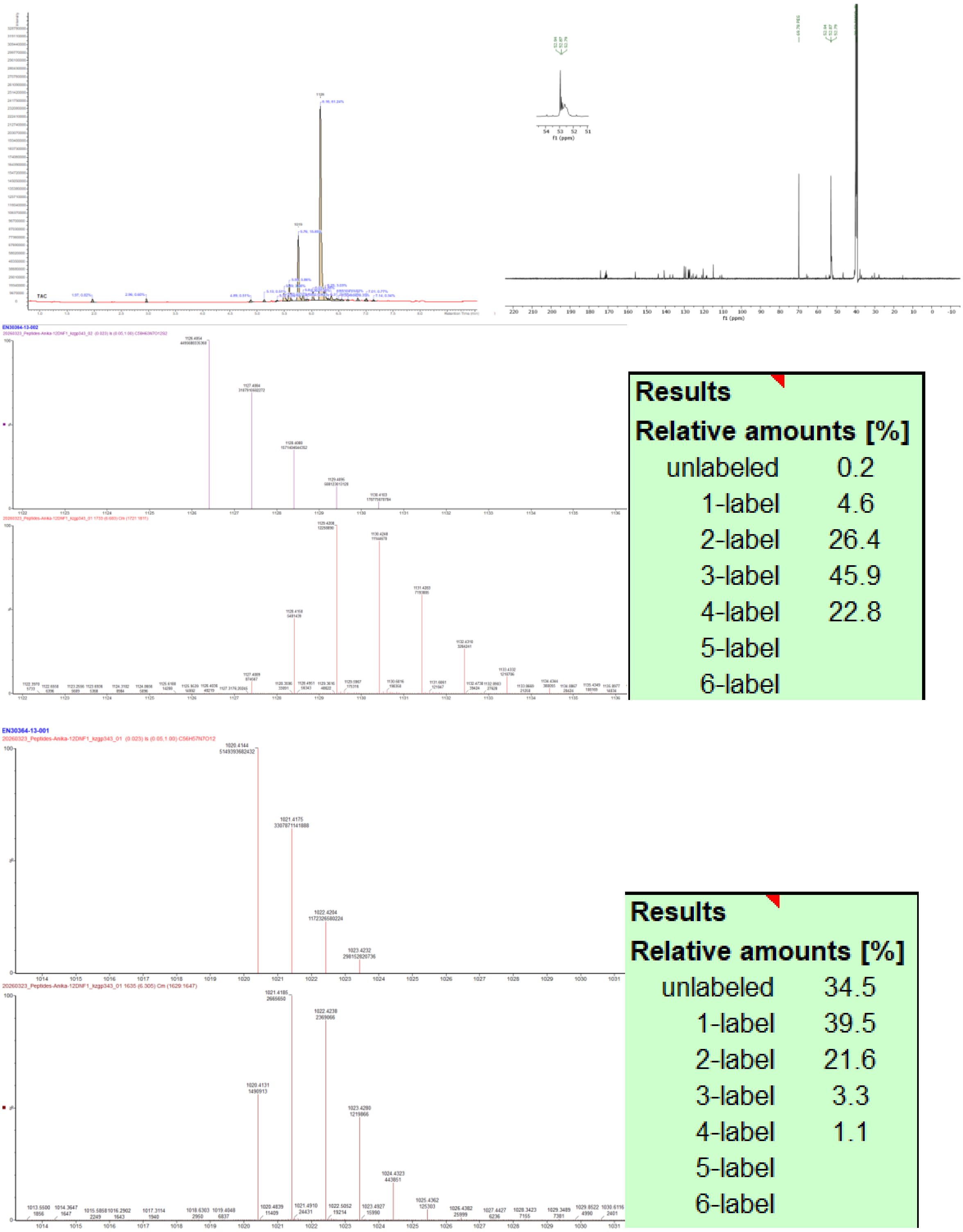

Figure 1. **Top-Left:** UPLC-UV chromatogram of the crude peptide, revealing 61% yield by UPLC-UV purity. **Top-Right:** ^{13}C-NMR of crude peptide **[^{13}C, ^{2}H]2B**, and a carbon isotope label at 52.9 ppm of the ^{13}C-labeled thioacetal. **Middle:** HR-MS analysis of analyte **[^{13}C, ^{2}H]2B** (bottom) vs. predicted unlabeled molecule (top), shows an incorporation of ca. 2.86 stable isotopes/molecule, i.e. ca. 0.99 ^{13}C/molecule, and 1.87 ^{2}H/molecule. **Bottom:** as a side note, the HRMS of the assigned starting material signal in the crude is added, which has a deuterium incorporation of 0.97 ^{2}H/molecule, HR-MS analysis of analyte **[^{2}H]2SM** (bottom) vs. predicted unlabeled molecule (top).

The resin wash solution after hydroformylation containing DMSO-d_6, D_2O, MeOH, CH_2Cl_2 and 0.1 mmol internal standard 1,3,5-trimethoxybenzene (TMB) was then analyzed for ^{13}C-labeled COD

hydroformylation products (Figure 2). Quantitative ^{1}H-NMR indicated recovery of ca. 14 µmol 1,5-COD of the initial 36 µmol used. Since D_2O was used in the solvent, any potential COD-derived aldehydes formed would be masked by incorporated deuterium in the ^{1}H-NMR spectrum. However, because no ^{13}C-aldehyde signals in ^{13}C-NMR appeared in the near 200 ppm region, we concluded that no detectable ^{13}C-aldehyde was incorporated, i.e. no formation of COD-hydroformylation products could be detected. Reductive hydroformylation of 1,5-COD, i.e. reduction to alcohols, may be more difficult to detect due to the ^{13}C-resonances in the 60 ppm region. However, reductive hydroformylation is rather unlikely with the mild conditions used and since the peptide itself was not undergoing reductive hydroformylation either. Therefore, we did not quantify for formation of alcohols. Another side reaction that can occur with 1,5-COD is isomerization of the double bond or reduction (which would also lead to ^{2}H-incorporation when using D_2O, and masking of the signals). The presence of the washing solvent dichloromethane in ^{1}H-NMR analysis overlaps with potentially isomerized COD that may have been formed. The formation of cyclooctane was not further investigated, but no major signal was observed in the ^{1}H-NMR in the 1.5 ppm region. Noteworthy, in the following experiment (Experiment 2), part of cyclooctadiene (despite its high boiling point of 150 °C) could be also detected in the syngas releasing chamber. This explains (in part) why only 14 µmol of 1,5-COD were recovered in the hydroformylation chamber. The presence of additional non-identified COD-derived products cannot be excluded either.

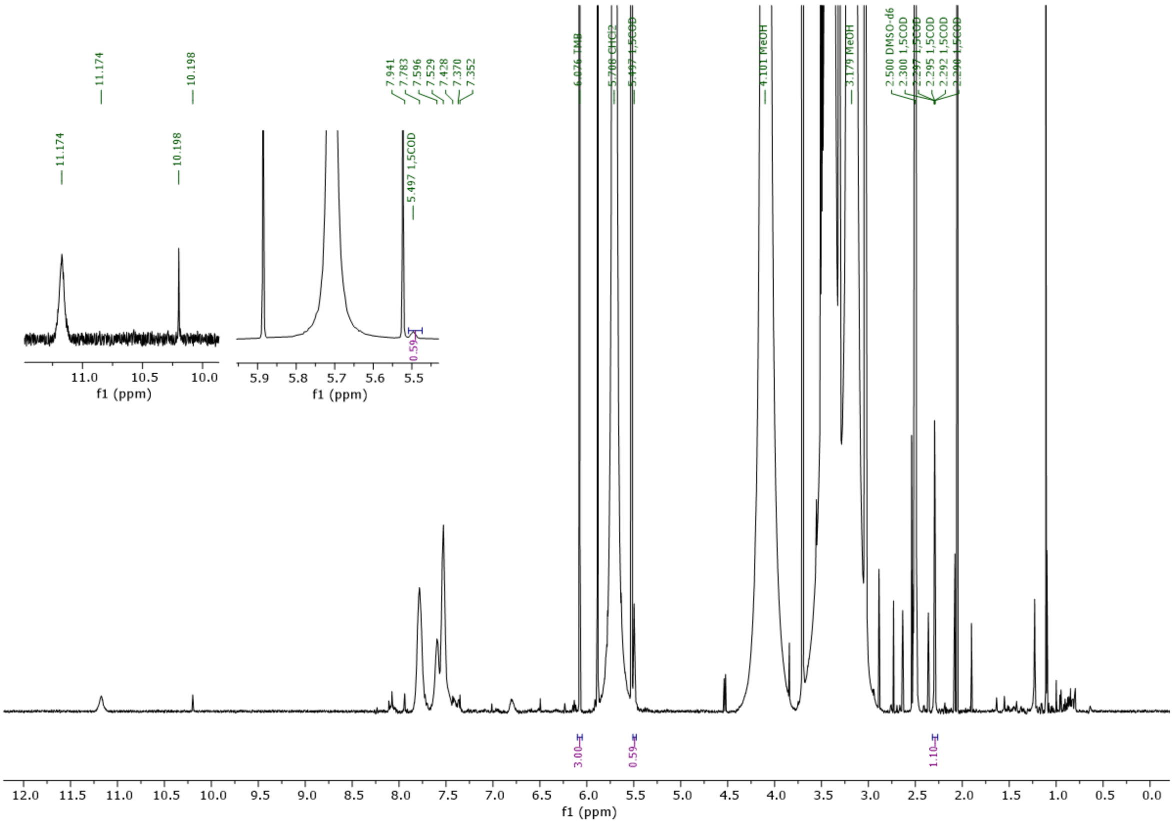

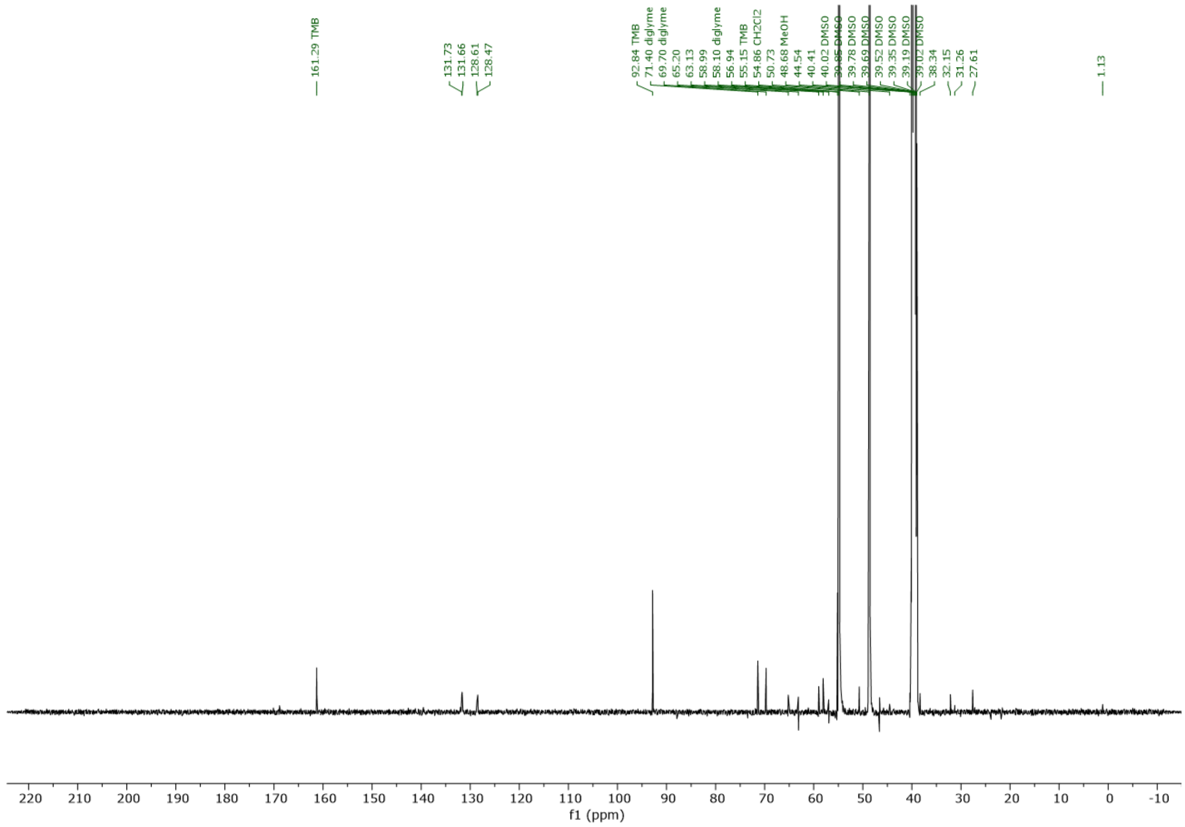

Figure 2. ^{1}H- and ^{13}C-NMR analysis of the resin wash solution after hydroformylation, containing DMSO-d_6, D_2O, MeOH, CH_2Cl_2 and 0.1 mmol internal standard 1,3,5-trimethoxybenzene (TMB), to analyze for ^{13}C-labeled COD-derived hydroformylation products. Quantitative ^{1}H-NMR showed presence of only 14 µmol of 1,5-COD (of the initial 36 µmol used), but no ^{13}C-aldehyde signals (the resonance at 161 ppm in ^{13}C-NMR stems from 1,3,5-TMB).

GC-MS analysis of the waste fraction shows the major signal of the internal standard 1,3,5-TMB at 6.936 min, alongside 1,5-COD at 4.882 min, and a minor signal at 4.718 and 4.658 min, likely corresponding to deuterated isomerized COD (Figure 6, Row 1 and 2). The signal at 4.969 min is unknown (M=58.1, 59.1).

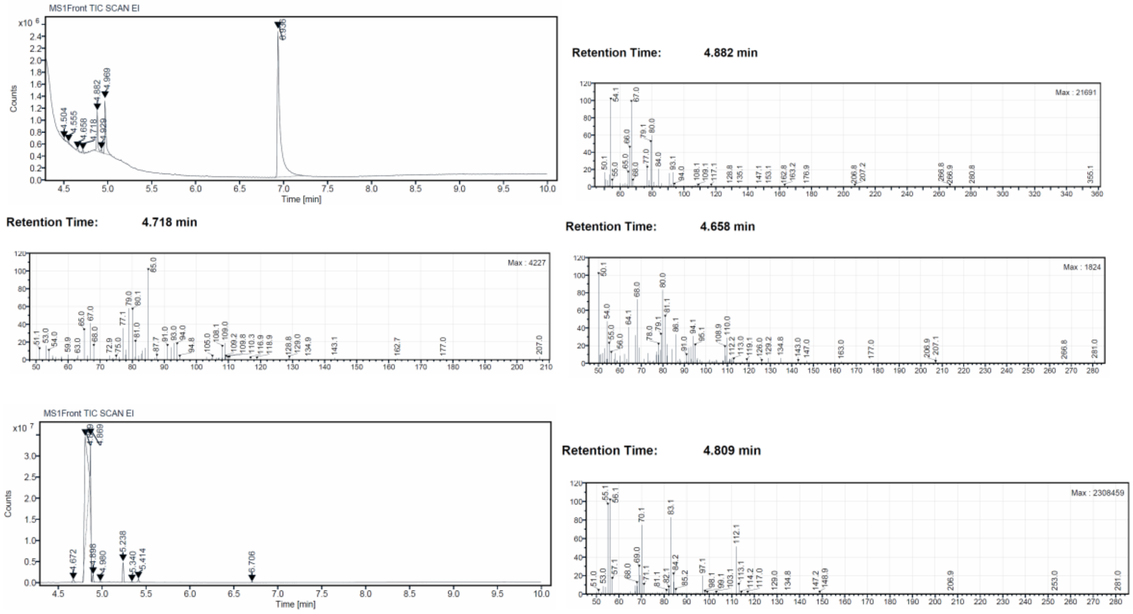

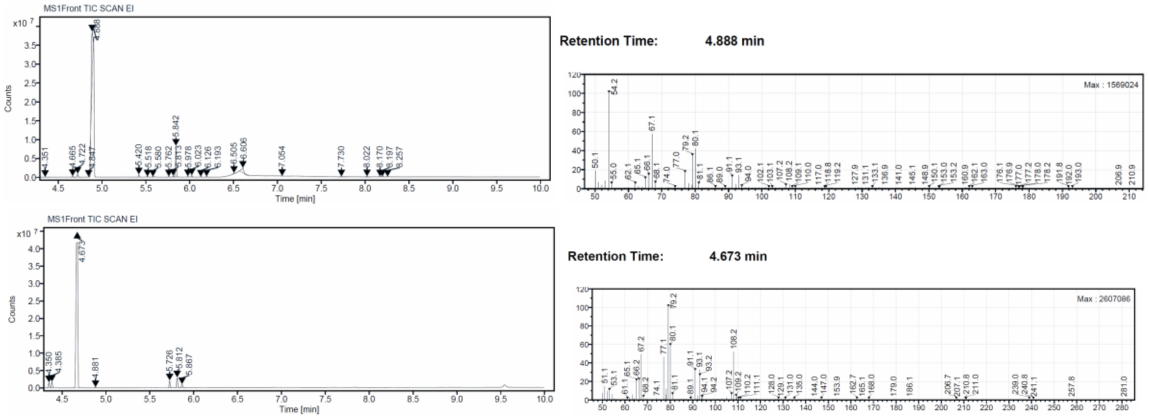

Figure 3. **Row1 and 2:** GC-MS analysis of the wash-solution; signal at 6.931 min is derived from 1,3,5-trimethoxybenzene (internal standard), and signal at 4.969 min is unknown (M=58.1, 59.1). **Row3:** cyclooctane as reference, M=112, 56.1. **Row4:** 1,5-cyclooctadiene as reference, fragment M=54.2. **Row5:** 1,3-cyclooctadiene as reference, M=108.2. Details for the GC-MS instrument Agilent Intuvo 9000 GC 5977C MS: split injection full scan method: split injection; 1 µL injection, split ratio 50:1; liner 250 °C; carrier gas He at 1.2 mL/min; column HP-5MS, 30 m x 250 µm x 0.25 µm; oven temp program: 40 °C (2.25 min); 40 °C/min to 250 (2.5 min); total run time 10 min; transfer line temperature: 250 °C; scan range: 50–550 m/z.

Finally, it is worth to mention that the NMR spectra and upcoming NMR spectra were recorded in DMSO-d_6 as solvent, without special sample preparation (prepared under atmospheric air). In fact, the pre-catalyst $Rh(COD)_2BF_4$ does in DMSO-d_6 not only give the 1,5-COD signals at 5.5 and 2.3 ppm, but also resonances at 4.0, 2.4 and 1.7 ppm. The pre-catalyst in $CDCl_3$ as NMR solvent only shows the signals at 5.4 and 2.5/2.6 ppm. The $Rh(COD)_2BF_4$/6-DPPon (1:5) mixture in DMSO-d_6 seems to show complete displacement of 1,5-COD from rhodium (no signals at 4, 2.4 and 1.7 ppm), and should therefore also be detectable as such in the crude qNMR measurements. The (unlikely) COD-religation to rhodium was not accounted for in the qNMR measurements of 1,5-COD recovery, but together with further loss of COD into the gas phase and the poor quality of NMR spectra (presence of solvents) these may be both sources of consistent errors in the qNMR measurements. The obtained yields should therefore not be regarded as absolute yields, but the relative composition of COD-derived products is more relevant.

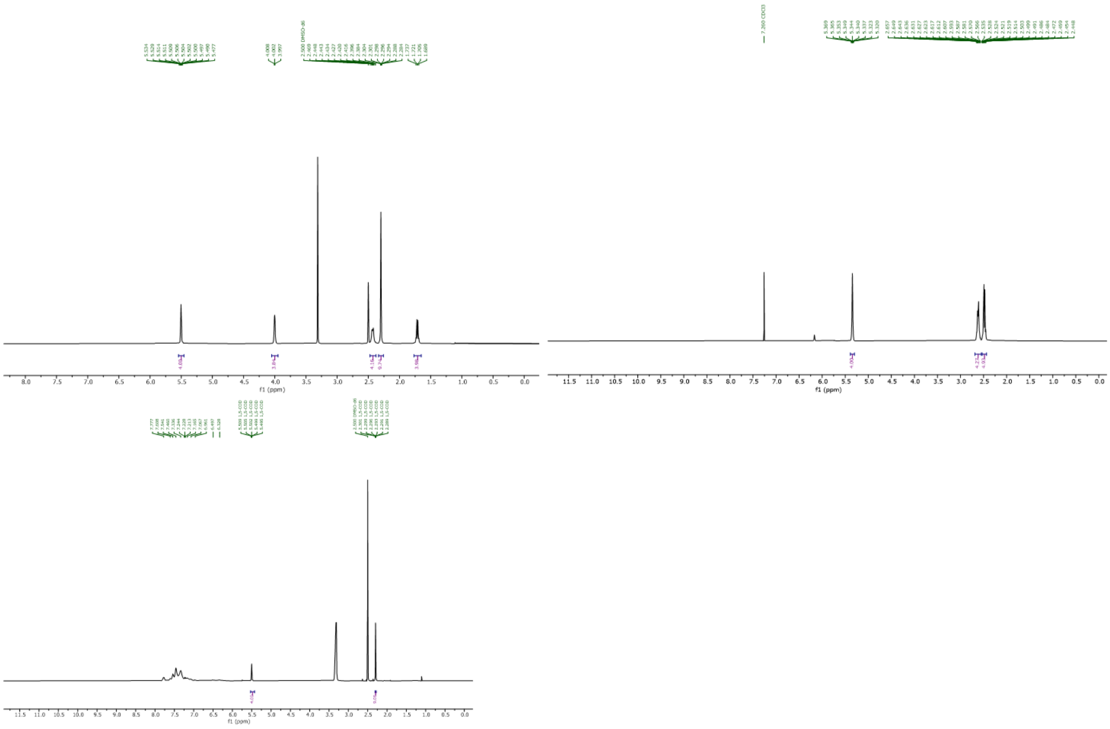

Figure 4. ^{1}H-NMR spectra. **Top-left:** $Rh(COD)_2BF_4$ in DMSO-d_6. **Top-right:** $Rh(COD)_2BF_4$ in $CDCl_3$. **Bottom:** $Rh(COD)_2BF_4$/6-DPPon catalyst mixture in DMSO-d_6/D_2O. NMR samples prepared under air.

Details for Experiment 2:

Next, we conducted an experiment with only 1,5-COD as potential substrate and using ^{13}CO (Scheme 2). While the $Rh(COD)_2BF_4$/6-DPPon (18 µmol, 1:5) concentration and syngas concentration (100 µmol) was kept the same as in the upper Experiment 1, we added on purpose additional 1,5-COD substrate (250 µmol), leading to a total amount of 286 µmol of 1,5-COD present. This equals 0.35 equiv. of syngas per 1,5-COD and means that a maximum of 100 µmol of COD-hydroformylation product (35% relative to 286 µmol 1,5-COD) can be obtained.

Under these concentrated conditions and without the allylglycine containing peptide as competitive substrate present, ca. 16 µmol (5%) of aldehyde signals were detected in quantitative ^{1}H-NMR, which likely corresponds to two different aldehyde species whose structure was not elucidated. In total, ca. 1.4% of aldehyde species 1 (multiplicity: doublet, J = 171.8 Hz) and 4% of aldehyde species 2 (multiplicity: doublet, J = 173.2 Hz) were formed, with the multiplicity as obtained by coupling of the aldehyde protons with the ^{13}C-nucleus. In the ^{13}C-NMR analysis, two aldehyde-labeled signals appeared at 204.95 and 204.70 ppm. The species were not further elucidated and assigned, but could likely be [^{13}C]cyclooct-4-enecarboxyaldehyde (isomers) or [^{13}C]cyclooctanecarboxyaldehyde (isomers) as previously found by Trzeciak et al (reference: Trzeciak et al. *J. Organomet. Chem.* **479**, 213–216 (1994)). In the experiment, ca. 111 µmol of 1,5-COD were recovered in the hydroformylation chamber B, and 43 µmol in the syngas-producing chamber A (which was in this case also analyzed by qNMR). This underscores that 1,5-COD can be lost in the gas phase, leading to no full recovery of COD-derived products.

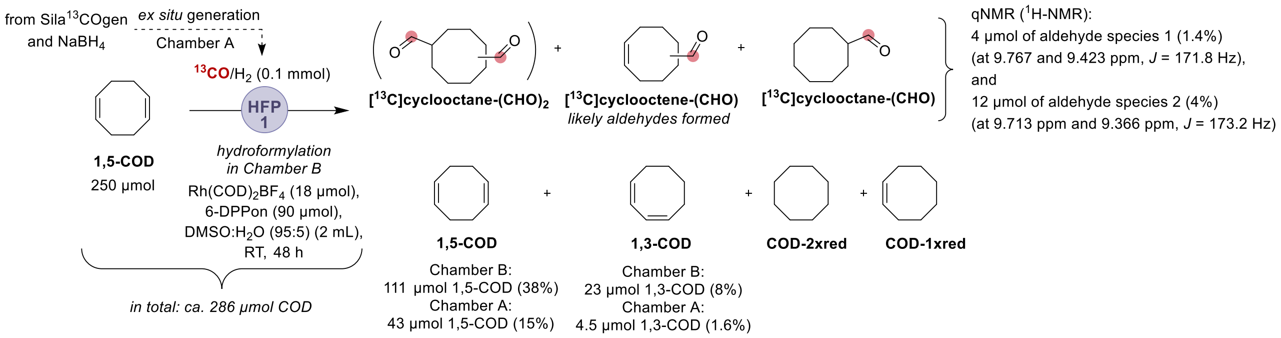

Scheme 2. Experiment 2 with only 1,5-COD as potential substrate in higher concentration (286 µmol/2 mL) during hydroformylation.

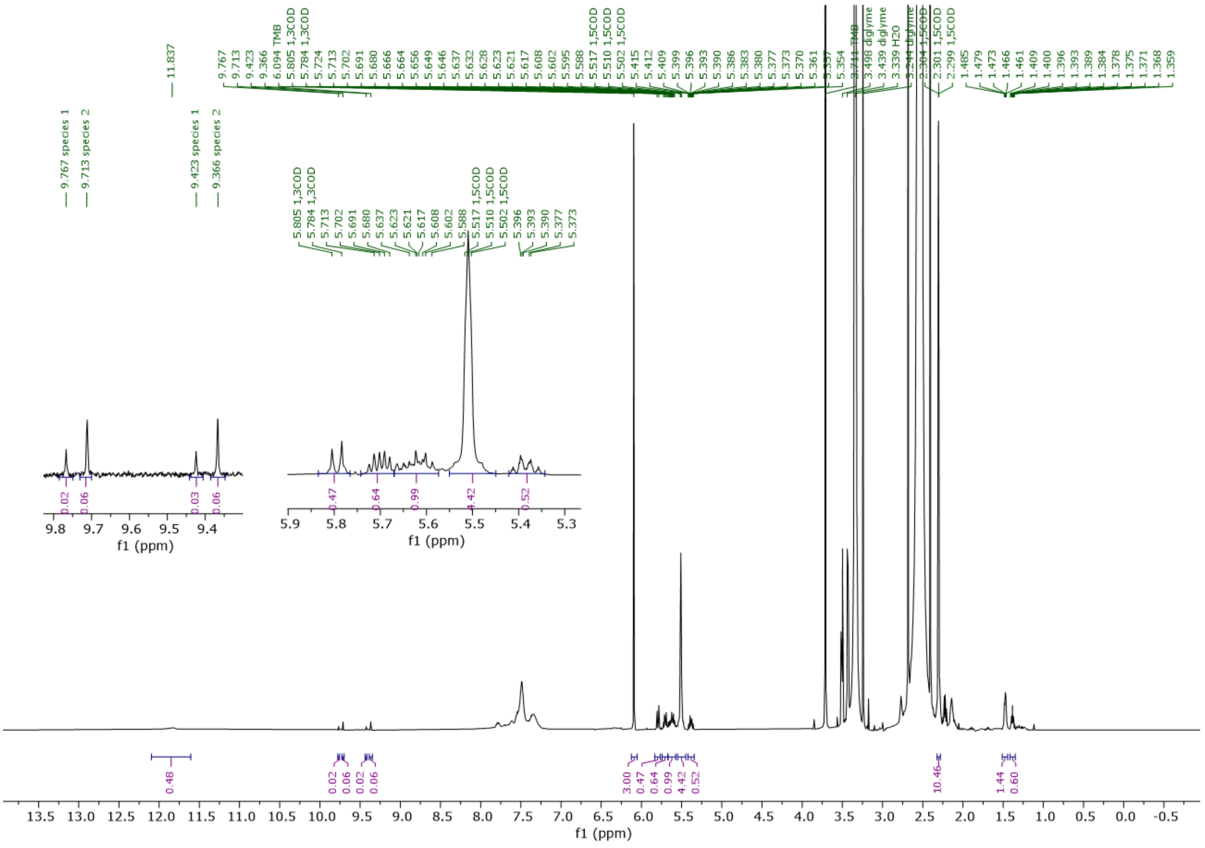

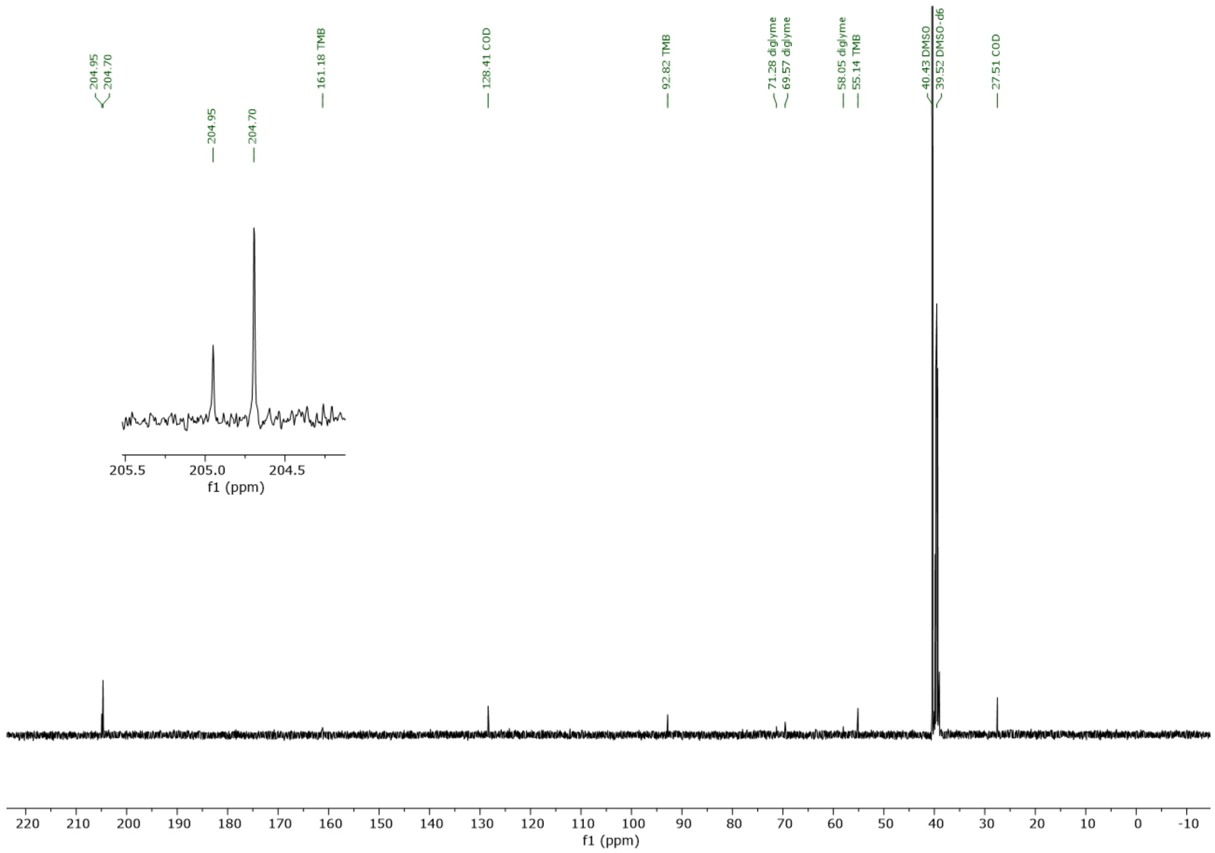

Figure 5. **Top:** ^{1}H-NMR, and **Bottom:** ^{13}C-NMR of the crude Experiment 2. References: ^{1}H-NMR report for 1,5-COD: ^{1}H NMR (500 MHz, DMSO-d$_6$) δ **5.50** (m, 4H), 2.30 (dt, J = 2.5, 1.1 Hz, 8H). ^{1}H-NMR report for 1,3-COD: ^{1}H NMR (500 MHz, DMSO-d$_6$) δ **5.78** (m, 2H), 5.60 (m, 2H), 2.14 (m, 4H), 1.47 (m, 4H).

GC-MS analysis of the washing solution shows the presence of 1,5-COD at 4.882 min (M=54.1, 80.1), alongside smaller amounts of 1,3-COD at 4.665 min (M=108.1), an unknown COD-isomer at 4.722 min (M=108.1), and an unknown signal at 4.969 min (M=58.1, 59.1). No formation of cyclooctane could be detected. The signal at 6.123 min likely is a [^{13}CHO]-derived signal (when comparing to the mass of Experiment 4, shown in upcoming pages), and has a mass of M=121.1, that likely derives from dehydration of the cyclooctene-carbaldehyde (isomers) in the MS (M-18).

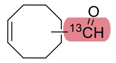

[^{13}C]cyclooctene-(CHO)
likely aldehydes formed
Exact Mass: 139.1078
$[M-18]^{+}$=121.1

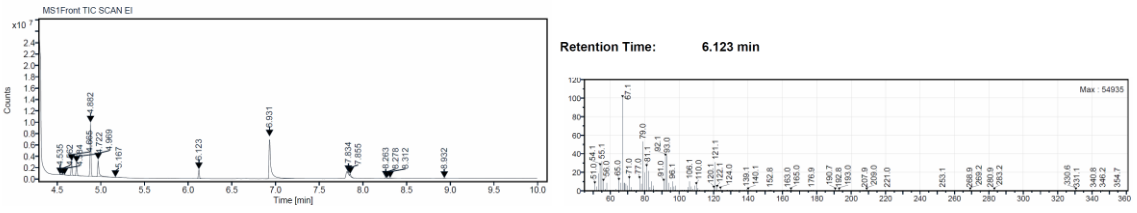

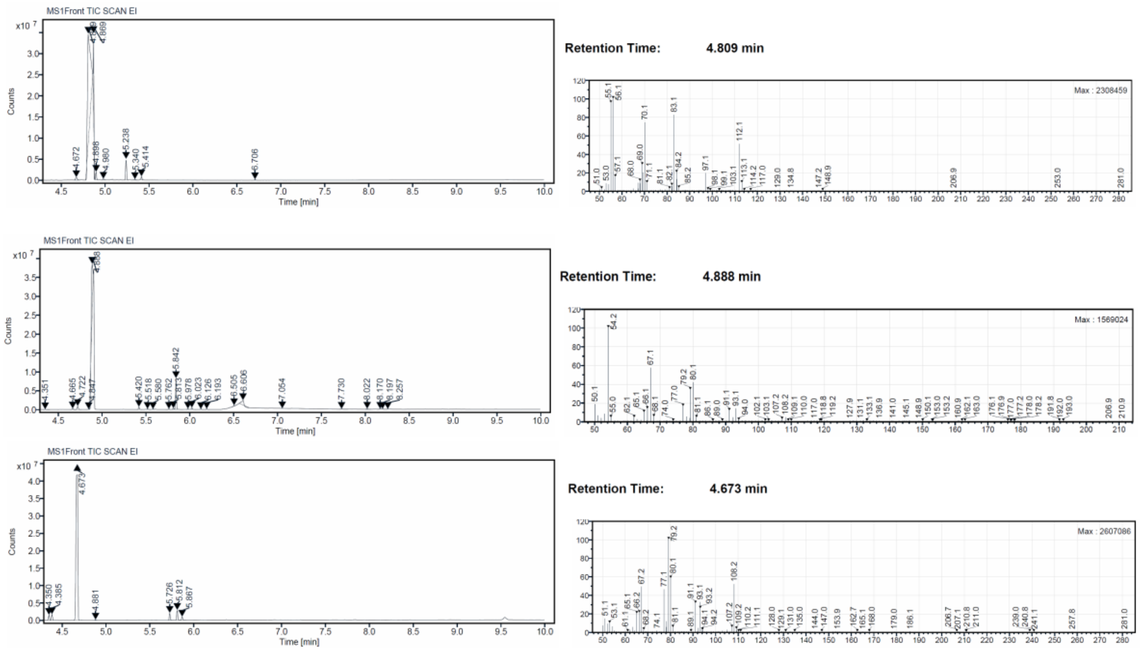

Figure 6. **Row1:** GC-MS analysis of the wash-solution, proposed aldehyde product at 6.123 min, M=121.1; signal at 6.931 min is derived from 1,3,5-trimethoxybenzene (internal standard). **Row2:** cyclooctane as reference, M=112, 56.1. **Row3:** 1,5-cyclooctadiene as reference, fragment M=54.2. **Row4:** 1,3-cyclooctadiene as reference, M=108.2. Details for the GC-MS instrument Agilent Intuvo 9000 GC 5977C MS: split injection full scan method: split injection; 1 µL injection, split ratio 50:1; liner 250 °C; carrier gas He at 1.2 mL/min; column HP-5MS, 30 m x 250 µm x 0.25 µm; oven temp program: 40 °C (2.25 min); 40 °C/min to 250 (2.5 min); total run time 10 min; transfer line temperature: 250 °C; scan range: 50-550 m/z.

Noteworthy, 1,3-COD was detected in relatively large amounts in chamber B (43 µmol, 15%) and chamber A (4.5 µmol, 1.6%), with the qNMR yields calculated using the resonance at 5.8 ppm. This observation may indicate that when we use isotope-labeled hydrogen gas, the label could likely be diluted during the hydroformylation process with ^{1}H-protons of 1,5-COD. This would occur if 1,5-COD-isomerization to 1,3-COD is mediated by the catalyst and exchanges the isotope-labeled hydrogen. If so, this may likely contribute to the <2 ^{3}H/molecule incorporation in tritium experiments (0.84 for **[^{3}H]11C**, 0.68 for **[^{3}H]12C**), among other factors, such as presence of water traces and the use of the waste recovery tritium bed for tritium labeling. Since we use for the tritium experiments a small reaction scale and relatively large amounts of syngas (17–45 equivalents $^{3}H_2$), we think that the isomerization may not be a substantial issue. In addition, for ^{3}H-labeled molecules we typically do not require high incorporations due to the high specific activity of tritium. As a side note, in the established hydrogen-isotope-exchange reaction used for tritium labeling, 1,5-COD-ligands are often used. Herein, the pre-catalytic Ir(I) of Kerr et al. is activated by hydrogenative loss of COD, thereby consuming isotope-labeled hydrogen gas (reference: Kerr et al. *ACS Catalysis* **5**, 402–410 (2015)). While academic work typically aims to keep the catalyst loadings in isotope labeling reactions as low as possible (in the latter case 6.5 mol%), pharmaceutical applications often use very high amounts of "catalyst" loading, as shown in the following two examples:

- example 1, Synthesis of [^{3}H]SCH 430765: uses a substrate (2.7 µmol), $[Ir(COD)(dppe)]PF_6$ (7.8 µmol), and tritium gas (11.1 µmol); yielding the product with 0.85 ^{3}H/molecule incorporation (reference: Hesk et al. *J. Labelled Compd. Radiopharm.* **61**, 533–539 (2018)).
- example 2, Synthesis of AZD5248: uses a substrate (4.9 µmol), $Ir(COD)PMe_2Ph(IMes)PF_6$ (2.5 µmol), and tritium gas (2.3 Ci, 40 µmol), yielding the product with 0.46 ^{3}H/molecule incorporation (reference: Allen et al. *J. Labelled Compd. Radiopharm.* **60**, 124–129 (2017)).

In general, it would be more elegant to develop a method with a catalyst that does not consume the isotope-labeled precursor.

Details for Experiment 3:

In a third experiment, we examined whether the hydroformylation reaction using the ligand BIPHEPHOS at elevated temperature (60 °C) (instead of 6-DPPon at RT) would make COD more prone to hydroformylation and to act as competitive substrate. For this, we repeated the test-scale optimization experiment where we used semaglutide (shown in the Supporting Information, Table S11, entry 4) with ^{13}CO, and analyzed the washing solutions after hydroformylation for presence of COD and COD-derived products (Table 1, entry 5).

Table 1. Analysis of hydroformylation reaction using ligand BIPHEPHOS for COD-derived hydroformylation products.

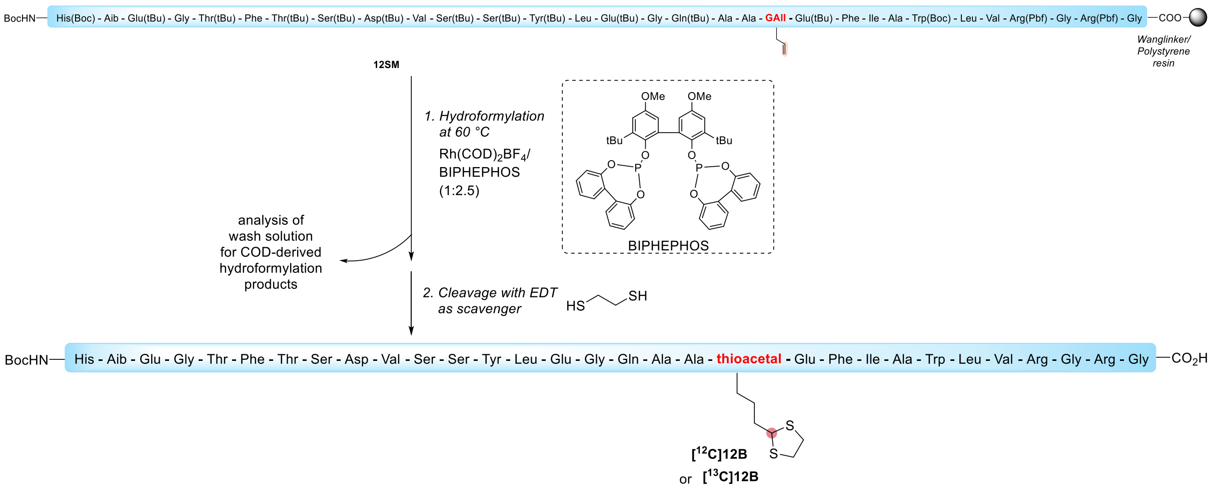

Entry	Conditions	Conversion to B, l:b [%][a,b]	Purity of remaining SM [%][a,b]	Analysis of wash solution for COD-derived products
**SI, entry 4**	BIPHEPHOS, 60 °C, Sila^{12}COgen	50	29	
**5**	BIPHEPHOS, 60 °C, using Sila^{13}COgen	52	15	qNMR: no COD detected, but also no ^{13}C-aldehyde signals in ^{1}H-NMR

Hydroformylation: Chamber A – SilaCOgen (0.2 mmol, 74 equiv.), granular $NaBH_4$ (0.2 mmol, 74 equiv.), diglyme (1 mL). Chamber B – **12SM** (ca. 2.7 µmol, 34 mg), $Rh(COD)_2BF_4$/BIPHEPHOS (1:2.5, 6.9 µmol), DMSO:THF, 17:3 (1 mL), 60 °C, 60 h, shaking. *Cleavage/Deprotection:* TFA:EDT:H_2O (92.5:5:2.5), 3 h, RT. [a]Yield estimated by LC-UV-MS after cleavage with TFA:EDT:H_2O (92.5:5:2.5) via comparing the peak integration. [b]Conversion to aldehyde **A** and conversion of **SM** estimated by cleaving a test-portion of the resin after hydroformylation with TFA:EDT:H_2O (92.5:5:2.5) and analyzing the purity of formed thioacetal **B** and remaining **SM**.

The UPLC-UV chromatogram of the crude peptide gave similar conversion to the product when we repeated the test scale reaction with Sila^{13}COgen, and the ^{13}C-NMR showed a single ^{13}C-resonance of the thioacetal as expected at 52.9 ppm (linear selectivity). Instead, the qNMR of the wash solution did not reveal an aldehyde signal that could originate from a ^{13}C-labeled COD-derived aldehyde (which needed to be a doublet in ^{1}H-NMR that could not be observed). In addition, no 1,5-COD was detected, which may be due to the elevated temperature, loss of the ligand into the gas phase when opening the

chamber, or loss into the syngas-releasing chamber. A small unidentified ^{13}C-resonance at 194.3 ppm was detected in the ^{13}C-NMR.

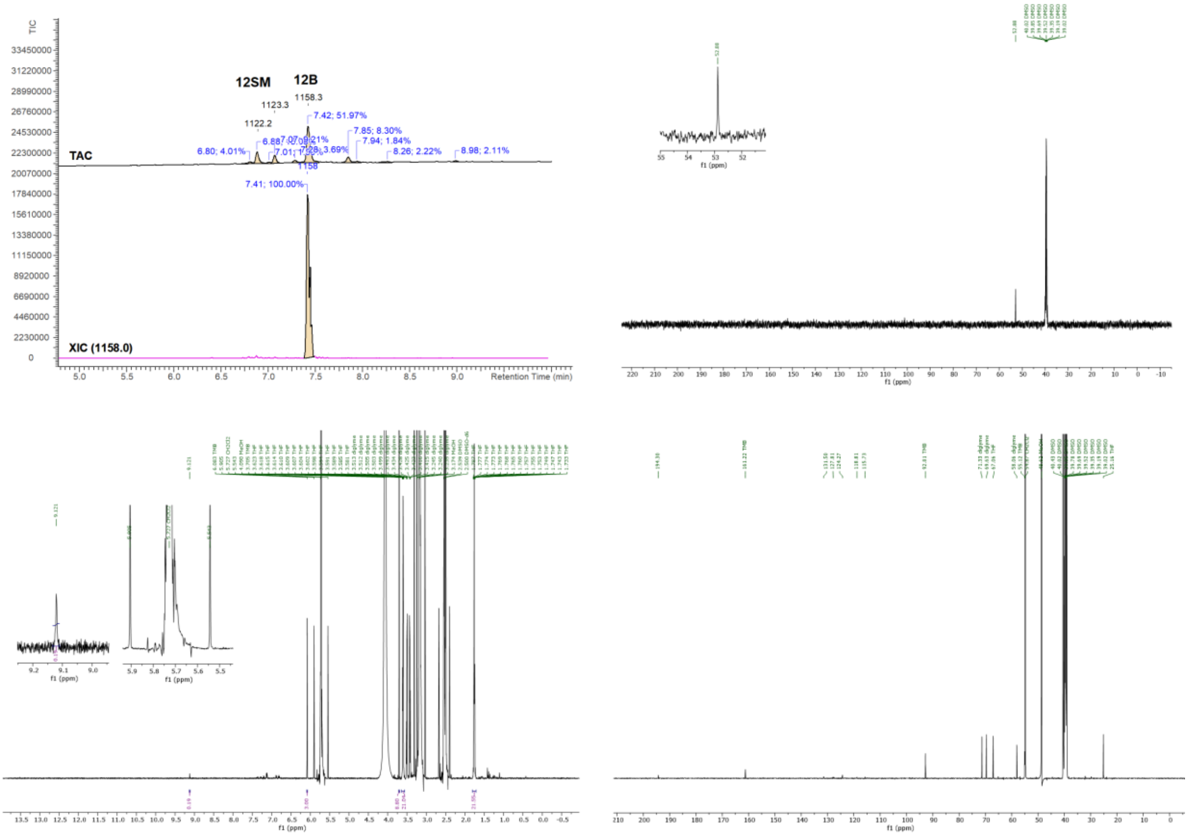

Figure 7. **Top-Left:** UPLC-UV chromatogram of the crude peptide **[^{13}C]12B** after cleavage with the scavenger EDT, entry 5. **Top-Right:** ^{13}C-NMR of the crude peptide. Note that in comparison to the UPLC-UV chromatograms shown in the Supporting Information, another UPLC instrument and gradient was used (due to availability), which is responsible for different low-resolution masses and retention times. Experiments shown here: 10–50% gradient. Experiments shown in Supporting Information, Table S11: 20–60% gradient. **Bottom-left:** qNMR of wash solution after hydroformylation, using 1,3,5-trimethoxybenzene (0.1 mmol) as internal standard; no 1,5-COD detected, signal at 9.121 ppm does not match the reaction where 286 µmol 1,5-COD starting material was employed (i.e. no ^{13}C splitting (doublet) detected). **Bottom-right:** ^{13}C-NMR of wash solution, a small unidentified ^{13}C-resonance at 194.3 ppm was detected in the ^{13}C-NMR.

<u>Details for Experiment 4:</u>

In a last experiment, we used more forcing hydroformylation conditions at 60 °C, in the solvent DMSO:THF (17:3), and using 0.2 mmol syngas (^{12}CO, H_2). Further, no substrate other than the 1,5-COD already present in the Rh-precatalyst was used (ca. 36 µmol 1,5-COD) and a reaction time of only 16 hours. These conditions were chosen as they may enable detection of COD-products, compared to Experiment 3, where they were not detectable. We compared the ligands 6-DPPon and BIPHEPHOS and analyzed the crude reaction in the hydroformylation chamber by qNMR analysis for COD-derived hydroformylation products. The results are shown in Table 2, the qNMR spectrum is shown in Figure 8, and GC-MS analyses in Figure 9.

The analyses reveal that when using ligand 6-DPPon two [^{12}C]aldehyde species are formed, i.e. 2 and 4 µmol with respective resonances at 9.59 ppm and 9.54 ppm (they fit the ^{13}C-splitting resonances of the aldehyde species in Experiment 2). Additionally, 4–5 µmol 1,5-COD were recovered and 4–5 µmol 1,3-COD were formed. Instead, when using the ligand BIPHEPHOS no aldehyde species were detected in the 9.5 ppm region, but a signal at 9.38 ppm appeared (ca. 1 µmol). In total, less than 1 µmol 1,5-COD was recovered, while major formation of 14 µmol isomerization product 1,3-COD were found. Whether the signal in the aldehyde region stemmed from the ligand itself or is a COD-

derived aldehyde was not further investigated. The GC-MS analyses were puzzling. In the reaction using 6-DPPon, a peak at 6.117 min with M=120.1 appeared, corresponding to the mass of [^{12}C]cyclooctene-carbaldehyde minus 18 (in the ^{13}C-labeling Experiment 2, this signal had a mass of M=121.1). Instead, for the reaction using BIPHEPHOS, this signal did not appear, but instead a trace peak with the mass of 138.1 was found at 6.313 min that would fit the parent mass of the [^{12}C]cyclooctene-carbaldehyde but no aldehyde resonance in ^{1}H-NMR other than the small trace at 9.38 ppm was found.

In summary, we could identify aldehyde resonances in the ^{1}H-NMR that are likely derived from COD, the GC-MS analysis was however inconclusive for the different ligands (different masses/retention times with different ligands, that are both related to the same structure).

Table 2. Analysis of the hydroformylation reaction using the ligands 6-DPPon or BIPHEPHOS at 60 °C without a substrate other than the 1,5-COD present in the pre-catalyst.

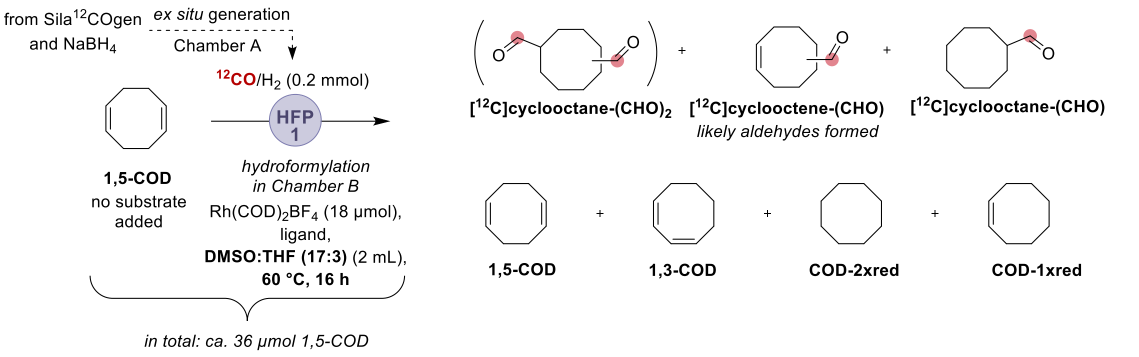

Entry	Ligand	Analysis of crude solution in Chamber B for COD products by qNMR[a]				
		Potential aldehyde species 1 at 9.59 ppm	Potential aldehyde species 2 at 9.54 ppm	Potential aldehyde species at 9.38 ppm	Recovery of 1,5-COD	1,3-COD
1	6-DPPon (90 µmol)	2 µmol	4 µmol	-	4–6 µmol	4–5 µmol
2	BIPHEPHOS (45 µmol)	-	-	1 µmol	<1 µmol	14 µmol

Hydroformylation: Chamber A – SilaCOgen (0.2 mmol), granular $NaBH_4$ (0.2 mmol), diglyme (1 mL). Chamber B – **no substrate** (but ca. 36 µmol 1,5-COD of pre-catalyst present), $Rh(COD)_2BF_4$ (18 µmol)/ 6-DPPon (90 µmol) or BIPHEPHOS (45 µmol), DMSO:THF, 17:3 (2 mL), 60 °C, 16 h, stirring. [a]Using 1,3,5-trimethoxybenzene (0.1 mmol) as internal standard.

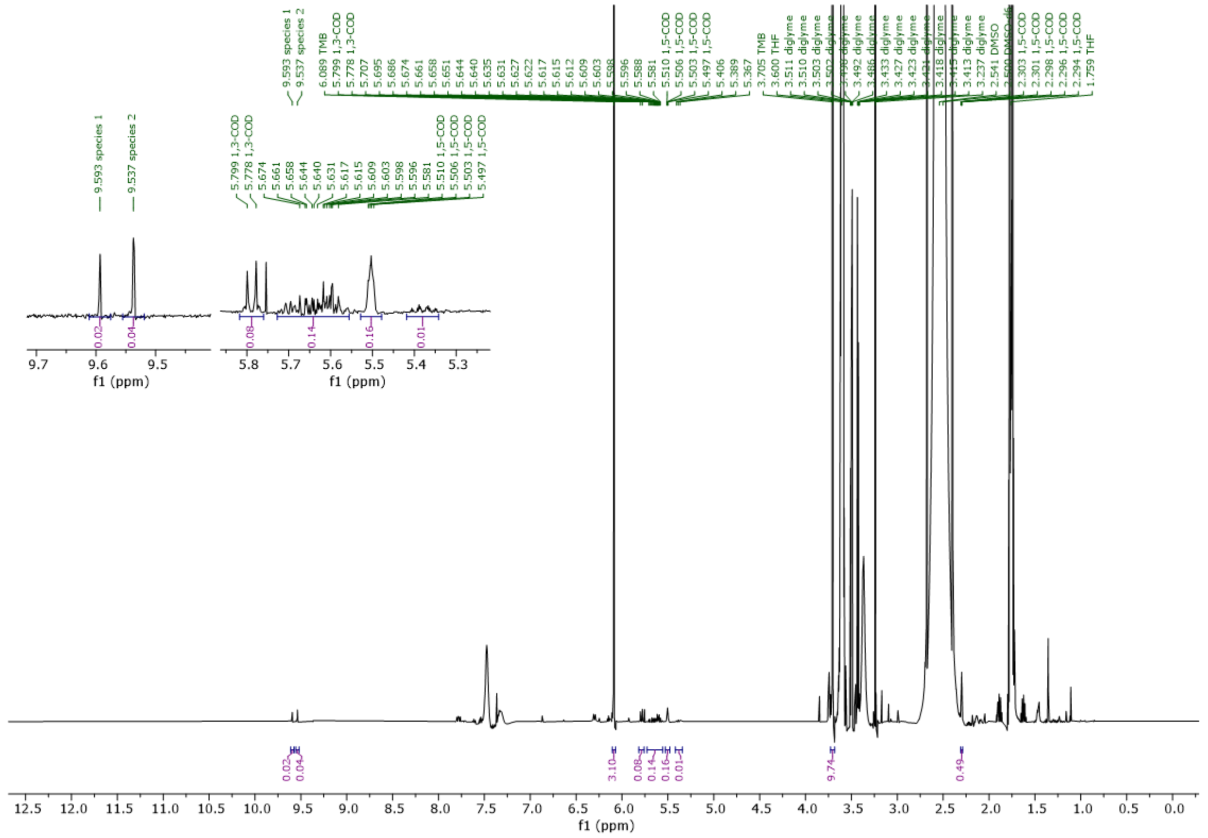

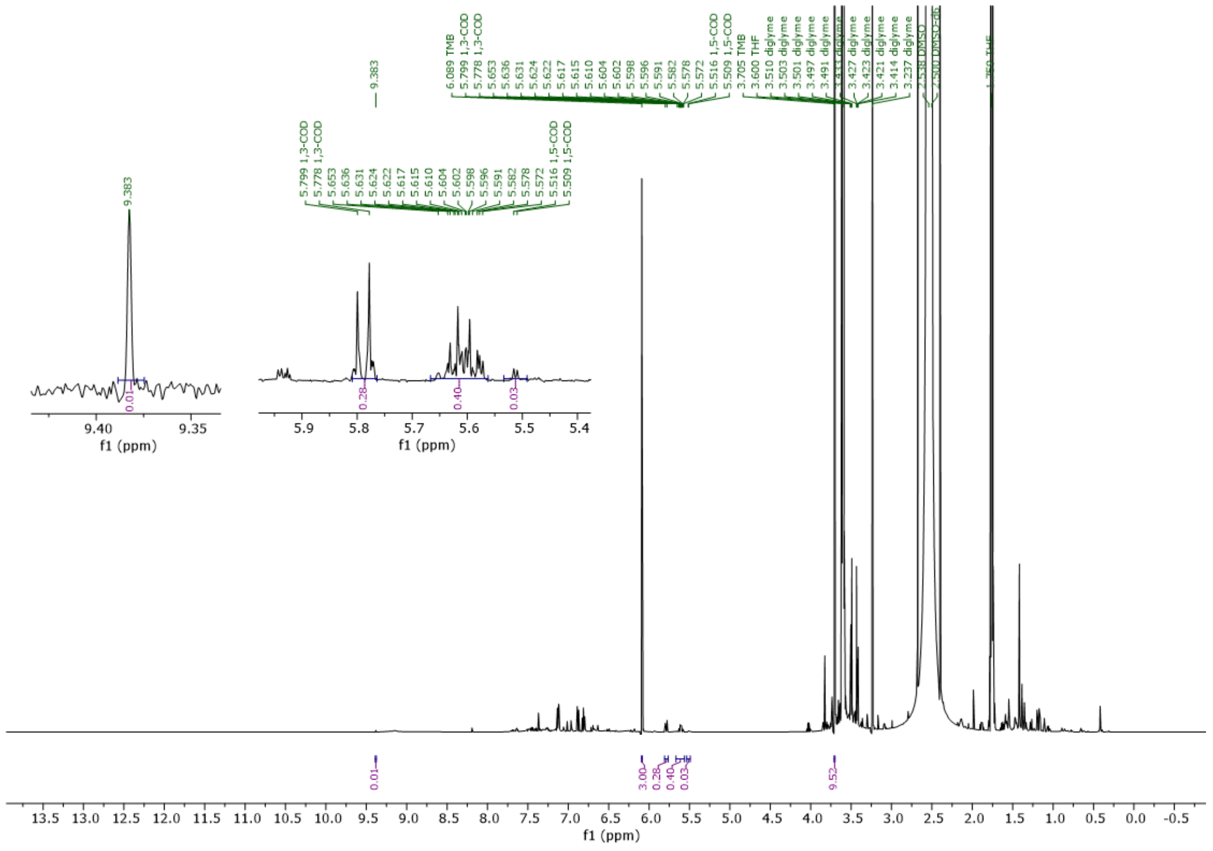

Figure 8. ^{1}H-NMR (qNMR) of the reaction mixtures of Experiment 4, using internal standard 1,3,5-trimethoxbenzene (TMB) (0.1 mmol). **Top:** using the ligand 6-DPPon. **Bottom:** using the ligand BIPHEPHOS.

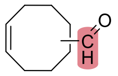

[^{12}C]cyclooctene-(CHO)
likely aldehydes formed
Exact Mass: 138.1045
$[M-18]^+$=120.1

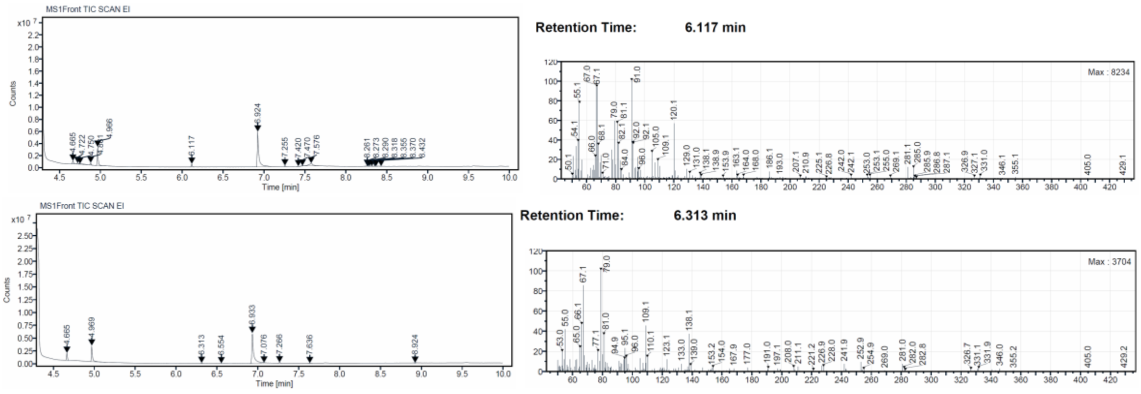

Figure 9. GC-MS analyses. **Top:** using the ligand 6-DPPon, mass 120.1 at 6.117 min found that likely corresponds to the [^{12}C]cyclooctene-carbaldehyde (M-18). **Bottom:** using the ligand BIPHEPHOS, mass 138.1 found in trace signal at 6.313 min that could correspond to [^{12}C]cyclooctene-carbaldehyde; inconclusive but may be an artifact.

<u>Catalyst loading experiments shown in the manuscript:</u>

In Figure 3a of the manuscript we showed a catalyst loading screen. Increasing the Rh-loading by a factor of 3.6 and 7.2 (which would lead to 7.2 and 14.4x more COD ligand, and 14.4 and 28.8x more COD-derived alkene) did lead to a significantly increased yield of the peptide. We therefore assume that the increasing amount of COD as a potential competitive substrate likely does not influence the yield significantly for the specific peptide used, while the increase in Rh-catalyst has an overall beneficial effect. Whether increasing amounts of catalyst may lead to higher isotope dilution for ^{3}H-labeling experiments could be an interesting future study, but was not further pursued in the frame of the revision for this project.

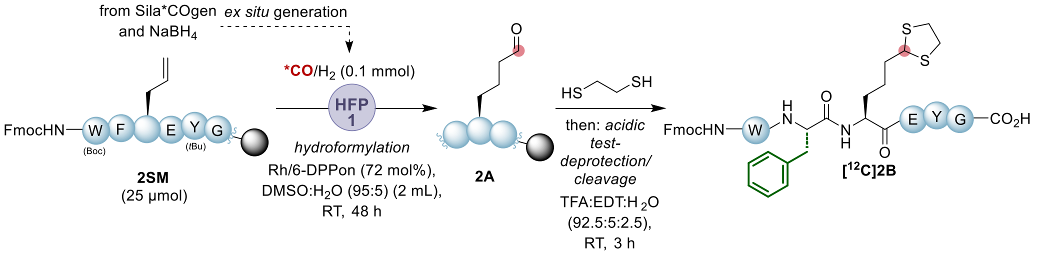

Entry	Change in hydroformylation-conditions	Conversion of **2SM**[a]	Yield of **2B**
1	Standard conditions	^{12}C: 96%	^{12}C: 91%
2	72 mol%, 60 h	100%	98%
3	36 mol%, 60 h	56%	52%
4	10 mol%, 60 h	32%	31%

Figure 10. Catalyst loading screen shown in the manuscript.

2. The SI clearly states that branched and linear isomers are generated in the hydroformylation and explains the otherwise unusual HPLC assignments. This information should be included directly in the main manuscript.

Changes in the manuscript: We added this sentence to page 7: "The major isomer **1B** was assigned as the linear thioacetal, while the minor UPLC-UV signal likely corresponds to branched thioacetal isomers that are formed from branched aldehydes (side product during hydroformylation, see Supporting Information for isomer details)."
We also added to each relevant figure titles that the Supporting Information shows detailed product isomer ratios.

3. In my opinion, the manuscript uses excessive and non-standard abbreviations. Some are not defined (e.g., TiPS, DODT), and some appear to be used inconsistently (e.g., CD vs DC). In many cases, writing out the full terms in the text or figures would improve readability.

Changes in the manuscript: We included the definition of TiPS and DODT in the description of Figure 3 (manuscript). To improve readability, we wrote out the non-standard abbreviations HFP, RA and DC (i.e. hydroformylation process, reductive amination, deprotection and cleavage). For "Conditions/Setups" HFP-1, HFP-2, HFP-3 we keep these names because they refer to differing setups.

4. The first paragraph on page 11 appears to describe a key finding that water improves reactivity, but no specific data are shown, and it is not clear on which substrate this was confirmed. The relevant data appear to be provided only in the SI. If this is an important conclusion, representative data should be included in the main text.

Answer: In the previous version of the manuscript, we have shown the direct comparison using neat DMSO vs. DMSO:H_2O (95:5) only once, in Figure 3a, entry 1 vs. 6, and also showed the UPLC-UV chromatogram. We agree with Reviewer 2 that a direct comparison should already appear in Figure 2.

Changes in the manuscript: We added to Figure 2e an additional column (to the right), in which the results for solvent variation are shown. Those were before only described in the Supporting Information.

5. Reactions are conducted at low concentrations (~5–10 µM), often in 1–2 mL of solvent. I did not see this parameter varied in the SI. Increasing the concentration might improve reaction rates and lead to full substrate conversion with fewer side reactions. Is this limited by the use of specialized glassware or other experimental constraints?

Answer: In the main manuscript, we varied the concentration parameter once, see Figure 3a, entry 1 (2 mL) vs. entry 5 (1 mL). For the more concentrated version, we obtained a similar/slightly lower yield. However, we assume that this effect is mostly due to the limitations in the glassware, as suggested by Reviewer 2. The glassware does not have a conical bottom part which would be optimal when working with small volumes. Since the reaction is heterogeneous (peptide-on resin) and shaken, smaller volumes may cause the resin to be spread above the solvent line, which reduces effective contact to the reaction solution and reproducibility. This would be especially pronounced when using smaller reaction volumes. Typical resin loadings of ca. 0.1–0.2 mmol/g and swelling further limit a minimum solvent level. A 25 µmol peptide scale would correspond to 125–250 mg resin which further swells when exposed to the solvent. Therefore, a smaller volume, despite the small µmol-scale, could lead to problems with irreproducibility in case the resin is not properly exposed to the solvent.

In the Supporting Information, the concentration was also varied once, however in an early optimization series of a resin-supported tripeptide. In this case, a tripeptide (15 µmol, corresponding to only 34 mg of peptide on a high-loading resin) in 0.5 mL vs. 1 mL THF was tested in a hydroformylation reaction. The more concentrated reaction gave almost double the yield, but we ultimately favored the more dilute conditions to ensure consistent contact of the resin to the reaction solution and mass transfer for reproducibility (see Supporting Information, Table S2).

6. The results shown in Figure 6 and the corresponding discussion could potentially be removed from the paper. The Wittig reaction does not appear optimized, is not particularly surprising, and seems to have limited immediate relevance to the isotope-labeling work.

Answer: We thank Reviewer 2 for this suggestion and implemented it. We understand that the late-stage functionalization part (which was initially intended as an outlook) would lead to confusion since it is not relevant for the isotope-labeling work (as highlighted by Reviewer 2). We followed their advice and omitted this part in the manuscript.

<u>Changes in the manuscript/SI:</u> We completely removed the Wittig/ hydrogenation products from both the manuscript and Supporting Information. We remove thioacetal **15B** from the manuscript but leave it in the Supporting Information, since it also fits the labeling part of the manuscript (optimization of the hydroformylation with trapping of aldehyde as thioacetal).

7. It is not clear from the data or discussion whether the extent of ^{3}H/^{14}C labeling of the targets is sufficient for the applications described in the introduction. If this is sufficient, that should be explicitly stated, if not this limitation should be acknowledged.

In this manuscript, both **[^{14}C]2C** and **[^{14}C]11C** were prepared at a level of 0.05 ^{14}C/molecule. This was pre-determined by the use of 95:5 ^{12}CO vs. ^{14}CO in the starting ^{14}C-reagent in these experiments (^{14}COgen). This was done to limit the radioactive waste produced. A peptide with ca. 1 ^{14}C/molecule is likely acceptable for use in an *in vitro* experiment such as plasma protein binding or cross species metabolism. However, it may be too low for *in vivo* studies such as quantitative whole-body autoradiography (QWBA) or mass balance. That will depend on the molecular weight and dose of the peptide being given to the animal. The same constraint would hold for the human ADME study with conventional radioactive detection (liquid scintillation counting), but this level would be perfect for a study using accelerator mass spectrometry (AMS) detection. AstraZeneca has used a modified version of this methodology to label a very small peptide (2-mer) with ^{14}C and the QWBA worked perfectly. However, that data cannot be disclosed yet.

For tritium, both **[^{3}H]11C** and **[^{3}H]12C** were labeled with modest ^{3}H-incorporations of 0.84 and 0.68 ^{3}H/molecule respectively. This level of labeling is enough for nearly any DMPK application. Furthermore, this level of incorporation was somewhat impacted by our desire to keep our research as "green" as possible. In tritium chemistry, there is a main reservoir of tritium gas stored on uranium that has nearly pure ^{3}H. There is a second uranium bed reservoir that recovers excess tritium gas after reactions to prevent its release into the atmosphere. This reservoir is often contaminated with protons from previous reactions and the level of tritium gas to hydrogen gas is lower. We opted to use the second (waste) bed of tritium gas for this application to conserve our main tritium supply and to use what is generally just a waste product (albeit one that gets recycled). AstraZeneca has run several DMPK studies (QWBA and mass balance) since 2020 on labeled biologics including peptides with lower specific activity than is reported here. All these studies required a known and specific labeling pattern; this methodology provides a product with exactly those parameters. The tritium labeled peptides produced using this methodology might work for *in vitro* experiments (0.68 ^{3}H/molecule, 700 MBq/µmol, 19 Ci/mmol is a very typical level of labeling

for a small molecule for use in a binding experiment), but that is very dependent upon the affinity of the compound and the expression of the biological target.

Changes in the manuscript: "The low levels of 0.05 ^{14}C/molecule are pre-determined by using a dilution of 95:5, ^{12}CO:^{14}CO in these experiments, due to economic and environmental reasons. However, for real biological applications, more ^{14}CO could be used in relation to ^{12}CO (or only using ^{14}CO) to achieve higher specific activity of the product to enable easier detection in biological studies."

Changes in the Supporting Information in General Procedure GP5: "In tritium chemistry, there is a main reservoir of tritium gas stored on uranium that has nearly pure ^{3}H. There is a second uranium bed reservoir that recovers excess tritium gas after reactions to prevent its release into the atmosphere. This reservoir is often contaminated with protons from previous reactions and the ratio of tritium gas to hydrogen gas is lower. To make our chemistry greener, the second (waste) bed of tritium gas was used for this proof-of-concept study of the methodology, allowing to conserve our main tritium supply and to use what is generally just a waste product. The obtained lower tritium incorporations in the reactions (<2 ^{3}H/molecule) may be in part explained by using this waste reservoir, by the presence of water traces during the reaction, or potential hydrogen isotope dilution from 1,5-COD isomerization of the pre-catalyst."

Reviewer #3 (Remarks to the Author):

Late-Stage 14C/3H- Radiolabeling of Lysine Residues via 1 Hydroformylation of Peptides

Schick et al.

The authors present a neat method for the radiolabelling of peptides, involving an on-resin hydroformylation-reductive amination late-stage modification cascade. Peptides containing allyl- or homoallylglycine residues were synthesised using solid-phase peptide synthesis and then subjected to 1) hydroformylation with syngas in a two-chamber setup, followed by 2) reductive amination of the resulting allysine. The reaction conditions were carefully optimised and demonstrated to be applicable to a variety of peptides containing all relevant functional groups. These conditions were then used for radiolabelling, albeit with slight adaptations due to the use of 3H2 gas instead of an H2 gas surrogate. Radiolabelling of complex peptides such as 13-mer STAT3-Hel2A-2 and 31-mer semaglutide has also been demonstrated. In the final chapter, the authors discuss extending their reaction cascade to include a hydroformylation-reductive amination-Wittig reaction cascade for the late-stage diversification of resin-bound peptides.
I am somewhat conflicted about this study. I really like the first part, the hydroformylation-reductive amination cascade and its use for the radiolabeling of peptides. The conditions were carefully optimized and described in detail in the extensive supplementary material. It is really important that the authors provided such detailed information on the reaction conditions and setups. This will certainly enable others to successfully apply this method to their peptides. Only minor points need to be addressed in this part. However, the last section on late-stage diversification is very weak! My advice to the authors: invest more time and present a more mature study on the hydroformylation-reductive amination-Wittig reaction cascade, or omit this part entirely. With this study included, I cannot recommend the manuscript for publication in Nature Communications without really extensive revisions. However, if the authors decide to omit this part, I would be happy to recommend publication in Nature Communications, as the first part is of great importance.

Please see below for the points that require improvement prior to publication:

1) The phrase 'late-stage radiolabelling of lysine', as used in the title, is misleading, since it is technically the allylglycine residues that are modified. Perhaps the authors should revise the title to 'Late-Stage 14C/3H Radiolabelling of Resin-Bound Peptides via a Hydroformylation–Reductive Amination Cascade on Allylglycine Residues'.

Answer: We agree with Reviewer 3 that the title was misleading and appreciate the suggestion of a new title. We propose the following title: “Late-Stage Generation of ^{14}C/^{3}H-Radiolabeled Lysine Residues via Hydroformylation of Peptides”. This title still highlights that we generate a labeled natural amino acid (lysine), which is one of the key advantages of the method. To keep a shorter title (requirement 15 words or fewer), we would not include reductive amination since it is not the key step.

2) The authors annotate the introduced C1 building block with 12/13C in large parts of the manuscript. At first, it seemed to me that this was a mixture of both isotopes. However, if I understand correctly, it means 12C and 13C, since both are produced using the same method. This nomenclature should perhaps be clarified in one place or formulated more clearly in general.

<u>Changes in the manuscript and Supporting Information:</u> For clarity we decided to substitute “$^{12/13/14}$C” with “^{12}C or ^{13}C or ^{14}C” whenever it appeared in the manuscript and in the Supporting Information. This was also done for “Sila$^{12/13}$COgen” which we substituted with Sila^{12}COgen or Sila^{13}COgen. In the schemes, * was used.

3) The authors had to modify the conditions considerably for semaglutide to make the reaction work. Are these modified conditions generally superior to other conditions, or are they only optimal for semaglutide? Could these reaction conditions also be used for the other test peptides, for which the yield and selectivity were low?

Answer: This is an interesting and relevant remark. The main change in the hydroformylation reaction for semaglutide was using elevated temperature of 60 °C to improve starting material conversion and using the ligand BIPHEPHOS to improve product selectivity. In addition, we used more syngas equivalents. We should, as Reviewer 3 highlights, show for one of the other peptides how they perform in the hydroformylation reaction using BIPHEPHOS as ligand.

To answer the first question of Reviewer 3, the changed conditions are optimal for semaglutide (which was not sensitive to high temperatures), but may not be otimal for all other peptides. We would advise that, in case a new peptide sequence is tested in the hydroformylation reaction and the reaction may need to be re-optimized for this specific sequence, to first start the screen or optimization with mild room temperature conditions using 6-DPPon. First, this simplifies the experimental setup (no need for specialized adapter that connects the two-chamber system to a shaker that also heats). Additionally, some peptide sequences may be sensitive to the higher temperatures that are (typically) used with BIPHEPHOS. Instead, 6-DPPon is a ligand that is known to be active under ambient temperature, pressure and aqueous conditions (Straub et al. *Adv. Synth. Catal.* **355**, 2071–2075 (2013)). This was why we used 6-DPPon as the standard ligand for our reactions. In addition, water compatibility of the ligand should be assured since we have shown that for some peptide sequences the addition of water has a positive effect in preventing the formation of undesired cyclization side products. Based on reported literature, we assumed that BIPHEPHOS (being a phosphite-based ligand) should be prone to decomposition under aqueous conditions (reference 1: Vossen et al. *ACS Sustainable Chemistry & Engineering* **12**, 10665–10677 (2024); reference 2: Zhang et al. *ACS Catalysis* **6**, 7554–7565 (2016)).

To better address the question of Reviewer 3, we conducted a couple of additional reactions to study the generality of the ligand BIPHEPHOS (shown in detail in the upcoming pages):

- Experiment 5: addition of 5% water to the hydroformylation reaction of semaglutide using the ligand BIPHEPHOS. This experiment was performed to understand whether aqueous conditions (needed for peptides prone to cyclization) are compatible with the BIPHEPHOS ligand. The results revealed (to our surprise) comparable reactivity between the reaction with and without 5% water. While we did not study the ligand stability itself, the hydroformylation reaction using BIPHEPHOS seemed to be compatible with the aqueous conditions tested, and we could move on to testing the aqueous reaction conditions with BIPHEPHOS on another peptide (Experiment 6).
- Experiment 6: the BIPHEPHOS conditions were applied to the model hexapeptide using only 4 equiv. syngas. The BIPHEPHOS reaction conditions at 60 °C allow the formation of the desired product in high amounts, but alongside side products such as hydrogenated starting material. If a new peptide sequence is tested for the hydroformylation-reductive amination sequence, we would recommend to conduct the reaction with 6-DPPon at room temperature. If low conversion is obtained, higher catalyst loadings could be tested, as well as higher temperatures, and change to the BIPHEPHOS ligand. However, these conditions may sacrifice some starting material on formation of side products.

<u>Changes in the manuscript:</u> Experiment 6 was added to the optimization table in Figure 3a, entry 7 in the main manuscript. Changes in the text: "An alternative hydroformylation ligand BIPHEPHOS was tested at 60 °C, 36 mol% catalyst loading and 60 hours reaction time. High starting material conversion (96%) provided the product **[^{13}C]2C** in high yield (61%), but alongside ca. 19% hydrogenated starting material (entry 7)."

<u>Details for Experiment 5:</u>

First, we decided to repeat a test scale reaction where we used BIPHEPHOS on semaglutide, but with additional 5% water as co-solvent, to test whether the hydroformylation reaction using BIPHEPHOS is sensitive to aqueous conditions. The results below show that this seems to not the case.

Table 3. Analysis of hydroformylation reaction using ligand BIPHEPHOS for COD-derived hydroformylation products.

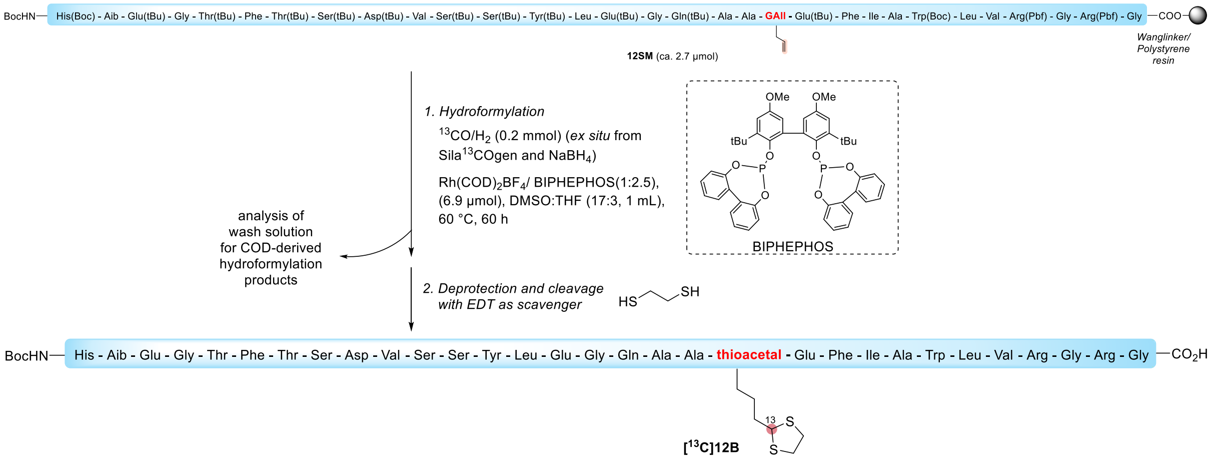

Entry	Conditions	Conversion to B, l:b [%][a,b]	Purity of remaining SM [%][a,b]
**5**	BIPHEPHOS, 60 °C, using Sila^{13}COgen, in DMSO:THF (17:3)	52	15

**6**	BIPHEPHOS, 60 °C, using Sila^{13}COgen, in **DMSO:THF:H_2O (17:3:1)**	51	18

Hydroformylation: Chamber A – Sila^{13}COgen (74 equiv.), granular $NaBH_4$ (74 equiv.), diglyme (1 mL). Chamber B – **12SM** (ca. 2.7 µmol, 34 mg), Rh/BIPHEPHOS (1:2.5, 6.9 µmol), DMSO:THF, 17:3 (1 mL), 60 °C, 60 h, shaking. *Cleavage/Deprotection:* TFA:EDT:H_2O (92.5:5:2.5), 3 h, RT. [a]Yield estimated by LC-UV-MS after cleavage with TFA:EDT:H_2O (92.5:5:2.5) via comparing the peak integration. [b]Conversion to aldehyde **A** and conversion of **SM** estimated by cleaving a test-portion of the resin after hydroformylation with TFA:EDT:H_2O (92.5:5:2.5) and analyzing the purity of formed thioacetal **B** and remaining **SM**.

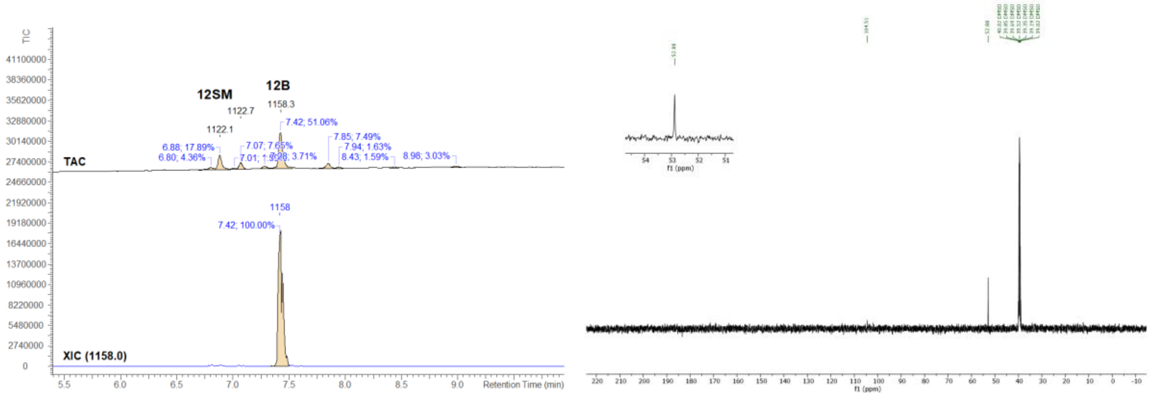

Figure 11. **Left:** UPLC-UV chromatogram of the crude peptide, entry 6, using 5% water as co-solvent. **Right**: ^{13}C-NMR of the crude peptide **[^{13}C]12B** after cleavage with EDT. Note that in comparison to the UPLC-UV chromatograms shown in the Supporting Information, another instrument and gradient was used, being responsible for different low-resolution masses and retention times. Here: 10–50% gradient. In Supporting Information, Table S11: 20–60% gradient.

<u>Details for Experiment 6:</u>

Based on Experiment 5, yield and selectivity (determined by UPLC-UV purity, and single ^{13}C-NMR label) was not affected by addition of 5% water using the small scale semaglutide conditions. Upon the request of Reviewer 3, we decided to apply the ligand BIPHEPHOS at 60 °C and in DMSO:THF (17:3) to a hexapeptide and similar conditions that were described in the manuscript in Figure 3a, entry 3 (with catalyst Rh/6-DPPon at room temperature). These conditions used a larger reaction scale (25 µmol), 36 mol% catalyst, 5% water as co-solvent to avoid cyclization, and using only 4 equiv. syngas, with a reaction time of 60 hours. Using BIPHEPHOS at 60 °C instead of 6-DPPon at room temperature allowed higher starting material conversion, with high conversion of 70% to **[^{13}C]2B** alongside hydrogenated starting material, while **[^{13}C]2C** was yielded in a slightly lower yield of 61%.

In summary, the BIPHEPHOS reaction conditions at 60 °C allow the formation of the desired product in high amounts, but alongside side products. If a new peptide sequence is tested for the hydroformylation-reductive amination sequence, we would recommend to conduct the reaction with 6-DPPon at room temperature. If low conversion is obtained, higher catalyst loadings could be tested, as well as higher temperatures. Change to the BIPHEPHOS ligand can be also an alternative, as shown with this experiment, but may sacrifice some starting material on formation of side products.

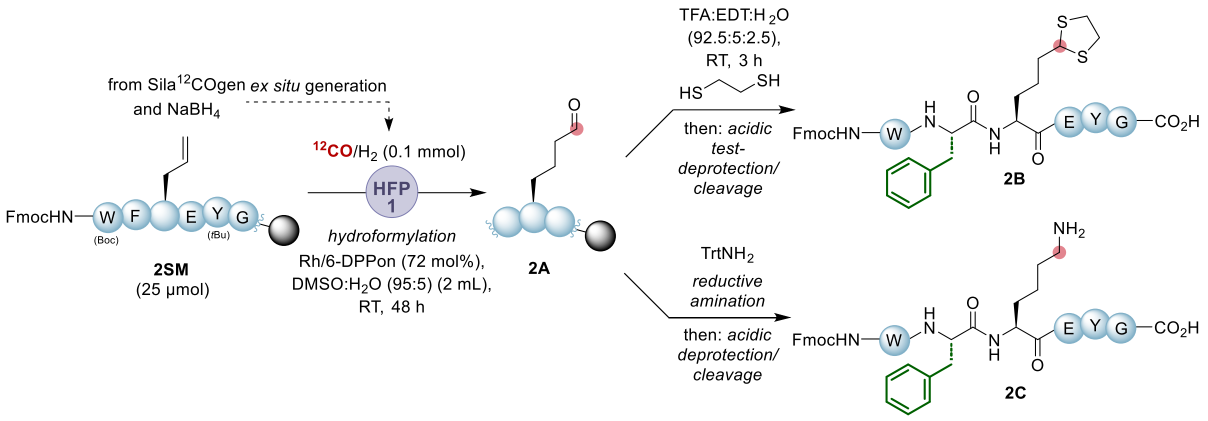

Entry	Change in hydroformylation-conditions	Conversion of **2SM**[a]	Yield of **2B**[a]	Yield of **2C**[b]
2	60 h	100%	98%	86%
3	36 mol%, 60 h	56%	52%	67%
8	Sila^{13}COgen, 36 mol% Rh/BIPHEPHOS (1:2.5), DMSO:THF:H$_2$O (17:3:1) (2 mL), 60 °C, 60 h	96%[c]	70%	61%[d]

Figure 12. *Hydroformylation:* Use of ligand BIPHEPHOS at elevated temperature on a 25 µmol scale of peptide **2SM**. *Reductive amination:* tritylamine in DMSO, 10% AcOH (0.12 M), RT, 2 h, then NaCNBH$_3$ in CH$_2$Cl$_2$:MeOH, 3:1 (0.15 M), RT, 1 h. *Deprotection and Cleavage*: TFA:H$_2$O:TiPS (triisopropylsilane):DODT (3,6-dioxa-1,8-octanedithiol) (92.5:2.5:2.5:2.5), RT, 3 h.[a]Determined by cleaving a test-portion of the resin after hydroformylation with TFA:EDT:H$_2$O (92.5:5:2.5), RT, 3 h, and analyzing thioacetal **B** and remaining **SM** by UPLC-UV purity. [b]Yield of **2C** determined by UPLC-UV purity analysis after cleavage with TFA:H$_2$O:TiPS:DODT (92.5:2.5:2.5:2.5). [c]96% conversion of **2SM**, but formation of 19% hydrogenated allylglycine residue. [d]Test cleavage with TFA:EDT:H$_2$O (92.5:5:2.5), RT, 3 h, shows presence of 10% remaining **[^{13}C]2B**.

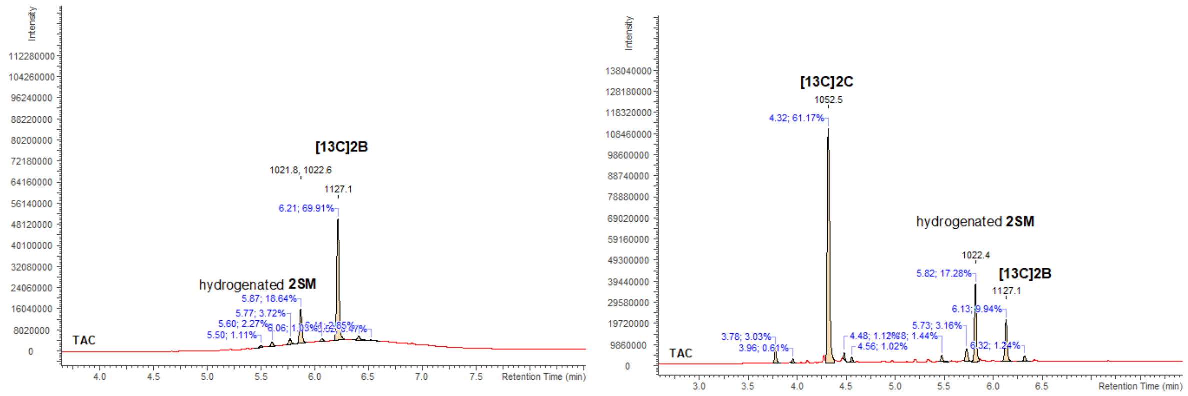

Figure 13. **Left:** UPLC-UV profile after hydroformylation using BIPHEPHOS, **Right:** UPLC-UV profile after reductive amination with tritylamine.

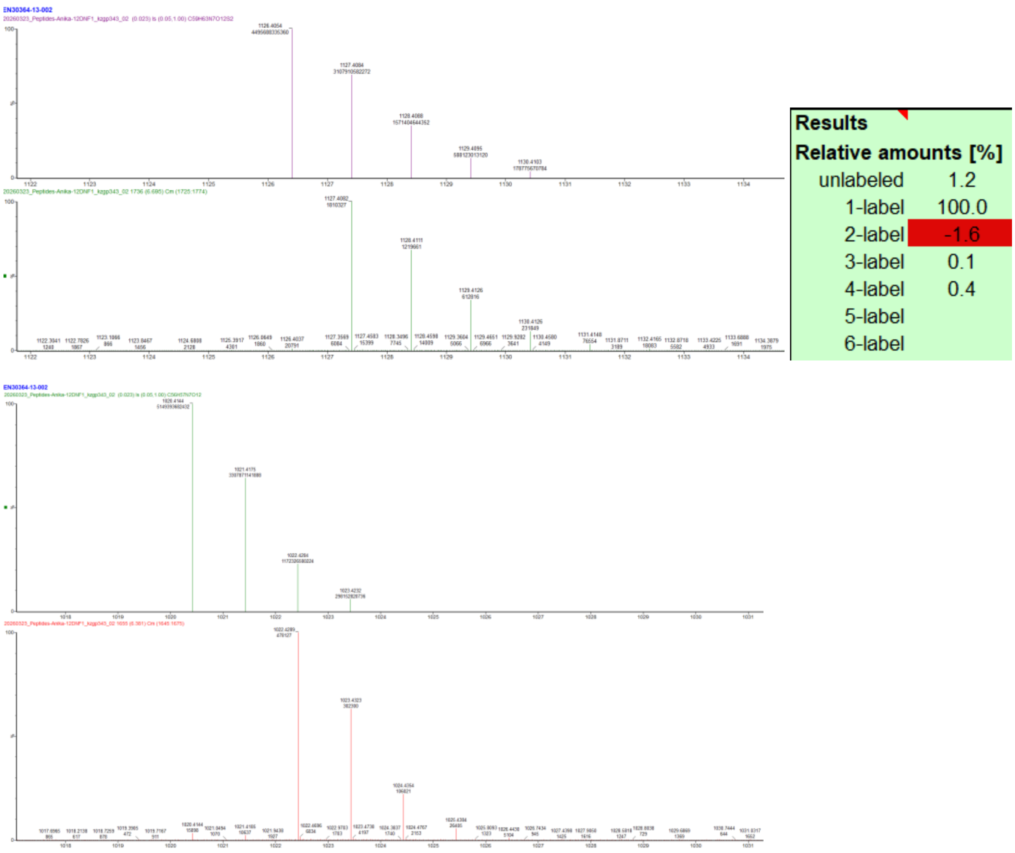

Figure 14. **Top:** HRMS analysis of the thioacetal [^{13}C]**2B** (bottom) vs. predicted [^{12}C]**2B** (top). **Bottom:** HRMS analysis of the hydrogenated starting material **2SM** (bottom) vs. predicted **2SM** (top).

4) On p. 19, the authors mention that Raj et al. presented allysine as a handle for reductive amination. That is correct, but Luo et al., Chem. Commun. 2012, 48, 1514-1516, did so before them, to my knowledge. Admittedly, on a smaller scope, but also using allysine. However, they achieved this through the oxidation of hydroxynorleucine with Dess-Martin periodinate. Nonetheless, this should be mentioned and cited.

Answer: We thank Reviewer 3 for making us aware of this report, which we agree is important to add for a complete literature background. Since we decided to completely omit the second part of the manuscript (Wittig-cascade functionalization, see Reviewer 2, comment 6), the citation will not be added in p.19 as suggested by Reviewer 3, but instead will be added already in the Introduction.

Changes in the manuscript: Addition of citation Luo et al. To the sentence “Recent oxidative and electrochemical methodologies enable its synthetic installation within peptides,[28-30] ...”

5) These were the minor points of criticism regarding the first part. I find the second part, or rather the last results chapter, to be very premature. The late-stage diversification using a hydroformylation-reductive amination-Wittig reaction cascade was only demonstrated for a very small scope of peptides and, at best, with a moderate yield. This stands in stark contrast to the excellently executed work presented in the first part of the manuscript. No optimization of reaction conditions, no HPLC traces in the manuscript, etc. In its current state, this is not publishable! Either the authors invest more time and present a mature study here, or they leave this part out entirely and publish it separately. Honestly, I consider the manuscript without this part to be publishable and of higher quality.

We thank Reviewer 3 for this remark. Please see answer to Reviewer 2, comment 6. We agree with both reviewers that keeping the second part would cause confusion since it is not relevant for the isotope labeling, and decided to completely omit the Wittig-cascade functionalization part.

Editorial requests and other changes by the authors:

- Few formatting and wording/spelling changes (shown in track changes)
- We also added this reference published recently in Nat. Comm. (March 2026): Martinelli, E., Weck, R., Güssregen, S. & Derdau, V. Radiolabeling of oligopeptides by selective hydrogen isotope exchange with deuterium and tritium in aqueous buffers. *Nat. Commun.* **17**, 2317 (2026).
- We also added this reference that uses tritylamine as an ammonia equivalent: Sharma, S. K. *et al.* Reductive amination with tritylamine as an ammonia equivalent: efficient preparation of the 5-(4-(9-fluorenylmethyloxycarbonyl)aminomethyl-3,5-dimethoxyphenoxy)valeric acid (PAL) handle for peptide synthesis. *J. Org. Chem.* **58**, 4993–4996 (1993)
- Addition of page numbers to this reference: Hammershøj, H. C. D. *et al.* Multi-carbon labelling of active pharmaceutical ingredients enabled by a three-gas surrogate hydroformylation. *Nat. Synth.* **2**, 243–250 (2023).
- In Figure 1 we removed the numbering of the subheadings since those were not according to the formatting standards of Nature Communications
- Changes in all figures:
  -the abbreviations HFP, RA and CD were replaced with their written out names.
  -the arrow heads were normalized (in general smaller)
- Changes in Scheme 1:
  -formatting of the pipette in Figure 1b
  -Figure 3: addition of "^{12}CO and ^{14}CO"/ "^{12}COgen and ^{14}COgen" to setup HFP2
- Addition of "Figure1c" behind "Current peptide radiolabeling strategies (Figure 1c)"
- Figure 3a: Addition of "0.05 ^{14}C/molecule" in the scheme for **[^{14}C]2C**
- Addition of names to the Acknowledgements section
- Supporting Information:
  - formatting/wordings
  - addition of GC-MS instrument description under chapter 1.2
  - in General Procedure GP4: addition of "(^{12}CO:^{14}CO, 95:5):H_2"
  - Table S12: Deuterium incorporation and influence of water; here we had a typo, change of 1-A to SI-8A
  - We added this sentence to the "General notes on reporting yield": "Isomerized starting material (internal olefin) or hydrogenated starting material may co-elute with the starting material containing the allylglycine residue, which could not be accounted for when reporting starting material conversion/ remaining starting material in crude UPLC-UV profile using low-resolution mass spectrometry.", and a similar one to the semaglutide analog optimization in the Supporting Information.
  - we deleted the last paragraph in Chapter 2.3 (iii), and replaced it with "The reason for varying Rh-catalysts influencing allysine cyclization to a different extent was not further investigated."
  - Table S11: remove "K26-Bn" in the scheme
- We added the following paragraphs to the manuscript as required by Nat. Comm.:

Data availability: The experimental procedures, UPLC-UV profiles and NMR data of this study are available in the Supporting Information.
Author contributions: The project was initiated by C.E., T.S, and H.C.D.H., and the reaction workflow was designed with A.S. The method was optimized by A.S., H.C.D.H., M.S.J.G., and V.J.E. The reaction scope was carried out by A.S. and M.S.J.G. Radiolabeling experiments were performed by A.S., peptide synthesis was supported by J.B., and HRMS analyses were carried out by L.S. The project was supervised by K.S.M, R.G., P.M.P., A.H., T.S., and C.E. The manuscript was conceptualized by A.S. and all authors contributed to the preparation and review of the work.
Correspondence should be addressed to Chad Elmore and Anika Schick.
